# Stable BF_2_ Boracycles as Versatile Reagents for Selective *Ortho* C–H Functionalization

**DOI:** 10.1002/anie.202518421

**Published:** 2026-01-16

**Authors:** Ganesh H. Shinde, Jonatan Babiker, Michelle Mebrahtu, Anaïs Prigent, Gauthier Foucras, Yogesh N. Aher, Francoise M. Amombo Noa, Magnus J. Johansson, Janez Košmrlj, Ross D. Jansen‐van Vuuren, Thomas Cailly, Henrik Sundén

**Affiliations:** ^1^ Department of Chemistry and Molecular Biology University of Gothenburg Gothenburg Sweden SE‐41296; ^2^ Université de Caen Normandie CERMN UR 4258 Caen 14000 France; ^3^ Department of Nuclear Medicine Centre François Baclesse Caen 14000 France; ^4^ Université de Caen Normandie CYCERON UAR 3408‐US50, IMOGERE Caen 14000 France; ^5^ Institut Blood and Brain @Caen Normandie (BB@C) Caen 14000 France; ^6^ Department of Nuclear Medicine CHU Côte de Nacre Caen 14000 France; ^7^ Department of Chemistry and Chemical Engineering Chalmers University of Technology Gothenburg Sweden SE‐41296; ^8^ Medicinal Chemistry, Research and Early Development, Cardiovascular, Renal and Metabolism (CVRM) Biopharmaceuticals R&D, AstraZeneca, Gothenburg Pepparedsleden 1 Mölndal 431 50 Sweden; ^9^ Faculty of Chemistry and Chemical Technology University of Ljubljana Večna pot 113 Ljubljana 1000 Slovenia

**Keywords:** BBr_3_, Boron, Cross‐coupling, Radioiodination, Late‐stage functionalization

## Abstract

The development of new boron reagents continues to play a crucial role in advancing modern organic synthesis, particularly in C–H functionalization and cross‐coupling reactions. Herein, we report a metal‐free, robust, and scalable multigram protocol for the synthesis of stable BF_2_ boracycles that require no column chromatography, providing a practical and efficient route to access this valuable boron species. The BF_2_ boracycles exhibit enhanced stability and reactivity, making them highly versatile intermediates for late‐stage diversification. They undergo *ipso*‐substitution to afford a wide array of derivatives, including halogenated (e.g., radioiodinated), hydroxylated, and azidated products. Furthermore, they display excellent reactivity in Suzuki–Miyaura cross‐coupling reactions, enabling both C(sp^2^)─C(sp^2^) and C(sp^2^)─C(sp^3^) bond formation. These results underscore the utility of BF_2_ boracycles as powerful tools for selective functionalization in pharmaceutical synthesis and beyond. Our work represents a significant advancement in organoboron chemistry, offering both a streamlined synthetic approach and broad applicability for complex molecule construction.

## Graphical abstract

We report a robust, metal‐free and scalable synthesis of stable BF_2_ boracycles via directed ortho CH borylation, delivering isolable, shelf‐stable reagents without chromatographic purification. These BF_2_ boracycles exhibit unique and tunable reactivity, enabling highly selective ipso‐functionalization across a broad range of transformations. Their exceptional stability, wide substrate scope, and superior reactivity establish BF_2_ boracycles as powerful alternatives to conventional organoboron reagents for pharmaceutical and radiochemical applications.

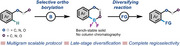

## Introduction

Organoboron reagents are indispensable tools in modern organic synthesis due to their exceptional versatility and broad applicability across a wide range of chemical transformations, including cross‐coupling reactions,^[^ ^1^, ^2^, ^3^ ^]^ radical‐mediated processes,^[^ ^4^, ^5^, ^6^, ^7^ ^]^ photochemical transformations,^[^ ^8^, ^9^, ^10^, ^11^ ^]^ and materials synthesis.^[^ ^12^, ^13^, ^14^ ^]^ Their widespread use is attributed not only to their reactivity but also to their favorable physicochemical properties. Furthermore, organoboron compounds are relatively stable, generally considered to be nontoxic,^[^ ^2^, ^3^ ^]^ and compatible with diverse reaction conditions. These attributes have contributed to the broad commercial availability of a wide array of boronic acids, which remain central to numerous synthetic applications.

**Figure 1:**
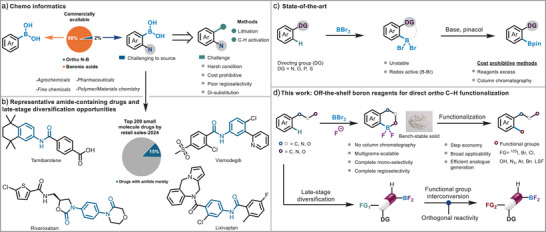
a) Sigma–Aldrich survey of commercially available boronic acids. b) Top 200 small‐molecule drugs by retail sales in 2024, based on data from the Njardarson Laboratory.^[^ ^18^ ^]^ c) State‐of‐the‐art for BBr_2_ to Bpin. d) This work: Development of BF_2_ complexes and their utility in late‐stage diversification.

Despite this utility, *ortho*‐substituted boronic acids remain significantly more challenging to access and are underrepresented in commercial catalogs (Figure 1a). This limitation is particularly pronounced for *ortho*‐*N*‐substituted boronic acids, whose synthesis typically involves multi‐step sequences, including *ortho*‐lithiation of anilides with pyrophoric *n*‐BuLi, borylation and subsequent hydrolysis.^[^ ^15^, ^16^, ^17^ ^]^ Such procedures pose limitations in terms of operational safety, scalability, and tolerance to functional groups, restricting their broader application in complex molecule synthesis and late‐stage functionalization.

Triggered by the high density of functional groups in small‐molecule drugs, many of which can serve as directing groups for metal coordination,^[^ ^19^, ^20^ ^]^ recent advances in metal‐catalyzed directed C–H borylation have addressed several limitations associated with traditional borylation methods.^[^ ^21^, ^22^, ^23^ ^]^ These strategies enable regioselective installation of boron functionalities under mild conditions and in the presence of a wide range of functional groups, making them particularly attractive for the late‐stage diversification of complex, drug‐like scaffolds. As such, they have significantly expanded the synthetic utility of organoboron chemistry in pharmaceutical and materials science.^[^ ^18^, ^24^, ^25^, ^26^, ^27^ ^]^

However, metal‐catalyzed protocols are not without their drawbacks. Regioisomeric mixtures and over‐functionalization remain common, especially in substrates bearing multiple or electronically ambiguous directing groups.^[^ ^20^, ^21^ ^]^ These complications can lead to reduced selectivity and yield and pose challenges in purification and downstream transformations, thereby limiting the broader applicability of these methods in the synthesis of highly functionalized or sensitive molecules.

In recent years, boron chemistry has progressed even further, with boron tribromide (BBr_3_) emerging as a key player in metal‐free approaches to intramolecular electrophilic aromatic *ortho* C–H borylation and borylation of alkenes (Figure 1c).^[^ ^28^, ^29^, ^30^, ^31^, ^32^, ^33^, ^34^, ^35^, ^36^, ^37^, ^38^, ^39^, ^40^, ^41^, ^42^, ^43^, ^44^, ^45^, ^46^, ^47^, ^48^, ^49^, ^50^, ^51^, ^52^, ^53^, ^54^ ^]^ This strategy, guided by donor atoms such as nitrogen,^[^ ^34^, ^35^, ^36^, ^37^, ^38^, ^39^, ^40^ ^]^ oxygen,^[^ ^41^, ^42^, ^43^, ^44^, ^45^, ^46^, ^47^, ^48^, ^49^, ^50^, ^51^, ^52^ ^]^ phosphorus,^[^ ^53^ ^]^ and sulfur,^[^ ^54^ ^]^ has enabled selective *ortho* C–H borylation, leading to the formation of highly reactive dibromoboracycles.

Inspired by these developments, we focused on *ipso*‐substitution and cross‐coupling reactions involving similar boracycle intermediates.^[^ ^55^, ^56^, ^57^, ^58^ ^]^ These transformations not only demonstrate the inherent reactivity and versatility of the dibromo‐boracycle motif (Figure 1b) but also highlight its potential in achieving highly regioselective *ortho* C–H functionalization. However, the boron bromine bond in dibromoboracycles (Figure 1c) is highly labile, prone to oxidative ligand exchange^[^ ^55^, ^56^ ^]^ or nucleophilic substitution at the boron,^[^ ^28^, ^29^, ^30^, ^31^, ^32^, ^33^, ^34^, ^35^, ^36^, ^37^, ^38^, ^39^, ^40^, ^41^, ^42^, ^43^, ^44^, ^45^, ^46^, ^47^, ^48^, ^49^, ^50^, ^51^, ^52^, ^53^, ^54^, ^55^, ^56^, ^57^, ^58^ ^]^ limiting their utility in late‐stage functionalization. While converting these boracycles into stable Bpin species is one solution, we sought a more robust strategy. Building on this foundation, we envisioned that site‐selectively formed dibromoboracycles could be converted into more stable difluoroboracycles. The strength of the boron‐fluorine bond enhances the overall stability of the boracycle and selectively weakens the adjacent boron‐carbon bond, allowing for *ipso*‐substitution at the carbon center.^[^ ^55^, ^56^ ^]^ Herein, we report the development of a robust and highly selective synthetic route to isolable BF_2_ boracycles that function as shelf‐stable reagents, significantly expanding the utility of organoboron chemistry. Given the widespread occurrence of classical directing groups in drug‐like molecules, this strategy provides a powerful platform for late‐stage diversification via boron‐mediated transformations.

## Results and Discussion

After identifying optimal conditions for introducing boron at the *ortho*‐position of anilide (**1a**) using BBr_3_, the primary objective of this study shifted toward determining the appropriate fluorine source and solvent to facilitate the formation of BF_2_ **3a**. We initially employed tetrafluoroborate as a nucleophilic fluoride source and chose methanol as the solvent due to solubility considerations. This approach yielded the desired product in 80% (Table S1, entry 1). The introduction of water to the reaction medium improved solubility, increasing the yield to 91% (Table S1, entry 2). Subsequently, using an acetonitrile solvent system further improved the reaction yield, resulting in a 95% yield of **3a**, which was better than the methanol system (Table S1, entry 3 versus entry 2). Reducing the fluoride source loading led to a slight drop in yield from 95% to 89% (Table S1, entry 4 versus entry 3,). Testing other tetrafluoroborate salts showed minimal variation in yields, with all providing comparable results (Table S1, entries 5 − 6,). Given availability and cost considerations, we selected NaBF_4_ as the preferred fluoride source. Alkaline fluoride sources, though effective, led to slower reactions and yields between 85% and 89% (Table S1, entries 7 − 9).

**Scheme 1:**
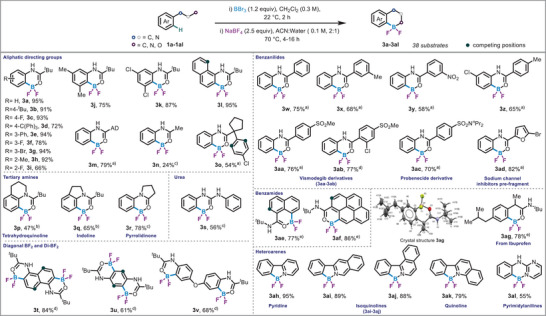
Reaction scope, conditions (**1a**‐**1k**, **1o**, **1q**): Step i) **1a**‐**1q** (0.6 mmol, 1 equiv.), BBr_3_ (0.72 mmol, 1.2 equiv.), in 2 mL anhydrous CH_2_Cl_2_ at 22 °C, 2 h; Step ii) NaBF_4_ (1.5 mmol, 2.5 equiv.) in 4 mL ACN and 2 mL distilled water, at 70 ^0^C for 4 h. ^a)^Step i) 40 °C, 16 h. ^b)^Step i) time 1 h. ^c)^Step i) 60 °C, 24 h. For product **3t**‐**3v**: Step i) Amide (0.6 mmol, 1 equiv.), BBr_3_ (1.8 mmol, 3 equiv.), in 2.5 mL anhydrous CH_2_Cl_2_ at 60 °C, 65 h; Step ii) NaBF_4_ (3 mmol, 5 equiv.). ^d)^Step i) 60 °C, 65 h. For **3ae**‐**3ag**: Step i) Amide (0.6 mmol, 1 equiv.), BBr_3_ (1.05 mmol, 1.75 equiv.), in 2.5 mL anhydrous CH_2_Cl_2_. ^e)^Step i) 60 °C, 16 h. For **3ah**‐**3ak**: **1ah**‐**1ak** (0.6 mmol, 1 equiv.), BBr_3_ (1.8 mmol, 3 equiv.), 2,6‐lutidine (1.2 mmol, 2 equiv.) in 2 mL anhydrous CH_2_Cl_2_, 40 °C, 16 h. For **3al**: **1al** (0.6 mmol, 1 equiv.), BBr_3_ (1.8 mmol, 3 equiv.), 2,3,5,6‐tetramethylpyrazine (0.72 mmol, 1.2 equiv.) in 2 mL anhydrous CH_2_Cl_2_ at 22 °C, 16 h.

After identifying the optimal fluorine source for difluoroboron formation, the scope of the reaction was explored with a diverse set of directing groups. Initially, a series of pivalamides (Scheme 1, **1a **− **1m**) were examined, demonstrating excellent tolerance for various functional groups. Both electron‐donating and electron‐withdrawing substituents at the *ortho*, *meta*, and *para*‐positions were well tolerated, resulting in yields ranging from 66% to 95% (Scheme 1, **3a **− **3i**). Disubstituted (**1j **− **1k**) and extended aromatic (**1l**) substrates also proved effective, delivering the desired products in 75% to 95%. Additionally, alternative acyl groups (**1m **− **1o**), such as adamantyl and other acyl derivatives, were accommodated by the reaction, with yields ranging from 24% to 79%. Substrates bearing tertiary amides (**1p**–**1r**) were also tested, and the reaction successfully tolerated these functional groups, yielding products in 47% to 78% yields. For tetrahydroquinoline (**1p**) and indoline (**1q**) derivatives, the reaction time was reduced to prevent the hydrolysis of the amide functional group, ensuring the amide bond remained intact. Notably, a substrate possessing a urea directing group (**1s**) provided the desired compound in 56% yield, which is significant as ureas are found in biologically active compounds, and their BF_2_ derivatives could serve as valuable substrates. Furthermore, urea derivatives are exceedingly difficult to functionalize with Directed *ortho* Metalation (D*o*M) as most metals bind efficiently to ureas hampering their catalytic activity. Additionally, diagonal double borylation was achieved on substrates **1t **and **1u**, producing the respective products in 84% and 61%. These compounds are important in materials chemistry, particularly in applications like organic field − effect transistors (OFETs), organic photovoltaics (OPVs), and organic light‐emitting diodes (OLEDs). Double borylation was also successful with substrate **1v**, providing **3v** in a yield of 68%.

Building on the success of the pivalamides and extended aromatic substrates, we next explored benzanilides to investigate site‐selective borylation on the aniline portion of the ring (Scheme 1, **1w **− **1ad**). Under the optimized conditions, a variety of substituents on the phenyl ring, including methyl (**1x**), nitro (**1y**), and substrates with modifications on both the aniline and benzyl portions (**1z**), were well tolerated, yielding the desired products (**3w **− **3z**) in 58% to 75%. Given the widespread occurrence of the anilide functional group in biologically active molecules, we extended the study to substrates with known pharmaceutical relevance, encompassing various levels of molecular complexity. Remarkably, these complex substrates were also well tolerated, delivering arylated products (**3aa **− **3ad**) in yields ranging from 70% to 82%, demonstrating the broad applicability of the reaction in the context of drug‐like molecules.

Following the successful installation of BF_2_ group on various anilides, we expanded our investigation to explore borylation on substrates with diverse directing groups. Specifically, carbonyl − directed substrates, including naphthyl (**1ae**), pyrene (**1af**), and pharmaceutically active compound ibuprofen (**1ag**) were subjected to the optimized conditions. These substrates demonstrated broad tolerance, delivering the corresponding aryl‐BF_2_ products in yields ranging from 77% to 86% (Scheme 1, **3ae **− **3ag**). The regioselectivity in **3ae** was determined by single‐crystal XRD (see Supporting Information section 9 for details). Further extending the scope, nitrogen‐containing directing groups were also examined (**1ah **− **1al**), while current transition‐metal (TM)‐catalyzed methods typically show limited applicability to nitrogen‐containing aromatic heterocycles,^[^ ^59^ ^]^ our protocol proved highly effective. Notably, substrates such as pyridine, isoquinoline, quinoline, and pyrimidylaniline furnished the desired difluoroboron products (**3ah **− **3al**) in good to excellent yields (55% − 95%). These results highlight the versatility and efficiency of the borylation strategy, particularly for challenging nitrogen‐based substrates. It is important to note, however, that the method's limitations are associated with the initial BBr_3_‐mediated borylation step not the subsequent BF_2_ formation, and these constraints are well‐documented in the literature.^[^ ^28^, ^29^, ^30^, ^31^, ^32^, ^33^, ^34^, ^35^, ^36^, ^37^, ^38^, ^39^, ^40^, ^41^, ^42^, ^43^, ^44^, ^45^, ^46^, ^47^, ^48^, ^49^, ^50^, ^51^, ^52^, ^53^, ^54^, ^55^, ^56^, ^57^, ^58^ ^]^

**Scheme 2:**
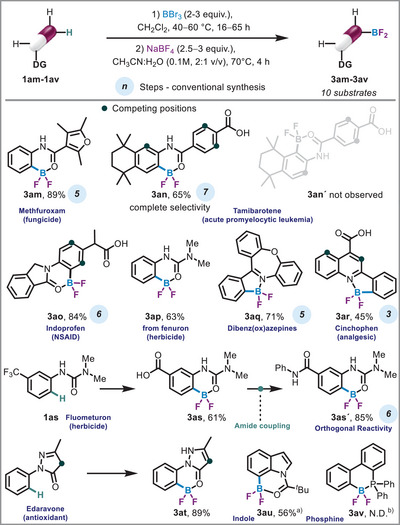
Late‐stage diversification. For condition refer Supporting Information Not Detected (N.D.). ^a)^KHF_2_ was used for fluorination. ^b)^BBr_2_ complex was observed.

Late‐stage functionalization strategies enable efficient diversification of complex molecules, including pharmaceutically relevant scaffolds and heteroaromatic systems. This approach expands the synthetic utility of functionalized cores, bridging applications in bioconjugation,^[^ ^60^ ^]^ drug discovery, biology‐oriented synthesis,^[^ ^60^, ^61^ ^]^ and materials science. To demonstrate this versatility, we evaluated complex substrates bearing diverse functional groups (Scheme 2). For instance, methfuroxam was well‐tolerated under the reaction conditions, affording the desired BF_2_ (**3am**) in excellent 89% yield. Notably, tamibarotene (**1an**), which contains four competing reactive sites, yielded a single regioisomeric BF_2_‐product **3an** in 65% yield, highlighting the exceptional selectivity of this transformation. Indoprofen underwent chemo‐ and regioselective amide‐directed borylation in the presence of a free carboxylic acid, delivering the BF_2_‐derivative **3ao** in 84% yield. Although TM‐catalyzed *β*‐C(*sp^3^*)−H functionalization of carboxylate has been reported in prior studies, our investigations did not observe either carboxylate‐directed ortho C–H functionalization or *β*‐C(*sp^3^*)−H activation.^[^ ^62^ ^]^ Urea‐based agrochemical such as fenuron **1ap** also participated efficiently, providing the corresponding BF_2_‐adduct **3ap** in 63% yield.

Given the therapeutic relevance of azepine‐containing scaffolds, we investigated dibenzoxazepine **1aq**, which underwent smooth borylation–fluorination to furnish the BF_2_‐product **3aq** in 71% yield (See Supporting Information for single‐crystal XRD). In the case of cinchophen, the reaction proceeded exclusively via quinoline‐directed borylation, yielding **3ar** in 45% yield. A particularly instructive example was observed with fluometuron **1as**: prolonged reaction times and excess BBr_3_ induced hydrolysis of the trifluoromethyl group, generating a carboxylic acid **3as** that was subsequently converted to an amide **3as´** without perturbing the BF_2_ moiety. This sequential functionalization exemplifies the orthogonal reactivity of BF_2_ groups and unlocks new avenues for late‐stage molecular editing. The robustness of this protocol was further demonstrated with edaravone **1at**, where despite observable tautomerization under Lewis acidic conditions, the BF_2_‐product **3at** was isolated in 89% yield (See Supporting Information section 9 for single‐crystal XRD). Furthermore, we achieved selective borylation at the C7‐position of the pivaloyl protected indole^[^ ^42^, ^63^ ^]^ **1au** to form BF_2_ derivative **3au** in 56% yield. The reaction under optimized conditions resulted in competitive hydrolysis and protodeborylation. However, by performing the second step at room temperature, these side reactions were suppressed. Diphenylphosphine was also investigated as a directing group^[^ ^53^ ^]^ but the BF_2_ product **3av** could not be obtained due to the low solubility and high stability of the intermediate BBr_2_ species **2av**. Collectively, these results establish the method as a powerful platform for late‐stage diversification of structurally intricate and functionally dense molecules, with immediate implications for medicinal chemistry and materials design.

To assess the efficiency of our protocol, we compared the step count of our synthetic route to that of a hypothetical alternative pathway leading to the corresponding boronic acid derivative (a synthetic equivalent of BF_2_) (Scheme 2 and Supporting Information Section 8). The boronic acid route typically involves 3–7 *de novo* steps in its longest linear sequence. In contrast, our route offers potential advantages in terms of step economy, convergence, and reduced solvent usage, all contributing to a lower environmental impact. This aligns with the well‐established principle that shorter, more convergent synthetic sequences with optimized complexity tend to produce less waste.^[^ ^64^ ^]^

**Scheme 3:**
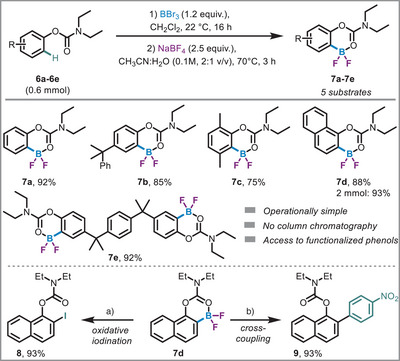
Reaction scope of carbamates. a) **7d** (0.15 mmol), Selectfluor, KI, ACN:Water at 22 °C for 2 h, then 65 °C, 5 h. b) **7d** (0.15 mmol), 1‐iodo‐4‐nitrobenzene, Pd(OAc)_2_, K_2_CO_3_, MeOH, 70 °C, 4 h.

Following the successful installation of BF_2_ groups on anilides and 2‐aryl heteroarenes, we turned our attention to phenols. Previous efforts to functionalize phenols were hampered by hydrolysis of the directing group on phenol derivatives.^[^ ^42^, ^56^ ^]^ We envisioned that a carbamate group^[^ ^65^ ^]^ would overcome this limitation. Indeed, this strategy proved highly effective, *O*‐carbamate protection enabled regioselective borylation with BBr_3_ of phenols (**6a–6e**) and the corresponding BF_2_s (**7a–7e**) could be isolated in excellent yields across a range of substrates (Scheme 3). The reaction tolerated various substrates, extended aromatic systems (**6b**, **6d**), and sterically hindered phenols (**6c**), and allowed for double borylation as demonstrated with substrate **6e**. Furthermore, we demonstrated the derivatization of the resulting *O*‐carbamate‐BF_2_ compound (**7d**) through iodination (**8**) and arylation (**9**), both of which proceeded in excellent yields (Scheme 3). This reactivity aligns well with the pattern observed for other BF_2_ species.

**Scheme 4:**
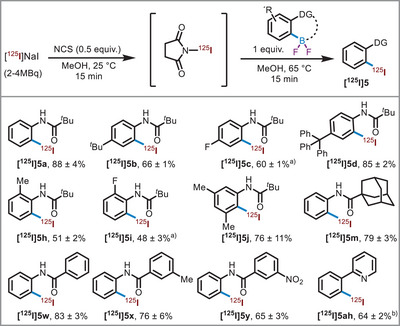
Reaction scope, radiodiodination of Ar‐BF_2_ compounds. RCCs were determined by radio‐HPLC and are shown for *n* = 2. ^a)^Reaction time in the second step was extended to 0.5 h. ^b)^RCC calculated for *n* = 4.

Organic compounds labeled with radioactive iodine isotopes ^123^I, ^124^I, ^125^I, or ^131^I are widely used in nuclear medicine for diagnostic imaging, radiotherapy, and radio‐assays in biomedical research and drug discovery.^[^ ^66^, ^67^, ^68^, ^69^, ^70^, ^71^ ^]^ In all these applications, NaI is the sole chemical source available to perform radioiodination. Given the relative stability of the C*sp^2^*−I bond, radioiodine is often incorporated within an arene moiety. In this context, we seized the opportunity to incorporate iodine‐125 from BF_2_ derivatives through a metal‐free oxidative iododeborylation process (Scheme 4).

Based on our previous reports on *ipso*‐halogenation of anilides and ureas,^[^ ^55^, ^56^ ^]^ where we utilized Selectfluor as an oxidant, we adapted a similar strategy here. However, in this case, *N*‐chlorosuccinimide (NCS) was chosen as the oxidant due to its superior solubility in methanol compared to Selectfluor (see Supporting Information section 4.1 for details). We developed a two‐step, one‐pot protocol (Scheme 4) involving the rapid formation of electrophilic [^125^I]NIS species from [^125^I]NaI and NCS followed by *ipso*‐radioiodination of aryl‐difluoroboranes **3**.

Using the optimized conditions, we then explored the scope of the reaction (Scheme 4). Several BF_2_s showed excellent incorporation of iodine‐125 with high radiochemical conversions (RCC). The radioiodination of pivalamide‐bearing anilides generally showed excellent incorporation of iodine‐125, yielding products with high efficiency. Substituents at the *para*, *ortho*, and *meta* positions, including electron‐donating groups, halogens, and extended aromatic systems, were well‐tolerated, providing the corresponding ^125^I‐labeled products in moderate to excellent conversions (Scheme 4, [^125^I]**5a**–**5ah**, 48%–88%). For fluorine‐containing substrates, such as **3c** and **3i**, a longer reaction time was required to achieve complete conversion. Additionally, the adamantyl‐containing substrate (**3m**) demonstrated efficient radioiodination, yielding the ^125^I‐labeled product in 79%. Furthermore, we explored benzanilide derivatives with various substituents on the benzoyl moiety. These substrates also exhibited excellent incorporation of iodine‐125, delivering the labeled products in high conversions (Scheme 4, [^125^I]**5w**–**5y**, 65%–83%). Notably, the protocol is also applicable for the radioiodination of pyridine‐based heteroarenes (Scheme 4, [^125^I]**5ah**, 64%). For comparison, when the same conditions were applied to the BBr_2_‐derivative (**2a**), the reaction proved sluggish and furnished the desired ^1^ ^2^ ^5^I‐labeled product ([^1^ ^2^ ^5^I]**5a**) in only 3% RCC (see Supporting Information, section 8.2.1). These results underscore the versatility of BF_2_s in this radioiodination protocol, which accommodates a wide range of functional groups and molecular frameworks while consistently providing high yields of the desired ^125^I‐labeled products.

**Scheme 5:**
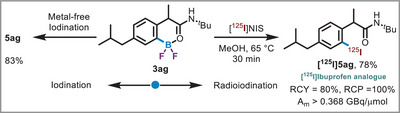
Late‐stage functionalization of Ibuprofen analogue. For radioiodination: [^125^I]NIS solution was prepared by mixing NaI (2–4 MBq) and NCS (0.5 equiv.) in MeOH at 25 °C for 15 min.

Subsequently, we conducted late‐stage radioiodination of an ibuprofen analogue **3ag** (Scheme 5, Supporting Information section 4.3). The results demonstrated highly efficient metal‐free iodination (**5ag**, 83%) and radioiodination (^125^I) reactions, yielding an impressive 80% radiochemical yield with 100% radiochemical purity. These findings highlight the potential of BF_2_ for use in advanced synthetic and radiochemical processes, as the same precursor can serve both as the radiolabeling agent and as the analytical standard for monitoring the radiochemical transformation. Given the versatility of iodine radioisotopes in imaging and therapeutic applications, this process appears well‐suited for the production of theranostic pairs.

**Scheme 6:**
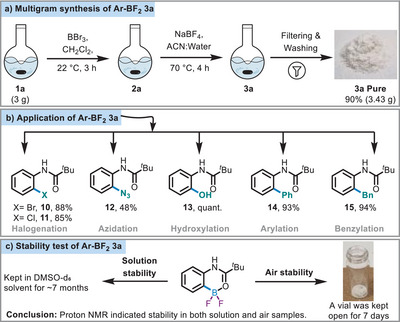
a) Multigram synthesis of **3a**. b) Application of Ar‐BF_2_ compound (for conditions refer Supporting Information section 6). c) Stability test of **3a**.

Following the exploration of the substrate scope for *ipso*‐radioiodination, we next investigated the scalability of BF_2_. We successfully synthesized the BF_2_ **3a** at a 1‐g scale, as well as at a multigram scale (3g, 90% yield), achieving excellent yields (Scheme 6a, Supporting Information section 5). The synthesis is robust and mild and the purification was achieved simply by washing with pentane to obtain the pure desired BF_2_ **3a**.

We next explored the application of Ar‐BF_2_ **3a** in various functionalization reactions. The BF_2_ **3a** can be functionalized through both metal‐free approaches (Schemes 4 and 5) and TM‐catalyzed reactions (Scheme 6b). Under copper catalysis, halogens (**10**–**11**) and azide (**12**) were successfully installed at the *ortho*‐position of *N*‐phenyl pivalamide (**1a**). The tunable reactivity of **3a** under metal‐free and TM conditions demonstrates the significance of BF_2_ complexes in novel synthetic transformations. Additionally, compound **3a** was oxidized to the corresponding hydroxyl product (**13**, Scheme 6b) in quantitative yields. Furthermore, we demonstrated the compatibility of **3a** in Suzuki–Miyaura cross‐coupling reactions. The BF_2_ **3a** was found to effectively participate in both C*sp^2^*–C*sp^2^* (**14**) and C*sp^2^*–C*sp^3^* (**15**) cross‐coupling reactions, thereby broadening its applicability in various catalytic processes (Scheme 6b).

Next, the stability of the newly synthesized BF_2_ **3a** was evaluated under both solution and solid‐state conditions (Scheme 6c, Supporting Information section 7). In the solution state, **3a** was dissolved in DMSO‐*d*_6_ and stored for approximately seven months at ambient temperature. Periodic monitoring by ^1^H spectroscopy revealed no signs of decomposition or the appearance of any new peaks, confirming the compound's high stability in solution (Supporting Information section 7.1). Additional derivatives of BF_2_s were also tested under similar conditions, remaining in DMSO‐*d*_6_ for 6–8 months (Supporting Information section 7.1.2). NMR analyses of these samples consistently showed clear spectra with no evidence of degradation, further supporting the robust solution stability of this class of compounds. The air stability of **3a** was investigated by leaving a solid sample exposed to air in an open vial for 7 days. Subsequent NMR spectroscopy revealed no signs of decomposition, confirming its stability under ambient atmospheric conditions. These results demonstrate the high chemical and environmental stability of BF_2_s, both in solution and as solids, making them promising candidates for applications in synthetic chemistry and related fields.

To demonstrate the unique utility of the BF_2_ moiety beyond its synthetic accessibility, we conducted a comparative reactivity study of three boron species: BBr_2_ (**2a**), BF_2_ (**3a**), and Bpin (**16**). These substrates were subjected to four distinct transformations such as, bromination, chlorination, dimerization, and cyclization under uniform reaction conditions (Table 1 and Supporting Information Section 8.1–8.3).

**Table 1:** Reactivity comparison study of BBr_2_, BF_2_, and Bpin.

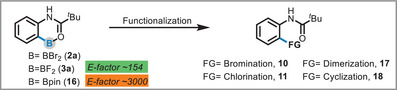				
Reaction 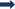				
Substrates 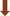	Bromination (**10**)^a)^ Yield%	Chlorination (**11**)^b)^ Yield%	Dimerization (**17**)^c)^ Yield%	Cyclization (**18**)^d)^Yield%
BBr_2_ (**2a**)	24%^e)^	10%^e)^	N.D.	N.D.
BF_2_ (**3a**)	88%^f)^	85%^f)^	85%^f)^	63%^f)^
Bpin (**16**)	68%^e)^	72%^e)^	71%^e)^	N.D.
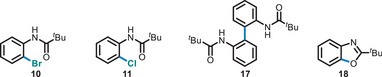				

^a)^ CuBr_2_, MeOH:Water, 85 °C, 22 h.

^b)^ CuCl_2_, MeOH:Water, 85 °C, 22 h.

^c)^ CuF_2_, MeOH, 60 °C, 16 h.

^d)^ Cu(OTf)_2_, KF, CH_3_CN, 80 °C, 3 h.

^e)^ Yield calculated by ^1^H NMR.

^f)^ Isolated yields. N.D: Not Detected. For detailed conditions, refer to the Supporting Information

The BBr_2_ species (**2a**) proved to be poorly reactive, affording the bromination product in only 24% yield (**10**) and the chlorination product in a mere 10% yield (**11**). Notably, neither the dimerization nor the cyclization reaction proceeded with this substrate. In contrast, the Bpin derivative (**16**) showed improved reactivity, yielding the bromination, chlorination, and dimerization (**17**) products in good yields as determined by NMR spectroscopy. However, it failed to produce the desired cyclization to the benzoxazole (**18**, Table 1 and Supporting Information section 8.3.6). The BF_2_ derivative **3a** exhibited superior and exceptional reactivity, delivering excellent isolated yields across all tested reactions (Table 1 and Supporting Information section 8.3.7). Most significantly, the successful dimerization and, in particular, the cyclization to the benzoxazole represents an unprecedented reaction manifold for the BX_2_ boracycles.

Next, the environmental efficiency of the synthetic routes was quantitatively assessed using the E‐factor system. While the synthesis of the Bpin derivative^[^ ^42^ ^]^ **16** resulted in a high E‐factor of 3000, indicating substantial waste generation, the route to the BF_2_ product **3a** proved markedly more sustainable, with an E‐factor of 154 (see Supporting Information, Section 7.2). This ∼20‐fold reduction in waste is partly attributable to the straightforward workup of our method, which involves a rapid and simple filtration and washing protocol. In contrast, the Bpin synthesis requires purification by column chromatography, which is substantially more solvent‐intensive. Consequently, our method offers not only an efficient transformation but also a significantly greener alternative.

In conclusion, the development of BF_2_s represents a significant advancement in boron chemistry, offering substantial improvements over traditional directed *ortho* boron reagents.^[^ ^28^, ^29^, ^30^, ^31^, ^55^, ^56^, ^57^, ^58^ ^]^ The robust and scalable synthesis method for BF_2_s, free from the need for column chromatography, ensures their practical applicability, especially in large‐scale synthesis. These compounds demonstrate remarkable stability both in solution and solid states, making them highly suitable for various synthetic applications. Their ability to undergo *ipso*‐functionalization with ease, combined with excellent reactivity in Suzuki–Miyaura cross‐coupling reactions, positions them as a powerful alternative to Bpin derivatives. Furthermore, the ability to activate BF_2_s under both metal‐free and metal‐catalyzed conditions broadens their reactivity scope, making them an attractive choice for future applications in complex molecule construction, including radiolabeling for imaging and therapeutic purposes. Overall, this work highlights the transformative potential of BF_2_s in late‐stage diversification strategies and paves the way for their broader use in organic synthesis and beyond. Further functionalization of *O*‐carbamates is currently being investigated in our laboratory.

## Author Contributions

Henrik Sundén supervised the overall project. Ganesh H. Shinde designed the study and conducted experimental work. Jonatan Babiker also carried out the experimental work for BF_2_s. Michelle Mebrahtu carried out experimental work for carbamates. Anaïs Prigent, Gauthier Foucras, and Thomas Cailly conducted radioiodination studies. Yogesh N. Aher conducted reactivity comparison study. The green parameter assessment was performed by Yogesh N. Aher and Ganesh H. Shinde. Magnus J. Johansson designed the LSF study. Francoise M. Amombo Noa performed the X‐ray structure analysis. Ross D. Jansen‐van Vuuren and Janez Košmrlj conceived the idea of carbamate borylation. Henrik Sundén and Ganesh H. Shinde cowrote the manuscript.

## Conflict of Interests

The authors declare no conflict of interest.

## Supporting information



Supporting Information

## Data Availability

The data that support the findings of this study are available in the Supporting Information of this article.

## References

[1] Selected review: A. Suzuki , Angew. Chem. Int. Ed. 2011, 50, 6722–6737, 10.1002/anie.201101379. 21618370

[2] A. J. J. Lennox , G. C. Lloyd‐Jones , Chem. Soc. Rev. 2014, 43, 412–443, 10.1039/C3CS60197H. 24091429

[3] J. W. B. Fyfe , A. J. B. Watson , Chem 2017, 3, 31–55. 10.1016/j.chempr.2017.05.008.

[4] C. Ollivier , P. Renaud , Chem. Rev. 2001, 101, 3415–3434, 10.1021/cr010001p. 11840989

[5] G. Yan , M. Yang , X. Wu , Org. Biomol. Chem. 2013, 11, 7999, 10.1039/c3ob41851k. 24150591

[6] D. Leifert , A. Studer , Chem. Rev. 2023, 123, 10302–10380, 10.1021/acs.chemrev.3c00212. 37578429

[7] R. Sang , J. E. Gestwicki , J. Am. Chem. Soc. 2025, 147, 23259–23269. 10.1021/jacs.5c07856. 40550745 PMC12232318

[8] G. Duret , R. Quinlan , P. Bisseret , N. Blanchard , Chem. Sci. 2015, 6, 5366–5382, 10.1039/C5SC02207J. 28717443 PMC5502392

[9] V. D. Nguyen , V. T. Nguyen , S. Jin , H. T. Dang , O. V. Larionov , Tetrahedron 2019, 75, 584–602, 10.1016/j.tet.2018.12.040. 31564756 PMC6764765

[10] F. Juli , D. Leonori , Chem. Rev. 2022, 122, 2292–2352, 10.1021/acs.chemrev.1c00558. 34882396

[11] C. Zhu , J. Lin , X. Bao , J. Wu , Nat. Commun. 2025, 16, 3225. 10.1038/s41467-025-58347-8. 40185738 PMC11971404

[12] D. L. Crossley , R. J. Kahan , S. Endres , A. J. Warner , R. A. Smith , J. Cid , J. J. Dunsford , J. E. Jones , I. Vitorica‐Yrezabal , M. J. Ingleson , Chem. Sci. 2017, 8, 7969–7977, 10.1039/C7SC02793A. 29568443 PMC5853289

[13] X. Yin , J. Liu , F. Jäkle , Chem. – A Eur. J. 2021, 27, 2973–2986, 10.1002/chem.202003481. 32852793

[14] Y. Y. Zhang , G. W. Yang , C. Lu , X. F. Zhu , Y. Wang , G. P. Wu , Chem. Soc. Rev. 2024, 53, 3384–3456. 10.1039/D3CS00115F. 38411207

[15] M. Lauer , G. Wulff , J. Organomet. Chem. 1983, 256, 1–9, 10.1016/S0022-328X(00)99290-8.

[16] P. Rocca , F. Marsais , A. Godard , G. Queguiner , Tetrahedron 1993, 49, 49–64. 10.1016/S0040-4020(01)80505-6.

[17] Recent monograph: Boronic Acids. Preparation, Applications in Organic Synthesis, (Ed.: D. G. Hall ), Wiley‐VCH, Weinheim, 2011. 10.1002/9783527639328.

[18] N. A. McGrath , M. Brichacek , J. T. Njardarson , J. Chem. Educ. 2010, 87, 1348–1349, 10.1021/ed1003806.

[19] Selected book and review. C.‐H. Bond , Activation in Organic Synthesis (Ed.: L. J. Jack ), CRC, Boca Raton, 2015.

[20] C. Sambiagio , D. Schönbauer , R. Blieck , T. Dao‐Huy , G. Pototschnig , P. Schaaf , T. Wiesinger , M. F. Zia , J. Wencel‐Delord , T. Besset , B. U. W. Maes , M. Schnürch , Chem. Soc. Rev. 2018, 47, 6603–6743. 10.1039/C8CS00201K. 30033454 PMC6113863

[21] Selected reviews A. Ros , R. Fernández , M. J. Lassaletta , Chem. Soc. Rev. 2014, 43, 3229–3243, 10.1039/C3CS60418G. 24553599

[22] L. Xu , G. Wang , S. Zhang , H. Wang , L. Wang , L. Liu , J. Jiao , P. Li , Tetrahedron 2017, 73, 7123–7157, 10.1016/j.tet.2017.11.005.

[23] R. Bisht , C. Haldar , M. M. M. Hassan , M. E. Hoque , J. Chaturvedi , B. Chattopadhyay , Chem. Soc. Rev. 2022, 51, 5042–5100. 10.1039/D1CS01012C. 35635434

[24] Selected reviews: L, X.u , S. Zhang , P. Li , Chem. Soc. Rev. 2015, 44, 8848–8858. 26393673 10.1039/c5cs00338e

[25] T. C. Wilson , T. Cailly , V. Gouverneur , Chem. Soc. Rev. 2018, 47, 6990–7005, 10.1039/C8CS00499D. 30140795

[26] R. J. Grams , W. L. Santos , I. R. Scorei , A. Abad‐García , C. A. Rosenblum , A. Bita , H. Cerecetto , C. Viñas , M. A. Soriano‐Ursúa , Chem. Rev. 2024, 124, 2441–2511, 10.1021/acs.chemrev.3c00663. 38382032

[27] R. de Jesus , K. Hiesinger , M. van Gemmeren , Angew. Chem. Int. Ed. 2023, 62, e202306659. 10.1002/anie.202306659. 37283078

[28] For review and highlight: S. A. Iqbal , J. Pahl , K. Yuan , M. J. Ingleson , Chem. Soc. Rev. 2020, 49, 4564–4591, 10.1039/C9CS00763F. 32495755

[29] S. Rej , N. Chatani , Angew. Chem. Int. Ed. 2022, 61, 202209539, 10.1002/anie.202209539. 35945136

[30] C. H. Yang , Org. Chem. Front. 2023, 10, 6010–6020, 10.1039/D3QO01487H.

[31] For highlight: D. Zhao , G. Yang , Synfacts 2023, 19, 0554.

[32] S. Tanaka , Y. Saito , T. Yamamoto , T. Hattori , Org. Lett. 2018, 20, 1828–1831, 10.1021/acs.orglett.8b00335. 29527895

[33] S. Li , C. Hu , X. Cui , J. Zhang , L. L. Liu , L. Wu , Angew. Chem. Int. Ed. 2021, 60, 26238–26245. 10.1002/anie.202111978. 34536251

[34] L. Niu , H. Yang , R. Wang , H. Fu , Org. Lett. 2012, 14, 2618–2621, 10.1021/ol300950r. 22548500

[35] S. Rej , N. Chatani , J. Am. Chem. Soc. 2021, 143, 2920–2929, 10.1021/jacs.0c13013. 33586953

[36] G. Wu , B. Pang , Y. Wang , L. Yan , L. Chen , T. Ma , Y. Ji , J. Org. Chem. 2021, 86, 5933–5942, 10.1021/acs.joc.1c00520. 33829798

[37] S. Rej , A. Das , N. Chatani , Chem. Sci. 2021, 12, 11447–11454. 34567499 10.1039/d1sc02937a PMC8409464

[38] G. Wu , X. Xu , S. Wang , L. Chen , B. Pang , T. Ma , Y. Ji , Y. , Chinese Chem. Lett., 2022, 33, 2005–2008.

[39] Z. Yang , L. Hao , X. Xu , Y. Wang , G. Wu , Y. Ji , Org. Lett. 2023, 25, 5875–5879, 10.1021/acs.orglett.3c02142. 37498107

[40] P. Kumar Someswara Ashwathappa , T. Higashi , V. Desrosiers , A. A. Omaña , F. G. Fontaine , Angew. Chem. Int. Ed. 2023, 62, 1–7. 10.1002/anie.202309295. 37535392

[41] S. A. Iqbal , J. Cid , R. J. Procter , M. Uzelac , K. Yuan , M. J. Ingleson , Angew. Chem. Int. Ed. 2019, 58, 15381–15385, 10.1002/anie.201909786. PMC6856876 31461213

[42] J. Lv , X. Chen , X. S. Xue , B. Zhao , Y. Liang , M. Wang , L. Jin , Y. Yuan , Y. Han , Y. Zhao , Y. Lu , J. Zhao , W. Y. Sun , K. N. Houk , Z. Shi , Nature 2019, 575, 336–340, 10.1038/s41586-019-1640-2. 31723273

[43] G. Wu , X. Fu , Y. Wang , K. Deng , L. Zhang , T. Ma , Y. Ji , Org. Lett. 2020, 22, 7003–7007, 10.1021/acs.orglett.0c02552. 32820932

[44] Z. J. Wang , X. Chen , L. Wu , J. J. Wong , Y. Liang , Y. Zhao , K. N. Houk , Z. Shi , Angew. Chem. Int. Ed. 2021, 60, 8500–8504, 10.1002/anie.202016573. 33449421

[45] S. A. Iqbal , M. Uzelac , I. Nawaz , Z. Wang , H. Jones , G. S. Nichol , K. Yuan , C. Millet , G. A. Chotana , M. J. Ingleson , Chem. Sci. 2023, 14, 3865–3872, 10.1039/D2SC06483A. 37035693 PMC10074396

[46] T. Wang , Z. J. Wang , M. Wang , L. Wu , X. Fang , Y. Liang , J. Lv , Z. Shi , Angew. Chem, Int. Ed 2023, 62, e202313205, 10.1002/anie.202313205. 37721200

[47] W. Jiang , J. Bai , J. Lv , Y. Zhao , C. Yan , Z. Shi , Synlett 2023, 34, 2220– 2226.

[48] R. Meena , S. Shekhar , S. B. Ansari , A. Tiwari , J. Lal , D. N. Reddy , Chem. – An Asian J. 2023, 18, 3–8. 10.1002/asia.202300638 37847482

[49] S. Maji , P. Rawal , A. Ghosh , K. Pidiyar , S. A. Al‐Thabaiti , P. Gupta , D. Maiti , 2024, JACS Au, 4, 3679–3689. 39328765 10.1021/jacsau.4c00660 PMC11423307

[50] W. Chen , J. Xia , J. Huang , L. Zhou , G. Wu , Org. Lett. 2024, 26, 4631–4636, 10.1021/acs.orglett.4c01244. 38780154

[51] S. Kang , J. Lv , Z. Shi , T. Wang , B. Wu , M. Wang , Nat. Commun. 2024, 15, 7380, 10.1038/s41467-024-51484-6. 39191737 PMC11350172

[52] Y. Liang , C. Du , C. Dong , J. Cao , Y. Xu , H. Zhang , 2025, Org. Lett. 27, 4650–4655. 10.1021/acs.orglett.5c00822. 40279296

[53] J. Lv , X. J. Zhang , M. Wang , Y. Zhao , Z. Shi , Chem. ‐ Eur. J. 2022, 28, e202104100. 34878200 10.1002/chem.202104100

[54] J. Lv , Y. Liang , Y. Ouyang , H. Zhang , Org. Lett. 2024, 26, 3709–3714. 10.1021/acs.orglett.4c00691. 38691629

[55] G. H. Shinde , H. Sundén , Chem. ‐ Eur. J. 2022, 29 e202203505. 36383388 10.1002/chem.202203505

[56] G. H. Shinde , G. S. Ghotekar , F. M. Amombo Noa , L. Öhrström , P.‐O. Norrby , H. Sundén , Chem. Sci. 2023, 14, 13429–13436, 10.1039/D3SC04628A. 38033885 PMC10685333

[57] G. H. Shinde , G. S. Ghotekar , H. Sundén , Chem. ‐ Eur. J. 2025, 31, e202403938, 10.1002/chem.202403938. 39513957

[58] G. H. Shinde , H. Castlind , G. S. Ghotekar , F. M. Amombo Noa , L. Öhrström , H. Sundén , Org. Lett. 2025, 27, 207–211, 10.1021/acs.orglett.4c04196. 39690449 PMC11731384

[59] J. H. Kim , T. Constantin , M. Simonetti , J. Llaveria , N. S. Sheikh , D. Leonori , Nature 2021, 595, 677–683, 10.1038/s41586-021-03637-6. 34015802

[60] M. Zheng , L. Kong , J. Gao , Chem. Soc. Rev. 2024, 53, 11888–11907, 10.1039/D4CS00750F. 39479937 PMC11525960

[61] S. Wetzel , R. S. Bon , K. Kumar , H. Waldmann , Angew. Chem. Int. Ed. 2011, 50, 10800–10826, 10.1002/anie.201007004. 22038946

[62] A. Uttry , S. Mal , M. Van Gemmeren , J. Am. Chem. Soc. 2021, 143, 10895–10901, 10.1021/jacs.1c06474. 34279928

[63] J. Lv , B. Zhao , Y. Han , Y. Yuan , Z. Shi , Chinese Chem. Lett. 2021, 32, 691–694, 10.1016/j.cclet.2020.06.028.

[64] L. Angelini , C. E. Coomber , G. P. Howell , G. Karageorgis , B. A. Taylor , Green Chem. 2023, 25, 5543–5556, 10.1039/D3GC00878A.

[65] R. D. Jansen‐van Vuuren , S. Liu , M. A. J. Miah , J. Cerkovnik , J. Košmrlj , V. Snieckus , Chem. Rev. 2024, 124, 7731–7828, 10.1021/acs.chemrev.3c00923. 38864673 PMC11212060

[66] A. W. Volkert , T. J. Hoffman , Chem. Rev. 1999, 99, 2269–2292, 10.1021/cr9804386. 11749482

[67] M. J. Adam , D. S. Wilbur , Chem. Soc. Rev. 2005, 34, 153, 10.1039/b313872k. 15672179

[68] S. L. Pimlott , A. Sutherland , Chem. Soc. Rev. 2011, 40, 149–162, 10.1039/B922628C. 20818455

[69] L. Zhu , K. Ploessl , H. F. Kung , Chem. Soc. Rev. 2014, 43, 6683–6691, 10.1039/C3CS60430F. 24676152 PMC4159420

[70] E. Dubost , H. McErlain , V. Babin , A. Sutherland , T. Cailly , J. Org. Chem. 2020, 85, 8300–8310, 10.1021/acs.joc.0c00644. 32369696

[71] D. S. Wilbur , M. J. Adam , Radiochim. Acta 2019, 107, 1033–1063, 10.1515/ract-2019-0004.

