# Supplementary material for: Stable BF2 Boracycles as Versatile Reagents for Selective Ortho C–H Functionalization
Source: Angew Chem Int Ed Engl. 2026 Jan 16;65(8):e18421. doi: 10.1002/anie.202518421 (PMC12910154; doi:10.1002/anie.202518421)

Supporting Information

Table of Contents

1. General information 2
2. Optimization table-S1 4
3. Experimental section 5
4. Radioiodination 32
5. Multigram synthesis of Ar−BF_2 3a 46
6. Applications Ar−BF_2 47
7. Stability study of Ar−BF_2s 53
7.2 Sustainability metrics 58
8. Proposed de novo synthesis 60
8.1 Comparison study of Ar−BBr_2 and Ar−BF_2 62
8.2 Reactivity comparison study of Ar−BBr_2, Ar−BF_2 63
9. Single crystal X-ray diffraction 74
10. References 84
11. NMR spectra of target compounds 85

1. General information

Experimental procedures, reagents, and glassware: All reactions were carried out in dry glassware under a nitrogen atmosphere using standard Schlenk techniques. A 25 mL microwave vial was used for the BF_2 reactions. A heating block was used for the heating source. 5 mL screw top V-Vial® was used for the application reactions. Analytically grade acetonitrile was purchased from VWR and anhydrous dichloromethane was purchased from Sigma Aldrich. Sodium tetrafluoroborate was purchased from Sigma Aldrich. Compounds **1ab**,[2] **1ae-1ag**[3] were synthesized according to the literature protocol. Compounds **1n**, **1r**, **1s**, **1w**, **1ah**, **1ai**, **1aj**, **1ak**, **1al**, **1ao**, **1ap**, **1ar**, **1as**, and **1at** were purchased from VWR. Compounds **1am** and **1an** were borrowed from AstraZeneca, Gothenburg, Sweden. Pivalamides,[1,2] and benzanilides[2] starting materials were synthesized according to literature protocols.[1,2] All reagents were used as received from commercial suppliers unless otherwise stated. Reported yields of final compounds are calculated based on the amide starting material without consideration of dibromoboraneyl complex formation.

Chromatography: Reaction progress was monitored by thin-layer chromatography (TLC) performed on aluminum plates coated with silica gel 60 F254 (Art 5715, 0.25 mm). Chromatograms were visualized with UV light at 254 nm or by staining using potassium permanganate.

Melting Points (MP): Melting points performed on solids were recorded on a Büchi M560 and are uncorrected.

Mass Spectrometry: High-resolution mass spectrometry (HRMS) data were recorded with a QExactive HF Orbitrap mass spectrometer interfaced with the Dionex Ultimate 3000 liquid chromatography system (Thermo Fisher Scientific). The sample was injected into a reversed-phase XBridge BEH C18 column (3.5 µm, 2.1x50 mm, Waters Corporation). The measurements were also performed by CMSI service at the Chalmers University of Technology. An Agilent 6520 equipped with an electrospray interface was operated in the positive and negative ionization mode

NMR Spectroscopy: All ^{1}H NMR and ^{13}C NMR, spectra were recorded using 600 MHz, 700 MHz and 800 MHz bruker spectrometers at 300K. Proton decoupled ^{19}F NMR spectra were recorded on 700 MHz. ^{11}B NMR was recorded on a 600 MHz Bruker spectrometer. Chemical shifts are given in parts per million (ppm, δ), referenced to the solvent peak of $CDCl_3$, defined at $\delta = 7.26$ ppm (^{1}H NMR) and $\delta = 77.16$ ppm (^{13}C NMR); $(CD_3)SO$ defined at $\delta = 2.50$ ppm (^{1}H NMR), $\delta = 39.52$ ppm (^{13}C NMR); Coupling constants are quoted in Hz (J). ^{1}H, ^{13}C, ^{19}F, and ^{11}B NMR splitting patterns are designated as singlet (s), doublet (d), triplet (t), quartet (q), bs (broad singlet) as they appeared in the spectrum. Splitting patterns that could not be interpreted or easily visualized are designated as multiplet (m).

Radioiodination: Sodium [^{125}I]iodide was purchased from Revvity as no carrier added [^{125}I]sodium iodide in 1x10^{-5} M aqueous NaOH. In each case, this was diluted with MeOH prior to use. Radio-HPLC analysis was performed with a Waters ARC HPLC system equipped with an autosampler, a Water 2998 Photodiode Array UV detector and LB 500 HERM GAMMA radiodetector. Radiochemical conversions were determined by integration of the observed peaks on the radio-chromatogramm. Identity of the radioiodinated molecules was assessed by comparison of retention times of standards on the UV-detector. The activities of [^{125}I]radiolabeled samples were determined using a radioisotope dose calibrator CRC-15R (Capintec) and standardized with a calibration source of ^{133}Ba (9.402 MBq from Eckert & Ziegler). All experiments involving radio-elements were performed in IMOGERE facilities (University of Caen). All experiments involving radioactive isotopes were carried out in dedicated radiochemistry laboratories authorized for handling unsealed radioactive sources, in full compliance with French national regulations and institutional safety procedures. Appropriate shielding, contamination controls, and personal protective equipment were employed throughout.

Radio-HPLC gradient: Water/acetonitrile containing 0.1% of formic acid, 0.8 mL/min,Waters XSelect HSS C18 column, 3.5 µm, 4.6 x 100 mm. 0−5 minute (5% to 50% MeCN) linear increase, 5−7 minute (50% MeCN) isocratic, 7−8 minute (50% MeCN to 95% MeCN) linear increase, 8−15 minute (95% MeCN) isocratic, 15−16 minute (95% to 5% MeCN) linear decrease, 16-19 minute (5% MeCN) isocratic.

2. Optimization table-S1

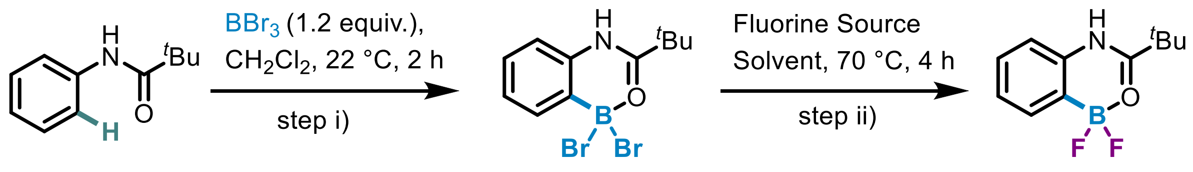

Entry	Fluorine Source (equiv.)	Solvent (ratio)	Yield[c] (**3a**)
1	$NaBF_4$ (2.5)	MeOH	80%
2	$NaBF_4$ (2.5)	MeOH:Water (2:1)	91%
3[a]	$NaBF_4$ (2.5)	ACN:Water (2:1)	95%
4	$NaBF_4$ (2.2)	ACN:Water (2:1)	89%
5	KBF_4 (2.5)	ACN:Water (2:1)	94%
6	$LiBF_4$ (2.5)	ACN:Water (2:1)	93%
7[b]	KF (2.5)	ACN:Water (2:1)	89%
8[b]	CsF (2.5)	ACN:Water (2:1)	86%
9	KHF_2 (2.5)	ACN:Water (2:1)	85%

[a] Reaction conditions: Step i) **1a** (0.6 mmol), BBr_3 (0.72 mmol), in 2 mL anhydrous CH_2Cl_2 at 22 ºC, 2 h; Step ii) $NaBF_4$ (1.5 mmol) in 4 mL ACN and 2 mL distilled water, at 70 ºC for 4 h; Entries 1-9: 0.1M solvent was used; [b] step ii) time 6 h; [c] Isolated yields.

3. Experimental section

3.1 Starting material spectral data:

2-chloro-4-(methylsulfonyl)-*N*-phenylbenzamide (1ab):

The starting material **1ab** was synthesized by following the literature procedure[2] using Aniline (1eq.), 2-chloro-4-(methylsulfonyl)benzoic acid (1.2 eq.), EDCI (1.4 eq.), 4-Dimethylaminopyridine (0.1 eq.), in CH_2Cl_2, 0 ºC to rt for 16 h.

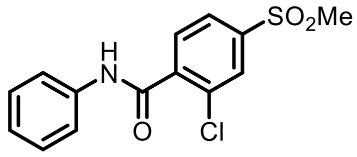

1ab

Off white solid; **Rf:** 0.16 (hexane/EtOAc, 70:30); **^{1}H NMR (600 MHz, $CDCl_3$) δ=** 7.99 (s, 1H), 7.97 (bs, 1H), 7.88 (d, *J* = 1.1 Hz, 2H), 7.68 – 7.62 (m, 2H), 7.43 – 7.38 (m, 2H), 7.23 – 7.19 (m, 1H), 3.08 (s, 3H); **^{13}C{^{1}H} NMR (151 MHz, $CDCl_3$) δ=** 163.1, 143.3, 140.6, 137.2, 132.3, 131.2, 129.4 (2C), 126.2, 125.6, 120.4, 44.6; **HRMS (ESI) (m/z):** calculated for $[M+H]^+$ $C_{14}H_{12}ClNO_3S^+$ 310.02992; found 310.02927.

(1,4-phenylenebis(propane-2,2-diyl))bis(4,1-phenylene) bis(diethylcarbamate) (6e):

The starting material **1ab** was synthesized by following the literature procedure[14] using 4,4'-(1,4-phenylenebis(propane-2,2-diyl))diphenol (1 eq.), NaH (60% suspension in mineral oil, 3 eq.) and diethylcarbamoyl chloride (2.4 eq.) in THF 0 ºC to rt for 16 h.

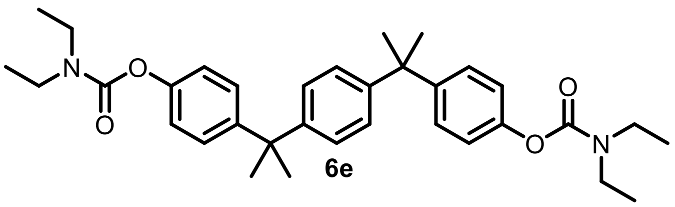

6e

Off white solid; **Rf:** 0.53 (hexane/EtOAc, 70:30); **^{1}H NMR (600 MHz, $CDCl_3$) δ=** 7.22 – 7.18 (m, 4H), 7.09 (s, 4H), 7.02 – 6.97 (m, 4H), 3.45 – 3.35 (m, 8H), 1.64 (s, 12H), 1.26 – 1.17 (m, 12H); **^{13}C{^{1}H} NMR (151 MHz, $CDCl_3$) δ=** 154.5, 149.4, 147.8, 147.6, 127.8, 126.5, 121.1, 42.4, 42.4, 42.1, 30.9, 14.4, 13.6; **HRMS (ESI) (m/z):** calculated for $[M+H]^+$ $C_{34}H_{44}N_2O_4^+$ 545.33738; found 545.33649.

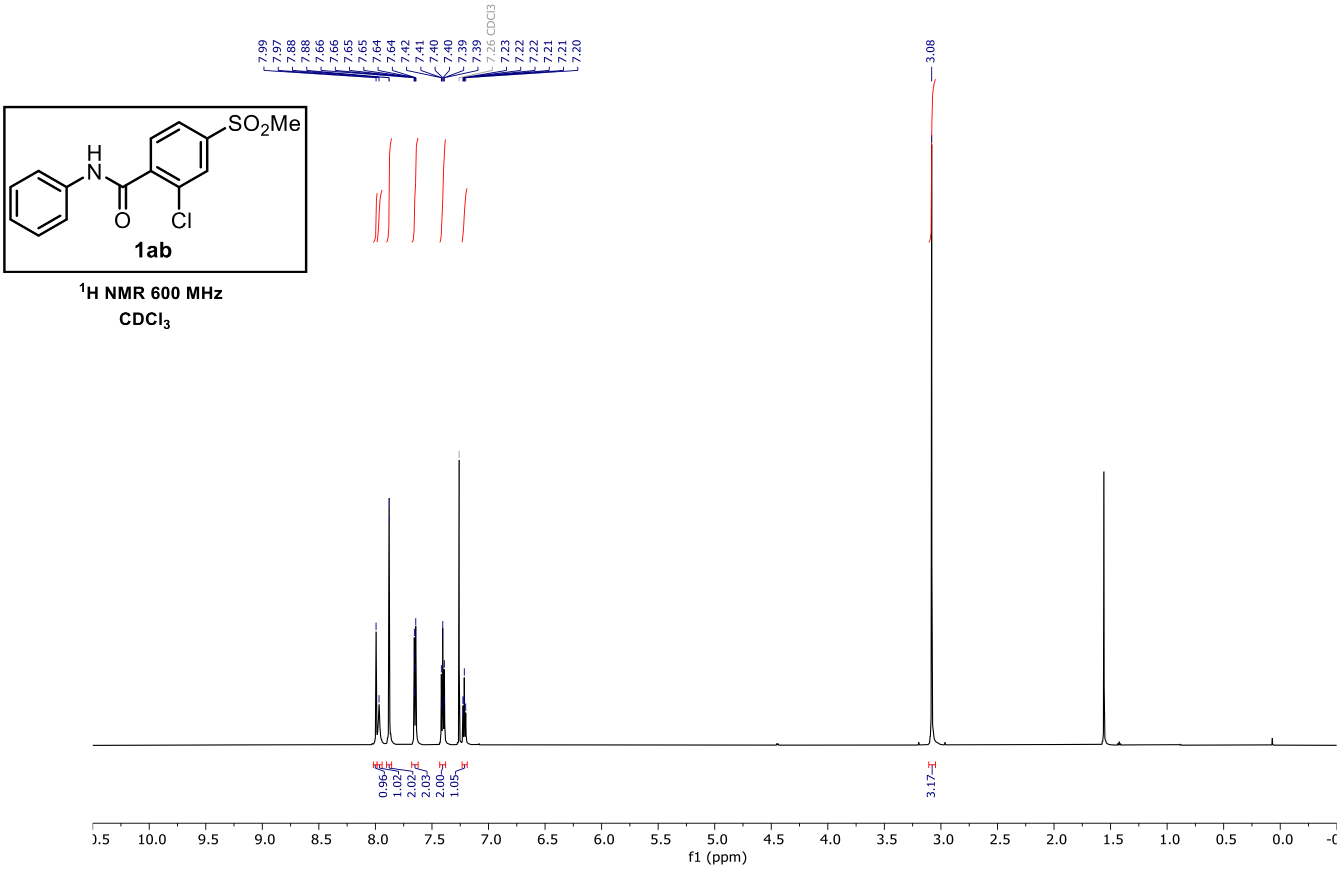
SO2Me
H
N
O
Cl
1ab
1H NMR 600 MHz
CDCl3
7.99
7.97
7.88
7.88
7.66
7.66
7.65
7.65
7.64
7.64
7.42
7.41
7.40
7.40
7.39
7.39
7.26 CDCl3
7.23
7.22
7.22
7.22
7.21
7.21
7.20
3.08
0.96
1.02
2.02
2.03
2.00
1.05
3.17
f1 (ppm)

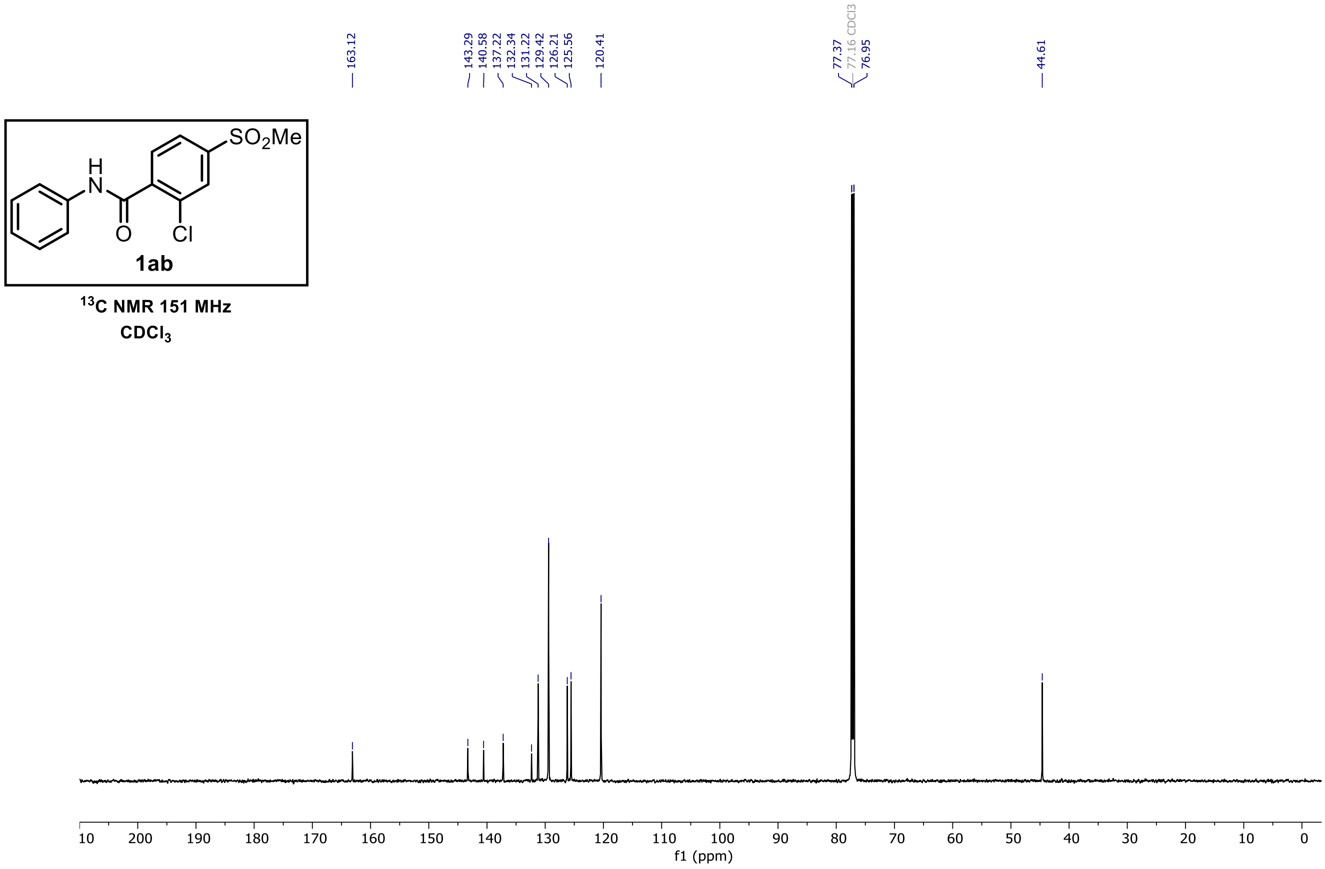
SO2Me
H
N
O
Cl
1ab
13C NMR 151 MHz
CDCl3
163.12
143.29
140.58
137.22
132.34
131.22
129.42
126.21
125.56
120.41
77.37
77.16 CDCl3
76.95
44.61
f1 (ppm)

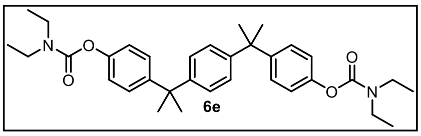

^{1}H NMR 600 MHz
$CDCl_3$

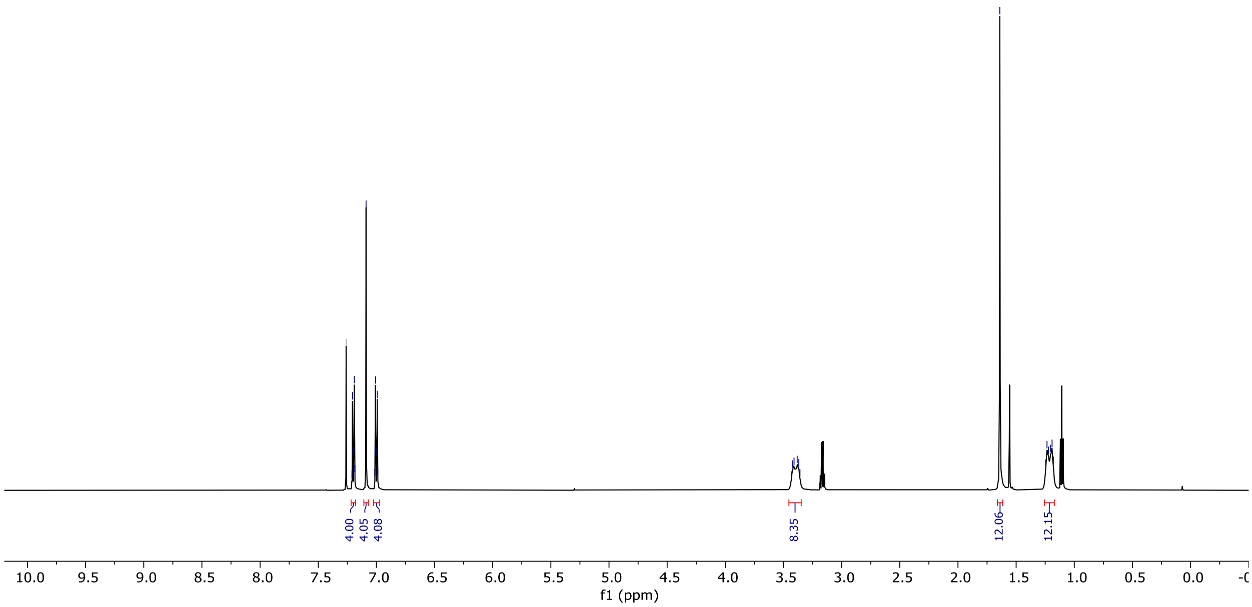

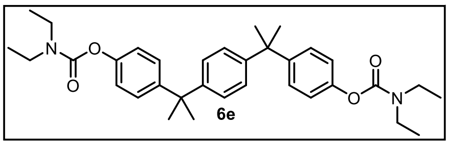

^{13}C NMR 151 MHz
$CDCl_3$

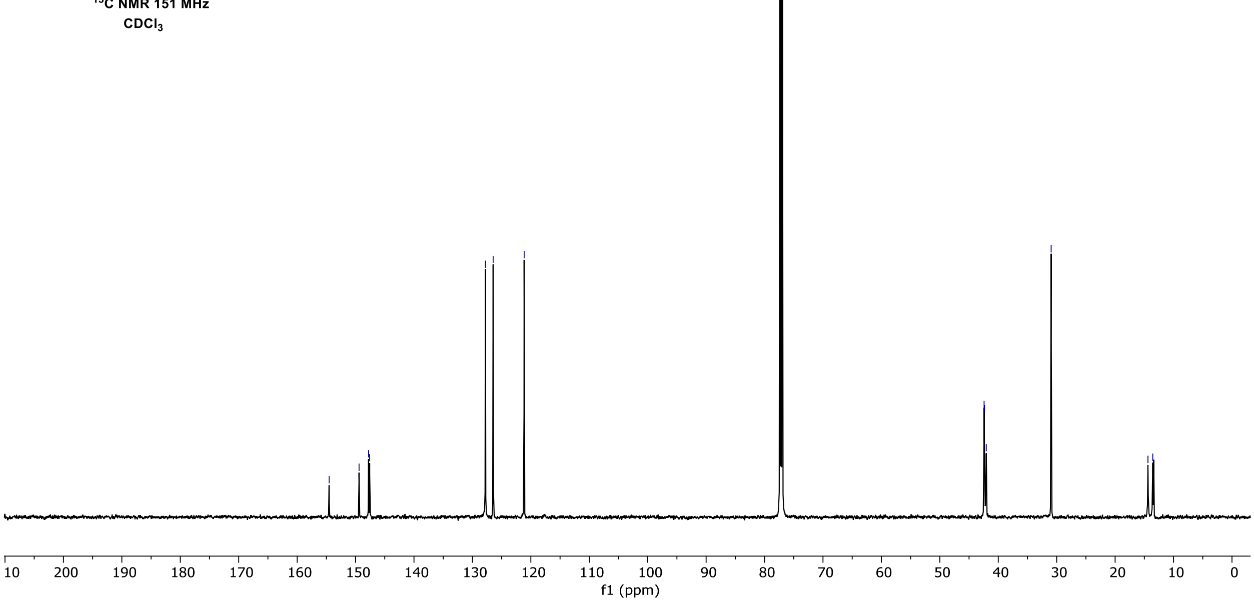

3.2 General Procedure A for reaction optimization: (3a-3v)

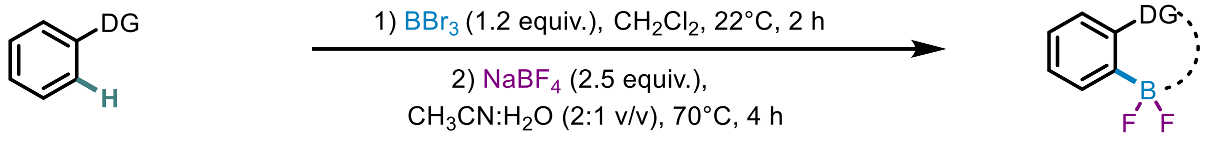

Step i) To a dry 25 mL microwave vial, equipped with a rubber septum, stir bar, the amide derivative (0.6 mmol, 1 equiv.) in anhydrous CH_2Cl_2 (2 mL) under a nitrogen atmosphere was added dropwise BBr_3 (720 µl, 0.72 mmol, 1.2 equiv., 1M solution in CH_2Cl_2). After the complete addition of BBr_3, the reaction mixture was stirred at 22 ºC for 2 h after which the solvent was removed under reduced pressure.

Step ii) To the crude residue from step i) were added $NaBF_4$ (164.69 mg, 1.5 mmol, 2.5 equiv.), 4 mL acetonitrile, 2 mL distilled water and the reaction mixture was heated at 70 ºC for 4 h. The reaction was allowed to reach room temperature and the acetonitrile was evaporated under *vacuo* to afford the crude solid. After removal of complete acetonitrile, the crude solid was taken in 20 mL distilled water and filtered. Additional 30 mL water wash was given to remove inorganic impurities. The crude solid was washed with pentane to remove unreacted starting material.

Note: The crude product can also be purified by stirring the mixture in pentane.

3.3 Spectral data

***N*-(2-(difluoroboraneyl)phenyl)pivalamide (3a):**[2]

Following the general procedure A using *N*-phenylpivalamide (106.35 mg, 0.6 mmol, 1 equiv.), BBr_3 (720 µL, 0.72 mmol, 1.2 equiv. 1M in CH_2Cl_2), in 2 mL CH_2Cl_2 at 22 ºC for 2 h and $NaBF_4$ (164.69 mg, 1.5 mmol, 2.5 equiv.) at 70 ºC, for 4 h. The crude product was purified by pentane wash and the desired product was obtained as an off white solid (128.3 mg, 95%); **^{1}H NMR (700 MHz, DMSO–d$_6$) δ=** 11.75 (s, 1H), 7.47 – 7.43 (m, 2H), 7.37 (td, J = 7.4, 1.6 Hz, 1H), 7.28 (td, J = 7.3, 1.1 Hz, 1H), 1.37 (s, 9H); **^{13}C{^{1}H} NMR (151 MHz, DMSO–d$_6$) δ=** 178.0, 136.5, 130.6, 128.2, 126.9, 116.9, 38.5, 26.6; **^{19}F NMR= (659 MHz, DMSO–d$_6$) δ= -**129.04, -129.11; **^{11}B NMR (193 MHz, DMSO–d$_6$) δ=** 2.98.

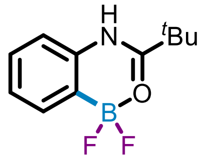

3a

***N*-(4-(*tert*-butyl)-2-(difluoroboraneyl)phenyl)pivalamide (3b):**

Following the general procedure A using *N*-(4-(*tert*-butyl)phenyl)pivalamide (140.01 mg, 0.6 mmol, 1 equiv.), BBr_3 (720 µL, 0.72 mmol, 1.2 equiv. 1M in CH_2Cl_2), in 2 mL CH_2Cl_2 at 22 ºC for 2 h and $NaBF_4$ (164.69 mg, 1.5 mmol, 2.5 equiv.) at 70 ºC, for 4 h. The crude product was purified by pentane wash and the desired product was obtained as an off white solid (153.1 mg, 91%); **Rf:** 0.29 (hexane/EtOAc, 70:30); **Mp:** 316-318 ºC; **^{1}H NMR (600 MHz, DMSO–d$_6$) δ=** 11.69 (bs, 1H), 7.44 (d, J = 2.2 Hz, 1H), 7.42 – 7.36 (m, 2H), 1.36 (s, 9H), 1.28 (s, 9H); **^{13}C{^{1}H} NMR (151 MHz, DMSO–d$_6$) δ=** 177.5, 149.2, 134.2, 126.6, 125.2, 116.7,

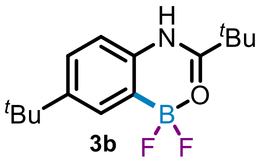

3b

38.4, 34.3, 31.2, 26.6; **^{19}F NMR= (659 MHz, DMSO–d$_6$) δ=** -129.14, -129.21; **^{11}B NMR (193 MHz, DMSO–d$_6$) δ=** 3.12; **HRMS (ESI) (m/z):** calculated for [M-H]$^-$ $C_{15}H_{21}BF_2NO^-$ 280.1689; found 280.1690.

***N*-(2-(difluoroboraneyl)-4-fluorophenyl)pivalamide (3c):**

Following the general procedure A using *N*-(4-fluorophenyl)pivalamide (117.14 mg, 0.6 mmol, 1 equiv.), BBr_3 (720 µL, 0.72 mmol, 1.2 equiv. 1M in CH_2Cl_2), in 2 mL CH_2Cl_2 at 22 ºC for 2 h and $NaBF_4$ (164.69 mg, 1.5 mmol, 2.5 equiv.) at 70 ºC, for 4 h. The crude product was purified by pentane wash and the desired product was obtained as an off white solid (135.6 mg, 93%); **Rf:** 0.20 (hexane/EtOAc, 70:30); **Mp:** 265-267ºC; **^{1}H NMR (600 MHz, DMSO–d$_6$) δ=** 11.84 (bss, 1H), 7.52 (dd, *J* = 8.9, 4.5 Hz, 1H), 7.19 (td, *J* = 8.8, 3.0 Hz, 1H), 7.14 (dd, *J* = 8.3, 3.0 Hz, 1H), 1.36 (s, 9H); **^{13}C{^{1}H} NMR (151 MHz, DMSO–d$_6$) δ=** 177.8, 160.8 (d, *J* = 245.0 Hz), 132.8, 119.8 (d, *J* = 7.8 Hz), 116.1 (d, *J* = 19.9 Hz), 115.5 (d, *J* = 23.7 Hz), 38.5, 26.6; **^{19}F NMR= (659 MHz, DMSO–d$_6$) δ=** -114.57 (F), -129.73 (BF_2), -129.81 (BF_2); **^{11}B NMR (193 MHz, DMSO–d$_6$) δ=** 2.60; **HRMS (ESI) (m/z):** calculated for [M-H]$^-$ $C_{11}H_{12}BF_3NO^-$ 242.0969; found 242.0966.

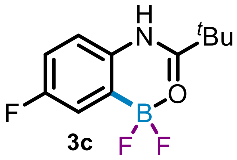

3c

***N*-(2-(difluoroboraneyl)-4-tritylphenyl)pivalamide (3d):**

Following the general procedure A using *N*-(4-tritylphenyl)pivalamide (251.72 mg, 0.6 mmol, 1 equiv.), BBr_3 (720 µL, 0.72 mmol, 1.2 equiv. 1M in CH_2Cl_2), in 2 mL CH_2Cl_2 at 22 ºC for 2 h and $NaBF_4$ (164.69 mg, 1.5 mmol, 2.5 equiv.) at 70 ºC, for 4 h. The crude product was purified by pentane wash and the desired product was obtained as an off white solid (200.7 mg, 72%); **Rf:** 0.29 (hexane/EtOAc, 70:30); **Mp:** 292-294 ºC; **^{1}H NMR (600 MHz, DMSO–d$_6$) δ=** 11.81 (bs, 1H), 7.38 (d, *J* = 8.6 Hz, 1H), 7.31–7.27 (m, 7H), 7.21 (t, *J* = 7.4 Hz, 3H), 7.14 (d, *J* = 8.0 Hz, 7H), 1.36 (s, 9H); **^{13}C{^{1}H} NMR (151 MHz, DMSO–d$_6$) δ=** 177.9, 146.4, 144.9, 134.4, 131.9, 130.9, 130.5, 127.7, 125.9, 116.4, 64.4, 38.4, 26.6; **^{19}F NMR= (659 MHz, DMSO–d$_6$) δ=** -128.96, -129.03; **^{11}B NMR (193 MHz, DMSO–d$_6$) δ=** 2.90; **HRMS (ESI) (m/z):** calculated for [M-H]$^-$ $C_{30}H_{27}BF_2NO^-$ 466.2159; found 466.2163.

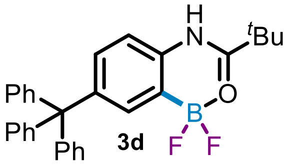

3d

***N*-(4-(difluoroboraneyl)-[1,1'-biphenyl]-3-yl)pivalamide (3e):**

Following the general procedure A using *N*-([1,1'-biphenyl]-3-yl)pivalamide (152 mg, 0.6 mmol, 1 equiv.), BBr_3 (720 µL, 0.72 mmol, 1.2 equiv. 1M in CH_2Cl_2), in 2 mL CH_2Cl_2 at 22 ºC for 2 h and $NaBF_4$ (164.69 mg, 1.5 mmol, 2.5 equiv.) at 70 ºC, for 4 h. The crude product was purified by pentane wash and the desired product was obtained as an off white solid (170 mg, 94%); **Rf:** 0.35 (hexane/EtOAc, 70:30); **Mp:** 256-258 ºC; **^{1}H NMR (600 MHz, DMSO–d$_6$) δ=** 11.76 (bs, 1H), 7.77 (d, *J* = 1.7 Hz, 1H), 7.67 – 7.63 (m, 2H), 7.60

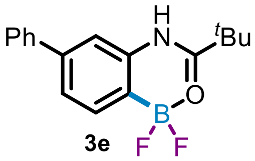

3e

(dd, $J = 7.6$, 1.6 Hz, 1H), 7.54 (d, $J = 7.6$ Hz, 1H), 7.50 (t, $J = 7.7$ Hz, 2H), 7.40 (t, $J = 7.4$ Hz, 1H), 1.40 (s, 9H); **$^{13}C\{^{1}H\}$ NMR (151 MHz, DMSO–d_6) δ=**178.3, 140.2, 139.6, 137.1, 131.3, 129.1, 127.8, 126.5, 125.3, 115.2, 38.6, 26.6; **^{19}F NMR= (659 MHz, DMSO–d_6) δ=** -129.13, -129.21; **^{11}B NMR (193 MHz, DMSO–d_6) δ=** 3.04; **HRMS (ESI) (m/z):** calculated for $[M\text{-}H]^-$ $C_{17}H_{17}BF_2NO^-$ 300.1376; found 300.1377.

***N*-(2-(difluoroboraneyl)-5-fluorophenyl)pivalamide (3f):**

Following the general procedure A using *N*-(3-fluorophenyl)pivalamide (117.14 mg, 0.6 mmol, 1 equiv.), BBr_3 (720 µL, 0.72 mmol, 1.2 equiv. 1M in CH_2Cl_2), in 2 mL CH_2Cl_2 at 22 ºC for 2 h and $NaBF_4$ (164.69 mg, 1.5 mmol, 2.5 equiv.) at 70 ºC, for 4 h. The crude product was purified by pentane wash and the desired product was obtained as an off white crystals (114 mg, 78%); **Rf:** 0.31 (hexane/EtOAc, 70:30); **Mp:** 266-268 ºC; **^{1}H NMR (600 MHz, DMSO–d_6) δ=** 11.83 (bs, 1H), 7.47 (dd, $J = 8.2$, 6.8 Hz, 1H), 7.29 (dd, $J = 10.7$, 2.4 Hz, 1H), 7.13 (ddd, $J = 9.2$, 8.2, 2.4 Hz, 1H), 1.37 (s, 9H); **$^{13}C\{^{1}H\}$ NMR (151 MHz, DMSO–d_6) δ=** 178.9, 161.7 (d, $J = 242.3$ Hz), 137.6 (d, $J = 10.3$ Hz), 132.7 (d, $J = 8.4$ Hz), 114.1 (d, $J = 19.9$ Hz), 104.1 (d, $J = 24.9$ Hz), 38.6, 26.5; **^{19}F NMR= (659 MHz, DMSO–d_6) δ=** -112.78 (F), -128.57 (BF_2), -128.64 (BF_2); **^{11}B NMR (193 MHz, DMSO–d_6) δ=** 2.88; **HRMS (ESI) (m/z):** calculated for $[M\text{-}H]^-$ $C_{11}H_{12}BF_3NO^-$ 242.0969; found 242.0968.

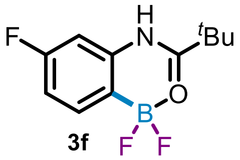

3f

***N*-(5-bromo-2-(difluoroboraneyl)phenyl)pivalamide (3g):**

Following the general procedure A using *N*-(3-bromophenyl)pivalamide (153.68 mg, 0.6 mmol, 1 equiv.), BBr_3 (720 µL, 0.72 mmol, 1.2 equiv. 1M in CH_2Cl_2), in 2 mL CH_2Cl_2 at 22 ºC for 2 h and $NaBF_4$ (164.69 mg, 1.5 mmol, 2.5 equiv.) at 70 ºC, for 4 h. The crude product was purified by pentane wash and the desired product was obtained as an off white solid (170.8 mg, 94%); **Rf:** 0.44 (hexane/EtOAc, 70:30); **Mp:** 294-296 ºC; **^{1}H NMR (600 MHz, DMSO–d_6) δ=** 11.74 (bs, 1H), 7.70 (d, $J = 1.9$ Hz, 1H), 7.47 (dd, $J = 7.9$, 1.8 Hz, 1H), 7.39 (d, $J = 7.9$ Hz, 1H), 1.37 (s, 9H); **$^{13}C\{^{1}H\}$ NMR (151 MHz, DMSO–d_6) δ=** 178.9, 137.8, 132.7, 129.7, 120.7, 119.8, 38.6, 26.5; **^{19}F NMR= (659 MHz, DMSO–d_6) δ=** -129.16, -129.23; **^{11}B NMR (193 MHz, DMSO–d_6) δ=** 2.86; **HRMS (ESI) (m/z):** calculated for $[M\text{-}H]^-$ $C_{11}H_{12}BBrF_2NO^-$ 302.0168; found 302.0173.

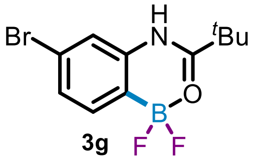

3g

***N*-(2-(difluoroboraneyl)-6-methylphenyl)pivalamide (3h):**

Following the general procedure A using *N*-(*o*-tolyl)pivalamide (114.75 mg, 0.6 mmol, 1 equiv.), BBr_3 (720 µL, 0.72 mmol, 1.2 equiv. 1M in CH_2Cl_2), in 2 mL CH_2Cl_2 at 22 ºC for 2 h and $NaBF_4$ (164.69 mg, 1.5 mmol, 2.5 equiv.) at 70 ºC, for 4 h. The crude product was purified by pentane wash and the desired product was obtained as an off white solid (132.2 mg, 92%); **Mp:** 191-193 ºC; **^{1}H NMR (600 MHz, DMSO–d$_6$) δ=** 9.86 (bs, 1H), 7.29 (dd, J = 6.4, 2.4 Hz, 1H), 7.23 – 7.19 (m, 2H), 2.44 (s, 3H), 1.41 (s, 9H); **^{13}C{^{1}H} NMR (151 MHz, DMSO–d$_6$) δ=** 178.5, 133.9, 129.9, 128.2, 127.1, 125.1, 38.7, 25.9, 16.4; **^{19}F NMR= (659 MHz, DMSO–d$_6$) δ= -**131.21, -131-28; **^{11}B NMR (193 MHz, DMSO–d$_6$) δ=** 3.13; **HRMS (ESI) (m/z):** calculated for $[M\text{-}H]^-$ $C_{12}H_{15}BF_2NO^-$ 238.1220; found 238.122.

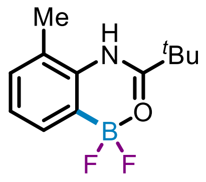

3h

***N*-(2-(difluoroboraneyl)-6-fluorophenyl)pivalamide (3i):**

Following the general procedure A using *N*-(2-fluorophenyl)pivalamide (117.14 mg, 0.6 mmol, 1 equiv.), BBr_3 (720 µL, 0.72 mmol, 1.2 equiv. 1M in CH_2Cl_2), in 2 mL CH_2Cl_2 at 22 ºC for 2 h and $NaBF_4$ (164.69 mg, 1.5 mmol, 2.5 equiv.) at 70 ºC, for 4 h. The crude product was purified by pentane wash and the desired product was obtained as an off white solid (96.2 mg, 66%); **Mp:** 258-260 ºC; **^{1}H NMR (600 MHz, DMSO–d$_6$) δ=** 11.43 (bs, 1H), 7.35 – 7.31 (m, 1H), 7.30 – 7.27 (m, 2H), 1.40 (s, 9H); **^{13}C{^{1}H} NMR (151 MHz, DMSO–d$_6$) δ=** 179.7, 150.3 (d, J = 250.8 Hz), 128.4 (d, J = 6.6 Hz), 126.0, 123.8 (d, J = 8.7 Hz), 114.7 (d, J = 17.8 Hz), 38.9, 26.2; **^{19}F NMR= (659 MHz, DMSO–d$_6$) δ=** -128.04 (F), -130.76 (BF_2), -130.83 (BF_2); **^{11}B NMR (193 MHz, DMSO–d$_6$) δ=** 2.62; **HRMS (ESI) (m/z):** calculated for $[M\text{-}H]^-$ $C_{11}H_{12}BF_3NO^-$ 242.0969; found 242.0969.

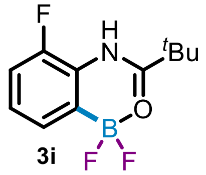

3i

***N*-(2-(difluoroboraneyl)-3,5-dimethylphenyl)pivalamide (3j):**

Following the general procedure A using *N*-(3,5-dimethylphenyl)pivalamide (123.17 mg, 0.6 mmol, 1 equiv.), BBr_3 (720 µL, 0.72 mmol, 1.2 equiv. 1M in CH_2Cl_2), in 2 mL CH_2Cl_2 at 22 ºC for 2 h and $NaBF_4$ (164.69 mg, 1.5 mmol, 2.5 equiv.) at 70 ºC, for 4 h. The crude product was purified by pentane wash and the desired product was obtained as an off white solid (114.1 mg, 75%); **Rf:** 0.29 (hexane/EtOAc, 70:30); **Mp:** 205-207 ºC; **^{1}H NMR (600 MHz, DMSO–d$_6$) δ=** 11.46 (bs, 1H), 7.08 (s, 1H), 6.90 (s, 1H), 2.32 (s, 3H), 2.27 (s, 3H), 1.36 (s, 9H); **^{13}C{^{1}H} NMR (151 MHz, DMSO–d$_6$) δ=** 177.3, 141.2, 137.1, 136.6, 129.1, 114.8, 38.3, 26.6, 20.8, 20.6; **^{19}F NMR= (659 MHz, DMSO–d$_6$) δ=** -126.96, -127.03; **^{11}B NMR (193 MHz, DMSO–d$_6$) δ=** 3.21; **HRMS (ESI) (m/z):** calculated for $[M\text{-}H]^-$ $C_{13}H_{17}BF_2NO^-$ 252.1376; found 252.1375.

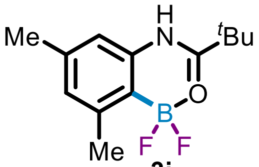

3j

N-(3,5-dichloro-2-(difluoroboraneyl)phenyl)pivalamide (3k):

Following the general procedure A using *N*-(3,5-dichlorophenyl)pivalamide (147.68 mg, 0.6 mmol, 1 equiv.), BBr_3 (720 µL, 0.72 mmol, 1.2 equiv. 1M in CH_2Cl_2), in 2 mL CH_2Cl_2 at 40 °C for 24 h and $NaBF_4$ (164.69 mg, 1.5 mmol, 2.5 equiv.) at 70 °C, for 4 h. The crude product was purified by pentane wash and the desired product was obtained as an off white solid (153.43 mg, 87%); **Rf:** 0.25 (hexane/EtOAc, 70:30); **Mp:** 285-287 °C; **^{1}H NMR (600 MHz, DMSO–d$_6$) δ=** 11.85 (bs, 1H), 7.55 (d, *J* = 1.8 Hz, 1H), 7.46 (d, *J* = 1.8 Hz, 1H), 1.36 (s, 9H); **^{13}C{^{1}H} NMR (151 MHz, DMSO–d$_6$) δ=** 179.1, 138.5, 138.0, 133.1, 127.1, 116.3, 38.7, 26.4; **^{19}F NMR= (659 MHz, DMSO–d$_6$) δ=** -128.35, -128.42; **^{11}B NMR (193 MHz, DMSO–d$_6$) δ=** 2.06; **HRMS (ESI) (m/z):** calculated for $[M\text{-}H]^-$ $C_{11}H_{11}BCl_2F_2NO^-$ 292.0284; found 292.0287.

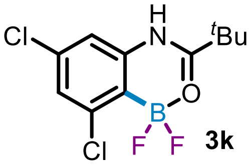

3k

N-(2-(difluoroboraneyl)naphthalen-1-yl)pivalamide (3l):

Following the general procedure A using *N*-(naphthalen-1-yl)pivalamide (136.38 mg, 0.6 mmol, 1 equiv.), BBr_3 (720 µL, 0.72 mmol, 1.2 equiv. 1M in CH_2Cl_2), in 2 mL CH_2Cl_2 at 22 °C for 2 h and $NaBF_4$ (164.69 mg, 1.5 mmol, 2.5 equiv.) at 70 °C, for 4 h. The crude product was purified by pentane wash and the desired product was obtained as an off white solid (156.81 mg, 95%); **Rf:** 0.32 (hexane/EtOAc, 50:50); **Mp:** 194-196 °C; **^{1}H NMR (600 MHz, DMSO–d$_6$) δ=** 11.14 (bs, 1H), 8.34 (dd, *J* = 8.5, 1.1 Hz, 1H), 7.99 (dd, *J* = 8.2, 1.3 Hz, 1H), 7.88 (d, *J* = 8.0 Hz, 1H), 7.69 (ddd, *J* = 8.4, 6.8, 1.4 Hz, 1H), 7.61 (ddd, *J* = 8.0, 6.8, 1.1 Hz, 1H), 7.58 (d, *J* = 8.1 Hz, 1H), 1.50 (s, 9H); **^{13}C{^{1}H} NMR (151 MHz, DMSO–d$_6$) δ=** 179.4, 133.2, 130.6, 128.4, 127.3, 127.1, 126.3 (2C), 122.2, 121.1, 39.0, 26.2; **^{19}F NMR= (659 MHz, DMSO–d$_6$) δ=** -129.92, -130.0; **^{11}B NMR (193 MHz, DMSO–d$_6$) δ=** 3.30; **HRMS (ESI) (m/z):** calculated for $[M\text{-}H]^-$ $C_{15}H_{15}BF_2NO^-$ 274.1220; found 274.1224.

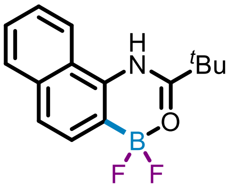

3l

(3*r*,5*r*,7*r*)-*N*-(2-(difluoroboraneyl)phenyl)adamantane-1-carboxamide (3m):

Following the general procedure A using (3*r*,5*r*,7*r*)-*N*-phenyladamantane-1-carboxamide (153.21 mg, 0.6 mmol, 1 equiv.), BBr_3 (720 µL, 0.72 mmol, 1.2 equiv. 1M in CH_2Cl_2), in 2 mL CH_2Cl_2 at 40 °C for 16 h and $NaBF_4$ (164.69 mg, 1.5 mmol, 2.5 equiv.) at 70 °C, for 4 h. The crude product was purified by pentane wash and the desired product was obtained as an off white solid (144.4 mg, 79%); **Rf:** 0.48 (hexane/EtOAc, 70:30); **Mp:** 323-325 °C; **^{1}H NMR (600 MHz, DMSO–d$_6$) δ=** 11.61 (bs, 1H), 7.47 – 7.42 (m, 2H), 7.36 (td, *J* = 7.7, 1.7 Hz, 1H), 7.27 (td, *J* = 7.3, 1.1 Hz, 1H), 2.12 – 2.07 (m, 3H), 2.03 (d, *J* = 3.0 Hz, 6H), 1.75 (t, *J* = 2.5 Hz, 6H); **^{13}C{^{1}H} NMR (151 MHz, DMSO–d$_6$) δ=** 177.0, 136.4, 130.6, 128.2, 126.9, 116.9, 40.1, 37.5, 35.5, 27.1; **^{19}F NMR= (659 MHz, DMSO–d$_6$) δ=** -128.99, -129.06; **^{11}B NMR (193 MHz, DMSO–d$_6$) δ=** 2.98; **HRMS (ESI) (m/z):** calculated for $[M\text{-}H]^-$ $C_{17}H_{19}BF_2NO^-$ 302.1533; found 302.1536.

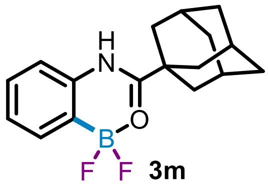

3m

_N_-(2-(difluoroboraneyl)phenyl)acetamide (3n):

Following the general procedure A using _N_-phenylacetamide (81.09 mg, 0.6 mmol, 1 equiv.), BBr_3 (1.8 mL, 1.8 mmol, 3 equiv. 1M in CH_2Cl_2), in 2 mL CH_2Cl_2 at 60 °C for 24 h and $NaBF_4$ (164.69 mg, 1.5 mmol, 2.5 equiv.) at 70 °C, for 4 h. The crude product was purified by 10% Et_2O:pentane wash and the desired product was obtained as beige color solid (26.5 mg, 24%); **Rf:** 0.12 (hexane/EtOAc, 70:30); **Mp:** 240-242 °C; **^{1}H NMR (600 MHz, DMSO–d$_6$) δ=** 12.73 (bs, 1H), 7.44 (d, _J_ = 7.1 Hz, 1H), 7.35 (td, _J_ = 7.7, 1.6 Hz, 1H), 7.26 (td, _J_ = 7.3, 1.1 Hz, 1H), 7.11 (d, _J_ = 8.0 Hz, 1H), 2.43 (s, 3H); **^{13}C{^{1}H} NMR (151 MHz, DMSO–d$_6$) δ=** 170.1, 136.3, 130.9, 128.3, 126.8, 116.0, 21.1; **^{19}F NMR= (659 MHz, DMSO–d$_6$) δ=** -128.29, -128.37; **^{11}B NMR (193 MHz, DMSO–d$_6$) δ=** 2.89; **HRMS (ESI) (m/z):** calculated for $[M\text{-}H]^-$ $C_8H_7BF_2NO^-$ 182.0594; found 182.0587.

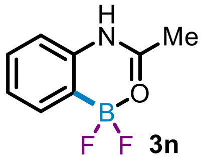

3n

1-(4-Chlorophenyl)-_N_-(2-(difluoroboraneyl)phenyl)cyclopentane-1-carboxamide (3o):

Following the general procedure A using 1-(4-chlorophenyl)-_N_-phenylcyclopentane-1-carboxamide (179.87 mg, 0.6 mmol, 1 equiv.), BBr_3 (720 µL, 0.72 mmol, 1.2 equiv. 1M in CH_2Cl_2), in 2 mL CH_2Cl_2 at 40 °C for 16 h and $NaBF_4$ (164.69 mg, 1.5 mmol, 2.5 equiv.) at 70 °C, for 4 h. The crude product was purified by pentane wash and the desired product was obtained as an off white solid (112.6 mg, 54%); **Rf:** 0.38 (hexane/EtOAc, 70:30); **Mp:** 252-254 °C; **^{1}H NMR (600 MHz, DMSO–d$_6$) δ=** 12.06 (bs, 1H), 7.52 – 7.41 (m, 5H), 7.34 (d, _J_ = 4.0 Hz, 2H), 7.27 (dt, _J_ = 8.1, 4.2 Hz, 1H), 2.71 – 2.59 (m, 2H), 2.23 – 2.18 (m, 2H), 1.72 (p, _J_ = 6.2 Hz, 4H; **^{13}C{^{1}H} NMR (151 MHz, DMSO–d$_6$) δ=** 174.9, 140.1, 136.5, 132.4, 130.6, 128.8, 128.5, 128.3, 127.2, 116.9, 58.0, 35.5, 22.5; **^{19}F NMR= (659 MHz, DMSO–d$_6$) δ=** -128.90, -128.97; **^{11}B NMR (193 MHz, DMSO–d$_6$) δ=** 3.20; **HRMS (ESI) (m/z):** calculated for $[M\text{-}H]^-$ $C_{18}H_{16}BClF_2NO^-$ 346.0987; found 346.0992.

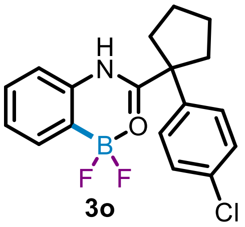

3o

1-(8-(Difluoroboraneyl)-3,4-dihydroquinolin-1(2_H_)-yl)-2,2-dimethylpropan-1-one (3p):

Following the general procedure A using 1-(3,4-Dihydroquinolin-1(2_H_)-yl)-2,2-dimethylpropan-1-one (130.38 mg, 0.6 mmol, 1 equiv.), BBr_3 (720 µL, 0.72 mmol, 1.2 equiv. 1M in CH_2Cl_2), in 2 mL CH_2Cl_2 at 22 °C for 1 h and $NaBF_4$ (164.69 mg, 1.5 mmol, 2.5 equiv.) at 70 °C, for 4 h. The crude product was purified by pentane wash and the desired product was obtained as brown solid (75 mg, 47%); **Rf:** 0.25 (hexane/EtOAc, 70:30); **Mp:** 184-186 °C; **^{1}H NMR (600 MHz, DMSO–d$_6$) δ=** 7.28 (dd, _J_ = 7.0, 2.0 Hz, 1H), 7.25 (t, _J_ = 7.2 Hz, 1H), 7.20 (dd, _J_ = 7.4, 1.8 Hz, 1H), 4.25 – 4.20 (m, 2H), 2.91 (t, _J_ = 6.5 Hz, 2H), 2.10 – 2.02 (m, 2H), 1.46 (s, 9H); **^{13}C{^{1}H} NMR (151 MHz, DMSO–d$_6$) δ=** 176.3, 137.0, 129.3, 127.8, 127.2, 127.1, 48.0, 40.2, 27.6,

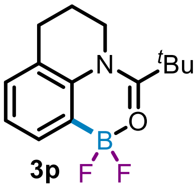

3p

26.5, 21.2; **^{19}F NMR= (564 MHz, DMSO–d$_6$) δ=** -135.10 (BF_2); **^{11}B NMR (193 MHz, DMSO–d$_6$) δ=** 2.30; **HRMS (ESI) (m/z):** calculated for $[M-H]^-$ $C_{14}H_{17}BF_2NO^-$ 264.1376; found 264.1359.

1-(7-(Difluoroboraneyl)indolin-1-yl)-2,2-dimethylpropan-1-one (3q):

Following the general procedure A using 1-(Indolin-1-yl)-2,2-dimethylpropan-1-one (121.97 mg, 0.6 mmol, 1 equiv.), BBr_3 (720 µL, 0.72 mmol, 1.2 equiv. 1M in CH_2Cl_2), in 2 mL CH_2Cl_2 at 22 °C for 1 h and $NaBF_4$ (164.69 mg, 1.5 mmol, 2.5 equiv.) at 70 °C, for 4 h. The crude product was purified by pentane wash and the desired product was obtained as beige color solid (98 mg, 65%); **Rf:** 0.28 (hexane/EtOAc, 70:30); **^{1}H NMR (600 MHz, DMSO–d$_6$) δ=** 7.35 – 7.25 (m, 3H, merged), 4.62 (dd, J = 8.4, 7.2 Hz, 2H), 3.31 (dd, J = 7.5 Hz, 2H, merged with water peak), 1.42 (s, 9H); **^{13}C{^{1}H} NMR (151 MHz, DMSO–d$_6$) δ=** 173.5, 142.4, 129.5, 128.1, 127.9, 124.1, 50.1, 39.2 (merged in DMSO peak), 28.2, 26.7; **^{19}F NMR= (564 MHz, DMSO–d$_6$) δ=** -128.05 (BF_2); **^{11}B NMR (193 MHz, DMSO–d$_6$) δ=** 3.03; **HRMS (ESI) (m/z):** calculated for $[MNa]^+$ $C_{13}H_{16}BF_2NNaO^+$ 274.1185; found 274.1982.

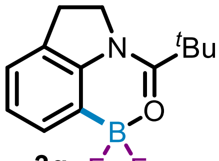

3q

1-(2-(Difluoroboraneyl)phenyl)pyrrolidin-2-one (3r):

Following the general procedure A using 1-phenylpyrrolidin-2-one (96.72 mg, 0.6 mmol, 1 equiv.), BBr_3 (1.8 mL, 1.8 mmol, 3 equiv. 1M in CH_2Cl_2), in 2 mL CH_2Cl_2 at 60 °C for 24 h and $NaBF_4$ (164.69 mg, 1.5 mmol, 2.5 equiv.) at 70 °C, for 4 h. The crude product was purified by pentane wash and the desired product was obtained as an off white solid (97.4 mg, 78%); **Rf:** 0.1 (hexane/EtOAc, 70:30); **Mp:** 263-265 °C; **^{1}H NMR (600 MHz, DMSO–d$_6$) δ=** 7.50 (d, J = 6.7 Hz, 1H), 7.43 (td, J = 7.8, 1.6 Hz, 1H), 7.34 (t, J = 7.3 Hz, 1H), 7.18 (d, J = 8.1 Hz, 1H), 4.21 (t, J = 7.4 Hz, 2H), 3.08 (t, J = 8.1 Hz, 2H), 2.24 (p, J = 7.9 Hz, 2H); **^{13}C{^{1}H} NMR (151 MHz, DMSO–d$_6$) δ=** 174.9, 136.7, 130.9, 128.4, 127.0, 115.1, 49.4, 31.9, 16.2; **^{19}F NMR= (659 MHz, DMSO–d$_6$) δ=** -127.83, -127.90; **^{11}B NMR (193 MHz, DMSO–d$_6$) δ=** 3.31; **HRMS (ESI) (m/z):** calculated for $[M+H]^+$ $C_{10}H_{11}BF_2NO^+$ 210.0896; found 210.0898.

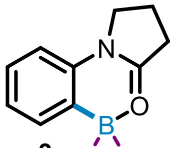

3r

1-(2-(Difluoroboraneyl)phenyl)-3-phenylurea (3s):

Following the general procedure A using 1,3-diphenylurea (127.34 mg, 0.6 mmol, 1 equiv.), BBr_3 (720 µL, 0.72 mmol, 1.2 equiv. 1M in CH_2Cl_2), in 2 mL CH_2Cl_2 at 60 °C for 24 h and $NaBF_4$ (164.69 mg, 1.5 mmol, 2.5 equiv.) at 70 °C, for 4 h. The crude product was purified by 30-50% Et_2O:Pentane and the desired product was obtained as an off white solid (87.7 mg, 56%); **Rf:** 0.1 (hexane/EtOAc, 70:30); **Mp:** 256-258 °C; **^{1}H NMR (600 MHz, DMSO–d$_6$) δ=** 10.64 (bs, 1H), 10.06 (bs, 1H), 7.48 – 7.43 (m, 2H), 7.41 – 7.37 (m, 3H), 7.28 – 7.25 (m, 2H), 7.10 (td, J = 7.3, 1.0 Hz, 1H), 7.04 (d, J = 7.6 Hz, 1H); **^{13}C{^{1}H} NMR (151 MHz, DMSO–d$_6$) δ=** 154.6, 137.8, 135.5, 130.6, 129.4, 128.2, 125.8, 124.3, 122.9, 115.2; **^{19}F NMR= (659 MHz,**

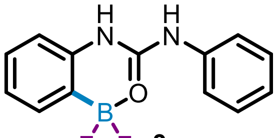

3s

DMSO–d$_6$) δ= -133.26, -134.34; **^{11}B NMR (193 MHz, DMSO–d$_6$) δ=** 3.46; **HRMS (ESI) (m/z):** calculated for [M-H]$^-$ $C_{13}H_{10}BF_2N_2O^-$ 259.0859; found 259.086.

***N,N′*-(2,6-bis(difluoroboraneyl)naphthalene-1,5-diyl)bis(2,2-dimethylpropanamide) (3t):**

Following the general procedure A using *N,N′*-(naphthalene-1,5-diyl)bis(2,2-dimethylpropanamide) (195.85 mg, 0.6 mmol, 1 equiv.), BBr_3 (1.8 mL, 1.8 mmol, 3 equiv. 1M in CH_2Cl_2), in 2 mL CH_2Cl_2 at 60 ºC for 65 h and $NaBF_4$ (329.38 mg, 3.0 mmol, 5 equiv.) at 70 ºC, for 16 h. The crude product was purified by pentane and 100% diethyl ether. The desired product was obtained as an off white solid (212.2 mg, 84%); **Mp:** 290-292 ºC; **^{1}H NMR (600 MHz, DMSO–d$_6$) δ=** 11.27 (bs, 2H), 8.26 (d, *J* = 8.4 Hz, 2H), 7.74 (d, *J* = 8.4 Hz, 2H), 1.50 (s, 18H); **^{13}C{^{1}H} NMR (151 MHz, DMSO–d$_6$) δ=** 179.8, 131.1, 127.5, 122.4, 120.3, 39.1, 26.2; **^{19}F NMR= (659 MHz, DMSO–d$_6$) δ=** -129.94, -130.01; **^{11}B NMR (193 MHz, DMSO–d$_6$) δ=** 3.41; **HRMS (ESI) (m/z):** calculated for [M-H]$^-$ $C_{20}H_{23}B_2F_4N_2O_2^-$ 421.1887; found 421.191

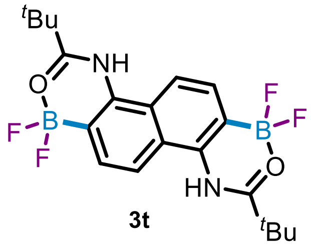

3t

***N,N′*-(2,5-bis(difluoroboraneyl)-1,4-phenylene)bis(2,2-dimethylpropanamide) (3u):**

Following the general procedure A using *N,N′*-(1,4-phenylene)bis(2,2-dimethylpropanamide) (165.82 mg, 0.6 mmol, 1 equiv.), BBr_3 (1.8 mL, 1.8 mmol, 3 equiv. 1M in CH_2Cl_2), in 2 mL CH_2Cl_2 at 60 ºC for 65 h and $NaBF_4$ (329.38 mg, 3.0 mmol, 5 equiv.) at 70 ºC, for 16 h. The crude product was purified by 50% Et_2O:pentane and the desired product was obtained as an off white solid (136.5 mg, 61%); **Mp:** >350 ºC; **^{1}H NMR (600 MHz, DMSO–d$_6$) δ=** 11.77 (bs, 2H), 7.56 (s, 2H), 1.37 (s, 18H); **^{13}C{^{1}H} NMR (151 MHz, DMSO–d$_6$) δ=** 177.8, 135.1, 119.0, 38.5, 26.6; **^{19}F NMR= (659 MHz, DMSO–d$_6$) δ=** -130.00, -130.07; **^{11}B NMR (193 MHz, DMSO–d$_6$) δ=** 3.07; **HRMS (ESI) (m/z):** calculated for [M-H]$^-$ $C_{16}H_{21}B_2F_4N_2O_2^-$ 371.1730; found 371.1735.

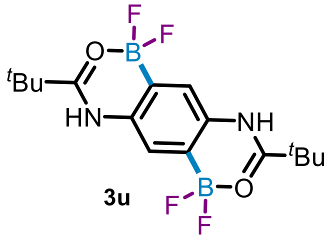

3u

***N,N′*-(oxybis(2-(difluoroboraneyl)-4,1-phenylene))bis(2,2-dimethylpropanamide) (3v):**

Following the general procedure A using *N,N′*-(oxybis(4,1-phenylene))bis(2,2-dimethylpropanamide) (221.07 mg, 0.6 mmol, 1 equiv.), BBr_3 (1.8 mL, 1.8 mmol, 3 equiv. 1M in CH_2Cl_2), in 2.5 mL CH_2Cl_2 at 22 ºC for 2 h and $NaBF_4$ (329.36 mg, 3.0 mmol, 5 equiv.). The crude product was purified by pentane wash and the desired product was obtained as an off white solid (189.34 mg, 68%); **Rf:** 0.23 (hexane/EtOAc, 50:50); **Mp:** 341-343 ºC; **^{1}H NMR (600 MHz, DMSO–d$_6$) δ=** 11.84 (bs, 2H), 7.51 (d, *J* = 8.8 Hz, 2H), 7.06 (dd, *J* = 8.7, 2.8 Hz, 2H), 6.90 (d, *J* = 2.8 Hz, 2H), 1.36 (s, 18H); **^{13}C{^{1}H} NMR (151 MHz, DMSO–d$_6$) δ=** 177.3, 155.6, 132.2, 119.4, 119.3, 119.1, 38.4, 26.6; **^{19}F NMR= (659 MHz, DMSO–d$_6$) δ=** -129.75, -129.82;

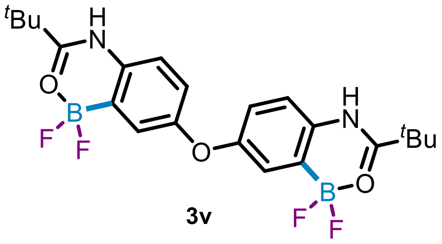

3v

^{11}B NMR (193 MHz, DMSO–d_6) δ= 2.83; **HRMS (ESI) (m/z):** calculated for $[M-H]^-$ $C_{22}H_{25}B_2F_4N_2O_3^-$ 463.1992; found 463.2003.

3.4 General Procedure B for reaction optimization: (3w-3ah)

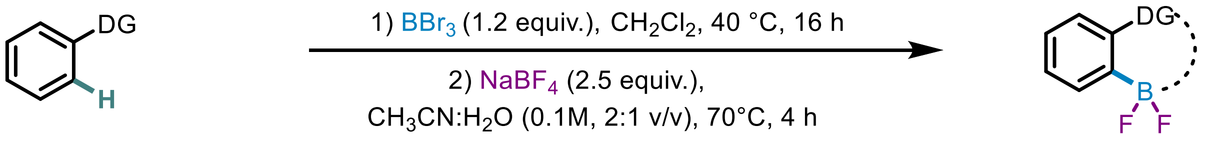

1w-1ah **3w-3ah**

Step i) To a dry 25 mL microwave vial, equipped with a rubber septum, stir bar, the amide derivative (0.6 mmol, 1 equiv.) in anhydrous CH_2Cl_2 (2 mL) under a nitrogen atmosphere was added dropwise BBr_3 (720 µl, 0.72 mmol, 1.2 equiv., 1M solution in CH_2Cl_2). After the complete addition of BBr_3, the reaction mixture was stirred at 40 ºC for 16 h after which the solvent was removed under reduced pressure.

Step ii) To the crude residue from step i) were added $NaBF_4$ (164.69 mg, 1.5 mmol, 2.5 equiv.), 4 mL acetonitrile, 2 mL distilled water and the reaction mixture was heated at 70 ºC for 4 h. The reaction was allowed to reach room temperature and the acetonitrile was evaporated under *vacuo* to afford the crude solid. After removal of complete acetonitrile, the crude solid was taken in 20 mL distilled water and filtered. Additional 30 mL water wash was given to remove inorganic impurities. The crude solid was washed with pentane or mixture of Et_2O:Pentane to remove unreacted starting material.

Note: The crude product can also be purified by stirring the mixture in pentane or mixture of Et_2O:Pentane.

3.5 Spectral data

***N*-(2-(difluoroboraneyl)phenyl)benzamide (3w):**

Following the general procedure B using *N*-phenylbenzamide (118.33 mg, 0.6 mmol, 1 equiv.), BBr_3 (720 µL, 0.72 mmol, 1.2 equiv. 1M in CH_2Cl_2), in 2 mL CH_2Cl_2 at 40 ºC for 16 h and $NaBF_4$ (164.69 mg, 1.5 mmol, 2.5 equiv.) at 70 ºC, for 4 h. The crude product was purified by 15% Et_2O:Pentane wash and the desired product was obtained as an off white solid (110.8 mg, 75%); **Rf:** 0.22 (hexane/EtOAc, 70:30); **Mp:** 271-273 ºC; **^{1}H NMR (600 MHz, DMSO–d$_6$) δ=** 12.93 (bs, 1H), 8.19 (d, *J* = 7.4 Hz, 2H), 7.81 (t, *J* = 7.4 Hz, 1H), 7.70 (t, *J* = 7.7 Hz, 2H), 7.52 (d, *J* = 7.2 Hz, 1H), 7.47 (d, *J* = 8.0 Hz, 1H), 7.43 (t, *J* = 7.6 Hz, 1H), 7.34 (t, *J* = 7.2 Hz, 1H); **^{13}C{^{1}H} NMR (151 MHz, DMSO–d$_6$) δ=** 164.8, 136.7, 134.6, 130.8, 129.2, 128.8, 128.4, 128.3, 127.3, 117.4; **^{19}F NMR= (659 MHz, DMSO–d$_6$) δ=** -128.44, -128.52; **^{11}B NMR (193 MHz, DMSO–d$_6$) δ=** 3.28; **HRMS (ESI) (m/z):** calculated for $[M\text{-}H]^-$ $C_{13}H_9BF_2NO^-$ 244.075; found 244.075.

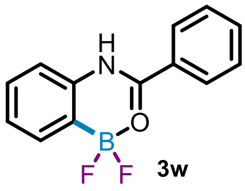

3w

***N*-(2-(difluoroboraneyl)phenyl)-3-methylbenzamide (3x):**

Following the general procedure B using 3-methyl-*N*-phenylbenzamide (126.75 mg, 0.6 mmol, 1 equiv.), BBr_3 (720 µL, 0.72 mmol, 1.2 equiv. 1M in CH_2Cl_2), in 2 mL CH_2Cl_2 at 40 ºC for 16 h and $NaBF_4$ (164.69 mg, 1.5 mmol, 2.5 equiv.) at 70 ºC, for 4 h. The crude product was purified by 15% Et_2O:Pentane wash and the desired product was obtained as an off white solid (106.1 mg, 68%); **Rf:** 0.33 (hexane/EtOAc,

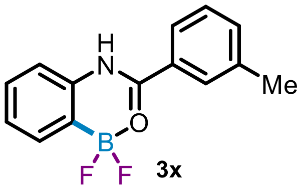

3x

70:30); **^{1}H NMR (600 MHz, DMSO–d$_6$) δ=** 12.87 (bs, 1H), 8.0 – 7.98 (m, 2H, singlet merged with doublet), 7.63 (d, *J* = 7.6 Hz, 1H), 7.59 (t, *J* = 7.6 Hz, 1H), 7.51 (dd, *J* = 7.3, 1.5 Hz, 1H), 7.47 (d, *J* = 8.0 Hz, 1H), 7.42 (td, *J* = 7.6, 1.6 Hz, 1H), 7.33 (td, *J* = 7.2, 1.2 Hz, 1H), 2.46 (s, 3H); **^{13}C{^{1}H} NMR (151 MHz, DMSO–d$_6$) δ=** 164.9, 138.8, 136.7, 135.2, 130.7, 129.1, 128.9, 128.4, 128.2, 127.2, 125.9, 117.4, 20.8; **^{19}F NMR= (659 MHz, DMSO–d$_6$) δ=** -128.52, -128.60; **^{11}B NMR (193 MHz, DMSO–d$_6$) δ=** 3.25; **HRMS (ESI) (m/z):** calculated for $[M\text{-}H]^-$ $C_{14}H_{11}BF_2NO^-$ 258.0907; found 258.0908.

***N*-(2-(difluoroboraneyl)phenyl)-3-nitrobenzamide (3y):**

Following the general procedure B using 3-nitro-*N*-phenylbenzamide (145.33 mg, 0.6 mmol, 1 equiv.). BBr_3 (720 µL, 0.72 mmol, 1.2 equiv. 1M in CH_2Cl_2), in 2 mL CH_2Cl_2 at 40 ºC for 16 h and $NaBF_4$ (164.69 mg, 1.5 mmol, 2.5 equiv.) at 70 ºC, for 4 h. The crude product was purified by 40% Et_2O:Pentane wash and the desired product was obtained as an off white solid (101.2 mg, 58%); **Mp:** 295-297 ºC; **^{1}H NMR (600 MHz, DMSO–d$_6$) δ=** 13.30 (bs, 1H), 9.03 (t, *J* = 2.0 Hz, 1H), 8.61 (dd, *J* = 8.3, 2.4 Hz, 1H), 8.59 (dt, *J* = 7.8, 1.4 Hz, 1H), 7.99 (t, *J* = 8.0 Hz, 1H), 7.54 (d, *J* = 7.2 Hz, 1H), 7.49 (d, *J* = 8.0 Hz, 1H), 7.47 – 7.43 (m, 1H), 7.37 (td, *J* = 7.2, 1.4 Hz, 1H); **^{13}C{^{1}H} NMR (151 MHz, DMSO–d$_6$) δ=** 162.8, 147.9, 136.6, 135.0, 130.9, 130.8, 130.0, 128.6, 128.6, 127.7, 123.6, 117.7; **^{19}F NMR= (659 MHz, DMSO–d$_6$) δ=** -127.70, -127.78; **^{11}B NMR (193 MHz, DMSO–d$_6$) δ=** 3.40; **HRMS (ESI) (m/z):** calculated for $[M\text{-}H]^-$ $C_{13}H_8BF_2N_2O_3^-$ 289.0601; found 289.0605.

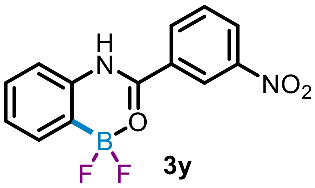

***N*-(5-chloro-2-(difluoroboraneyl)phenyl)-4-methylbenzamide (3z):**

Following the general procedure B using *N*-(3-chlorophenyl)-4-methylbenzamide (147.42 mg, 0.6 mmol, 1 equiv.), BBr_3 (720 µL, 0.72 mmol, 1.2 equiv. 1M in CH_2Cl_2), in 2 mL CH_2Cl_2 at 40 ºC for 16 h and $NaBF_4$ (164.69 mg, 1.5 mmol, 2.5 equiv.) at 70 ºC, for 4 h. The crude product was purified by 20% Et_2O:Pentane wash and the desired product was obtained as an off white solid (115 mg, 65%); **Rf:** 0.4 (hexane/EtOAc, 70:30); **Mp:** 297-299 ºC; **^{1}H NMR (600 MHz, DMSO–d$_6$) δ=** 12.80 (bs, 1H), 8.10 (d, *J* = 7.9 Hz, 2H), 7.52 (d, *J* = 6.8 Hz, 4H), 7.37 (d, *J* = 7.9 Hz, 1H), 2.46 (s, 3H); **^{13}C{^{1}H} NMR (151 MHz, DMSO–d$_6$) δ=** 165.3, 145.8, 138.0, 132.7, 132.5, 129.8, 128.9, 126.9, 125.1, 117.0, 21.3; **^{19}F NMR= (659 MHz, DMSO–d$_6$) δ=** -128.82, -128.90; **^{11}B NMR (193 MHz, DMSO–d$_6$) δ=** 3.08; **HRMS (ESI) (m/z):** calculated for $[M\text{-}H]^-$ $C_{14}H_{10}BClF_2NO^-$ 292.0512; found 292.0522.

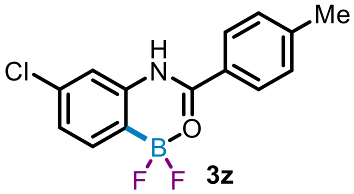

N-(2-(difluoroboraneyl)phenyl)-4-(methylsulfonyl)benzamide (3aa):

Following the general procedure B using 4-(methylsulfonyl)-*N*-phenylbenzamidede (165.19 mg, 0.6 mmol, 1 equiv.), BBr_3 (720 µL, 0.72 mmol, 1.2 equiv. 1M in CH_2Cl_2), in 2 mL CH_2Cl_2 at 40 ºC for 16 h

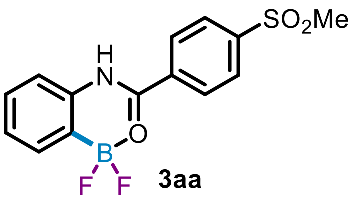

3aa

and $NaBF_4$ (164.69 mg, 1.5 mmol, 2.5 equiv.) at 70 ºC, for 4 h. The crude product was purified by 40% Et_2O:Pentane wash and the desired product was obtained as an off white solid (146.5 mg, 76%); **Rf:** 0.16 (hexane/EtOAc, 70:30); **Mp:** 287-289 ºC; **^{1}H NMR (600 MHz, DMSO–d$_6$) δ=** 13.24 (bs, 1H), 8.41 (d, *J* = 8.5 Hz, 2H), 8.24 (d, *J* = 8.4 Hz, 2H), 7.54 (d, *J* = 7.3 Hz, 1H), 7.48 (d, *J* = 8.0 Hz, 1H), 7.47 – 7.43 (m, 1H), 7.37 (td, *J* = 7.2, 1.4 Hz, 1H), 3.36 (s, 3H, methyl is merged with water peak); **^{13}C{^{1}H} NMR (151 MHz, DMSO–d$_6$) δ=** 163.5, 145.3, 136.6, 132.9, 130.8, 129.9, 128.6, 127.7, 127.6, 117.7, 43.1; **^{19}F NMR= (659 MHz, DMSO–d$_6$) δ=** -127.62, -127.70; **^{11}B NMR (193 MHz, DMSO–d$_6$) δ=** 3.25; **HRMS (ESI) (m/z):** calculated for $[M-H]^-$ $C_{14}H_{11}BF_2NO_3S^-$ 322.0526; found 322.0531.

2-Chloro-N-(2-(difluoroboraneyl)phenyl)-4-(methylsulfonyl)benzamide (3ab):

Following the general procedure B using 2-chloro-4-(methylsulfonyl)-*N*-phenylbenzamide (185.86 mg, 0.6 mmol, 1 equiv.), BBr_3 (1.2 mL, 1.2 mmol, 2 equiv. 1M in CH_2Cl_2), in 2 mL CH_2Cl_2 at 60 ºC for 65 h

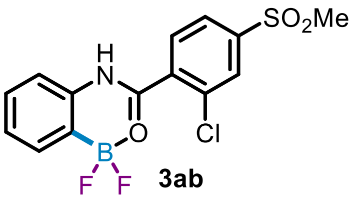

3ab

and $NaBF_4$ (164.69 mg, 1.5 mmol, 2.5 equiv.) at 70 ºC, for 4 h. The crude product was purified by 40% Et_2O:Pentane wash and the desired product was obtained as an off white solid (164.3 mg, 77%); **Rf:** 0.23 (hexane/EtOAc, 70:30); **Mp:** 261-263 ºC; **^{1}H NMR (600 MHz, DMSO–d$_6$) δ=** 13.72 (bs, 1H), 8.28 (d, *J* = 1.7 Hz, 1H), 8.22 (d, *J* = 8.1 Hz, 1H), 8.15 (dd, *J* = 8.0, 1.7 Hz, 1H), 7.56 (dd, *J* = 7.3, 1.6 Hz, 1H), 7.45 (td, *J* = 7.7, 1.7 Hz, 1H), 7.39 (td, *J* = 7.3, 1.2 Hz, 1H), 7.27 (d, *J* = 7.9 Hz, 1H), 3.39 (s, 3H); **^{13}C{^{1}H} NMR (151 MHz, DMSO–d$_6$) δ=** 163.7, 145.3, 136.2, 133.8, 132.4, 131.9, 131.0, 128.7, 128.6, 128.1, 126.1, 117.4, 42.9; **^{19}F NMR= (659 MHz, DMSO–d$_6$) δ=** -126.76, -126.84; **^{11}B NMR (193 MHz, DMSO–d$_6$) δ=** 3.44; **HRMS (ESI) (m/z):** calculated for $[M-H]^-$ $C_{14}H_{10}BClF_2NO_3S^-$ 356.0136; found 356.0141.

N-(2-(difluoroboraneyl)phenyl)-4-(N,N-dipropylsulfamoyl)benzamide (3ac):

Following the general procedure B using 4-(*N*,*N*-dipropylsulfamoyl)-*N*-phenylbenzamide (216.28 mg, 0.6 mmol, 1 equiv.), BBr_3 (720 µL, 0.72 mmol, 1.2 equiv. 1M in CH_2Cl_2), in 2 mL CH_2Cl_2 at 40 ºC for 16 h and $NaBF_4$ (164.69 mg, 1.5 mmol, 2.5 equiv.) at 70 ºC, for 4 h. The crude product was purified by 40% Et_2O:Pentane wash and the desired product was obtained as an off white solid (170.3 mg, 70%); **Rf:** 0.5 (hexane/EtOAc, 50:50); **Mp:**

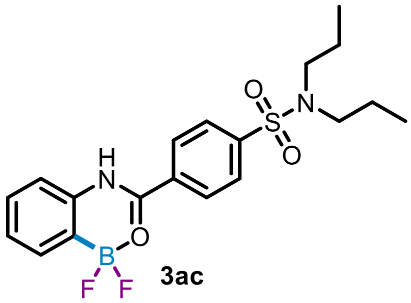

3ac

289-291 ºC; **^{1}H NMR (600 MHz, DMSO–d$_6$) δ=**13.20 (bs, 1H), 8.36 (d, *J* = 8.5 Hz, 2H), 8.11 (d, *J* = 8.4 Hz, 2H), 7.53 (dd, *J* = 7.3, 1.5 Hz, 1H), 7.48 (d, *J* = 8.0 Hz, 1H), 7.46 – 7.42 (m, 1H), 7.36 (td, *J* = 7.2, 1.3 Hz, 1H), 3.10 (t, *J* = 7.4 Hz, 4H), 1.49 (p, *J* = 7.4 Hz, 4H), 0.82 (t, *J* = 7.4 Hz, 6H); **^{13}C{^{1}H} NMR (151 MHz, DMSO–d$_6$) δ=** 163.6, 144.3, 136.6, 131.9, 130.8, 129.9, 128.5, 127.6, 127.3, 117.6, 49.6, 21.6, 10.9; **^{19}F NMR= (659 MHz, DMSO–d$_6$) δ=** -127.82, -127.89; **^{11}B NMR (193 MHz, DMSO–d$_6$) δ=** 3.24; **HRMS (ESI) (m/z):** calculated for $[M\text{-}H]^-$ $C_{19}H_{22}BF_2N_2O_3S^-$ 407.1417; found 407.1425.

5-Bromo-*N*-(2-(difluoroboraneyl)phenyl)furan-2-carboxamide (3ad):

Following the general procedure B using 5-bromo-*N*-phenylfuran-2-carboxamide (159.65 mg, 0.6 mmol, 1 equiv.), BBr_3 (720 µL, 0.72 mmol, 1.2 equiv. 1M in CH_2Cl_2), in 2 mL CH_2Cl_2 at 40 ºC for 16 h and $NaBF_4$ (164.69 mg, 1.5 mmol, 2.5 equiv.) at 70 ºC, for 4 h. The crude product was purified by 10% Et_2O:Pentane wash and the desired product was obtained as an off white solid (155 mg, 82%); **Rf:** 0.36 (hexane/EtOAc, 50:50); **Mp:** 270-272 ºC; **^{1}H NMR (600 MHz, DMSO–d$_6$) δ=** 13.10 (bs, 1H), 7.82 (d, *J* = 3.7 Hz, 1H), 7.47 (dd, *J* = 9.6, 7.8 Hz, 2H), 7.40 (td, *J* = 7.7, 1.6 Hz, 1H), 7.30 (td, *J* = 7.3, 1.1 Hz, 1H), 7.08 (d, *J* = 3.8 Hz, 1H); **^{13}C{^{1}H} NMR (151 MHz, DMSO–d$_6$) δ=** 153.9, 144.1, 136.4, 130.8, 130.3, 128.5, 127.2, 123.2, 117.3, 116.1; **^{19}F NMR= (659 MHz, DMSO–d$_6$) δ=** -128.76, -128.83; **^{11}B NMR (193 MHz, DMSO–d$_6$) δ=** 2.95; **HRMS (ESI) (m/z):** calculated for $[M\text{-}H]^-$ $C_{11}H_6BBrF_2NO_2^-$ 311.9648; found 311.9653.

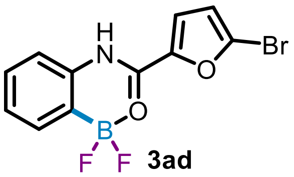

3ad

***N*-(*tert-butyl*)-8-(difluoroboraneyl)-1-naphthamide (3ae):**

Following the general procedure B using *N*-(*tert*-butyl)-1-naphthamide (136.38 mg, 0.6 mmol, 1 equiv.), BBr_3 (1.05 mL, 1.05 mmol, 1.75 equiv. 1M in CH_2Cl_2), in 3 mL CH_2Cl_2 at 60 ºC for 16 h and $NaBF_4$ (164.69 mg, 1.5 mmol, 2.5 equiv.) at 70 ºC, for 4 h. The crude product was purified by pentane wash and the desired product was obtained as an off white solid (127 mg, 77%); **Rf:** 0.22 (hexane/EtOAc, 70:30); **Mp:** 165-167 ºC; **^{1}H NMR (600 MHz, DMSO–d$_6$) δ=** 9.81 (bs, 1H), 8.67 (dd, *J* = 7.5, 1.1 Hz, 1H), 8.32 (dd, *J* = 8.3, 1.0 Hz, 1H), 7.96 (d, *J* = 7.6 Hz, 1H), 7.76 (d, *J* = 6.7 Hz, 1H), 7.68 (t, *J* = 7.8 Hz, 1H), 7.62 (dd, *J* = 8.2, 6.7 Hz, 1H), 1.58 (s, 9H); **^{13}C{^{1}H} NMR (151 MHz, DMSO–d$_6$) δ=** 168.7, 135.9, 132.1, 131.7, 130.5, 128.5, 127.1, 127.0, 124.3, 119.3, 55.3, 28.2; **^{19}F NMR= (659 MHz, DMSO–d$_6$) δ=** -135.80, -135.87; **^{11}B NMR (193 MHz, DMSO–d$_6$) δ=** 3.75; **HRMS (ESI) (m/z):** calculated for $[M\text{-}H]^-$ $C_{15}H_{15}BF_2NO^-$ 274.1220; found 274.1222.

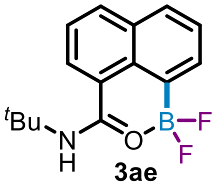

3ae

***N-(tert-butyl)*-10-(difluoroboraneyl)pyrene-1-carboxamide (3af):**

Following the general procedure B using *N*-(*tert*-butyl)pyrene-1-carboxamide (180.83 mg, 0.6 mmol, 1 equiv.), BBr_3 (1.05 mL, 1.05 mmol, 1.75 equiv. 1M in CH_2Cl_2) in 3 mL CH_2Cl_2 at 60 ºC for 16 h and

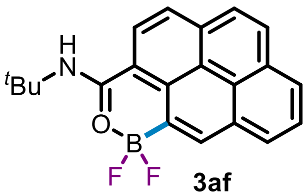

3af

$NaBF_4$ (164.69 mg, 1.5 mmol, 2.5 equiv.) at 70 ºC, for 4 h. The crude product was purified by 40% Et_2O:pentane wash and the desired product was obtained as yellow solid (180 mg, 86%); **Rf:** 0.62 (hexane/EtOAc, 50:50); **Mp:** 227-229 ºC; **^{1}H NMR (600 MHz, DMSO–d$_6$) δ=** 9.90 (bs, 1H), 9.06 (d, *J* = 8.4 Hz, 1H), 8.51 (s, 1H), 8.49 (d, *J* = 7.6 Hz, 1H), 8.46 – 8.40 (m, 3H), 8.30 (d, *J* = 8.9 Hz, 1H), 8.18 (t, *J* = 7.6 Hz, 1H), 1.65 (s, 9H); **^{13}C{^{1}H} NMR (151 MHz, DMSO–d$_6$) δ=** 168.7, 135.5, 133.3, 132.3, 131.1, 130.3, 130.1, 127.1, 126.9, 126.8 (2C), 125.9, 123.7, 122.5, 122.1, 115.2, 55.5, 28.3; **^{19}F NMR= (659 MHz, DMSO–d$_6$) δ=** -135.93, -136.00; **^{11}B NMR (193 MHz, DMSO–d$_6$) δ=** 3.74; **HRMS (ESI) (m/z):** calculated for $[M\text{-}H]^-$ $C_{21}H_{17}BF_2NO^-$ 348.1376; found 348.1380.

***N-(tert-butyl)*-2-(2-(difluoroboraneyl)-4-isobutylphenyl)propanamide (3ag):**

Following the general procedure B using *N*-(*tert*-butyl)-2-(4-isobutylphenyl)propanamide (156.84 mg, 0.6 mmol, 1 equiv.), BBr_3 (1.05 mL, 1.05 mmol, 1.75 equiv. 1M in CH_2Cl_2), in 3 mL CH_2Cl_2 at 60 ºC for

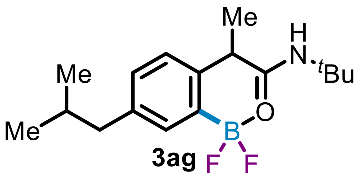

3ag

16 h and $NaBF_4$ (164.69 mg, 1.5 mmol, 2.5 equiv.) at 70 ºC, for 4 h. The crude product was purified by pentane wash and the desired product was obtained as beige color solid (145 mg, 78%); **Rf:** 0.64 (hexane/EtOAc, 50:50); **Mp:** 249-251 ºC; **^{1}H NMR (600 MHz, DMSO–d$_6$) δ=** 10.13 (bs, 1H), 7.16 (d, *J* = 1.8 Hz, 1H), 7.01 – 6.95 (m, 2H), 3.83 (q, *J* = 7.3 Hz, 1H), 2.39 (d, *J* = 7.1 Hz, 2H), 1.79 (hept, *J* = 6.7 Hz, 1H), 1.44 (d, *J* = 7.3 Hz, 3H), 1.38 (s, 9H), 0.85 (d, *J* = 6.6 Hz, 6H); **^{13}C{^{1}H} NMR (151 MHz, DMSO–d$_6$) δ=** 179.2 (179.2), 138.6, 137.3 (137.3), 130.5, 128.1, 125.2, 53.8, 44.6, 40.5, 29.8, 27.7, 24.6, 22.3 (22.2); **^{19}F NMR= (659 MHz, DMSO–d$_6$) δ=** (BF_1): -134.60, -134.67, (BF_{1*}): -134.75, -134.82; (BF_2): -146.81, -146.88, (BF_{2*}): -146.95, -147.03; **^{11}B NMR (193 MHz, DMSO–d$_6$) δ=** 4.37; **HRMS (ESI) (m/z):** calculated for $[M\text{-}H]^-$ $C_{17}H_{25}BF_2NO^-$ 308.2002; found 308.2006.

3.6 General Procedure C for reaction optimization: (3ah-3al)

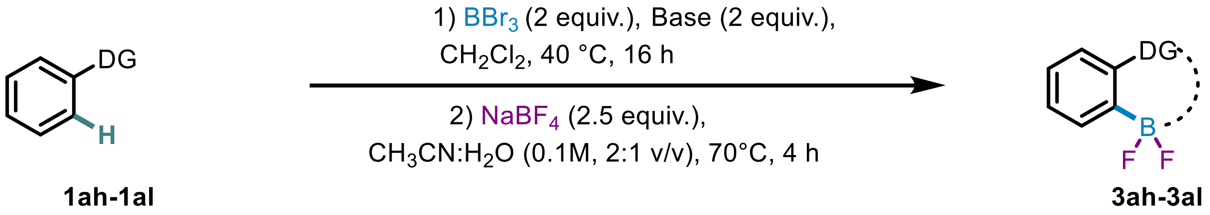

Step i) To a dry 25 mL microwave vial, equipped with a rubber septum, stir bar, the *N*-heteroarene derivative (0.6 mmol, 1 equiv.) in anhydrous CH_2Cl_2 (2 mL), 2,6-lutidine (139.8 µl, 1.2 mmol, 2 equiv.) under a nitrogen atmosphere at 0 ºC was added dropwise BBr_3 (1.2 mL, 1.2 mmol, 2 equiv., 1M solution

in CH_2Cl_2). After the complete addition of BBr_3, the reaction mixture was stirred at 40 ºC for 16 h after which the solvent was removed under reduced pressure.

Step ii) To the crude residue from step i) were added $NaBF_4$ (164.69 mg, 1.5 mmol, 2.5 equiv.), 4 mL acetonitrile, 2 mL distilled water and the reaction mixture was heated at 70 ºC for 4 h. The reaction was allowed to reach room temperature and the acetonitrile was evaporated under *vacuo* to afford the crude solid. After removal of complete acetonitrile, the crude solid was taken in 20 mL distilled water and filtered. Additional 30 mL water wash was given to remove inorganic impurities. The crude solid was stirred in pentane or 5% Et_2O:Pentane to remove unreacted starting material.

2-(2-(Difluoroboraneyl)phenyl)pyridine (3ah):

Following the general procedure C using 2-phenylpyridine (93.12 mg, 0.6 mmol, 1 equiv.), BBr_3 (1.2 mL, 1.2 mmol, 2 equiv. 1M in CH_2Cl_2), 2,6-lutidine (139.8 µl, 1.2 mmol, 2 equiv.) in 2 mL CH_2Cl_2 at 40 ºC

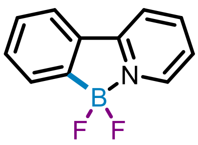

3ah

for 16 h and $NaBF_4$ (164.69 mg, 1.5 mmol, 2.5 equiv.) at 70 ºC, for 4 h. The crude product was purified by pentane wash and the desired product was obtained as beige color solid (115.71 mg, 95%); **^{1}H NMR (600 MHz, DMSO–d$_6$) δ=** 8.70 (d, *J* = 5.6 Hz, 1H), 8.41 (td, *J* = 7.8, 1.5 Hz, 1H), 8.37 – 8.33 (m, 1H), 8.03 (d, *J* = 6.7 Hz, 1H), 7.73 (ddd, *J* = 7.1, 5.5, 1.2 Hz, 1H), 7.58 (d, *J* = 6.2 Hz, 1H), 7.49 – 7.42 (m, 2H); **^{13}C{^{1}H} NMR (151 MHz, DMSO–d$_6$) δ=** 154.5, 145.1, 141.9, 136.8, 131.6, 129.5, 128.8, 124.7, 122.5, 118.9; **^{19}F NMR= (659 MHz, DMSO–d$_6$) δ=** -157.34, -157.47; **^{11}B NMR (193 MHz, DMSO–d$_6$) δ=** 7.95; **HRMS (ESI) (m/z):** calculated for $[M+H]^+$ $C_{11}H_9BF_2N^+$ 204.0790; found 204.0792.

3-(2-(Difluoroboraneyl)phenyl)isoquinoline (3ai):

Following the general procedure C using 3-phenylisoquinoline (123.15 mg, 0.6 mmol, 1 equiv.), BBr_3 (1.2 mL, 1.2 mmol, 2 equiv. 1M in CH_2Cl_2), 2,6-lutidine (139.8 µl, 1.2 mmol, 2 equiv.) in 2 mL CH_2Cl_2

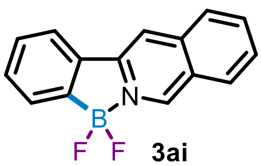

3ai

at 40 ºC for 16 h and $NaBF_4$ (164.69 mg, 1.5 mmol, 2.5 equiv.) at 70 ºC, for 4 h. The crude product was purified by 5% Et_2O:pentane wash and the desired product was obtained as beige color solid (136 mg, 89%); **Rf:** 0.41 (hexane/EtOAc, 70:30); **Mp:** 249-251 ºC; **^{1}H NMR (600 MHz, DMSO–d$_6$) δ=** 9.75 (s, 1H), 8.72 (s, 1H), 8.46 (dd, *J* = 8.3, 1.3 Hz, 1H), 8.17 (dd, *J* = 8.5, 1.1 Hz, 1H), 8.08 – 8.03 (m, 2H), 7.84 (ddd, *J* = 8.1, 6.9, 1.1 Hz, 1H), 7.59 (d, *J* = 6.9 Hz, 1H), 7.47 (td, *J* = 7.4, 1.4 Hz, 1H), 7.43 (td, *J* = 7.2, 1.2 Hz, 1H); **^{13}C{^{1}H} NMR (151 MHz, DMSO–d$_6$) δ=** 147.3, 146.5, 138.8, 137.5, 135.3, 130.6, 130.5, 129.5, 129.2, 129.0, 127.4, 127.0, 121.6, 114.9; **^{19}F NMR= (659 MHz, DMSO–d$_6$) δ=** -153.55, -153.64; **^{11}B NMR (193 MHz, DMSO–d$_6$) δ=** 8.07; **HRMS (ESI) (m/z):** calculated for $[M+H]^+$ $C_{15}H_{11}BF_2N^+$ 254.0947; found 254.0945.

1-(2-(Difluoroboraneyl)phenyl)isoquinoline (3aj):

Following the general procedure C using 1-phenylisoquinoline (123.15 mg, 0.6 mmol, 1 equiv.), BBr_3 (1.2 mL, 1.2 mmol, 2 equiv. 1M in CH_2Cl_2), 2,6-lutidine (139.8 µl, 1.2 mmol, 2 equiv.) in 2 mL CH_2Cl_2 at 40 °C for 16 h and $NaBF_4$ (164.69 mg, 1.5 mmol, 2.5 equiv.) at 70 °C, for 4 h. The crude product was purified by 5% Et_2O:pentane wash and the desired product was obtained as beige color solid (134 mg, 88%); **Rf:** 0.41 (hexane/EtOAc, 70:30); **Mp:** 186-188 °C; **^{1}H NMR (600 MHz, DMSO–d_6) δ=** 9.22 (d, J = 8.7 Hz, 1H), 8.66 (d, J = 7.4 Hz, 1H), 8.50 (d, J = 6.2 Hz, 1H), 8.29 (dd, J = 8.3, 1.2 Hz, 1H), 8.24 (d, J = 6.3 Hz, 1H), 8.11 (ddd, J = 8.1, 6.8, 1.0 Hz, 1H), 8.00 (ddd, J = 8.4, 6.9, 1.4 Hz, 1H), 7.67 (d, J = 5.9 Hz, 1H), 7.58 – 7.53 (m, 2H); **^{13}C{^{1}H} NMR (151 MHz, DMSO–d_6) δ=** 154.9, 139.6, 137.9, 134.3, 132.4, 131.8, 130.7, 129.4, 129.1, 128.6, 127.2, 126.8, 124.2, 123.6; **^{19}F NMR= (659 MHz, DMSO–d_6) δ=** -160.59, -160.68; **^{11}B NMR (193 MHz, DMSO–d_6) δ=** 7.62; **HRMS (ESI) (m/z):** calculated for $[M+H]^+$ $C_{15}H_{11}BF_2N^+$ 254.0947; found 254.0945.

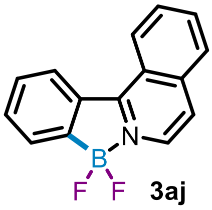

3aj

2-(2-(Difluoroboraneyl)phenyl)quinoline (3ak):

Following the general procedure C using 2-phenylquinoline (123.15 mg, 0.6 mmol, 1 equiv.), BBr_3 (1.2 mL, 1.2 mmol, 2 equiv. 1M in CH_2Cl_2), 2,6-lutidine (139.8 µl, 1.2 mmol, 2 equiv.) in 2 mL CH_2Cl_2 at 40 °C for 16 h and $NaBF_4$ (164.69 mg, 1.5 mmol, 2.5 equiv.) at 70 °C, for 4 h. The crude product was purified by 5% Et_2O:pentane wash and the desired product was obtained as beige color solid (120 mg, 79%); **Rf:** 0.41 (hexane/EtOAc, 70:30); **Mp:** 228-230 °C; **^{1}H NMR (600 MHz, DMSO–d_6) δ=** 9.02 (d, J = 8.6 Hz, 1H), 8.51 (d, J = 8.6 Hz, 1H), 8.41 (d, J = 8.7 Hz, 1H), 8.26 (dd, J = 8.2, 1.4 Hz, 1H), 8.22 (d, J = 7.5 Hz, 1H), 8.07 (ddd, J = 8.6, 6.9, 1.5 Hz, 1H), 7.80 (td, J = 7.4, 1.1 Hz, 1H), 7.66 (d, J = 7.0 Hz, 1H), 7.55 (t, J = 7.2 Hz, 1H), 7.50 (td, J = 7.5, 1.2 Hz, 1H); **^{13}C{^{1}H} NMR (151 MHz, DMSO–d_6) δ=** 156.8, 145.7, 139.3, 136.9, 133.7, 132.4, 129.4, 129.4, 128.9, 128.2, 127.8, 123.8, 121.3, 116.1; **^{19}F NMR= (659 MHz, DMSO–d_6) δ=** -154.29, -154.38; **^{11}B NMR (193 MHz, DMSO–d_6) δ=** 9.03; **HRMS (ESI) (m/z):** calculated for $[M+H]^+$ $C_{15}H_{11}BF_2N^+$ 254.0947; found 254.0945.

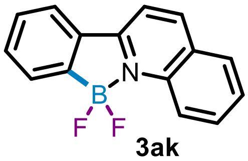

3ak

***N*-(2-(Difluoroboraneyl)phenyl)pyrimidin-2-amine (3al):**

Following the general procedure C using *N*-phenylpyrimidin-2-amine (102.72 mg, 0.6 mmol, 1 equiv.), BBr_3 (1.8 mL, 1.8 mmol, 3 equiv. 1M in CH_2Cl_2), 2,3,5,6-Tetramethylpyrazine (98.06 mg, 0.72 mmol, 1.2 equiv.) in 2 mL CH_2Cl_2 at 40 °C for 16 h and $NaBF_4$ (164.69 mg, 1.5 mmol, 2.5 equiv.) at 70 °C, for 4 h. The crude product was purified by pentane wash and the desired product was obtained as beige color solid (73 mg, 55%); **Rf:** 0.36 (hexane/EtOAc, 50:50); **Mp:** 281-281 °C; **^{1}H NMR (600 MHz, DMSO–d_6) δ=** 11.63

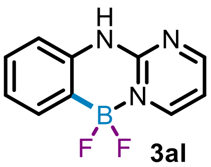

3al

(bs, 1H), 8.94 (dd, J = 4.3, 2.3 Hz, 1H), 8.63 (dd, J = 6.2, 2.3 Hz, 1H), 7.52 (dd, J = 7.4, 1.5 Hz, 1H), 7.33 (ddd, J = 8.5, 7.2, 1.6 Hz, 1H), 7.25 – 7.20 (m, 2H), 7.13 (td, J = 7.3, 1.1 Hz, 1H); **$^{13}C\{^{1}H\}$ NMR (151 MHz, DMSO–d$_6$) δ=** 164.5, 151.7, 148.0, 138.1, 130.9, 128.4, 123.5, 115.4, 112.4; **^{19}F NMR= (659 MHz, DMSO–d$_6$) δ=** -132.68, -132.76; **^{11}B NMR (193 MHz, DMSO–d$_6$) δ=** 3.14; **HRMS (ESI) (m/z):** calculated for $[M–H]^-$ $C_{10}H_7BF_2N_3^-$ 218.0706; found 218.0704.

3.7 General Procedure D for reaction optimization: Late-stage diversification

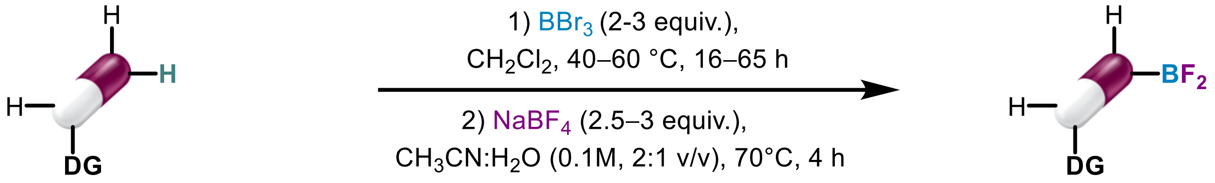

1am-1at **3am-3at**

Step i) To a dry 25 mL microwave vial, equipped with a rubber septum, stir bar, the *N*-heteroarene derivative (0.6 mmol, 1 equiv.) in anhydrous CH_2Cl_2 (2 mL), was added dropwise BBr_3 (1.2 mL, 1.2 mmol, 2 equiv., 1M solution in CH_2Cl_2). After the complete addition of BBr_3, the reaction mixture was stirred at 40 ºC for 16 h after which the solvent was removed under reduced pressure.

Step ii) To the crude residue from step i) were added $NaBF_4$ (164.69 mg, 1.5 mmol, 2.5 equiv.), 4 mL acetonitrile, 2 mL distilled water and the reaction mixture was heated at 70 ºC for 4 h. The reaction was allowed to reach room temperature and the acetonitrile was evaporated under *vacuo* to afford the crude solid. After removal of complete acetonitrile, the crude solid was taken in 20 mL distilled water and filtered. Additional 30 mL water wash was given to remove inorganic impurities. The crude solid was stirred in pentane or 5% Et_2O:Pentane to remove unreacted starting material.

***N*-(2-(difluoroboraneyl)phenyl)-2,4,5-trimethylfuran-3-carboxamide (3am):**

Following the general procedure D using 2,4,5-trimethyl-*N*-phenylfuran-3-carboxamide (22.93 mg, 0.1 mmol, 1 equiv.), BBr_3 (120 µL, 0.12 mmol, 1.2 equiv. 1M in CH_2Cl_2), in 0.2 mL CH_2Cl_2 at 40 ºC for 16

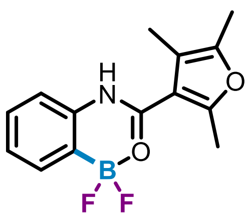

3am

h and $NaBF_4$ (32.94 mg, 0.3 mmol, 3 equiv.) at 70 ºC, for 4 h. The crude product was purified by pentane wash and the desired product was obtained as beige color solid (24.6 mg, 89%); **Rf:** 0.30 (hexane/EtOAc, 70:30); **Mp:** 251-253 ºC; **^{1}H NMR (700 MHz, DMSO–d$_6$) δ=** 12.47 (bs, 1H), 7.49 (d, J = 7.3 Hz, 1H), 7.39 (t, J = 7.7 Hz, 1H), 7.31 (t, J = 7.3 Hz, 1H), 7.25 (d, J = 8.0 Hz, 1H), 2.48 (s, 3H), 2.23 (s, 3H), 2.06 (s, 3H); **$^{13}C\{^{1}H\}$ NMR (151 MHz, DMSO–d$_6$) δ=** 162.5, 155.2, 147.2, 136.7, 130.7, 128.3, 127.0, 116.8, 113.8, 113.3, 13.4, 10.8, 8.4; **^{19}F NMR= (659 MHz, DMSO–d$_6$) δ=** -129.54, -129.61; **^{11}B NMR (193 MHz, DMSO–d$_6$) δ=** 3.04; **HRMS (ESI) (m/z):** calculated for $[M–H]^-$ $C_{14}H_{13}BF_2NO_2^-$ 276.1012; found 276.1009.

4-((3-(Difluoroboraneyl)-5,5,8,8-tetramethyl-5,6,7,8-tetrahydronaphthalen-2-yl)carbamoyl) benzoic acid (3an):

Following the general procedure D using 4-((5,5,8,8-tetramethyl-5,6,7,8-tetrahydronaphthalen-2-yl)carbamoyl)benzoic acid (52.72 mg, 0.15 mmol, 1 equiv.), BBr_3 (262 µL, 0.26 mmol, 1.75 equiv. 1M in CH_2Cl_2), in 0.5 mL CH_2Cl_2 at 40 ºC for 24 h and $NaBF_4$ (49.41 mg, 0.3 mmol, 3 equiv.) in 1.5 mL ACN and 0.5 mL water at 70 ºC, for 4 h. Note: Due to solubility issues, an additional 0.5 mL of ACN was used in the reaction. The crude product was purified by 20% diethyl ether:pentane wash and the desired product was obtained as off white solid (39 mg, 65%); **Rf:** 0.18 (EtOAc, 100); **Mp:** 217-219 ºC; **^{1}H NMR (600 MHz, DMSO–d$_6$) δ=** 13.50 (bs, 1H), 12.96 (bs, 1H), 8.28 (d, J = 8.6 Hz, 2H), 8.20 (d, J = 8.5 Hz, 2H), 7.45 (s, 1H), 7.42 (s, 1H), 1.67 (s, 4H), 1.28 (s, 6H), 1.27 (s, 6H); **^{13}C{^{1}H} NMR (151 MHz, DMSO–d$_6$) δ=** 166.3, 163.3, 144.9, 144.0, 135.5, 134.6, 131.9, 129.8, 128.9, 128.5, 115.0, 34.6, 34.3, 34.0, 33.9, 31.8, 31.7; **^{19}F NMR= (659 MHz, DMSO–d$_6$) δ=** -128.45, -128.52; **^{11}B NMR (193 MHz, DMSO–d$_6$) δ=** 3.24; **HRMS (ESI) (m/z):** calculated for $[M–H]^-$ $C_{22}H_{23}BF_2NO_3^-$ 398.1744; found 398.1778.

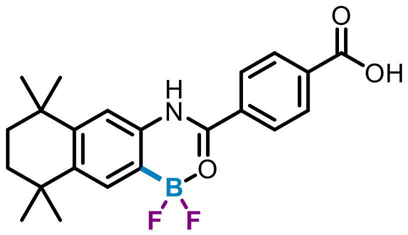

3an

2-(3-(Difluoroboraneyl)-4-(1-oxoisoindolin-2-yl)phenyl)propanoic acid (3ao):

Following the general procedure D using 2-(4-(1-oxoisoindolin-2-yl)phenyl)propanoic acid (56.26 mg, 0.2 mmol, 1 equiv.), BBr_3 (400 µL, 0.4 mmol, 2.0 equiv. 1M in CH_2Cl_2), in 0.5 mL CH_2Cl_2 at 60 ºC for 19 h and $NaBF_4$ (49.41 mg, 0.3 mmol, 3 equiv.) in 1.3 mL ACN and 0.7 mL water at 70 ºC, for 4 h. The crude product was purified by 20% diethyl ether:pentane wash and the desired product was obtained as off white solid (55 mg, 84%); **Rf:** 0.28 (DCM/MeOH, 95:5); **Mp:** decomposed after 230 ºC; **^{1}H NMR (600 MHz, DMSO–d$_6$) δ=** 12.33 (bs, 1H), 8.06 (dt, J = 7.8, 1.0 Hz, 1H), 7.90 – 7.84 (m, 2H), 7.70 (ddd, J = 8.1, 6.6, 1.7 Hz, 1H), 7.46 (t, J = 1.8 Hz, 1H), 7.40 (dd, J = 8.4, 2.1 Hz, 1H), 7.35 (d, J = 8.4 Hz, 1H), 5.48 (s, 2H), 3.76 (q, J = 7.1 Hz, 1H), 1.39 (d, J = 7.1 Hz, 3H); **^{13}C{^{1}H} NMR (151 MHz, DMSO–d$_6$) δ=** 175.3, 165.9, 142.5, 140.2, 135.6, 134.5, 130.1, 129.1, 128.3, 127.7, 123.7 (2C), 115.1, 52.5, 44.4, 18.6; **^{19}F NMR= (659 MHz, DMSO–d$_6$) δ=** -127.51 – -127.96 (m, 2F); **^{11}B NMR (193 MHz, DMSO–d$_6$) δ=** 3.56; **HRMS (ESI) (m/z):** calculated for $[M–H]^-$ $C_{17}H_{13}BF_2NO_3^-$ 328.0962; found 328.1020.

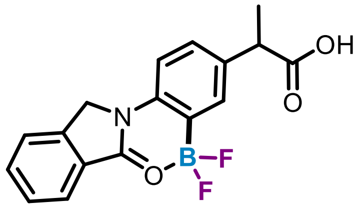

3ao

3-(2-(Difluoroboraneyl)phenyl)-1,1-dimethylurea (3ap):

Following the general procedure A using 1,1-dimethyl-3-phenylurea (98.52 mg, 0.6 mmol, 1 equiv.), BBr_3 (1.8 mL, 1.8 mmol, 3 equiv. 1M in CH_2Cl_2), in 2 mL CH_2Cl_2 at 60 ºC for 65 h and $NaBF_4$ (164.69 mg, 1.5 mmol, 2.5 equiv.) at 70 ºC, for 4 h. The crude product was purified by 30% Et_2O:Pentane and the desired product was obtained as an off white solid (80 mg, 63%); **Rf:** 0.16 (hexane/EtOAc, 70:30); **Mp:** 280-282 ºC; **^{1}H NMR (600 MHz, DMSO–d$_6$) δ=** 10.06 (bs, 1H), 7.32 (d, *J* = 7.2 Hz, 1H), 7.23 (td, *J* = 7.7, 1.6 Hz, 1H), 7.18 (d, *J* = 7.9 Hz, 1H), 7.06 – 7.02 (m, 1H), 3.11 (s, 6H); **^{13}C{^{1}H} NMR (151 MHz, DMSO–d$_6$) δ=** 154.9, 138.6, 130.3, 127.8, 123.7, 115.1, 36.7; **^{19}F NMR= (659 MHz, DMSO–d$_6$) δ=** -134.10, -134.17; **^{11}B NMR (193 MHz, DMSO–d$_6$) δ=** 3.13; **HRMS (ESI) (m/z):** calculated for $[M-H]^-$ $C_9H_{10}BF_2N_2O^-$ 211.0859; found 211.0855.

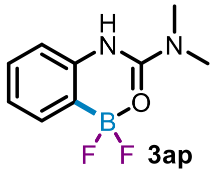

3ap

11-(2-(Difluoroboraneyl)phenyl)dibenzo[*b,f*][1,4]oxazepine (3aq):

Following the general procedure C using 11-phenyldibenzo[*b,f*][1,4]oxazepine (40.7 mg, 0.15 mmol, 1 equiv.), 2,6-lutidine (34.9 µL, 0.3 mmol, 2 equiv.), BBr_3 (300 µL, 0.3 mmol, 2 equiv. 1M in CH_2Cl_2), in 0.5 mL CH_2Cl_2 at 40 ºC for 4 h and $NaBF_4$ (41.18 mg, 0.375 mmol, 2.5 equiv.) at 70 ºC, for 4 h. The crude product was purified by pentane wash and the desired product was obtained as beige color solid (34 mg, 71%); **Rf:** 0.69 (EtOAc, 100); **Mp:** 245-247 ºC; **^{1}H NMR (600 MHz, DMSO–d$_6$) δ=** 8.06 (d, *J* = 7.8 Hz, 1H), 8.02 (d, *J* = 8.2 Hz, 1H), 7.96 – 7.91 (m, 1H), 7.78 (d, *J* = 7.8 Hz, 1H), 7.74 – 7.68 (m, 2H), 7.65 (d, *J* = 8.2 Hz, 1H), 7.62 – 7.52 (m, 4H), 7.49 – 7.44 (m, 1H); **^{13}C{^{1}H} NMR (151 MHz, DMSO–d$_6$) δ=** 174.1, 161.2, 153.9, 137.2, 136.7, 134.3, 132.3, 131.6, 131.4, 129.1, 128.9, 128.5, 126.9, 126.4, 124.5, 124.4, 121.9, 121.7, 120.9; **^{19}F NMR= (659 MHz, DMSO–d$_6$) δ=** (BF_1): -153.78, -153.86, (BF_{1*}): -153.96, -154.04; (BF_2): -159.53, -159.61, (BF_{2*}): -159.70, -159.79; **^{11}B NMR (193 MHz, DMSO–d$_6$) δ=** 9.01; **HRMS (ESI) (m/z):** calculated for [M+Na] $C_{19}H_{12}BF_2NNaO$ 342.08777; found 342.08684.

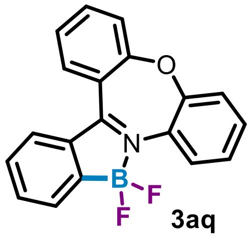

3aq

2-(2-(Difluoroboraneyl)phenyl)quinoline-4-carboxylic acid (3ar):

Following the general procedure C using 2-phenylquinoline-4-carboxylic acid (249.26 mg, 1.0 mmol, 1 equiv.), 2,3,5,6-Tetramethylpyrazine (204.29 mg, 1.5 mmol, 1.5 equiv.), BBr_3 (3.0 mL, 3.0 mmol, 3 equiv. 1M in CH_2Cl_2), in 3 mL CH_2Cl_2 at 40 ºC for 19 h and $NaBF_4$ (329.38 mg, 3.0 mmol, 3 equiv.) at 70 ºC, for 4 h. The crude product was purified by 20% Et_2O:Pentane wash and the desired product was obtained as yellow solid (133 mg, 45%); **Rf:** 0.11 (EtOAc, 100); **Mp:** 228-230 ºC; **^{1}H NMR (600 MHz, DMSO–d$_6$) δ=** 14.77 (bs, 1H), 8.79 (s, 1H), 8.63 (dd, *J* = 8.6, 1.4 Hz, 1H), 8.48 (d, *J* = 8.7 Hz, 1H), 8.33 (d, *J*

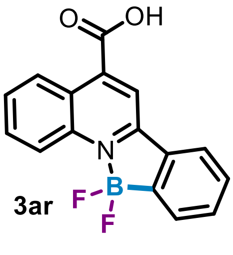

3ar

= 7.5 Hz, 1H), 8.13 (ddd, *J* = 8.6, 6.9, 1.5 Hz, 1H), 7.88 (ddd, *J* = 8.3, 6.9, 1.2 Hz, 1H), 7.67 (d, *J* = 7.0 Hz, 1H), 7.57 (t, *J* = 7.2 Hz, 1H), 7.51 (t, *J* = 7.5 Hz, 1H); **$^{13}C\{^1H\}$ NMR (151 MHz, DMSO–d_6) δ=** 166.4, 156.9, 145.7, 140.1, 136.5, 133.8, 132.8, 129.4, 129.0, 128.8, 126.7, 124.7, 124.3, 121.9, 116.3; **^{19}F NMR= (659 MHz, DMSO–d_6) δ=** -153.36, -153.44; **^{11}B NMR (193 MHz, DMSO–d_6) δ=** 9.32; **HRMS (ESI) (m/z):** calculated for $[M\text{-}H]^-$ $C_{16}H_9BF_2NO_2^-$ 295.0731; found 295.0736.

4-(Difluoroboraneyl)-3-(3,3-dimethylureido)benzoic acid (3as):

Following the general procedure B using 1,1-dimethyl-3-(3-(trifluoromethyl)phenyl)urea (139.32 mg, 0.6 mmol, 1 equiv.), BBr_3 (1.8 mL, 1.8 mmol, 3 equiv. 1M in CH_2Cl_2), in 2 mL CH_2Cl_2 at 40 °C for 19 h and

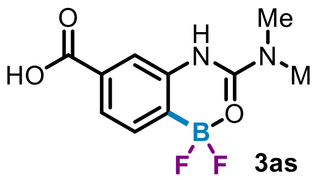

3as

$NaBF_4$ (164.69 mg, 1.5 mmol, 2.5 equiv.) at 70 °C, for 4 h. The crude product was purified by pentane wash and the desired product was obtained as an off white solid (93 mg, 61%); **Rf:** 0.77 (EtOAc/MeOH, 70:30); **Mp:** Decomposed after 370 °C; **^1H NMR (600 MHz, DMSO–d_6) δ=** 12.88 (bs, 1H), 10.26 (bs, 1H), 7.85 (d, *J* = 1.5 Hz, 1H), 7.62 (dd, *J* = 7.5, 1.5 Hz, 1H), 7.43 (d, *J* = 7.6 Hz, 1H), 3.15 (s, 3H), 3.10 (s, 3H); **$^{13}C\{^1H\}$ NMR (151 MHz, DMSO–d_6) δ=** 167.3, 154.9, 138.9, 130.5, 130.4, 124.3, 116.1, 36.8; **^{19}F NMR= (659 MHz, DMSO–d_6) δ=** -134.64, -134.71; **^{11}B NMR (193 MHz, DMSO–d_6) δ=** 2.95; **HRMS (ESI) (m/z):** calculated for $[M\text{-}H]^-$ $C_{10}H_{10}BF_2N_2O_3^-$ 255.07580; found 255,07594.

4-(Difluoroboraneyl)-3-(3,3-dimethylureido)-*N*-phenylbenzamide (3as′):

Following the literature procedure[2] using 4-(difluoroboraneyl)-3-(3,3-dimethylureido)benzoic acid (20.0 mg, 0.0781 mmol, 1 equiv.), aniline (8.0 mg, 0.0859 mmol, 1.1 equiv.), EDCI.HCl (19.47 mg, 0.1016

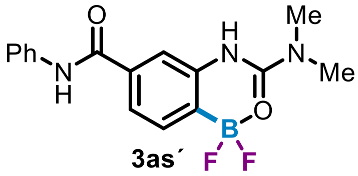

3as′

mmol, 1.3 equiv.), DMAP (950 µg, 0.00781 mmol, 0.1 equiv.), in 1 mL dry CH_2Cl_2 at rt for 18 h. The crude product was transfered and filtered using water and purified by pentane wash. The desired product was obtained as an off white solid (22 mg, 85%); **Rf:** 0.21 (DCM/MeOH, 95:5); **Mp:** 316-318 °C; **^1H NMR (600 MHz, DMSO–d_6) δ=** 10.31 (bs, 1H), 10.21 (bs, 1H), 7.79 (s, 1H), 7.77 (d, *J* = 7.5 Hz, 2H), 7.68 (dd, *J* = 7.6, 1.6 Hz, 1H), 7.46 (d, *J* = 7.6 Hz, 1H), 7.38 – 7.33 (m, 2H), 7.10 (t, *J* = 7.3 Hz, 1H), 3.14 (s, 6H); **$^{13}C\{^1H\}$ NMR (151 MHz, DMSO–d_6) δ=** 165.5, 154.9, 139.2, 139.0, 134.7, 130.2, 128.6, 123.6, 122.3, 120.3, 114.9, 36.8; **^{19}F NMR= (659 MHz, DMSO–d_6) δ=** -134.38, -134.46; **^{11}B NMR (193 MHz, DMSO–d_6) δ=** 2.74; **HRMS (ESI) (m/z):** calculated for $[M\text{-}H]^-$ $C_{16}H_{15}BF_2N_3O_2^-$ 330.1230; found 330.1300.

2-(2-(Difluoroboraneyl)phenyl)-5-methyl-1,2-dihydro-3*H*-pyrazol-3-one (3at):

Following the general procedure D using 5-methyl-2-phenyl-2,4-dihydro-3*H*-pyrazol-3-one (104.52 mg, 0.6 mmol, 1 equiv.), BBr_3 (1.8 mL, 1.8 mmol, 3 equiv. 1M in CH_2Cl_2), in 2 mL CH_2Cl_2 at 60 ºC for 24 h

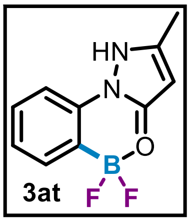

3at

and $NaBF_4$ (164.69 mg, 1.5 mmol, 2.5 equiv.) at 70 ºC, for 4 h. The crude product was purified by pentane wash and the desired product was obtained as an off white solid (119 mg, 89%); **Rf:** 0.47 (EtOAc/MeOH, 85:15); **Mp:** 247-249 ºC; **^{1}H NMR (600 MHz, DMSO–d$_6$) δ=** 7.52 – 7.46 (m, 2H), 7.42 (td, *J* = 7.7, 1.5 Hz, 1H), 7.25 (td, *J* = 7.3, 1.0 Hz, 1H), 5.86 (s, 1H), 2.37 (s, 3H); **^{13}C{^{1}H} NMR (151 MHz, DMSO–d$_6$) δ=** 155.2, 148.1, 135.6, 131.6, 128.1, 125.9, 111.5, 91.4, 12.0; **^{19}F NMR= (659 MHz, DMSO–d$_6$) δ=** -133.70; **^{11}B NMR (193 MHz, DMSO–d$_6$) δ=** 2.61; **HRMS (ESI) (m/z):** calculated for $[M\text{-}H]^-$ $C_{10}H_8BF_2N_2O^-$ 221.0732; found 221.0702.

1-(7-(difluoroboraneyl)-1*H*-indol-1-yl)-2,2-dimethylpropan-1-one (3au):

Following the general procedure A using 1-(1*H*-indol-1-yl)-2,2-dimethylpropan-1-one (120.76 mg, 0.6 mmol, 1 equiv.), BBr_3 (720 µL, 0.72 mmol, 1.2 equiv. 1M in CH_2Cl_2), in 2 mL CH_2Cl_2 at 22 ºC for 1 h

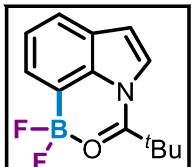

3au

and KHF_2 (234.31 mg, 3 mmol, 5 equiv.) in 2 mL MeOH and 4 mL CH_3CN at 22 ºC, for 22 h. The crude product was purified by pentane and 10% diethyl ether:pentane wash. The desired product was obtained as bedge color solid (84.1 mg, 56%); **Rf:** 0.12 (hexane/EtOAc, 70:30); **^{1}H NMR (600 MHz, CDCl$_3$) δ=** 7.72 (d, *J* = 7.2 Hz, 1H), 7.63 (d, *J* = 3.9 Hz, 1H), 7.53 (d, *J* = 7.7 Hz, 1H), 7.47 (t, *J* = 7.4 Hz, 1H), 6.92 (d, *J* = 4.0 Hz, 1H), 1.62 (s, 9H); **^{13}C{^{1}H} NMR (151 MHz, CDCl$_3$) δ=** 179.1, 138.2, 129.7, 128.1, 126.1, 123.5, 121.2, 117.6, 40.6, 28.6; **^{19}F NMR= (564 MHz, CDCl$_3$) δ=** -128.04, -128.16; **^{11}B NMR (193 MHz, CDCl$_3$) δ=** 4.73; Hydroxylated mass was observed: **HRMS (ESI) (m/z):** calculated for $[M]^+$ $C_{13}H_{15}BNO_2$ 228.11958; found 228.11901.

Purification of **3au**: After evaporating the solvent in the second step, a pinkish-beige solid was obtained. This solid was stirred with 20 mL of pentane at room temperature, and the pentane was decanted. This trituration process was repeated twice. The solid was then stirred in a 10% diethyl ether:pentane solution, and the organic layer was decanted. After the product was confirmed to be pure by TLC, the solid was transferred to a filter and washed with a small amount of pentane. Finally, the solid was washed portionwise with 30 mL of water to remove inorganic impurities.

3.8 General Procedure E for reaction optimization: (7a-7e)

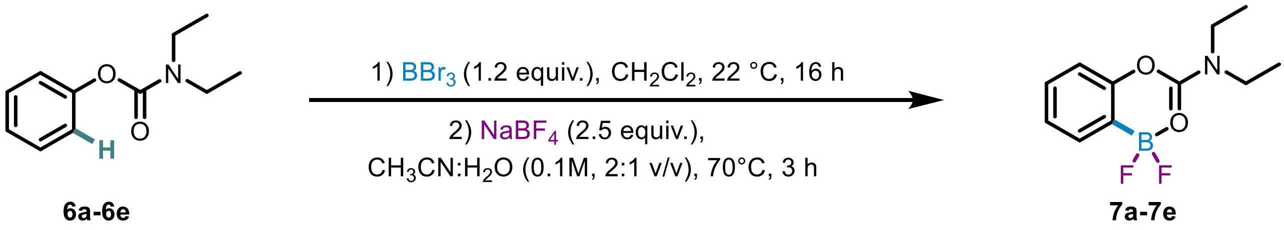

Step i) To a dry 25 mL microwave vial, equipped with a rubber septum, stir bar, the carbamate derivative (0.6 mmol, 1 equiv.) in anhydrous CH_2Cl_2 (2 mL) under a nitrogen atmosphere was added dropwise BBr_3 (720 µl, 0.72 mmol, 1.2 equiv., 1M solution in CH_2Cl_2). After the complete addition of BBr_3, the reaction mixture was stirred at 22 °C for 16 h after which the solvent was removed under reduced pressure at 30 °C.

Step ii) To the crude residue from step i) were added $NaBF_4$ (164.69 mg, 1.5 mmol, 2.5 equiv.), 4 mL acetonitrile, 2 mL distilled water and the reaction mixture was heated at 70 °C for 3 h. The reaction was allowed to reach room temperature and the acetonitrile was evaporated under *vacuo* to afford the crude solid. After removal of complete acetonitrile, the crude solid was taken in 10 mL distilled water and filtered. Additional 20 mL water wash was given to remove inorganic impurities. The crude solid was washed with pentane to remove unreacted starting material.

2-(difluoroboraneyl)phenyl diethylcarbamate (7a):

Following the general procedure E using phenyl diethylcarbamate (115.95 mg, 0.6 mmol, 1 equiv.), BBr_3 (720µL, 0.72 mmol, 1.2 equiv. 1M in CH_2Cl_2), in 2 mL CH_2Cl_2 at 22 °C for 16 h and $NaBF_4$ (164.69 mg,

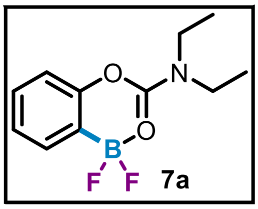

1.5 mmol, 2.5 equiv.) at 70 °C, for 3 h. The crude product was purified by pentane wash and the desired product was obtained as an off white solid (128.72 mg, 89%); **Rf:** 0.13 (hexane/EtOAc, 50:50); **Mp:** 85-88 °C; **^{1}H NMR (800 MHz, CDCl₃) δ=** 7.64 (d, J = 7.1 Hz, 1H), 7.31 (td, J = 7.8, 1.8 Hz, 1H), 7.26 (t, 7.2 Hz, 1H), 6.98 (d, J = 8.2 Hz, 1H), 3.60 (q, J = 7.2 Hz, 4H), 1.35 (t, J = 7.2 Hz, 3H), 1.32 (t, J = 7.2 Hz, 3H); **^{13}C{^{1}H} NMR (201 MHz, $CDCl_3$) δ=** 156.6, 153.8, 131.9, 129.2, 126.5, 114.4, 43.4, 42.9, 13.5, 13.0; **^{19}F NMR= (659 MHz, $CDCl_3$) δ=** -139.48, -139.57; **^{11}B NMR (193 MHz, $CDCl_3$) δ=** 2.95; **HRMS (ESI) (m/z):** calculated for [M+Na] $C_{11}H_{14}BF_2NNaO_2$ 264.09834; found 264.09778.

2-(difluoroboraneyl)-4-(2-phenylpropan-2-yl)phenyl diethylcarbamate (7b):

Following the general procedure E using 4-(2-phenylpropan-2-yl)phenyl diethylcarbamate (186.85 mg,

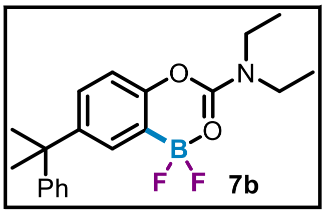

0.6 mmol, 1 equiv.), BBr_3 (720µL, 0.72 mmol, 1.2 equiv. 1M in CH_2Cl_2), in 2 mL CH_2Cl_2 at 22 °C for 16 h and $NaBF_4$ (164.69 mg, 1.5 mmol, 2.5 equiv.) at 70 °C, for 3 h. The crude product was purified by pentane wash and the desired product was obtained as an off white solid (183.2 mg, 85%); **Rf:** 0.15

(hexane/EtOAc, 50:50); **Mp:** 110-112 ºC; **^{1}H NMR (600 MHz, CDCl$_3$) δ=** 7.65 (d, *J* = 2.6 Hz, 1H), 7.26 – 7.20 (m, 4H), 7.18 – 7.14 (m, 1H), 7.04 (dd, *J* = 8.7, 2.5 Hz, 1H), 6.85 (d, *J* = 8.7 Hz, 1H), 3.58 (dq, *J* = 11.3, 7.2 Hz, 4H), 1.68 (s, 6H), 1.32 (td, *J* = 7.2, 5.5 Hz, 6H); **^{13}C{^{1}H} NMR (151 MHz, CDCl$_3$) δ=** 156.6, 151.9, 150.7, 148.8, 129.1, 128.7, 128.1, 126.9, 125.8, 114.1, 43.4, 42.9, 30.9, 13.5, 13.0; **^{19}F NMR= (659 MHz, CDCl$_3$) δ=** -139.43; **^{11}B NMR (193 MHz, CDCl$_3$) δ=** 3.13; **HRMS (ESI) (m/z):** calculated for [M+Na] $C_{20}H_{24}BF_2NNaO_2$ 382.17659; found 382.17579.

2-(difluoroboraneyl)-3,6-dimethylphenyl diethylcarbamate (7c):

Following the general procedure E using 2,5-dimethylphenyl diethylcarbamate (132.78 mg, 0.6 mmol, 1 equiv.), BBr_3 (720µL, 0.72 mmol, 1.2 equiv. 1M in CH_2Cl_2), in 2 mL CH_2Cl_2 at 22 ºC for 16 h and $NaBF_4$

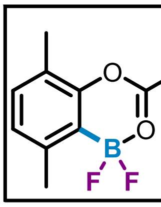
7c

(164.69 mg, 1.5 mmol, 2.5 equiv.) at 70 ºC, for 3 h. The crude product was purified by pentane wash and the desired product was obtained as an off white solid (121.09 mg, 75%); **Rf:** 0.17 (hexane/EtOAc, 50:50); **Mp:** 98-100 ºC; **^{1}H NMR (800 MHz, CDCl$_3$) δ=** 7.02 (d, *J* = 7.5 Hz, 1H), 6.94 (d, *J* = 7.6 Hz, 1H), 3.60 (dq, *J* = 11.2, 7.2 Hz, 4H), 2.47 (s, 3H), 2.25 (s, 3H), 1.36 (t, *J* = 7.2 Hz, 3H), 1.33 (t, *J* = 7.2 Hz, 3H); **^{13}C{^{1}H} NMR (151 MHz, CDCl$_3$) δ=** 156.2, 152.4, 140.9, 130.3, 127.3, 120.4, 43.3, 42.9, 20.9, 15.7, 13.2, 13.1; **^{19}F NMR= (659 MHz, CDCl$_3$) δ=** -137.58, -137.67; **^{11}B NMR (193 MHz, CDCl$_3$) δ=** 3.11; **HRMS (ESI) (m/z):** calculated for [M+Na] $C_{13}H_{18}BF_2NNaO_2$ 292.12964; found 292.12894.

2-(difluoroboraneyl)naphthalen-1-yl diethylcarbamate (7d):

Following the general procedure E using naphthalen-1-yl diethylcarbamate (145.98 mg, 0.6 mmol, 1 equiv.), BBr_3 (720µL, 0.72 mmol, 1.2 equiv. 1M in CH_2Cl_2), in 2 mL CH_2Cl_2 at 22 ºC for 16 h and $NaBF_4$

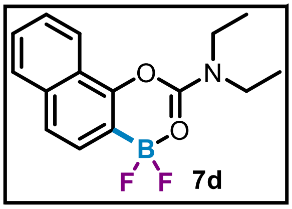
7d

(164.69 mg, 1.5 mmol, 2.5 equiv.) at 70 ºC, for 3 h. The crude product was purified by pentane wash and the desired product was obtained as an off white solid (153.69 mg, 88%); **Rf:** 0.11 (hexane/EtOAc, 50:50); **Mp:** 84-86 ºC; **^{1}H NMR (800 MHz, CDCl$_3$) δ=** 7.94 (d, *J* = 8.2 Hz, 1H), 7.86 (d, *J* = 8.0 Hz, 1H), 7.73 (d, *J* = 8.1 Hz, 1H), 7.69 (d, *J* = 8.1 Hz, 1H), 7.57 – 7.49 (m, 2H), 3.76 (q, *J* = 7.3 Hz, 2H), 3.66 (q, *J* = 7.3 Hz, 2H), 1.47 (t, *J* = 7.4 Hz, 3H), 1.36 (t, *J* = 7.3 Hz, 3H); **^{13}C{^{1}H} NMR (151 MHz, CDCl$_3$) δ=** 156.6, 148.6, 134.6, 128.3, 127.6, 126.7, 126.5, 125.9, 122.4, 119.5, 43.6, 43.3, 13.4, 13.0; **^{19}F NMR= (659 MHz, CDCl$_3$) δ=** -137.52; **^{11}B NMR (193 MHz, CDCl$_3$) δ=** 3.15; **HRMS (ESI) (m/z):** calculated for [M+Na] $C_{15}H_{16}BF_2NNaO_2$ 314.11399; found 314.11316.

(1,4-phenylenebis(propane-2,2-diyl))bis(2-(difluoroboraneyl)-4,1-phenylene) bis(diethylcarbamate) (7e):

Following the general procedure E using (1,4-phenylenebis(propane-2,2-diyl))bis(4,1-phenylene) bis(diethylcarbamate) (163.42 mg, 0.3 mmol, 1 equiv.), BBr_3 (750µL, 0.75 mmol, 2.5 equiv. 1M in CH_2Cl_2), in 1.5 mL CH_2Cl_2 at 22 ºC for 16 h and $NaBF_4$ (164.69 mg, 1.5 mmol, 5 equiv.) in 2.5 mL CH_3CN and 1 mL water at 70 ºC, for 3 h. The crude product was purified by pentane was7ch and the desired product was obtained as an off white solid (174.80 mg, 91%); **Rf:** 0.08 (hexane/EtOAc, 50:50); **Mp:** 231-233 ºC; **^{1}H NMR (600 MHz, $CDCl_3$) δ=** 7.63 (d, J = 2.5 Hz, 2H), 7.09 – 7.06 (m, 5H), 7.05 (d, J = 2.5 Hz, 1H), 6.86 (dd, J = 8.7, 1.2 Hz, 2H), 3.58 (dq, J = 9.6, 7.2 Hz, 8H), 1.65 (s, 12H), 1.31 (q, J = 7.1 Hz, 12H); **^{13}C{^{1}H} NMR (151 MHz, $CDCl_3$) δ=** 156.7, 151.8, 148.9, 147.7, 129.1, 128.7, 126.4 (2C), 114.0, 43.4, 42.9, 42.6, 30.9, 13.5, 13.0; **^{19}F NMR= (659 MHz, $CDCl_3$) δ=** -139.38, -139.46; **^{11}B NMR (193 MHz, $CDCl_3$) δ=** 3.09; **HRMS (ESI) (m/z):** calculated for [M+Na] $C_{34}H_{42}B_2F_4N_2NaO_4$ 663.31645; found 663.315.

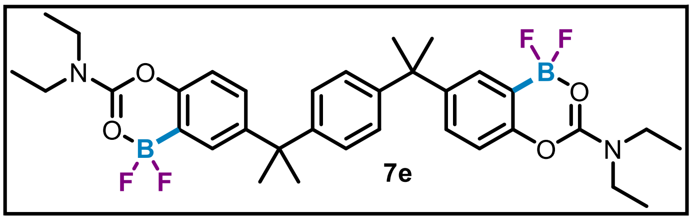

7e

4. Radioiodination

4.1 Optimization: Radioiodination of BF_2-arene 3a:

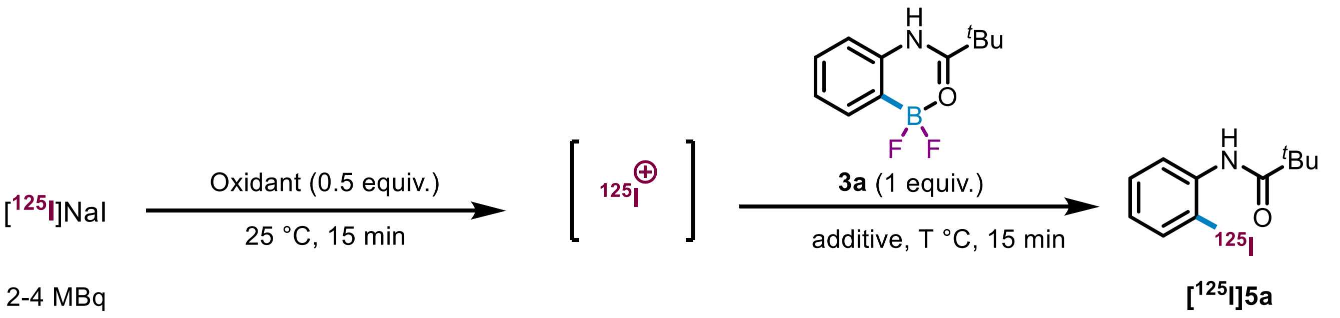

In a V-vial equipped with a stir bar, the chosen oxidant (0.5 equiv.) was added to a methanolic solution of [^{125}I]sodium iodide (5 µL, 2-4 MBq). The resulting mixture was stirred for 15 minutes at 25 °C and the substrate **3a** (1 equiv.) was added. The reaction mixture was stirred again for 15 minutes at the chosen temperature. After reaction, the mixture was quenched with a solution of sodium thiosulfate (200 µL, 0.05 M in water) and diluted with methanol (1100 µL). An aliquot was removed for analysis by radio-HPLC to assess the radiochemical conversion.

Entry	oxidant	$ArBF_2$ (3a)	T (°C)	additive	RCC
1	NCS 0.04M in MeOH 40 µL	0.16M in MeOH 20 µL	25	-	45%
2	NCS 0.04M in DMF 40 µL	0.16M in MeOH 20 µL	25	-	3%
3	Selectfluor 0.04M in H_2O 40 µL	0.16M in MeOH 20 µL	25	-	18%
4	NCS 0.04M in MeOH 40 µL	0.16M in MeOH 20 µL	50	-	76%
5	NCS 0.04M in MeOH 40 µL	0.16M in MeOH 20 µL	65	-	93%
6	NCS 0.04M in MeCN 40 µL	0.16M in MeCN 20 µL	65	-	78%
7	NCS 0.04M in MeOH 40 µL	0.16M in MeOH 20 µL	50	TFA 20 µL	80%

Table S2: Optimization: Radioiodination of $ArBF_2$ (**3a**)

4.2 General procedure for radioiodination scope:

In a V-vial equipped with a stir bar, *N*-chlorosuccinimide (40 µL, 0.04 M in MeOH, 1.6 mmol, 0.5 equiv.) was added to a methanolic solution of [^{125}I]sodium iodide (5 µL, 2-4 MBq). The resulting mixture was stirred for 15 minutes at 25 °C and the substrate **ArBF₂** (20 µL, 0.16 M in MeOH, 3 mmol, 1 equiv.) was added. The reaction mixture was stirred again for 15 or 30 minutes at 65 °C. After reaction, the mixture was quenched with a solution of sodium thiosulfate (200 µL, 0.05 M in water) and diluted with methanol (1100 µL). An aliquot was removed for analysis by radio-HPLC to assess the radiochemical conversion.

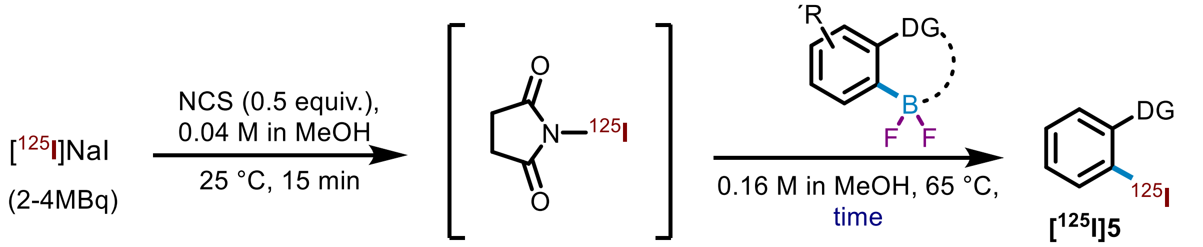

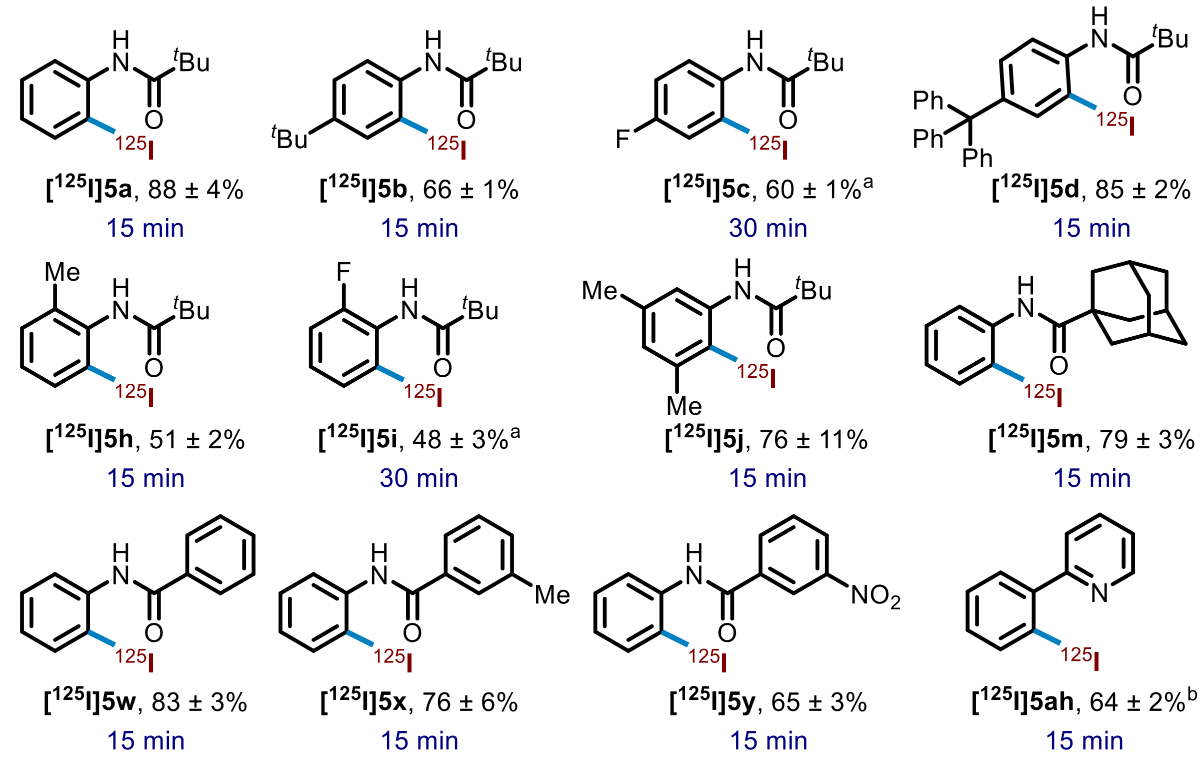

4.3 Radioiodination of [^{125}I]5ag

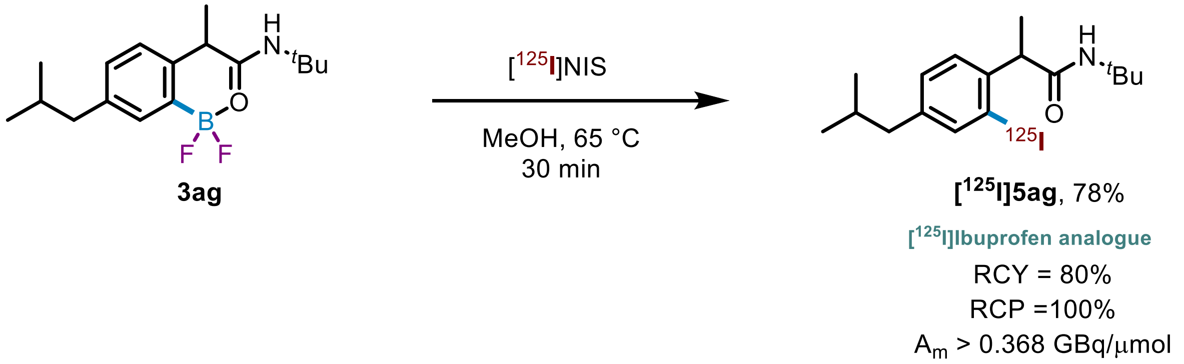

In a V-vial equipped with a stir bar, *N*-chlorosuccinimide (40 µL, 0.04 M in MeOH, 1.6 mmol, 0.5 equiv.) was added to a methanolic solution of [^{125}I]sodium iodide (5 µL, 2-4 MBq). The resulting mixture was stirred for 15 minutes at 25 °C and the substrate **3ag** (20 µL, 0.16 M in MeOH, 3 mmol, 1 equiv.) was added. The reaction mixture was stirred again for 30 minutes at 65 °C. After reaction, the mixture was quenched with a solution of sodium thiosulfate (200 µL, 0.05 M in water) and diluted with water (1100 µL). An aliquot was removed for analysis by radio-HPLC to assess the radiochemical conversion (RCC = 96%).

Determination of Radiochemical yield and Radiochemical Purity:

Following the above procedure, the obtained mixture was diluted with 0.9 mL of water and filtered through a C18-SepPak. **[^{125}I]5ag** was then eluted with 2 mL of methanol and an aliquot was analysed using radio-HPLC. Radiochemical was assessed yield using an activimeter.

Starting Activity (**A**)	H_2O flush activity	MeOH flush activity (**B**)	C18-SepPak + syringe residual activity	Vial residual activity	RCP (**C**)	RCY (**B/A**)***C**
2.65 MBq	0 MBq	2.13 MBq	0.4 MBq	0.1 MBq	100%	80%

Determination of Molar Activity

The injected **[^{125}I]5ag** was below the UV-detection limit and therefore the molar activity was calculated using the lowest detectable concentration of **5ag**.

Lowest detected UV concentration (**D**)	HPLC Injected volume (**E**)	Vial activity (**F**)	Experiment volume(**G**)	Molar Activity (**F*****E**^{2}***C**/100)/(**G*****D**)
2.89 nmol/mL	10 µL	2.13 MBq	2 mL	> 0.368 GBq/µmol

RADIO-CHROMATOGRAMS

In each case, the data are presented in the following order: a radio-chromatogram and a UV-chromatogram of the crude solution are shown, and these are compared to the UV-chromatogram of the cold reference.

Conditions Set-up

Table SS2-entry 1

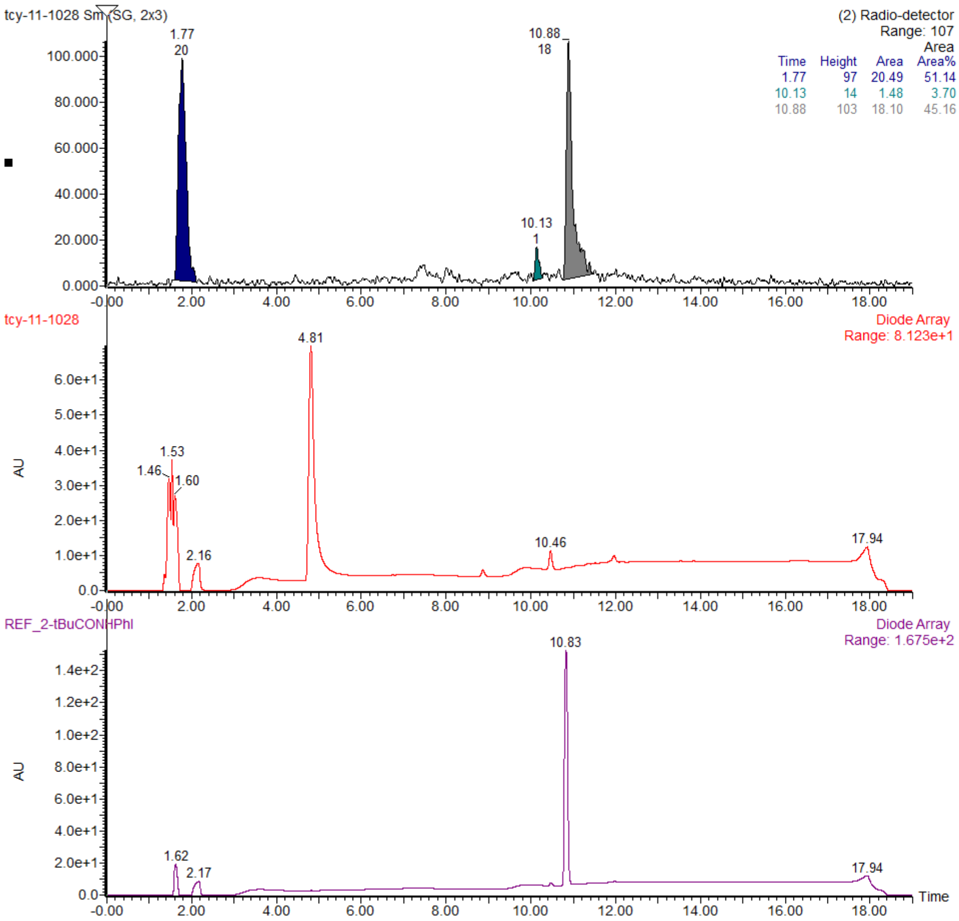

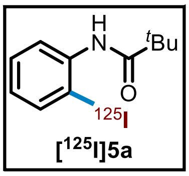

Table S2-entry 2

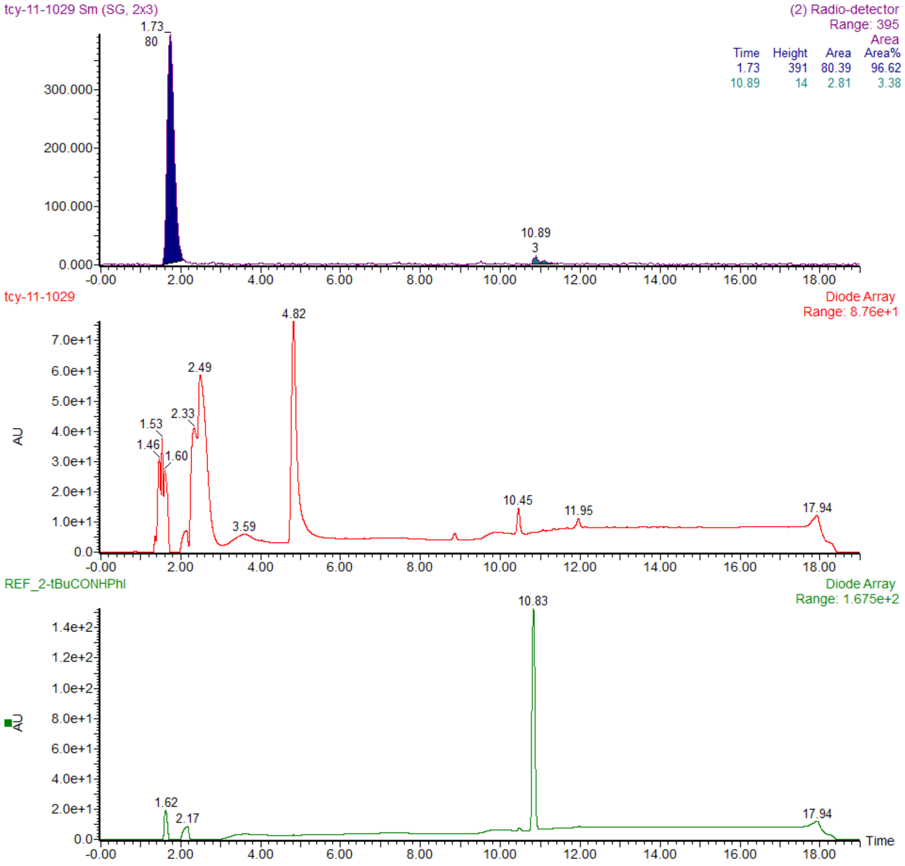

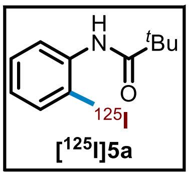

Table S2-entry 3

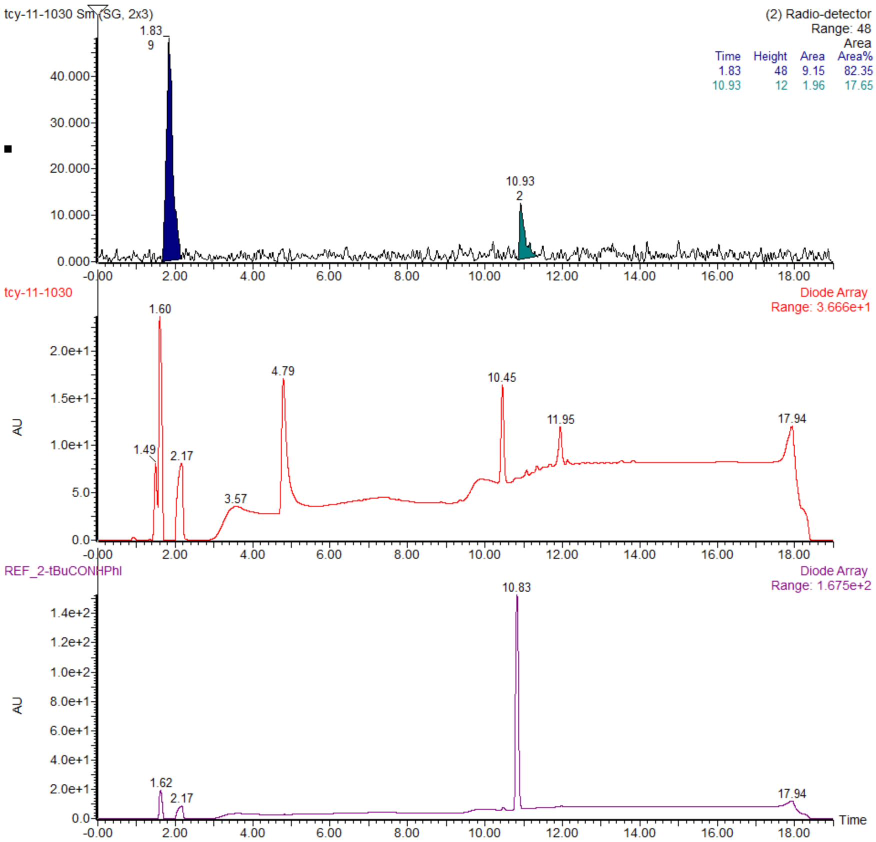

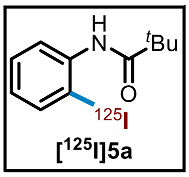

Table S2-entry 4

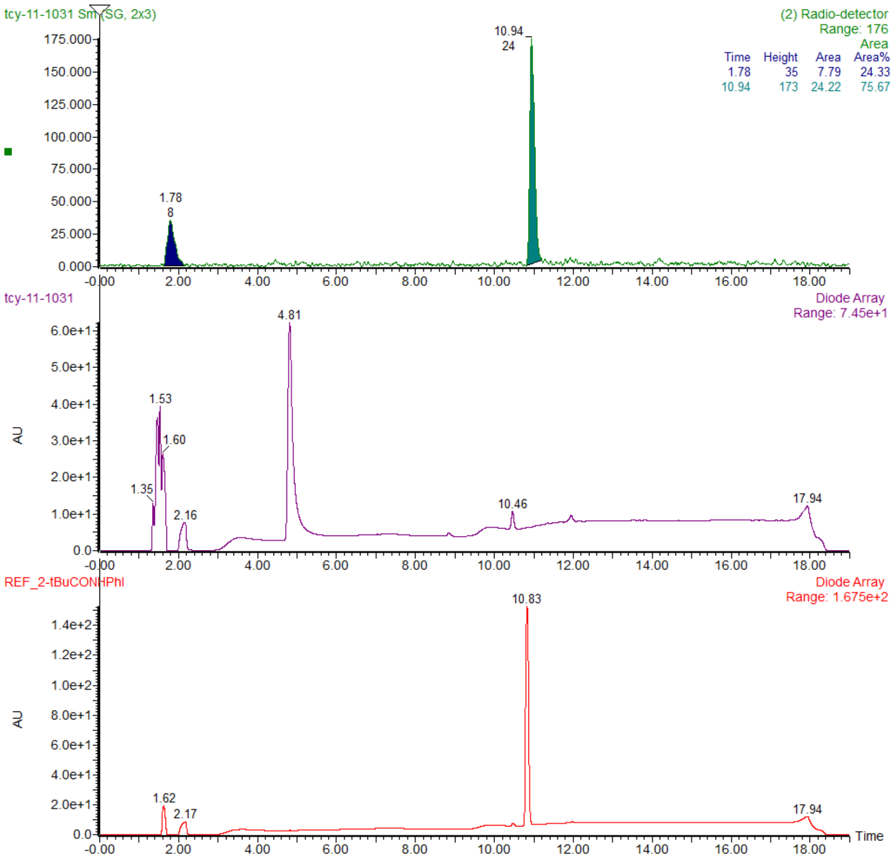

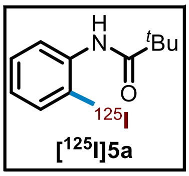

Table S2-entry 5

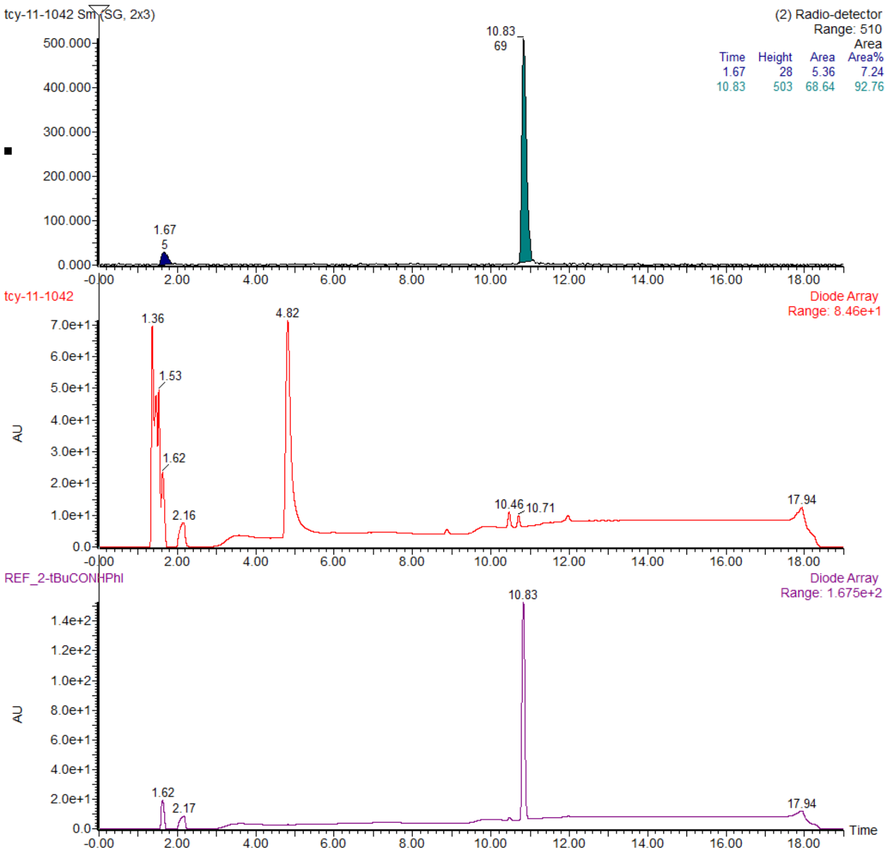

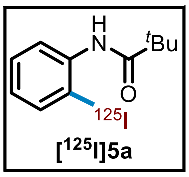

Table S2-entry 6

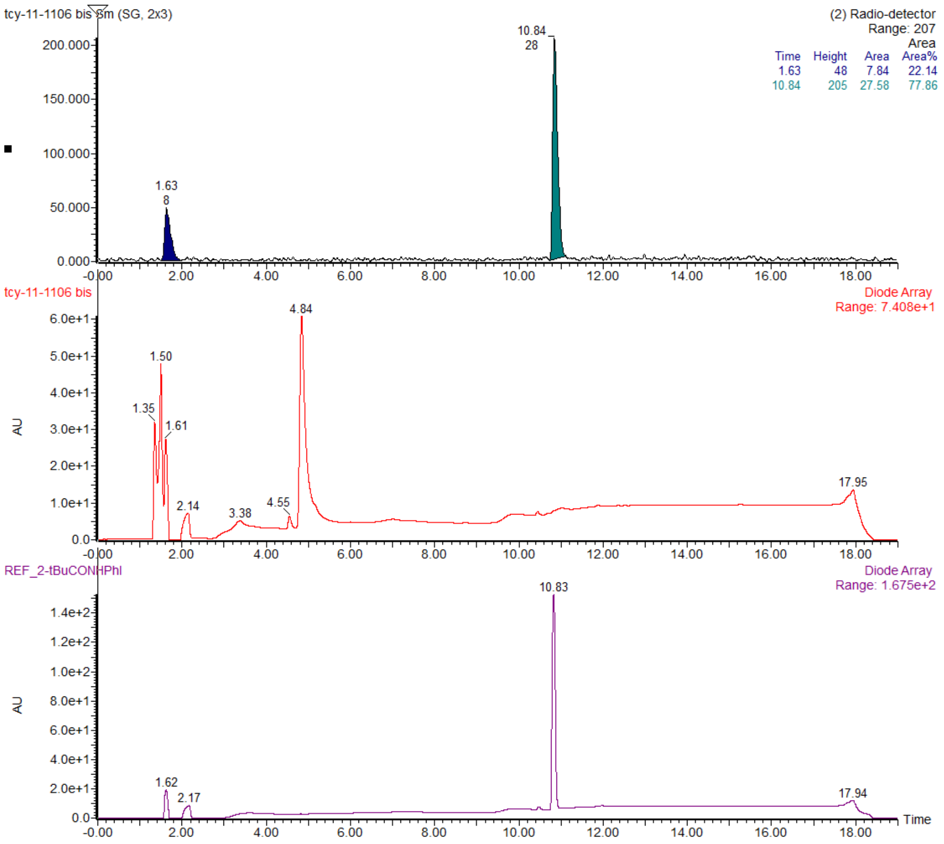

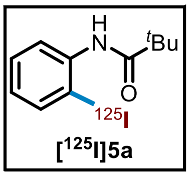

Table S2-entry 7

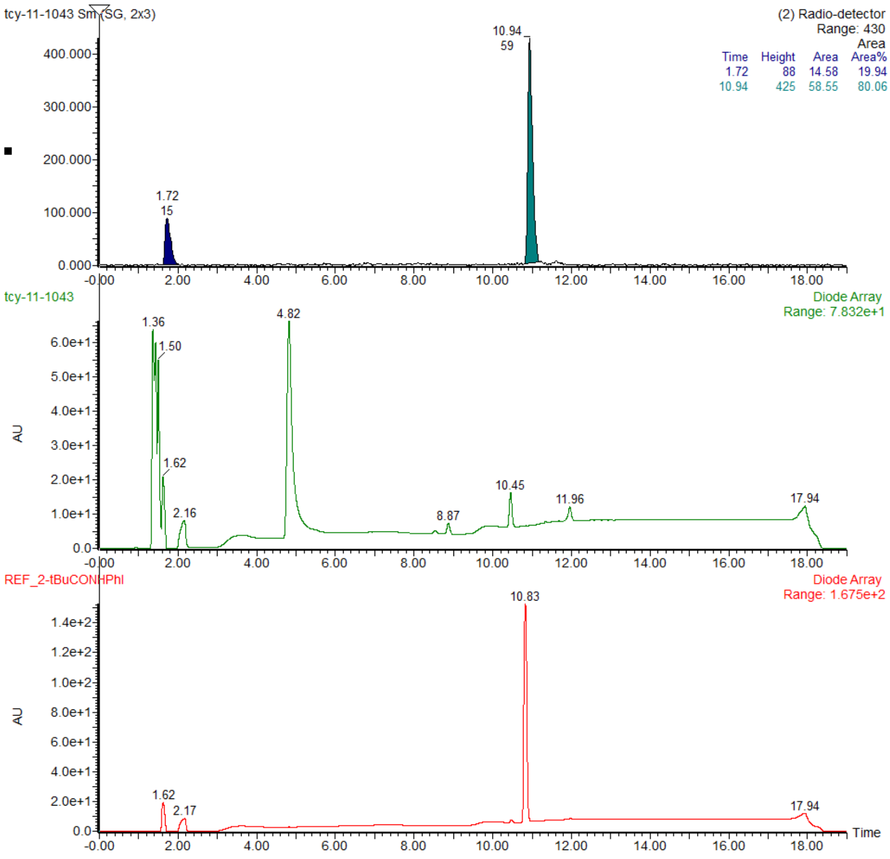

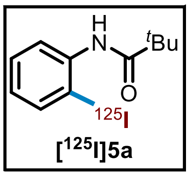

Substrates scope:

[^{125}I]N-(2-Iodophenyl)-2,2-dimethylpropanamide ***[^{125}I]5a***

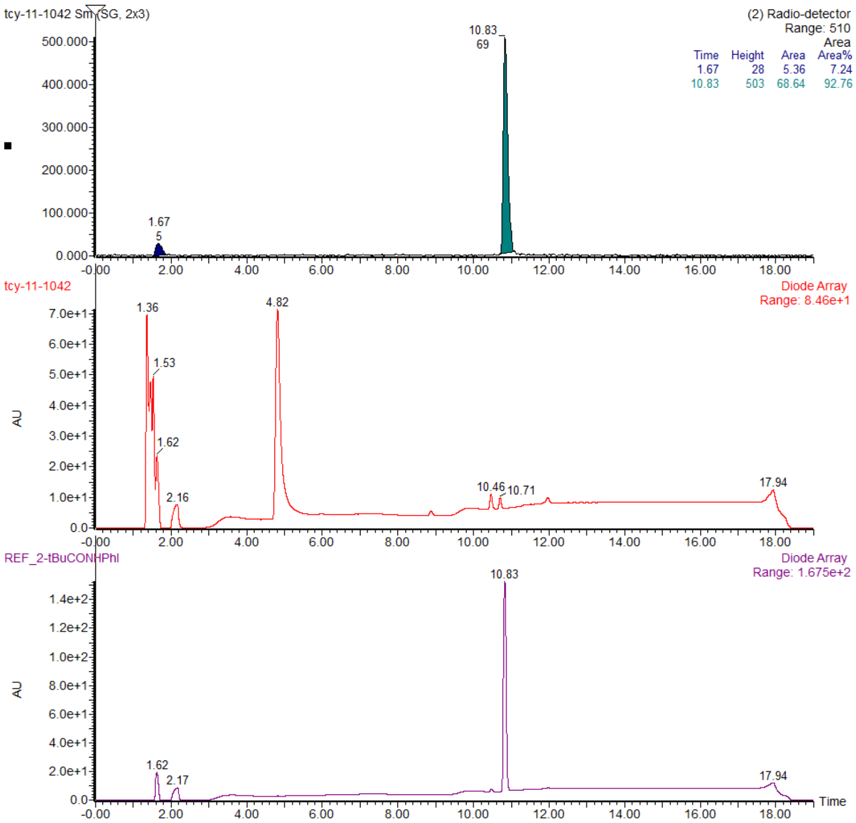

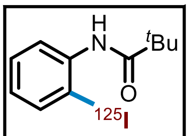

[^{125}I]5a

[^{125}I]N-(4-*tert*Butyl-2-iodophenyl)-2,2-dimethylpropanamide ***[^{125}I]5b***

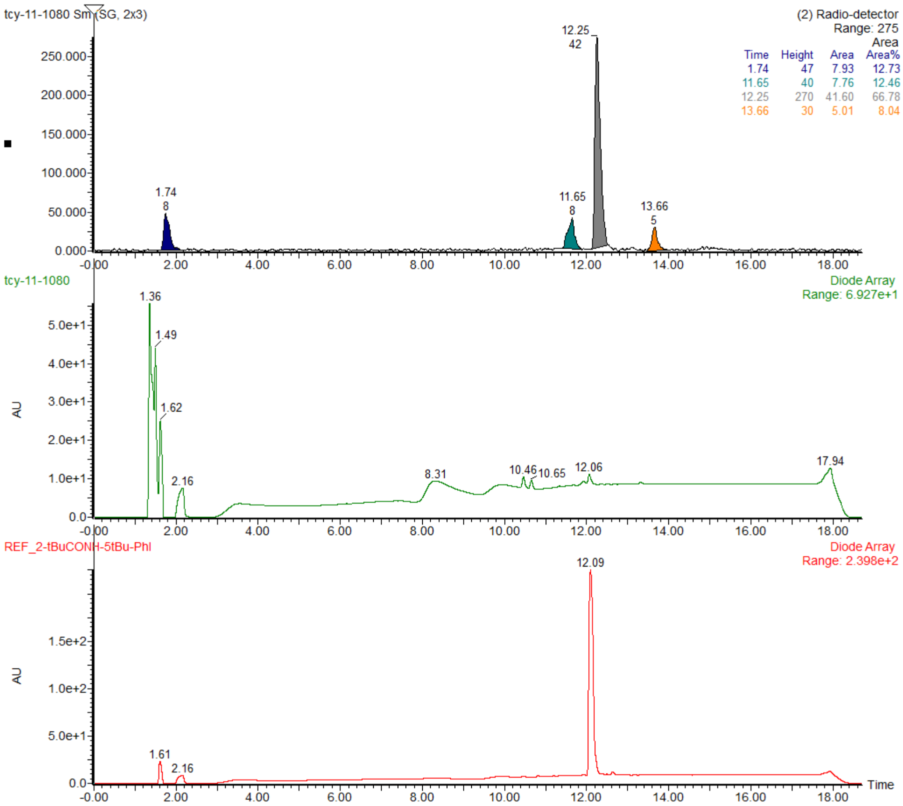

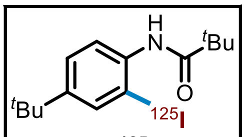

[^{125}I]5b

[^{125}I]N-(4-Fluoro-2-iodophenyl)-2,2-dimethylpropanamide ***[^{125}I]5c***

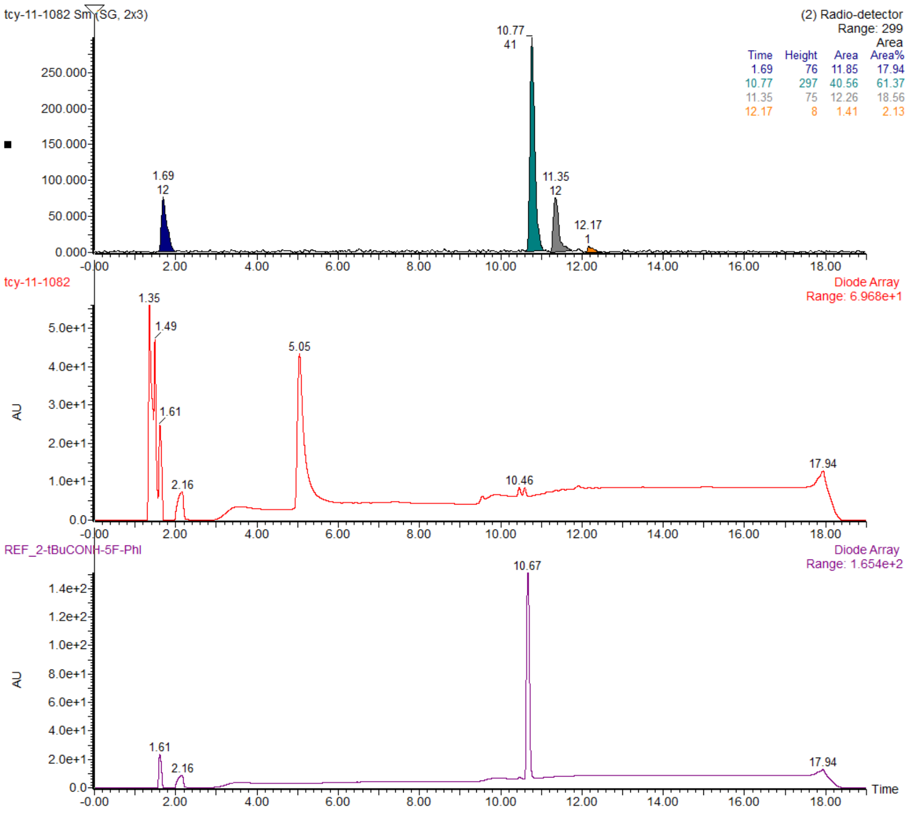

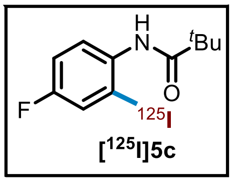

[^{125}I]N-(4-Trityl-2-iodophenyl)-2,2-dimethylpropanamide ***[^{125}I]5d***

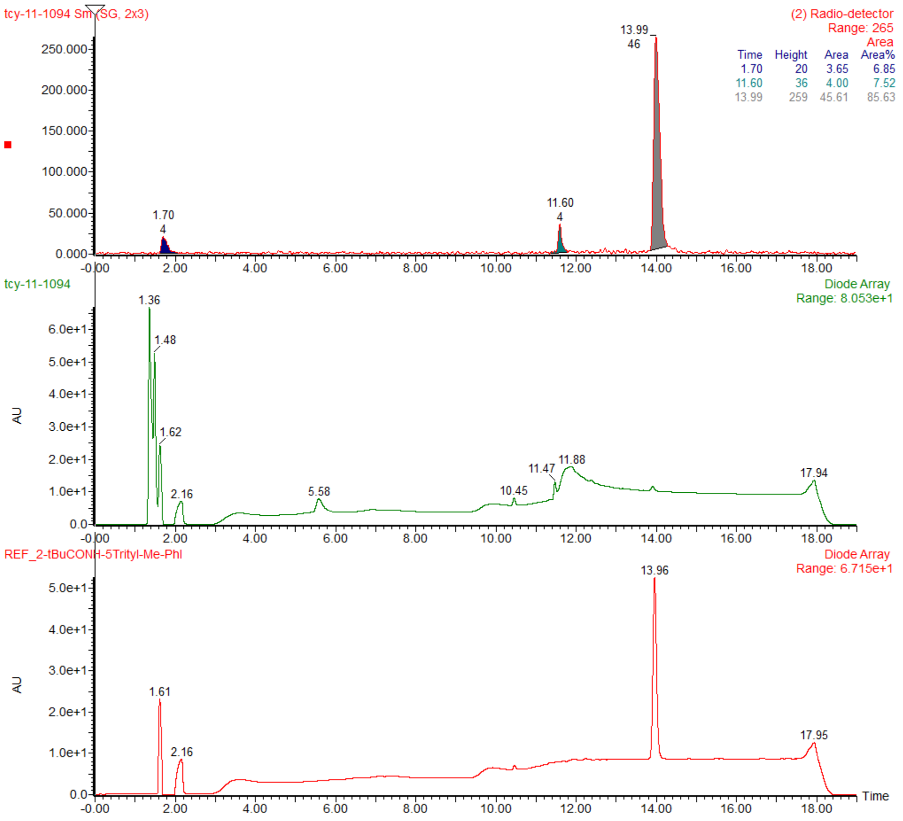

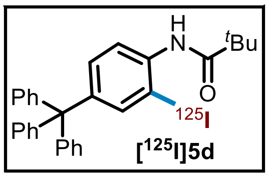

[^{125}I]*N*-(2-Iodo-6-methylphenyl)-2,2-dimethylpropanamide ***[^{125}I]5h***

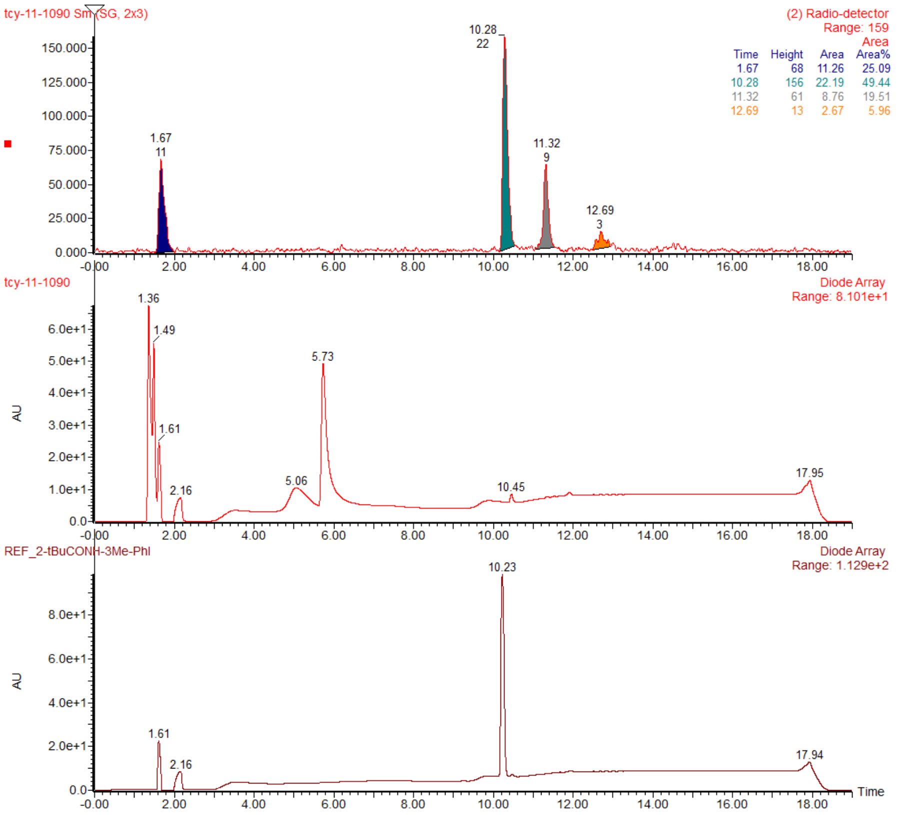

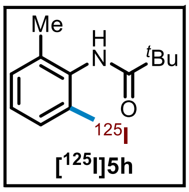

[^{125}I]*N*-(6-Fluoro-2-iodophenyl)-2,2-dimethylpropanamide ***[^{125}I]5i***

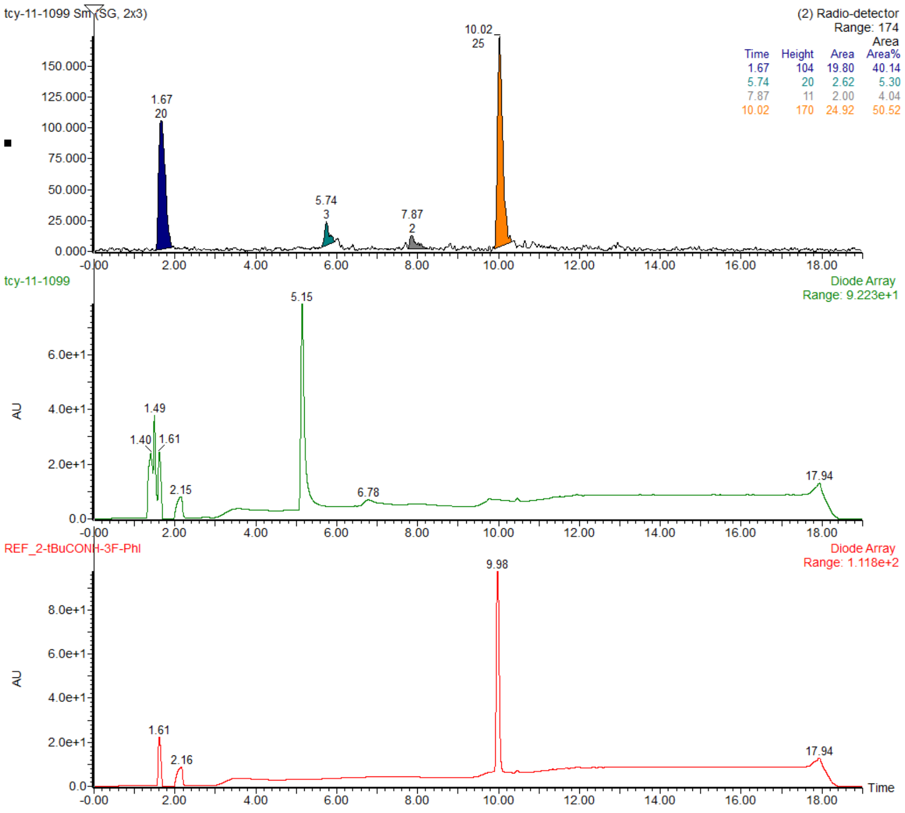

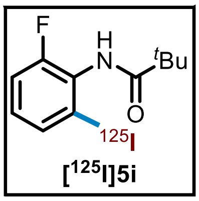

[^{125}I]*N*-(2-Iodo-3,5-dimethylphenyl)-2,2-dimethylpropanamide ***[^{125}I]5j***

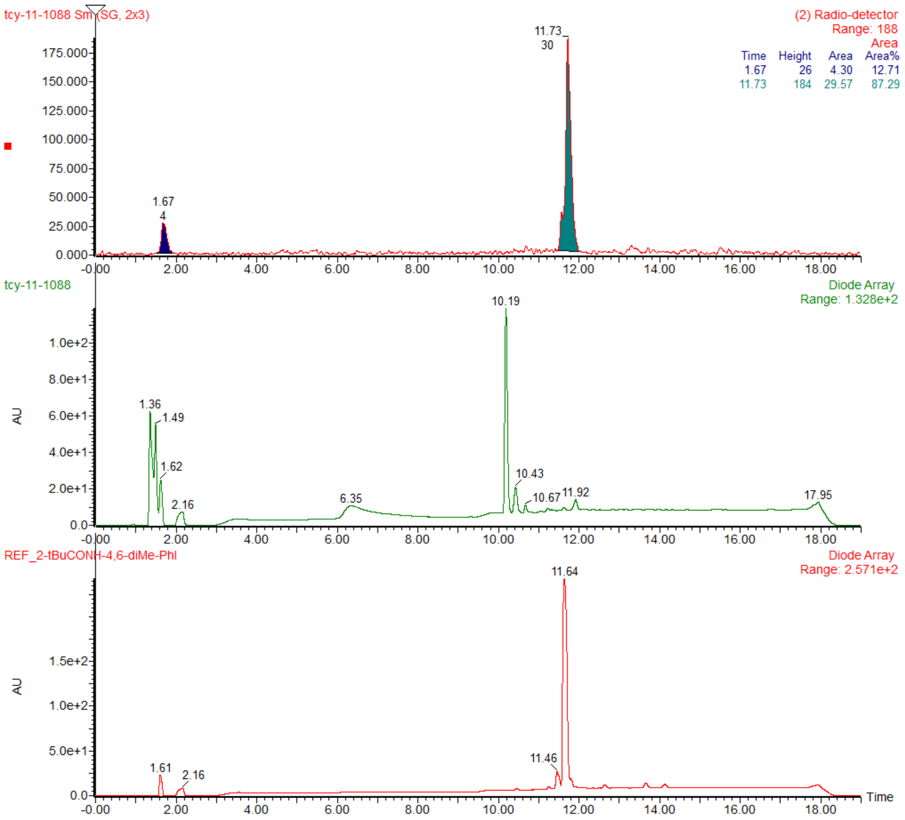

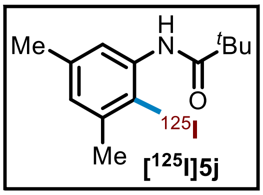

[^{125}I]*N*-(2-Iodophenyl)adamantanecarboxamide ***[^{125}I]5m***

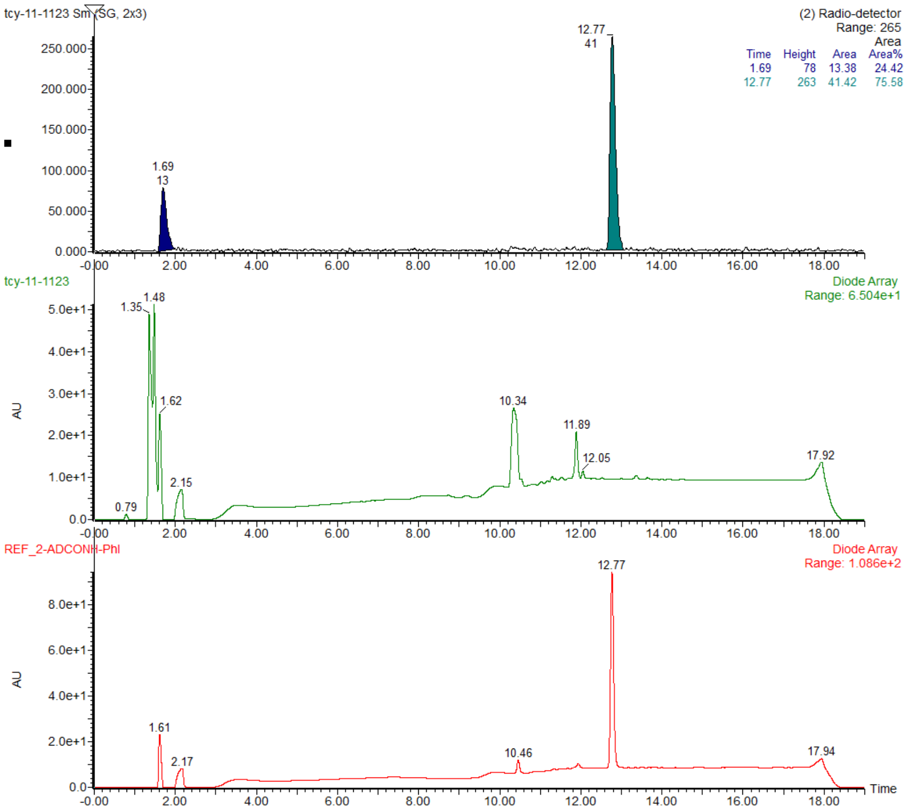

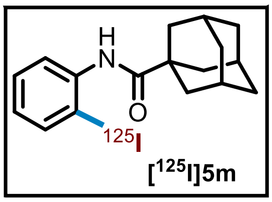

[^{125}I]N-(2-Iodophenyl)benzamide ***[^{125}I]5w***

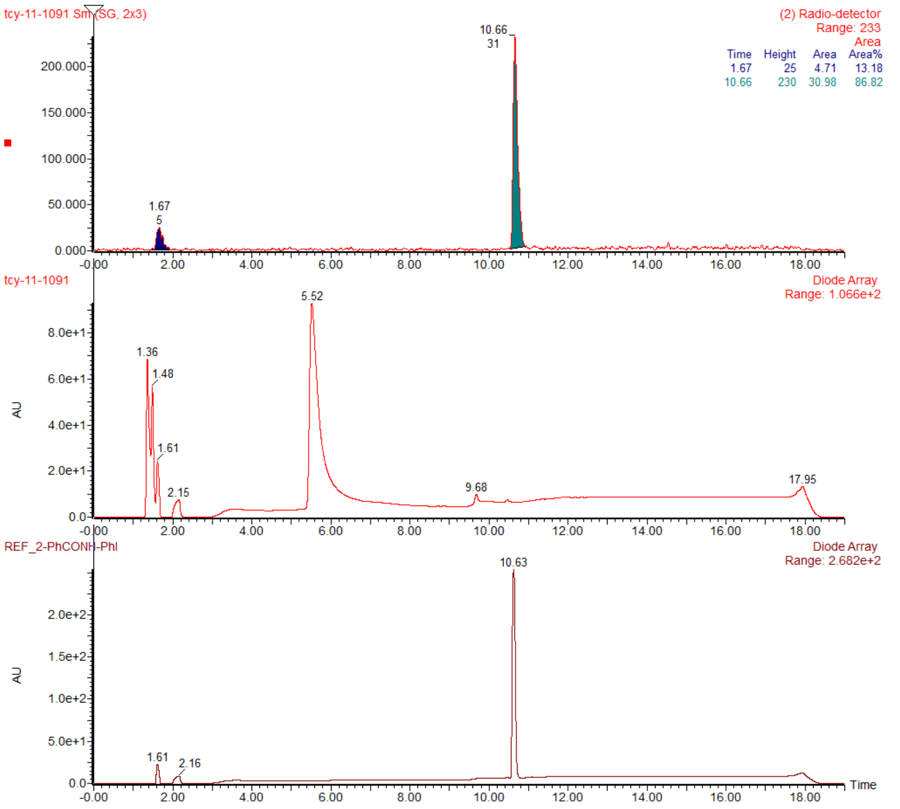

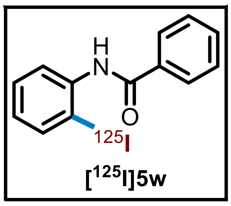

[^{125}I]N-(2-Iodophenyl)-3-methylbenzamide ***[^{125}I]5x***

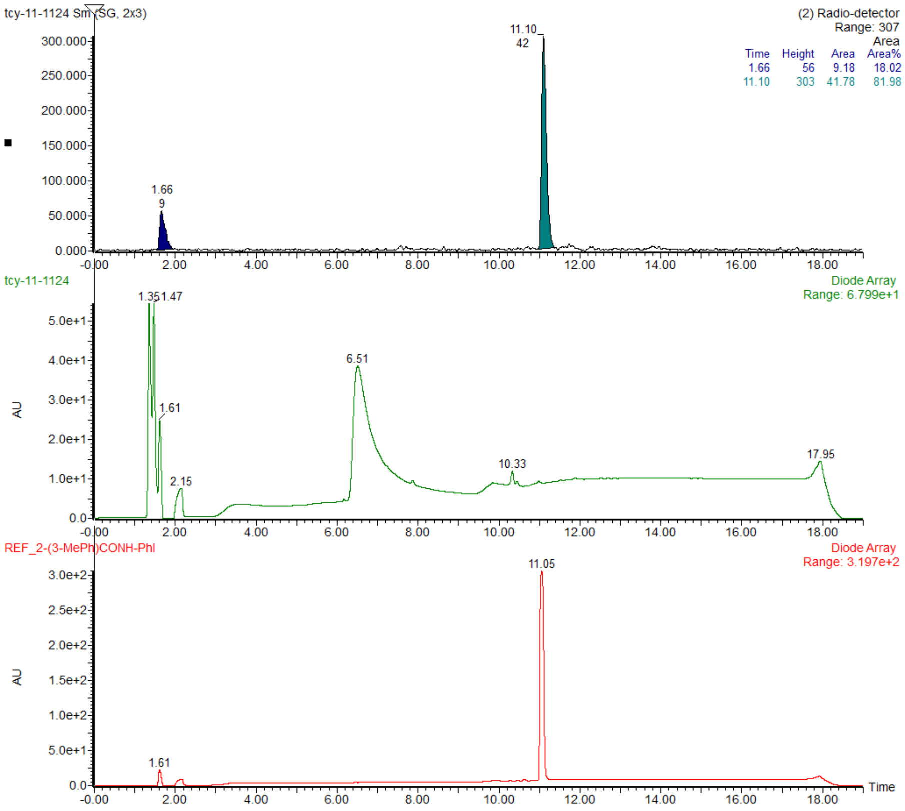

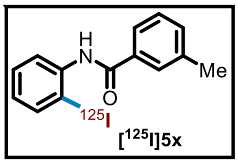

[^{125}I]N-(2-Iodophenyl)-3-nitrobenzamide ***[^{125}I]*5y**

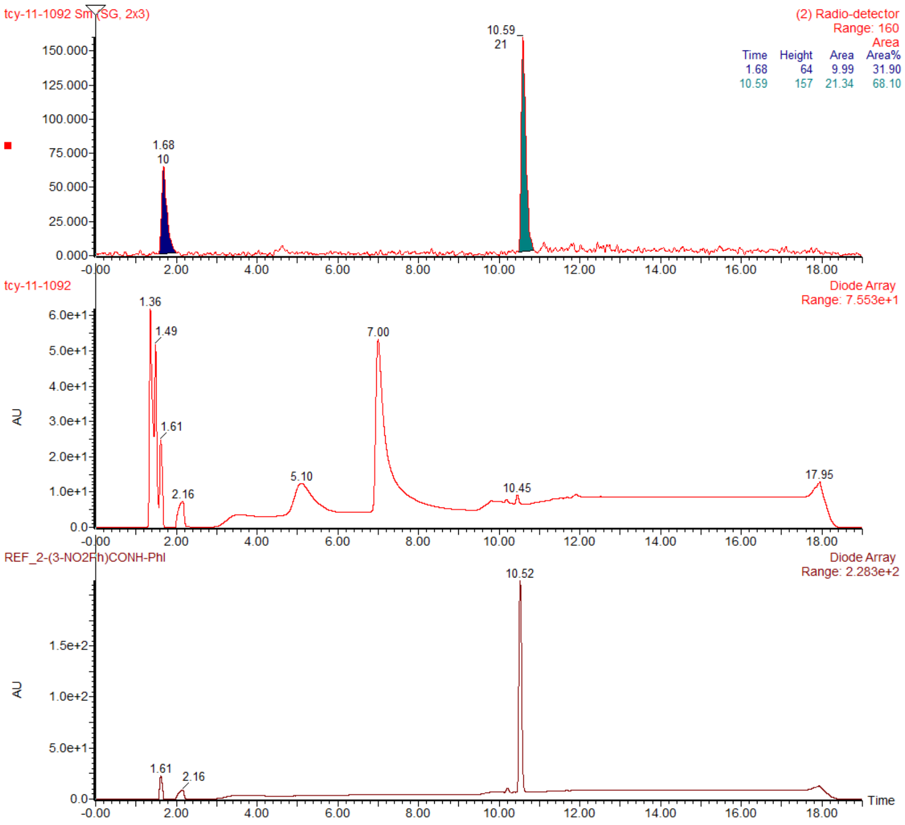

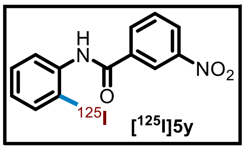

*[^{125}I]*2-(2-Iodophenyl)pyridine ***[^{125}I]*5ah**

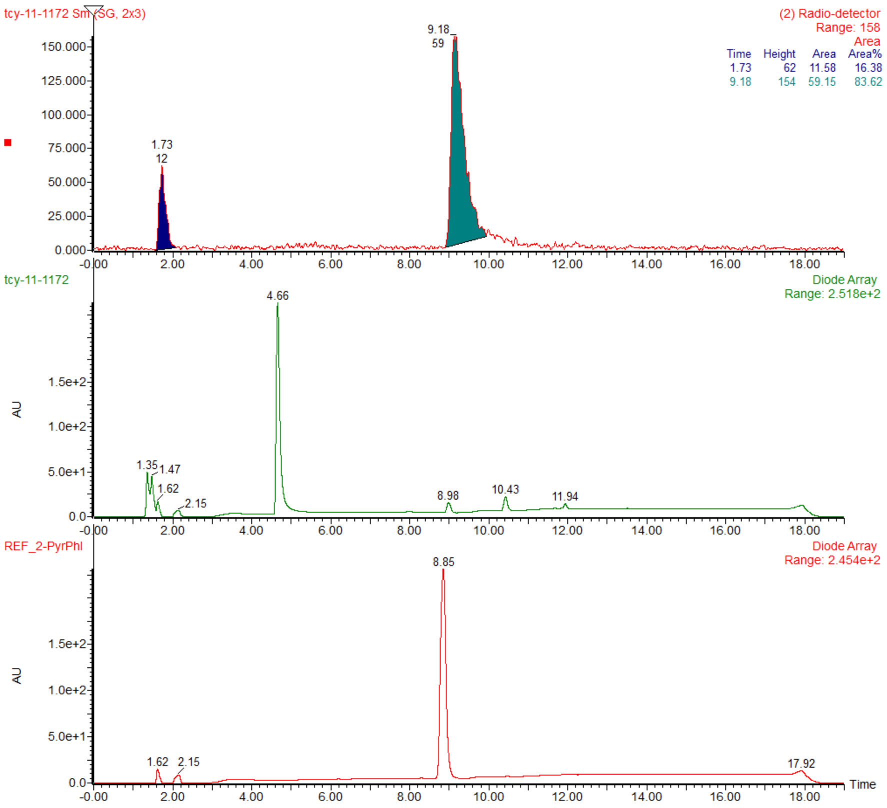

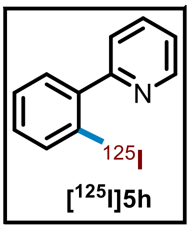

Radioiodination of [^{125}I]5ag

Crude Spectrum

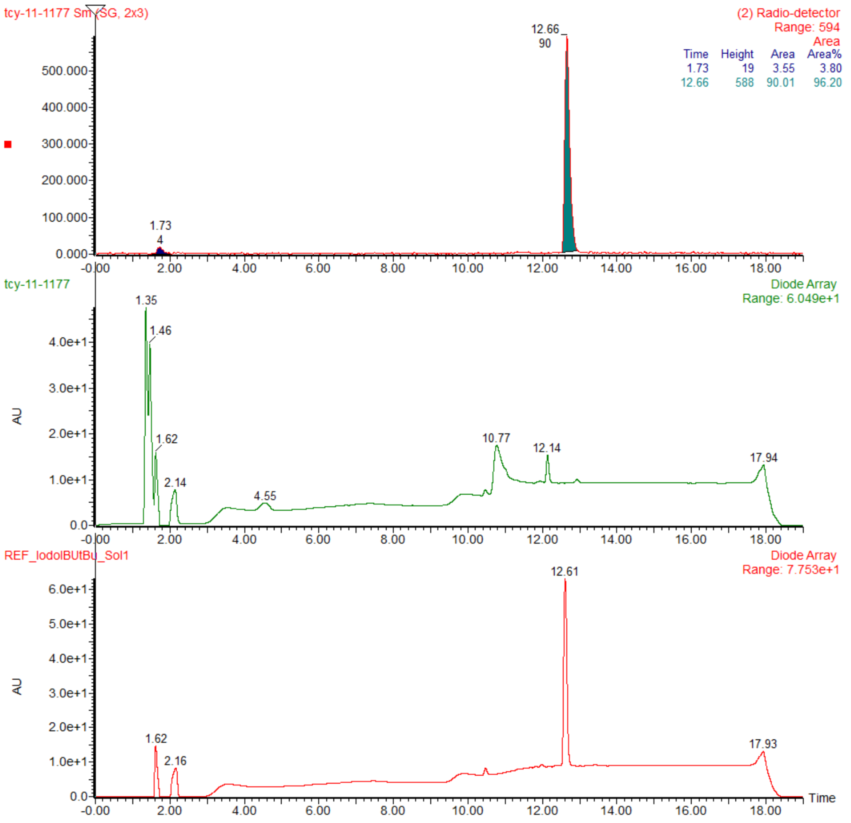

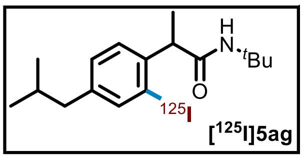

Purified [^{125}I]5ag

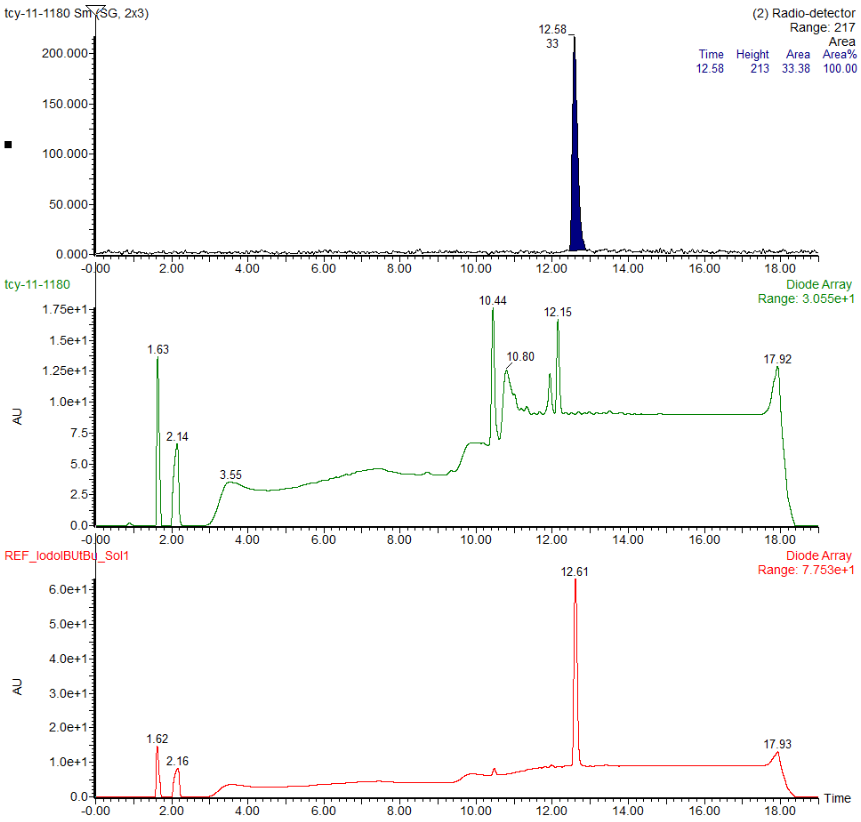

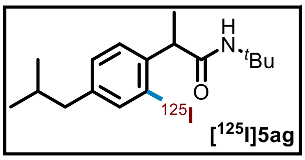

5. Multigram synthesis of Ar−BF$_2$ 3a

General procedure for the synthesis of 3a:

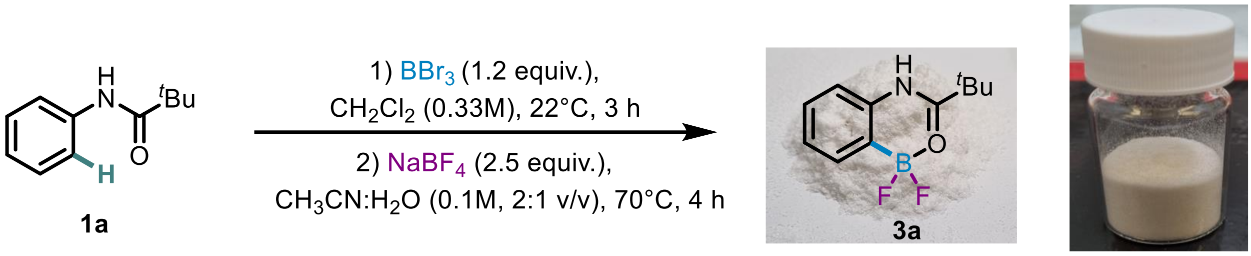

Step i) To a dry 250 mL schlenk flask, equipped with a rubber septum, stir bar, the *N*-phenyl pivalamide (3g, 16.93 mmol, 1 equiv.) in anhydrous CH_2Cl_2 (55 mL) under a nitrogen atmosphere was added dropwise BBr_3 (20.31 ml, 20.31 mmol, 1.2 equiv., 1M solution in CH_2Cl_2). After the complete addition of BBr_3, the reaction mixture was stirred at 22 ^{o}C for 3 h after which the solvent was removed under reduced pressure.

Step ii) To the crude residue from step i) were added $NaBF_4$ (4.65 g, 42.31 mmol, 2.5 equiv.), 113 mL acetonitrile, 56 mL distilled water and the reaction mixture was heated at 70 ^{o}C in a oil bath for 4 h. The reaction was allowed to reach room temperature and the acetonitrile was evaporated under *vacuo* to afford the crude solid. After removal of complete acetonitrile, the crude solid was filtered under *vacuo* and washed with 200 mL distilled water. The crude solid was washed with pentane to afford desired Ar-BF$_2$ product **3a** (off white solid, 3.43 g, 90%).

Reaction was also conducted at 1 g scale and the desired product **3a** was isolated in 93% yield (1.18 g).

Workup and purification protocol for 1g scale: After removal of complete acetonitrile, the crude solid was filtered under *vacuo* and washed with 60 mL distilled water. Then the crude solid was washed with pentane to afford desired Ar-BF$_2$ product **3a**

6. Applications Ar−BF2

6.1 Iodination of 7d:

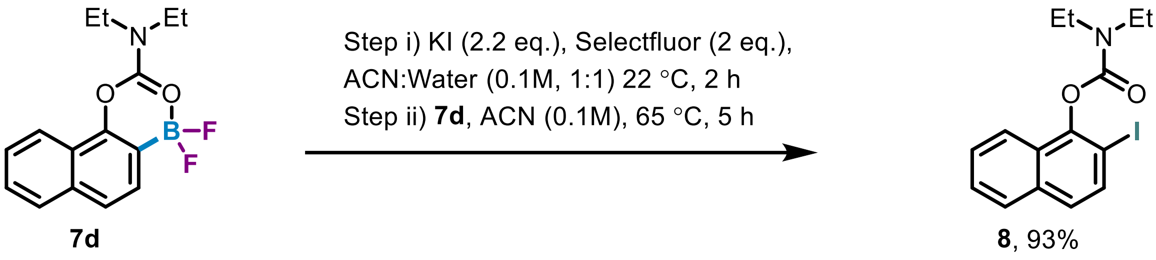

Procedure for iodination (**8**): Step i) To a 3 mL, screw-top V-Vial, (Sigma Aldrich, Product code-Z115150-12EA), equipped witha rubber septum, stir bar, Selectfluor (106.22 mg, 0.3 mmol, 2 equiv.), and KI (54.79 mg, 0.33 mmol, 2.2 equiv.), were dissolved in CH_3CN (1 mL) and water (1 mL) and the reaction mixture stirred at 22 ºCfor 2 h.

Step ii) To a new 5 mL, screw-top V-vial, **7d** (43.67 mg, 0.15 mmol, 1 eq.) was dissolved in CH_3CN (0.5 mL). To this mixture, the solution from step i) was added dropwise at 22 ºC, and the reaction mixture was heated at 60 ºC for 5 h. The reaction was quenched with saturated sodium thiosulfite solution at room temperature and the crude mixture was dissolved in EtOAc (20 mL) and H_2O (10 mL). The aqueous layer was washed with EtOAc (10 mL), and the combined organic layer was washed with brine, dried over sodium sulfate, filtered, and evaporated in vacuo to afford the crude product, which was purified using automated column chromatography (90:10, pentane/EtOAc).

Compound 8 spectral data:[15] Beige color solid, 51.4 mg (93%); **^{1}H NMR (800 MHz, $CDCl_3$) δ=** 7.84 – 7.82 (m, 2H), 7.80 (d, J = 8.6 Hz, 1H), 7.52 – 7.49 (m, 2H), 7.47 (d, J = 8.6 Hz, 1H), 3.79 – 3.58 (m, 2H), 3.55 – 3.40 (m, 2H), 1.45 (t, J = 7.1 Hz, 3H), 1.28 (t, J = 7.1 Hz, 3H); **^{13}C{^{1}H} NMR (201 MHz, $CDCl_3$) δ=** 152.8, 148.6, 134.8, 134.4, 128.8, 128.0, 127.2, 127.1, 126.9, 121.8, 88.8, 42.7, 42.4, 14.8, 13.6.

6.2 Suzuki-Miyuara cross coupling of 7d:

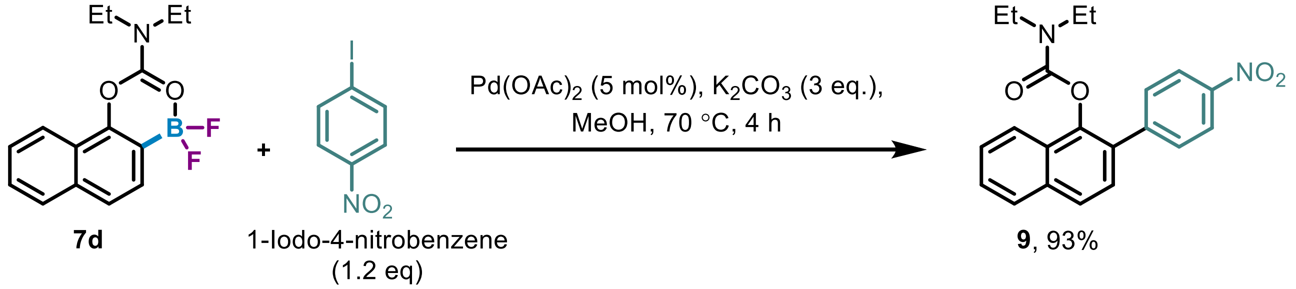

Procedure for cross-coupling (**9**): To a dry 5 mL, screw-top V-vial, **7d** (43.67 mg, 0.15 mmol, 1 eq.) were added K_2CO_3 (62.2 mg, 0.45 mmol, 3 equiv.) and palladium acetate (1.68 mg, 5 mol%, 0.01 equiv.) under

nitrogen atmosphere. To this, 1.5 mL degassed methanol was added along with 1-iodo-4-nitrobenzene (44.83 mg, 0.18 mmol, 1.2 equiv.) and the reaction mixture was heated at 70 ºC for 4 h. The reaction was allowed to reach room temperature and diluted with 2 mL ethyl acetate and filtered through a pad of celite. The celite was washed with additional 15 mL of ethyl acetate and the filtrate was evaporated in vacuo to afford the crude product, which was purified using automated column chromatography (88:12, pentane/EtOAc).

Compound 9 spectral data: Yellow solid, 50.7 mg (93%); **Rf:** 0.66 (hexane/EtOAc, 70:30); **^{1}H NMR (800 MHz, CDCl$_3$) δ=** 8.29 (d, *J* = 8.3 Hz, 2H), 7.91 (dd, *J* = 8.1, 3.2 Hz, 2H), 7.83 (d, *J* = 8.4 Hz, 1H), 7.71 (d, *J* = 8.3 Hz, 2H), 7.62 – 7.53 (m, 2H), 7.47 (d, *J* = 8.3 Hz, 1H), 3.45 (q, *J* = 7.1 Hz, 2H), 3.28 (q, *J* = 7.1 Hz, 2H), 1.21 (t, *J* = 7.1 Hz, 3H), 1.04 (t, *J* = 7.1 Hz, 3H); **^{13}C{^{1}H} NMR (201 MHz, CDCl$_3$) δ=** 153.6, 147.2, 145.6, 144.4, 134.7, 130.4, 129.5, 128.2, 128.2, 127.3, 127.1, 127.0, 126.3, 123.6, 122.0, 42.4, 42.1, 14.5, 13.3; **HRMS (ESI) (m/z):** calculated for $[M+H]^+$ $C_{21}H_{21}N_2O_4^+$ 365.14958; found 365.14902.

6.3 Halogenation of 3a:

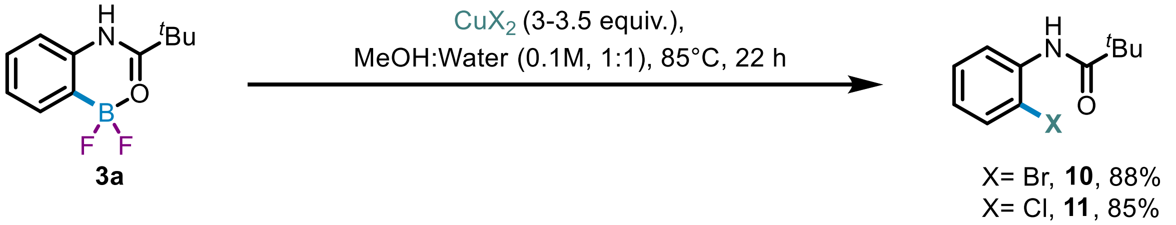

Procedure for bromination (**10**): To a dry 5 mL V vial, equipped with a rubber septum, stir bar, the Ar-BF$_2$ **3a** (45.01 mg, 0.2 mmol, 1 equiv.) in 1 mL MeOH and 1 mL distilled water was added Copper(II) bromide (156.35 mg, 0.7 mmol, 3.5 equiv.). The reaction mixture was heated at 85 ºC for 22 h. The reaction was allowed to reach room temperature and diluted with 2 mL ethyl acetate and filtered through a pad of celite and sodium sulfate. The celite was washed with 30 mL ethyl acetate and the filtrate was concentrated *in vacuo* to afford the crude product, which was purified using silica gel on automated column chromatography (pentane:EtOAc, 90:10).

Procedure for chlorination (**11**): Same as bromination procedure. Copper(II) chloride (80.67 mg, 0.6 mmol, 3 equiv.) was used for chlorination.

Compound 10 spectral data:[2] Beige color solid, 45 mg (88%); **^{1}H NMR (600 MHz, CDCl$_3$) δ=** 8.39 (dd, *J* = 8.3, 1.6 Hz, 1H), 8.01 (bs, 1H), 7.52 (dd, *J* = 8.0, 1.5 Hz, 1H), 7.31 (ddd, *J* = 8.6, 7.1, 1.5 Hz, 1H), 6.96 (ddd, *J* = 8.1, 7.4, 1.6 Hz, 1H), 1.35 (s, 9H); **^{13}C{^{1}H} NMR (151 MHz, CDCl$_3$) δ=** 176.8, 136.0, 132.2, 128.5, 125.0, 121.8, 113.8, 40.4, 27.7.

Compound 11 spectral data:[2] Off white solid, 36 mg (85%); **^{1}H NMR (600 MHz, CDCl$_3$) δ=** 8.41 (dd, *J* = 8.3, 1.6 Hz, 1H), 8.01 (bs, 1H), 7.35 (dd, *J* = 8.1, 1.5 Hz, 1H), 7.26 (ddd, *J* = 8.3, 7.0, 1.5 Hz, 1H),

7.02 (td, J = 7.7, 1.6 Hz, 1H), 1.34 (s, 9H); **$^{13}C\{^1H\}$ NMR (151 MHz, $CDCl_3$) δ=** 176.8, 134.9, 129.0, 127.9, 124.5, 123.0, 121.5, 40.3, 27.7.

6.4 Metal-free iodination of Ibuprfen analogue:

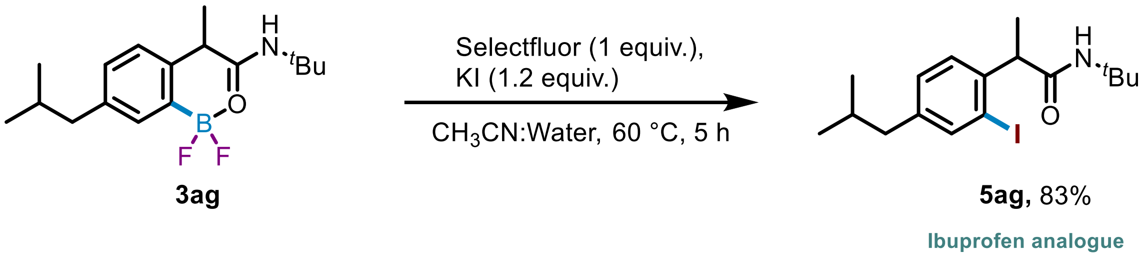

Step i) To a dry 3 mL, screw-top V-Vial, equipped with a rubber septum, stir bar, the Selectfluor (35.43 mg, 0.1 mmol, 1 equiv.), and KI (19.92 mg, 0.12 mmol, 1.2 equiv.), were dissolved in CH_3CN (1 mL) and water (0.5 mL) and the mixture stirred at 22 ºC for 2 h.

Step iii) The Ar-BF_2 **3ag** (30.92 mg, 0.1 mmol, 1 equiv.), was added to the above mixture at 22 ºC and the reaction was heated at 60 ºC, for 5 h. The reaction was quenched with saturated sodium thiosulfate solution at room temperature and the crude mixture was dissolved in EtOAc (10 mL) and H_2O (10 mL). The aqueous layer was washed with EtOAc (10 mL), and the combined organic layer was washed with brine, dried over sodium sulfate, filtered, and evaporated in vacuo to afford the crude product, which was purified using automated column chromatography (pentane/EtOAc, 90:10).

Compound 5ag spectral data: Off white solid, 32 mg (83%); **^1H NMR (600 MHz, $CDCl_3$) δ=** 7.63 (d, J = 1.8 Hz, 1H), 7.27 (d, J = 8.0 Hz, 1H), 7.11 (dd, J = 7.9, 1.8 Hz, 1H), 5.23 (bs, 1H), 3.75 (q, J = 7.0 Hz, 1H), 2.39 (d, J = 7.2 Hz, 2H), 1.84 (dh, J = 13.5, 6.7 Hz, 1H), 1.42 (d, J = 7.1 Hz, 3H), 1.27 (s, 9H), 0.89 (d, J = 6.6 Hz, 6H); **$^{13}C\{^1H\}$ NMR (151 MHz, $CDCl_3$) δ=** 172.7, 142.8, 141.7, 140.1, 130.0, 127.3, 101.3, 51.3, 50.9, 44.4, 30.2, 28.8, 22.4, 18.1; **HRMS (ESI) (m/z):** calculated for $[M+H]^+$ $C_{17}H_{27}INO^+$ 388.1131; found 388.1177.

6.5 Azidation of 3a:

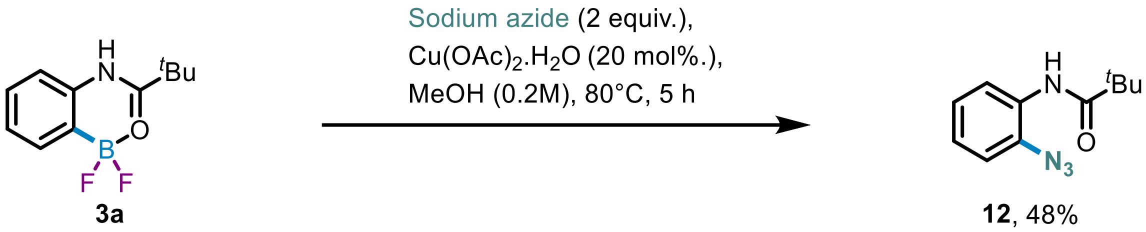

Procedure for azidation (**12**): To a dry 5 mL V vial, equipped with a rubber septum, stir bar, the Ar-BF_2-**3a** (45.01 mg, 0.2 mmol, 1 equiv.), in 1 mL methanol were added sodium azide (26 mg, 0.4 mmol, 2 equiv.), and $Cu(OAc)_2.H_2O$ (7.99 mg, 0.04 mmol, 0.2 equiv.). The reaction mixture was heated at 80 ºC for 5 h. The reaction was allowed to reach room temperature and diluted with 2 mL ethyl acetate and

filtered through a pad of celite. The celite was washed with 30 mL ethyl acetate and the filtrate was concentrated *in vacuo* to afford the crude product, which was purified using silica gel on automated column chromatography (pentane:EtOAc solvent, 90:10).

Compound 12 spectral data:[4] Yellow oil, 21 mg (48%); **^{1}H NMR (800 MHz, $CDCl_3$) δ=** 8.39 (d, J = 8.0 Hz, 1H), 7.85 (s, 1H), 7.15 – 7.10 (m, 3H), 1.32 (s, 9H); **^{13}C{^{1}H} NMR (201 MHz, $CDCl_3$) δ=** 176.7, 129.9, 128.0, 125.8, 124.2, 120.9, 117.6, 40.2, 27.7.

6.6 Hydroxylation of 3a:

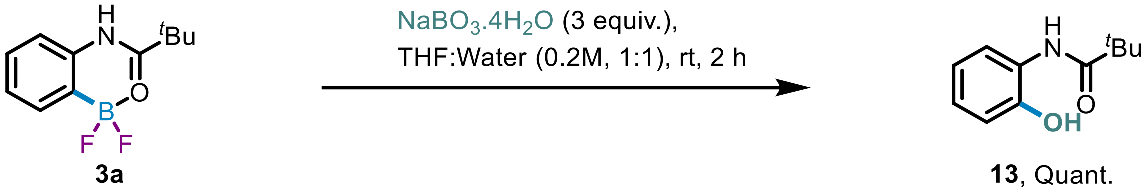

Procedure for hydroxylation (**13**): To a dry 5 mL V vial, equipped with a rubber septum, stir bar, the Ar-BF_2 **3a** (45.01 mg, 0.2 mmol, 1 equiv.) in 0.5 mL THF and 0.5 mL water was added $NaBO_3.4H_2O$ (92.32 mg, 0.6 mmol, 3 equiv.). The mixture was stirred at room temperature for 2 h. The reaction mixture dissolved in 30 mL ethyl acetate and washed with 20 mL water, followed by 20 mL brine solution. The organic layer was separated and dried using sodium sulfate, filtered and concentrated *in vacuo* to afford the pure product in quantitative yield.

Compound 13 spectral data:[5] Off white solid, **^{1}H NMR (800 MHz, $CDCl_3$) δ=** 8.80 (bs, 1H), 7.61 (bs, 1H), 7.12 (t, J = 7.7 Hz, 1H), 7.05 – 7.00 (m, 2H), 6.86 (t, J = 7.6 Hz, 1H), 1.36 (s, 9H); **^{13}C{^{1}H} NMR (201 MHz, $CDCl_3$) δ=** 179.1, 149.1, 127.3, 125.7, 122.3, 120.5, 120.1, 39.6, 27.8.

6.7 Suzuki-Miyuara cross coupling (Csp^2-Csp^2 and Csp^2-Csp^3) of 3a:

6.7.1 Csp^2-Csp^2:

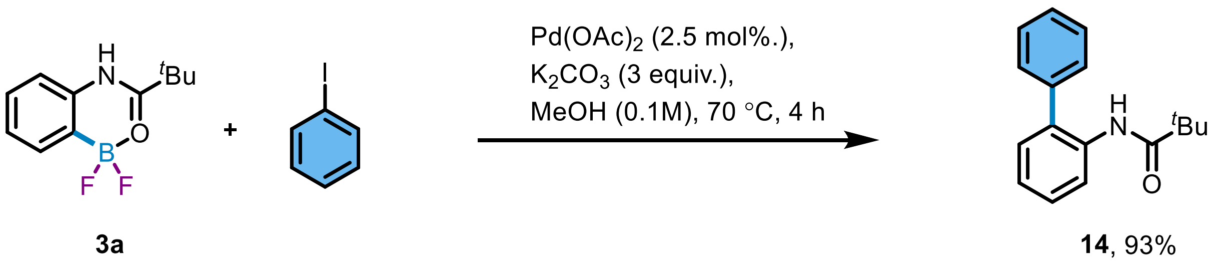

Procedure for arylation (**14**): To a dry 5 mL V vial, equipped with a rubber septum, stir bar, the Ar-BF_2 **3a** (45.01 mg, 0.2 mmol, 1 equiv.), K_2CO_3 (82.93 mg, 0.6 mmol, 3 equiv.), $Pd(OAc)_2$ (1.12 mg, 0.005 mmol, 2.5 mol%) was added 2 mL degassed methanol along with iodobenzene (48.96 mg, 0.24 mmol, 1.2 equiv.) under nitrogen atmosphere and the reaction mixture was heated at 70 °C for 4 h. The reaction

was allowed to reach room temperature and diluted with 2 mL ethyl acetate and filtered through a pad of celite. The celite was washed with 30 mL ethyl acetate and the filtrate was concentrated *in vacuo* to afford the crude product, which was purified using silica gel on automated column chromatography (pentane:EtOAc solvent, 90:10).

Compound 14 spectral data:[7] Off white solid, 46.9 mg (93%); **^{1}H NMR (600 MHz, $CDCl_3$) δ=** 8.37 (dd, *J* = 8.3, 1.2 Hz, 1H), 7.52 – 7.48 (m, 2H), 7.45 (bs, 1H), 7.44 – 7.40 (m, 1H), 7.38 – 7.35 (m, 3H), 7.24 (dd, *J* = 7.6, 1.7 Hz, 1H), 7.16 (td, *J* = 7.5, 1.2 Hz, 1H), 1.09 (s, 9H); **^{13}C{^{1}H} NMR (151 MHz, $CDCl_3$) δ=** 176.4, 138.3, 135.3, 132.3, 129.9, 129.5, 129.2, 128.6, 128.2, 124.0, 121.0, 39.9, 27.5.

6.7.2 Csp^2-Csp^3:

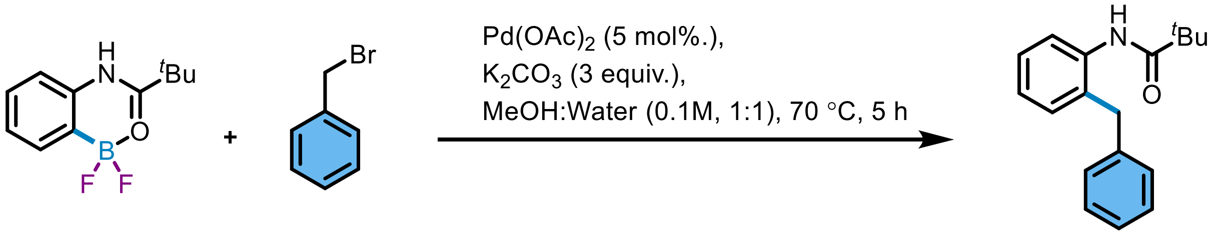

3a **15**, 94%

Procedure for benzylation (**15**): To a dry 5 mL V vial, equipped with a rubber septum, stir bar, the Ar-BF_2 **3a** (45.01 mg, 0.2 mmol, 1 equiv.), K_2CO_3 (82.93 mg, 0.6 mmol, 3 equiv.), $Pd(OAc)_2$ (2.25 mg, 0.01 mmol, 5 mol%) was added 1 mL degassed methanol along with benzyl bromide (51.31 mg, 0.3 mmol, 1.5 equiv.) and 1 mL degassed distilled water under nitrogen atmosphere and the reaction mixture was heated at 70 ºC for 5 h. The reaction was allowed to reach room temperature and diluted with 2 mL ethyl acetate and filtered through a pad of celite and sodium sulfate. The celite was washed with 30 mL ethyl acetate and the filtrate was concentrated *in vacuo* to afford the crude product, which was purified using silica gel on automated column chromatography (pentane:EtOAc solvent, 93:07).

Compound 15 spectral data:[8] Off white solid, 50.1 mg (94%), **^{1}H NMR (600 MHz, $CDCl_3$) δ=** 7.99 (dd, *J* = 8.2, 1.3 Hz, 1H), 7.33 – 7.29 (m, 3H), 7.26 – 7.22 (m, 2H), 7.16 – 7.12 (m, 3H), 7.04 (bs, 1H), 4.02 (s, 2H), 1.06 (s, 9H); **^{13}C{^{1}H} NMR (151 MHz, $CDCl_3$) δ=** 176.6, 138.8, 136.4, 131.2, 130.4, 129.1, 128.3, 127.8, 127.0, 124.9, 123.5, 39.6, 38.7, 27.5.

6.8 Dimerization of 3a:

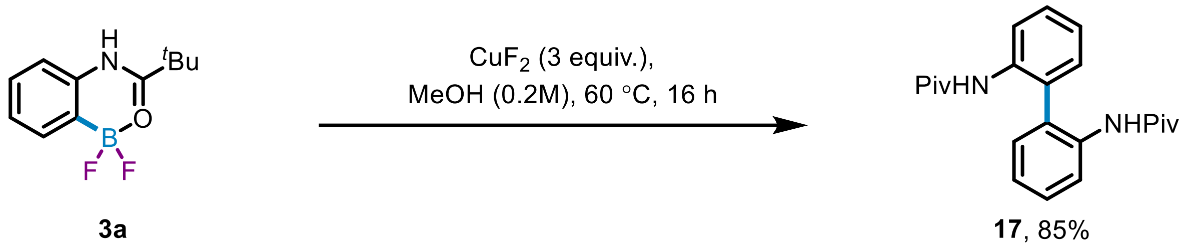

Procedure for homocoupling (**17**): To a dry 5 mL V vial, equipped with a rubber septum, stir bar, added the Ar-BF_2 **3a** (45.01 mg, 0.2 mmol, 1 equiv.) in anhydrous methanol (1 mL) under nitrogen atmosphere was added CuF_2 (60.93 mg, 0.6 mmol, 3 equiv.). The reaction mixture was stirred at 60 ºC for 16 h. The reaction was allowed to reach room temperature and diluted with 2 mL ethyl acetate and filtered through a pad of celite. The celite was washed with 30 mL ethyl acetate and the filtrate was concentrated *in vacuo* to afford the crude product, which was purified using silica gel on automated column chromatography (pentane:EtOAc solvent, 85:15).

Compound 17 spectral data:[6] Off white solid, 29.96 mg (85%); **^{1}H NMR (600 MHz, $CDCl_3$) δ=** 8.31 (d, *J* = 8.2 Hz, 2H), 7.49 – 7.41 (m, 2H), 7.25 – 7.22 (m, 4H), 7.17 (bs, 2H), 1.00 (s, 18H); **^{13}C{^{1}H} NMR (151 MHz, $CDCl_3$) δ=** 176.9, 136.2, 129.9, 129.7, 128.2, 124.7, 122.0, 39.8, 27.3.

7. Stability study of Ar−BF₂s

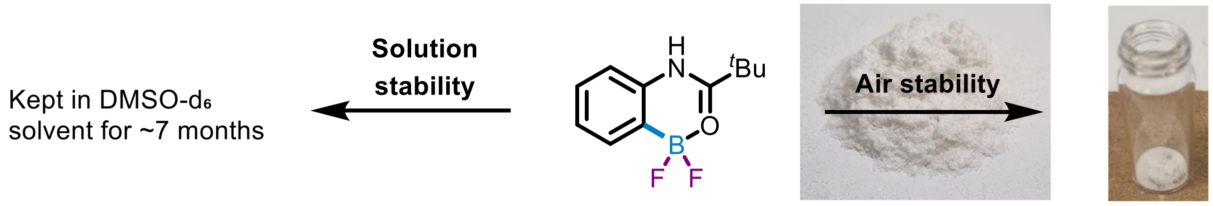

A vial was kept open for 7 days

Conclusion: Proton NMR indicated stability in both solution and air samples.

7.1 Air stability:

Protocol: Ar-BF_2 compound **3a** was placed in a vial and exposed to air for seven days. NMR analysis revealed no signs of decomposition or the appearance of any new peaks, confirming the compound's air stability.

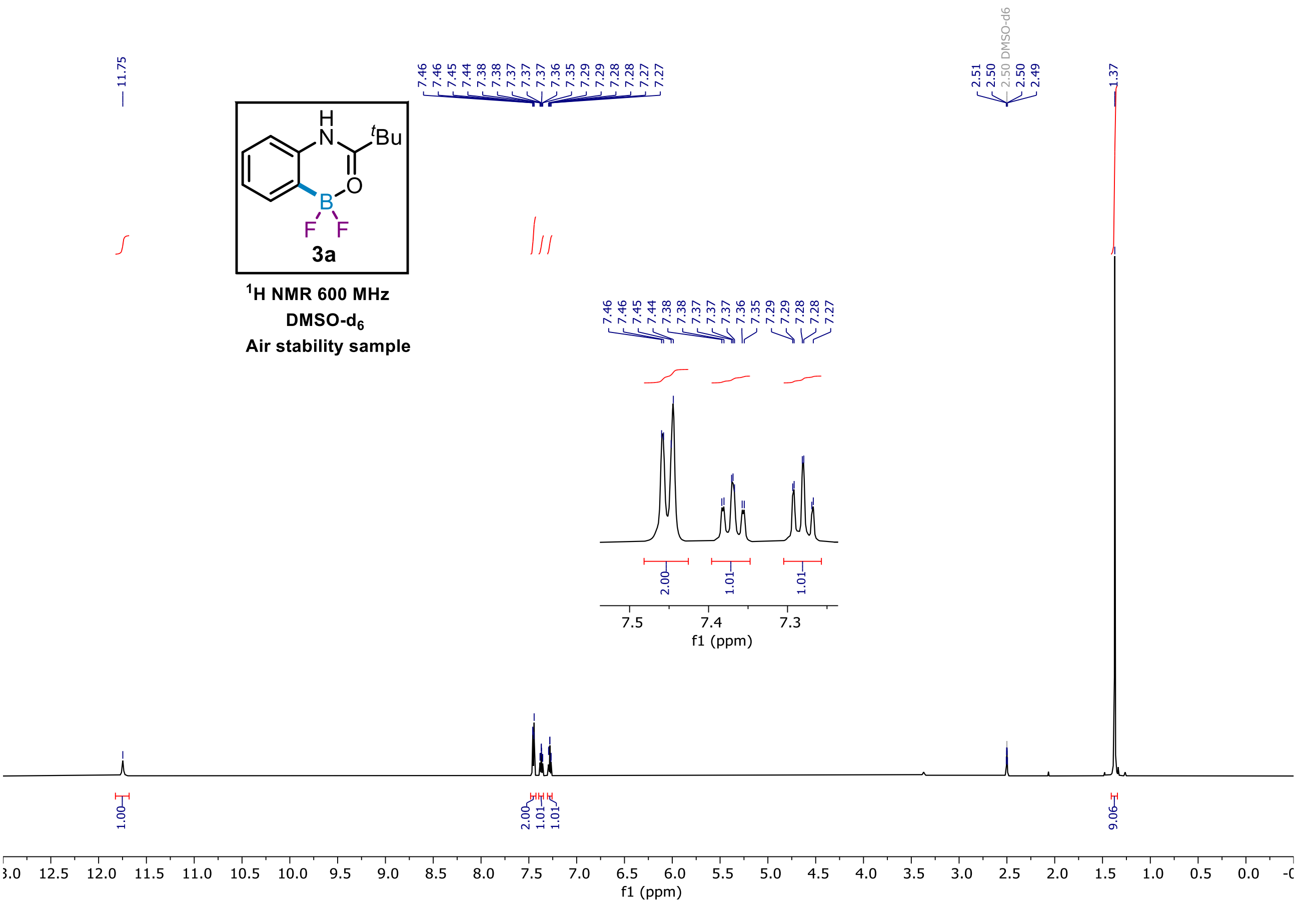

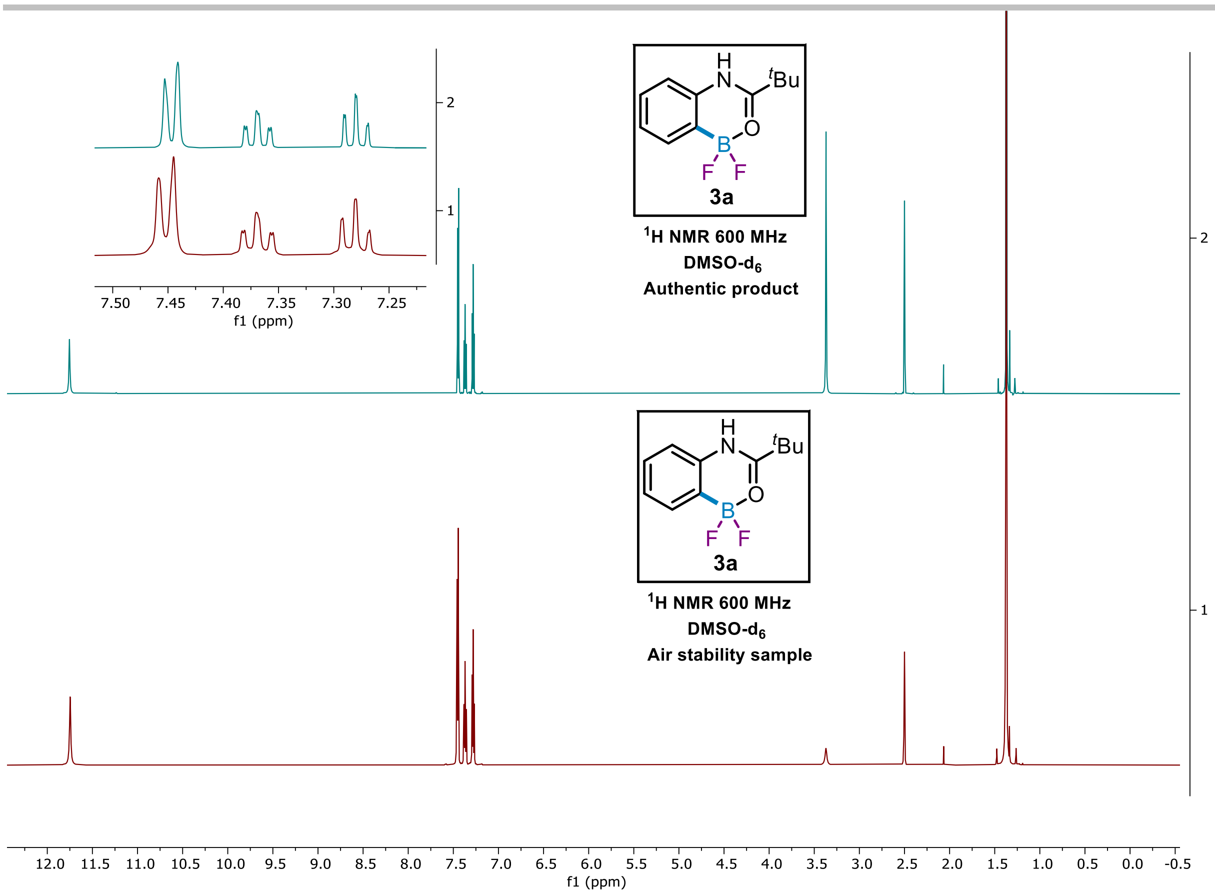

Figure: Stacked NMR of pure Ar-BF_2 3a and open air sample of Ar-BF_2 3a.

7.1.1 Solution stability: The Ar-BF_2 compound **3a** was dissolved in DMSO-d_6 and stored at ambient temperature for approximately seven months. Periodic monitoring using ^1H NMR spectroscopy showed no signs of decomposition.

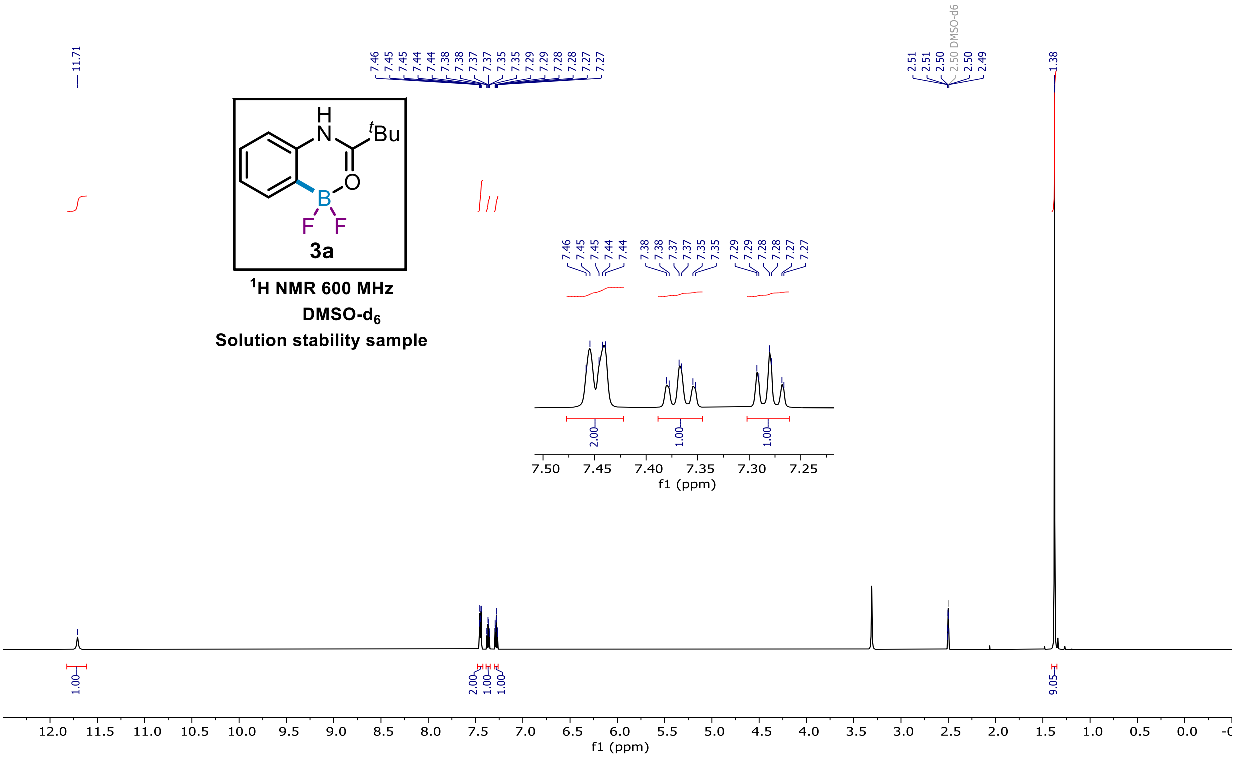

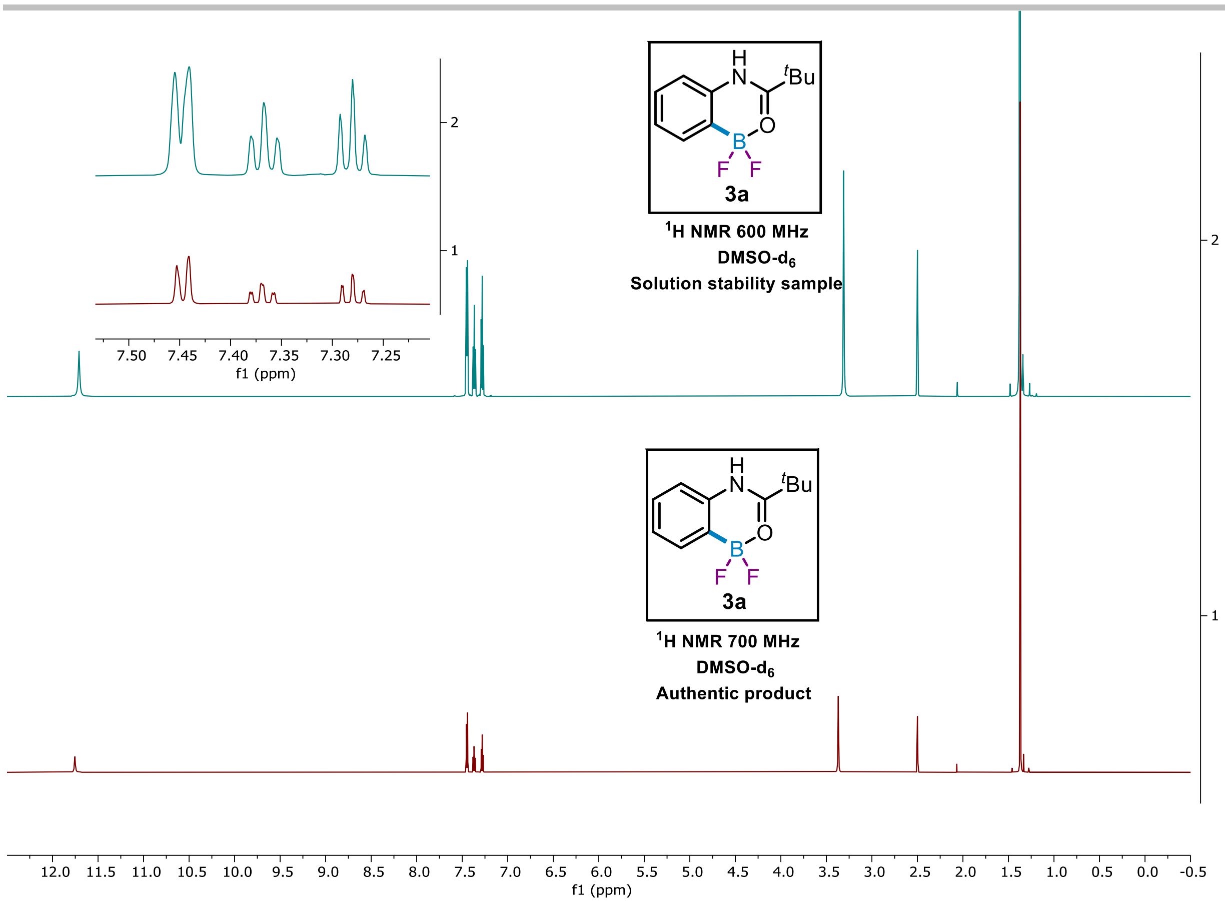

Figure: Stacked NMR of pure Ar-BF_2 **3a** and solution stability sample of Ar-BF_2 **3a**.

7.1.2 Solution stability of other Ar-BF2s:

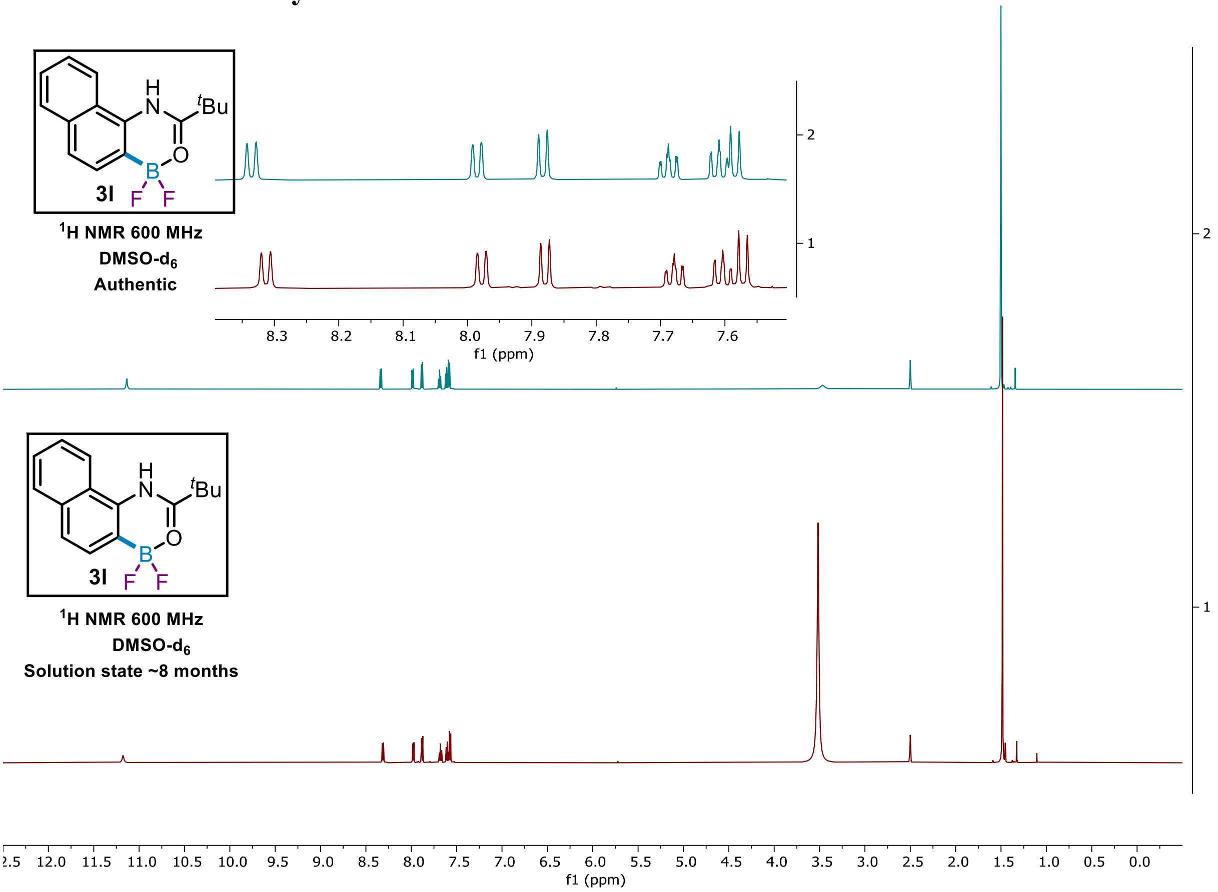

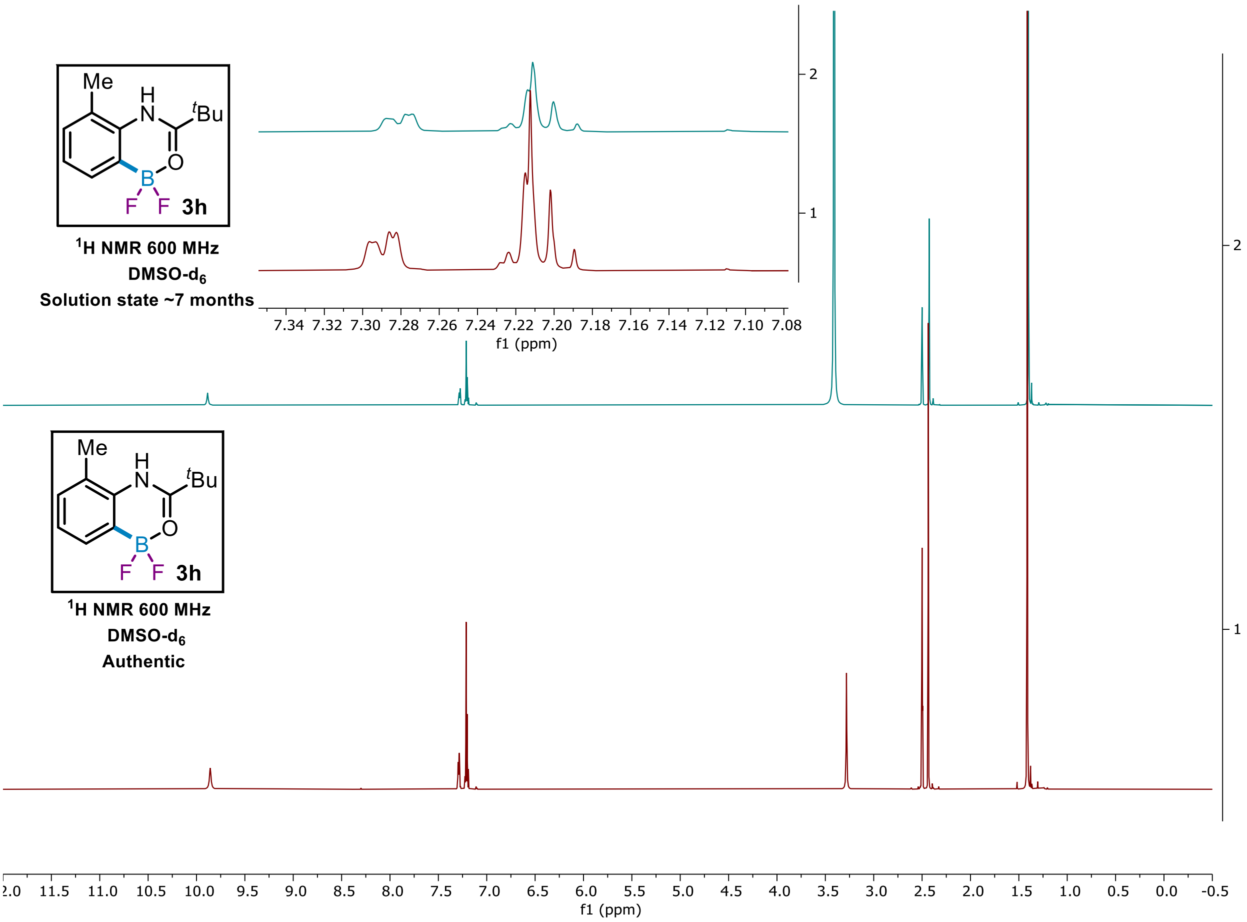

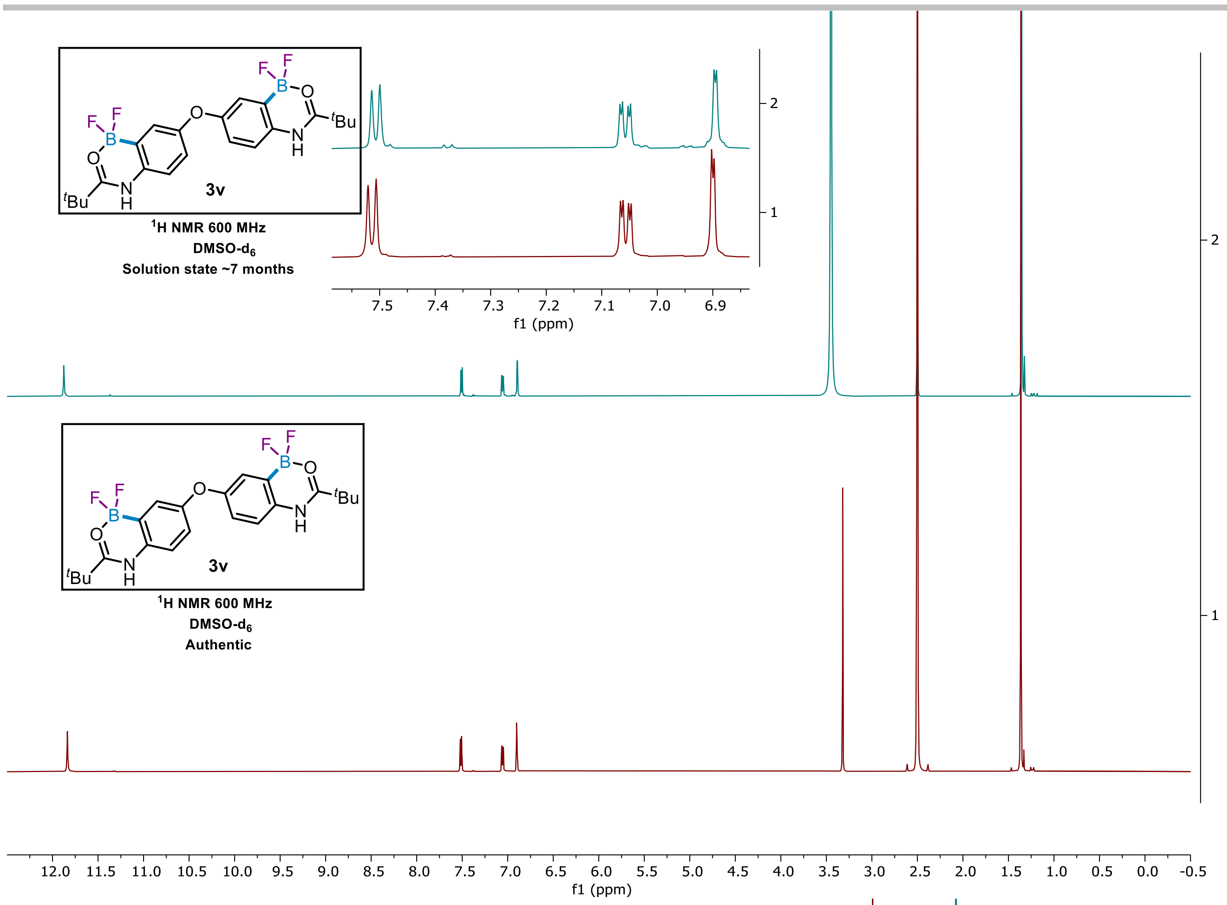
3v
¹H NMR 600 MHz
DMSO-d6
Solution state ~7 months
3v
¹H NMR 600 MHz
DMSO-d6
Authentic
f1 (ppm)

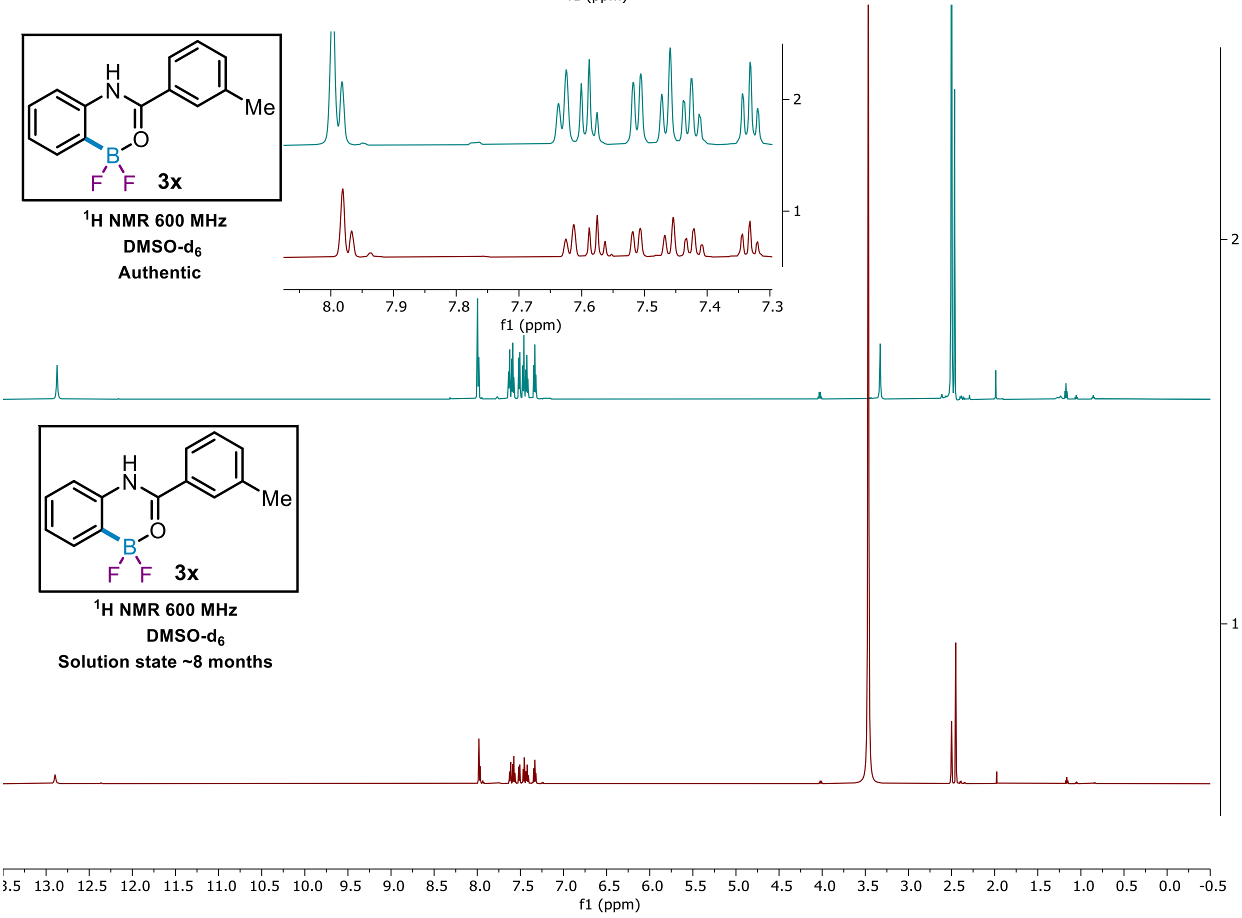
3x
¹H NMR 600 MHz
DMSO-d6
Authentic
3x
¹H NMR 600 MHz
DMSO-d6
Solution state ~8 months
f1 (ppm)

Conclusion: Compounds **3h**, **3l**, **3v**, and **3x** were stored in DMSO-d_6 for 7–8 months. NMR analysis revealed no signs of decomposition or the appearance of any significant new peaks.

7.2 Sustainability metrics

The E-factor was calculated as follows:

E–factor = (Mass of components- Mass of product)/Mass of product

mass of components = total mass of inputs − mass of product

Calculation of Green Metrics for Ar-Bpin (16)

The E-factor was determined following the reported synthetic protocol.[1] To complete the green metrics analysis, standard laboratory practices were applied to estimate the masses of materials used in the workup and purification, as these were not specified in the literature.

Chromatography: The purification of 50 mg of crude material was estimated to require 5 g of silica gel.

Solvent for Elution: The eluent volume was estimated at 10 column volumes (180 mL total), using a mixture of ethyl acetate and pentane in a 1:1.5 ratio.

Workup Procedure: The workup included drying the organic phase with 1.0 g of $MgSO_4$. A subsequent wash with 20 mL of ethyl acetate was included to account for product recovery during filtration.

**Ar-Bpin (16)**	Scale 0.2 mmol							
1. Reagent Molar Masses:		**2. Masses used (g)**	**2. Solvent Masses:**		3. Eluent Masses:		**4. Purification Masses:**	
N-phenylpivalamide (SM): (**1a**)	177,247 g·mol⁻¹ ($C_{11}H_{15}NO$).	0,03545	DCM (g): (1.33 g/mL)	2,2876	Ethyl acetate (g): (0.902 g/mL)	64,94	Alumina (g):	5
BBr_3 :	250,52 g·mol⁻¹.	0,0551	Ethyl acetate (g):	18,04	Pentane (g): (0.626 g/mL)	71,28	$MgSO_4$(g) :	1
Pinacol :	118,17 g·mol⁻¹ ($C_6H_{14}O_2$).	0,02369	Water (g):	0,5	Total Eluent:	136,2		
K2CO3	138.21	0,0691						
Product Ar–Bpin :	303.2 g·mol⁻¹ ($C_{17}H_{26}BNO_3$).	0,0535						
	5. Total Reaction Mass:	2,97094			6. Total Purification Mass:	160,3		
7. Total Mass of All Inputs:	163,23094	8. E-Factor Calculation:						
Total Inputs = Total Reaction Mass + Total Purification Mass		8.1 Mass of Product:	0,0535	5,35E-05				
		8.2 Mass of Waste = Total Inputs - Product Mass						
		**Mass of waste**	163,17744	0,163177				
			3050,045607					
		8.3 E-Factor =	~3050					
This means for every 1 kg of product isolated, about 3,050 kg of waste is generated.								

Calculation of Green Metrics for Ar-BF_2 (3a)

For 1 g scale the desired product **3a** was isolated in 93% yield (1.18 g).

Workup and purification protocol for 1g scale: After removal of complete acetonitrile, the crude solid was filtered under *vacuo* and washed with 60 mL distilled water. Then the crude solid was washed with 60 mL pentane to afford desired Ar-BF_2 product **3a.**

**Ar-BF_2 (3a)**	Scale 1 g					
1. Reagent Molar Masses:		**2. Masses used (g)**	**3. Solvent Masses:**		**4. Purification Masses:**	
N-phenylpivalamide (SM): (**1a**)	177,247 g·mol^{-1} ($C_{11}H_{15}NO$).	1	DCM (g): (1.33 g/mL)	33,656	Pentane (g): (0.626 g/mL)	37,56
BBr_3 :	250,52 g·mol^{-1}.	1,696	Acetonitrile (g): (0.78	29,554	Water (g):	60
$NaBF_4$: :	109,794 g·mol^{-1}	1,55	Water (g):	18,6	Total Eluent:	97,56
Product Ar–BF_2 : (93% yield)	225,043 g·mol^{-1} .	1,18	Total solvent (g):	81,81		
	5. Total Reaction Mass:	86,056			6. Total Purification Mass:	97,56
7. Total Mass of All Inputs:	183,616	8. E-Factor Calculation:				
Total Inputs = Total Reaction Mass + Total Purification Mass		**8.1** Mass of Product	1,18	0,00118		
		8.2 Mass of Waste = Total Inputs - Product Mass				
		**Mass of waste**	182,436	0,182436		
			154,6067797			
		**8.3 E-Factor =**	~154			
This means for every 1 kg of product isolated, about 154 kg of waste is generated.						

8. Proposed de novo synthesis

Proposed route for compound 3am:

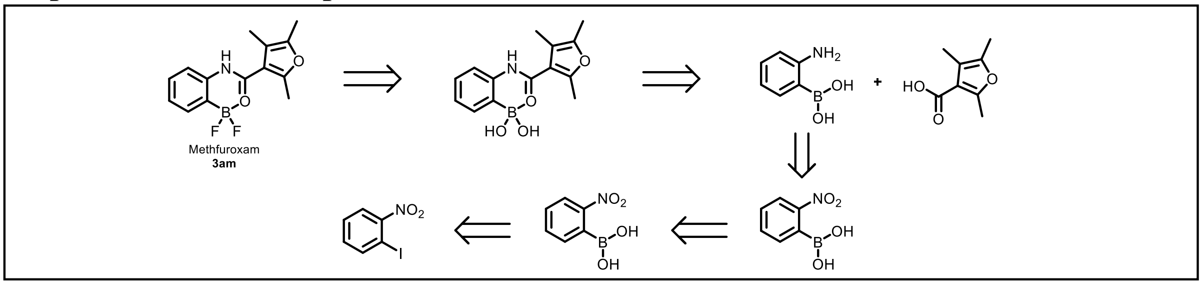

Proposed route for compound 3an:

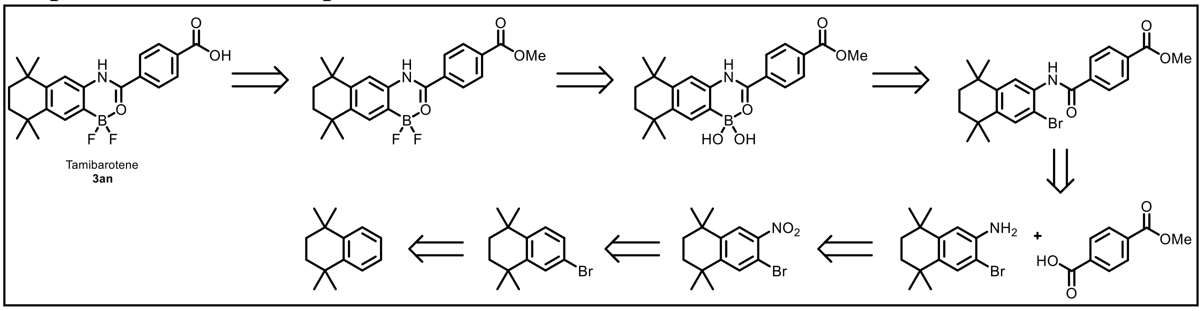

Proposed route for compound 3ao:

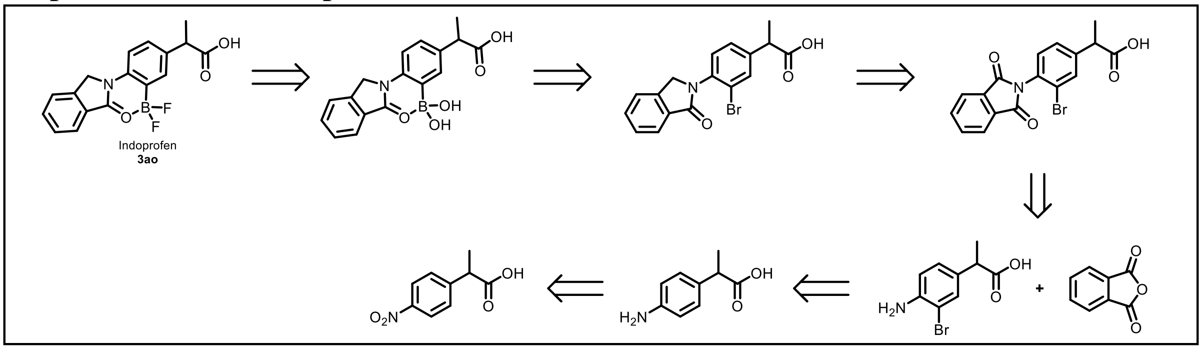

Proposed route for compound 3aq:

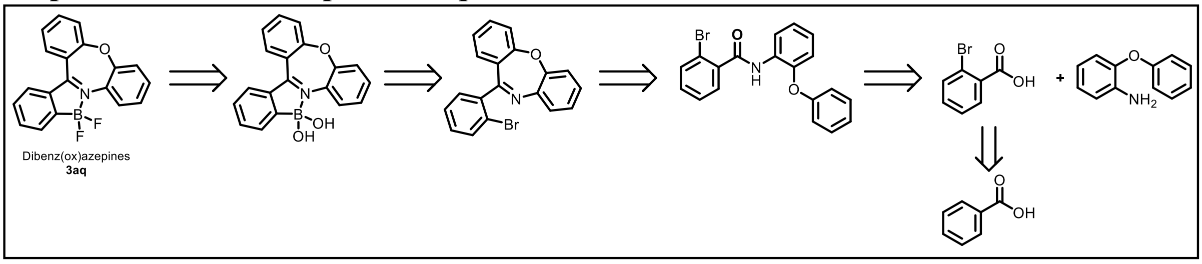

Proposed route for compound 3ar:

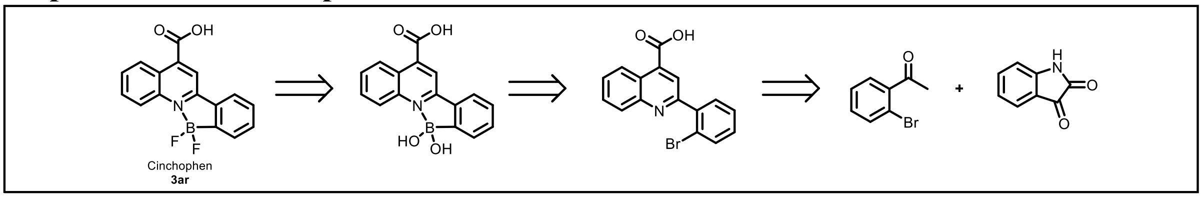

Proposed route for compound 3as´:

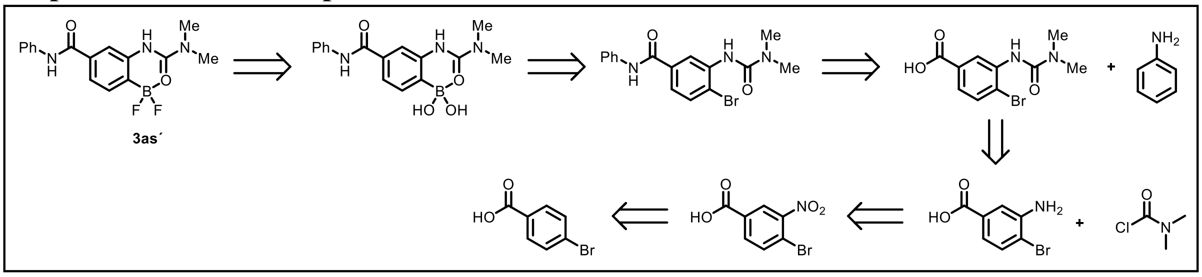

8.1 Comparison study of Ar−BBr_2 and Ar−BF_2

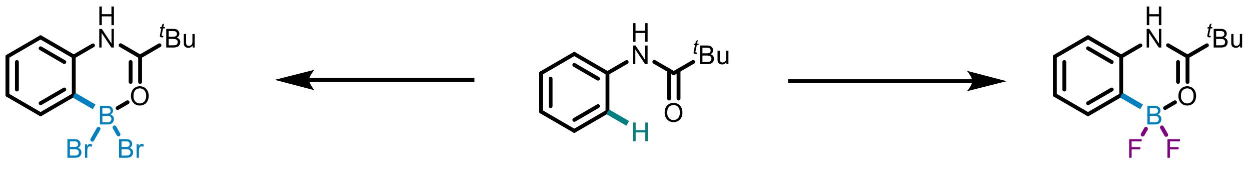

2a **1a** **3a**

- Unstable under air and solution
- Decomposes or reacts with nucleophilic solvents

- No column chromatography
- Multigram scalability
- Can be activated under metal-free and metal-catalyzed conditions

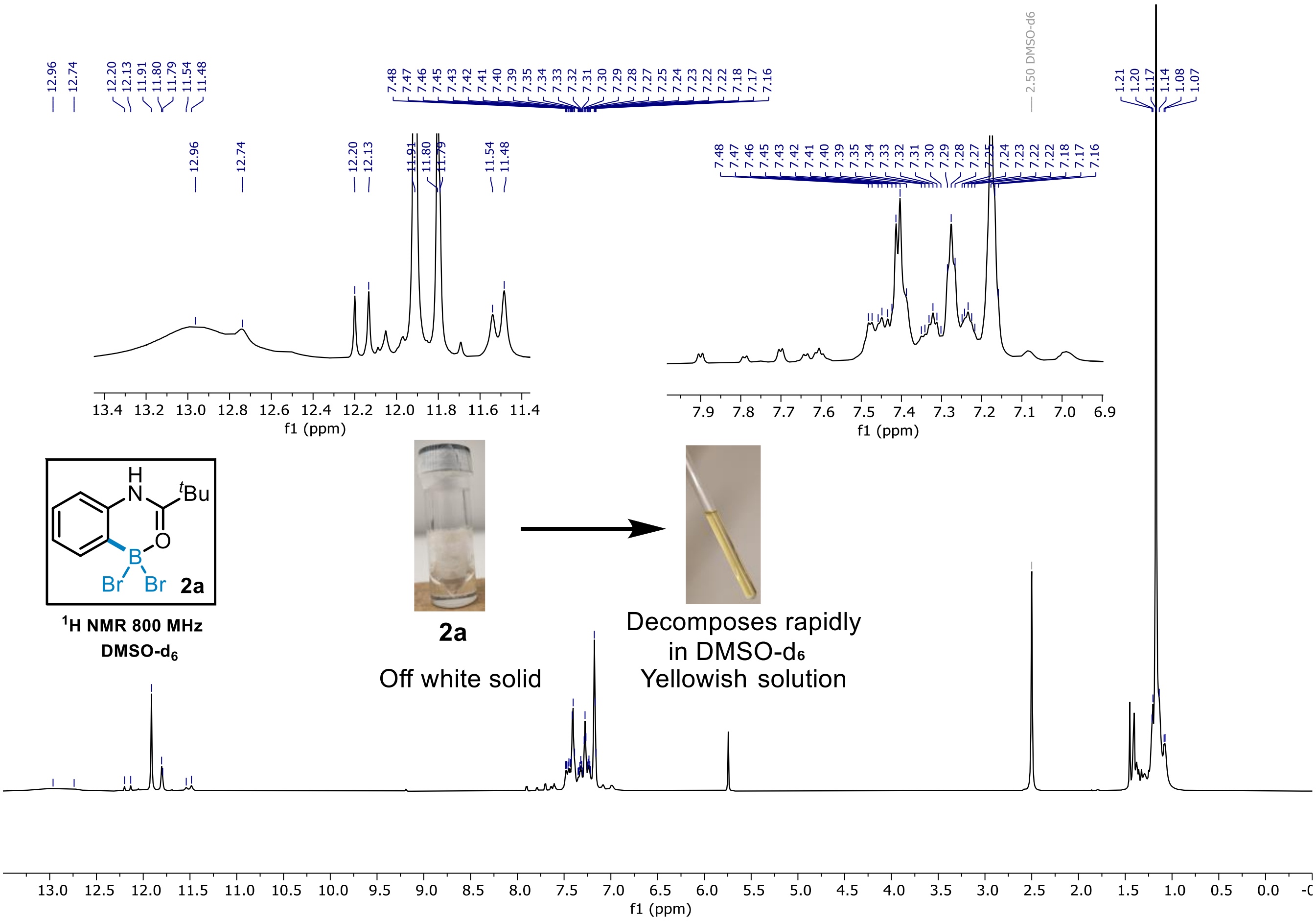

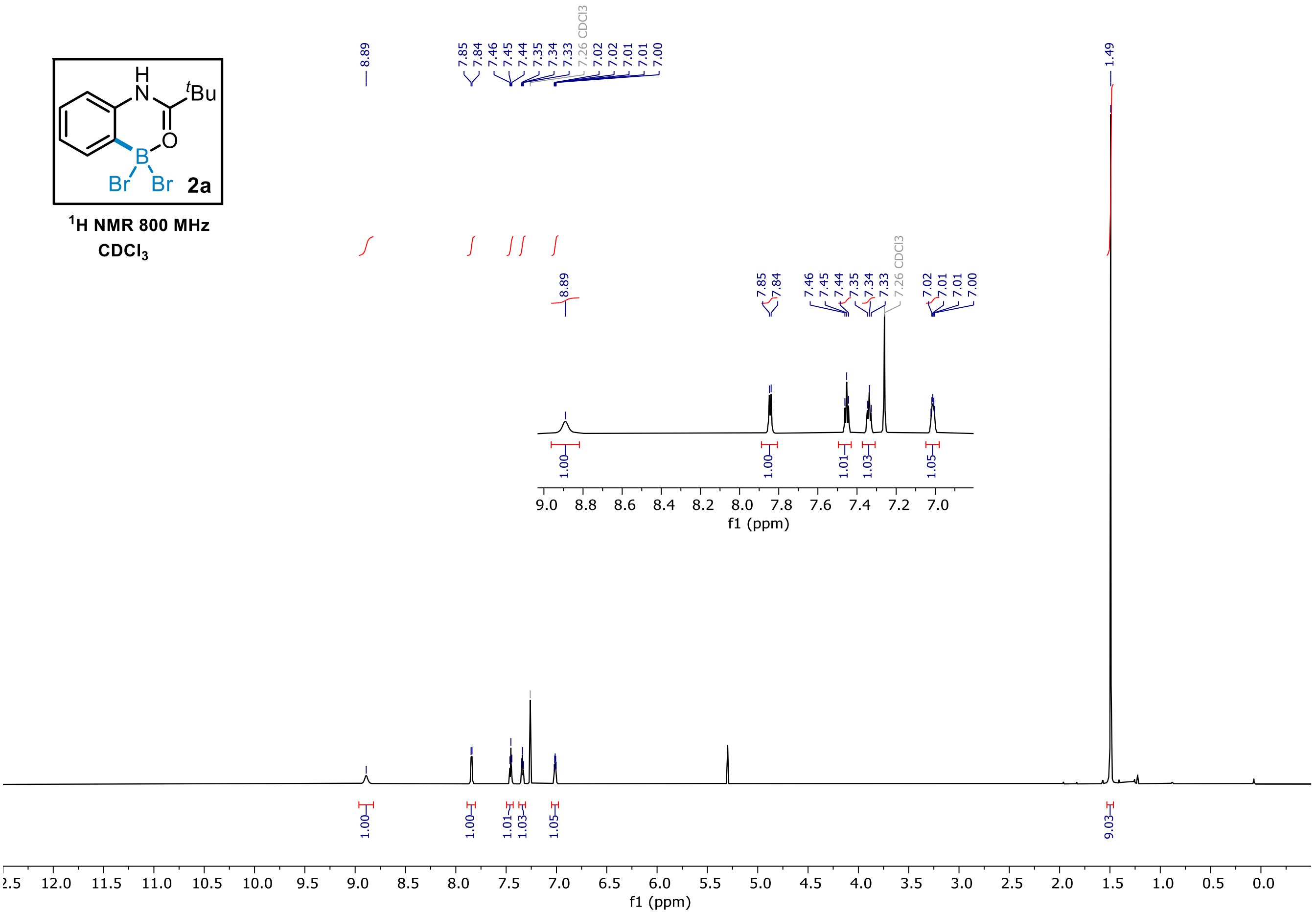

Conclusion: The proton NMR spectrum of crude **2a** in DMSO-d_6 displayed multiple peaks. The off-white solid turned into a yellowish solution upon dissolution in DMSO-d_6. The NMR measurement was conducted within 10 minutes of dissolving **2a** in DMSO-d_6. In contrast, the NMR spectrum of crude **2a** in $CDCl_3$ showed no signs of decomposition, indicating that **2a** is unstable in nucleophilic or chelating solvents.

8.2 Reactivity comparison study of Ar−BBr_2, Ar−BF_2

Radio-iodination attempt from $ArBBr_2$

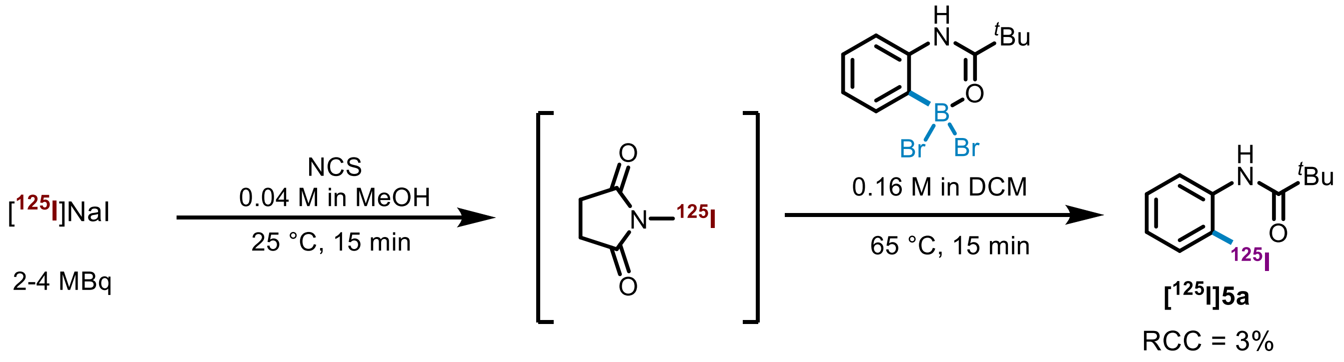

In a round bottom flask at 0 °C under N_2, were introduced *N*-phenylpivalamide (0.2 g, 1.13 mmol) and BBr_3 (1M in DCM, 1.35 mL, 1.35 mmol). After addition, the flask was allowed to stir at rt for 2h and completion of the reaction was checked by TLC. The flask was then evaporated to dryness under *vacuum* and DCM (7.06 mL) was added to obtain a 0.16 M **$ArBBr_2$** solution. In a V-vial equipped with a stir bar, *N*-chlorosuccinimide (40 µL, 0.04 M in MeOH, 1.6 µmol, 0.5 equiv.) was added to a methanolic solution of [^{125}I]sodium iodide (5 µL, 2-4 MBq). The resulting mixture was stirred for 15 minutes at 25 °C and the substrate **$ArBBr_2$** (20 µL, 0.16 M in DCM, 3 µmol, 1 equiv.) was added. The reaction mixture was stirred again for 15 minutes at 65 °C. After reaction, the mixture was quenched with a solution of sodium thiosulfate (200 µL, 0.05 M in water) and diluted with methanol (1100 µL). An aliquot was removed for analysis by radio-HPLC to assess the radiochemical conversion (RCC = 3%)

RADIO-CHROMATOGRAM

The data is presented in the following order: a radio-chromatogram and a UV-chromatogram of the crude solution, and UV-chromatogram of the cold reference.

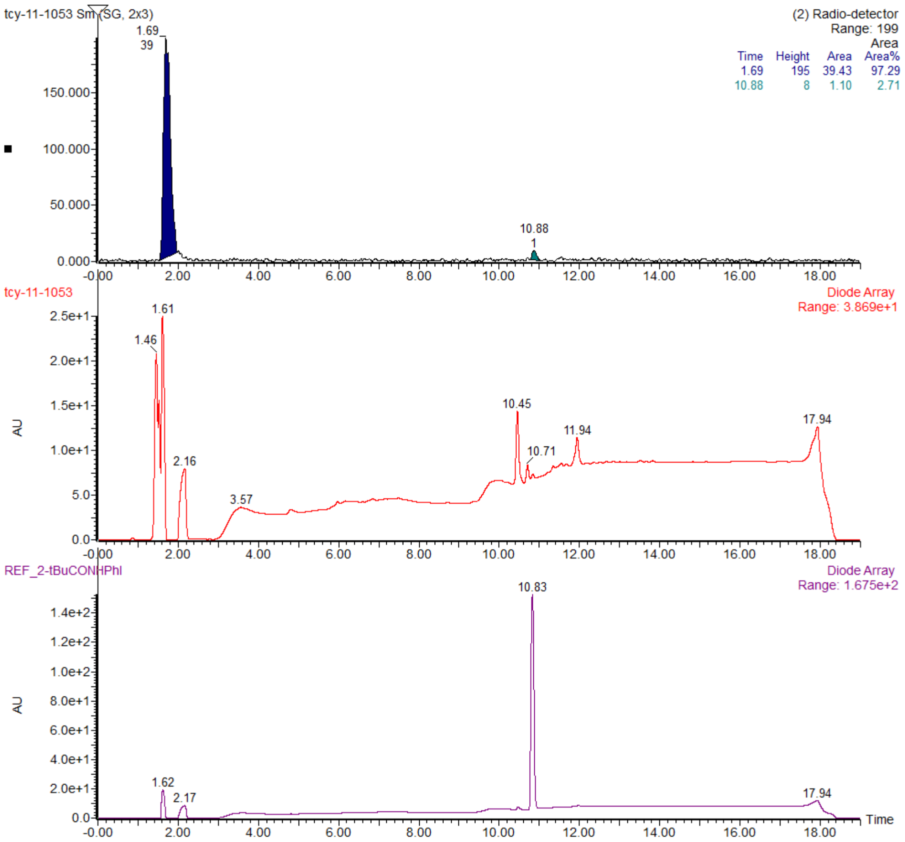

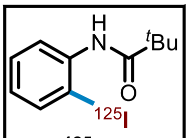

[^{125}I]5a

8.3. Reactivity comparison study of BBr_2 (2a), BF_2 (3a) and Bpin (16) derivative

8.3.1 Halogenation of BBr_2 derivative (2a):

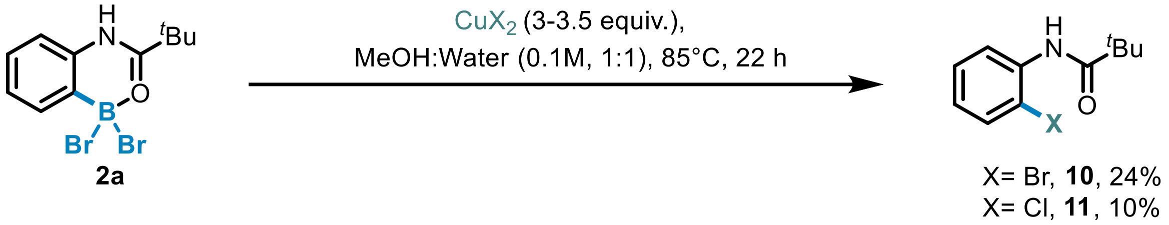

Step i) In a screw-capped 2 mL reaction vial equipped with a triangular Teflon-coated spinvane stir bar, the pivalamide **1a** (0.1 mmol, 1 equiv.) in anhydrous CH_2Cl_2 (0.4 mL) under a nitrogen atmosphere was added dropwise BBr_3 (1.2 equiv., 1M solution in CH_2Cl_2). After the complete addition of BBr_3, the reaction mixture was stirred at 22 ºC for 2 h after which the solvent was removed under reduced pressure. Step ii) To the crude residue from step i) were added 0.5 mL MeOH and 0.5 mL distilled water with copper(II) bromide (3.5 equiv.). The reaction mixture was heated at 85 ºC for 22 h. The reaction was allowed to reach room temperature and diluted with 2 mL ethyl acetate and filtered through a pad of celite and sodium sulfate. The celite was washed with 10 mL ethyl acetate and the filtrate was concentrated *in vacuo* to afford the crude product. The crude product yields and product identities were confirmed by ^{1}H NMR spectroscopy using 1,1,2,2-tetrachloroethane (0.1 mmol) as an internal standard. Yields were calculated relative to the limiting reagent (**1a**). A 24% formation of the brominated product (**10**) was observed in the crude ^{1}H NMR spectrum (Figure 8.3.1.1).

Procedure for chlorination (**11**): Same as bromination procedure. Copper(II) chloride (3 equiv.) was used for chlorination. A 10% formation of the chlorinated product was observed in the crude ^{1}H NMR spectrum (Figure 8.3.1.2).

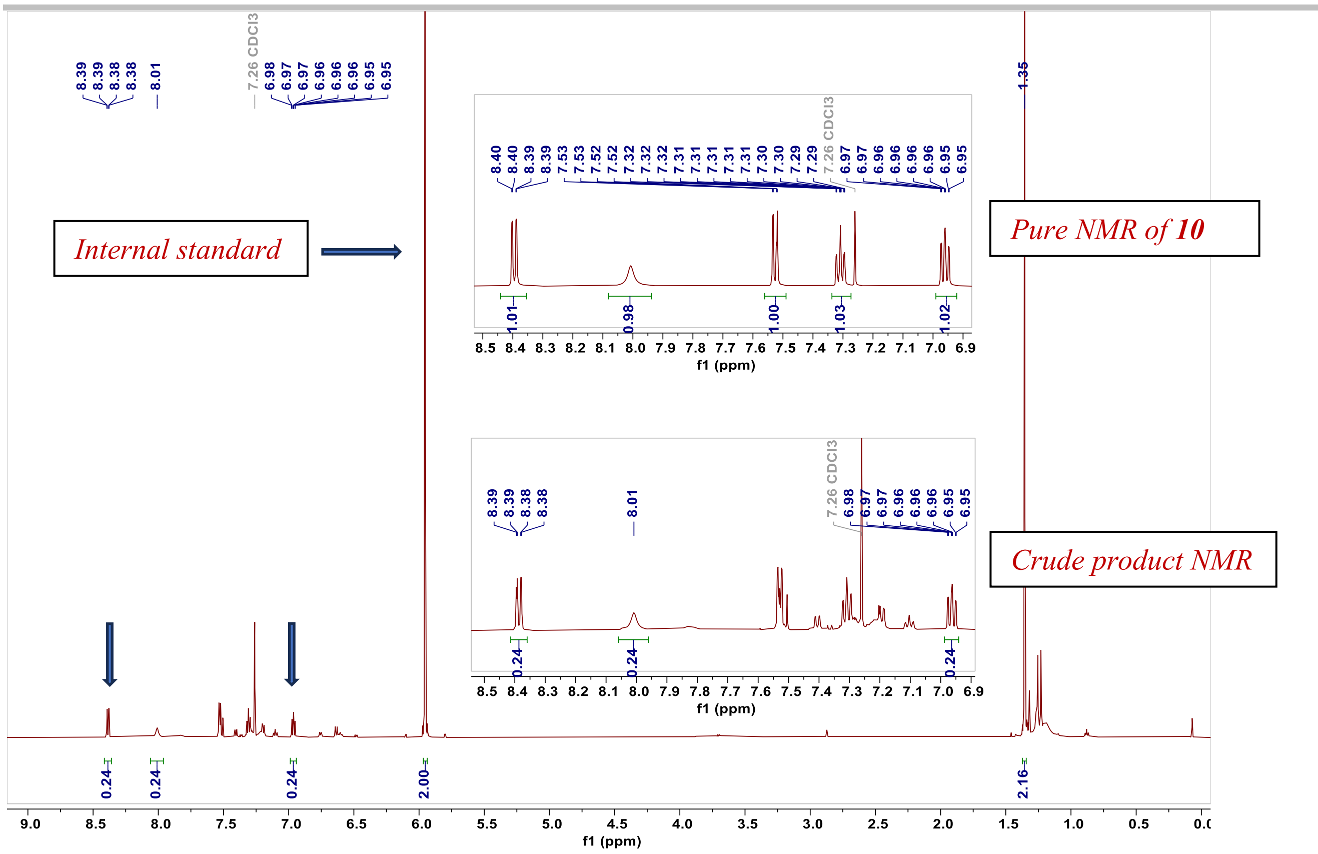

Figure 8.3.1.1: Crude ^{1}H NMR of **10**

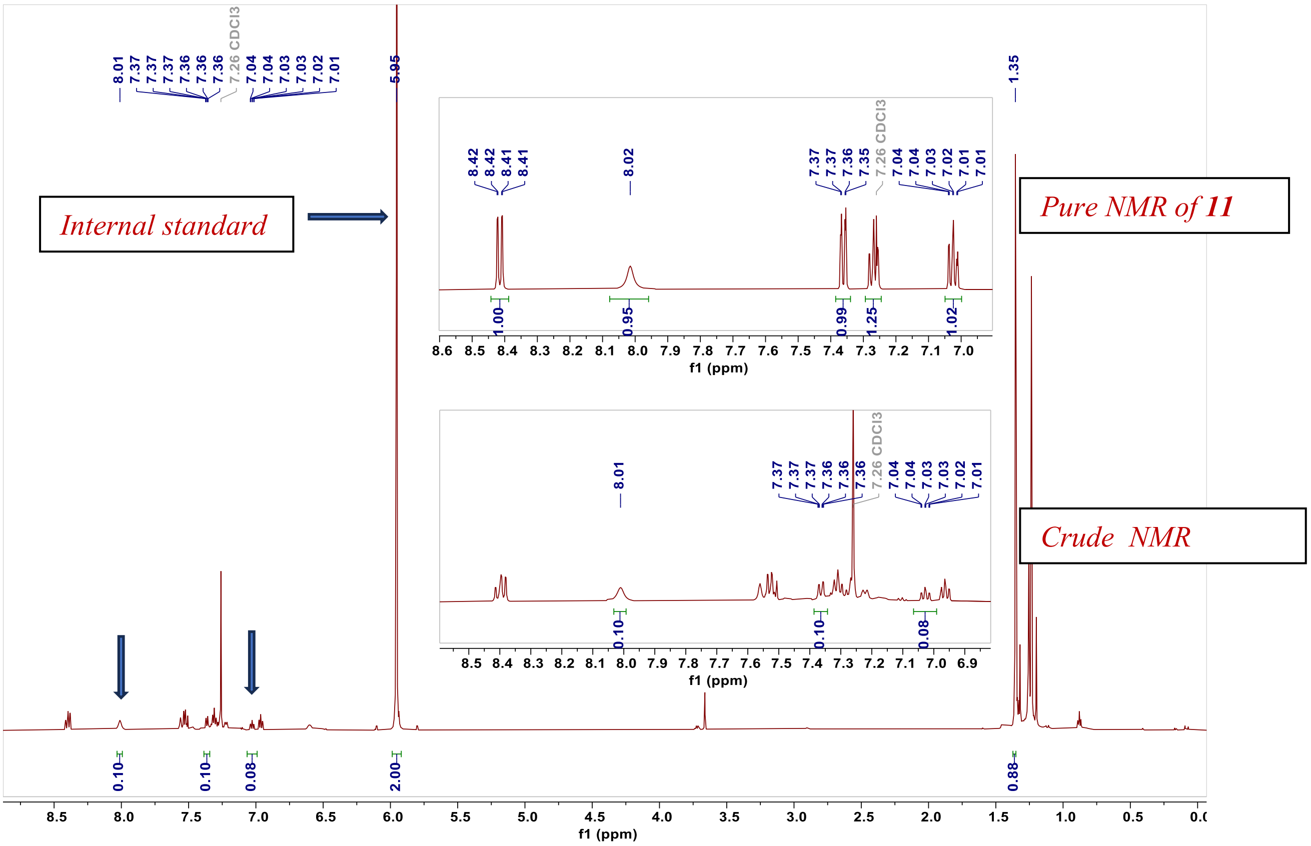

Figure 8.3.1.2: Crude ^{1}H NMR of **11**

8.3.2. Halogenation of Bpin derivative (16):

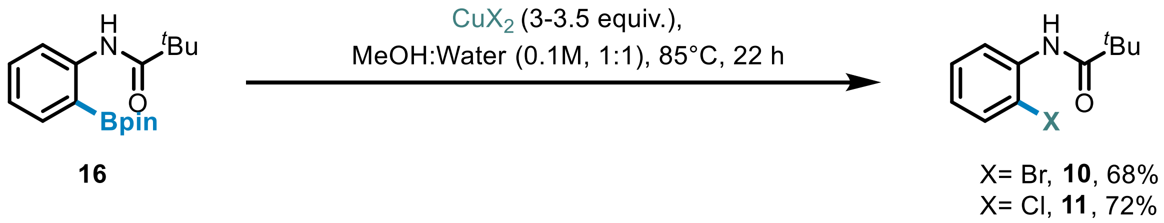

Compound **16** was prepared according to a previously reported protocol.[1]

Procedure for bromination (**10**): In a screw-capped 2 mL reaction vial equipped with a triangular Teflon-coated spinvane stir bar, the **11** (0.1 mmol, 1 equiv.) in 0.5 mL MeOH and 0.5 mL distilled water was added with copper(II) bromide (3.5 equiv.). The reaction mixture was heated at 85 ºC for 22 h. The reaction was allowed to reach room temperature and diluted with 2 mL ethyl acetate and filtered through a pad of celite and sodium sulfate. The celite was washed with 10 mL ethyl acetate and the filtrate was concentrated *in vacuo* to afford the crude product. The crude product yields and product identities were confirmed by ^{1}H NMR spectroscopy using 1,1,2,2-tetrachloroethane (0.1 mmol) as an internal standard. Yields were calculated relative to the limiting reagent (Ar-Bpin **16**). A 68% formation of the brominated product (**10**) was observed in the crude ^{1}H NMR spectrum (Figure 8.3.2.1).

Procedure for chlorination (**11**): Same as bromination procedure. Copper(II) chloride (3 equiv.) was used for chlorination. A 72% formation of the chlorinated product was observed in the crude ^{1}H NMR spectrum (Figure 8.3.2.2).

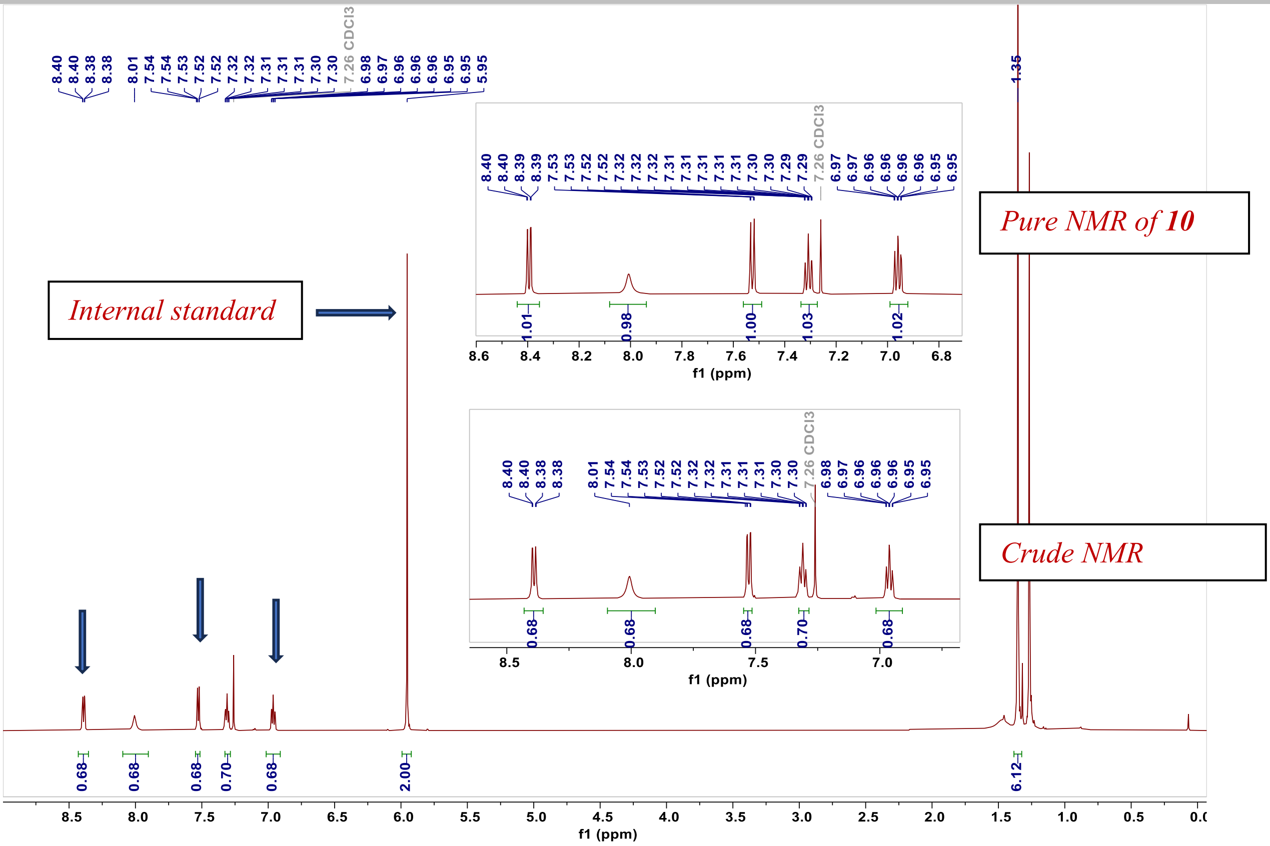

Figure 8.3.2.1: Crude ^{1}H NMR of **10**

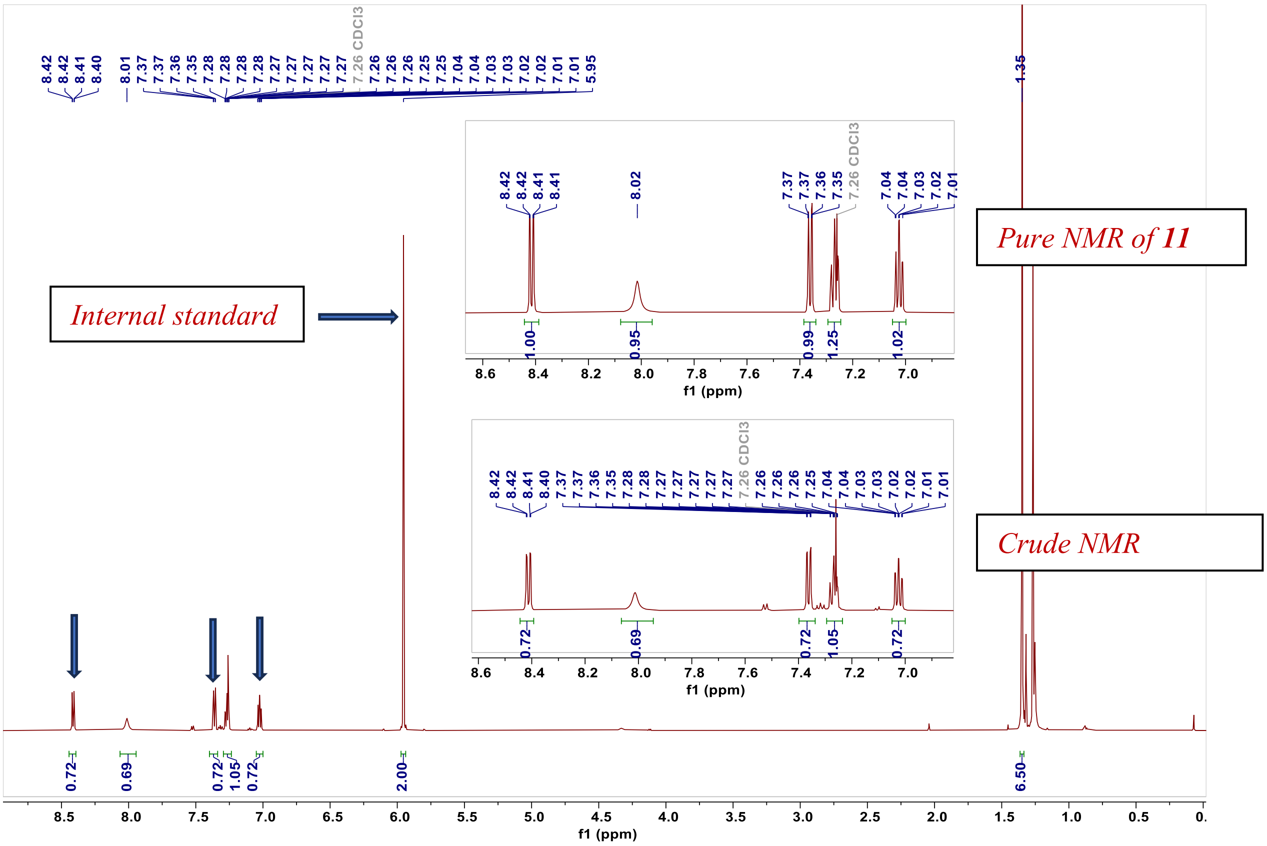

Figure 8.3.2.2: Crude ^{1}H NMR of **11**

8.3.3 Synthesis of *N,N'*-([1,1'-biphenyl]-2,2'-diyl)bis(2,2-dimethylpropanamide) (17) from BBr_2 derivative (2a):

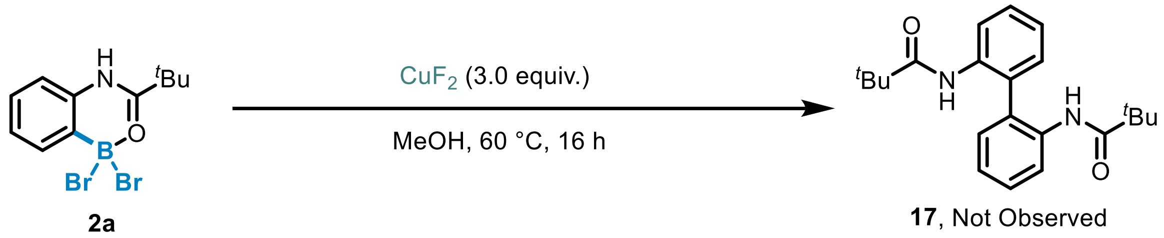

Step i) In a screw-capped 2 mL reaction vial equipped with a triangular Teflon-coated spinvane stir bar, the pivalamide **1a** (0.1 mmol, 1 equiv.) in anhydrous CH_2Cl_2 (0.4 mL) under a nitrogen atmosphere was added dropwise BBr_3 (1.2 equiv., 1M solution in CH_2Cl_2). After the complete addition of BBr_3, the reaction mixture was stirred at 22 ºC for 2 h after which the solvent was removed under reduced pressure. Step ii) To the crude residue from step i) were added 0.6 mL dry MeOH was added with copper(II) fluoride (3.0 equiv.). The reaction mixture was heated at 60 ºC for 16 h. The reaction was allowed to reach room temperature and diluted with 2 mL ethyl acetate and filtered through a pad of celite and sodium sulfate. The celite was washed with 10 mL ethyl acetate and the filtrate was concentrated *in vacuo* to afford the crude product. The crude product yields and product identities were confirmed by ^1H NMR spectroscopy using 1,1,2,2-tetrachloroethane (0.1 mmol) as an internal standard. No product formation was observed in the crude ^1H NMR spectrum (Figure 8.3.3.1).

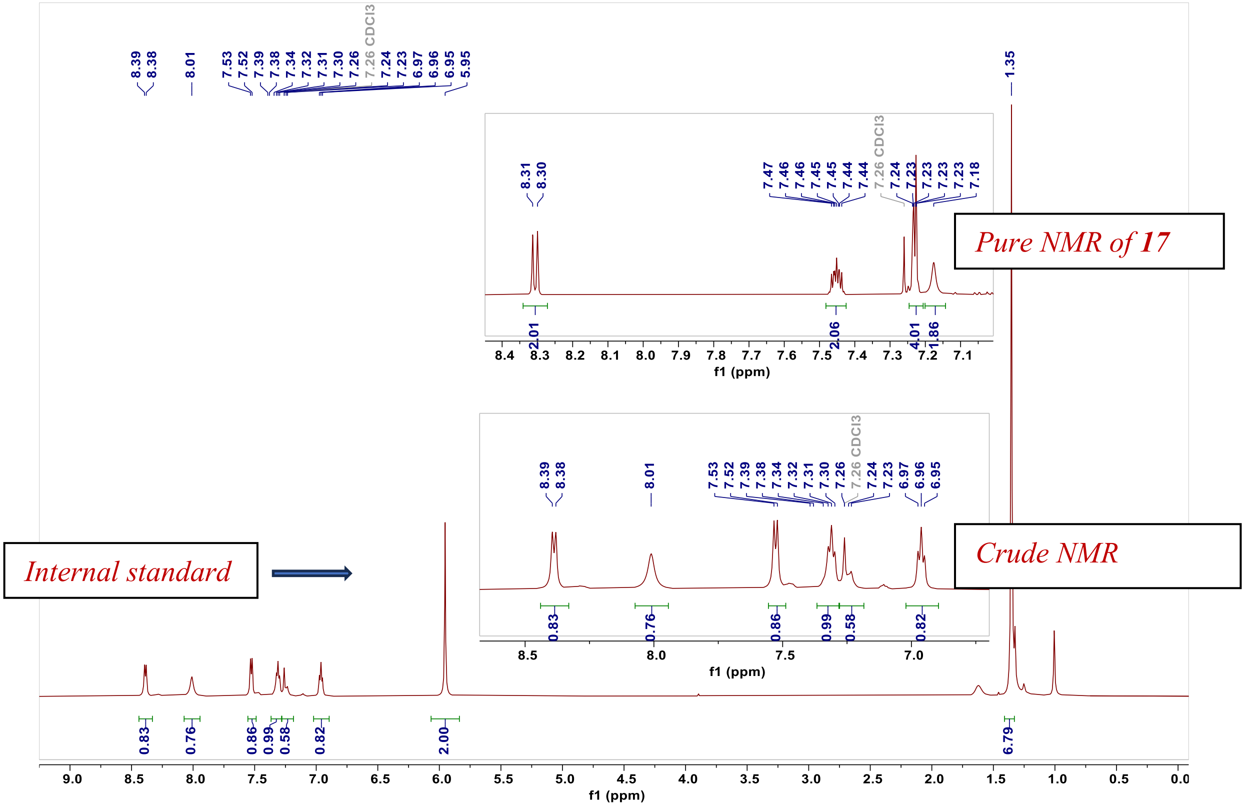

Figure 8.3.3.1: Crude ^1H NMR of **17**

8.3.4 Synthesis of *N,N'*-([1,1'-biphenyl]-2,2'-diyl)bis(2,2-dimethylpropanamide) (17) from Bpin derivative (16):

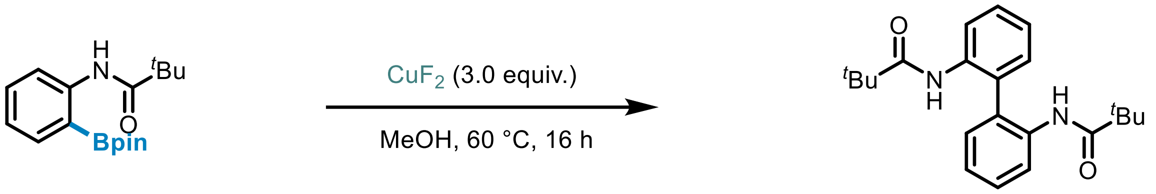

16 **17**, 71% NMR yield

Procedure: In a screw-capped 2 mL reaction vial equipped with a triangular Teflon-coated spinvane stir bar, the Ar-Bpin **16** (0.1 mmol, 1 equiv.) in 0.6 mL dry MeOH was added with copper(II) fluoride (3.0 equiv.). The reaction mixture was heated at 60 ºC for 16 h. The reaction was allowed to reach room temperature and diluted with 2 mL ethyl acetate and filtered through a pad of celite and sodium sulfate. The celite was washed with 10 mL ethyl acetate and the filtrate was concentrated *in vacuo* to afford the crude product. The crude product yields and product identities were confirmed by ^{1}H NMR spectroscopy using 1,1,2,2-tetrachloroethane (0.1 mmol) as an internal standard. Yields were calculated relative to the limiting reagent (**1a**). A 71% formation of the **17** was observed in the crude ^{1}H NMR spectrum (**Figure** 8.3.4.1).

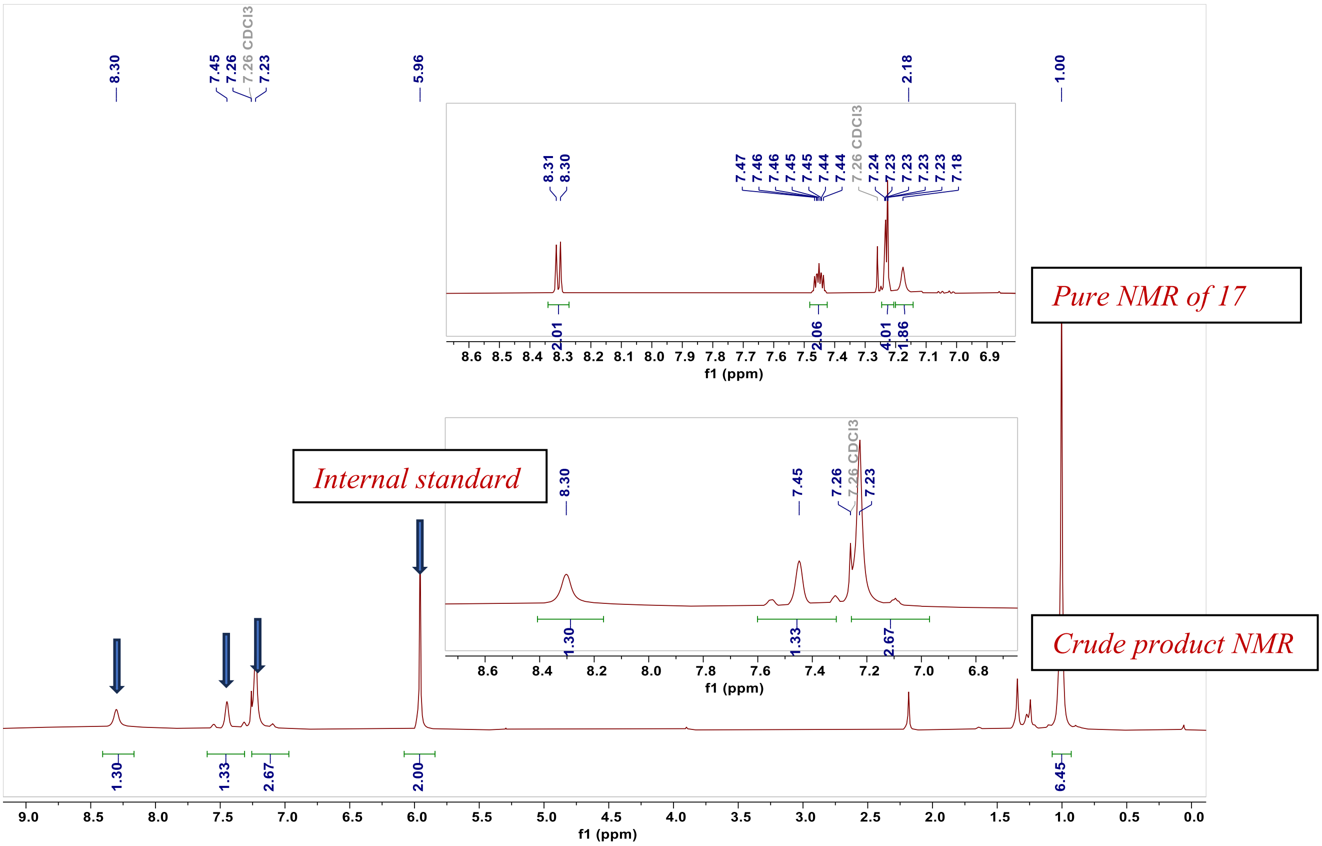

Figure 8.3.4.1: Crude ^{1}H NMR of **17**

8.3.5. Synthesisn of 2-(*tert*-butyl)benzo[*d*]oxazole (18) from BBr2 derivative (2a):

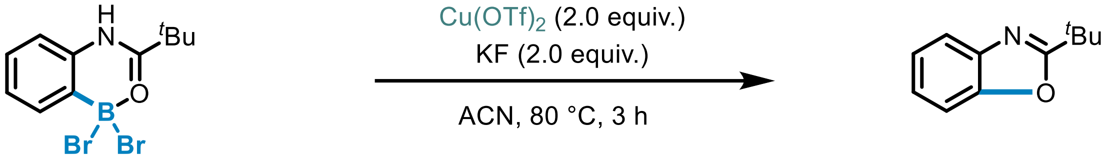

2a **18**, Not Observed

Step i) In a screw-capped 2 mL reaction vial equipped with a triangular Teflon-coated spinvane stir bar, the pivalamide **1a** (0.1 mmol, 1 equiv.) in anhydrous CH_2Cl_2 (0.4 mL) under a nitrogen atmosphere was added dropwise BBr_3 (1.2 equiv., 1M solution in CH_2Cl_2). After the complete addition of BBr_3, the reaction mixture was stirred at 22 ºC for 2 h after which the solvent was removed under reduced pressure. Step ii) To the crude residue from step i) were added 0.6 mL dry ACN with copper(II) triflate (2.0 equiv.) and KF (2.0 equiv.). The reaction mixture was heated at 80 ºC for 3 h. The reaction was allowed to reach room temperature and diluted with 2 mL ethyl acetate and filtered through a pad of celite and sodium sulfate. The celite was washed with 10 mL ethyl acetate and the filtrate was concentrated *in vacuo* to afford the crude product. The crude product yields and product identities were confirmed by ^{1}H NMR spectroscopy using 1,1,2,2-tetrachloroethane (0.1 mmol) as an internal standard. No product formation was observed in the crude ^{1}H NMR spectrum (**Figure** 8.3.5.1).

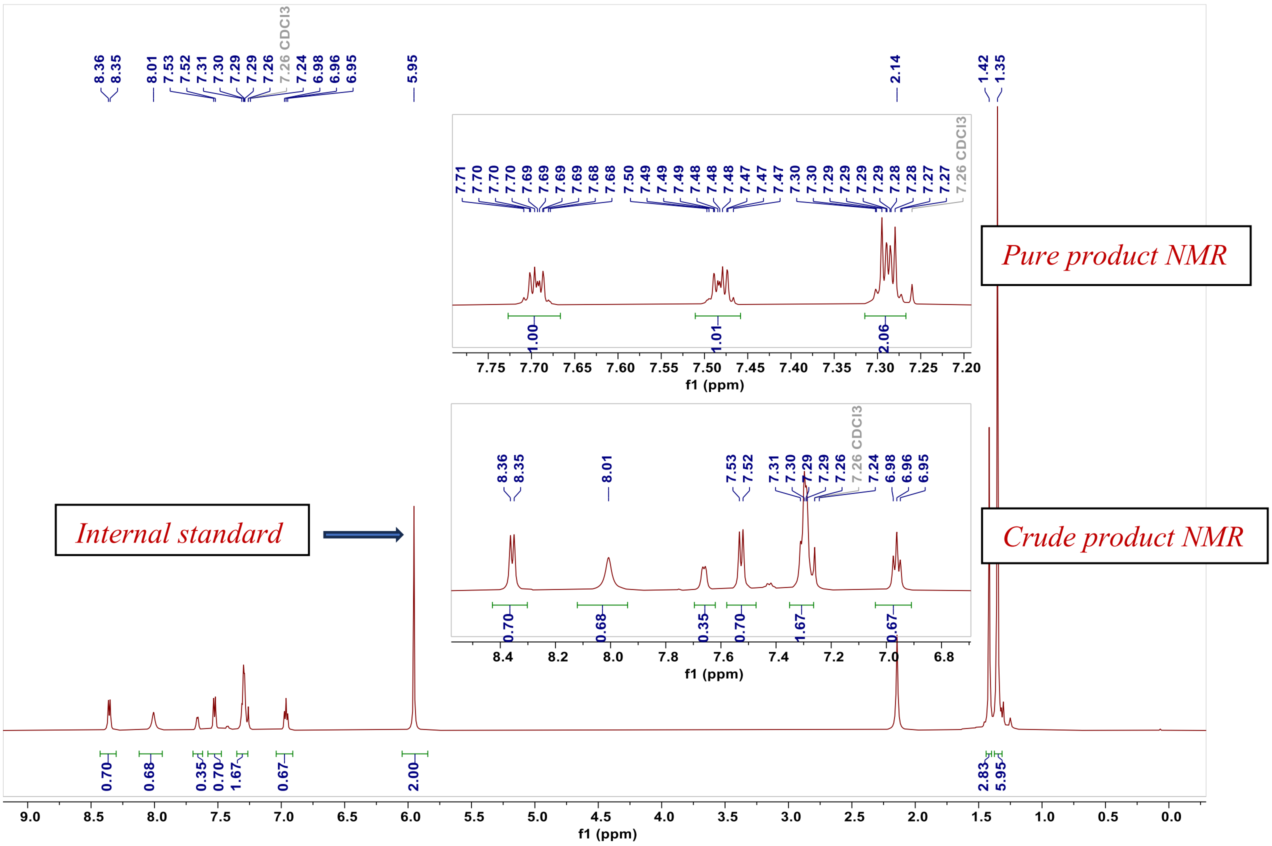

Figure 8.3.5.1: Crude ^{1}H NMR of **18**

8.3.6 Synthesis 2-(*tert*-butyl)benzo[*d*]oxazole (18) from Bpin (16) derivative:

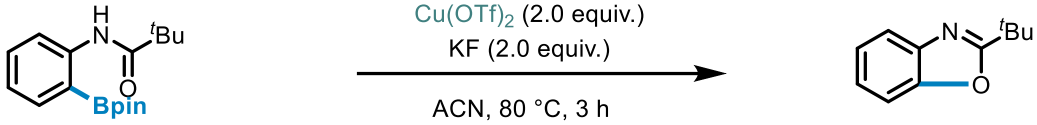

16 **18**, Not Observed

Procedure:[15] In a screw-capped 2 mL reaction vial equipped with a triangular Teflon-coated spinvane stir bar, the Ar-Bpin **16** (0.1 mmol, 1 equiv.) in 0.6 mL dry ACN was added with copper(II) triflate (2.0 equiv.) and KF (2.0 equiv.). The reaction mixture was heated at 80 ºC for 3 h. The reaction was allowed to reach room temperature and diluted with 2 mL ethyl acetate and filtered through a pad of celite and sodium sulfate. The celite was washed with 10 mL ethyl acetate and the filtrate was concentrated *in vacuo* to afford the crude product. The crude product yields and product identities were confirmed by ^{1}H NMR spectroscopy using 1,1,2,2-tetrachloroethane (0.1 mmol) as an internal standard. No product formation was observed in the crude ^{1}H NMR spectrum (**Figure** 8.3.6.1).

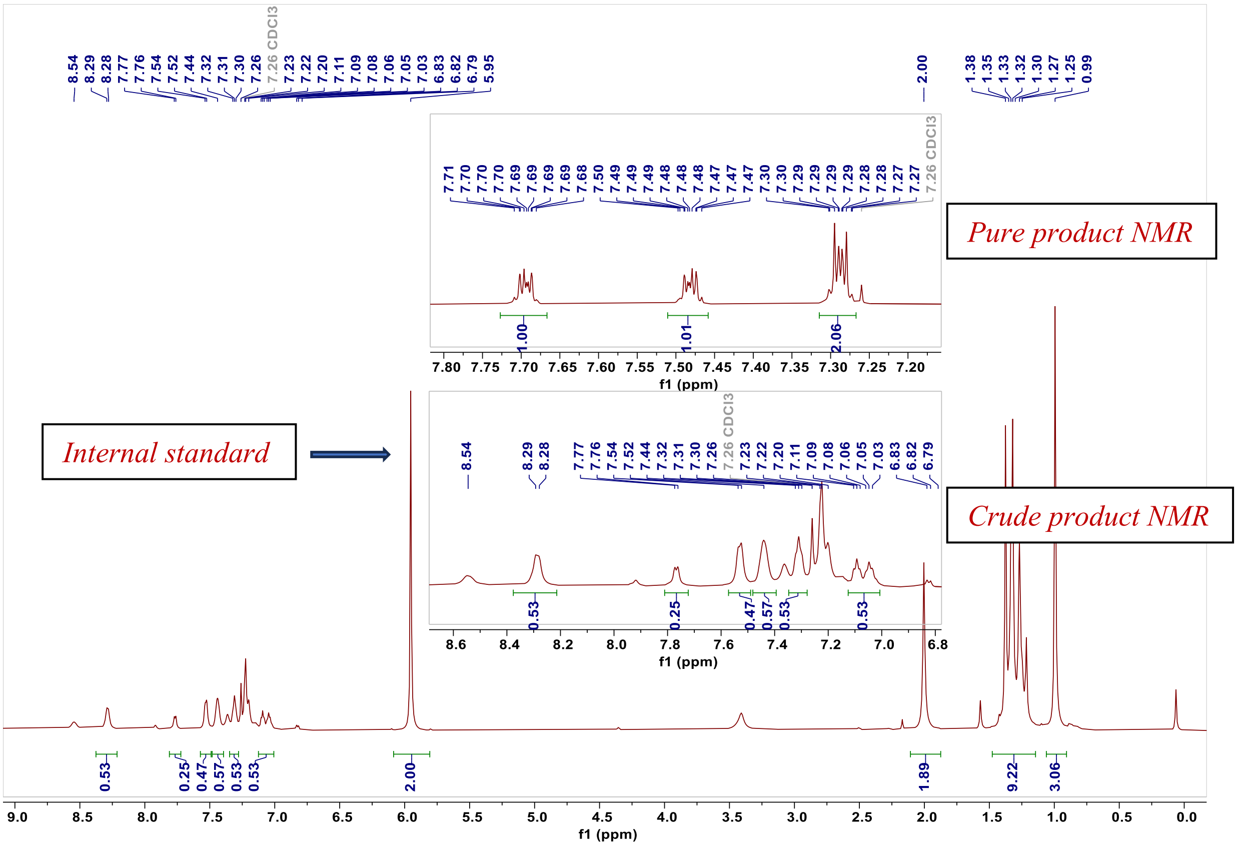

Figure 8.3.6.1: Crude ^{1}H NMR of cyclization

8.3.7 Synthesis of 2-(*tert*-butyl)benzo[*d*]oxazole (18) from BF$_2$ derivative (3a):

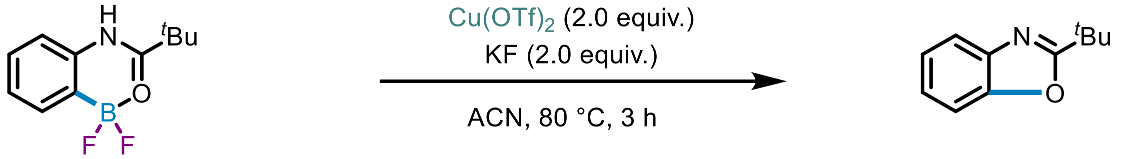

3a **18**, 63%

Procedure:[16] In a screw-capped 5 mL reaction vial equipped with a triangular Teflon-coated spinvane stir bar, the Ar-BF$_2$ **3a** (0.2 mmol, 1 equiv.) in 0.6 mL dry ACN was added with copper(II) triflate (2.0 equiv.) and KF (2.0 equiv.). The reaction mixture was heated at 80 ºC for 3 h. The reaction was allowed to reach room temperature and diluted with 5 mL ethyl acetate and filtered through a pad of celite and sodium sulfate. The celite pad was washed with 30 mL ethyl acetate and the filtrate was concentrated *in vacuo* to afford the crude product, which was purified using silica gel on automated column chromatography (pentane:EtOAc, 90:10).

Compound 18 spectral data:[16] colourless liquid, 22 mg (63%); **^{1}H NMR (600 MHz, CDCl$_3$) δ=** 7.73 – 7.67 (m, 1H), 7.51 – 7.46 (m, 1H), 7.32 – 7.27 (m, 2H), 1.49 (s, 9H).**^{13}C{^{1}H} NMR (151 MHz, CDCl$_3$) δ=** 173.6, 150.9, 141.4, 124.5, 124.1, 119.8, 110.4, 34.3, 28.6.

9. Single crystal X-ray diffraction

9.1-1 Single crystal X-ray diffraction of compound 3ag:

Single clear light colourless needle-shaped crystals of **3ag** was obtained by recrystallisation from ethanol/isopropanol mixture (1:1) at room temperature. A suitable crystal 0.28×0.04×0.02 mm^3 was selected and mounted on a suitable support on an XtaLAB Synergy R, HyPix diffractometer. The crystal was kept at a steady T = 152(3) K during data collection. The structure was solved with the ShelXT[11] structure solution program using the Intrinsic Phasing solution method and by using Olex2[12] as the graphical interface. The model was refined with version 2018/3 of ShelXL 2018/3[11] using Least Squares minimisation.

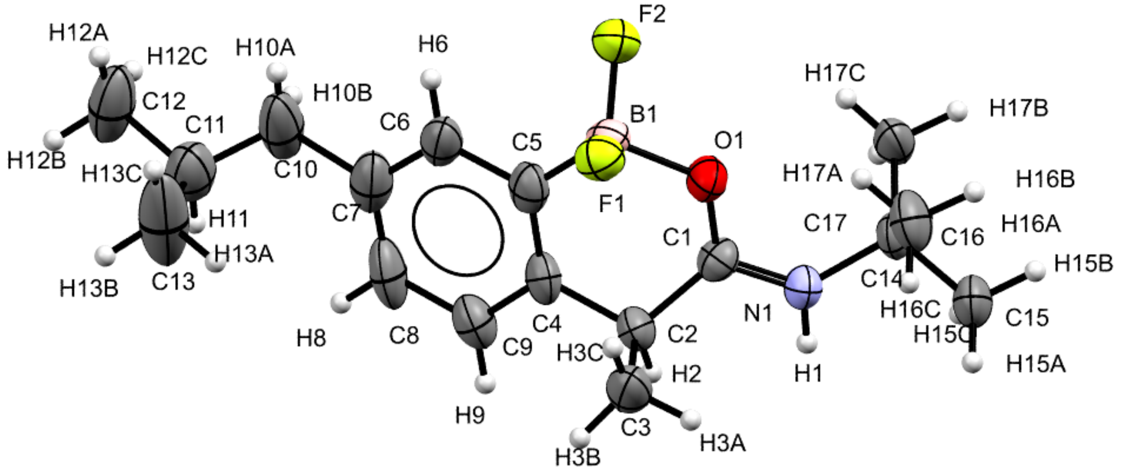

Figure 9.1-1. ORTEP of **3ag**: C (grey), N (purple), B (pink), O (red), F (green-yellow) and H (white) with 50% probability level. CCDC number: 2470008

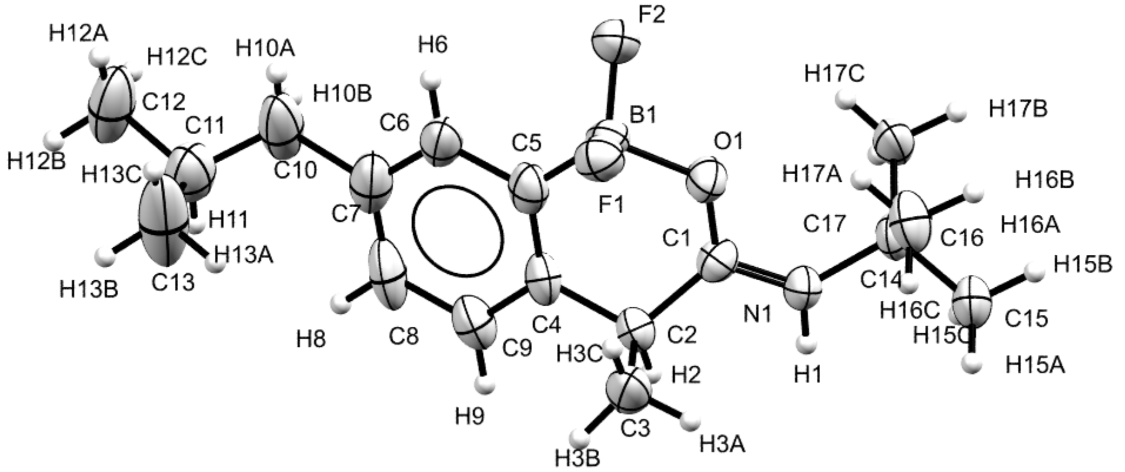

Table S3. Crystal data and structure refinement for compound **3ag**

**Compound**	**3ag**
Formula	$C_{17}H_{26}BF_2NO$
$D_{calc.}$/ g cm^{-3}	1.178
m/mm^{-1}	0.702
Formula Weight	309.20
Colour	clear light colourless
Shape	needle
Size/mm^3	0.28×0.04×0.02
T/K	152(3)
Crystal System	monoclinic
Space Group	$P2_1/n$
a/Å	6.5703(2)
b/Å	11.3767(3)
c/Å	23.3851(7)
α/°	90
β/°	94.413(3)
γ/°	90
V/Å^3	1742.81(9)
Z	4
Z'	1
Wavelength/Å	1.54184
Radiation type	Cu K_a
Θ_{min}/°	3.792
Θ_{max}/°	75.374
Measured Refl.	16456
Independent Refl.	3484
Reflections with I > 2(I)	2672
R_{int}	0.0409
Parameters	205
Restraints	0
Largest Peak	0.258
Deepest Hole	-0.242
GooF	1.067
wR_2 (all data)	0.1250
wR_2	0.1087
R_1 (all data)	0.0631
R_1	0.0452

9.1-2 Single crystal X-ray diffraction of compound 3ag:

Single light colourless crystals of **3ag** was obtained by recrystallisation from isopropanol. A suitable crystal 0.172 × 0.036 × 0.029 mm^3 was selected and mounted on a suitable support on an XtaLAB Synergy R, HyPix diffractometer. The crystal was kept at a steady T = 149.99(10)K during data collection. The structure was solved with the ShelXT[11] structure solution program using the Intrinsic Phasing solution method and by using Olex2[12] as the graphical interface. The model was refined with version 2018/3 of ShelXL 2018/3[11] using Least Squares minimisation.

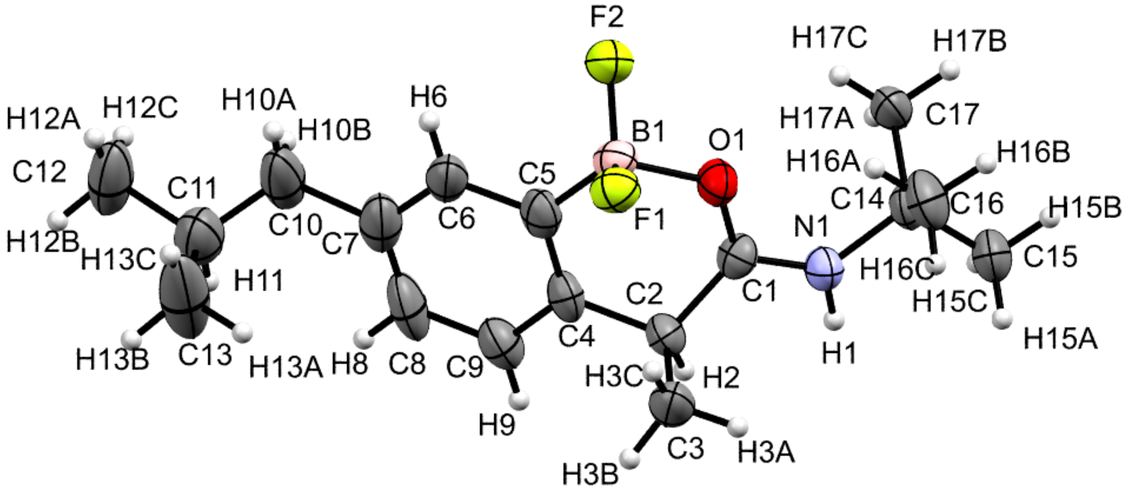

Figure 9.1-2. ORTEP of **3ag**: C (grey), N (purple), B (pink), O (red), F (green-yellow) and H (white) with 50% probability level. CCDC number: 2470007

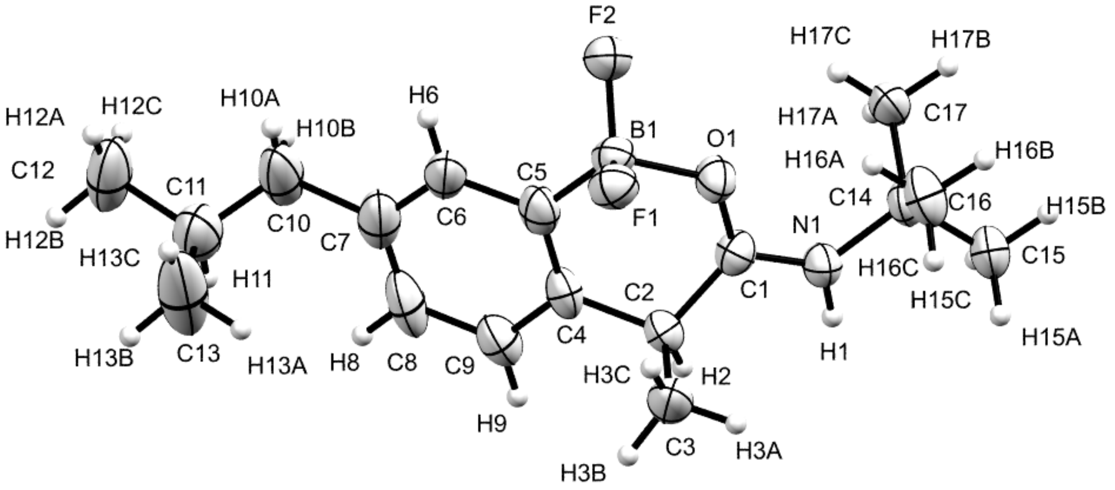

Table S4. Crystal data and structure refinement for compound **3ag**

**Compound**	**3ag**
Formula	$C_{17}H_{26}BF_2NO$
$D_{calc.}$/ g cm^{-3}	1.180
μ/mm^{-1}	0.703
F(000)	664.0
Formula Weight	309.20
Colour	clear light colourless
Shape	block
Size/mm^3	0.172 × 0.036 × 0.029
T/K	149.99(10)
Crystal System	monoclinic
Space Group	$P2_1/c$
a/Å	6.5230(5)
b/Å	13.7796(13)
c/Å	19.3828(19)
α/°	90
β/°	92.919(9)
γ/°	90
V/Å^3	1739.9(3)
Z	4
Wavelength/Å	1.54184
Radiation type	Cu Kα (λ = 1.54184)
Θ_{min}/°	7.876
Θ_{max}/°	157.062
Index ranges	$-7 \le h \le 7, 0 \le k \le 16, 0 \le l \le 24$
Reflections collected	3454
Independent reflections	3454 [R_{sigma} = 0.0798]
Data/restraints/parameters	3454/0/206
Goodness-of-fit on F^2	1.077
Final R indexes [I>=2σ (I)]	R_1 = 0.1048, wR_2 = 0.2777
Final R indexes [all data]	R_1 = 0.1727, wR_2 = 0.3298
Largest diff. peak/hole / e Å^{-3}	0.53/-0.45

9.2 Single crystal X-ray diffraction of compound 3at:

Single colourless block-shaped crystals of **3at** was obtained by recrystallisation from ethanol. A suitable crystal 0.20×0.15×0.05 mm^3 was selected and mounted on a suitable support on an XtaLAB Synergy R, HyPix diffractometer. The crystal was kept at a steady T = 154.34(10) K during data collection. The structure was solved with the ShelXT[11] structure solution program using the Intrinsic Phasing solution method and by using Olex2[12] as the graphical interface. The model was refined with version 2018/3 of ShelXL 2018/3[11] using Least Squares minimisation.

Ellipsoid

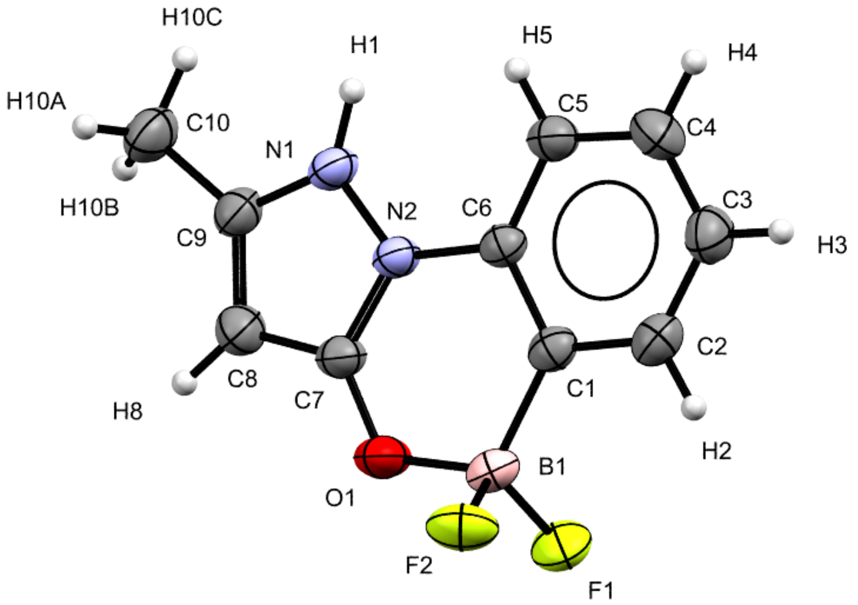

Figure 9.2-1. ORTEP of **3at**: C (grey), N (purple), O (red), B (pink), F (green-yellow) and H (white) with 50% probability level. CCDC number: 2470009

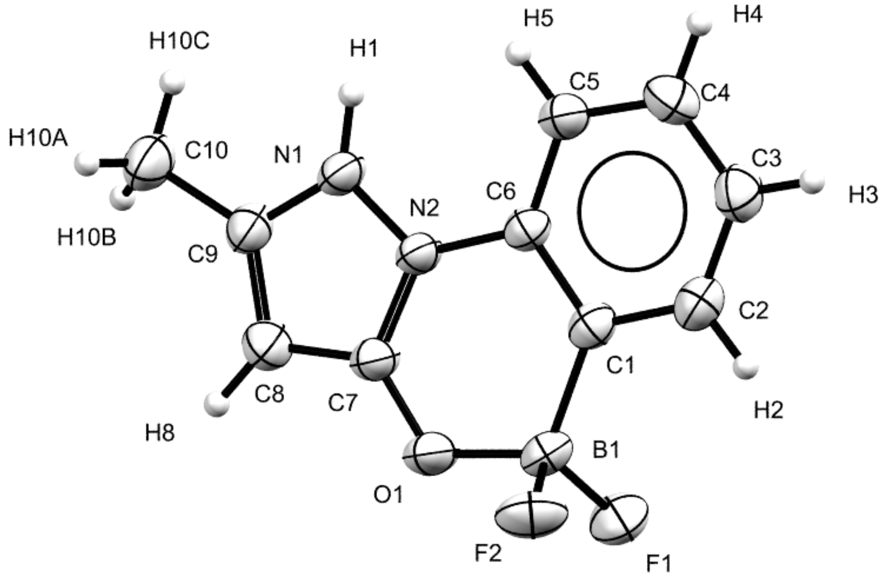

Table S5. Crystal data and structure refinement for compound **3at**

**Compound**	**3at**
Formula	$C_{10}H_9BF_2N_2O$
$D_{calc.}$/ g cm^{-3}	1.476
μ/mm^{-1}	1.038
Formula Weight	222
Colour	colourless
Shape	block
Size/mm^3	0.20×0.15×0.05
T/K	154.34(10)
Crystal System	monoclinic
Space Group	$P2_1/c$
a/Å	7.3989(3)
b/Å	9.2048(4)
c/Å	14.9507(6)
α/°	90
β/°	101.056(4)
γ/°	90
V/Å^3	999.32(7)
Z	4
Z'	1
Wavelength/Å	1.54184
Radiation type	Cu K_a
Θ_{min}/°	5.674
Θ_{max}/°	74.918
Measured Refl.	9430
Independent Refl.	1986
Reflections with I > 2(I)	1682
R_{int}	0.0387
Parameters	150
Restraints	0
Largest Peak	0.241
Deepest Hole	-0.231
GooF	1.067
wR_2 (all data)	0.1212
wR_2	0.1095
R_1 (all data)	0.0515
R_1	0.0426

9.3 Single crystal X-ray diffraction of compound 3ae:

Single clear light colourless prism-shaped crystals of **3ae** was obtained by recrystallisation from ethanol. A suitable crystal 0.24×0.08×0.04 mm^3 was selected and mounted on a suitable support on an XtaLAB Synergy R, HyPix diffractometer. The crystal was kept at a steady T = 154.20(15) K during data collection. The structure was solved with the ShelXT[11] structure solution program using the Intrinsic Phasing solution method and by using Olex2[12] as the graphical interface. The model was refined with version 2018/3 of ShelXL 2018/3[11] using Least Squares minimisation.

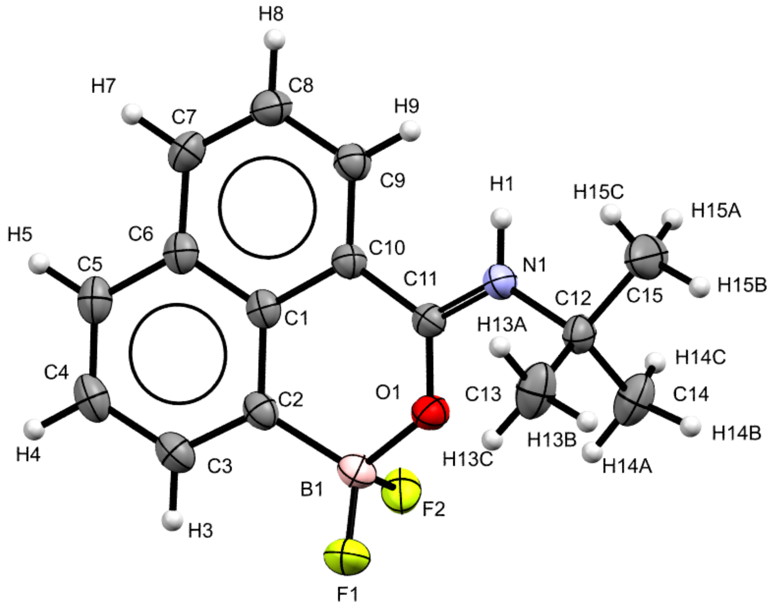

Figure 9.3-1. ORTEP of **3ae**: C (grey), N (purple), B (pink), O (red), F (green-yellow) and H (white) with 50% probability level. CCDC number: 2470010.

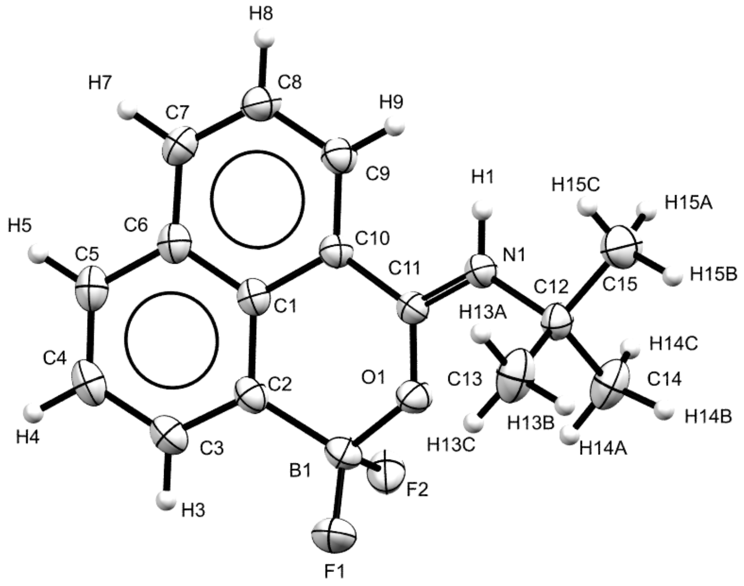

Table S6. Crystal data and structure refinement for compound **3ae**

**Compound**	**3ae**
Formula	$C_{15}H_{16}BF_2NO$
$D_{calc.}$/ g cm^{-3}	1.337
μ/mm^{-1}	0.841
Formula Weight	275.10
Colour	clear light colourless
Shape	prism
Size/mm^3	0.24×0.08×0.04
T/K	154.20(15)
Crystal System	triclinic
Space Group	*P*1
a/Å	7.16860(18)
b/Å	10.1326(3)
c/Å	10.2244(3)
α/°	106.624(2)
β/°	102.662(2)
γ/°	95.804(2)
V/Å^3	683.57(3)
Z	2
Z'	1
Wavelength/Å	1.54184
Radiation type	Cu K_a
Θ_{min}/°	4.626
Θ_{max}/°	75.012
Measured Refl.	12361
Independent Refl.	2673
Reflections with I > 2(I)	2310
R_{int}	0.0312
Parameters	185
Restraints	0
Largest Peak	0.285
Deepest Hole	-0.200
GooF	1.061
wR_2 (all data)	0.1217
wR_2	0.1085
R_1 (all data)	0.0473
R_1	0.0400

9.4 Single crystal X-ray diffraction of compound 3aq:

Single clear light colourless crystals of **3aq** was obtained by recrystallisation from ACN:$CHCl_3$. A suitable crystal 0.202×0.106×0.05 mm^3 was selected and the data was acquired on a Rigaku XtaL AB Synergy-DW diffractometer equipped with a HyPix-Arc 150 ⁰ detector using Cu Kα radiation with λ = 1.54184 Å. The structure was solved with the ShelXT[11] structure solution program using the Intrinsic Phasing solution method and by using Olex2[12] as the graphical interface. The model was refined with version 2018/3 of ShelXL 2018/3[11] using Least Squares minimisation.

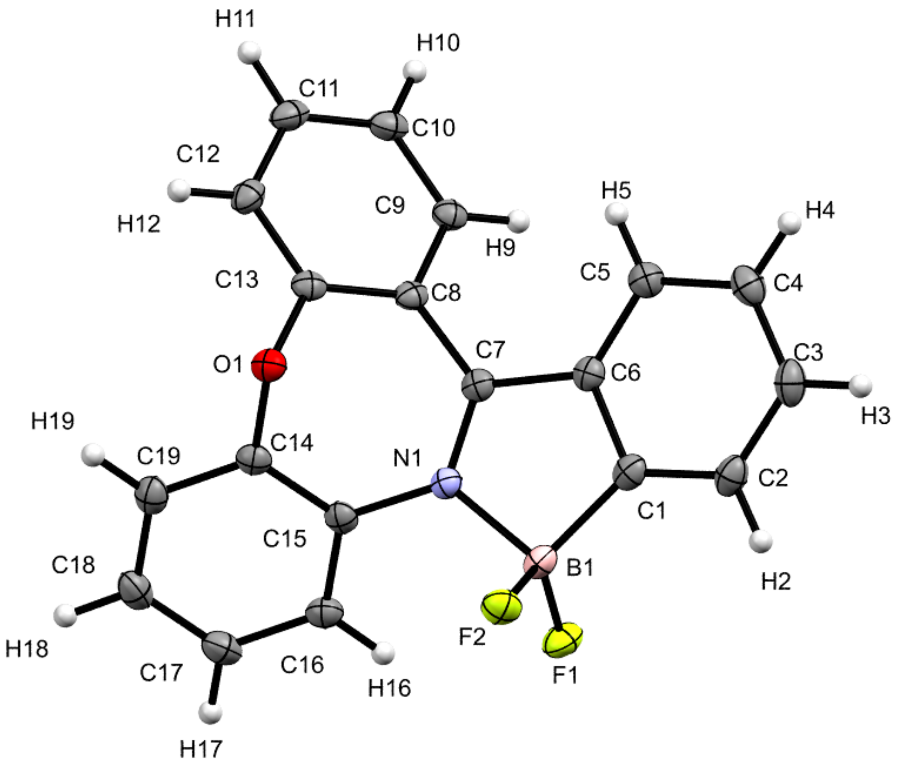

Figure 9.4.1. ORTEP of **3aq**: C (grey), N (purple), O (red), B (pink), F (green-yellow) and H (white) with 50% probability level. CCDC number: 2496224

Table S7. Crystal data and structure refinement for compound **3aq**

**Compound**	**3aq**
Formula	$C_{19}H_{12}BF_2NO$
$D_{calc.}$/ g cm^{-3}	1.484
μ/mm^{-1}	0.905
Formula Weight	319.11
Size/mm^3	0.202 × 0.106 × 0.05
T/K	111.00(13)
F(000)	328.0
Crystal System	triclinic
Space Group	*P*1
a/Å	8.71090(10)
b/Å	9.78640(10)
c/Å	10.26780(10)
α/°	112.8990(10)
β/°	94.9800(10)
γ/°	112.7170(10)
V/Å^3	714.244(15)
Z	2
Index ranges	-10 ≤ h ≤ 10, -12 ≤ k ≤ 10, -12 ≤ l ≤ 12
Wavelength/Å	1.54184
Radiation type	Cu K_a
Θ_{min}/°	9.738
Θ_{max}/°	150.248
Measured Refl.	26502
Independent Refl.	2849 [R_{int} = 0.0334, R_{sigma} = 0.0150]
Parameters	217
Restraints	0
Largest Peak	0.27
Deepest Hole	-0.26
GooF	1.036
Final R indexes [I>=2σ (I)]	R_1 = 0.0359, wR_2 = 0.0985
Final R indexes [all data]	R_1 = 0.0379, wR_2 = 0.1006

10. References

1) J. Lv, X. Chen, X. S. Xue, B. Zhao, Y. Liang, M. Wang, L. Jin, Y. Yuan, Y. Han, Y. Zhao, Y. Lu, J. Zhao, W. Y. Sun, K. N. Houk, Z. Shi, *Nature* **2019**, *575*, 336–340

2) G. H. Shinde, G. S. Ghotekar, F. M. Amombo Noa, L. Öhrström, P.-O, Norrby, H. Sundén, *Chem. Sci.* **2023**, *14*, 13429–13436.

3) S. Maji, P. Rawal, A. Ghosh, K. Pidiyar, S. A. Al-Thabaiti, P. Gupta, D. Maiti, D. *JACS Au*, **2024**, *4*, 3679−3689.

4) J. Shang, W. Si, W. Zhao, Y. Che, J. L. Hou, H. Jiang, *Org. Lett*. **2014**, *16*, 4008–4011.

5) J. Lv, B. Zhao, Y. Yuan, Y. Han, Z. Shi, Z. *Nat. Commun.* **2020**, *11*, 1316.

6) J. F. Cui, H. Huang, H. N. C. Wong, *Synlett* **2011**, *7*, 1018–1022.

7) G. H. Shinde, G. S. Ghotekar, H. Sundén, *Chem. Eur. J.* **2025**, *31*, e202403938

8) G. H. Shinde, H. Castlind, G. S. Ghotekar, F. M. Amombo Noa, L. Öhrström, H. Sundén, *Org. Lett*. **2025**, *27*, 207–211.

9) Crysalis CCD; Oxford Diffraction Ltd: Abingdon, Oxfordshire, UK, **2005**.

10) Crysalis RED; Oxford Diffraction Ltd: Abingdon, Oxfordshire, UK, **2005**.

11) G. M. Sheldrick, *Acta Crystallogr., Sect. C: Struct. Chem.* **2015**, *71*, 3– 8.

12) O.V. Dolomanov, L. J. Bourhis, R. J. Gildea, J. A. K. Howard, H. Puschmann, *J. Appl. Crystallogr*. **2009**, *42*, 339– 341.

13) C.F. Macrae, C. F.; I. J. Bruno, J. A. Chisholm, P. R. Edgington, P. McCabe, E. Pidcock, L. Rodriguez-Monge, R. Taylor, J. Van De Streek, P. A. Wood, *J. Appl. Crystallogr*. **2008**, *41*, 466-470.

14) M. E. Hoque, M. M. M. Hassan, B. Chattopadhyay, *J. Am. Chem. Soc*. **2021**, *143*, 5022–5037.

15) J. Morin, Y. Zhao, V. Snieckus, *Org. Lett.* **2013**, *15*, 4102-4105.

16) S. Ueda, H. Nagasawa, *J. Org. Chem.* **2009**, *74*, 4272–4277.

11. NMR spectra of target compounds

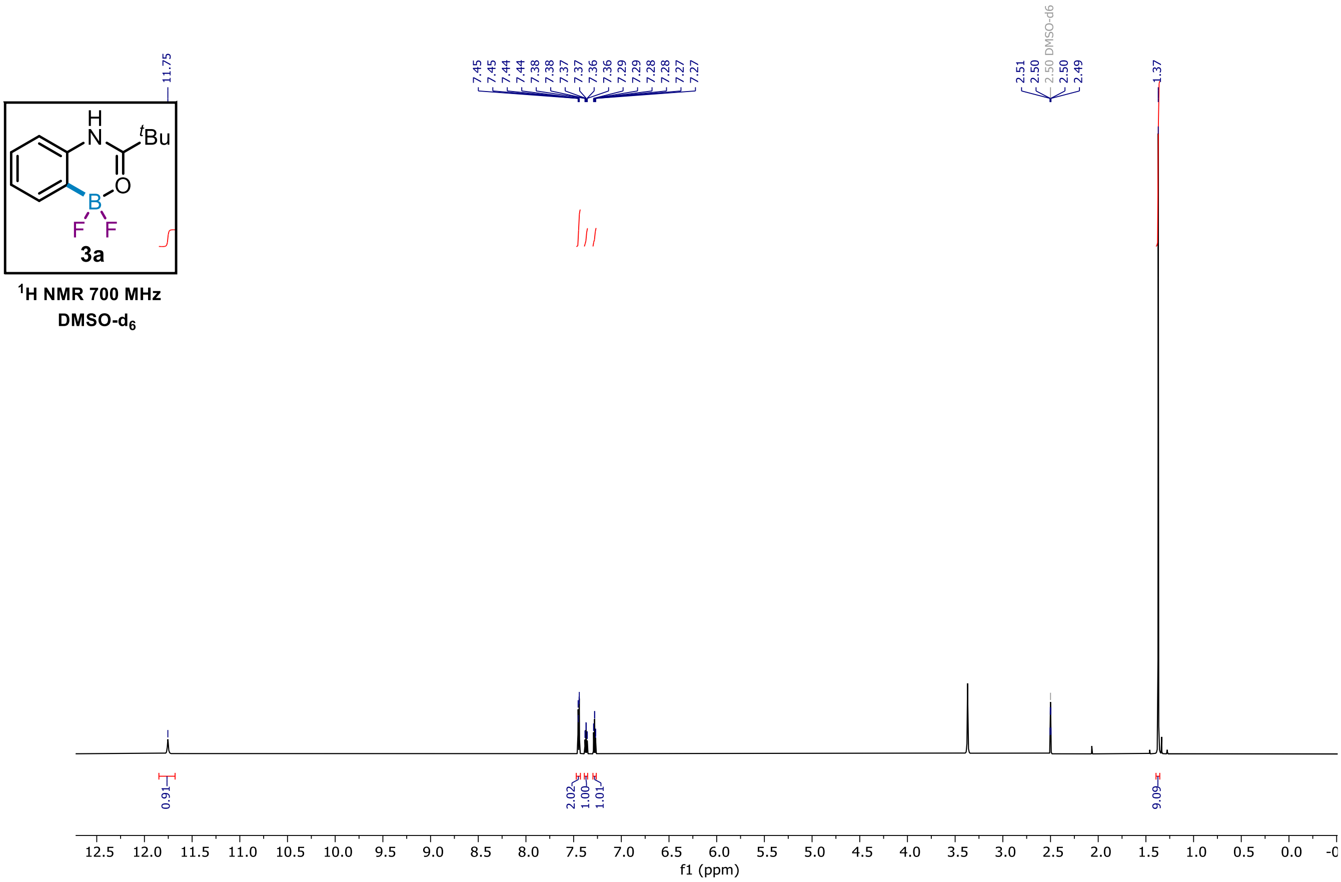

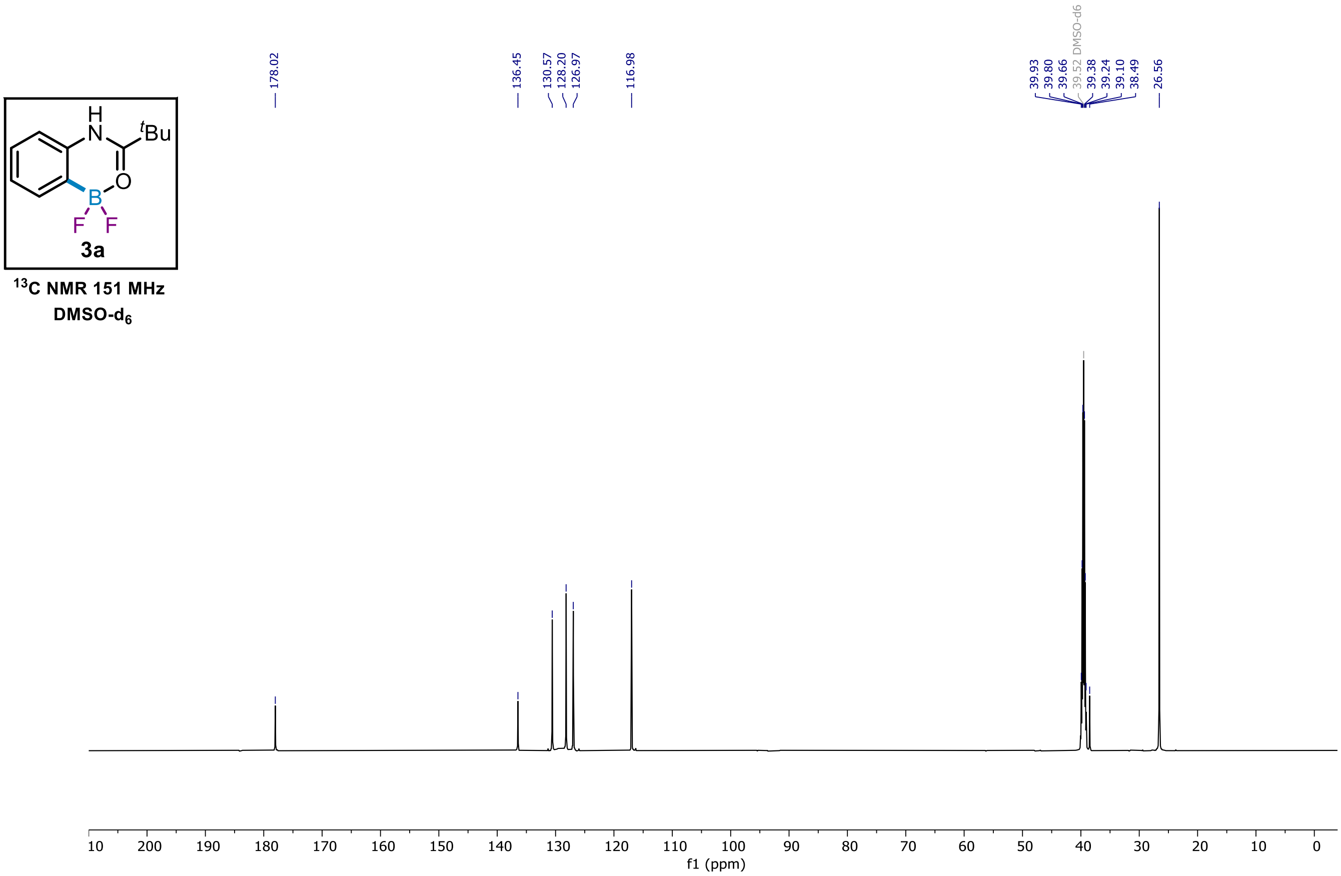

Figure S11-1: ^{13}C spectrum of compound **3a** in DMSO-d_6. Note that the ^{13}C signal for the C-BF_2 bond does not appear.

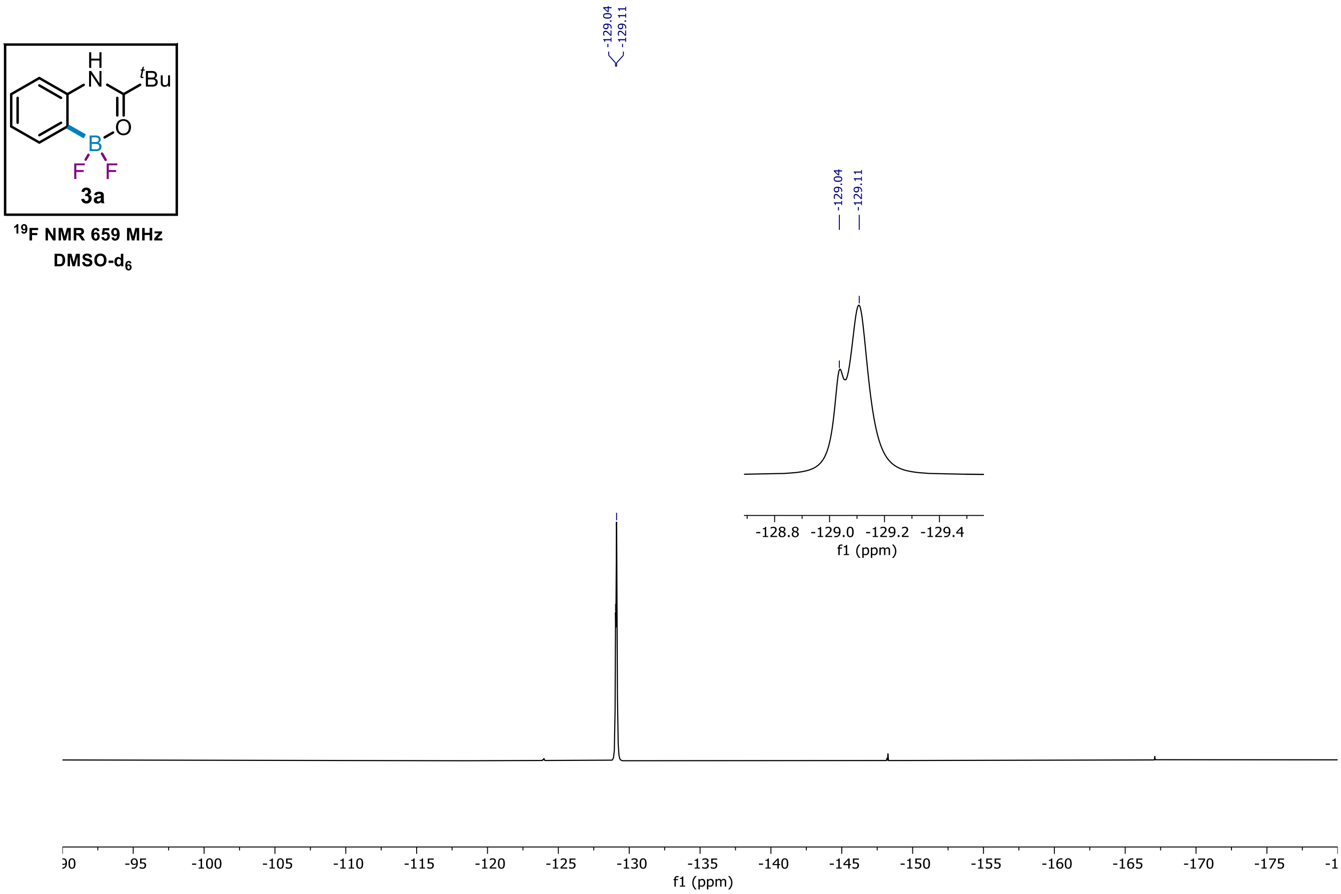

^{19}F NMR 659 MHz DMSO-d$_6$

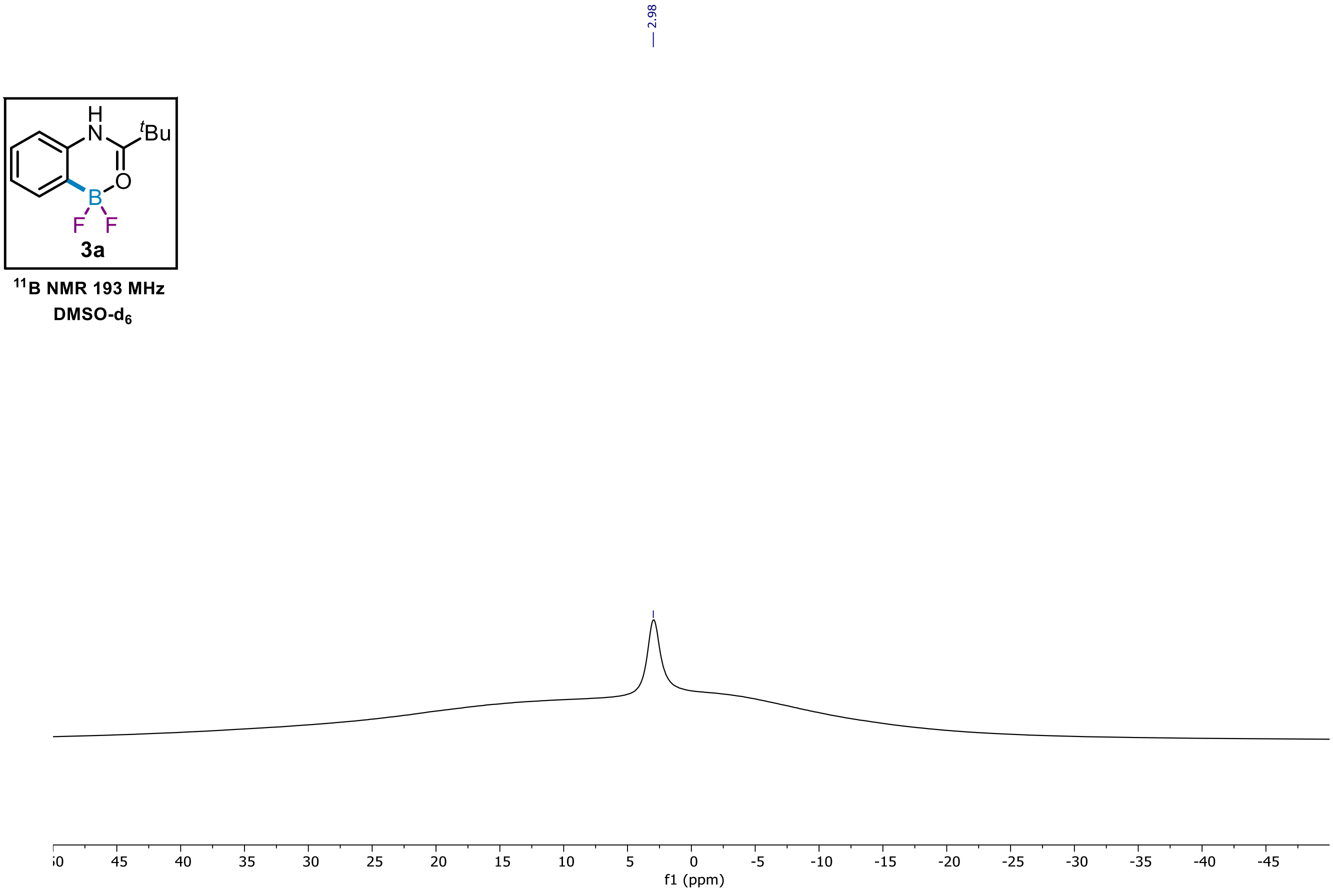

^{11}B NMR 193 MHz DMSO-d$_6$

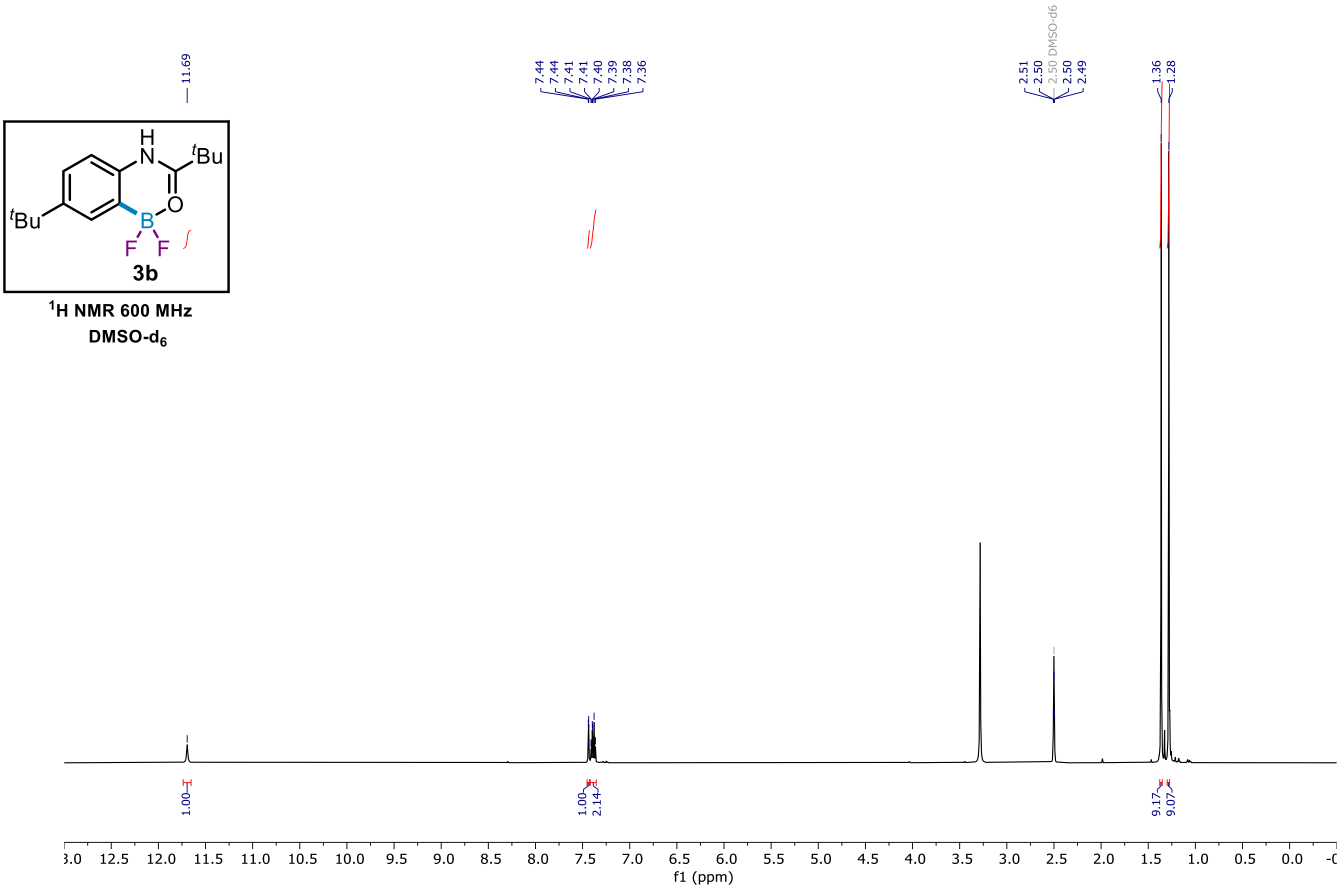

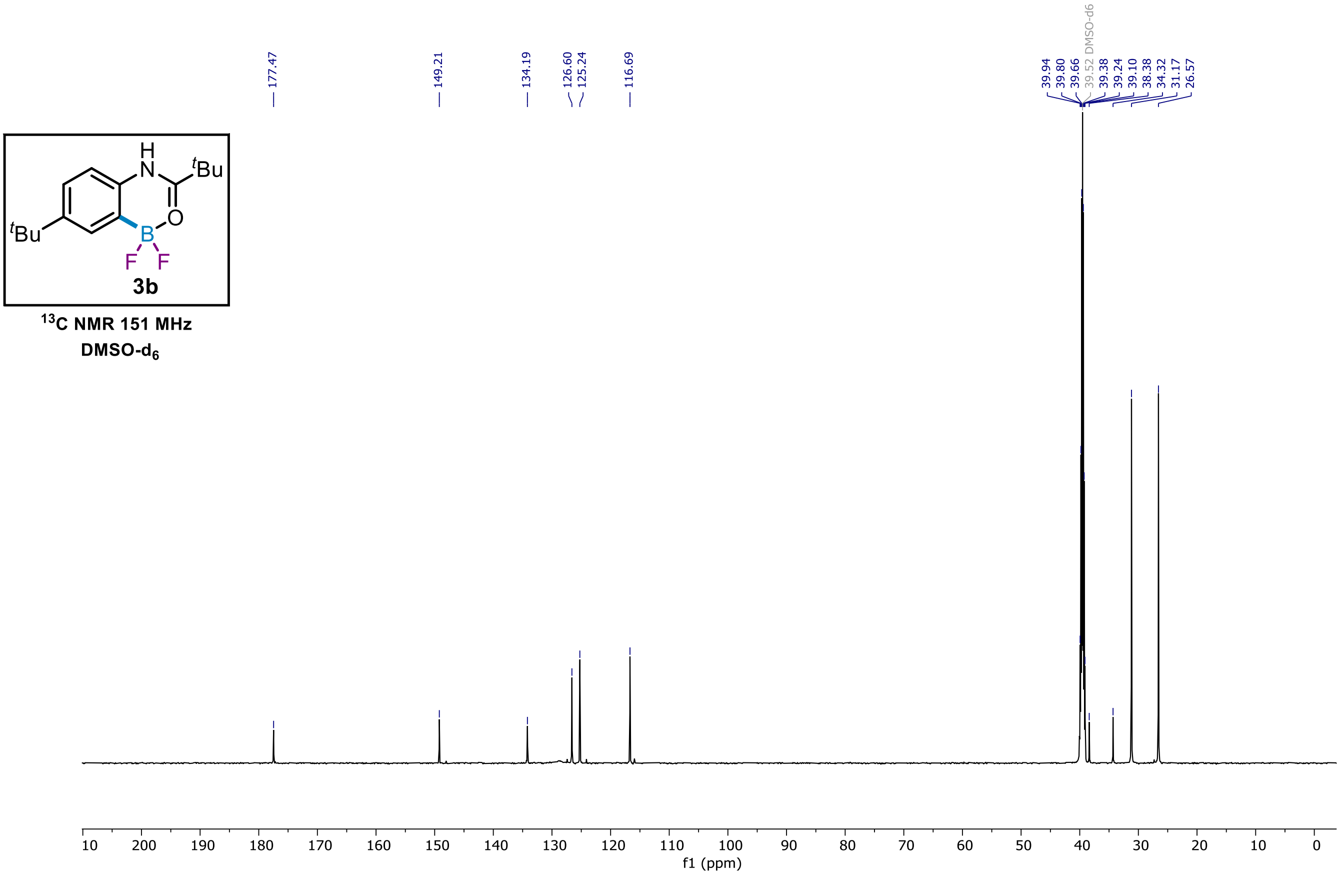

Figure S11-2: ^{13}C spectrum of compound **3b** in DMSO-d$_6$. Note that the ^{13}C signal for the C-BF$_2$ bond does not appear.

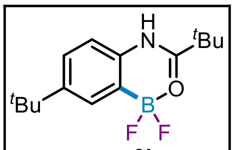

3b

^{19}F NMR 659 MHz
DMSO-d$_6$

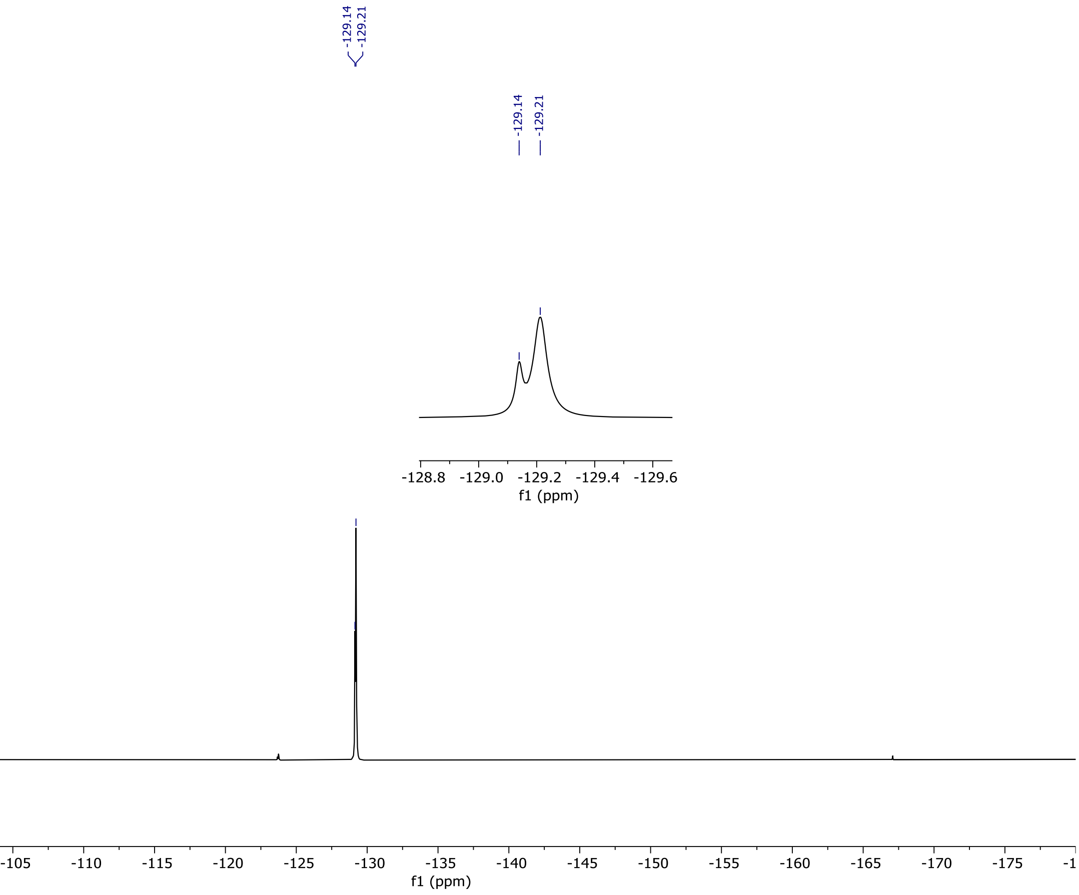

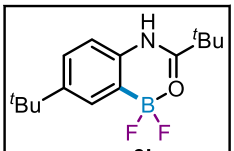

3b

^{11}B NMR 193 MHz
DMSO-d$_6$

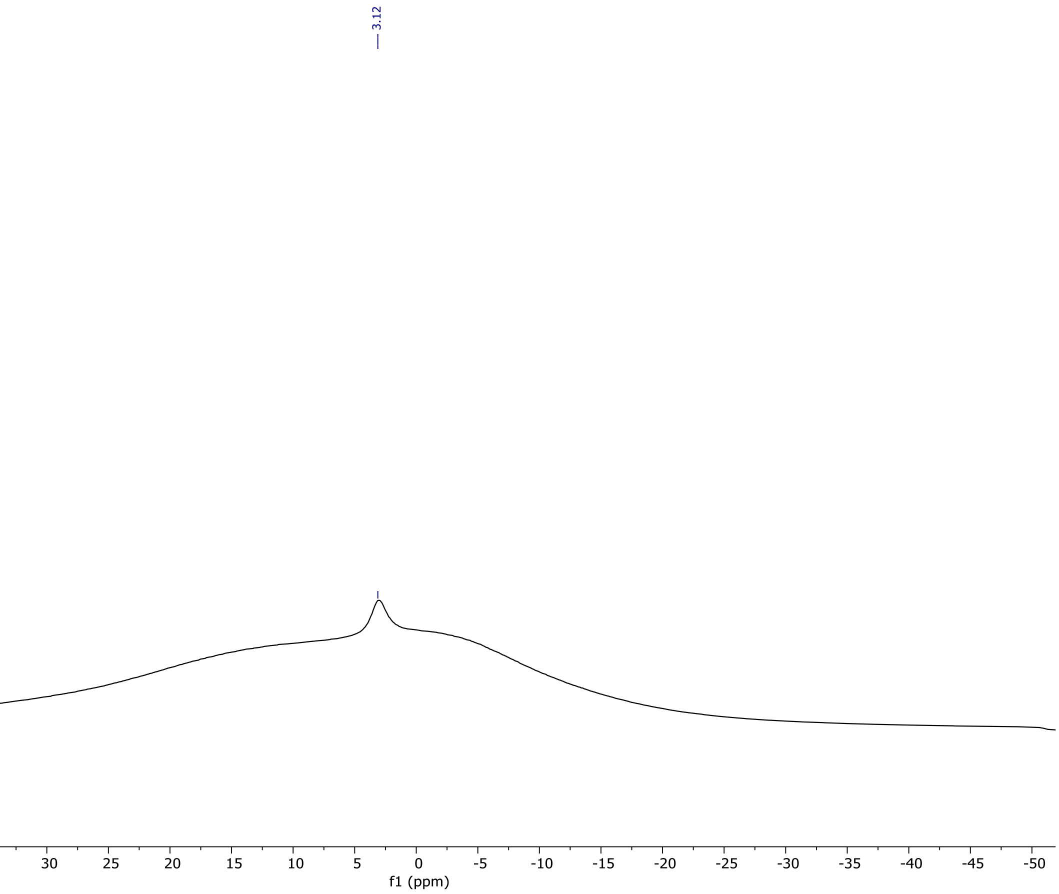

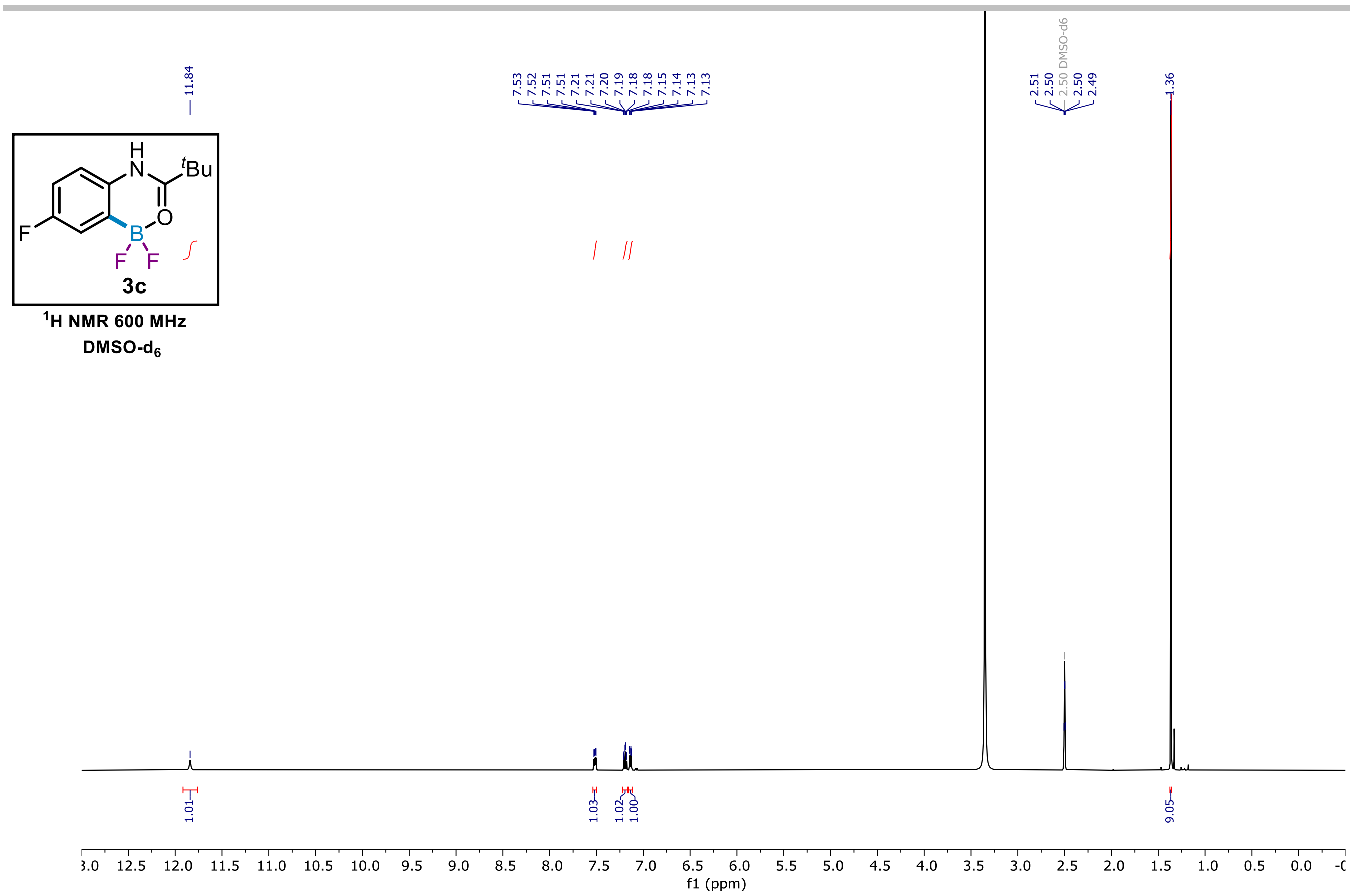

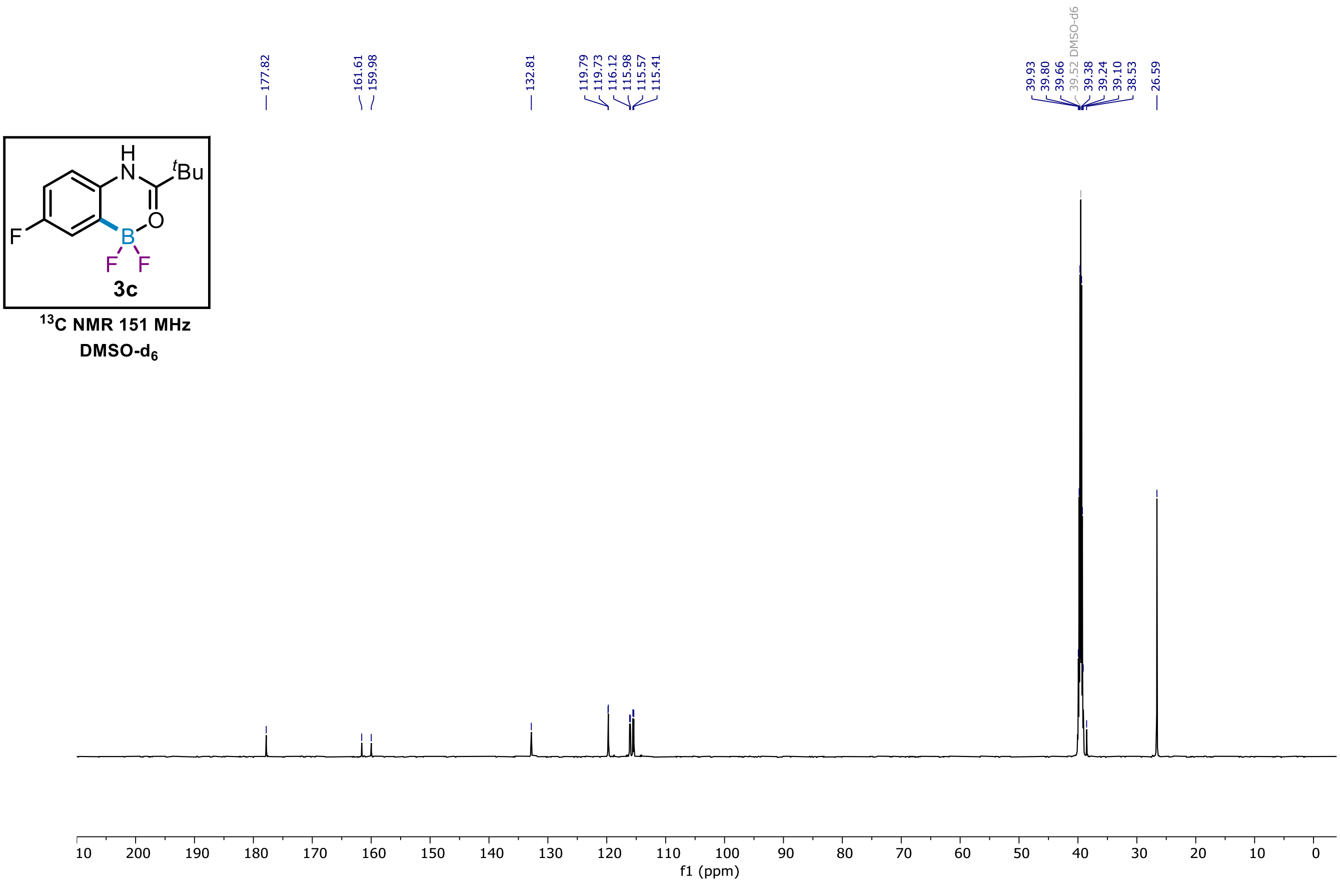

Figure S11-3: ^{13}C spectrum of compound **3c** in DMSO-d$_6$. Note that the ^{13}C signal for the C-BF$_2$ bond does not appear.

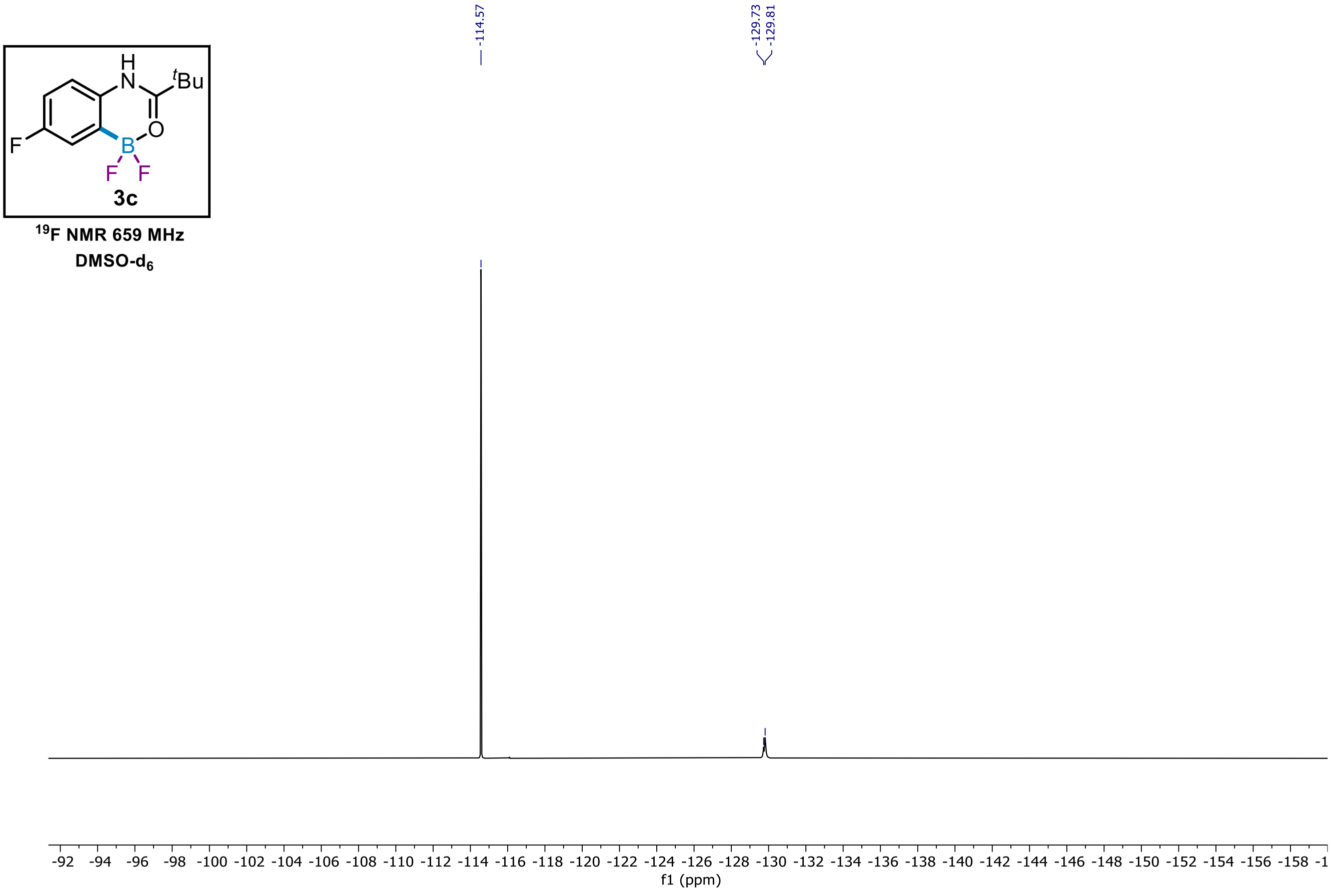
H
N
tBu
O
F
B
F F
3c
^{19}F NMR 659 MHz
DMSO-d_6
-114.57
-129.73
-129.81
-92 -94 -96 -98 -100 -102 -104 -106 -108 -110 -112 -114 -116 -118 -120 -122 -124 -126 -128 -130 -132 -134 -136 -138 -140 -142 -144 -146 -148 -150 -152 -154 -156 -158 -1
f1 (ppm)

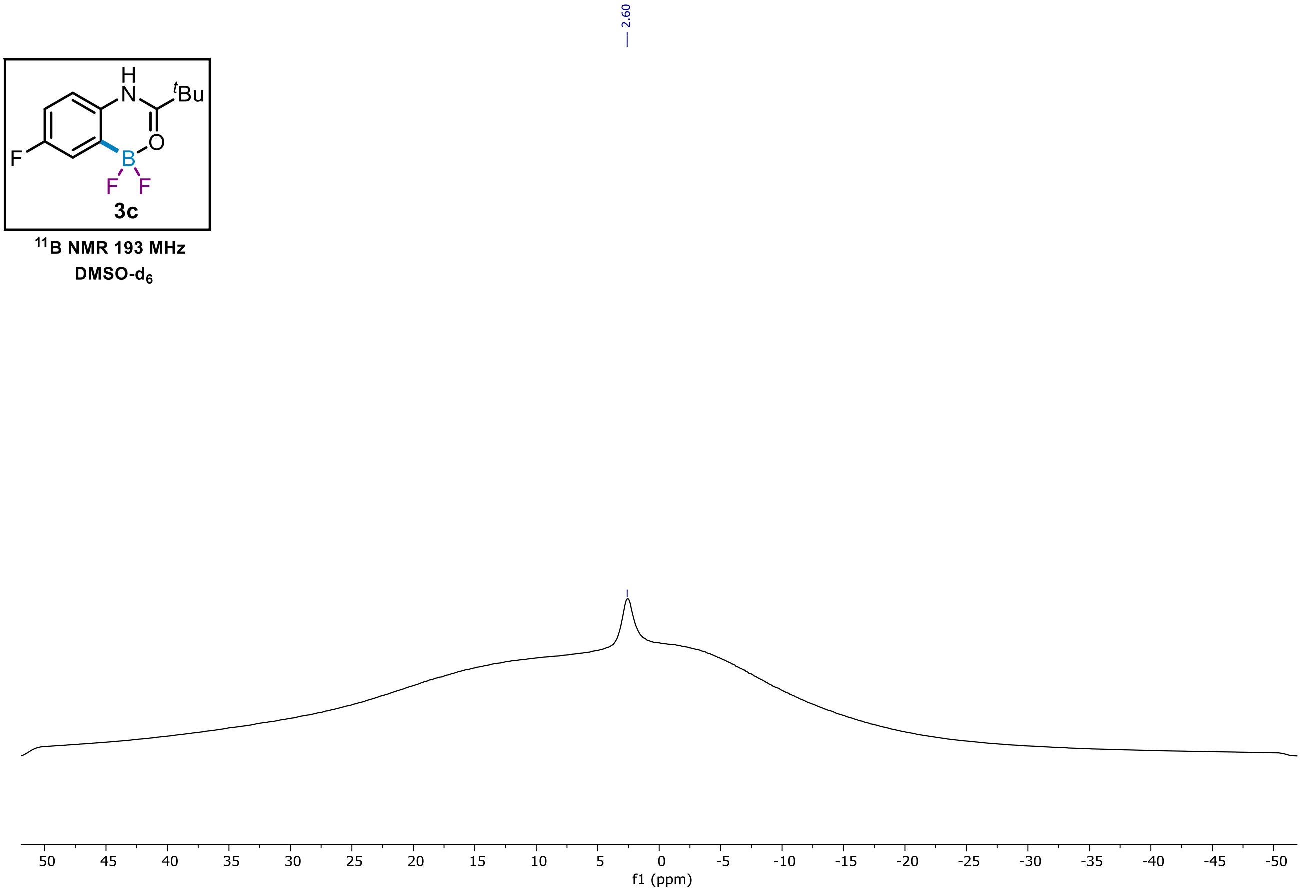
H
N
tBu
O
F
B
F F
3c
^{11}B NMR 193 MHz
DMSO-d_6
2.60
50 45 40 35 30 25 20 15 10 5 0 -5 -10 -15 -20 -25 -30 -35 -40 -45 -50
f1 (ppm)

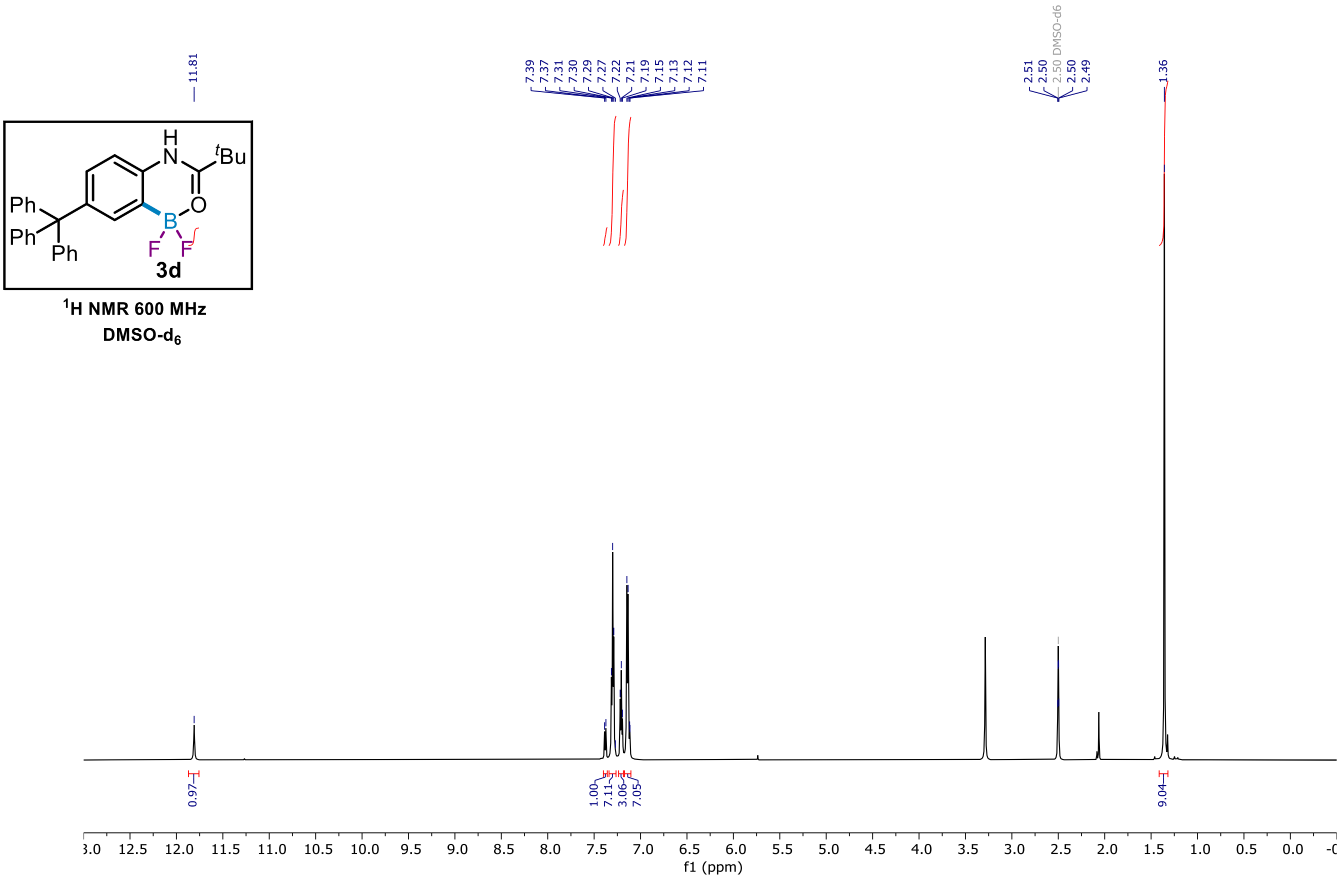

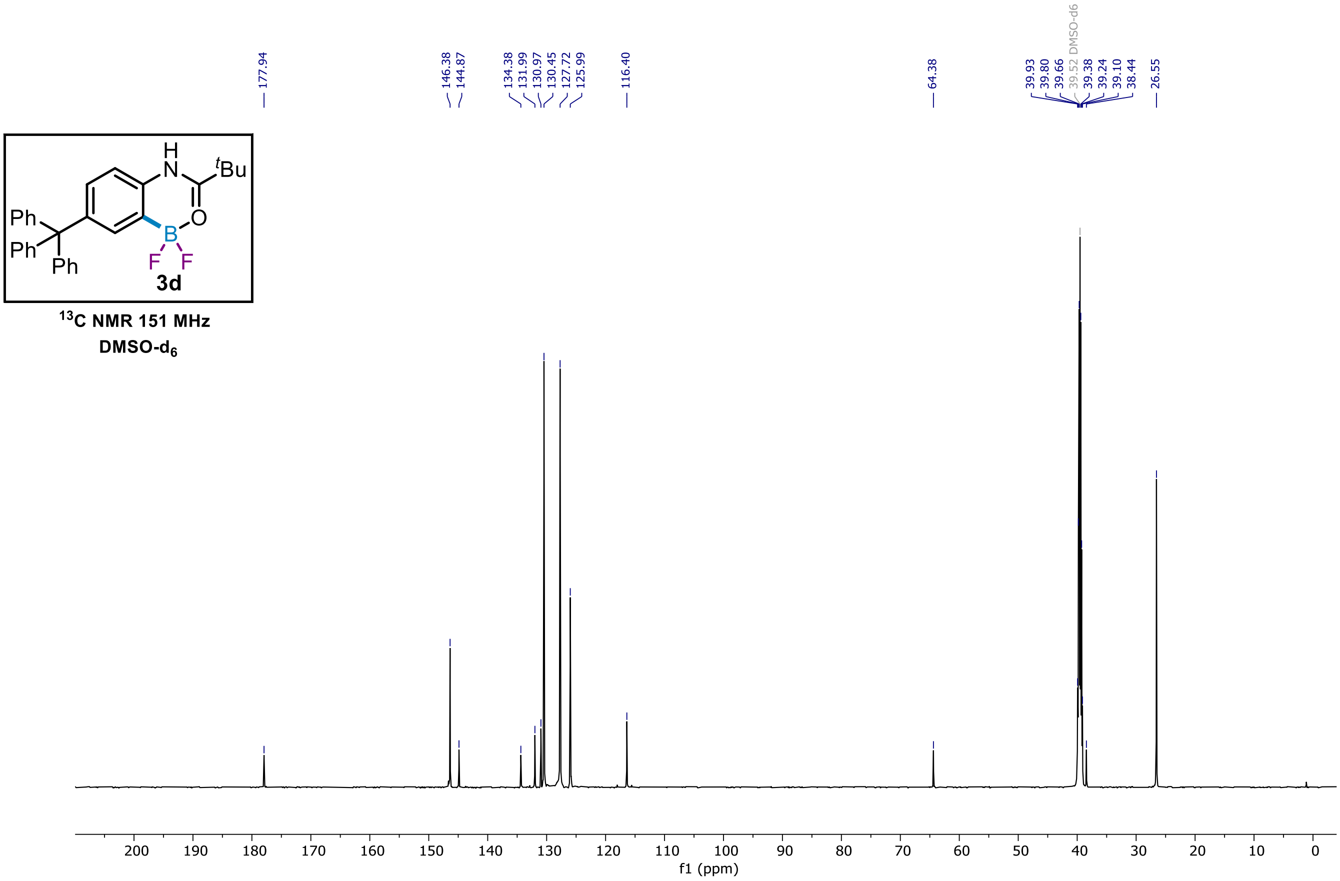

Figure S11-4: ^{13}C spectrum of compound **3d** in DMSO-d_6. Note that the ^{13}C signal for the C-BF_2 bond does not appear.

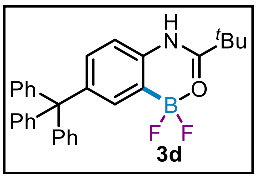

^{19}F NMR 659 MHz
DMSO-d_6

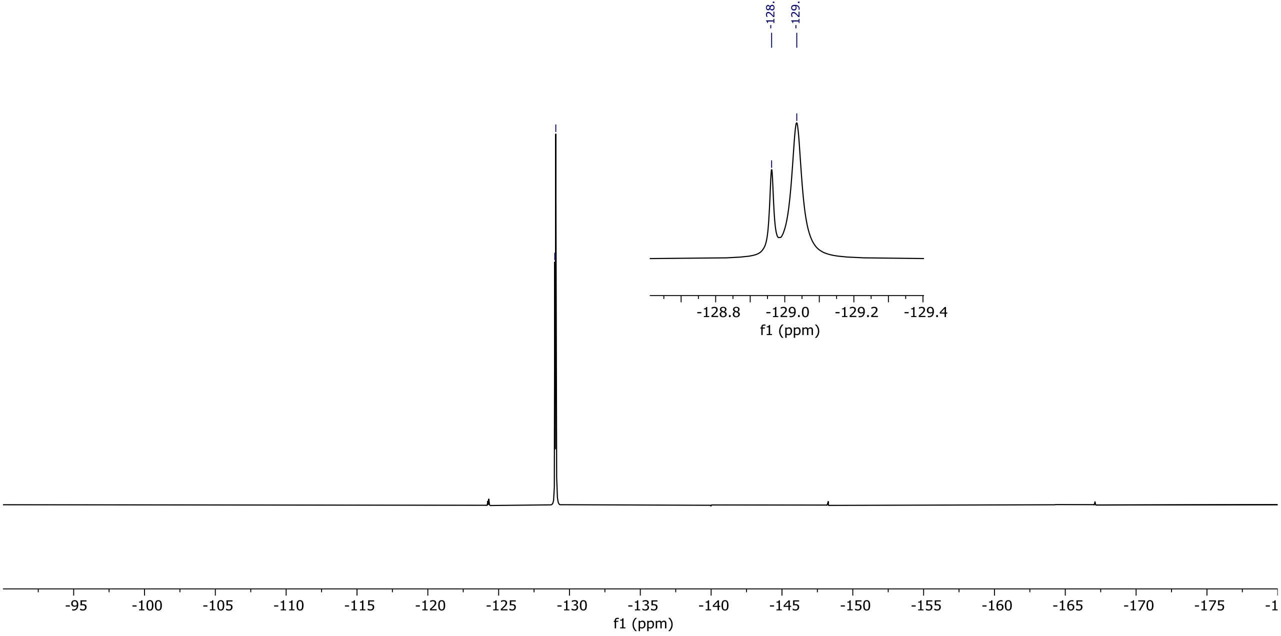

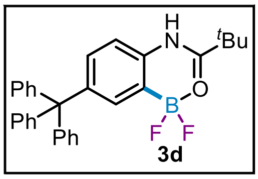

^{11}B NMR 193 MHz
DMSO-d_6

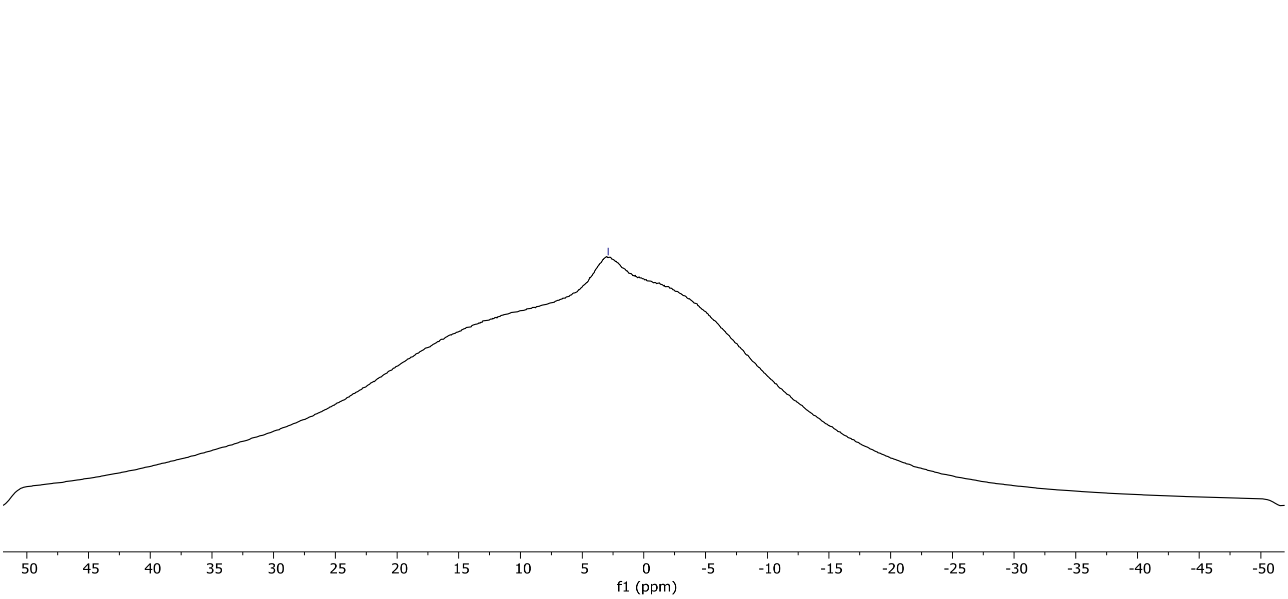

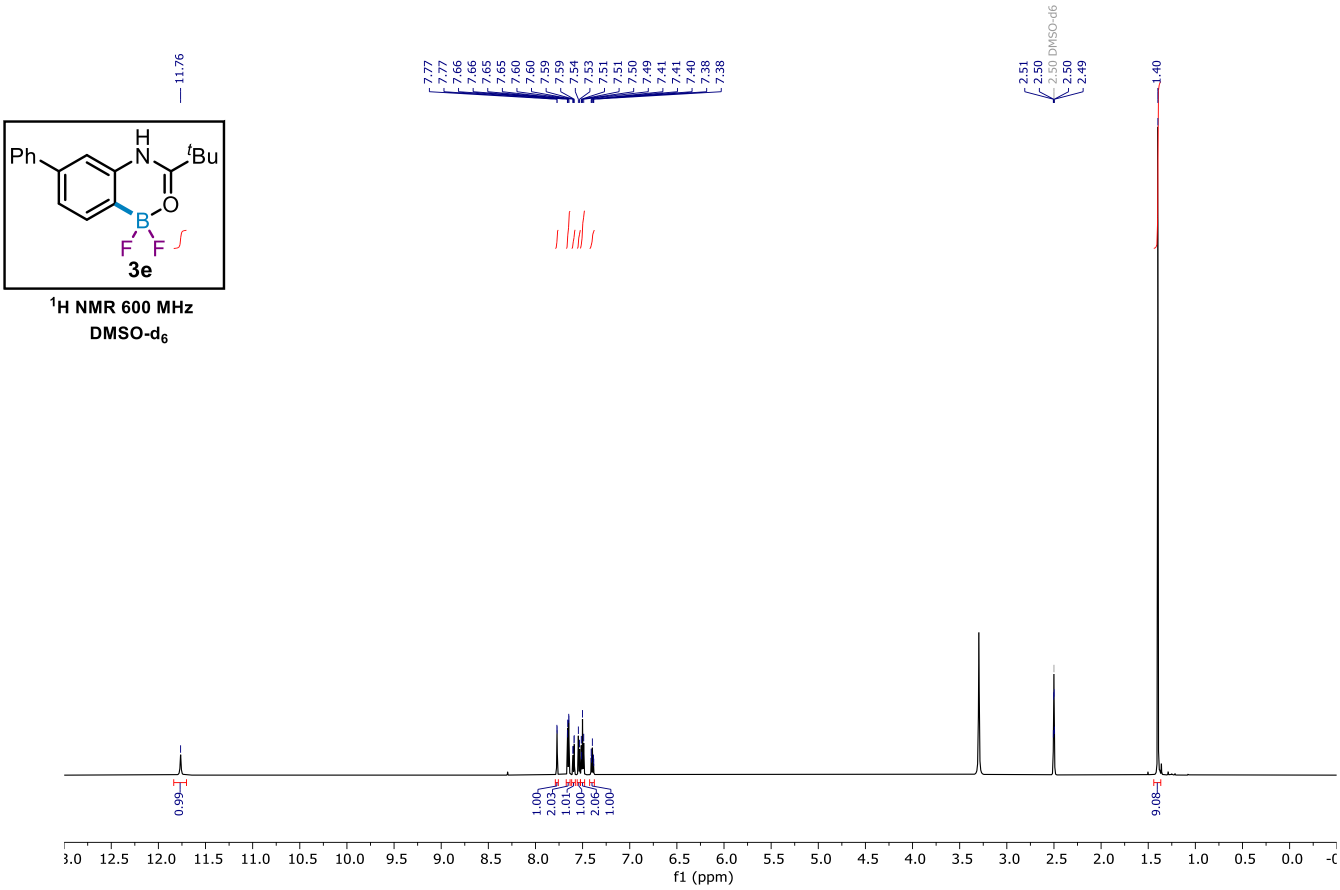

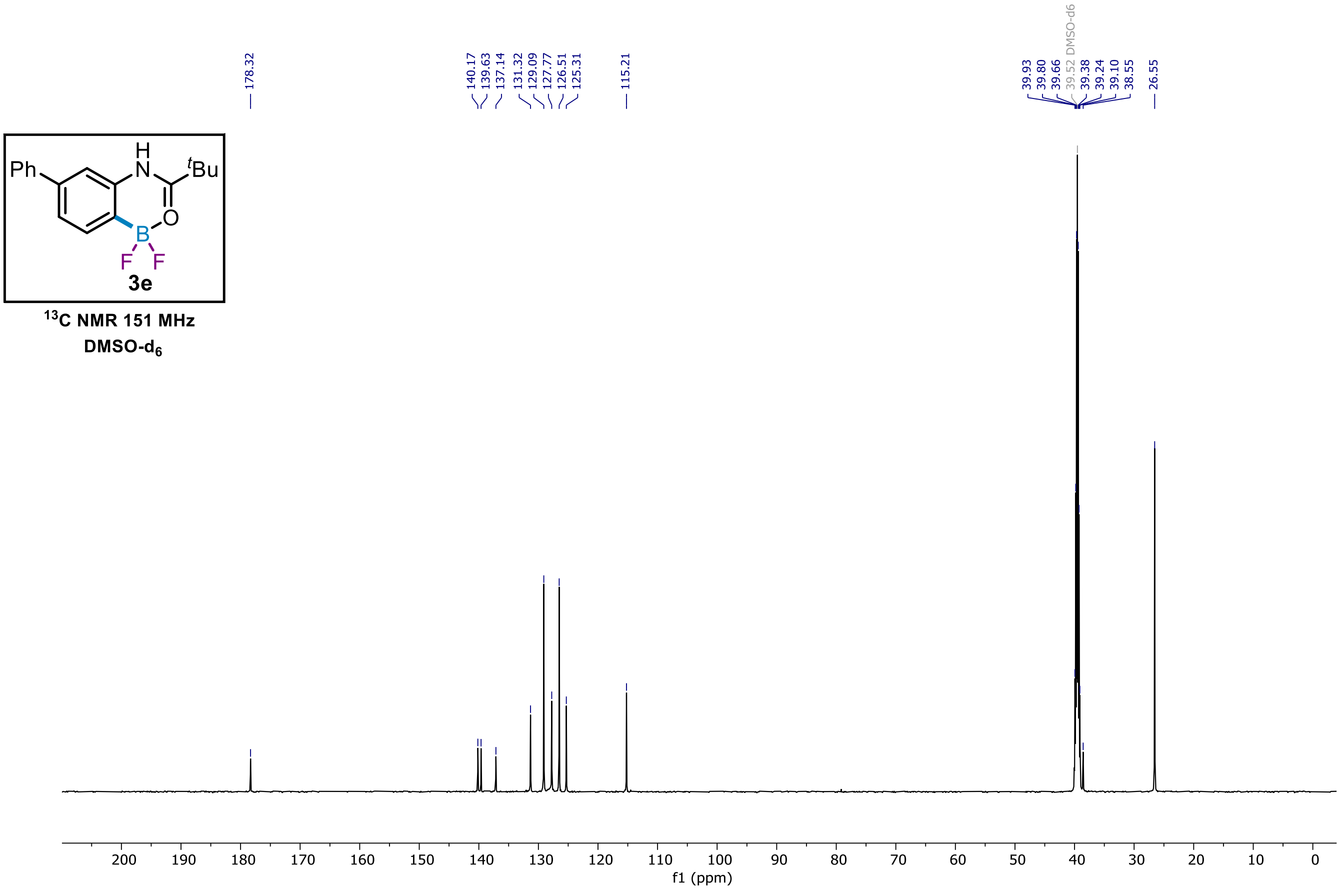

Figure S11-5: ^{13}C spectrum of compound **3e** in DMSO-d_6. Note that the ^{13}C signal for the C-BF_2 bond does not appear.

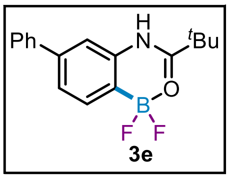

^{19}F NMR 659 MHz
DMSO-d_6

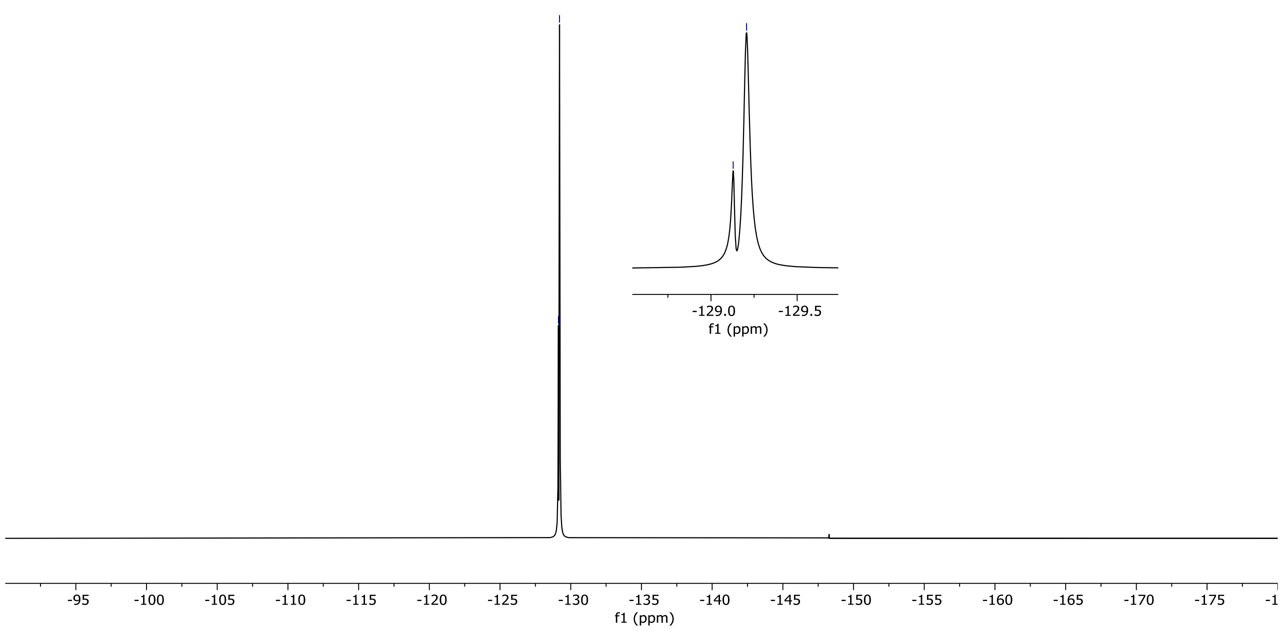

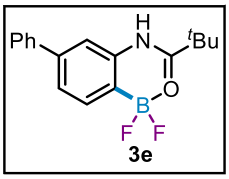

^{11}B NMR 193 MHz
DMSO-d_6

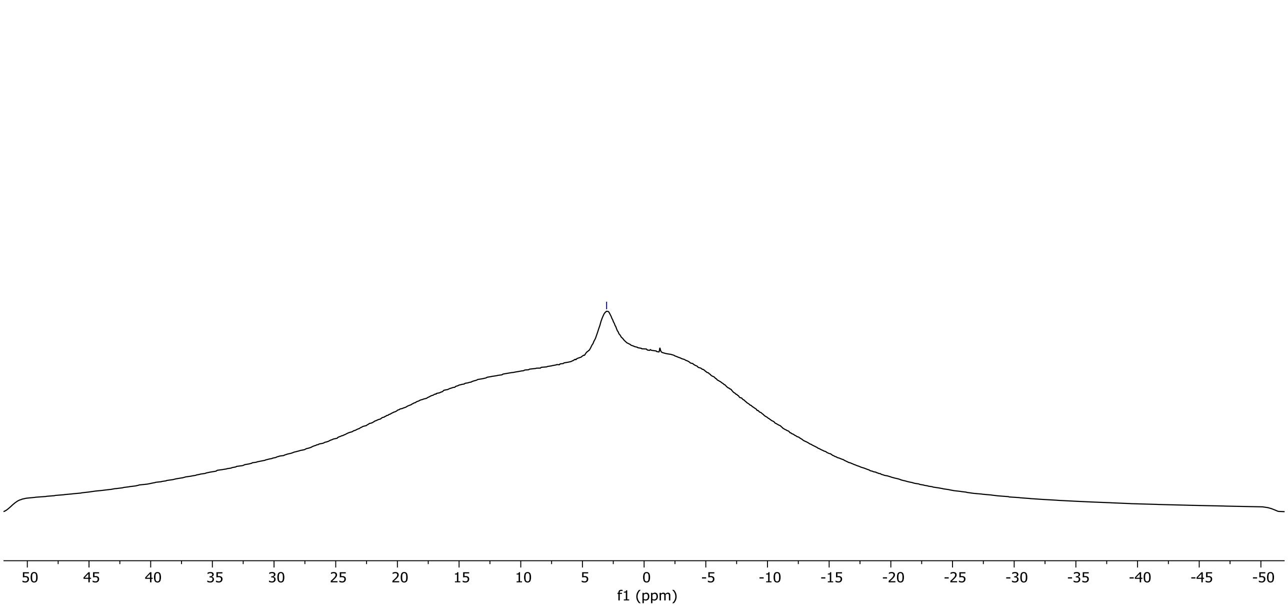

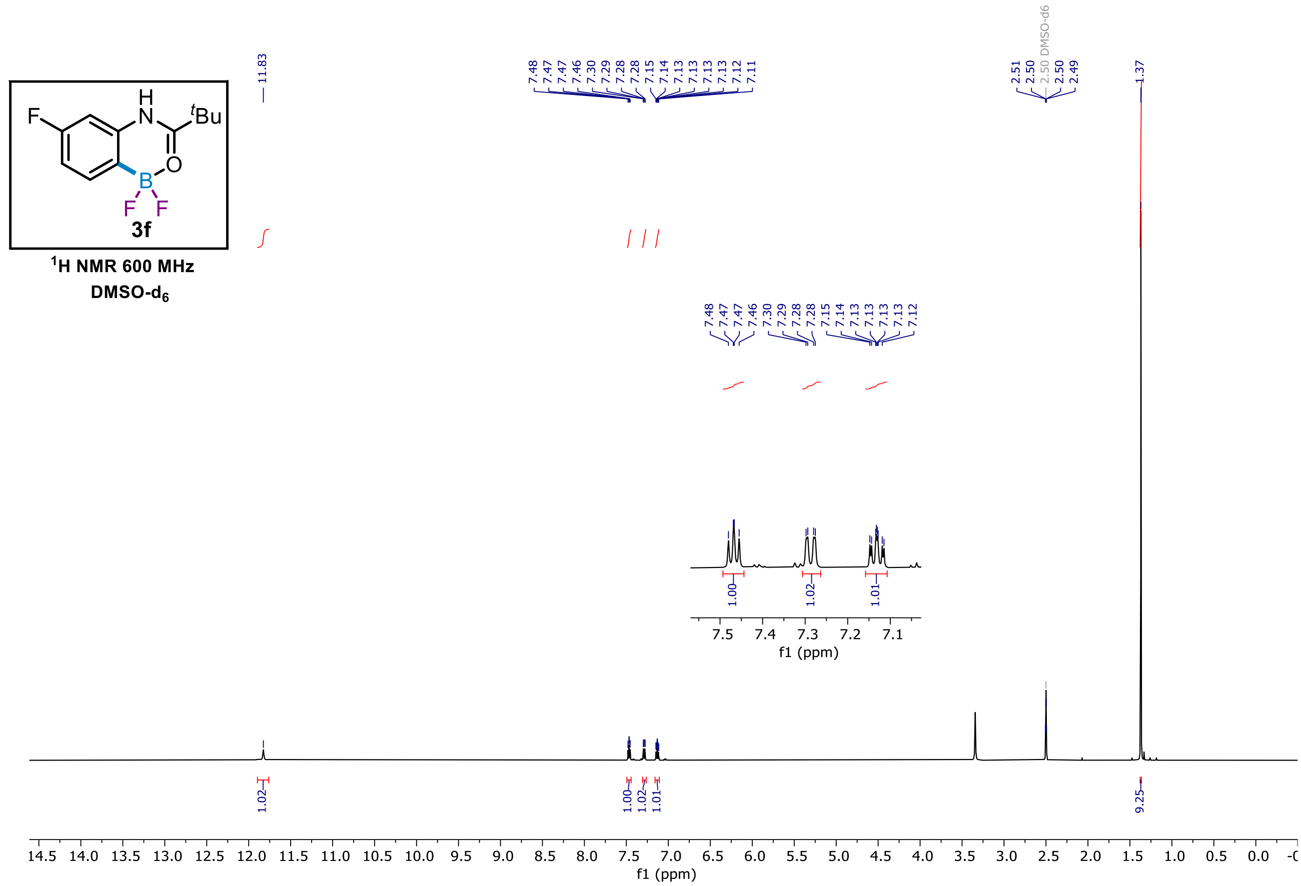

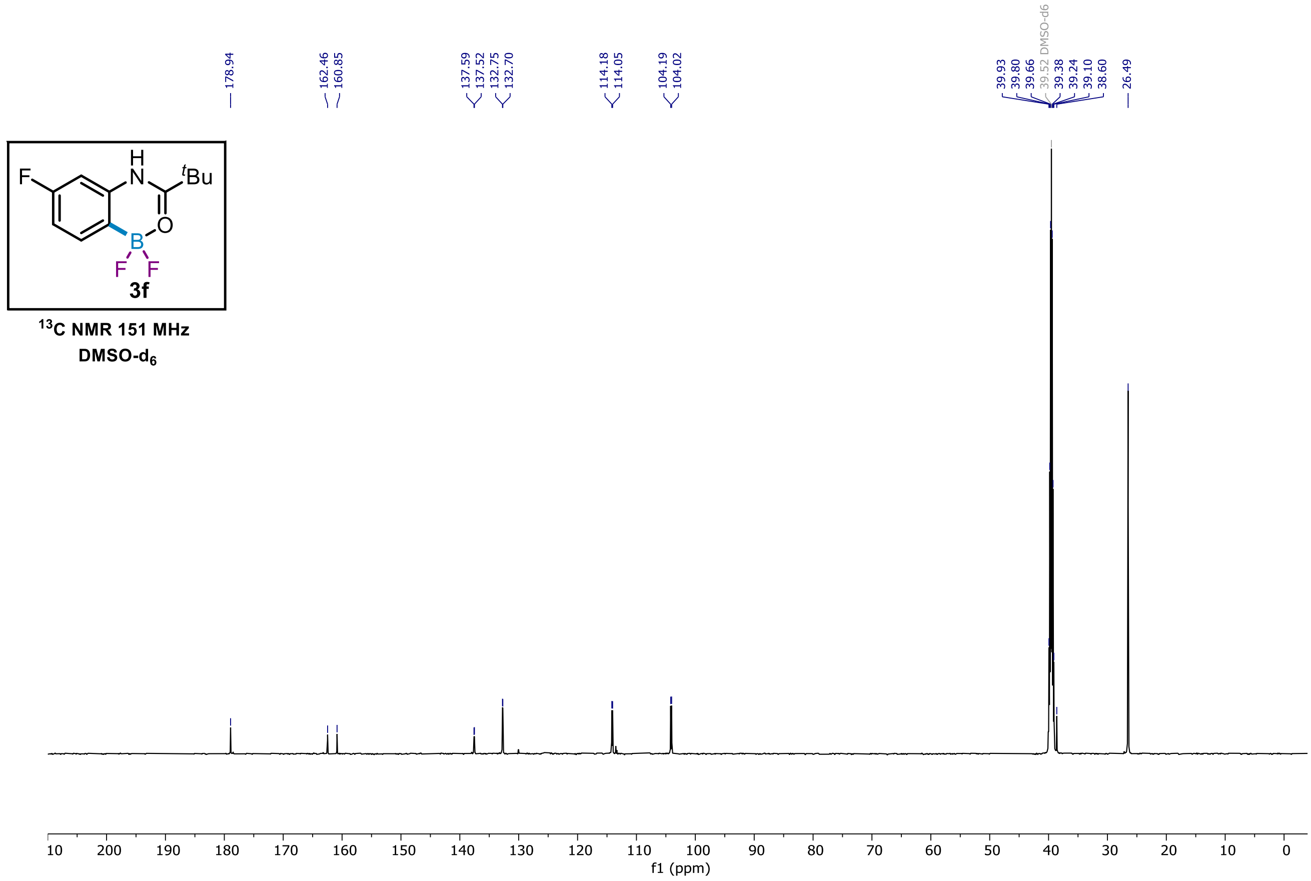

Figure S11-6: ^{13}C spectrum of compound **3f** in DMSO-d_6. Note that the ^{13}C signal for the C-BF_2 bond does not appear.

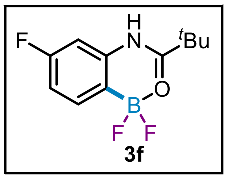

^{19}F NMR 659 MHz
DMSO-d$_6$

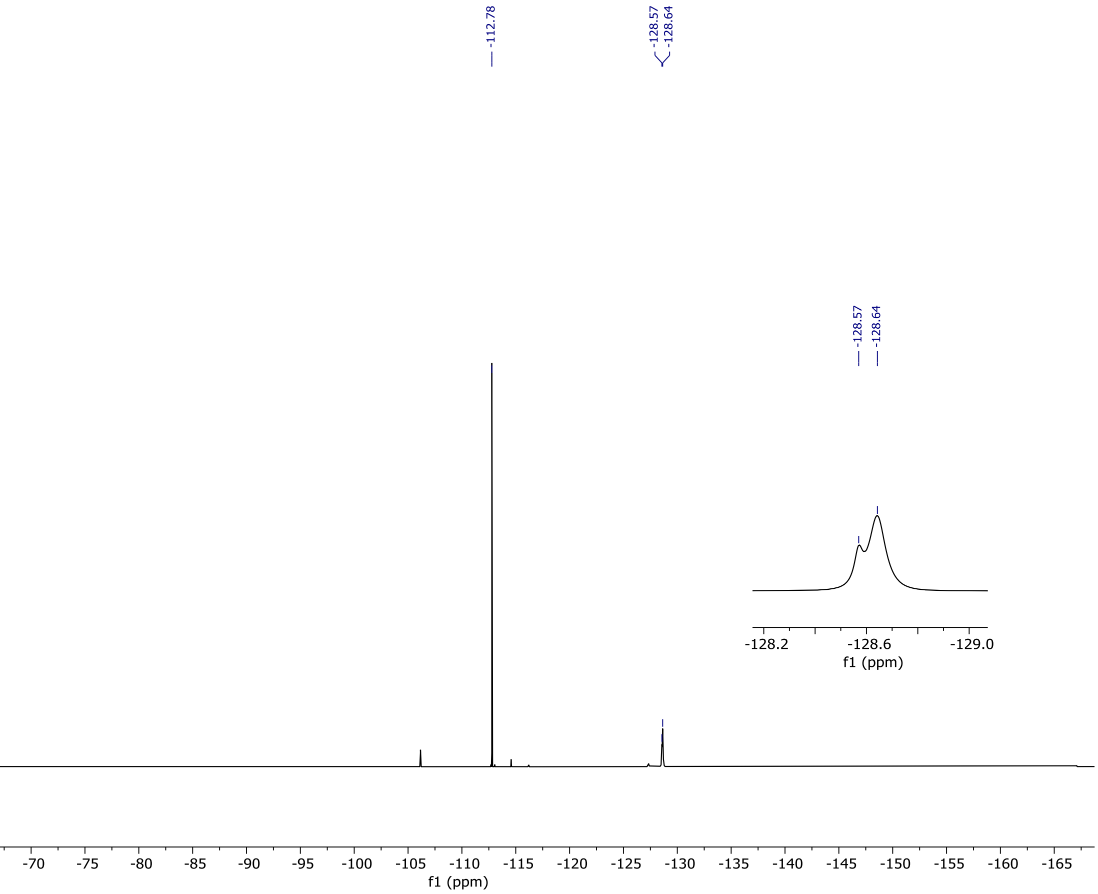

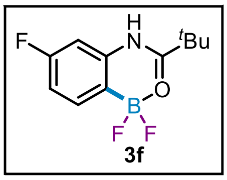

^{11}B NMR 193 MHz
DMSO-d$_6$

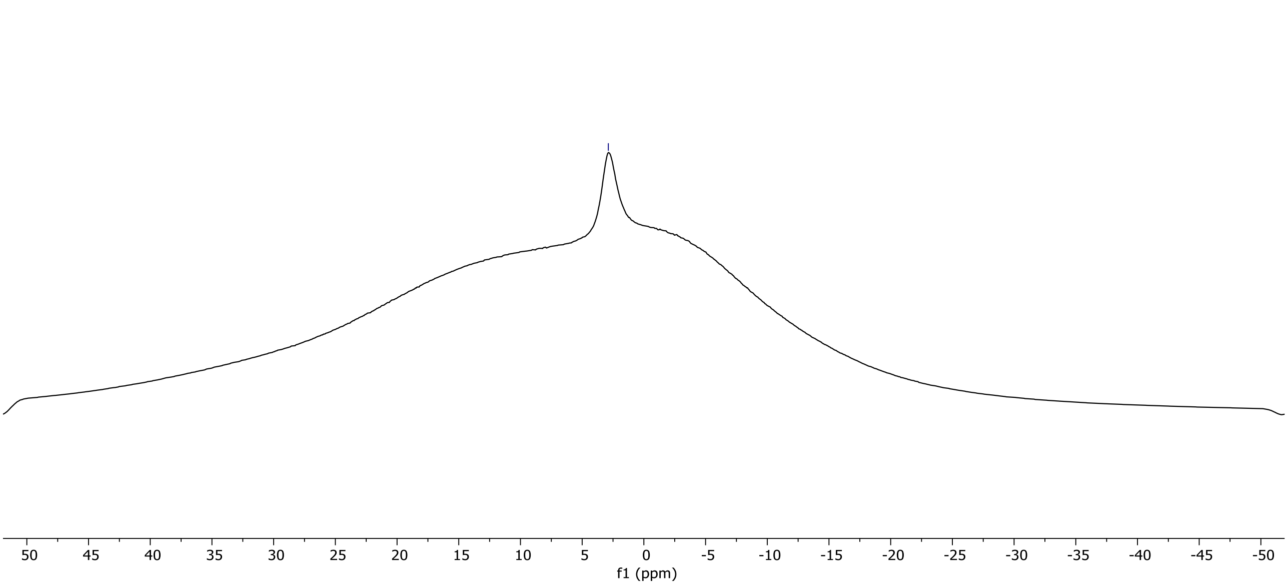

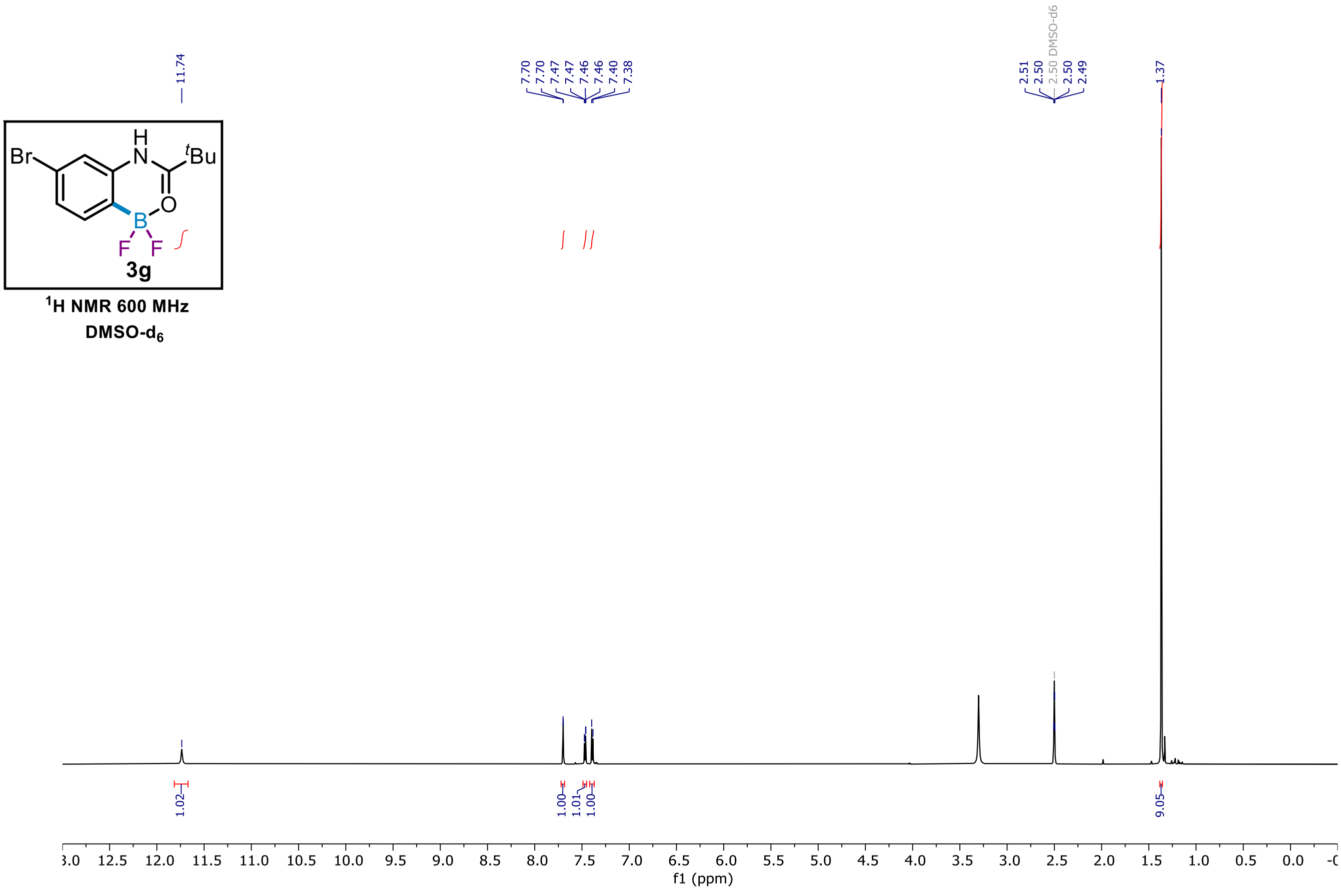

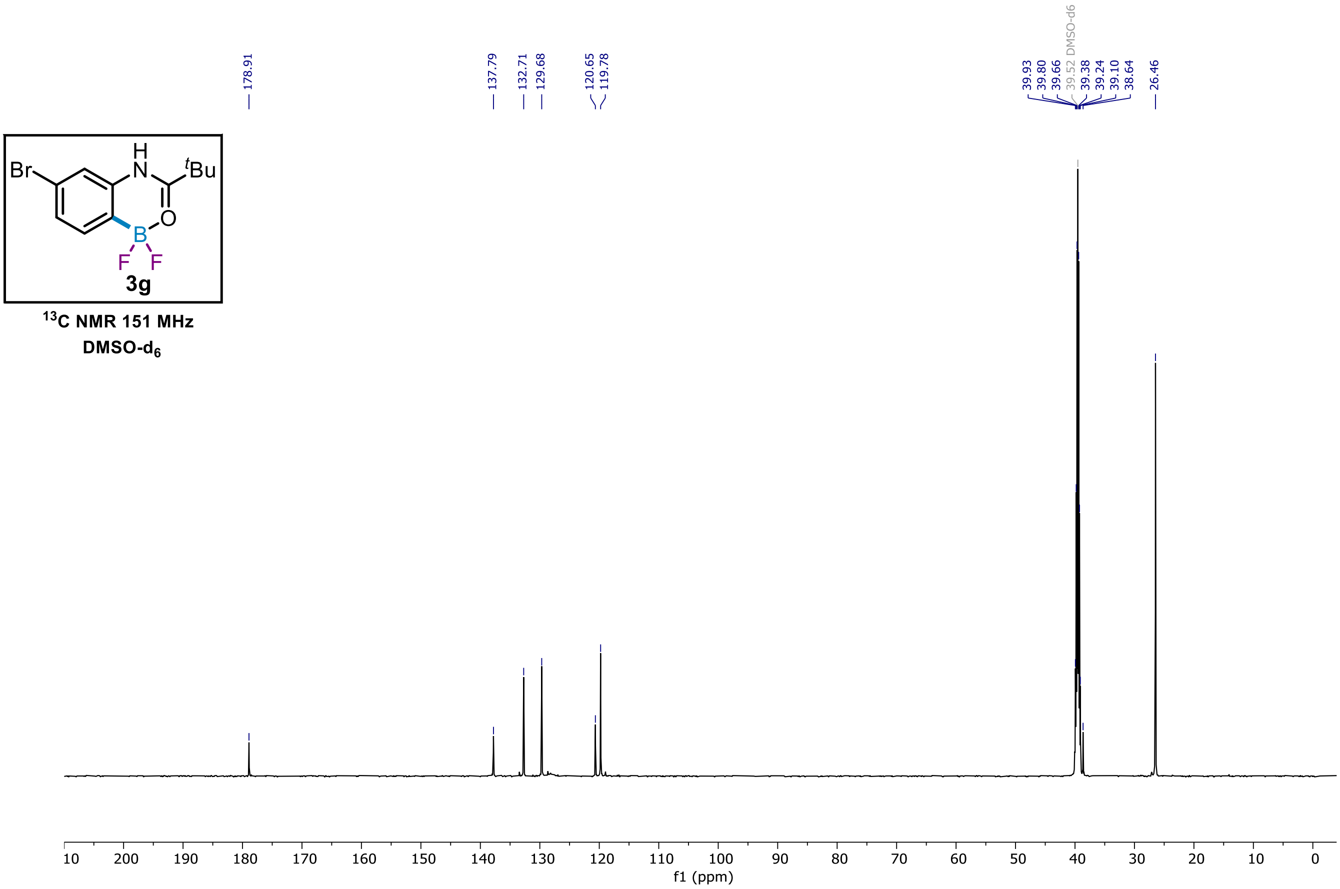

Figure S11-7: ^{13}C spectrum of compound **3g** in DMSO-d_6. Note that the ^{13}C signal for the C-BF_2 bond does not appear.

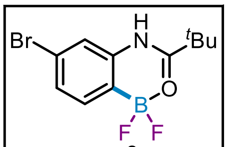

3g

^{19}F NMR 659 MHz
DMSO-d_6

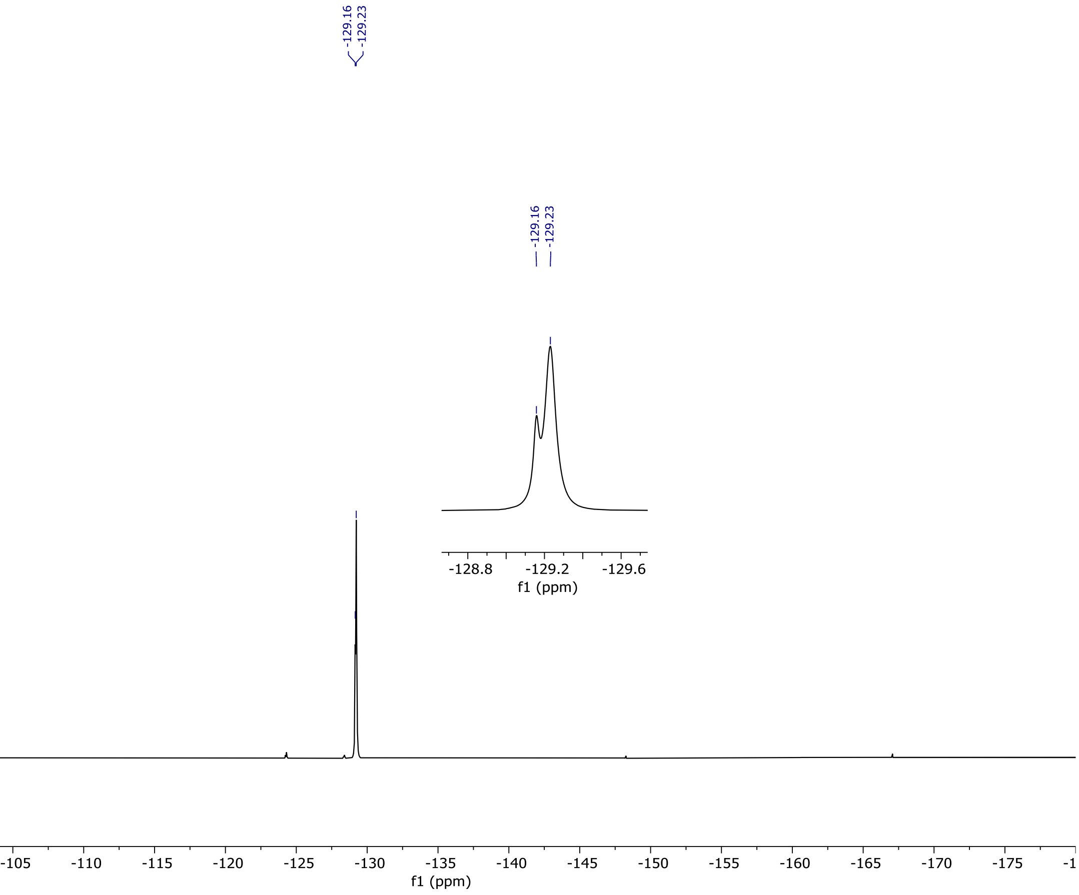

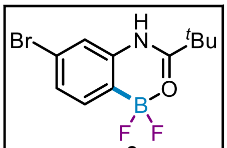

3g

^{11}B NMR 193 MHz
DMSO-d_6

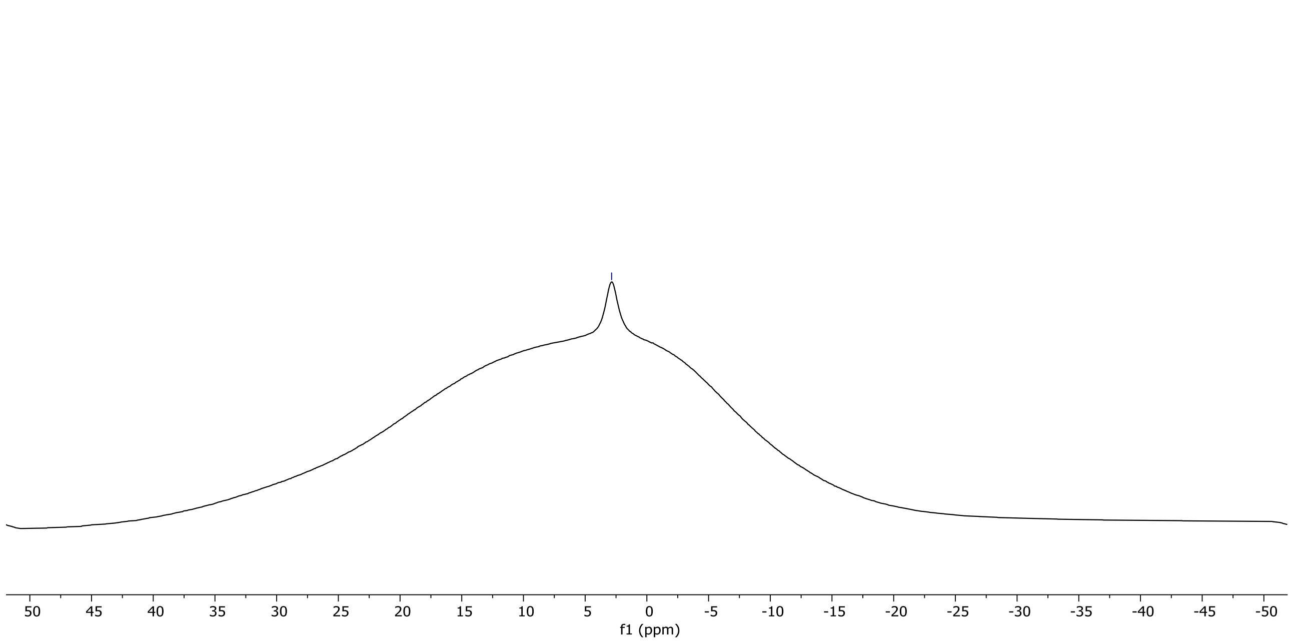

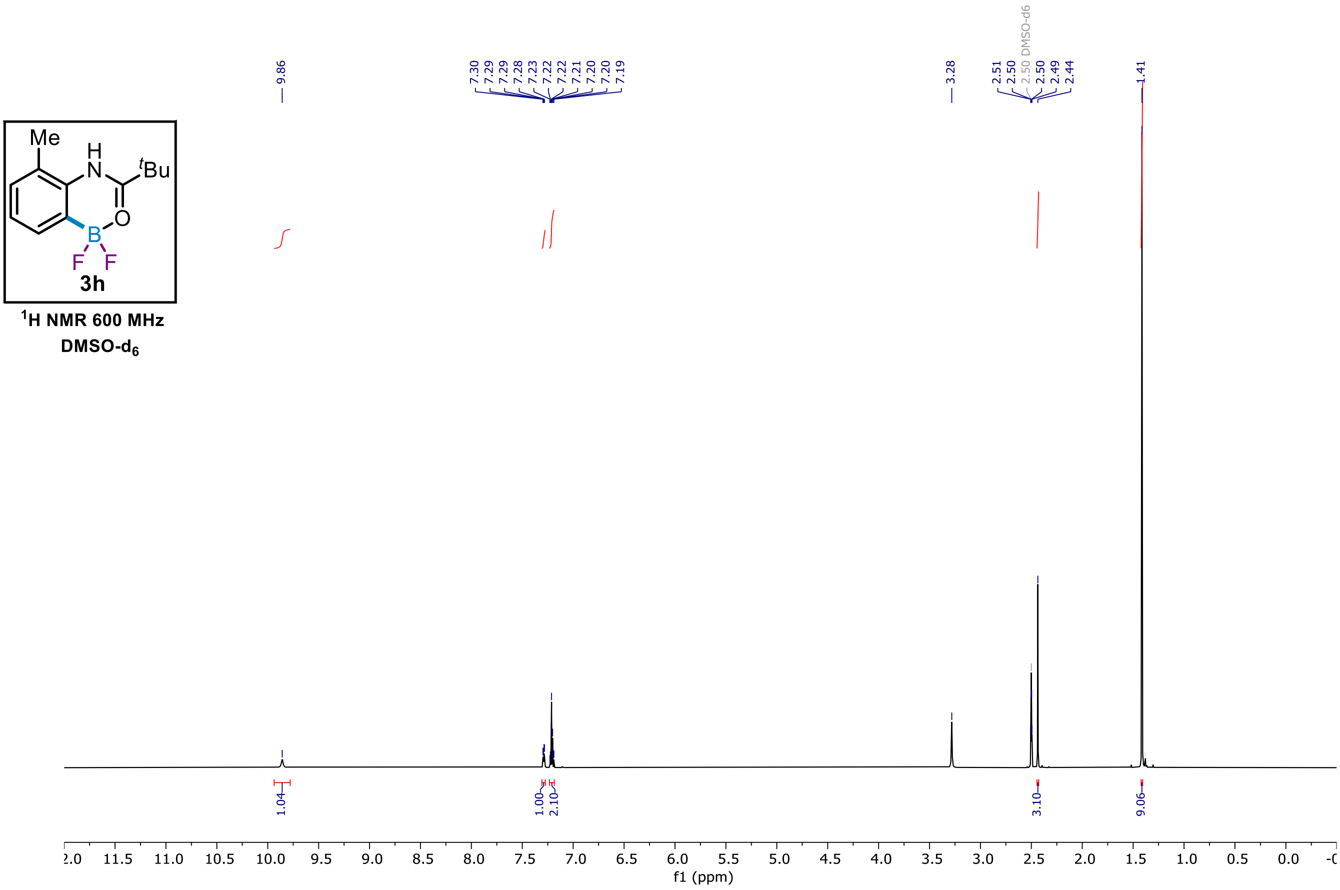

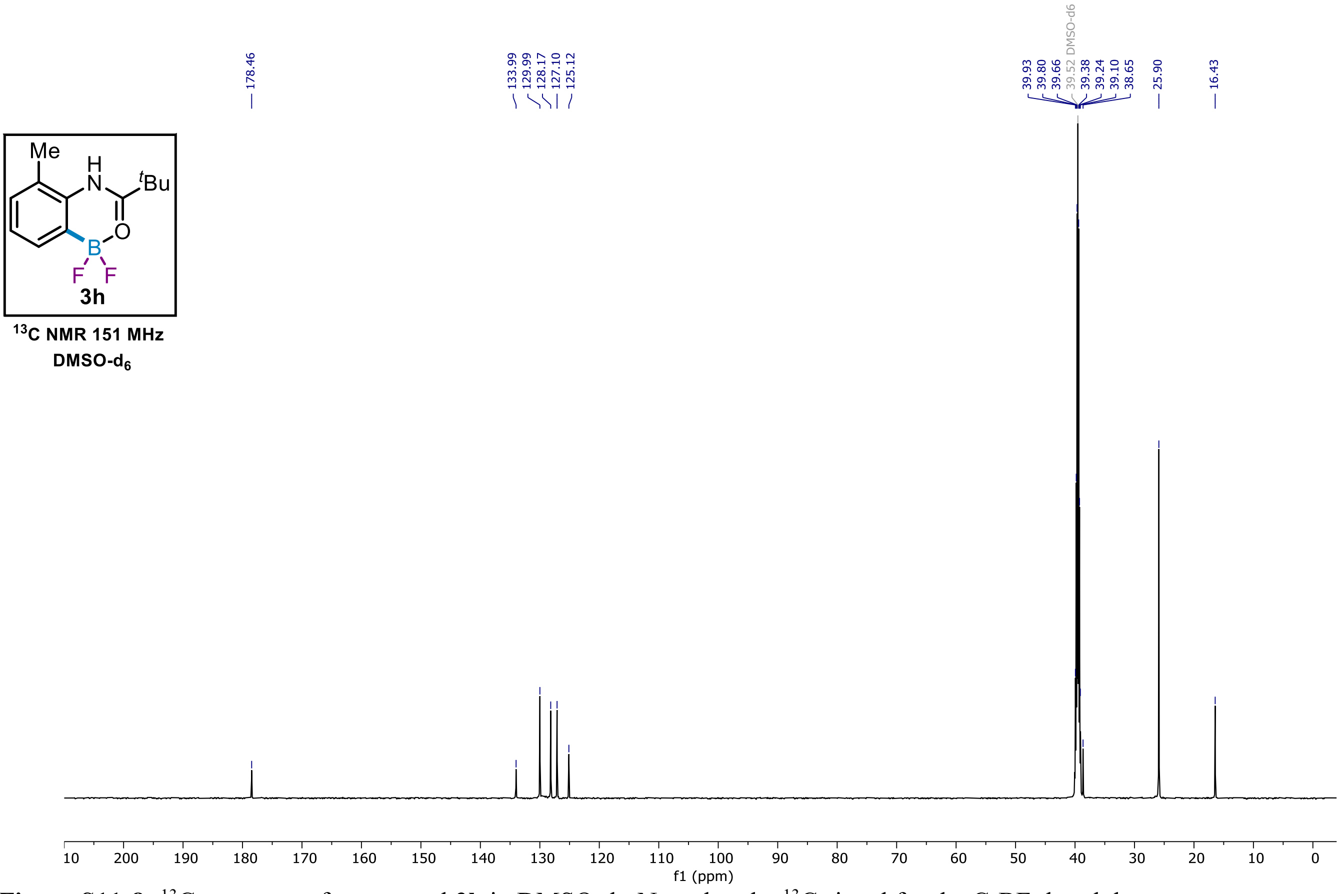

Figure S11-8: ^{13}C spectrum of compound **3h** in DMSO-d$_6$. Note that the ^{13}C signal for the C-BF_2 bond does not appear.

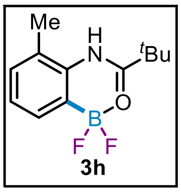

3h

^{19}F NMR 659 MHz
DMSO-d_6

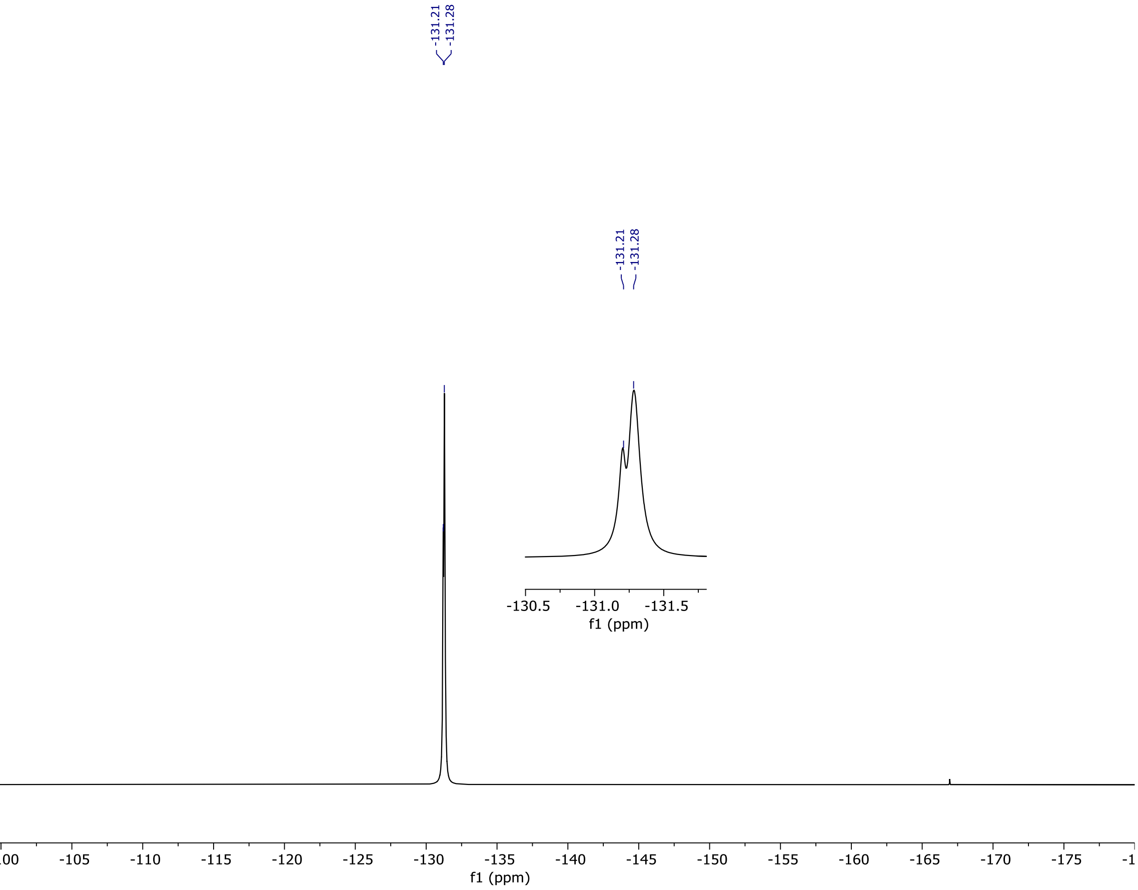

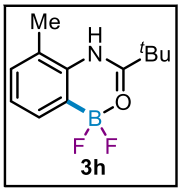

3h

^{11}B NMR 193 MHz
DMSO-d_6

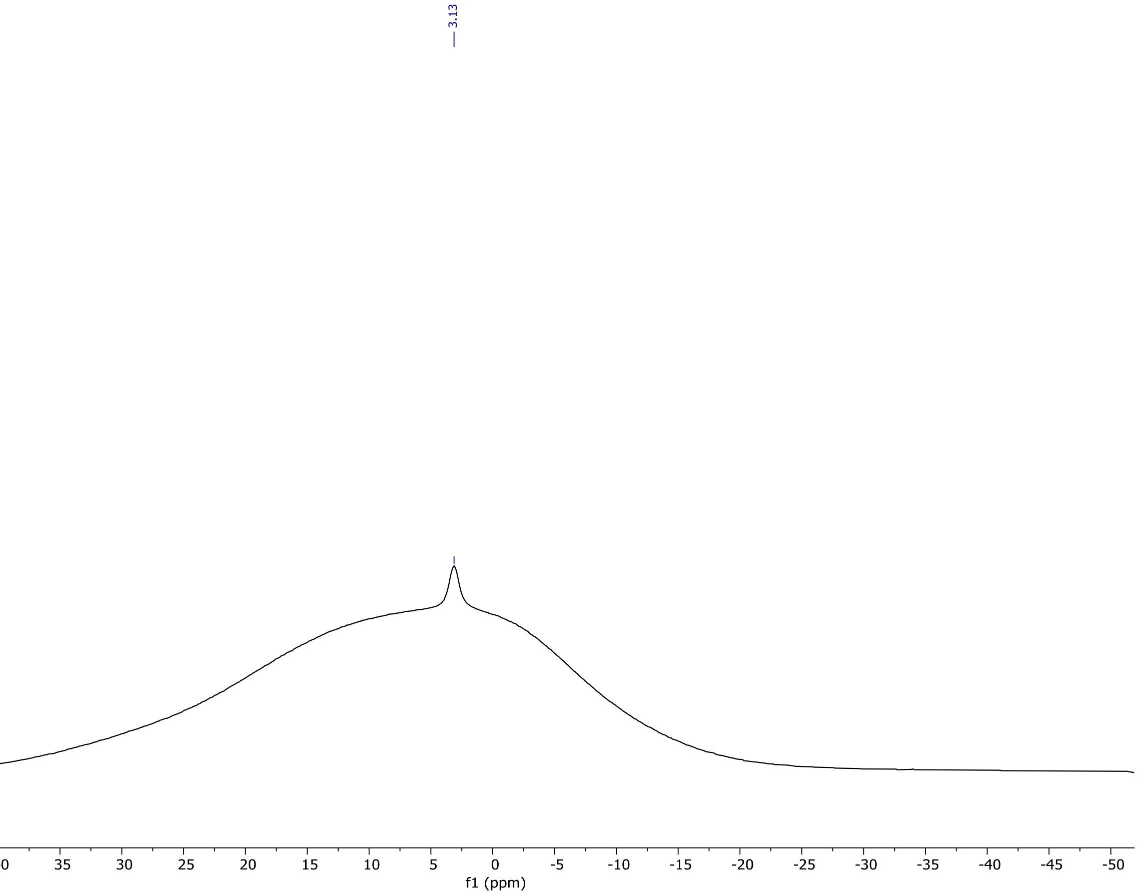

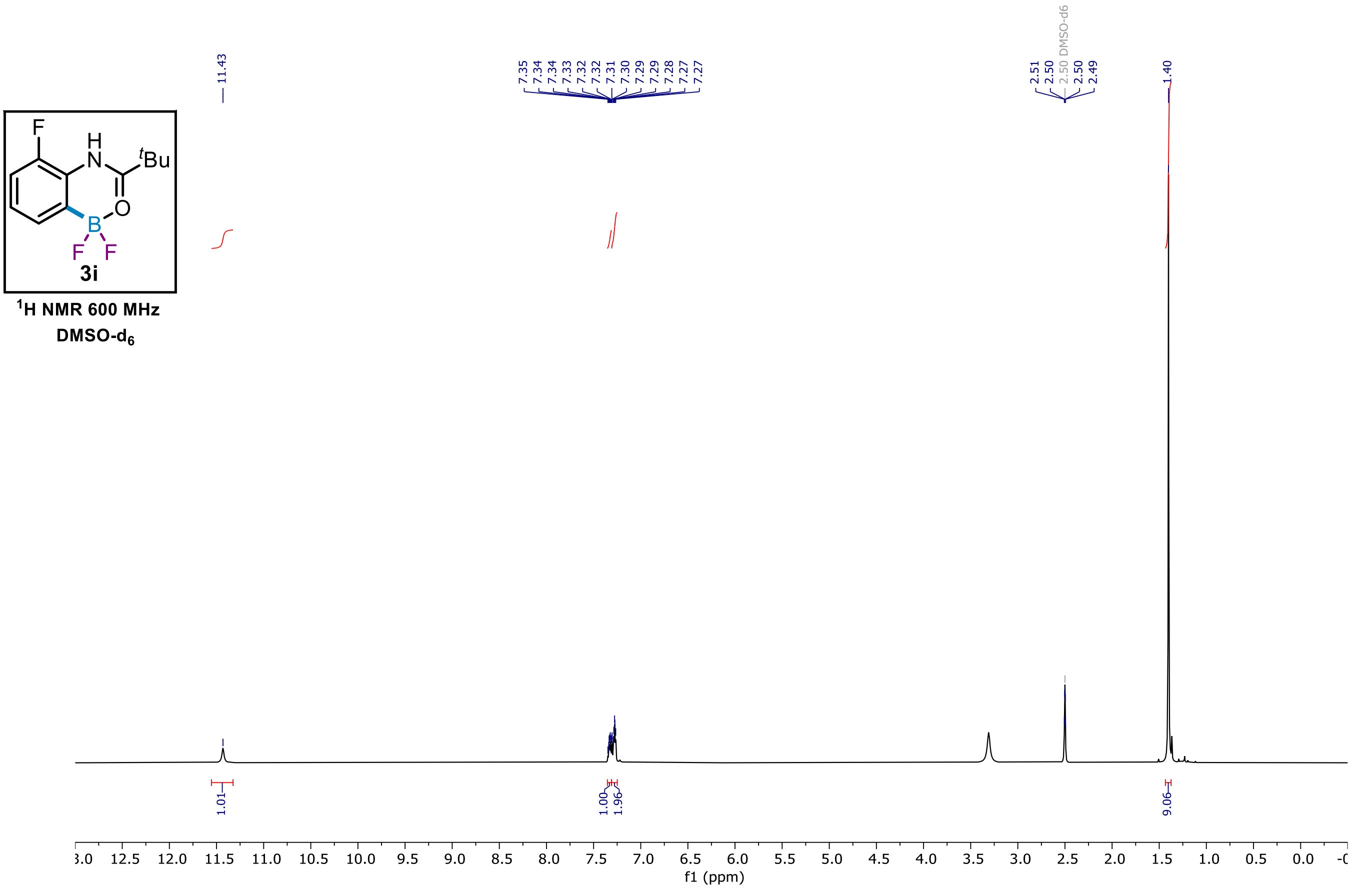

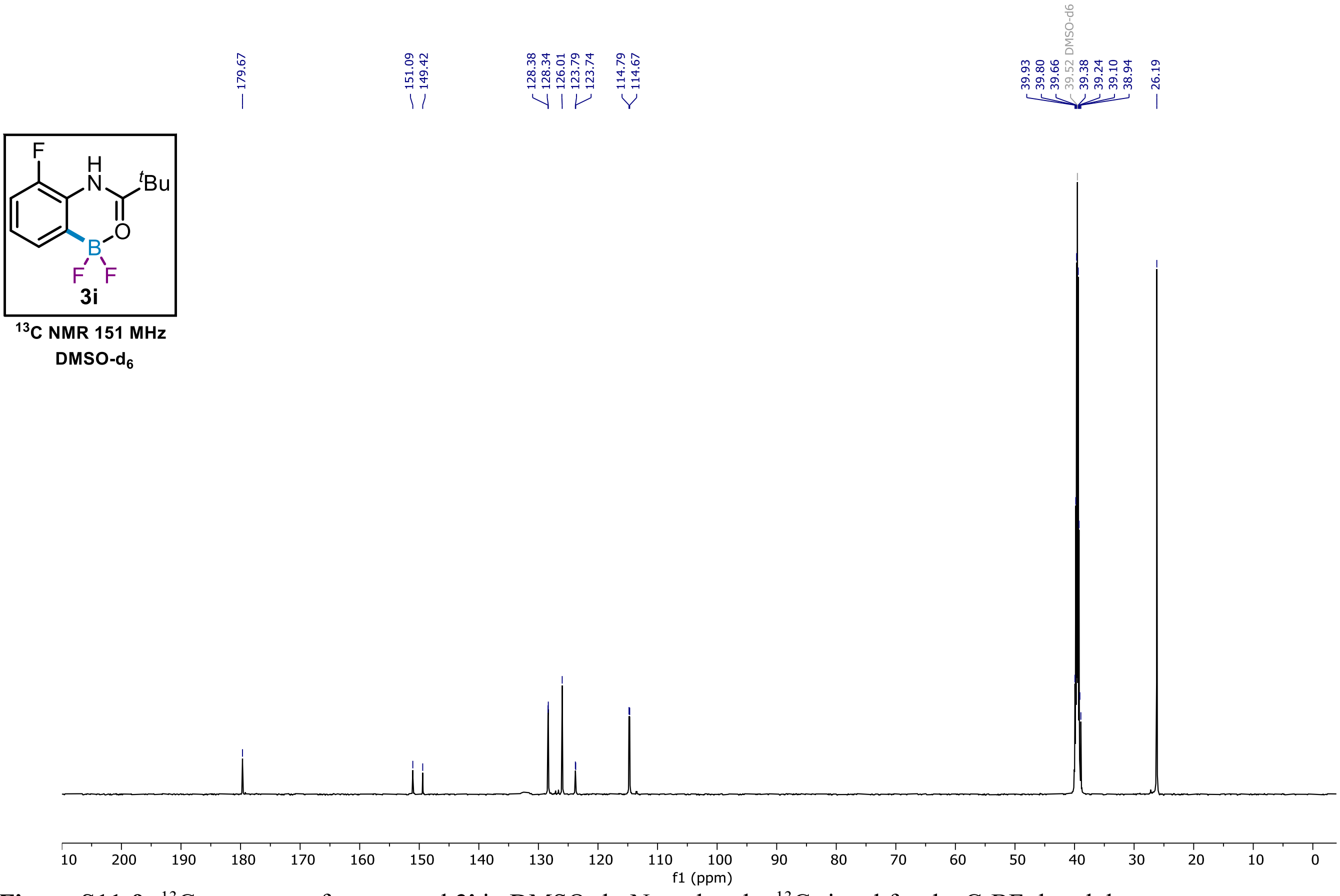

Figure S11-9: ^{13}C spectrum of compound **3i** in DMSO-d$_6$. Note that the ^{13}C signal for the C-BF$_2$ bond does not appear.

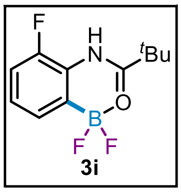

^{19}F NMR 659 MHz
DMSO-d_6

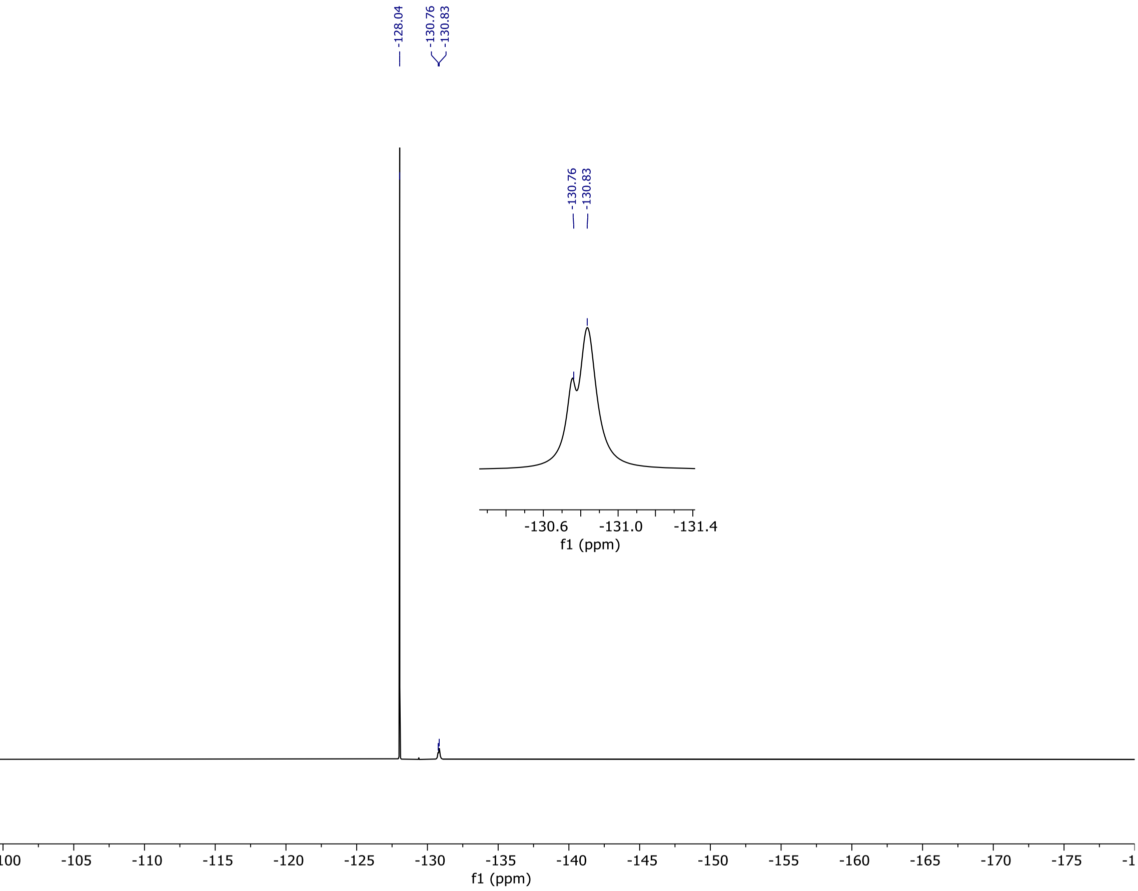

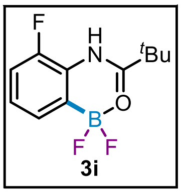

^{11}B NMR 193 MHz
DMSO-d_6

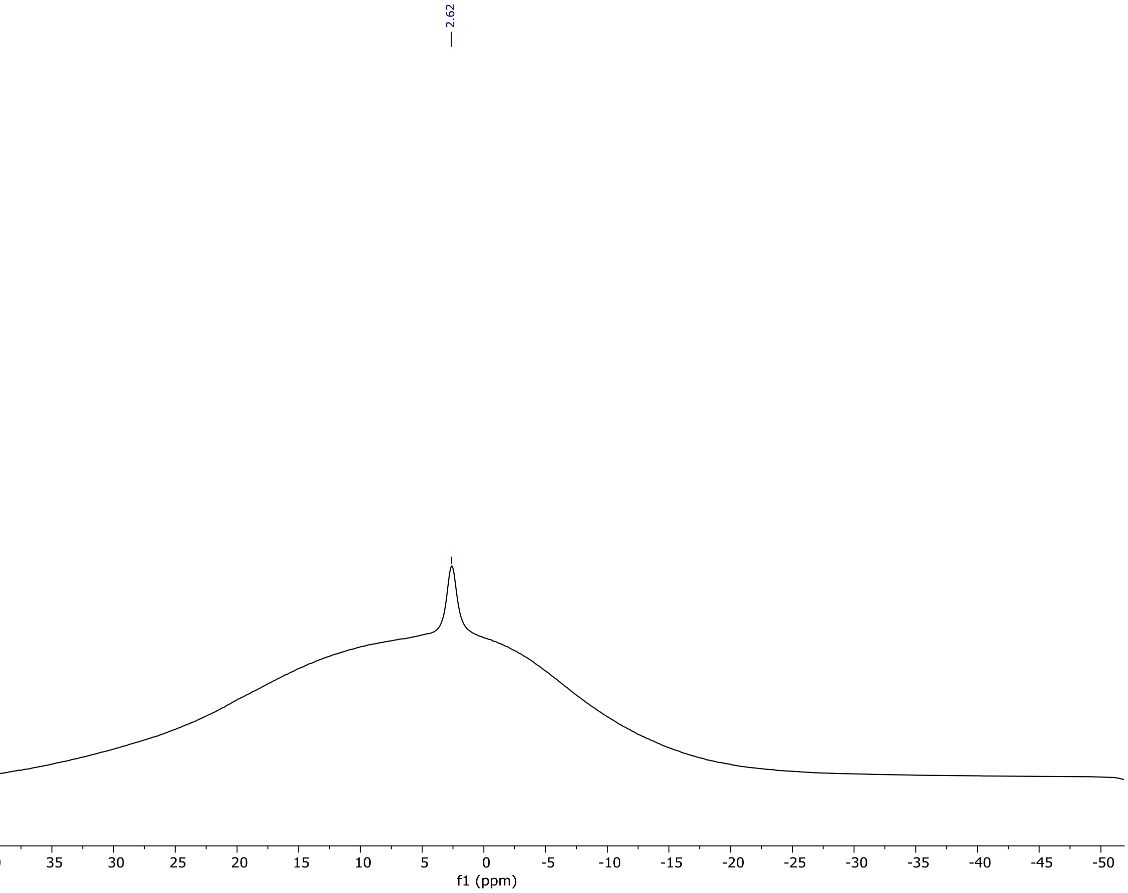

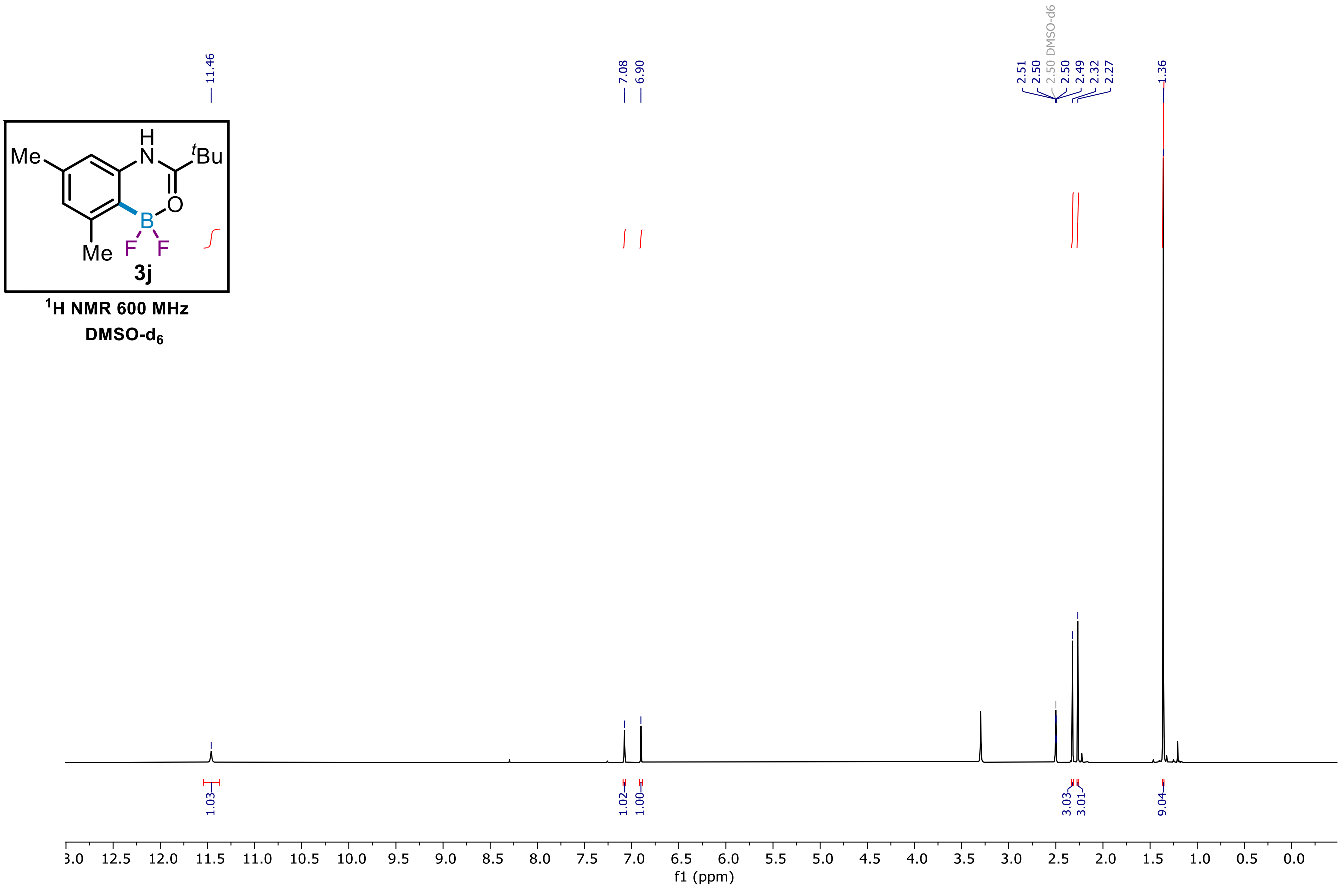

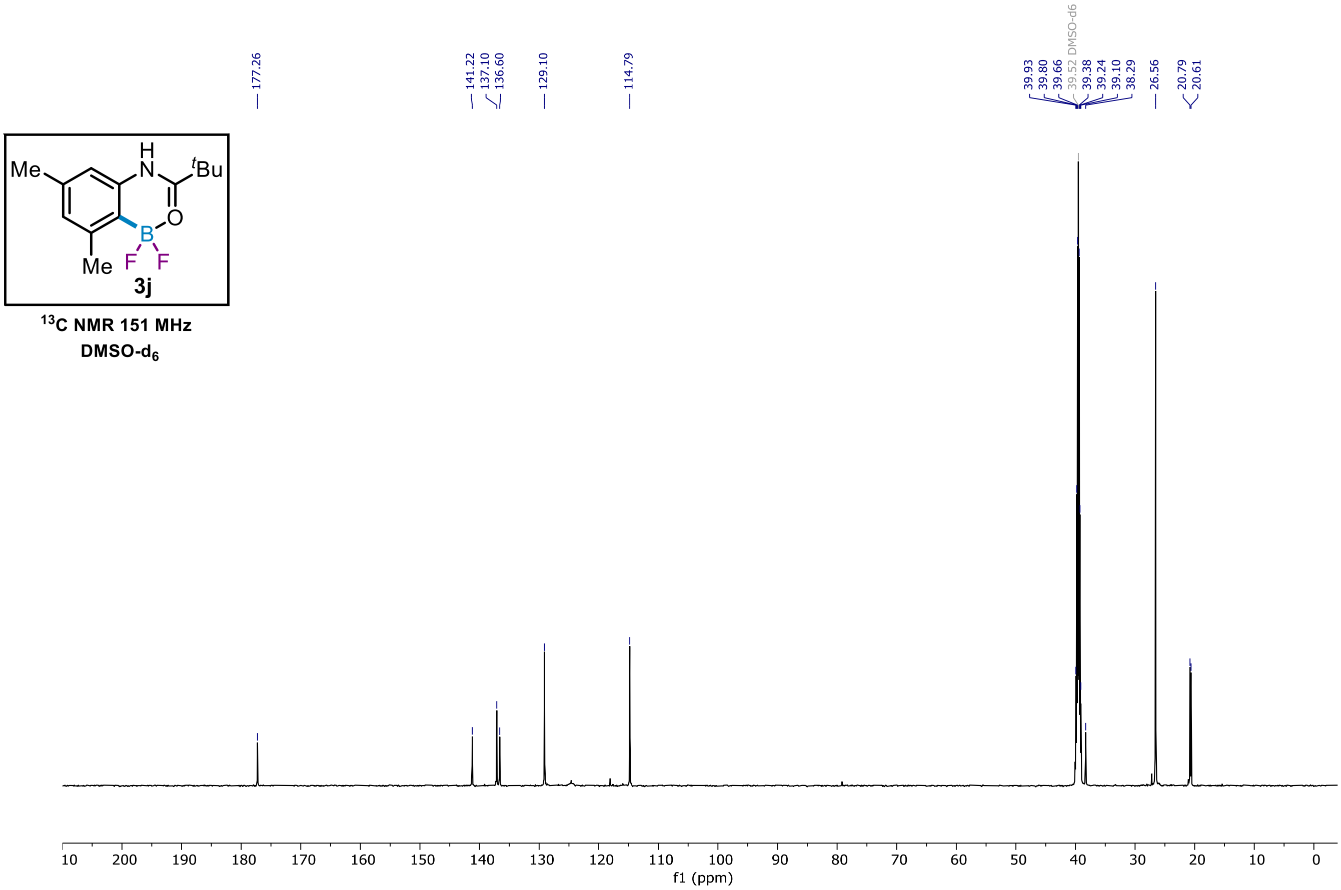

Figure S11-10: ^{13}C spectrum of compound **3j** in DMSO-d$_6$. Note that the ^{13}C signal for the C-BF$_2$ bond does not appear.

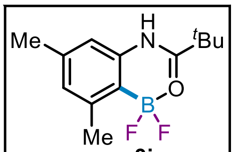

3j

^{19}F NMR 659 MHz
DMSO-d_6

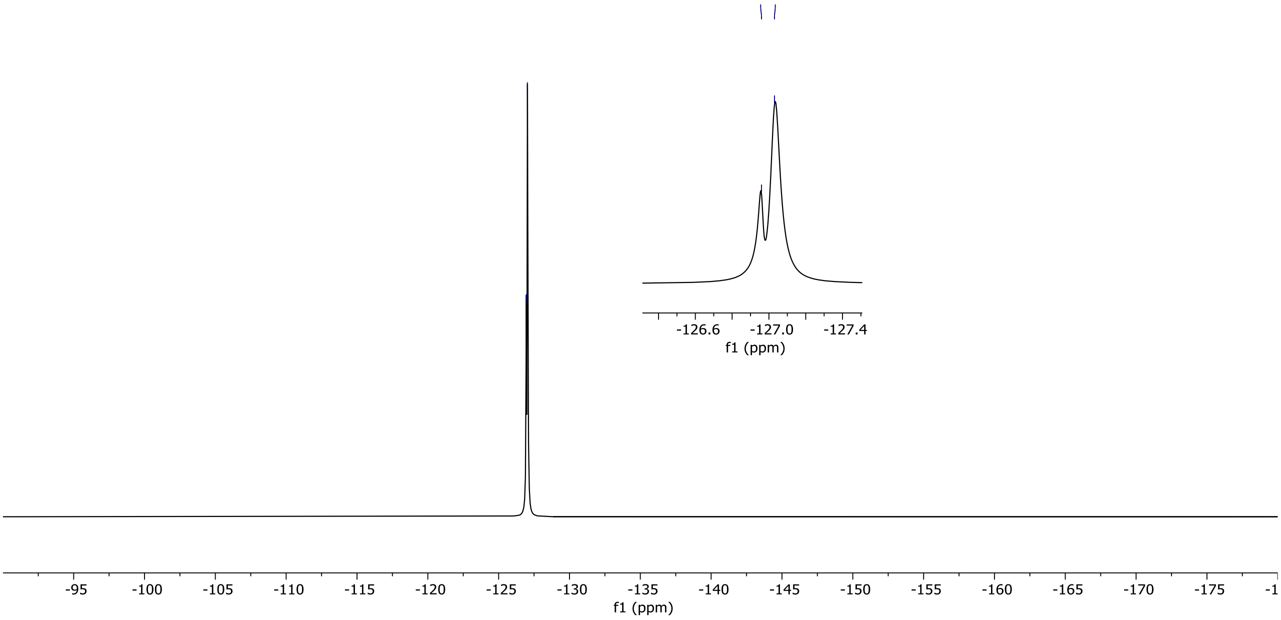

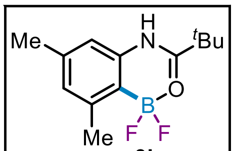

3j

^{11}B NMR 193 MHz
DMSO-d_6

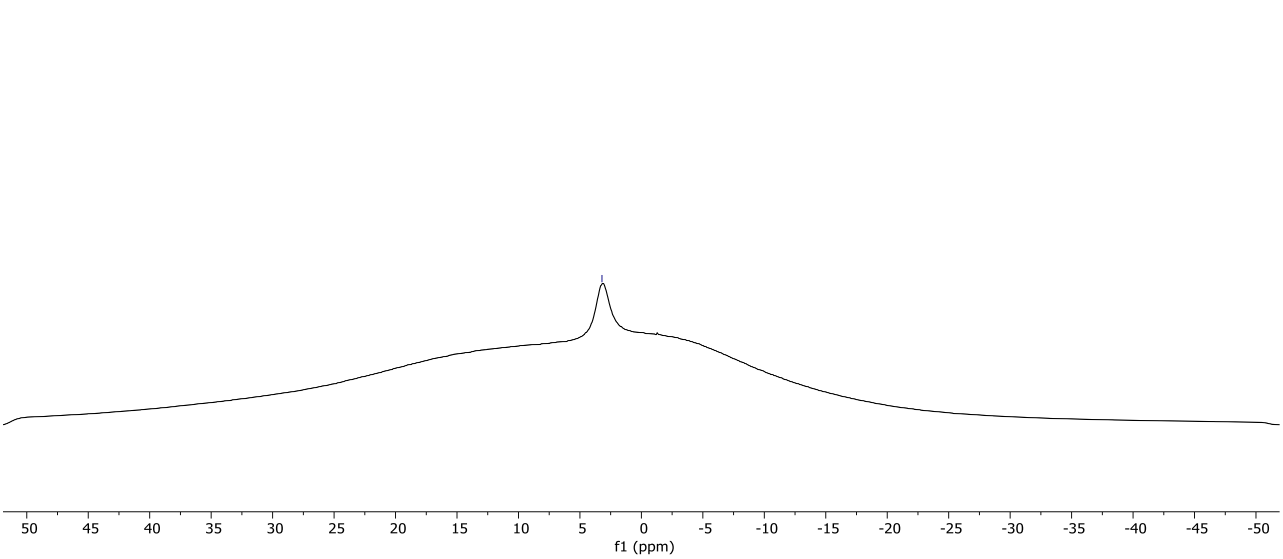

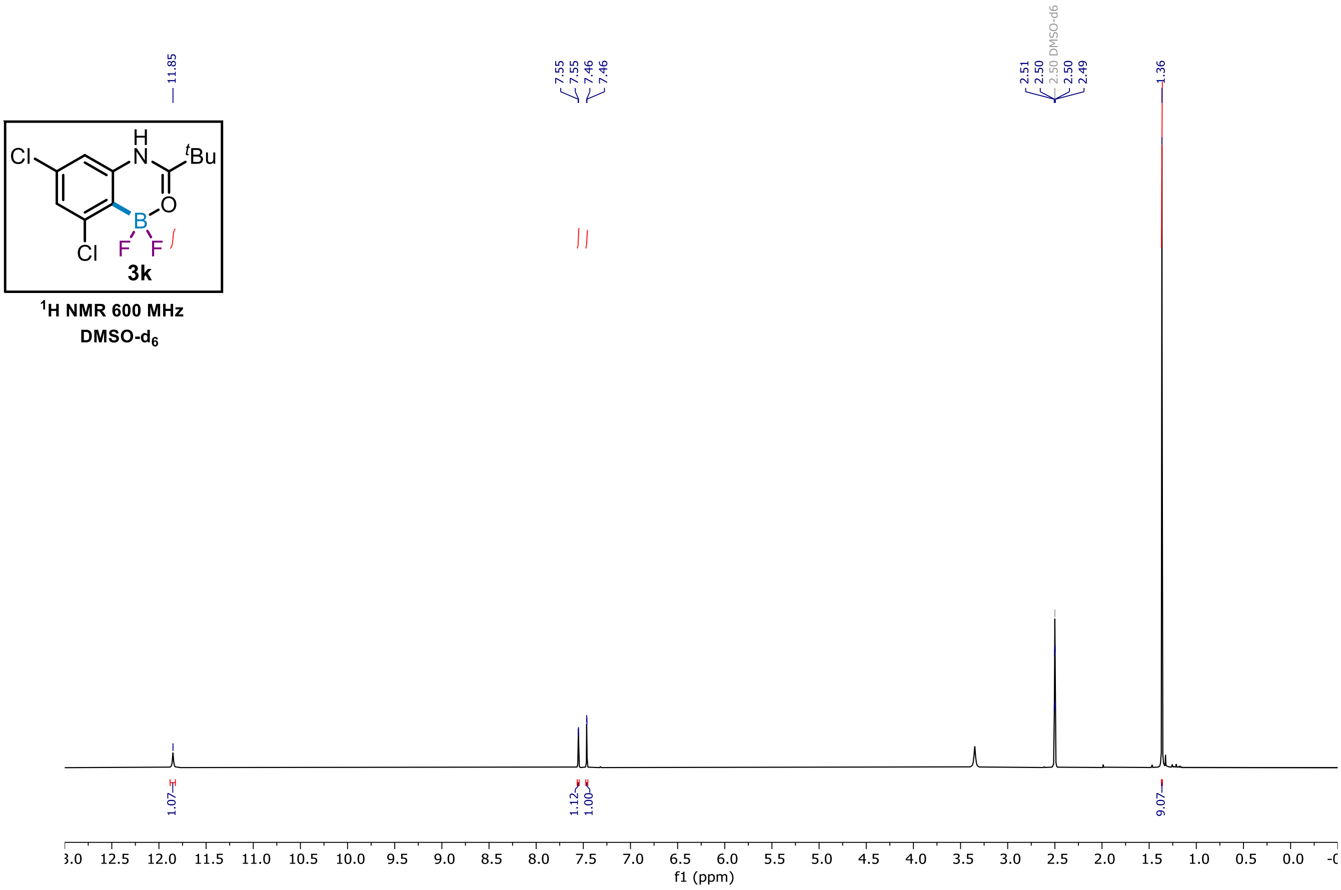

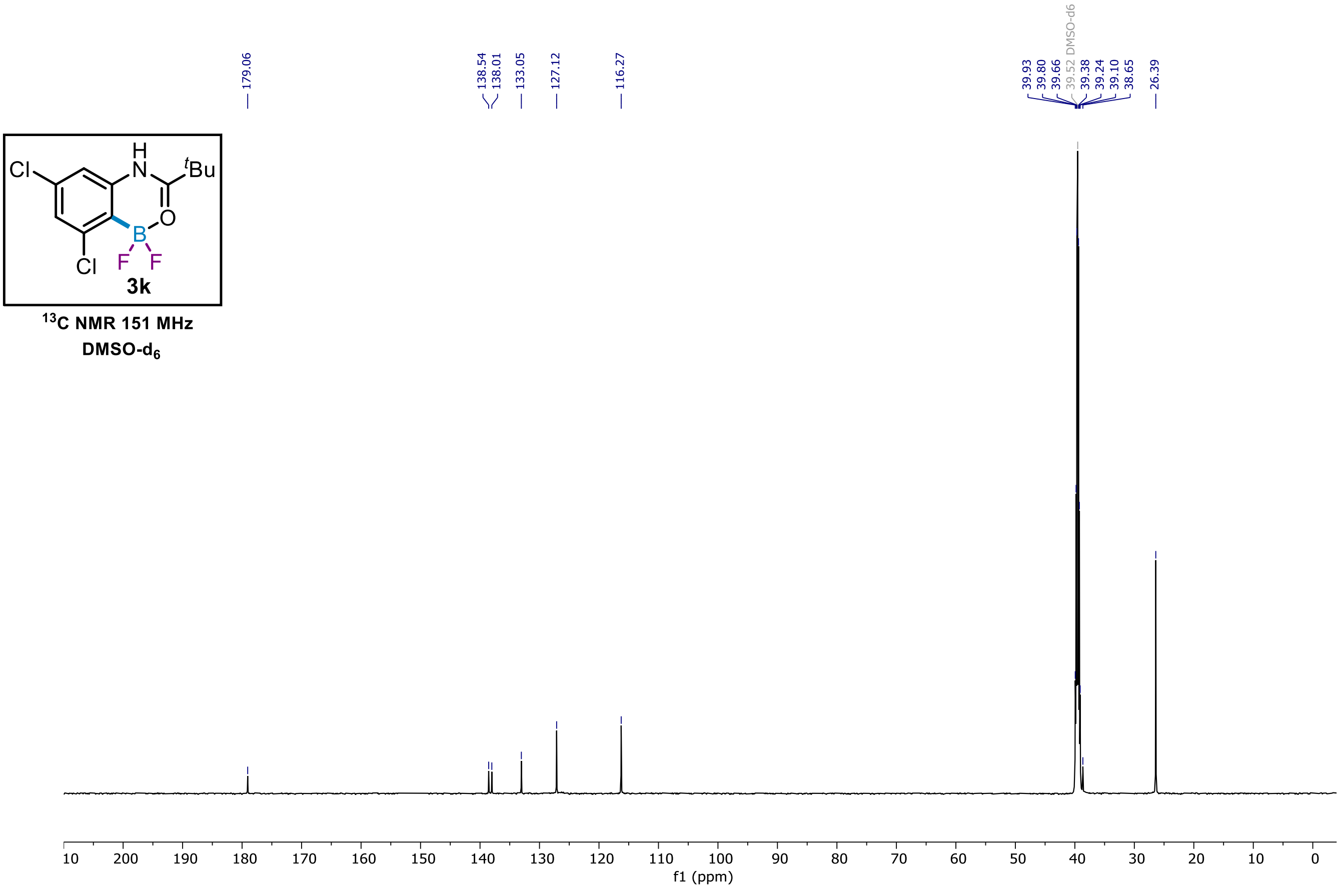

Figure S11-11: ^{13}C spectrum of compound **3k** in DMSO-d$_6$. Note that the ^{13}C signal for the C-BF$_2$ bond does not appear.

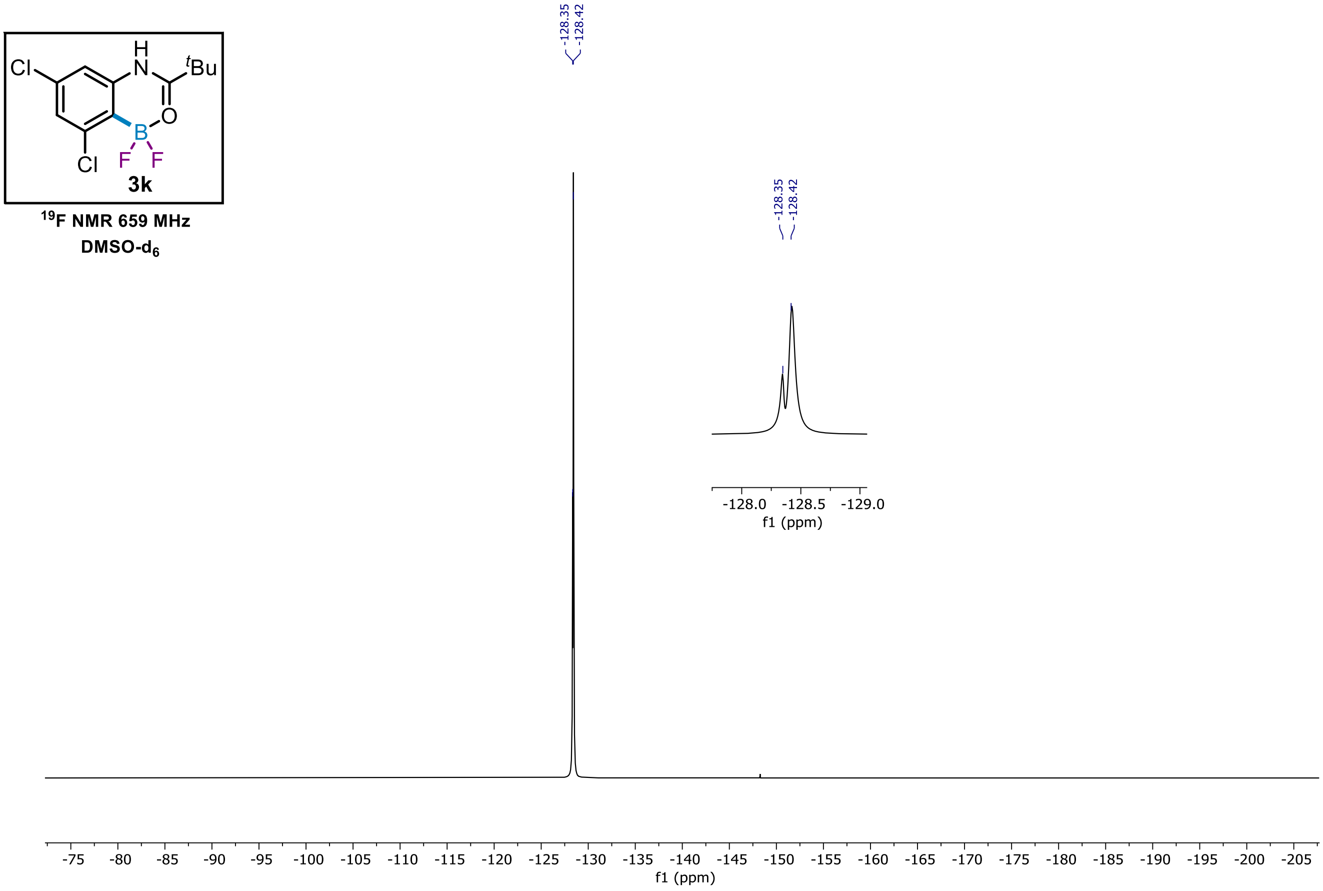
3k
^{19}F NMR 659 MHz
DMSO-d_6
-128.35
-128.42
f1 (ppm)

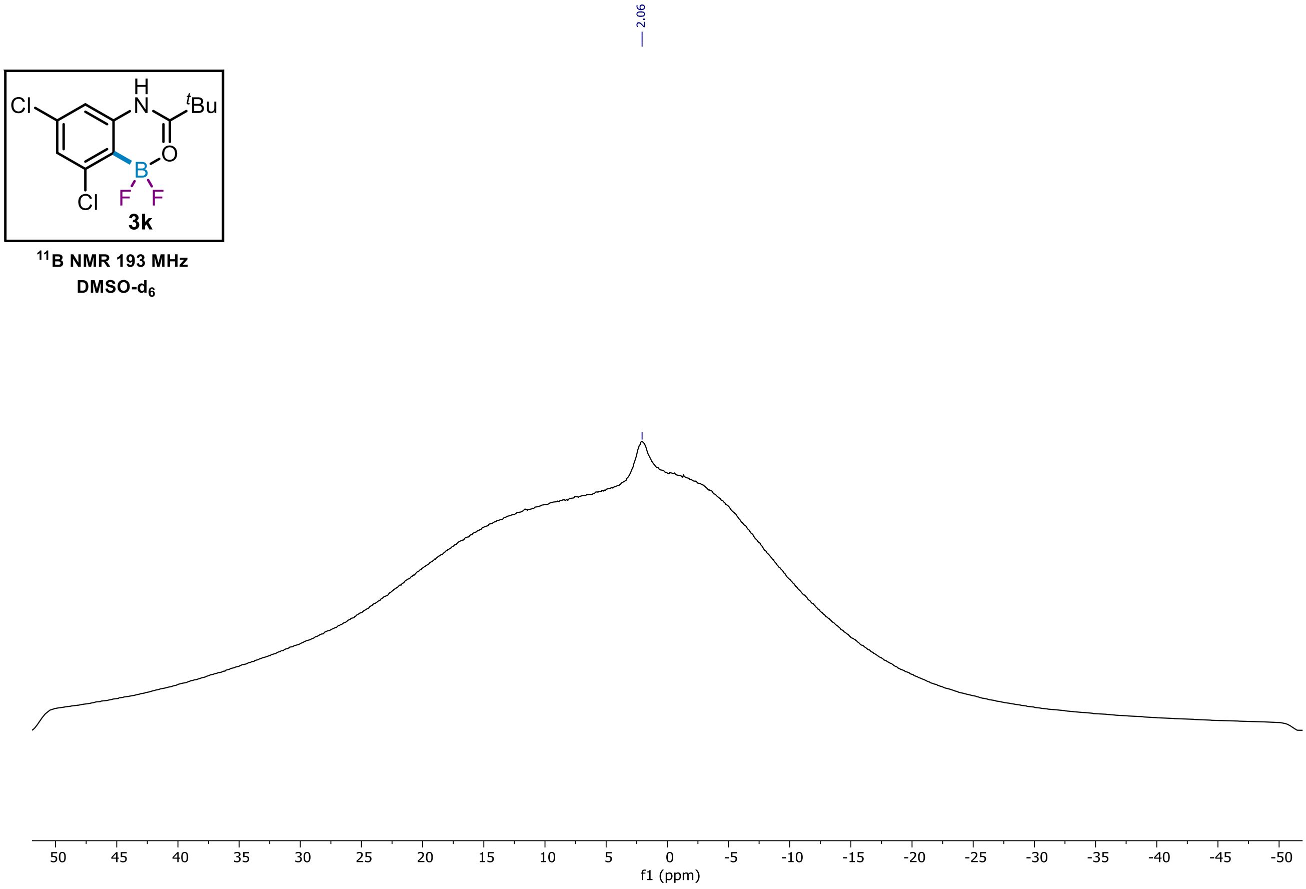
3k
^{11}B NMR 193 MHz
DMSO-d_6
2.06
f1 (ppm)

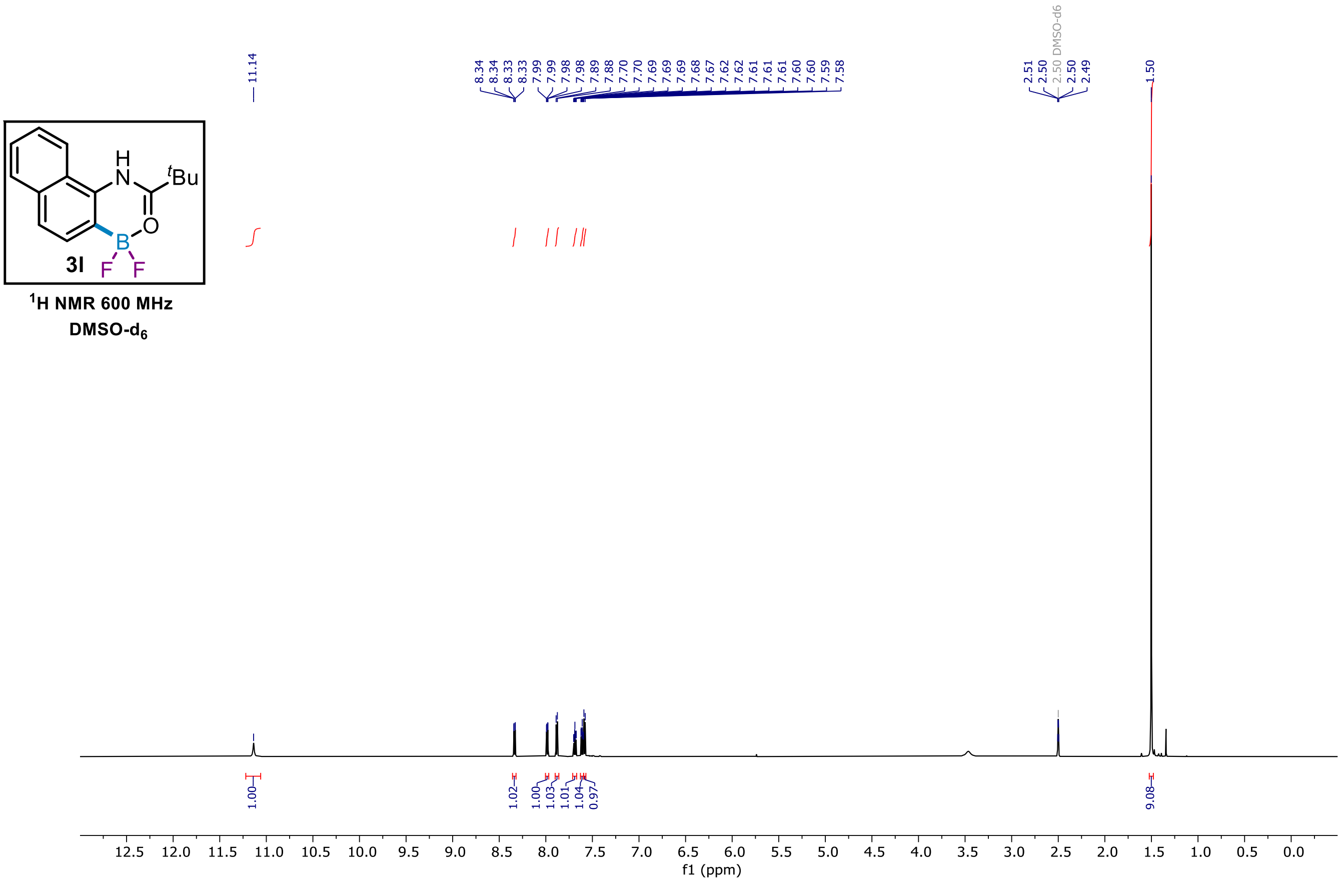

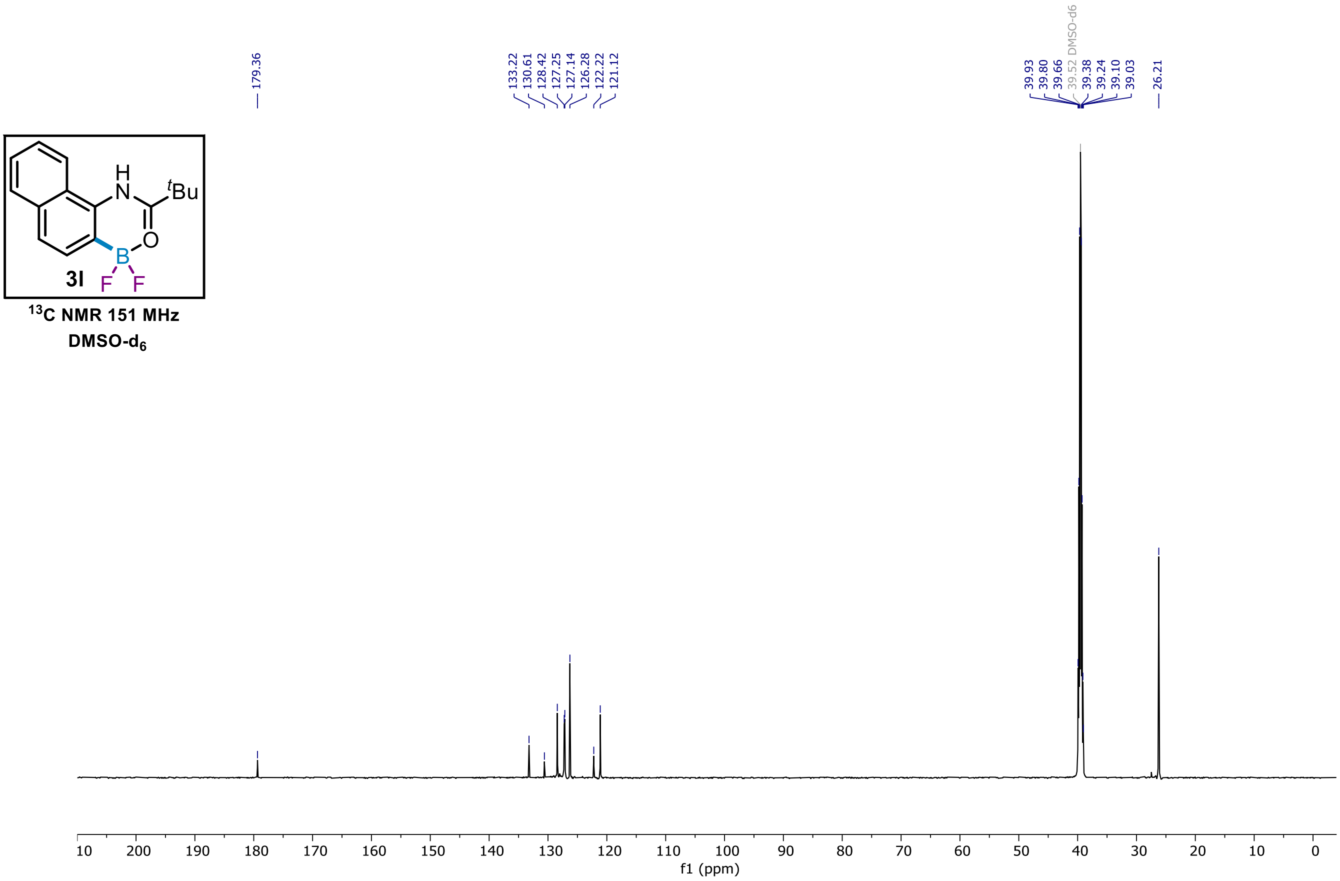

Figure S11-12: ^{13}C spectrum of compound **3l** in DMSO-d$_6$. Note that the ^{13}C signal for the C-BF_2 bond does not appear.

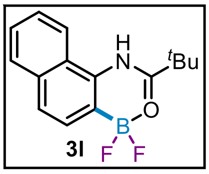

^{19}F NMR 659 MHz
DMSO-d$_6$

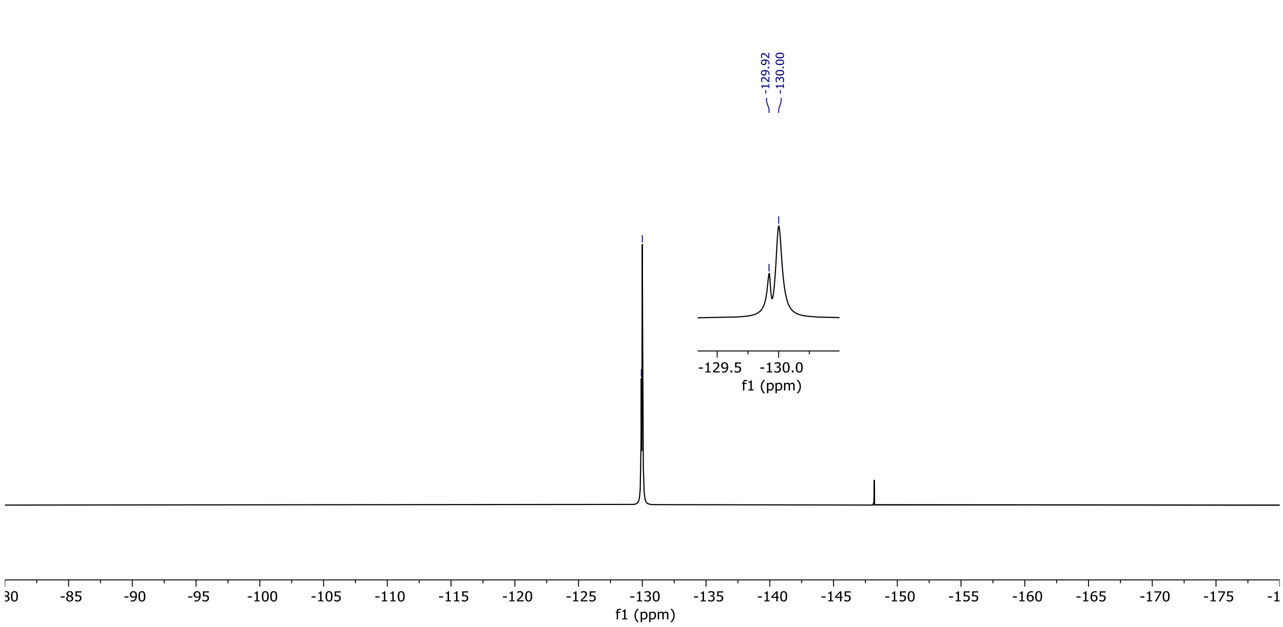

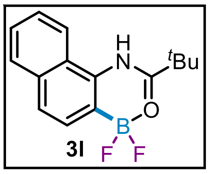

^{11}B NMR 193 MHz
DMSO-d$_6$

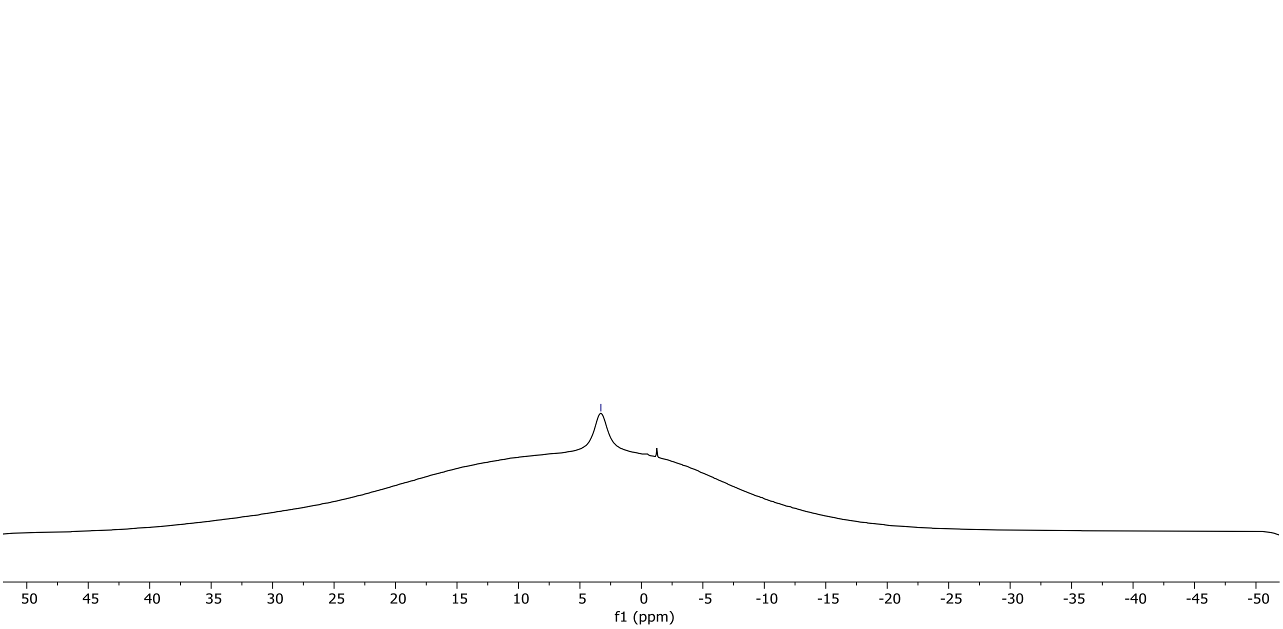

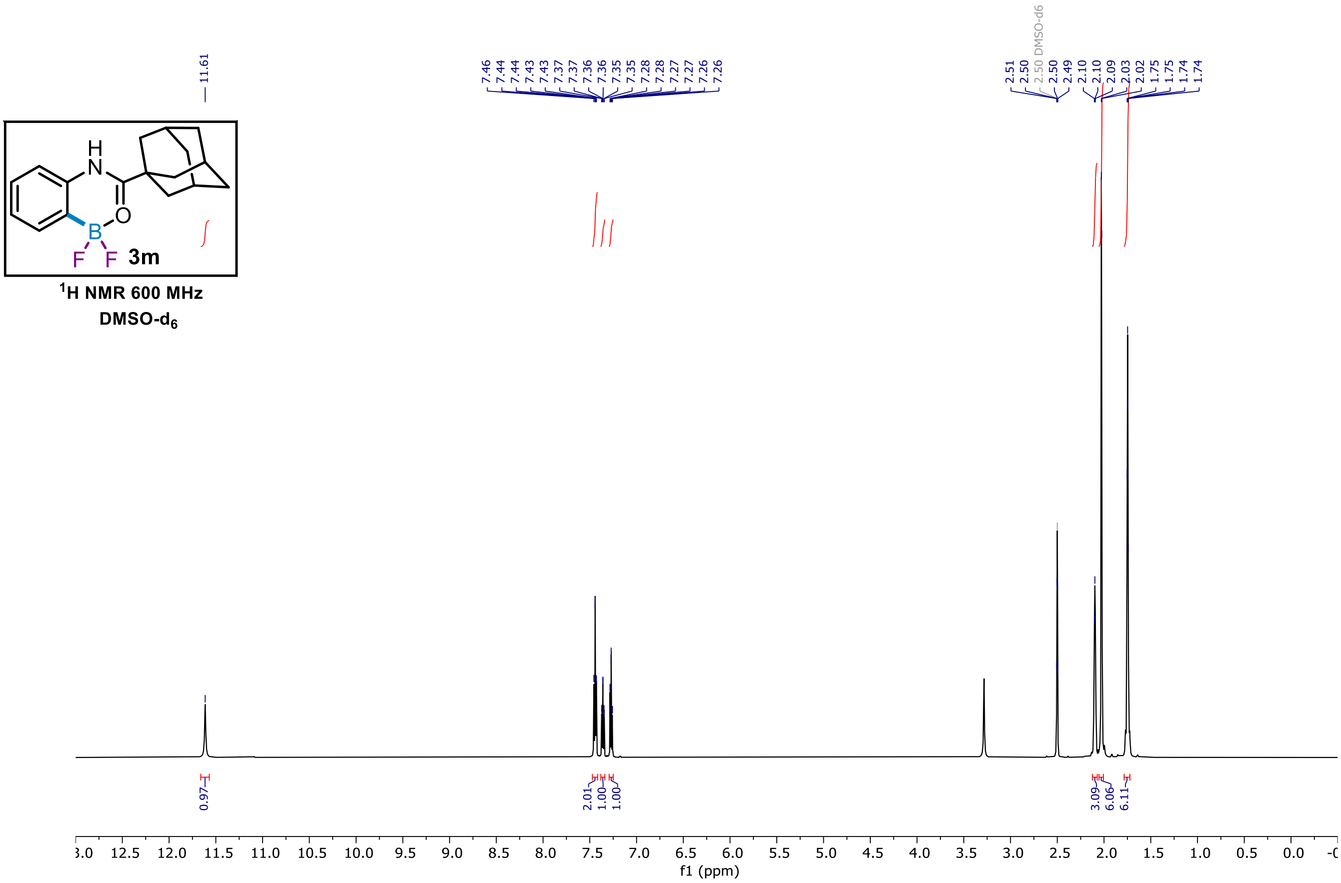

^{1}H NMR 600 MHz DMSO-d$_6$

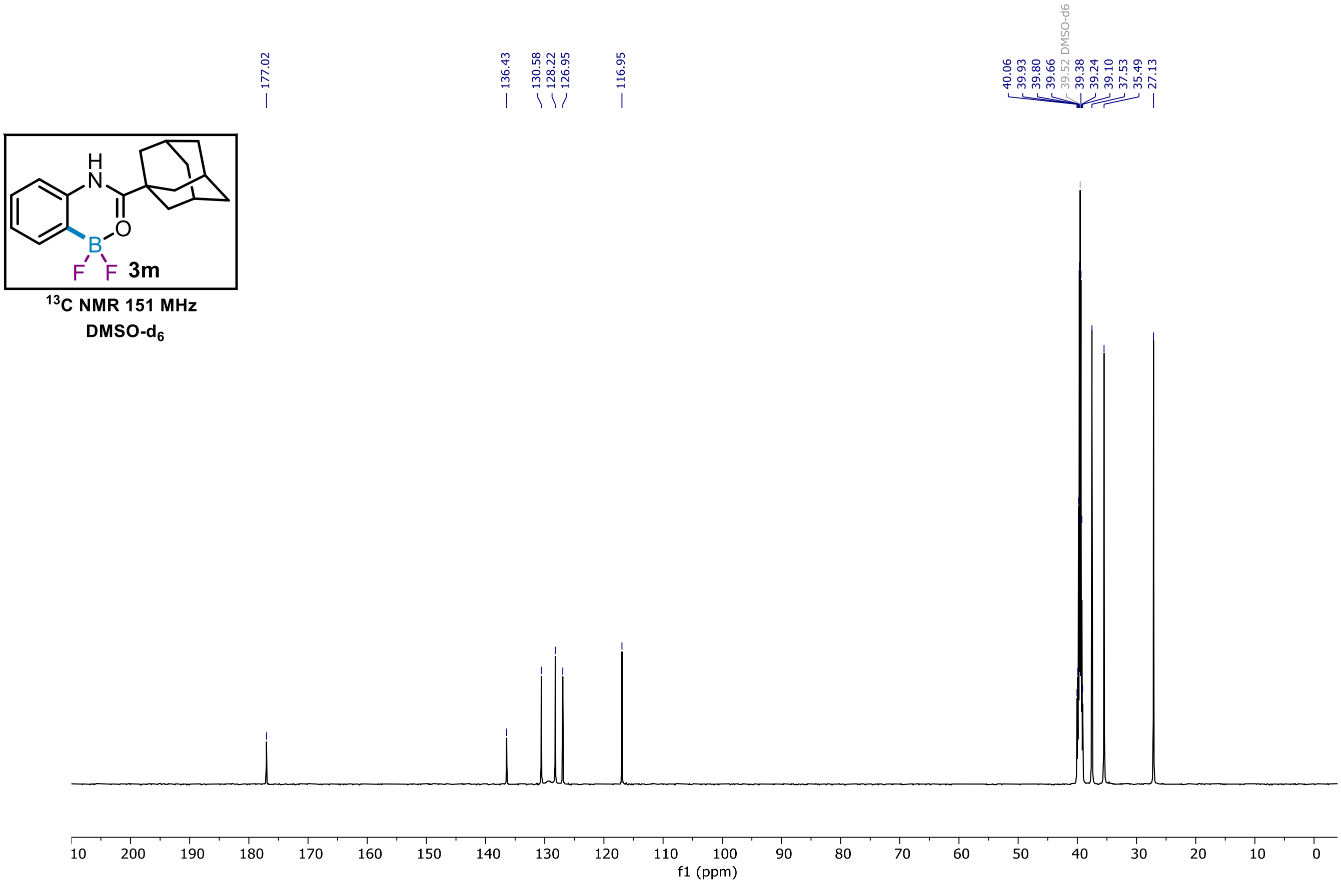

^{13}C NMR 151 MHz DMSO-d$_6$

Figure S11-13: ^{13}C spectrum of compound **3m** in DMSO-d$_6$. Note that the ^{13}C signal for the C-BF_2 bond does not appear.

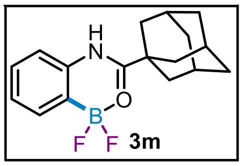

^{19}F NMR 659 MHz
DMSO-d_6

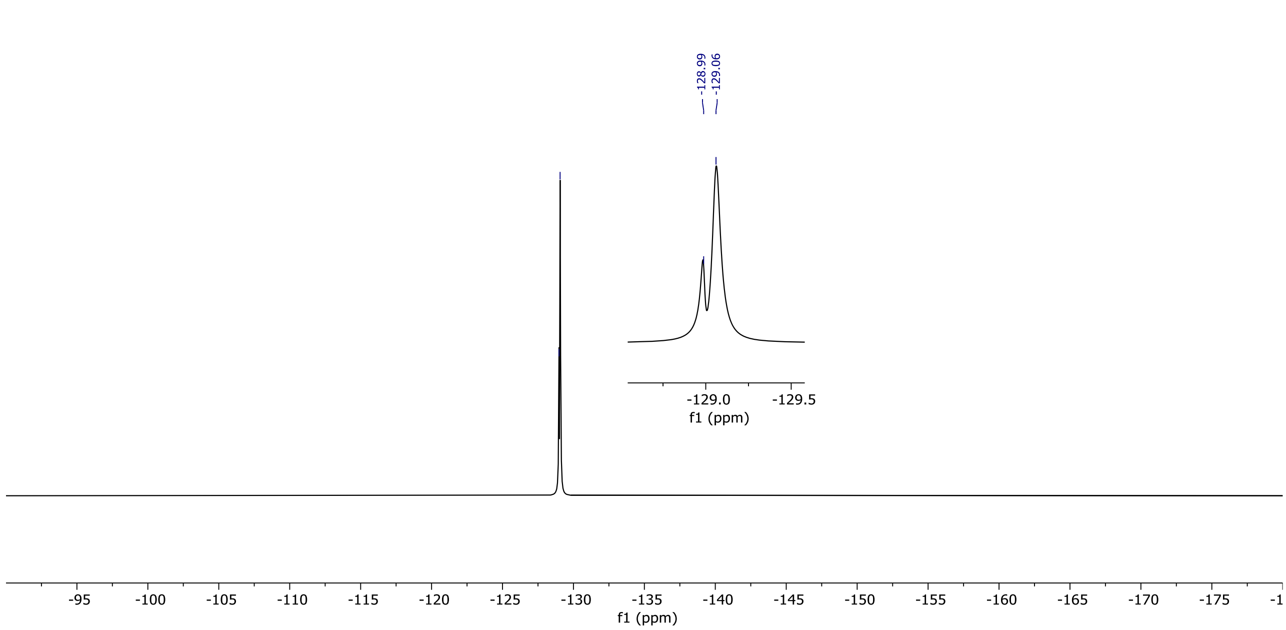

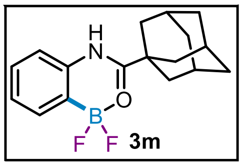

^{11}B NMR 193 MHz
DMSO-d_6

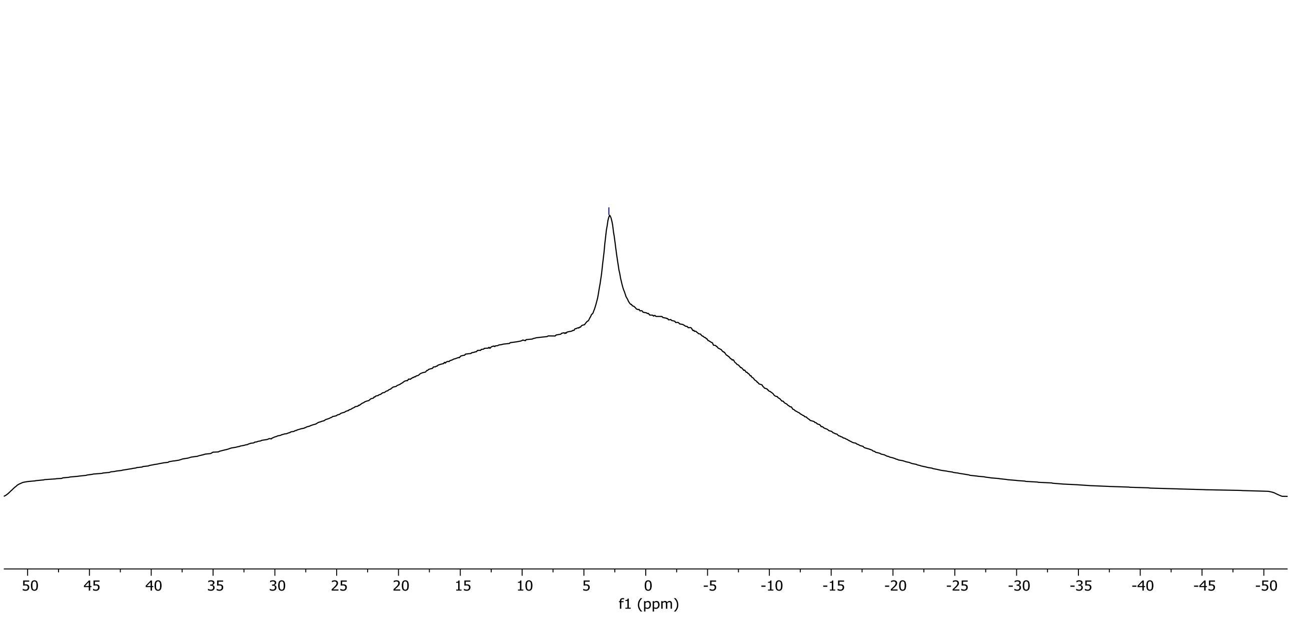

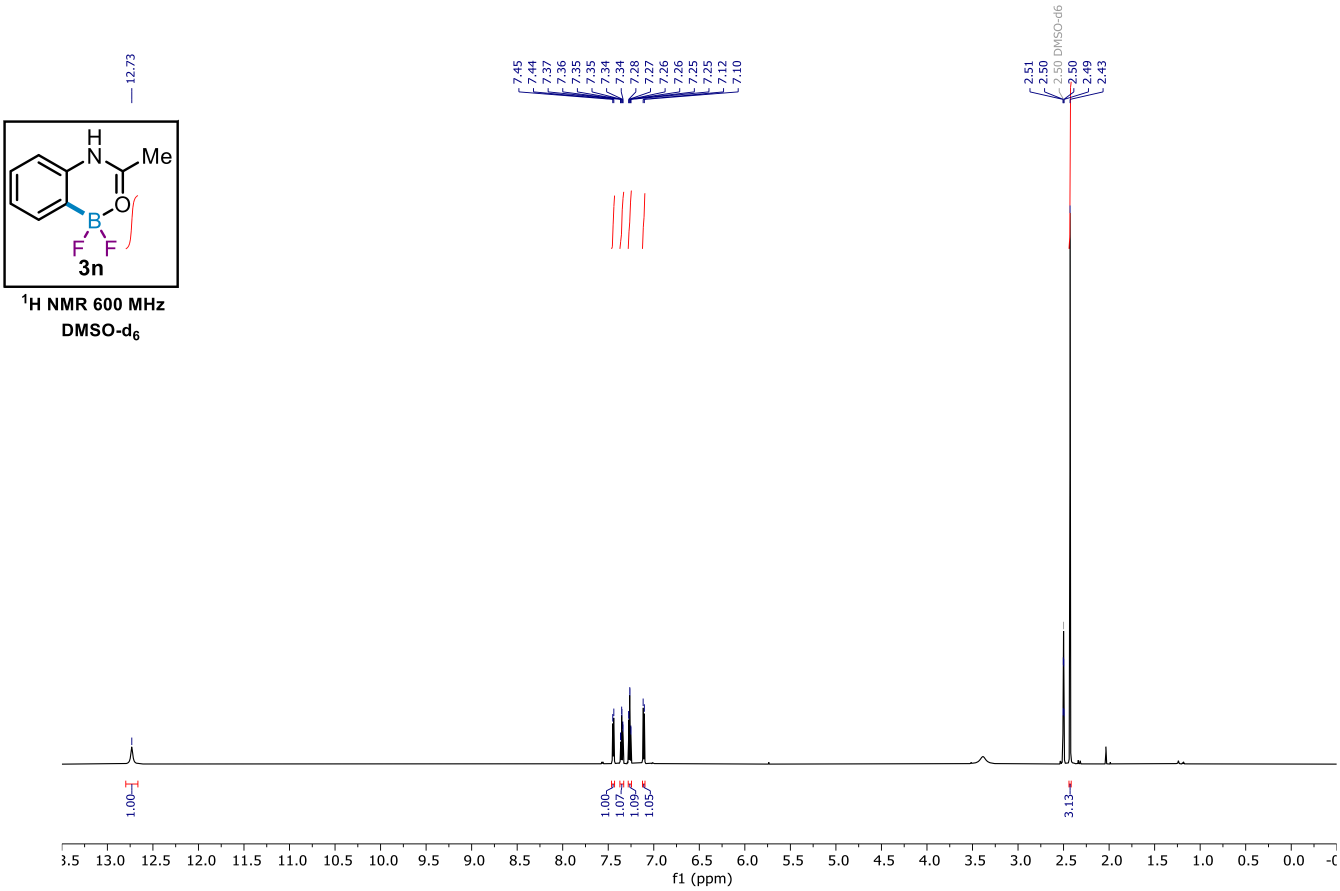

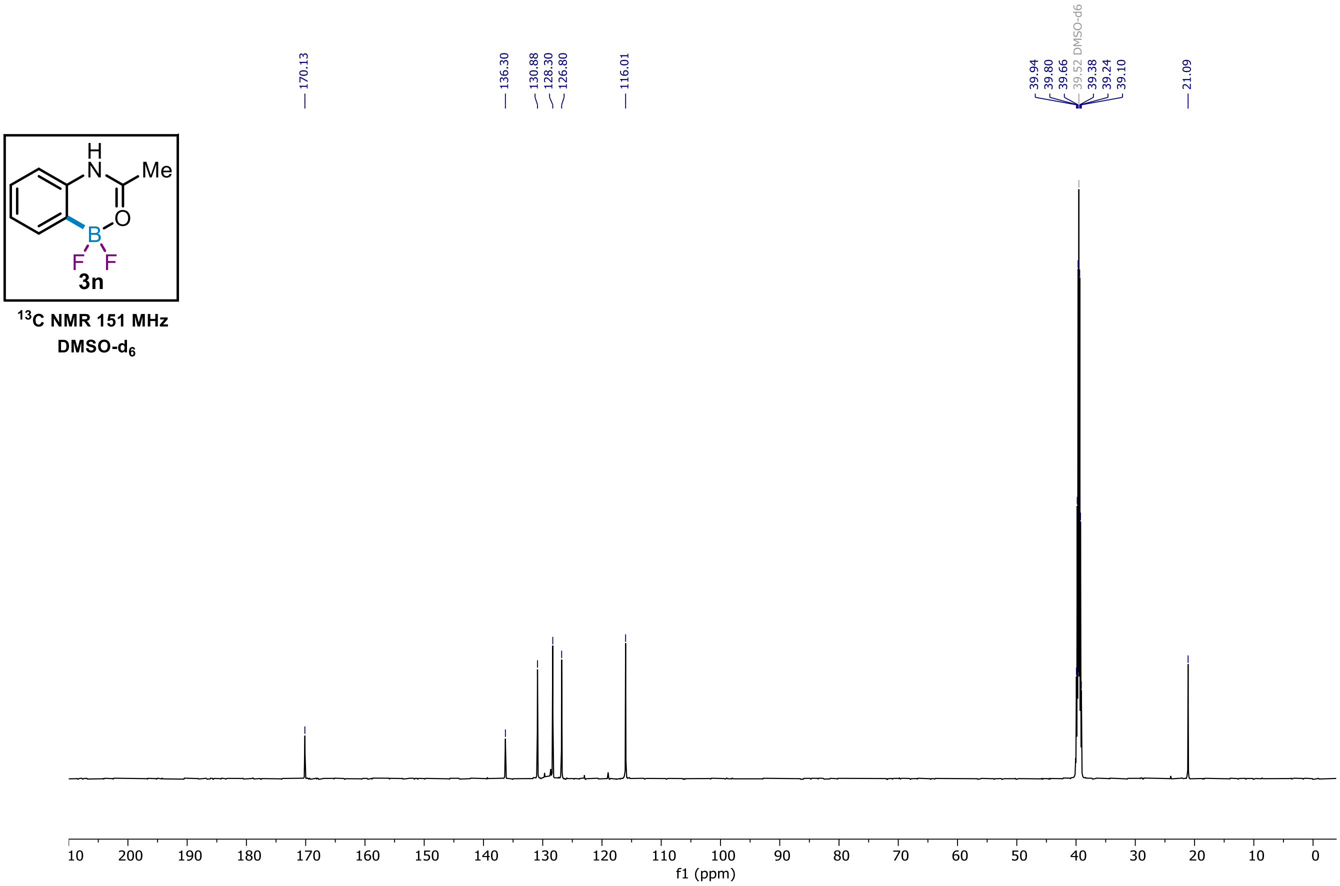

Figure S11-14: ^{13}C spectrum of compound **3n** in DMSO-d_6. Note that the ^{13}C signal for the C-BF_2 bond does not appear.

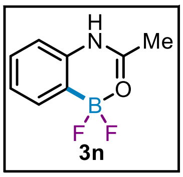

^{19}F NMR 659 MHz
DMSO-d_6

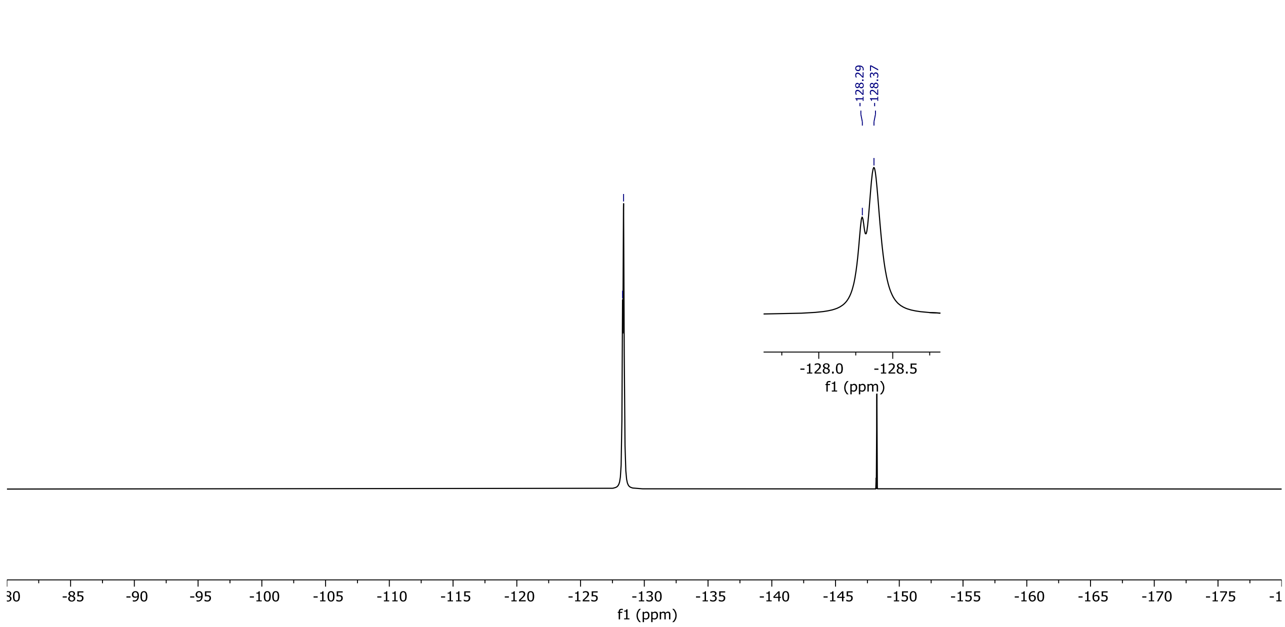

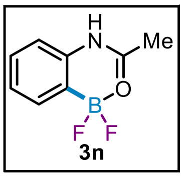

^{11}B NMR 193 MHz
DMSO-d_6

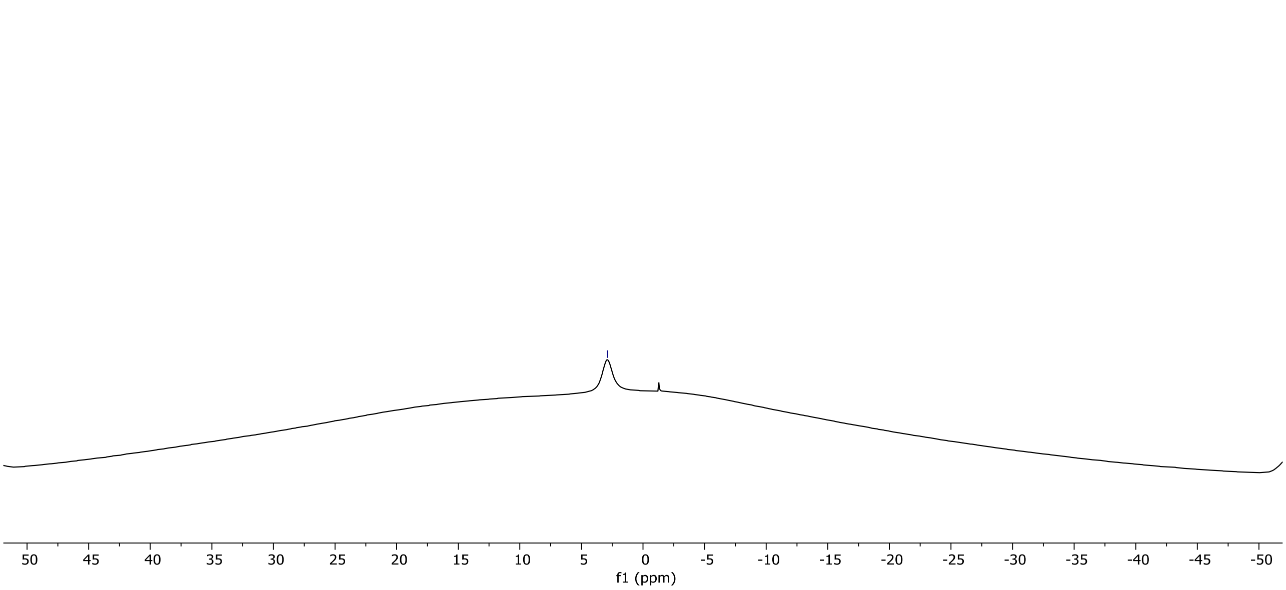

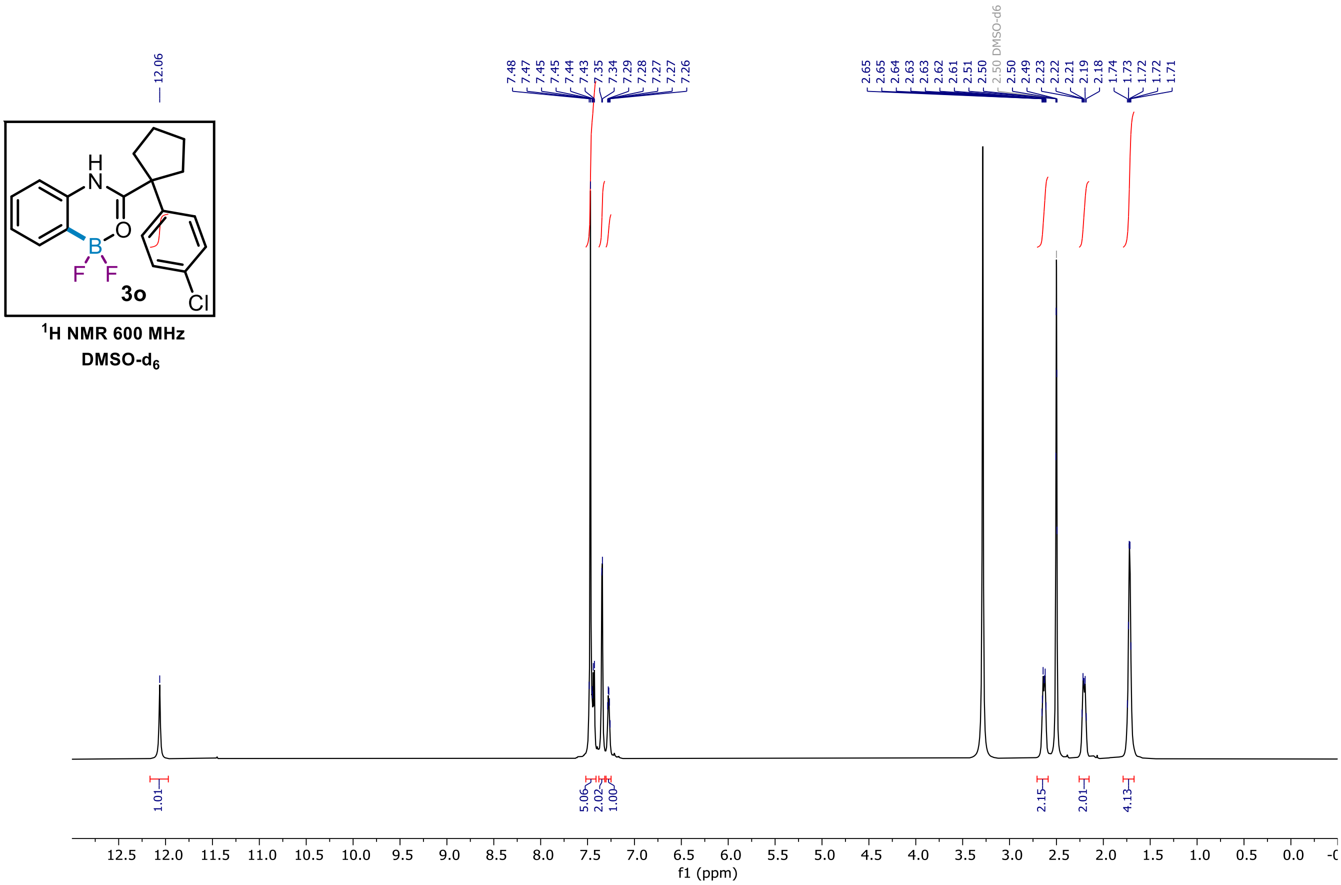

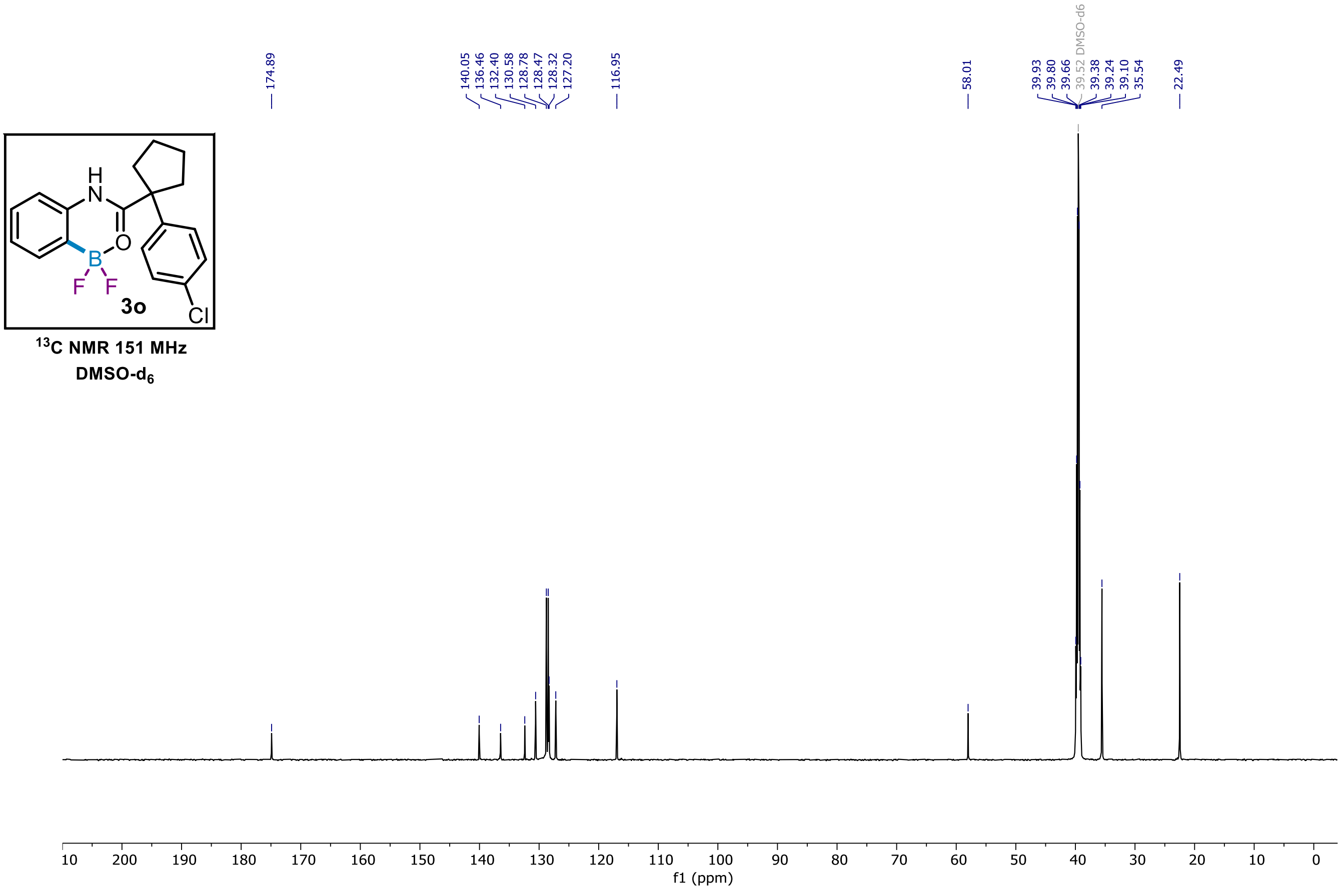

Figure S11-15: ^{13}C spectrum of compound **3o** in DMSO-d_6. Note that the ^{13}C signal for the C-BF_2 bond does not appear.

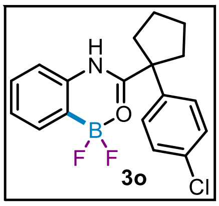

^{19}F NMR 659 MHz
DMSO-d$_6$

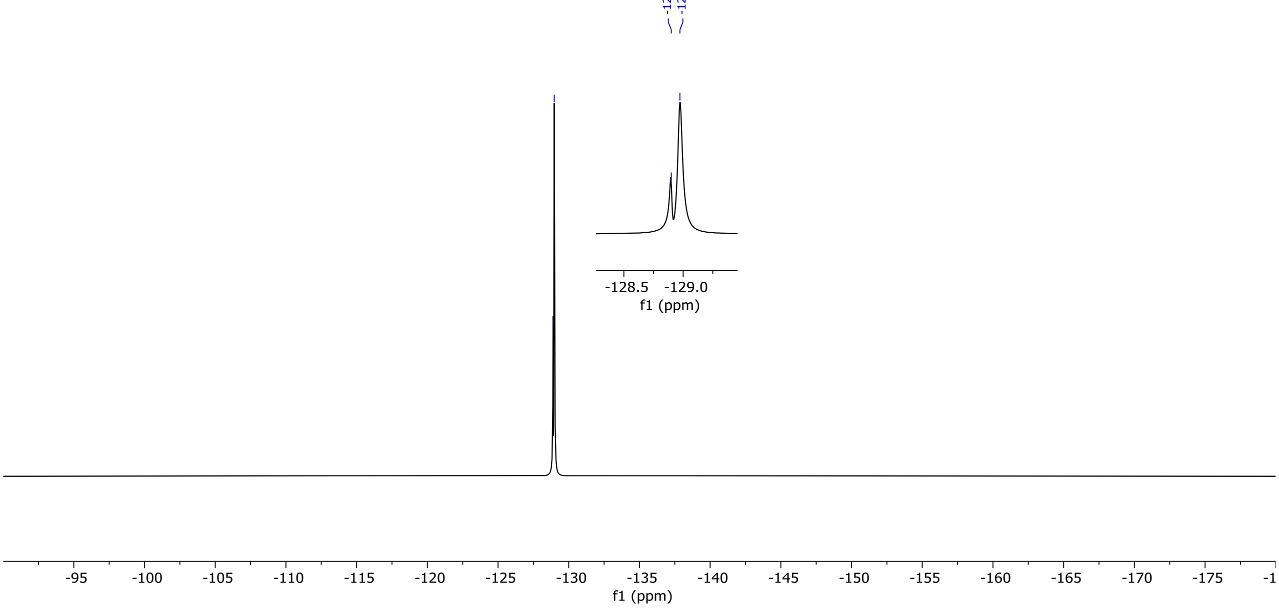

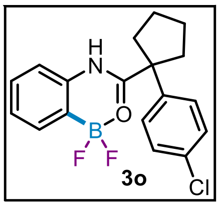

^{11}B NMR 193 MHz
DMSO-d$_6$

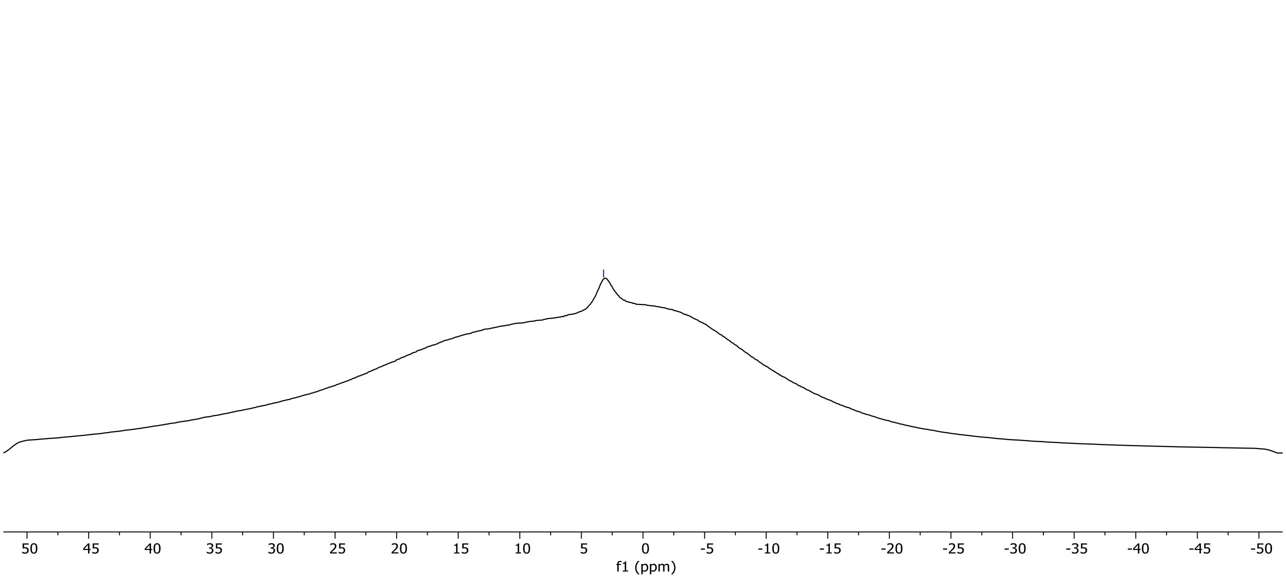

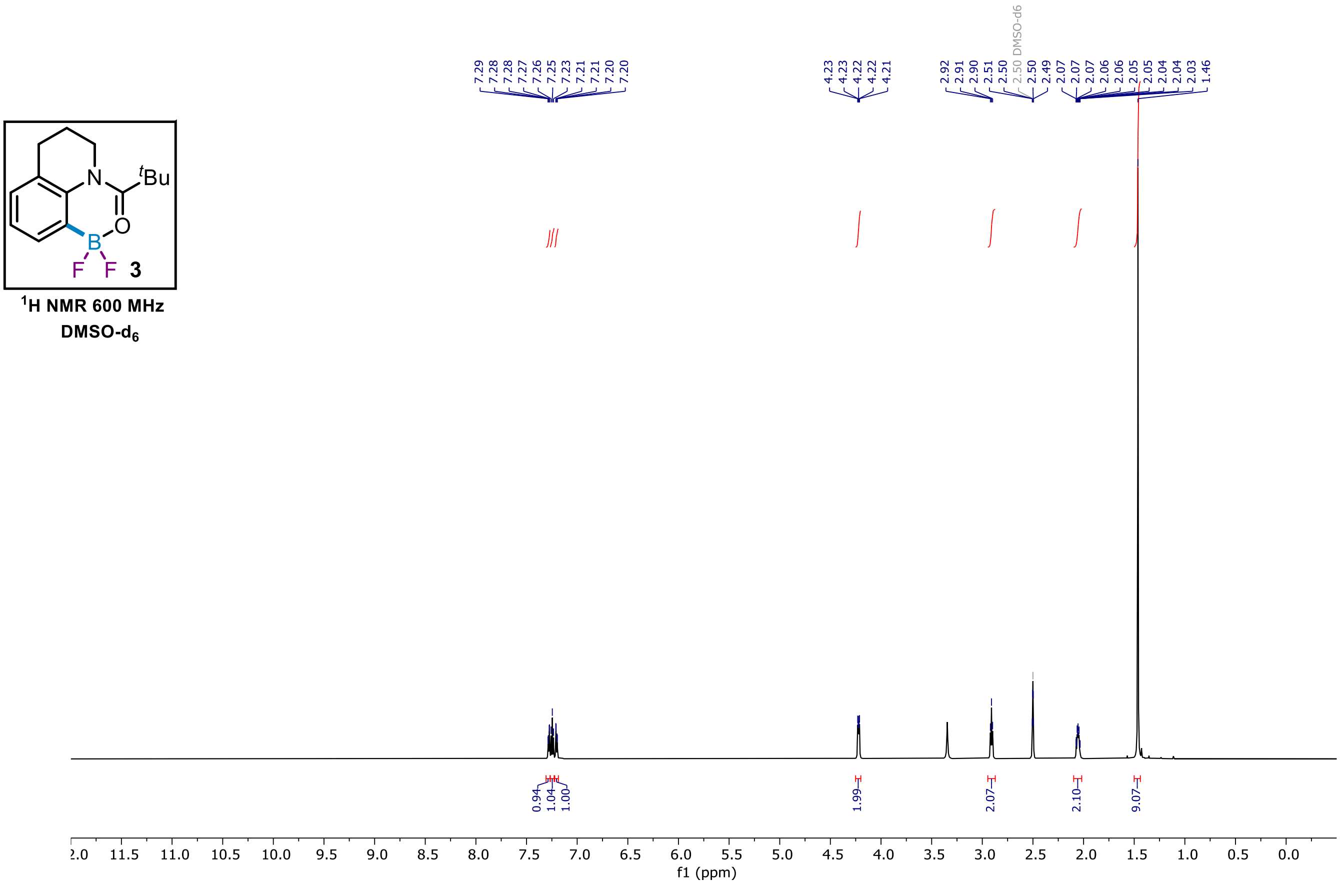

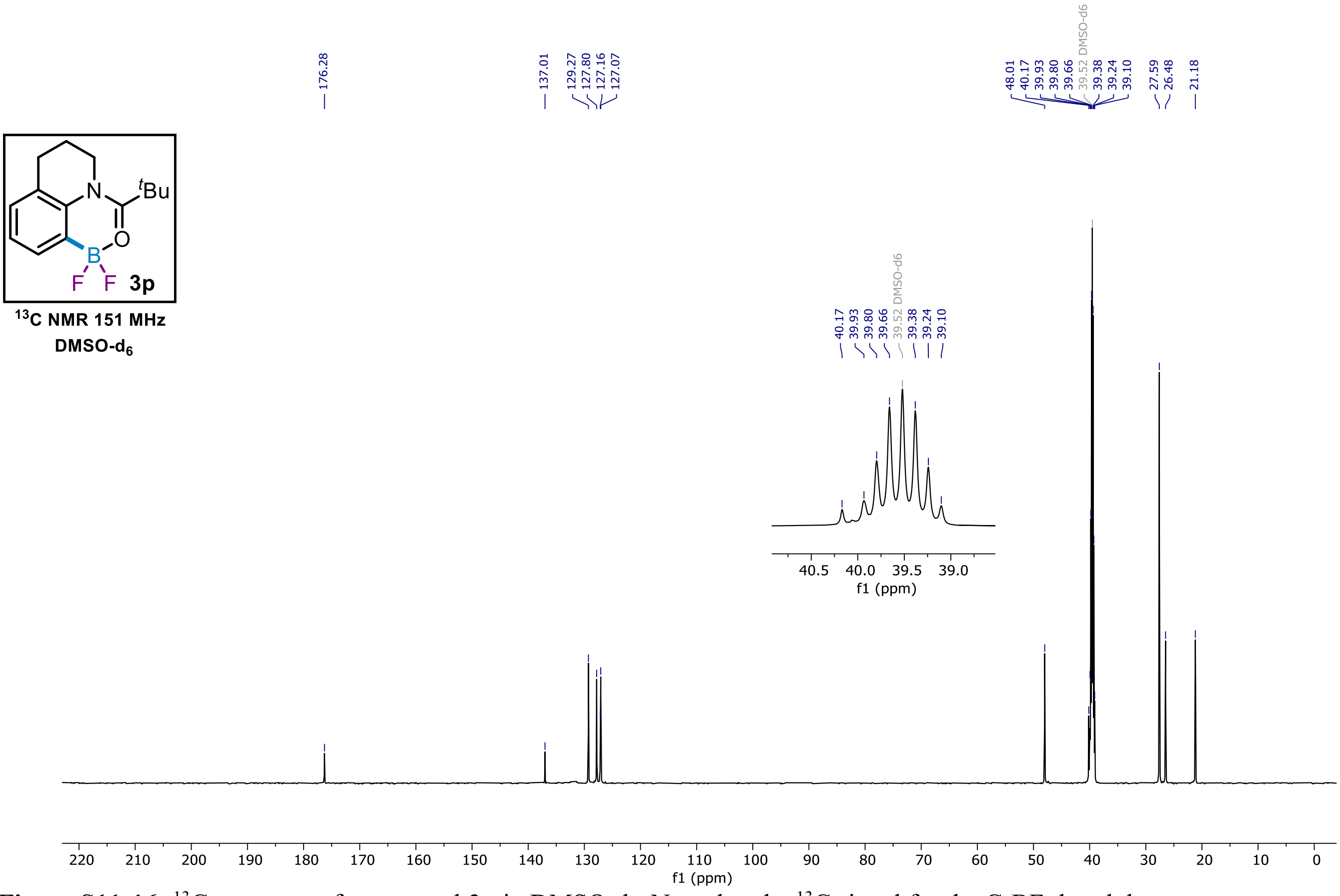

Figure S11-16: ^{13}C spectrum of compound **3p** in DMSO-d$_6$. Note that the ^{13}C signal for the C-BF$_2$ bond does not appear.

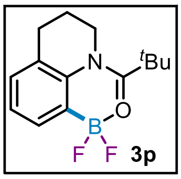

^{19}F NMR 564 MHz
DMSO-d_6

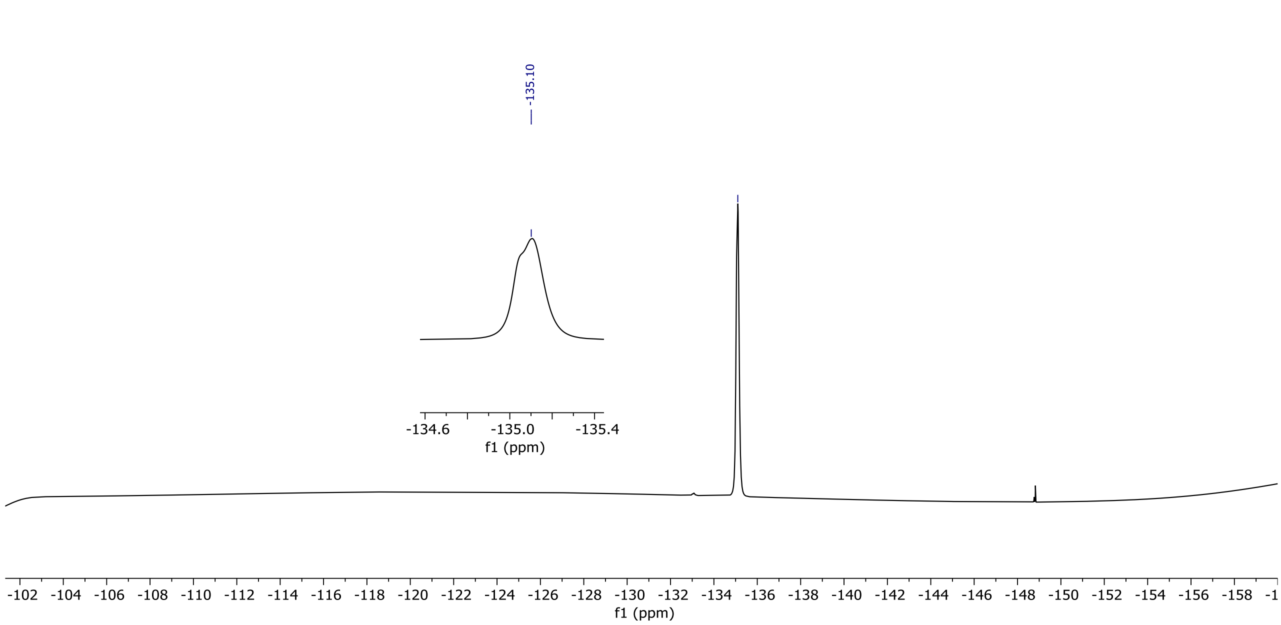

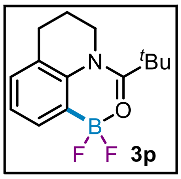

^{11}B NMR 193 MHz
DMSO-d_6

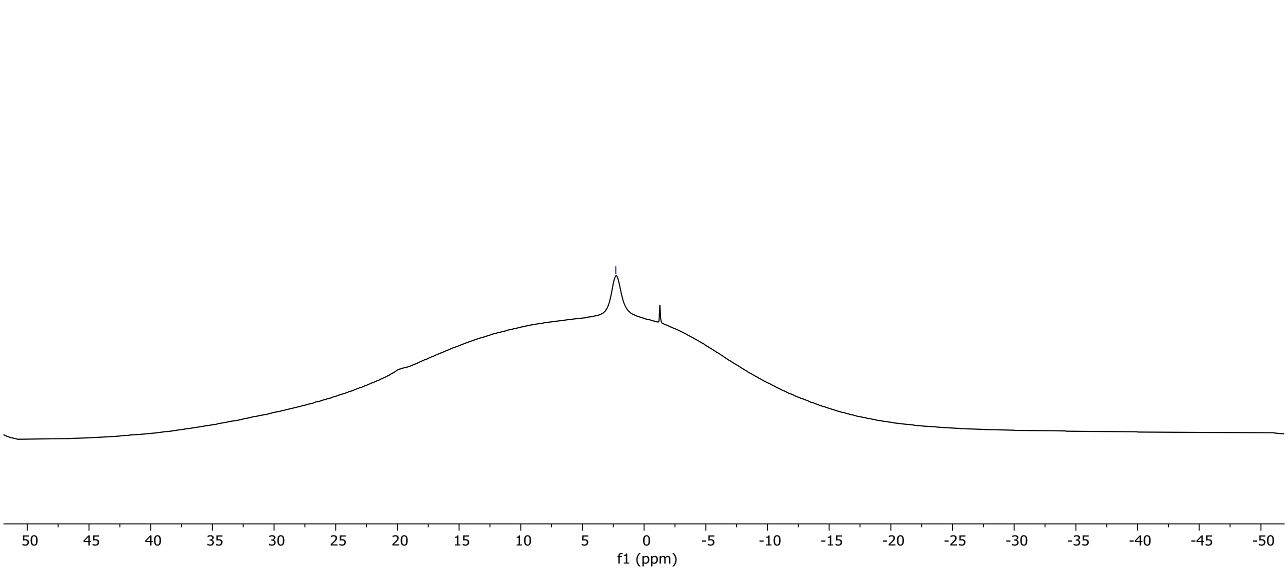

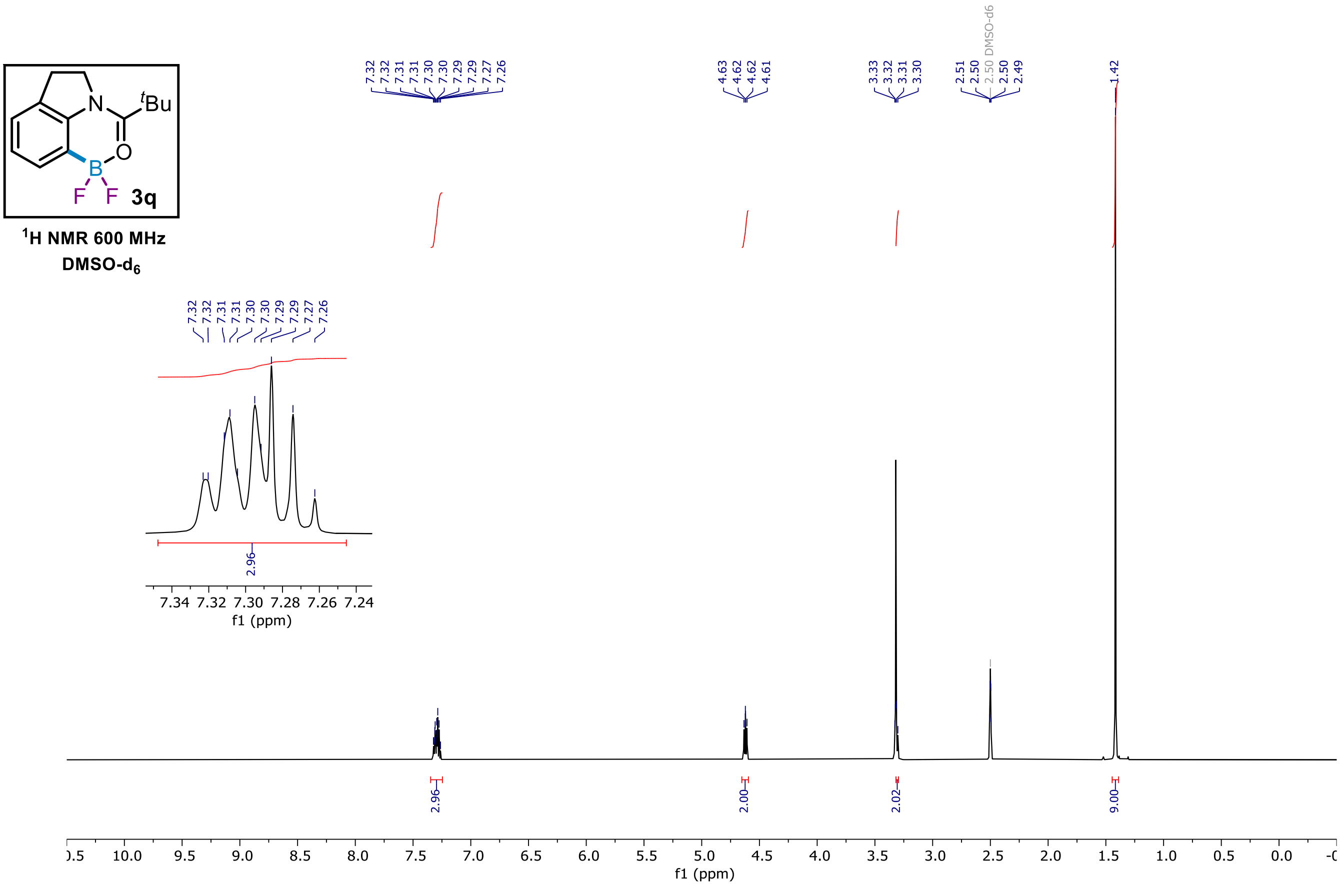

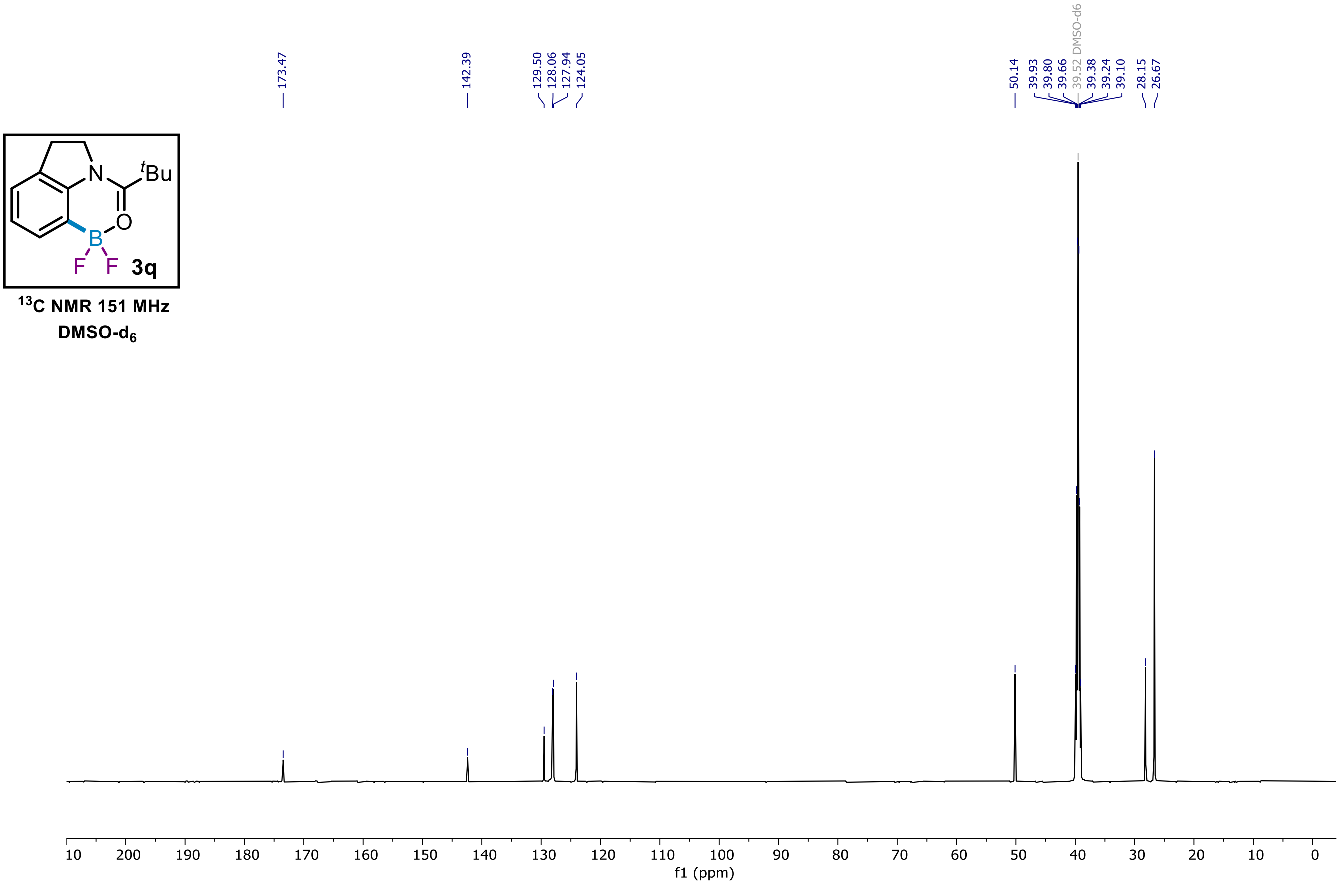

Figure S11-17: ^{13}C spectrum of compound **3q** in DMSO-d$_6$. Note that the ^{13}C signal for the C-BF$_2$ bond does not appear.

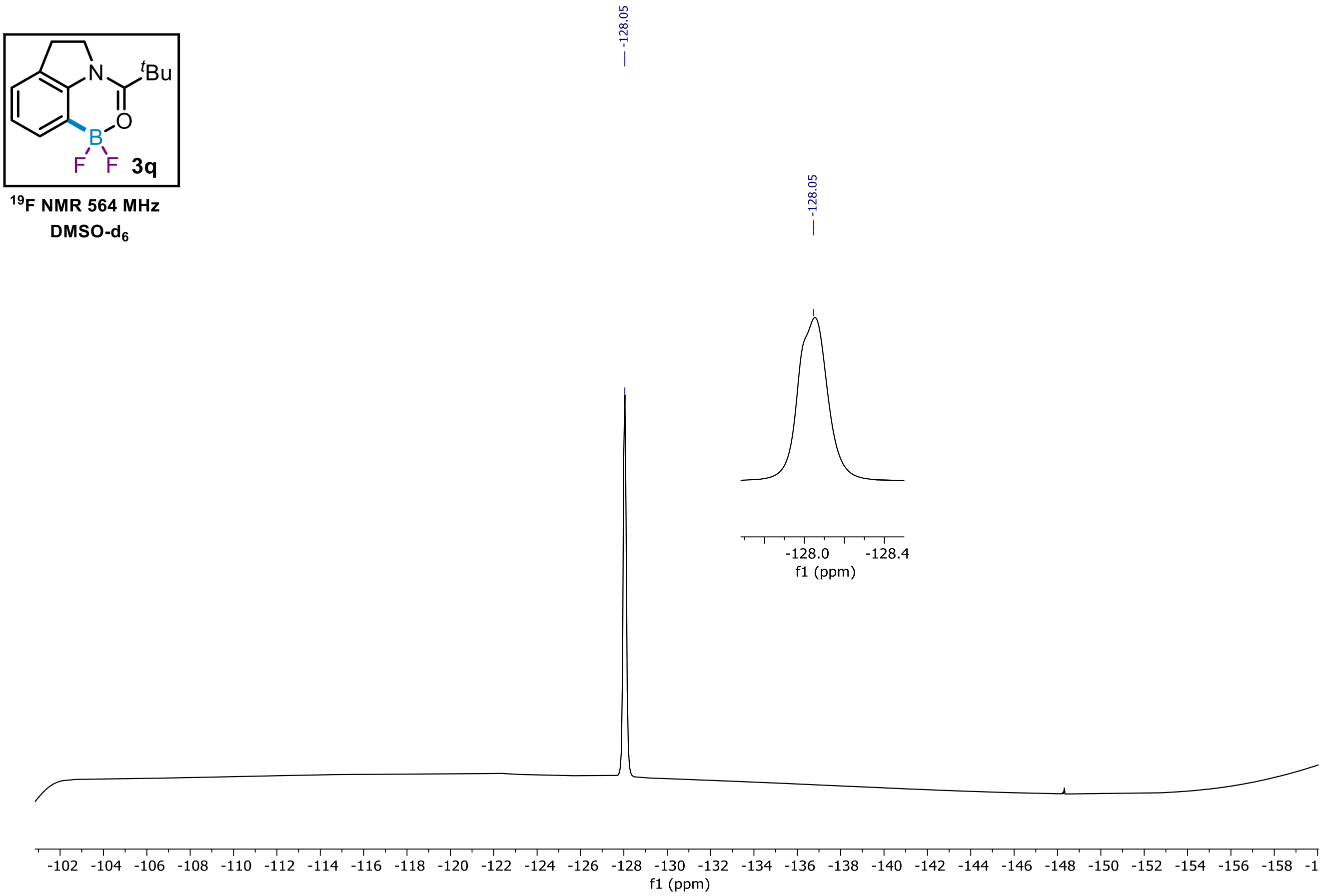
tBu
N
O
B
F F 3q
19F NMR 564 MHz
DMSO-d6
-128.05
-128.05
-128.0 -128.4
f1 (ppm)
-102 -104 -106 -108 -110 -112 -114 -116 -118 -120 -122 -124 -126 -128 -130 -132 -134 -136 -138 -140 -142 -144 -146 -148 -150 -152 -154 -156 -158
f1 (ppm)

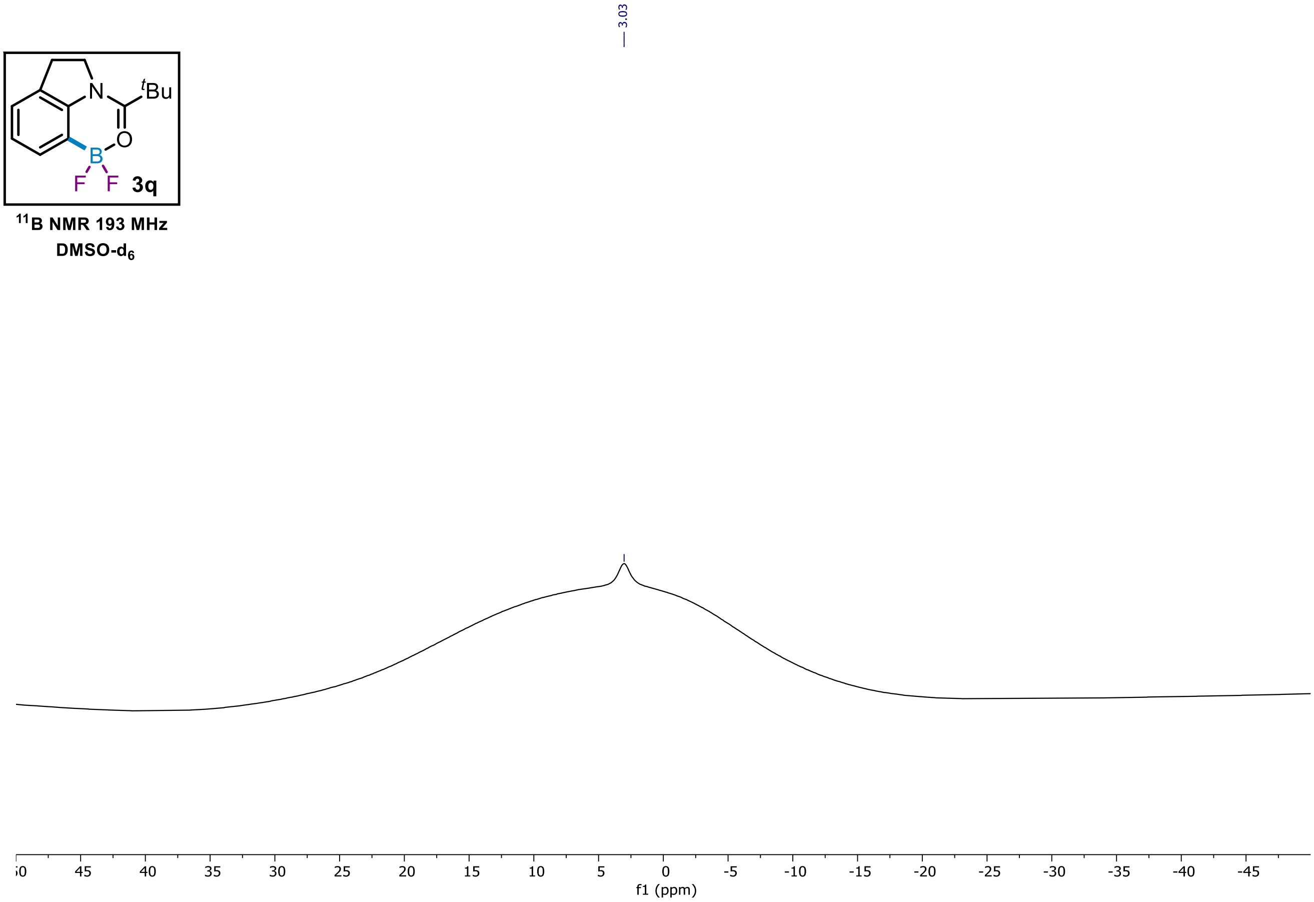
tBu
N
O
B
F F 3q
11B NMR 193 MHz
DMSO-d6
3.03
45 40 35 30 25 20 15 10 5 0 -5 -10 -15 -20 -25 -30 -35 -40 -45
f1 (ppm)

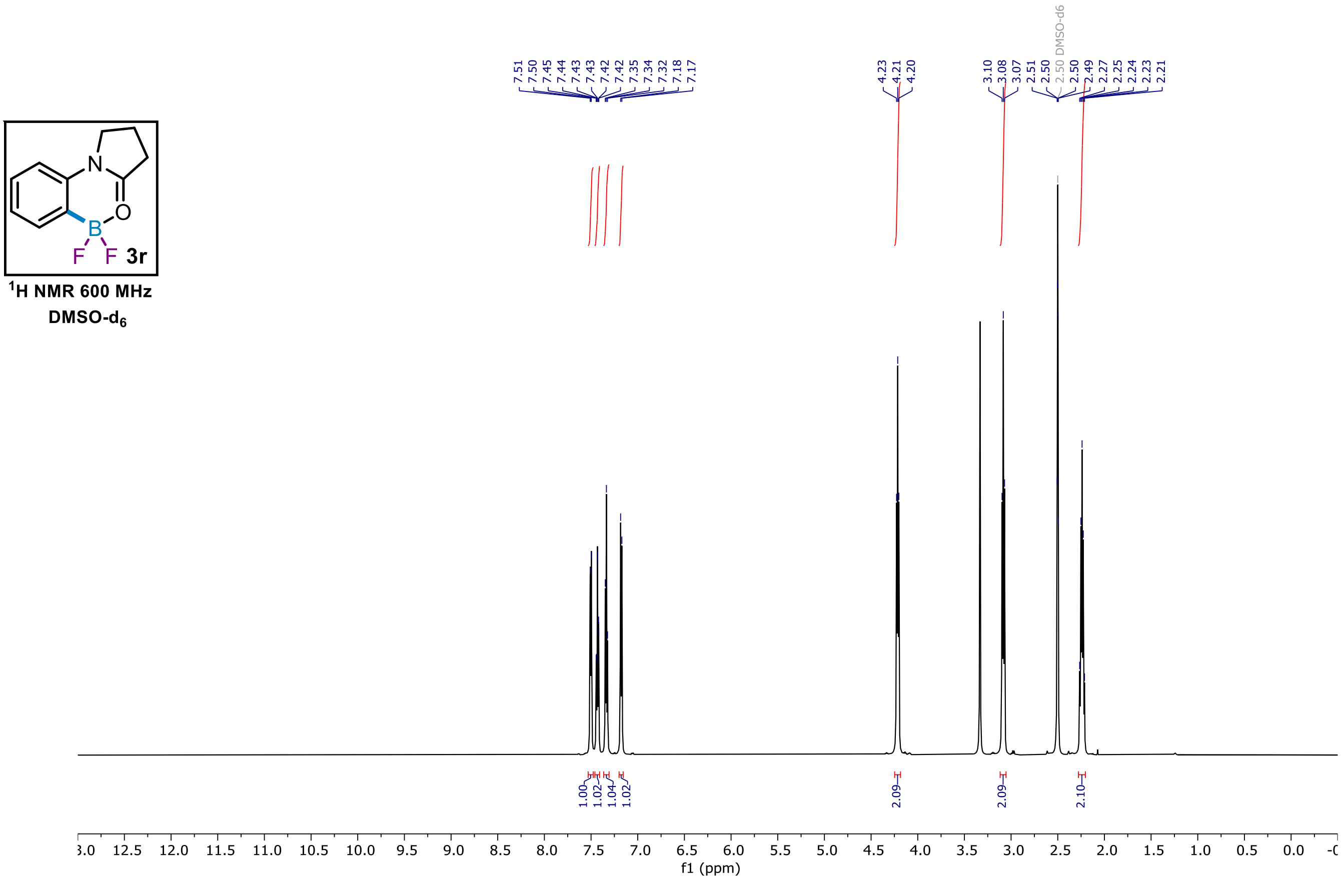

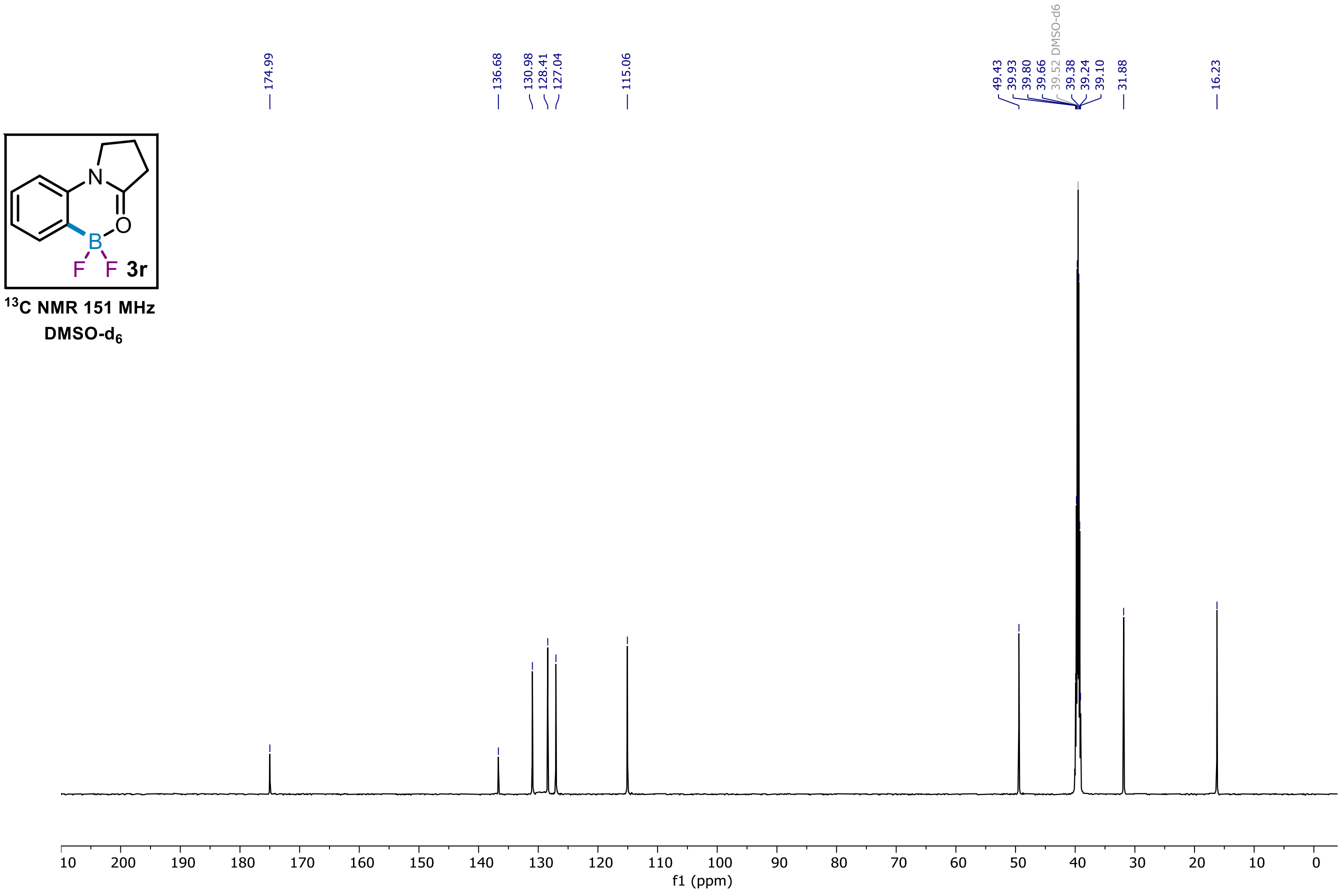

Figure S11-18: ^{13}C spectrum of compound **3r** in DMSO-d_6. Note that the ^{13}C signal for the C-BF_2 bond does not appear.

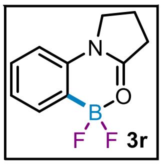

3r

^{19}F NMR 659 MHz
DMSO-d6

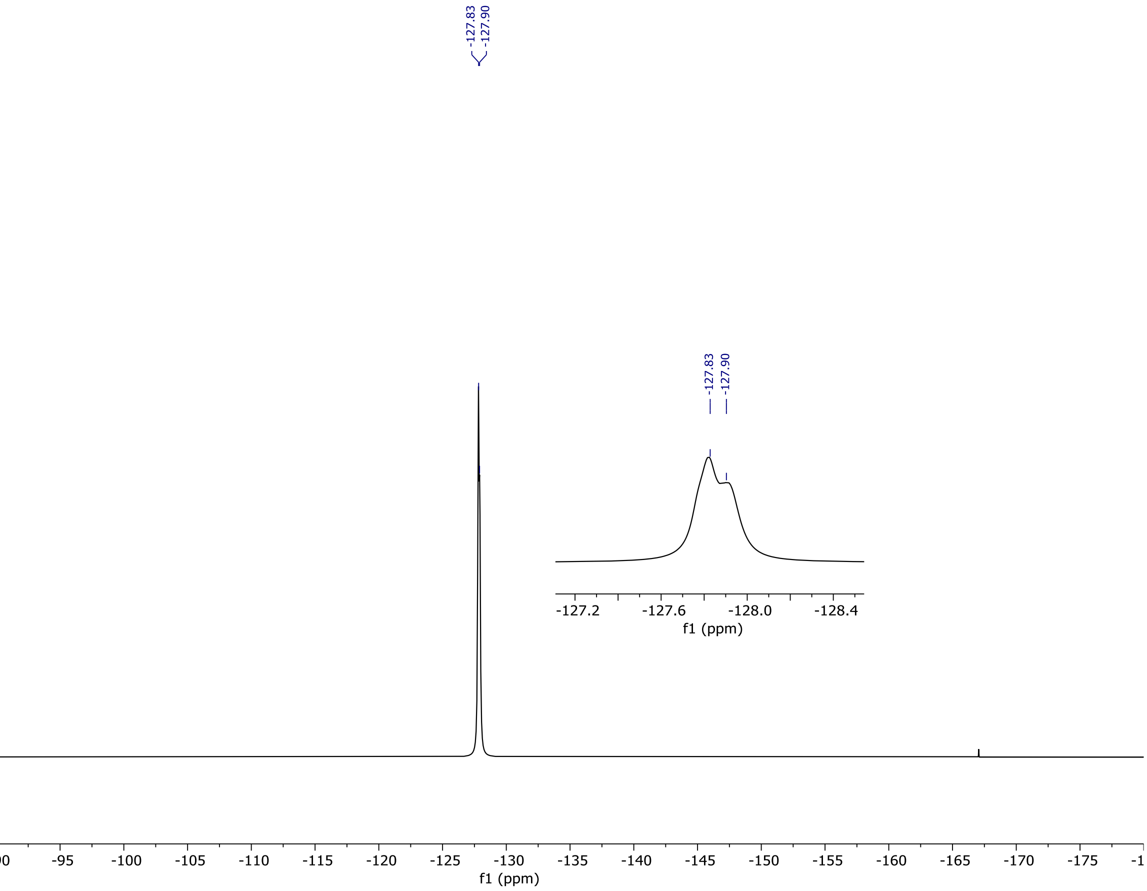

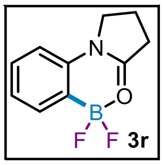

3r

^{11}B NMR 193 MHz
DMSO-d6

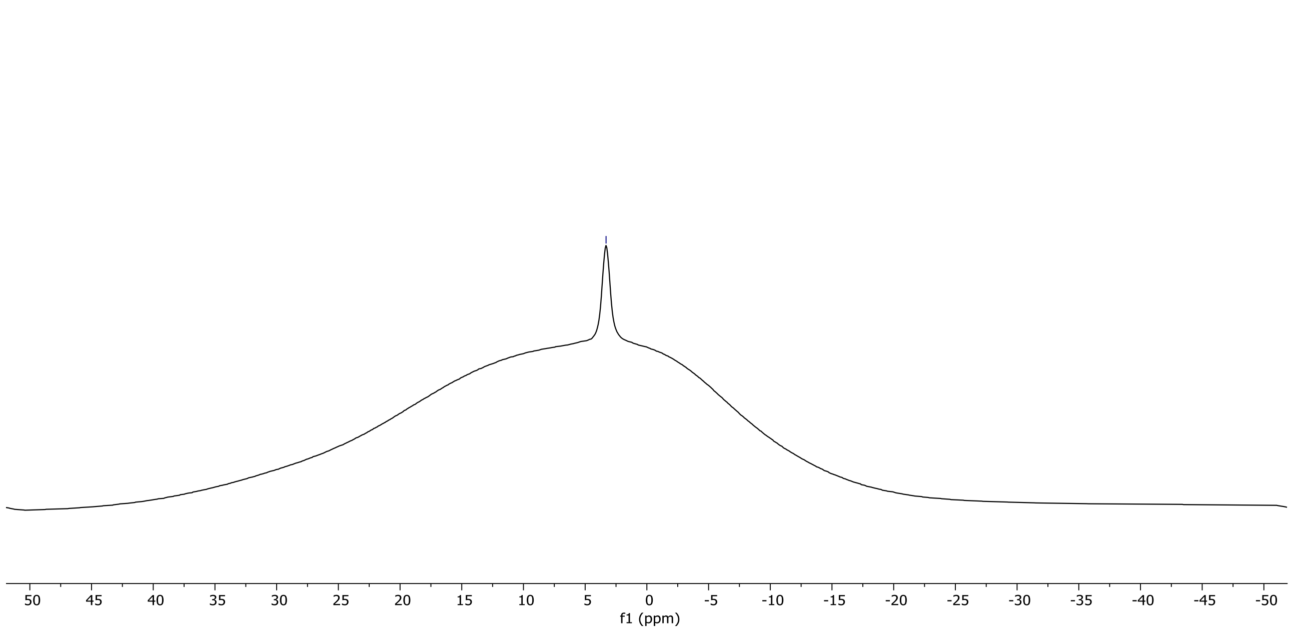

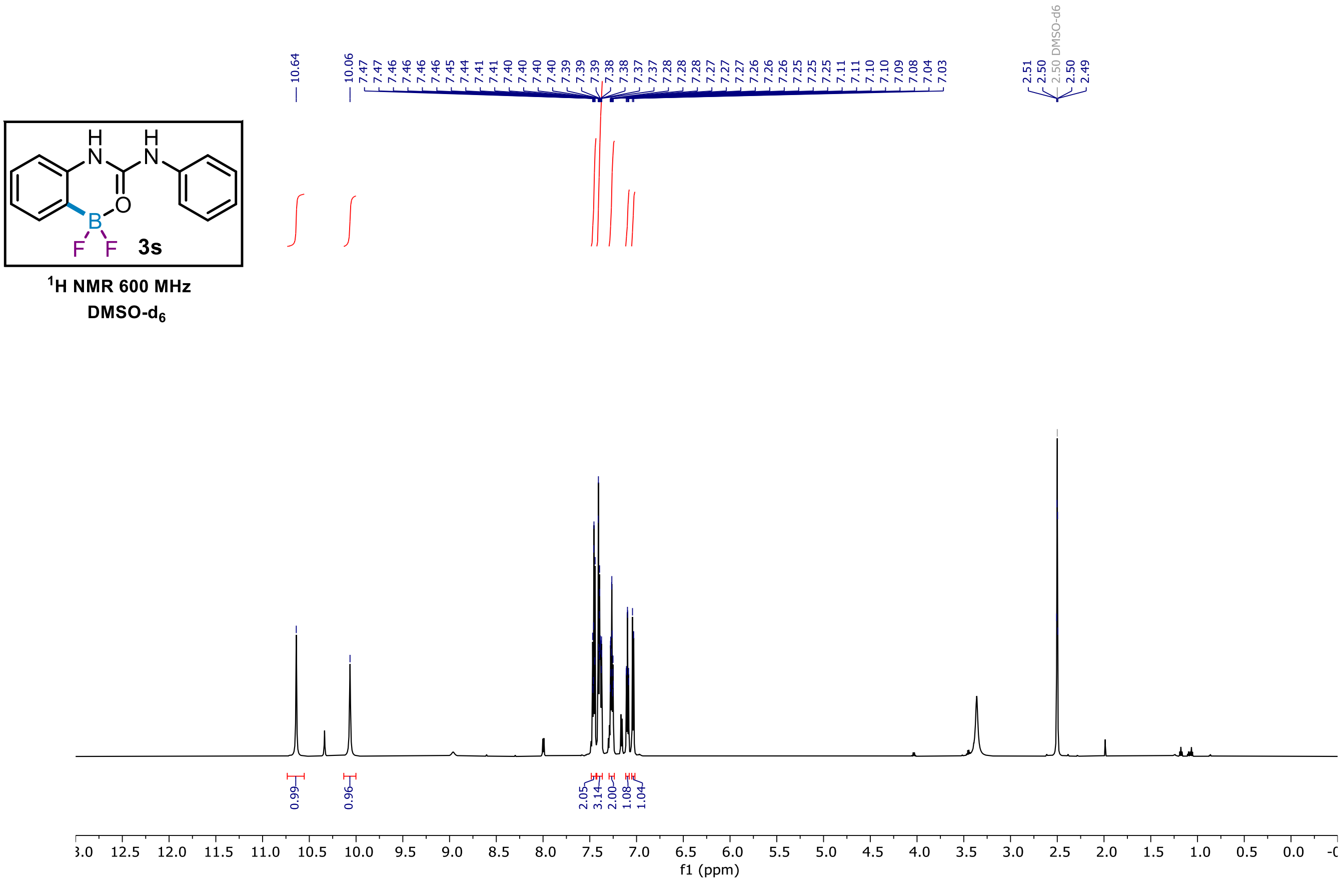

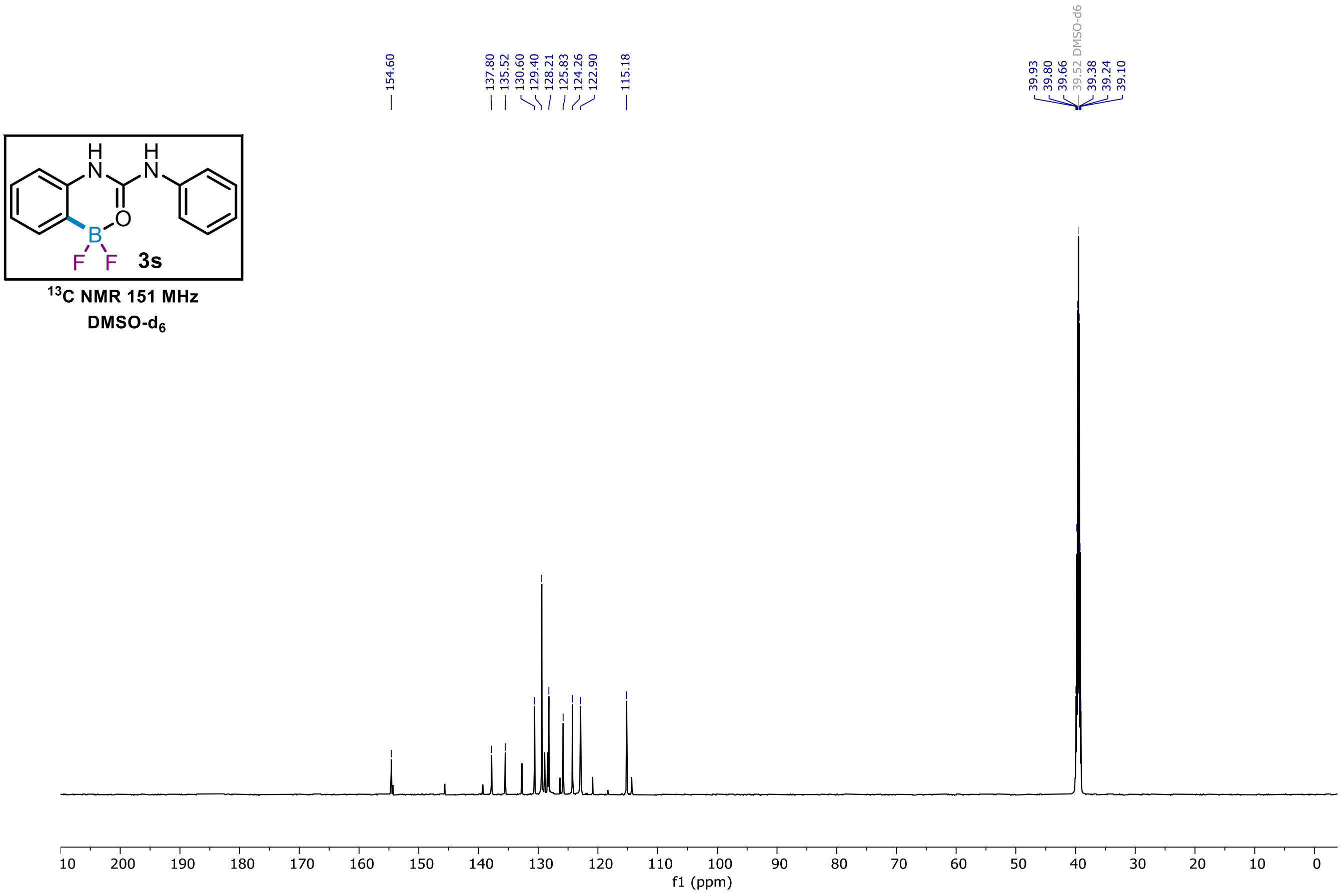

Figure S11-19: ^{13}C spectrum of compound **3s** in DMSO-d$_6$. Note that the ^{13}C signal for the C-BF_2 bond does not appear.

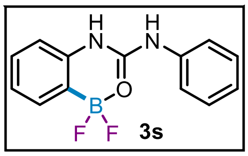

^{19}F NMR 659 MHz
DMSO-d_6

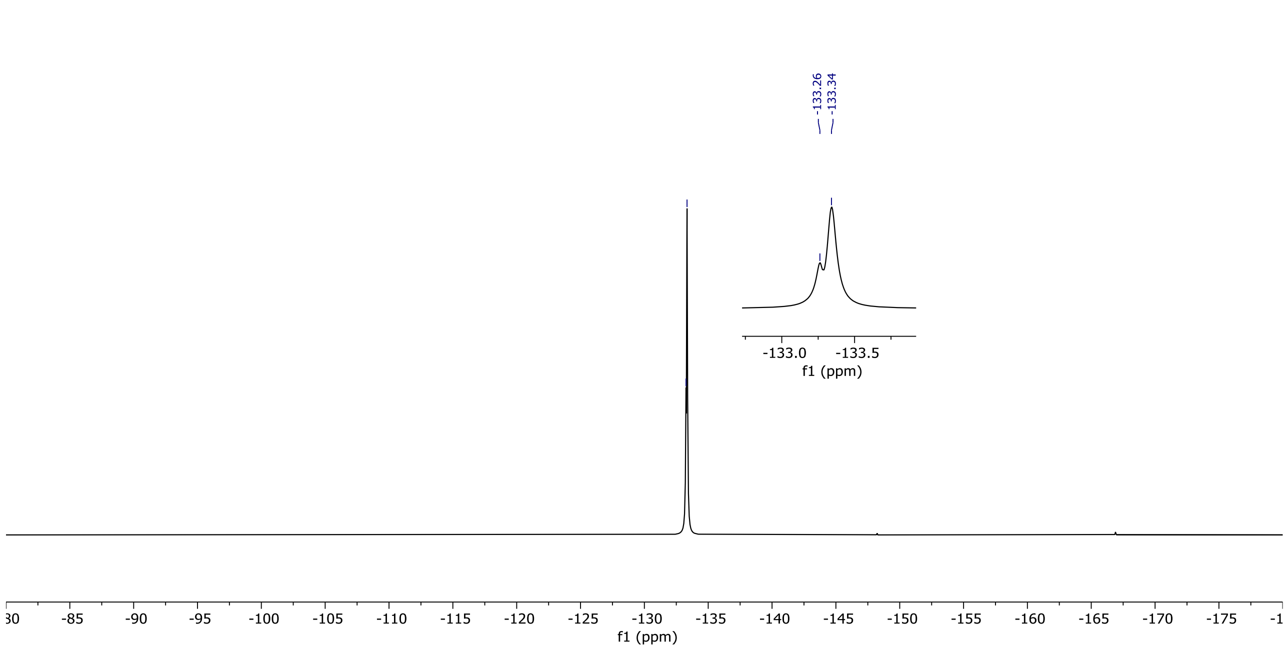

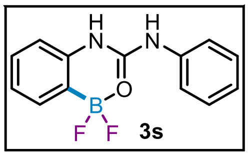

^{11}B NMR 193 MHz
DMSO-d_6

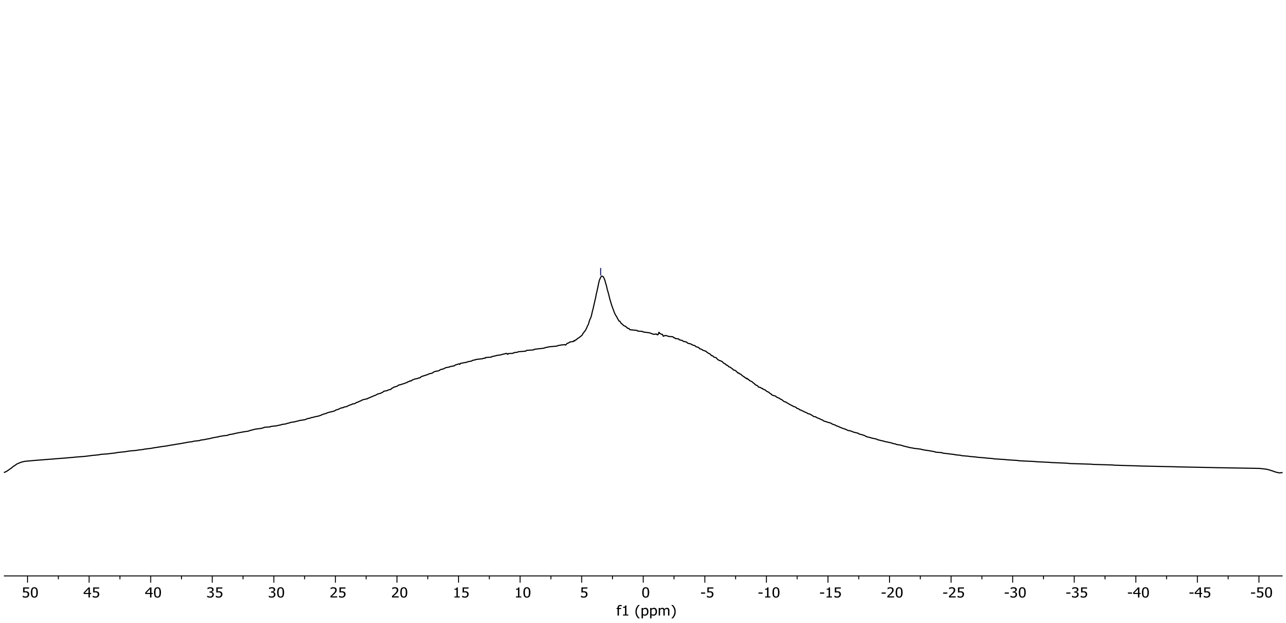

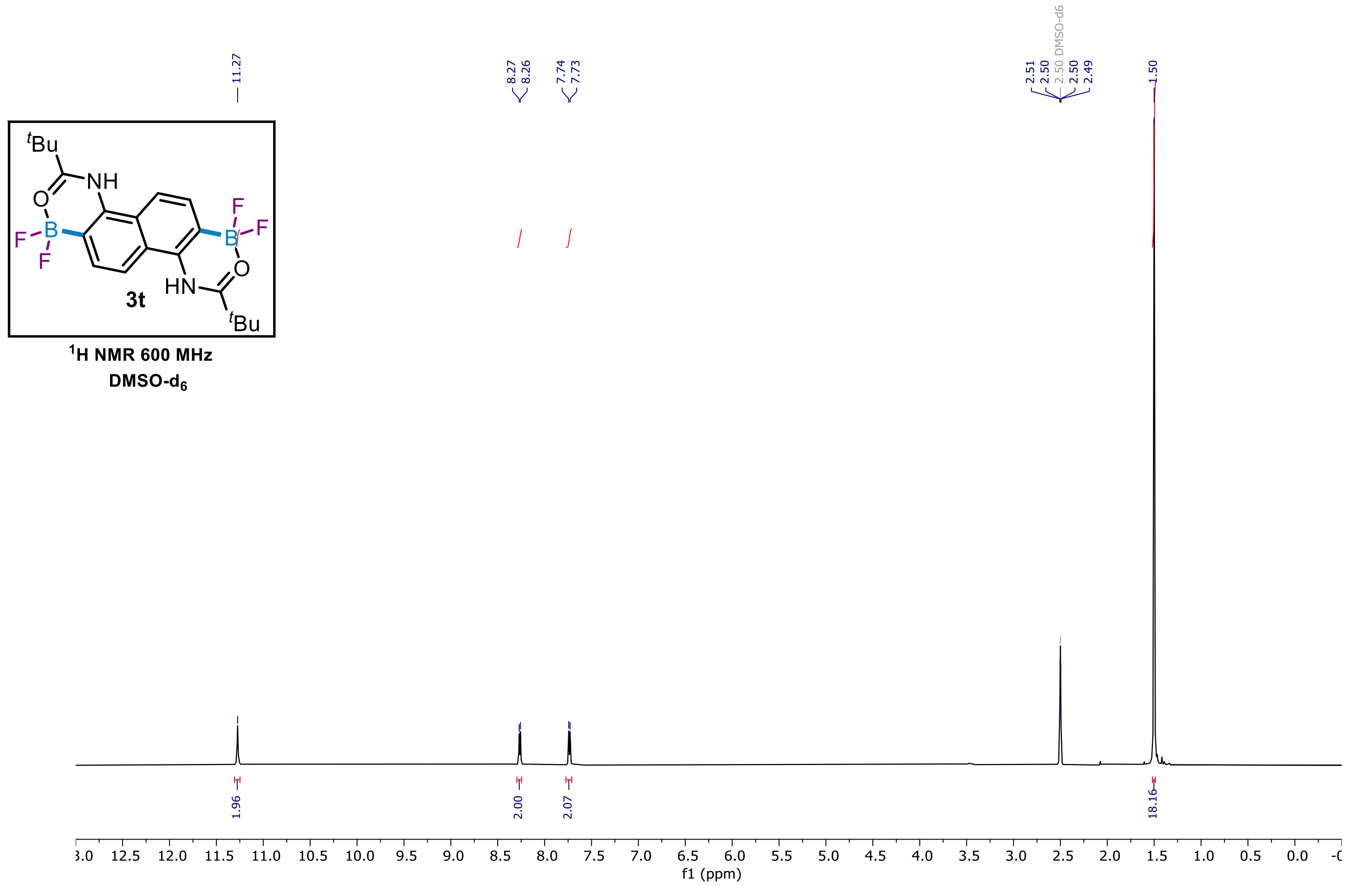

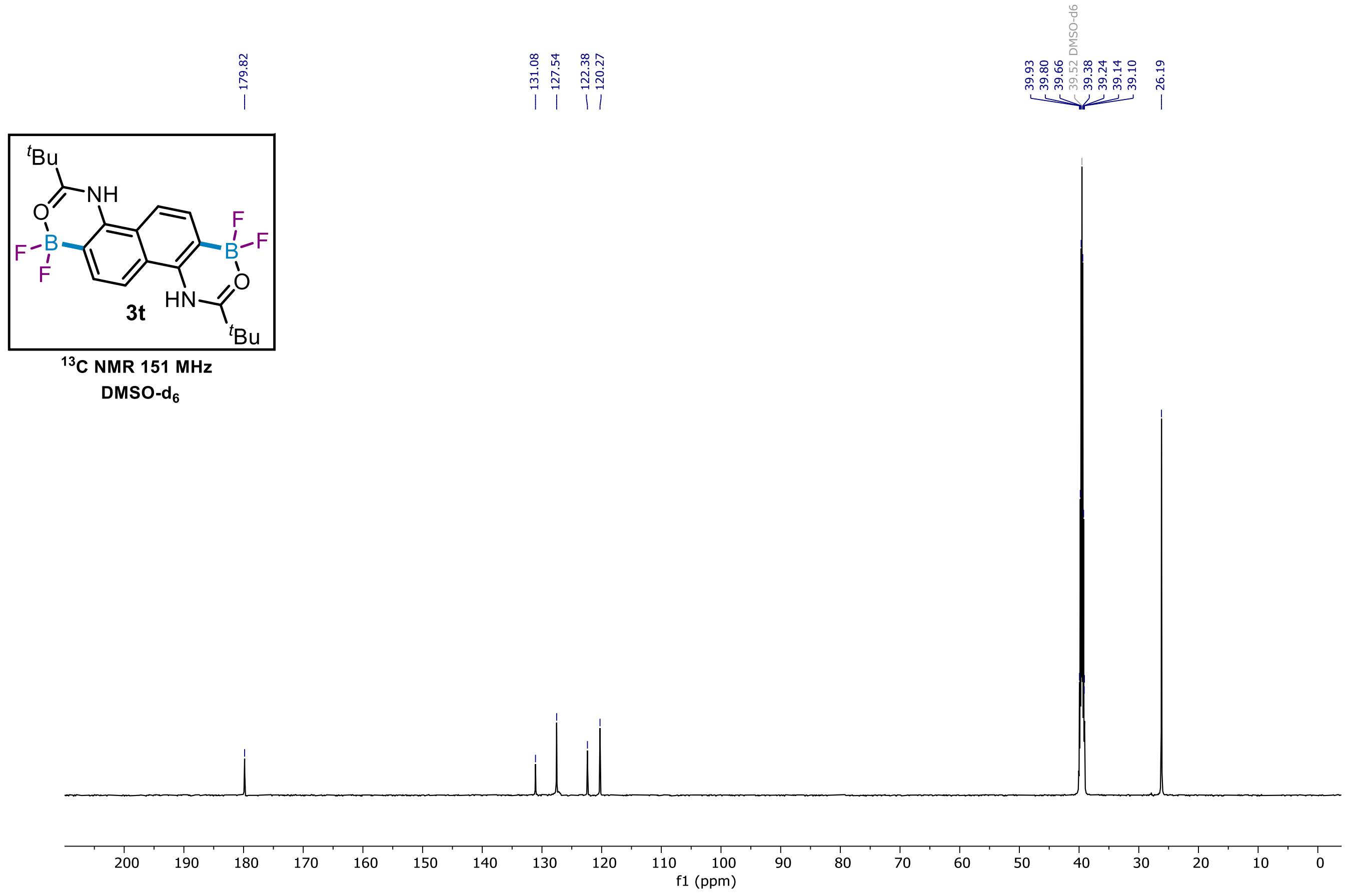

Figure S11-20: ^{13}C spectrum of compound **3t** in DMSO-d$_6$. Note that the ^{13}C signal for the C-BF_2 bond does not appear.

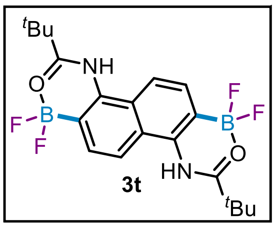

^{19}F NMR 659 MHz
DMSO-d$_6$

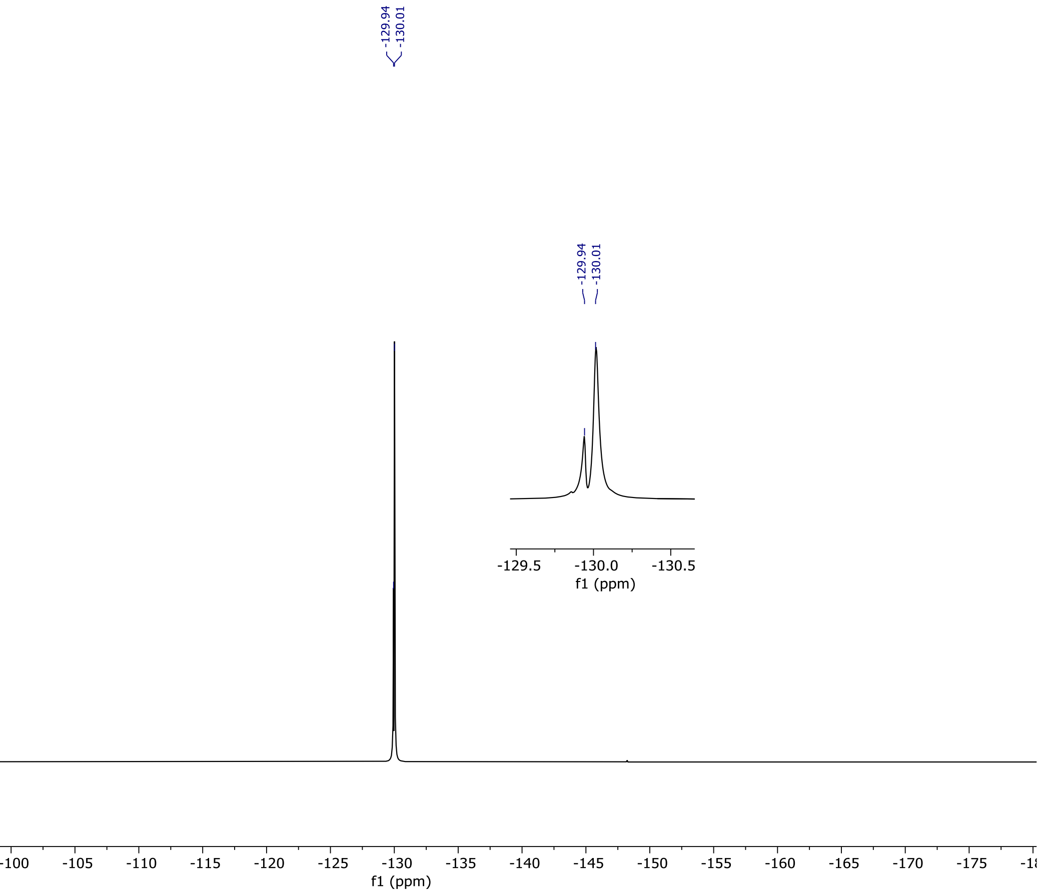

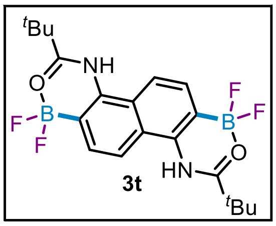

^{11}B NMR 193 MHz
DMSO-d$_6$

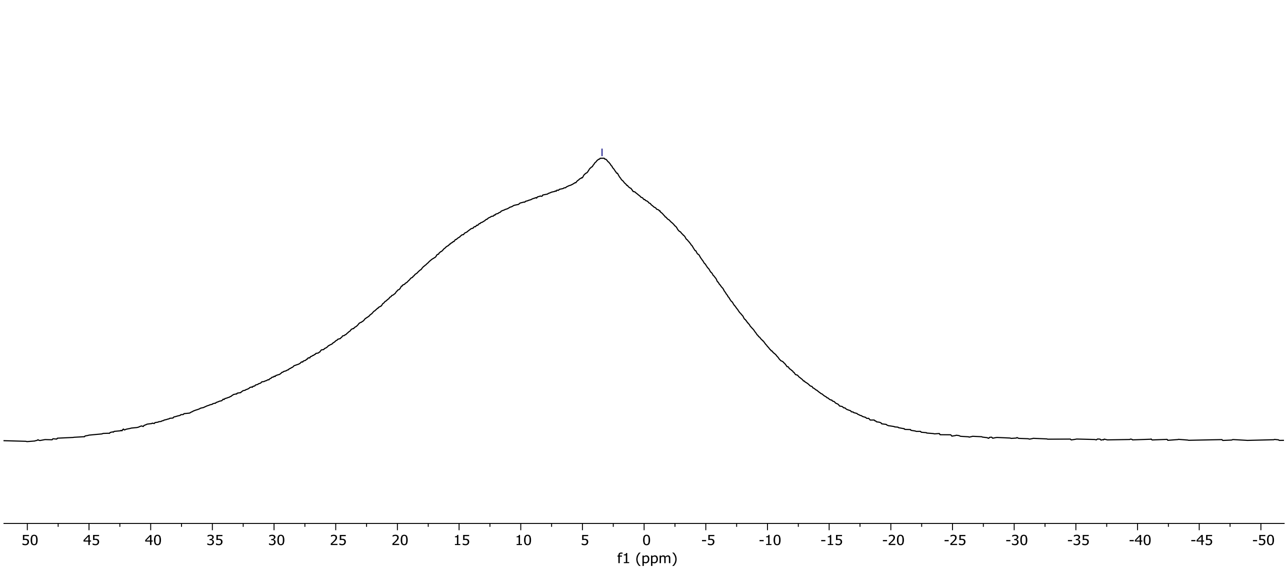

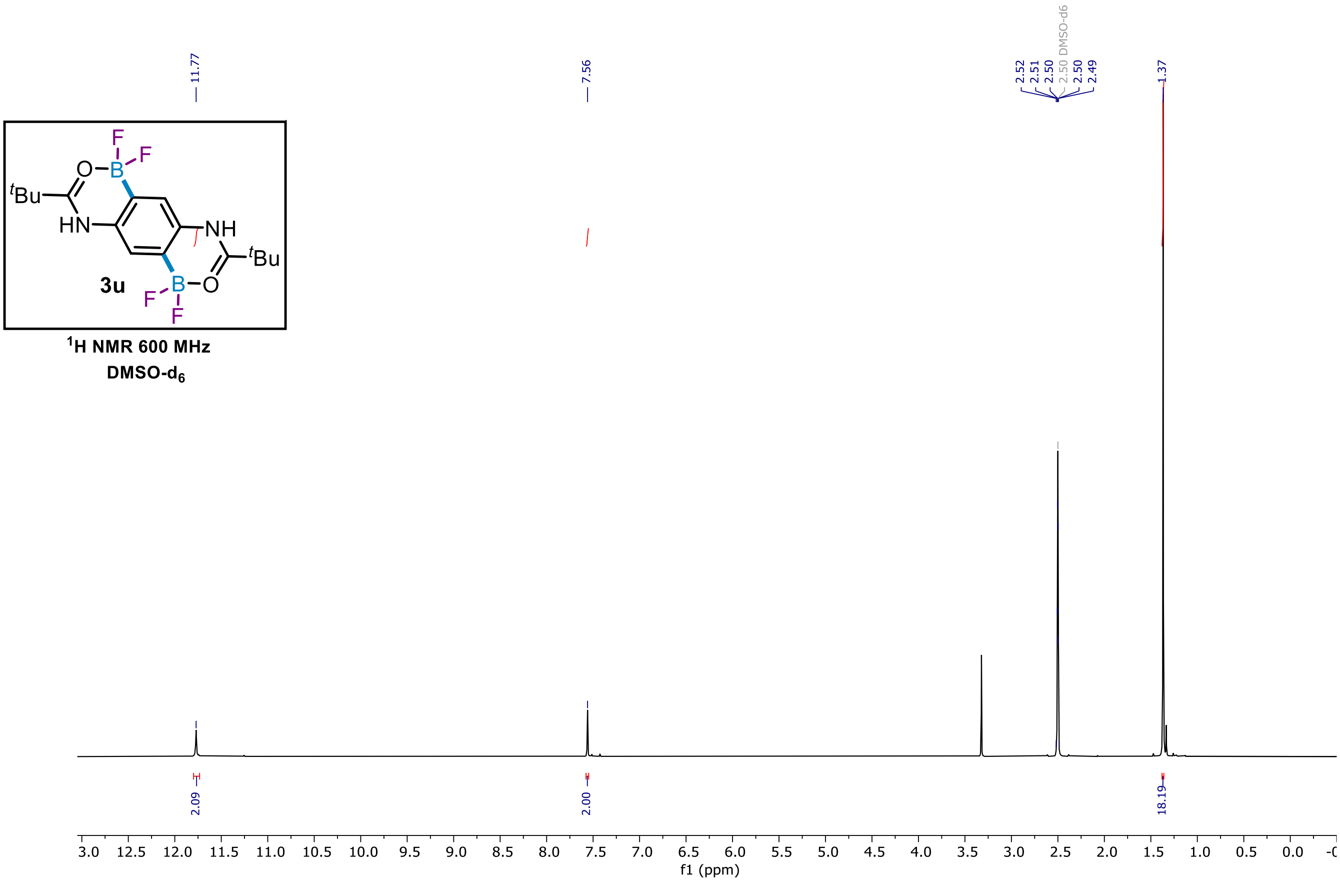

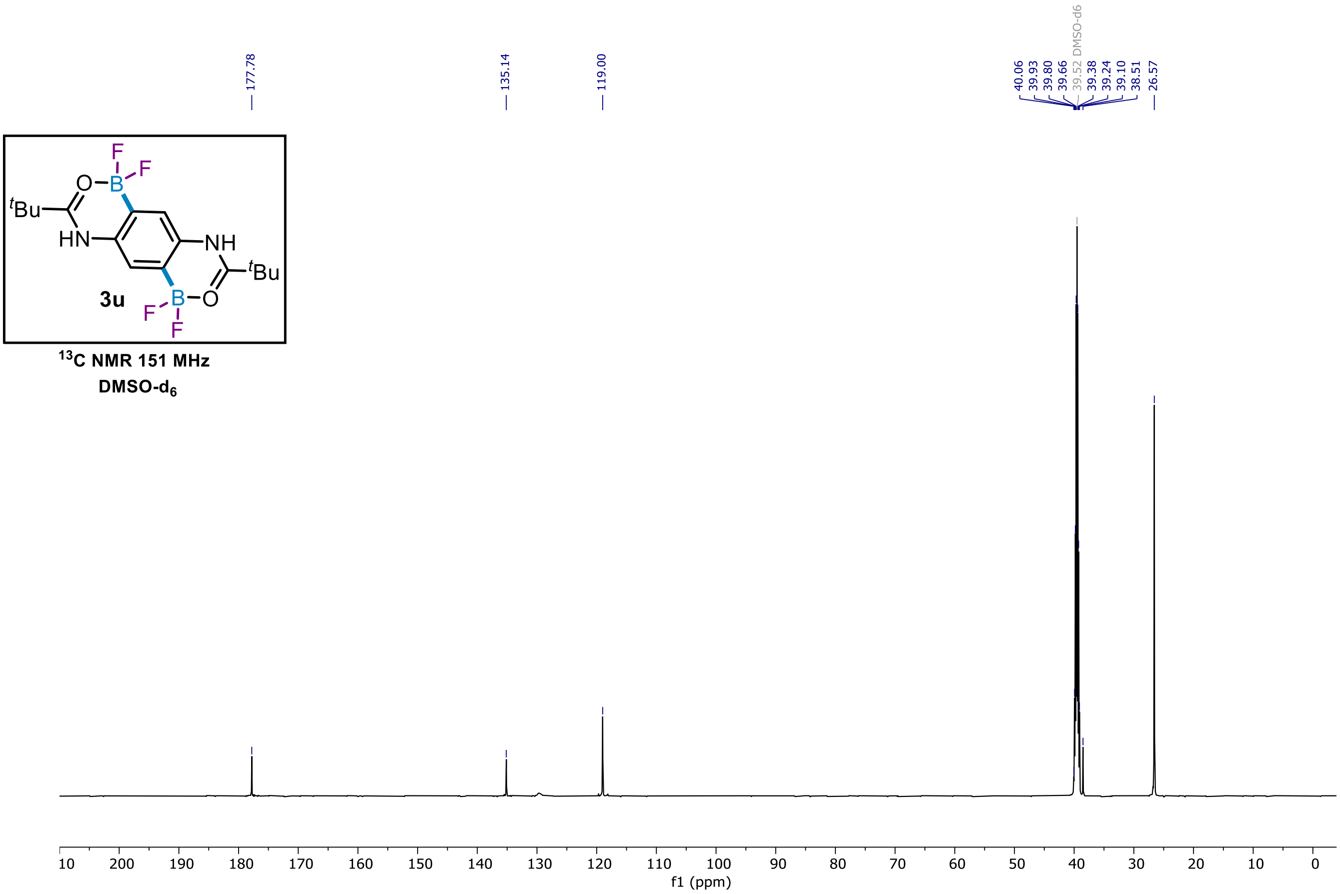

Figure S11-21: ^{13}C spectrum of compound **3u** in DMSO-d_6. Note that the ^{13}C signal for the C-BF_2 bond does not appear.

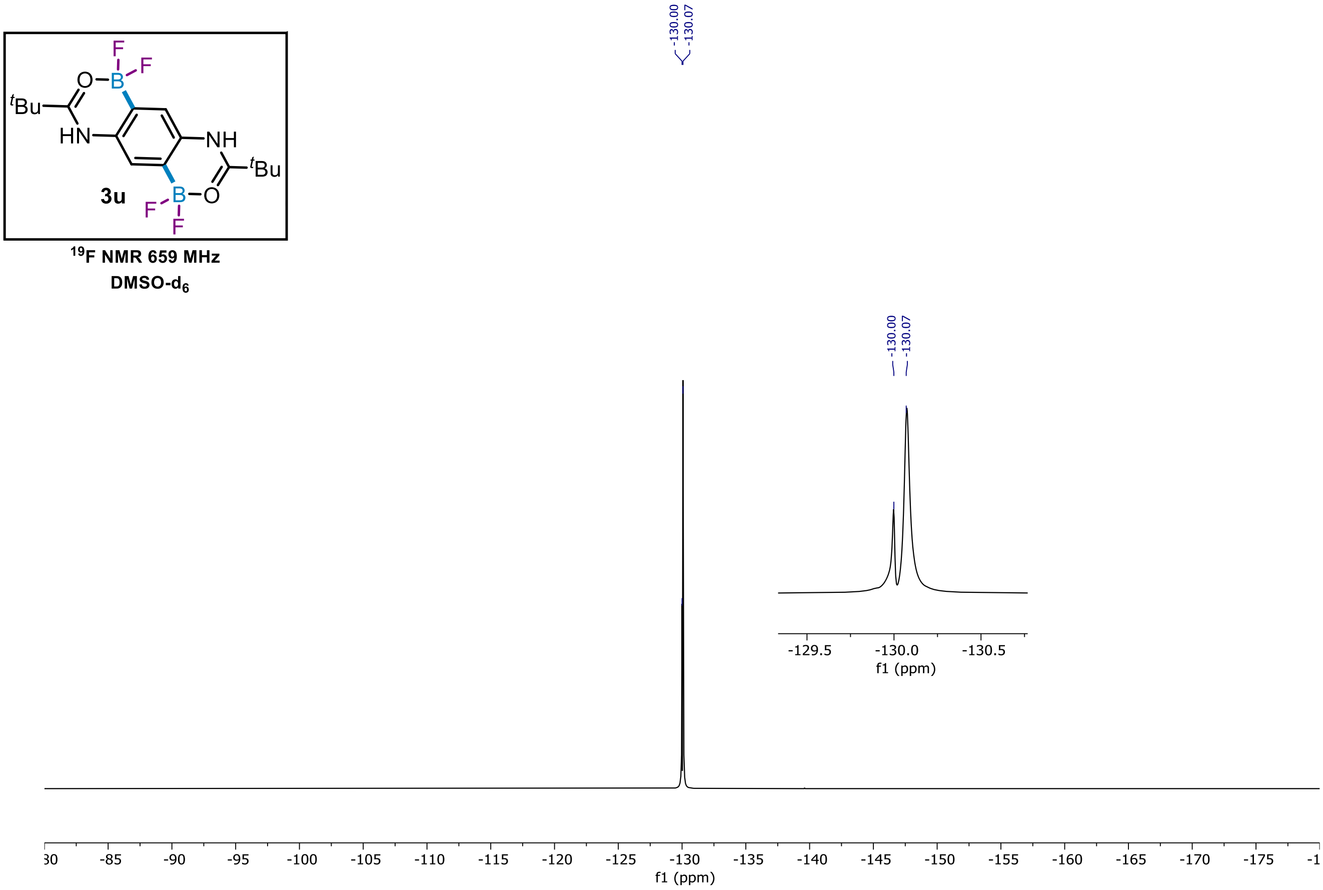

^{19}F NMR 659 MHz
DMSO-d$_6$

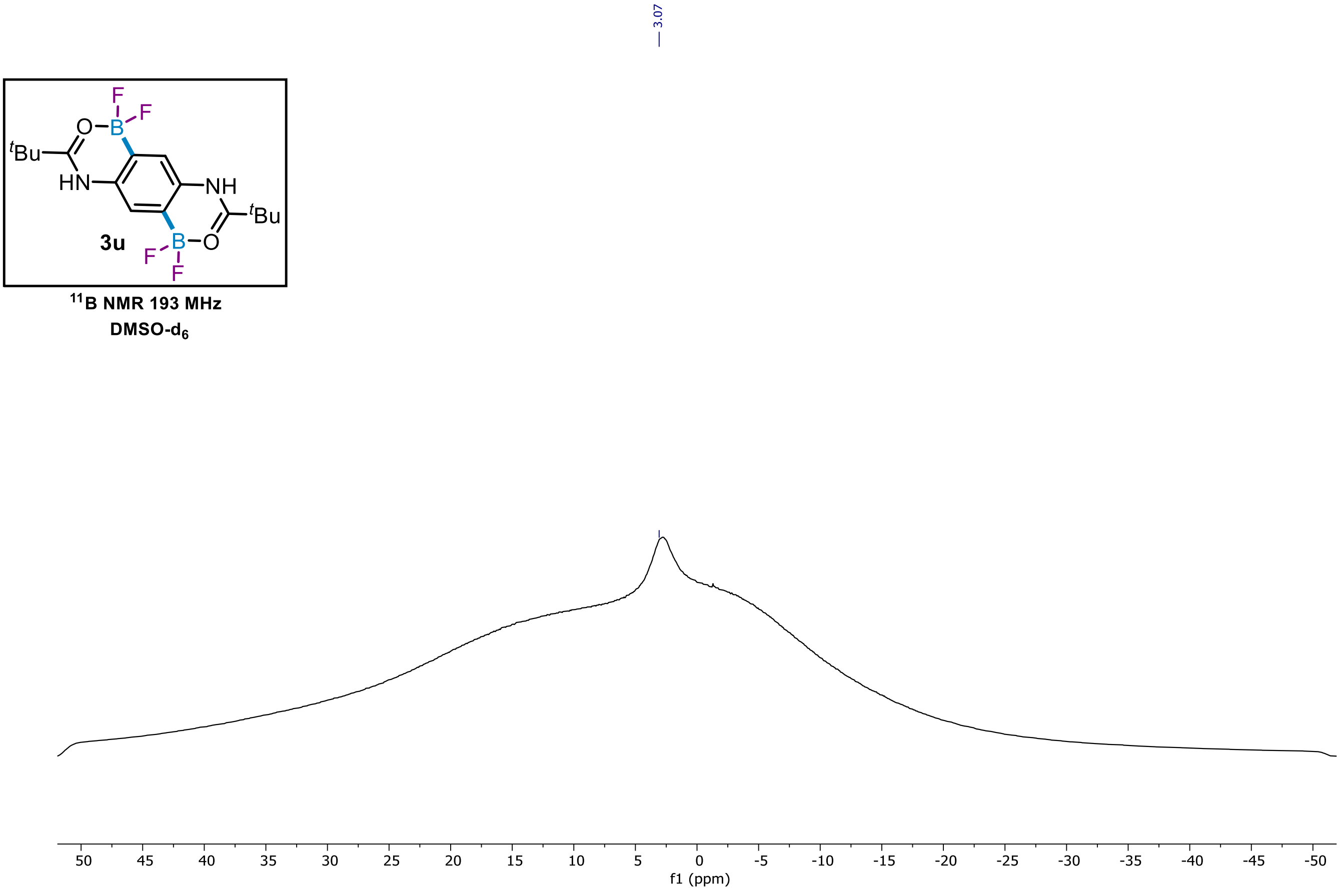

^{11}B NMR 193 MHz
DMSO-d$_6$

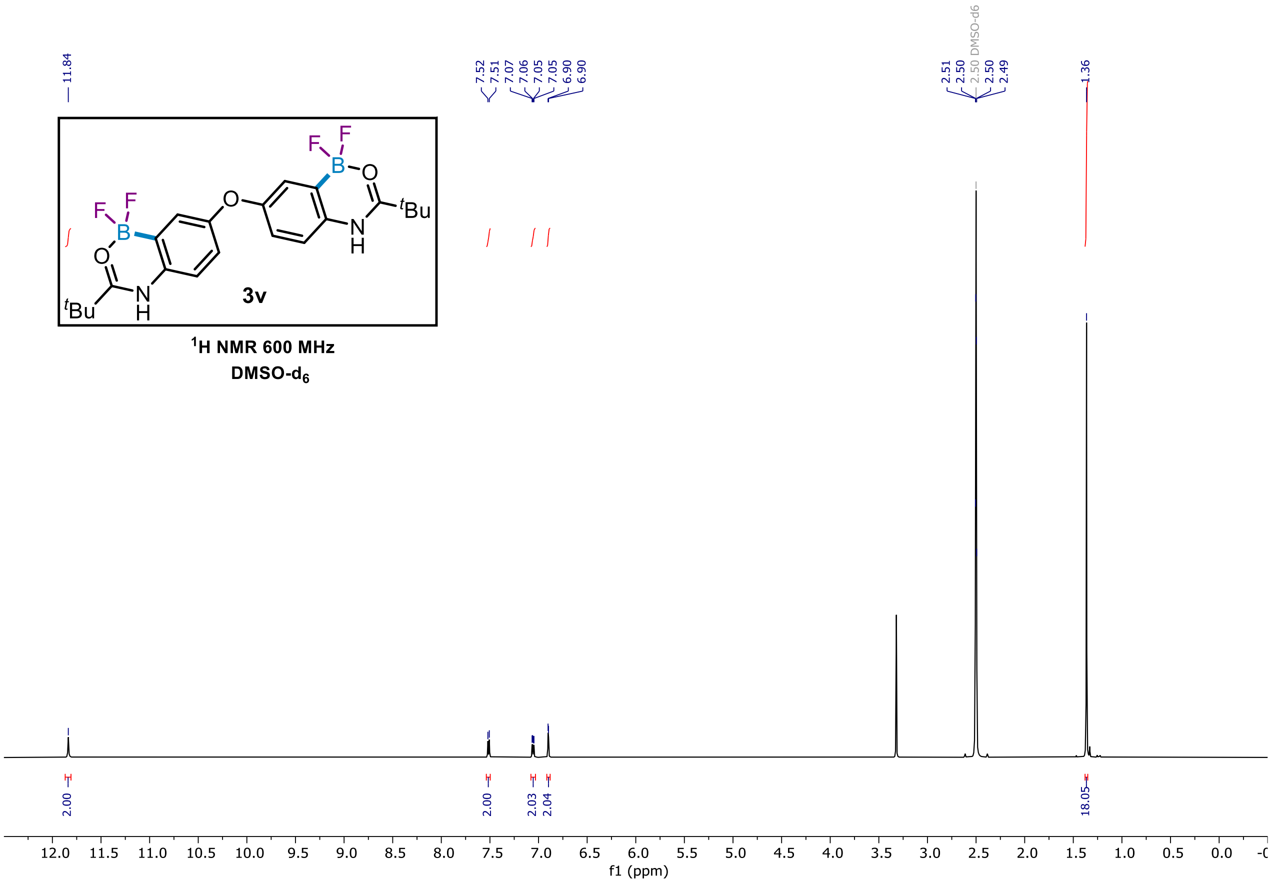

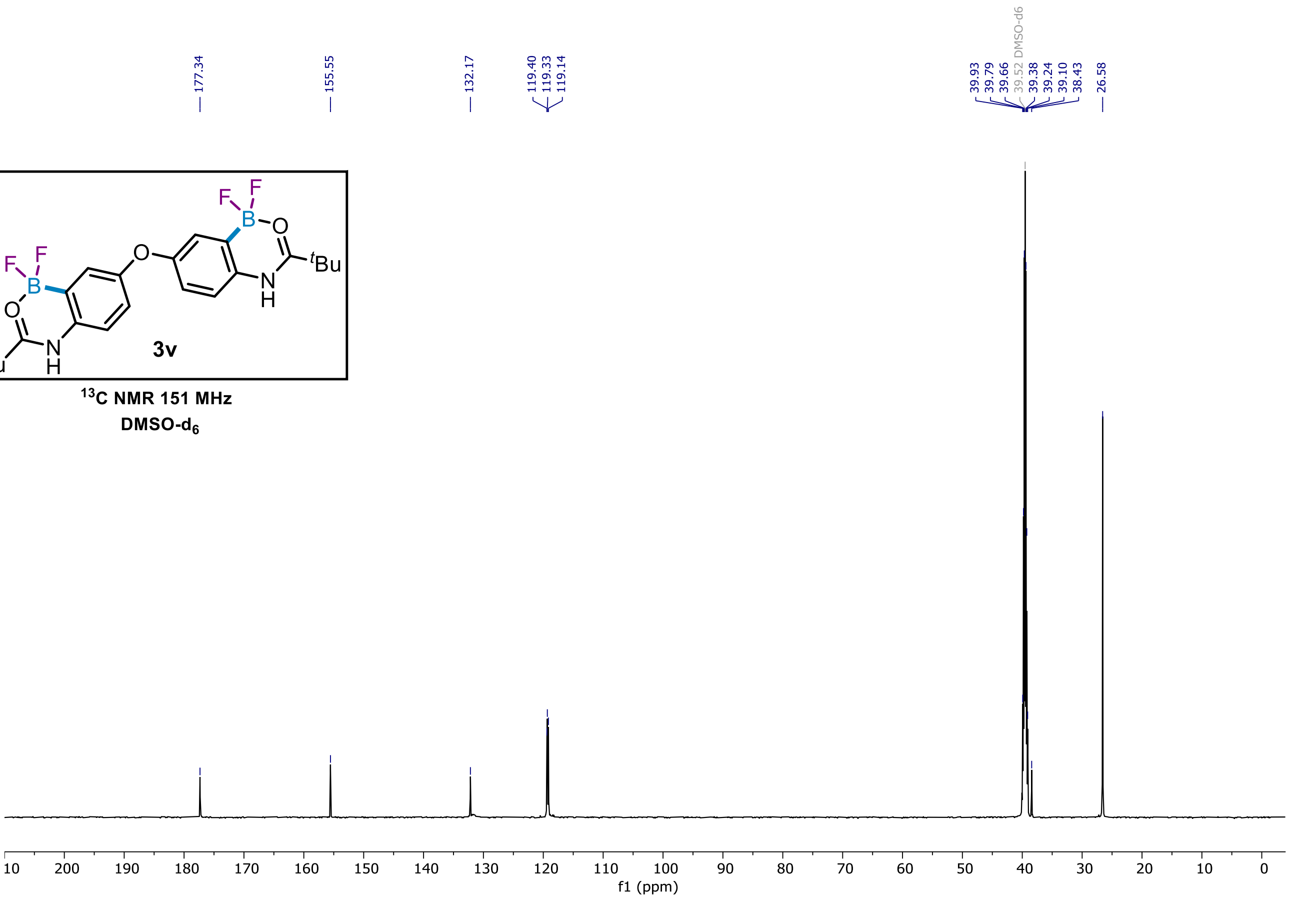

Figure S11-22: ^{13}C spectrum of compound **3v** in DMSO-d$_6$. Note that the ^{13}C signal for the C-BF$_2$ bond does not appear.

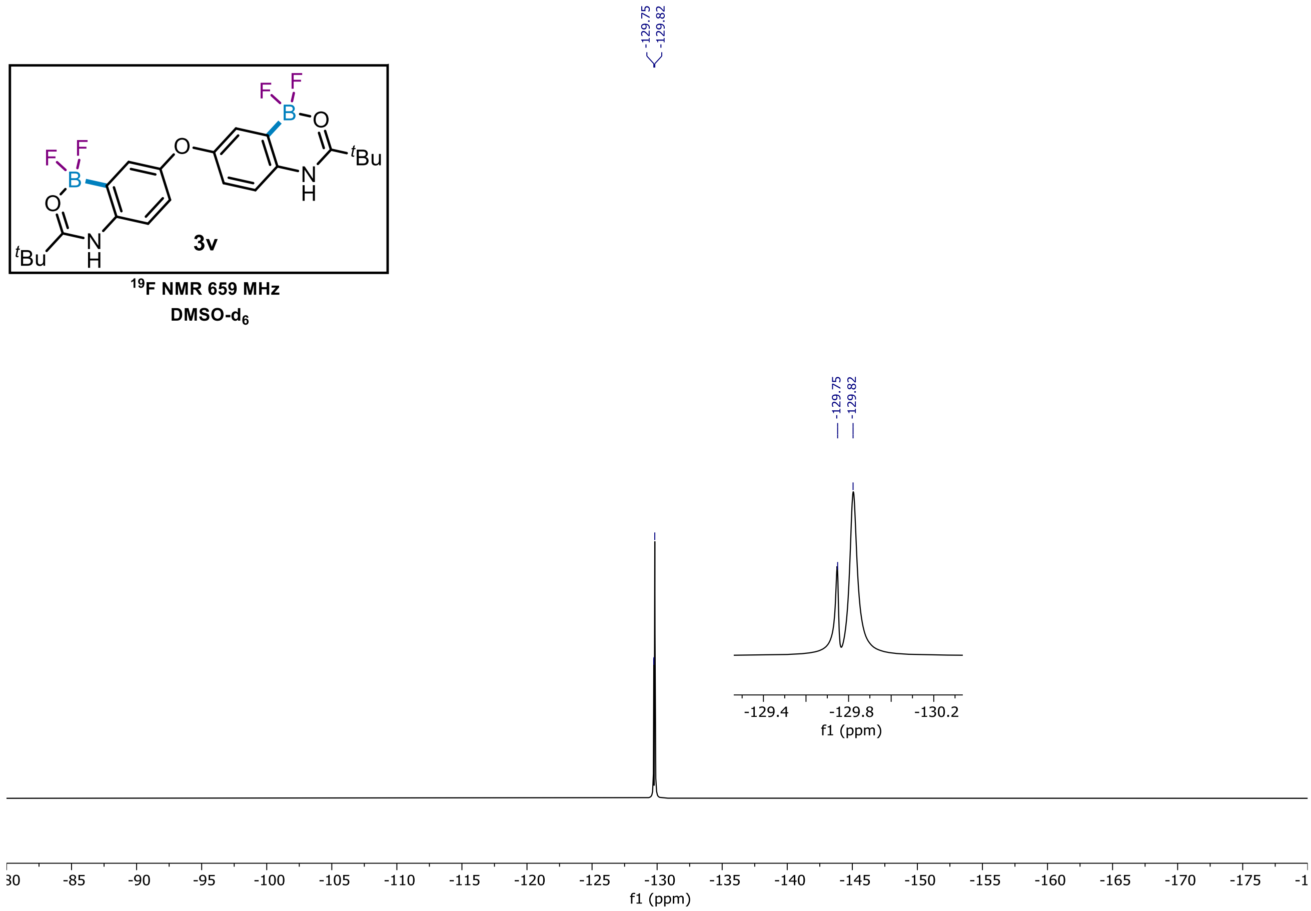
-129.75
-129.82
F
F
B
O
O
O
tBu
N
H
F
F
B
O
tBu
N
H
3v
19F NMR 659 MHz
DMSO-d6
-129.4
-129.8
-130.2
f1 (ppm)
f1 (ppm)

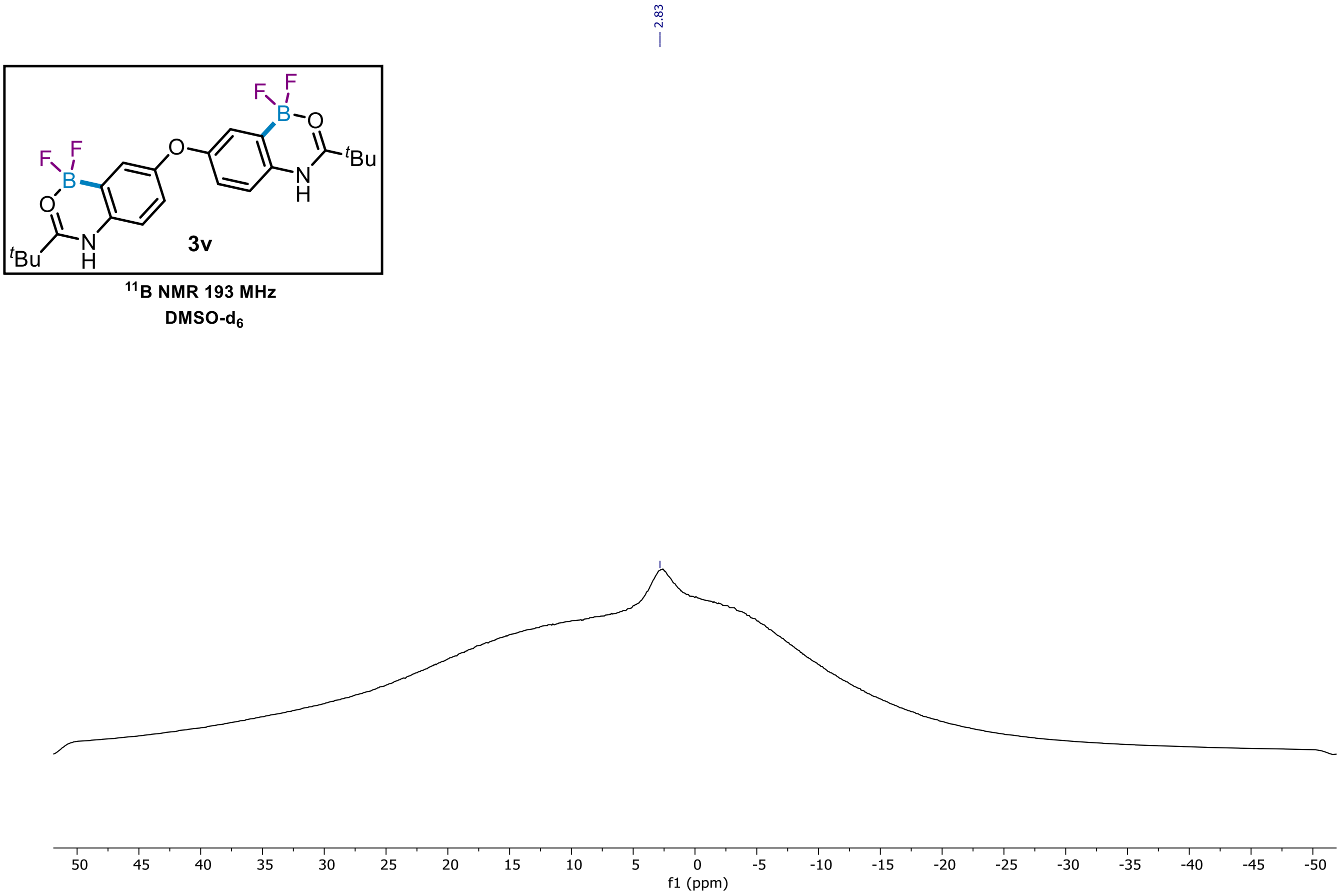
2.83
F
F
B
O
O
O
tBu
N
H
F
F
B
O
tBu
N
H
3v
11B NMR 193 MHz
DMSO-d6
f1 (ppm)

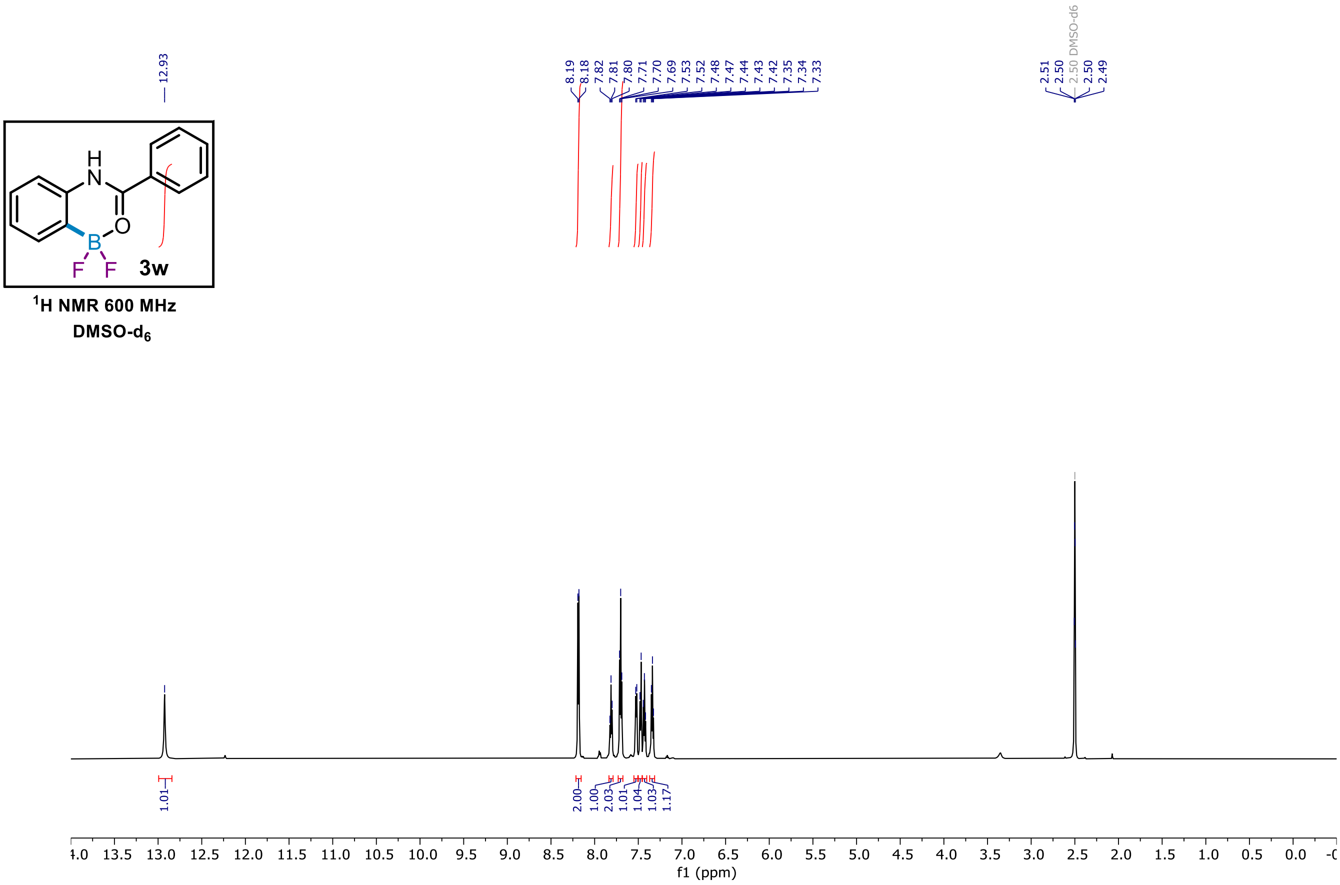

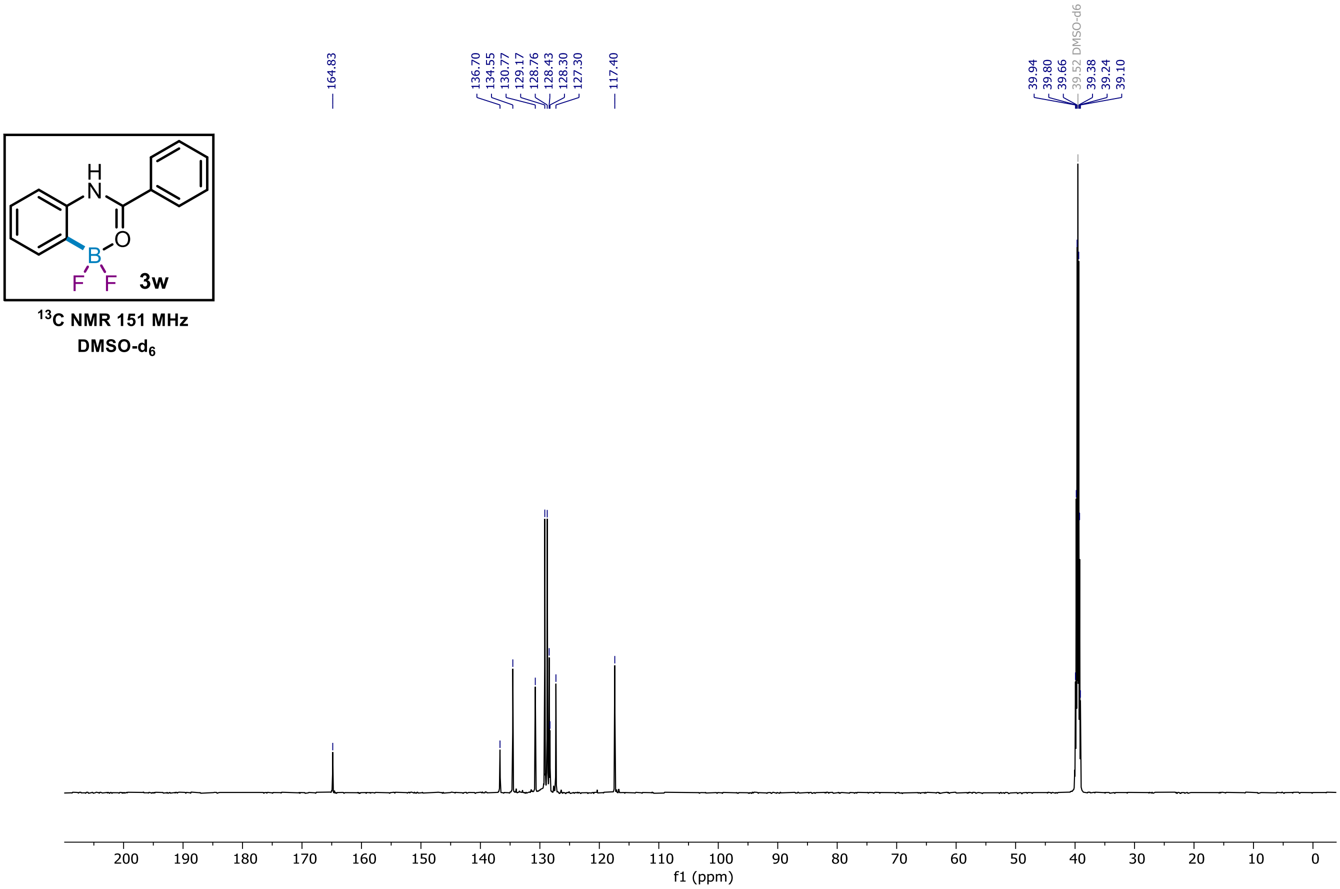

Figure S11-23: ^{13}C spectrum of compound **3w** in DMSO-d_6. Note that the ^{13}C signal for the C-BF_2 bond does not appear.

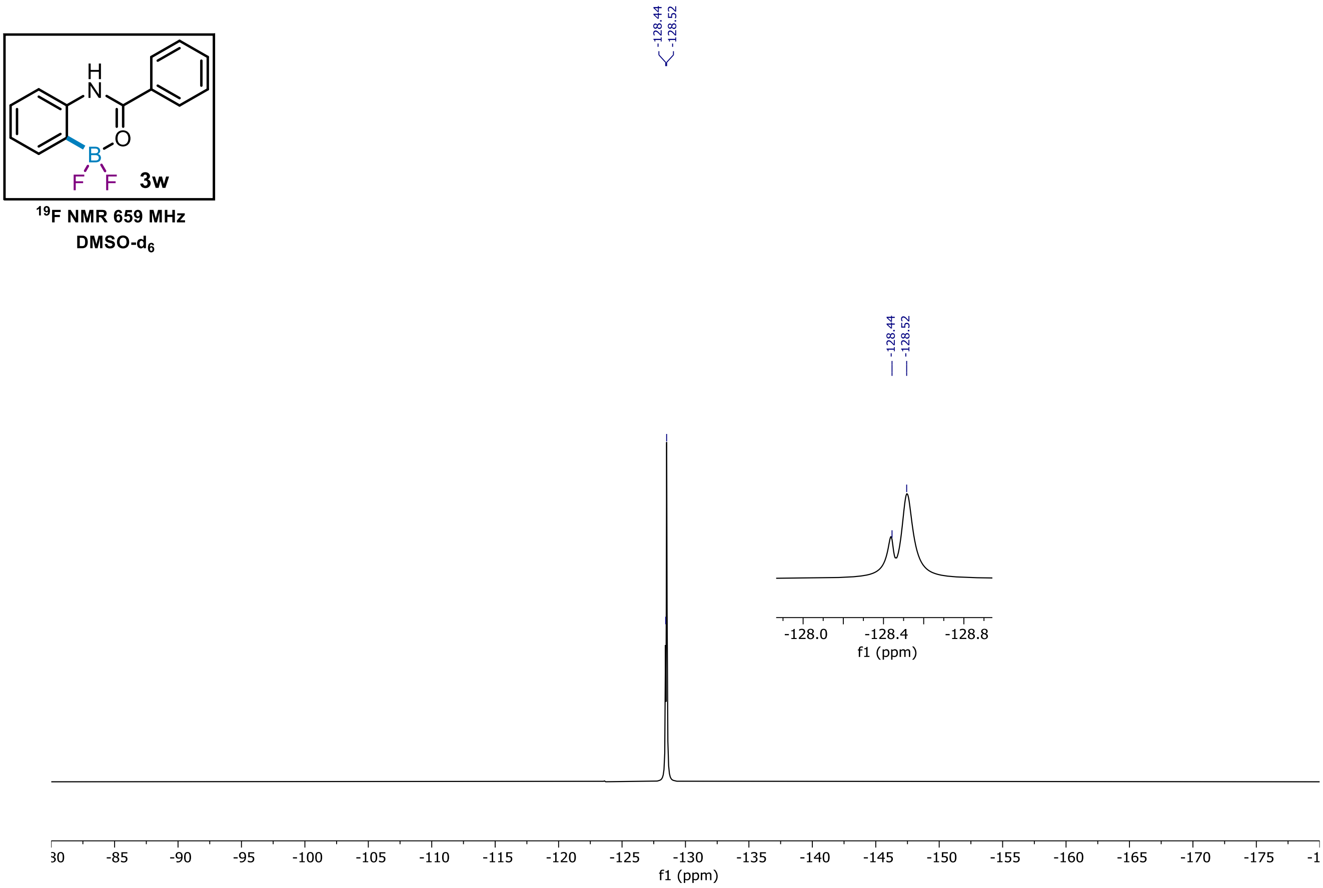
3w
19F NMR 659 MHz
DMSO-d6
-128.44
-128.52
f1 (ppm)

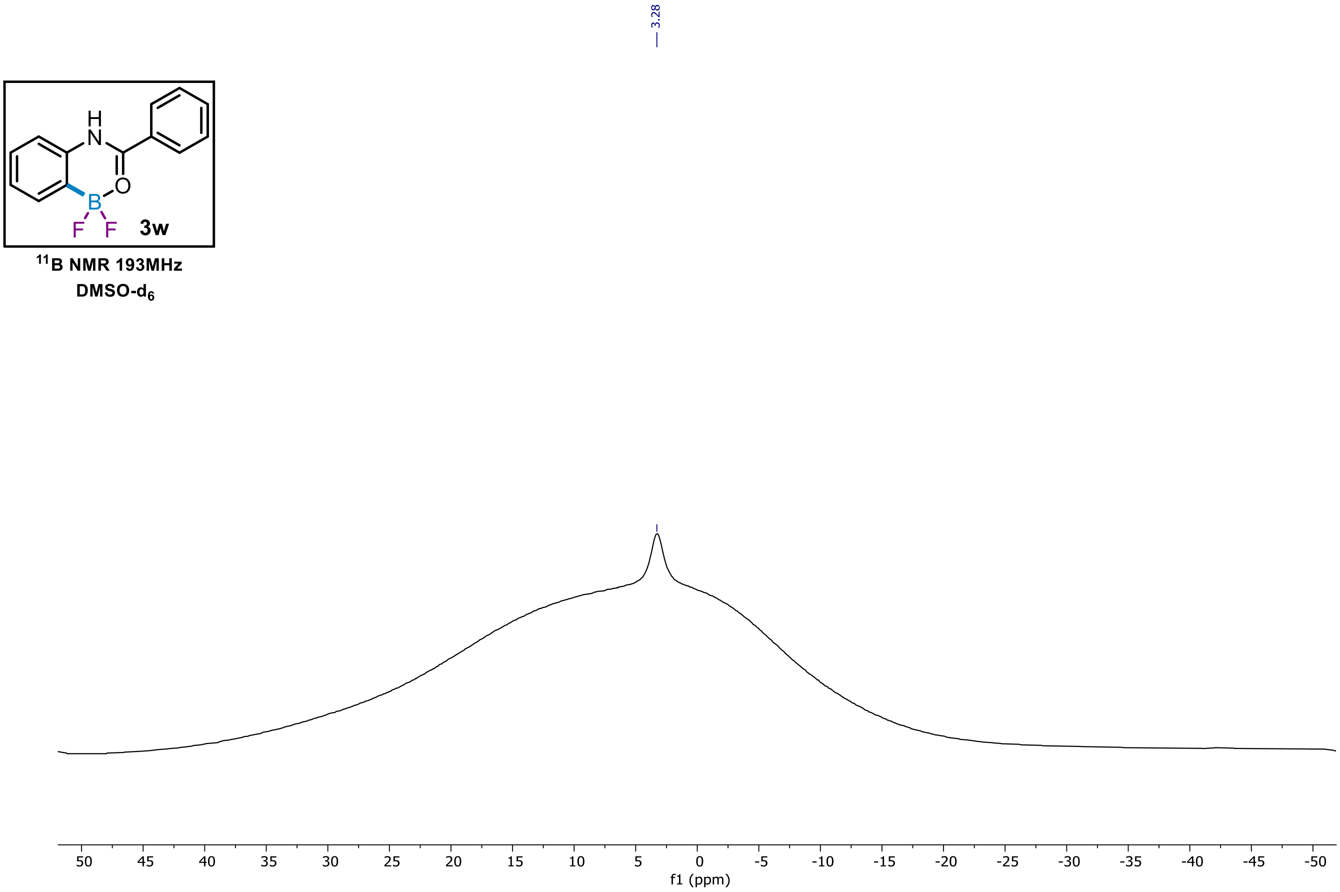
3w
11B NMR 193MHz
DMSO-d6
3.28
f1 (ppm)

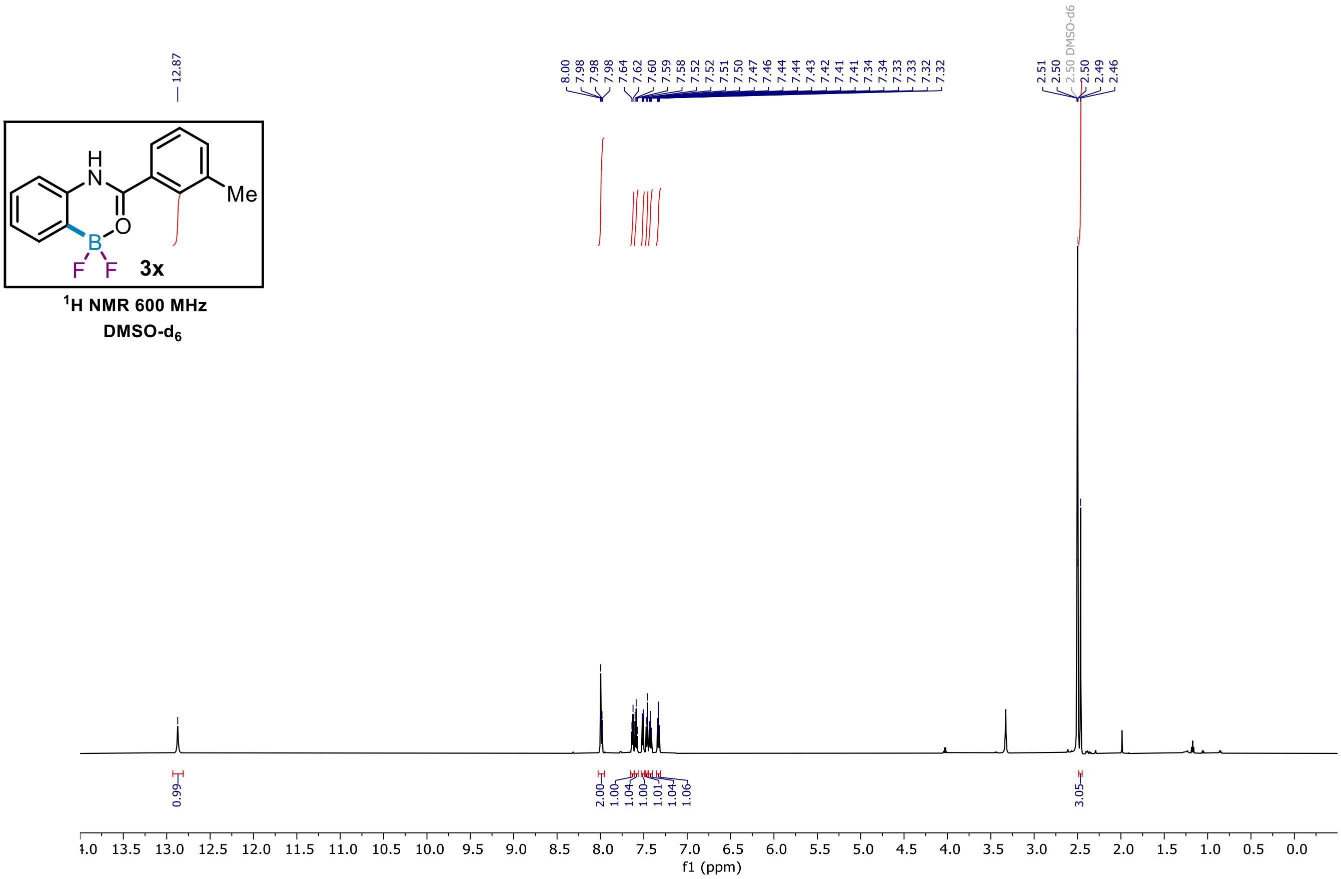

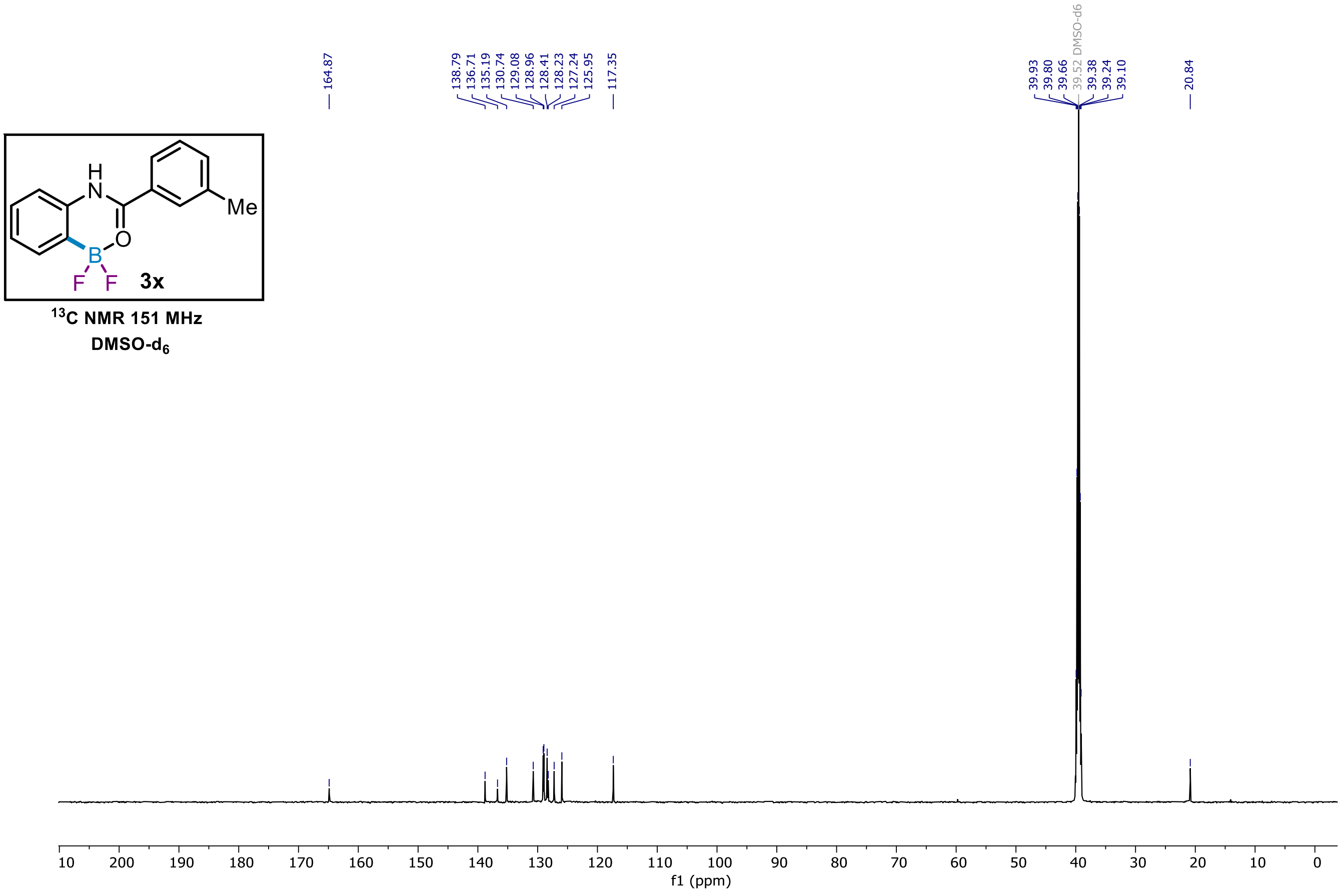

Figure S11-24: ^{13}C spectrum of compound **3x** in DMSO-d_6. Note that the ^{13}C signal for the C-BF_2 bond does not appear.

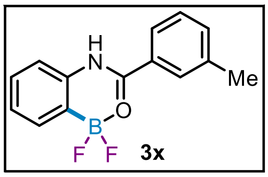

^{19}F NMR 659 MHz
DMSO-d$_6$

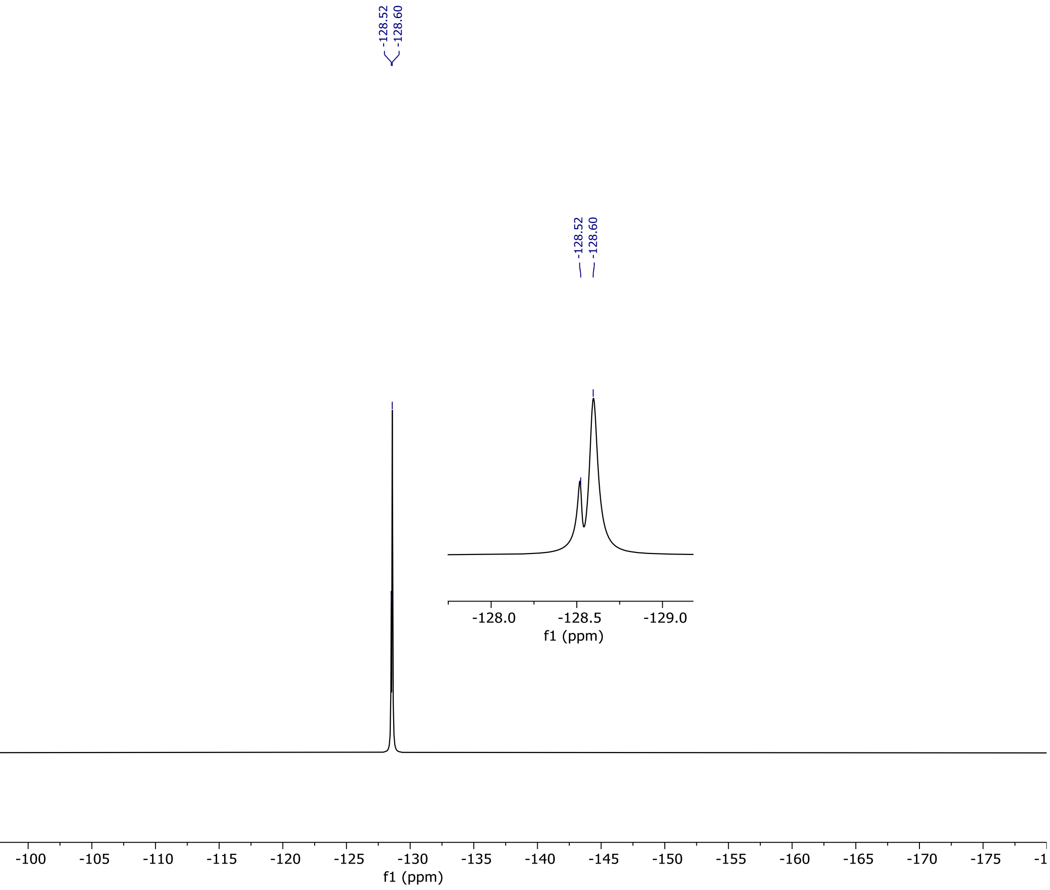

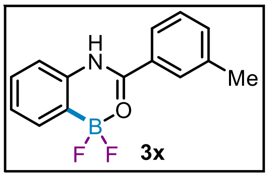

^{11}B NMR 193 MHz
DMSO-d$_6$

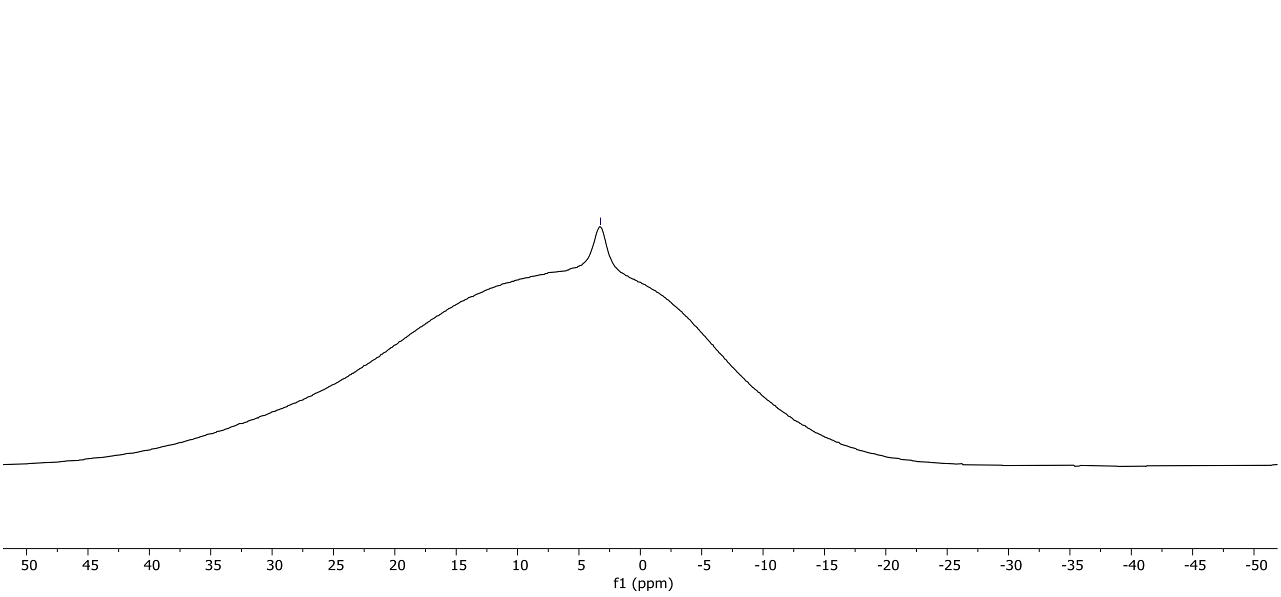

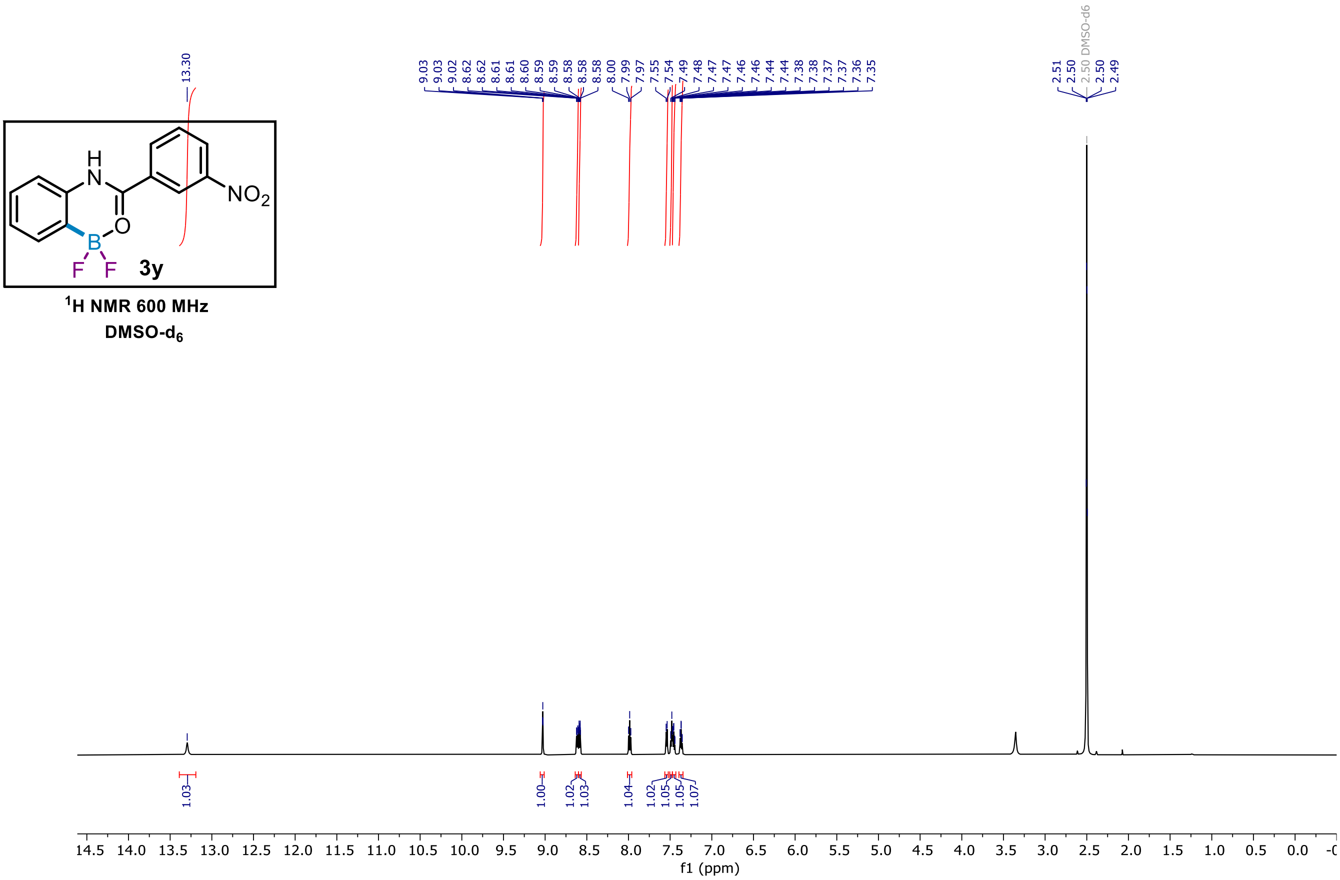

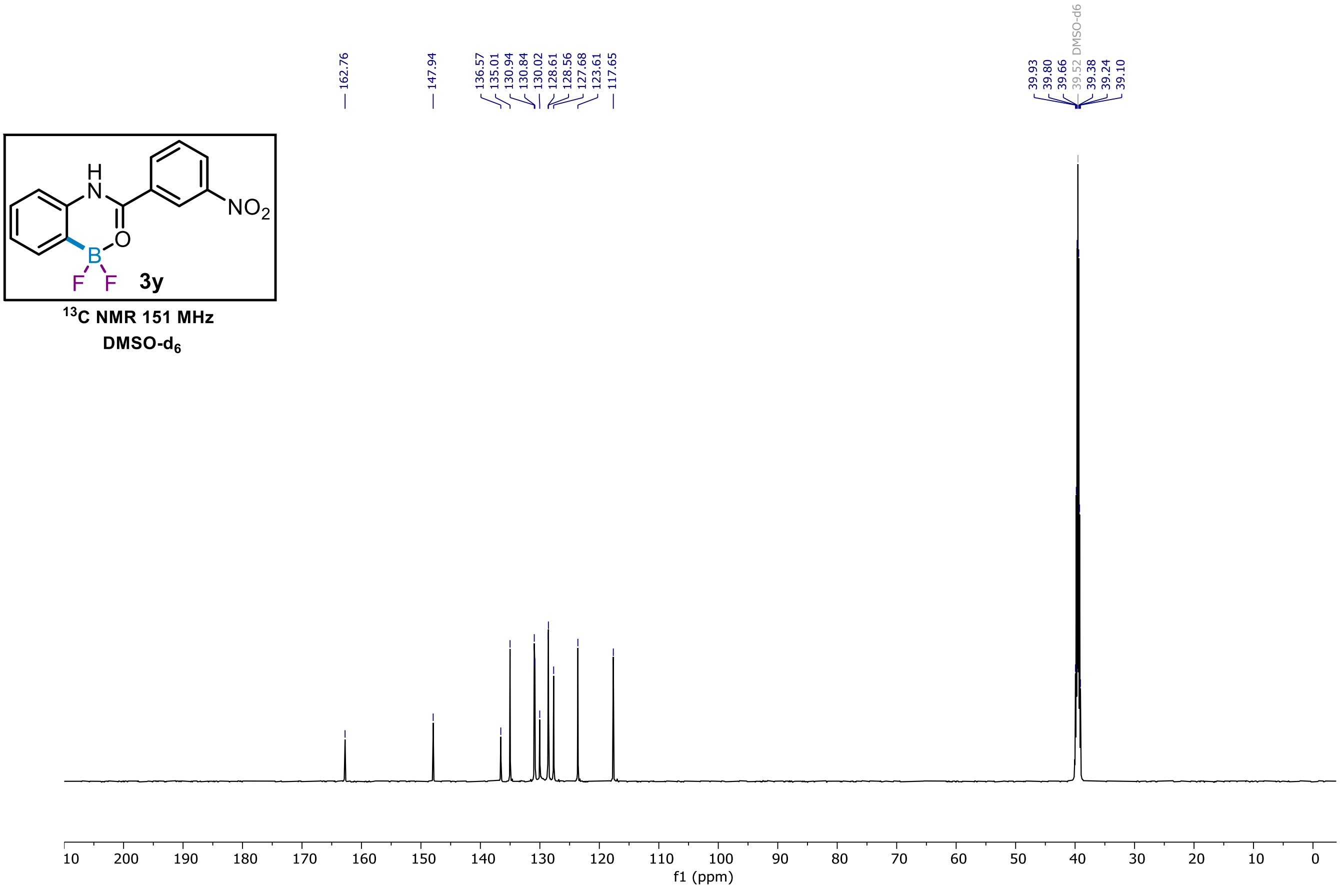

Figure S11-25: ^{13}C spectrum of compound **3y** in DMSO-d_6. Note that the ^{13}C signal for the C-BF_2 bond does not appear.

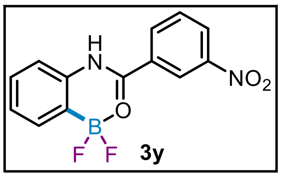

^{19}F NMR 659 MHz
DMSO-d$_6$

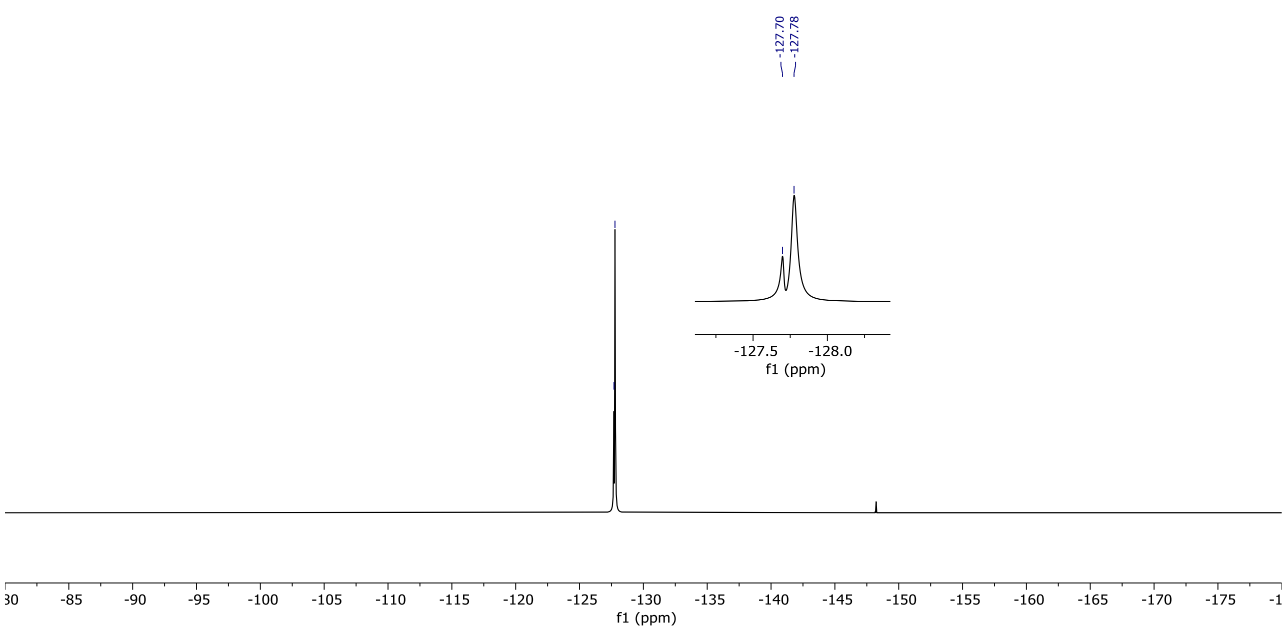

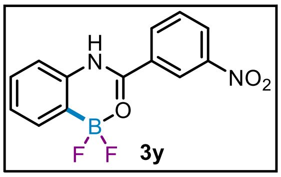

^{11}B NMR 193 MHz
DMSO-d$_6$

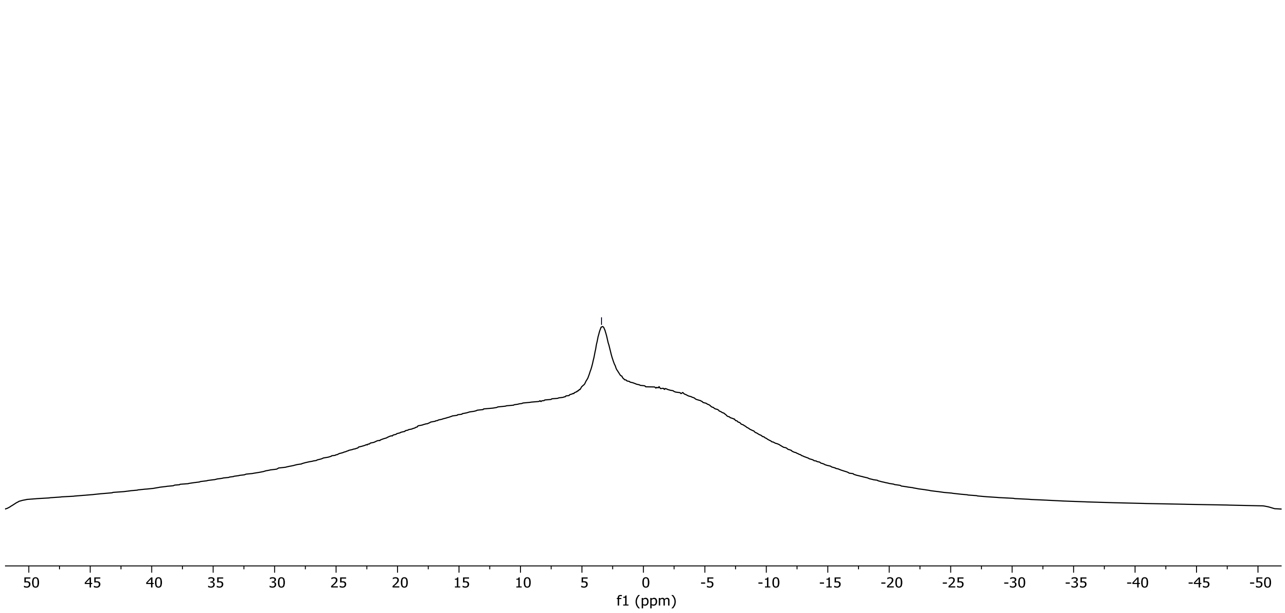

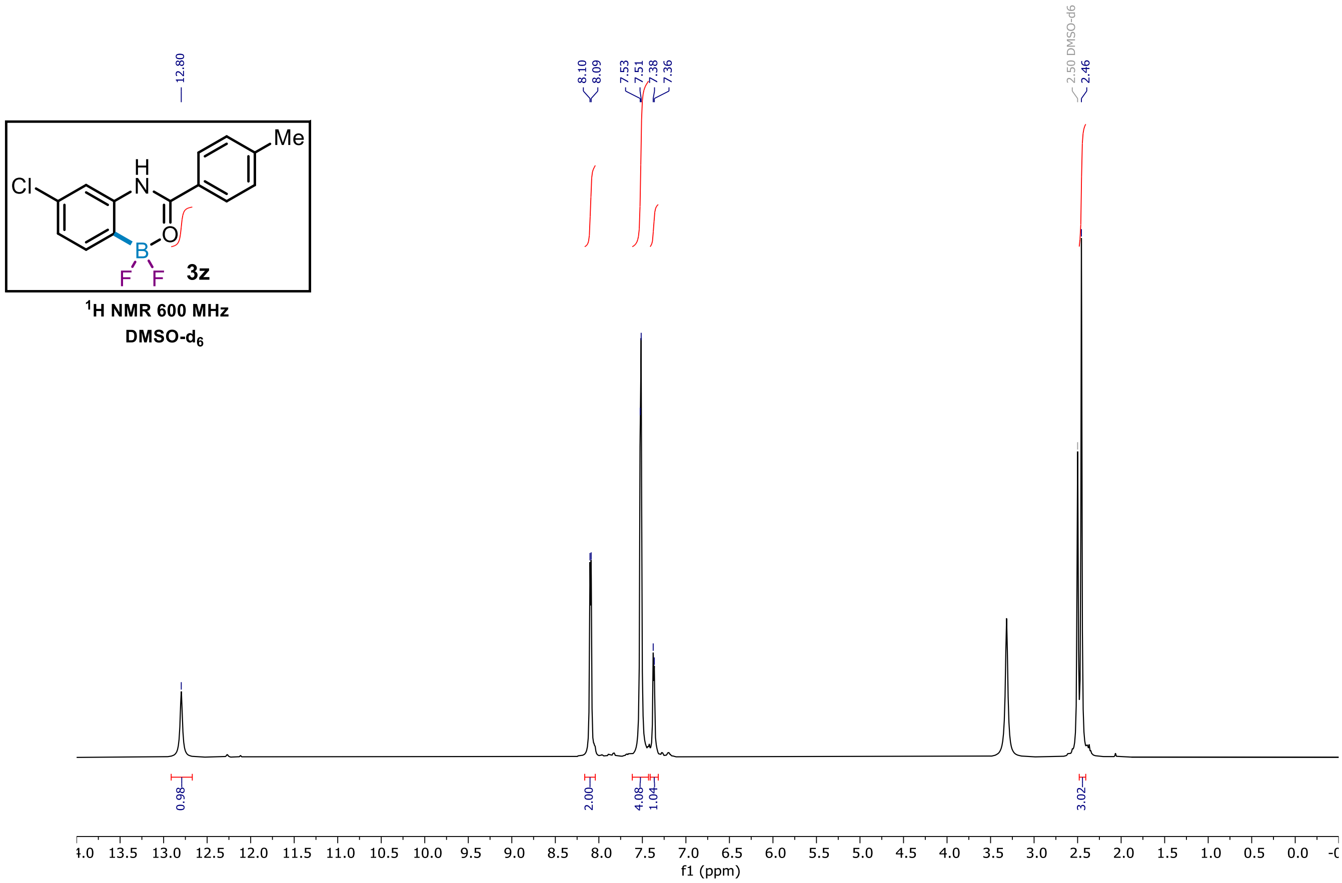

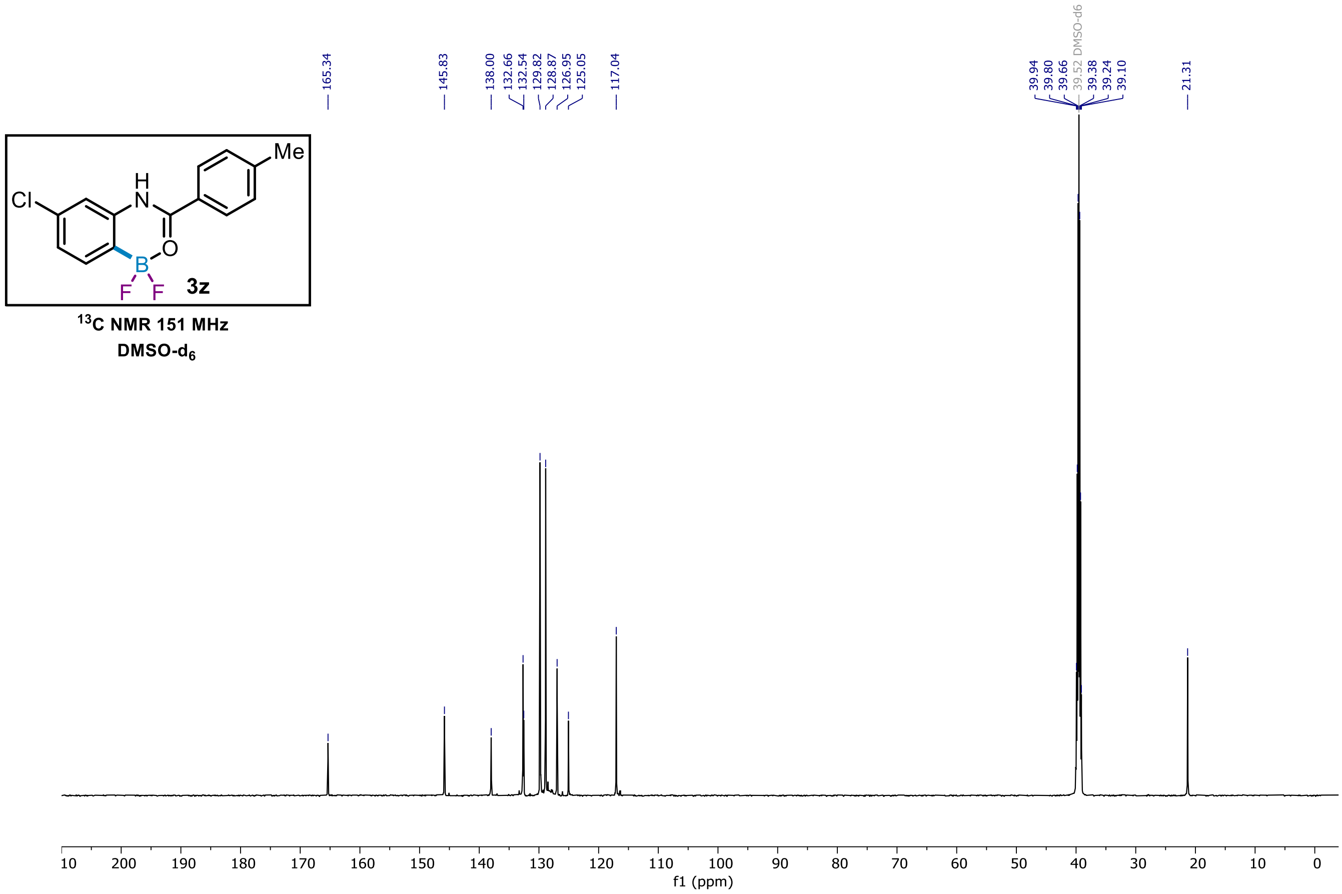

Figure S11-26: ^{13}C spectrum of compound **3z** in DMSO-d_6. Note that the ^{13}C signal for the C-BF_2 bond does not appear.

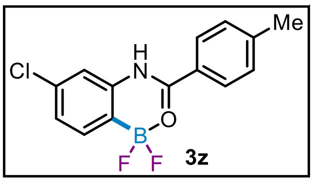

^{19}F NMR 659 MHz
DMSO-d$_6$

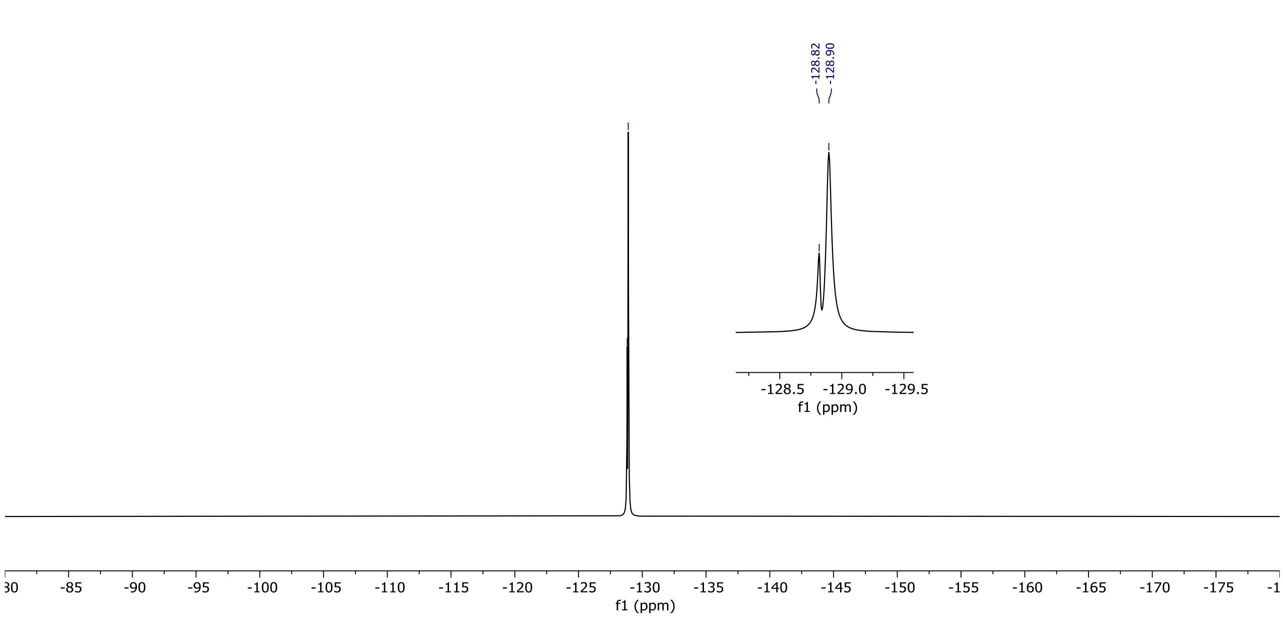

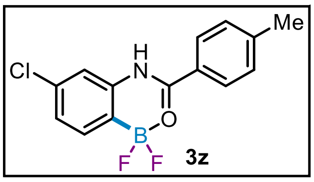

^{11}B NMR 193 MHz
DMSO-d$_6$

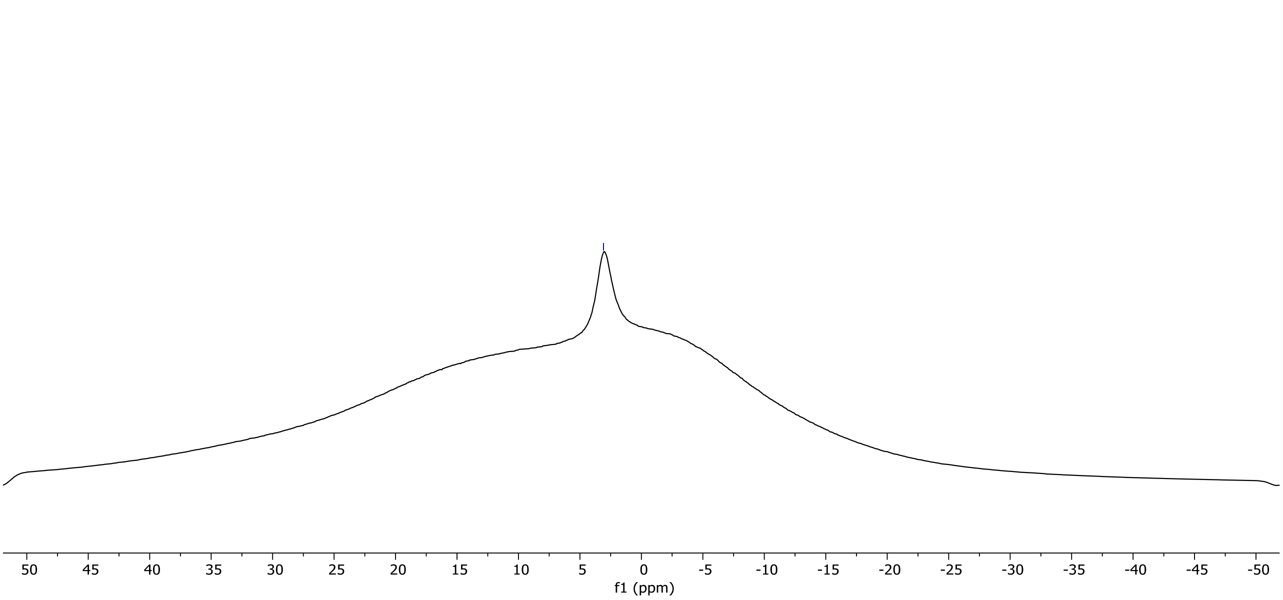

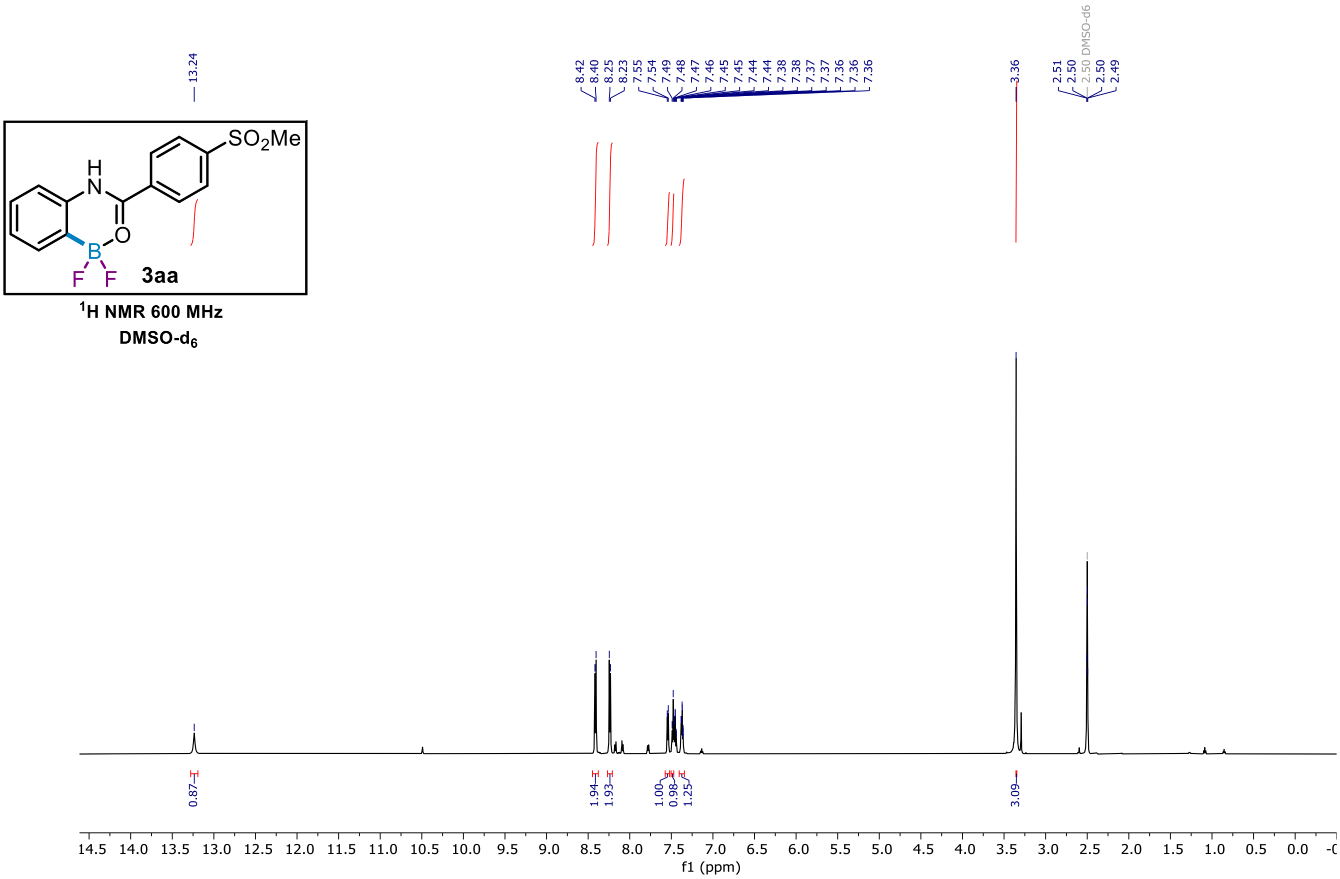

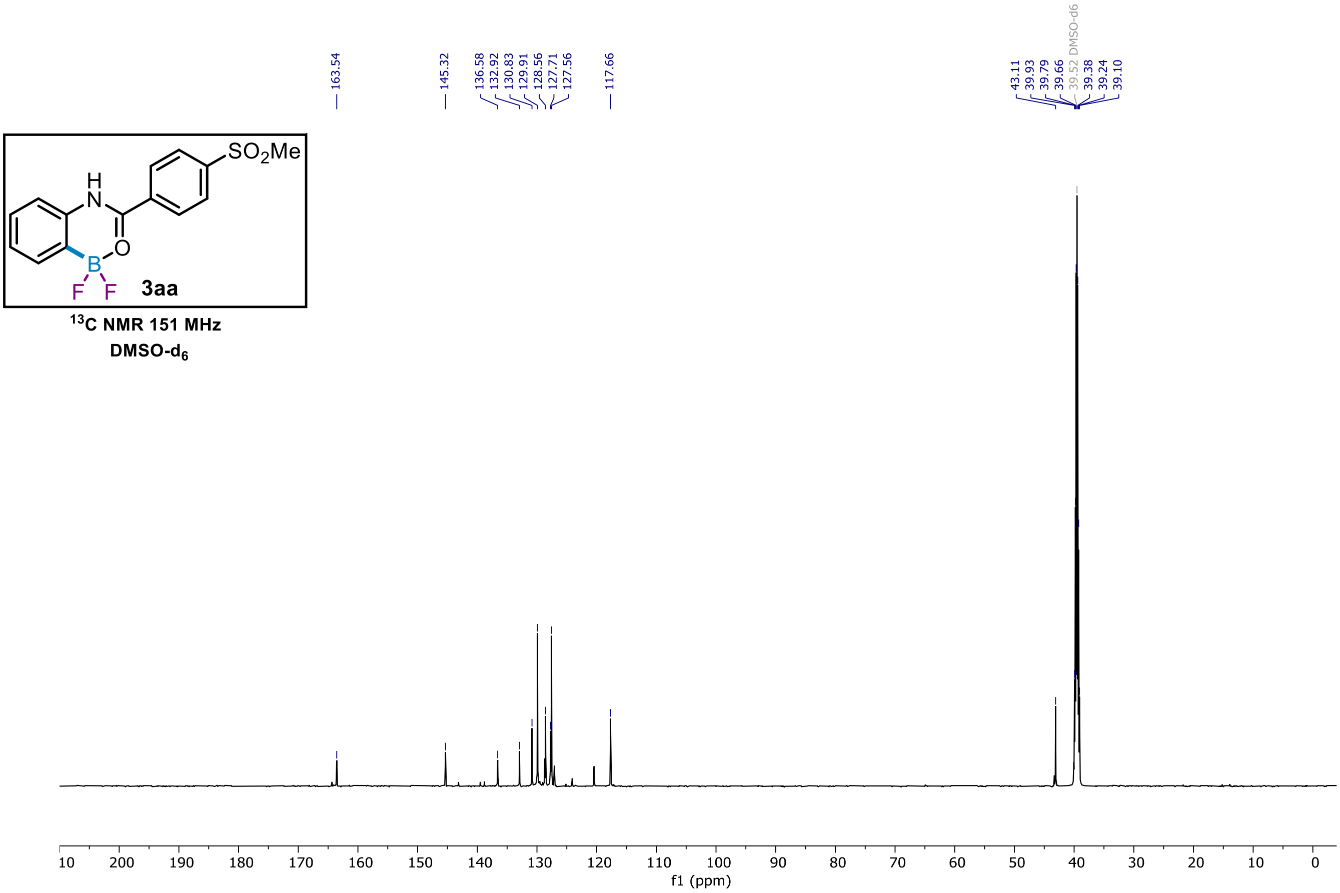

Figure S11-27: ^{13}C spectrum of compound **3aa** in DMSO-d$_6$. Note that the ^{13}C signal for the C-BF$_2$ bond does not appear.

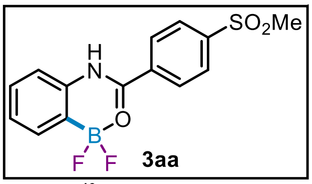

^{19}F NMR 659 MHz
DMSO-d_6

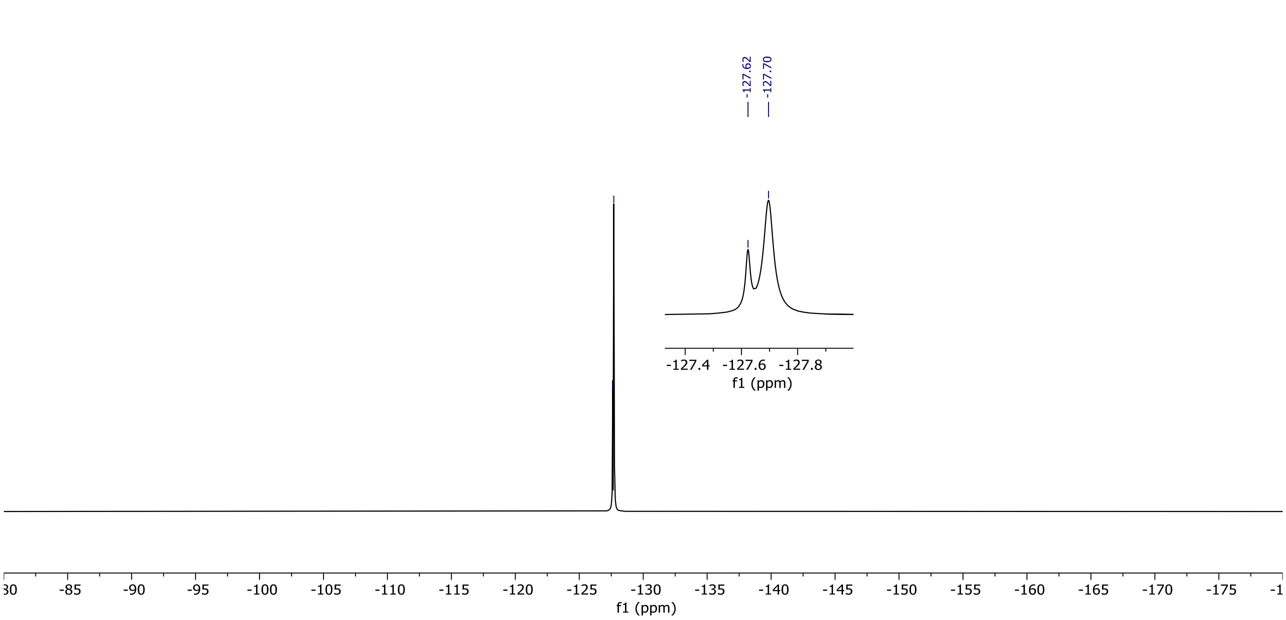

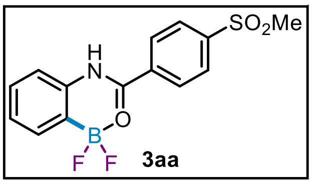

^{11}B NMR 193 MHz
DMSO-d_6

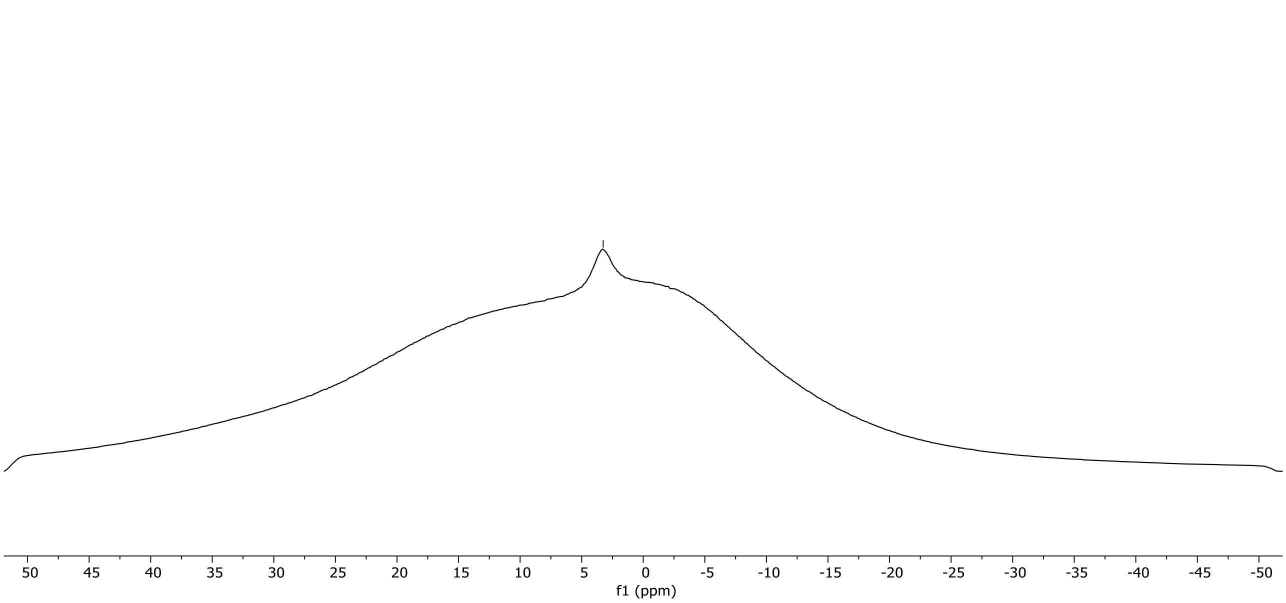

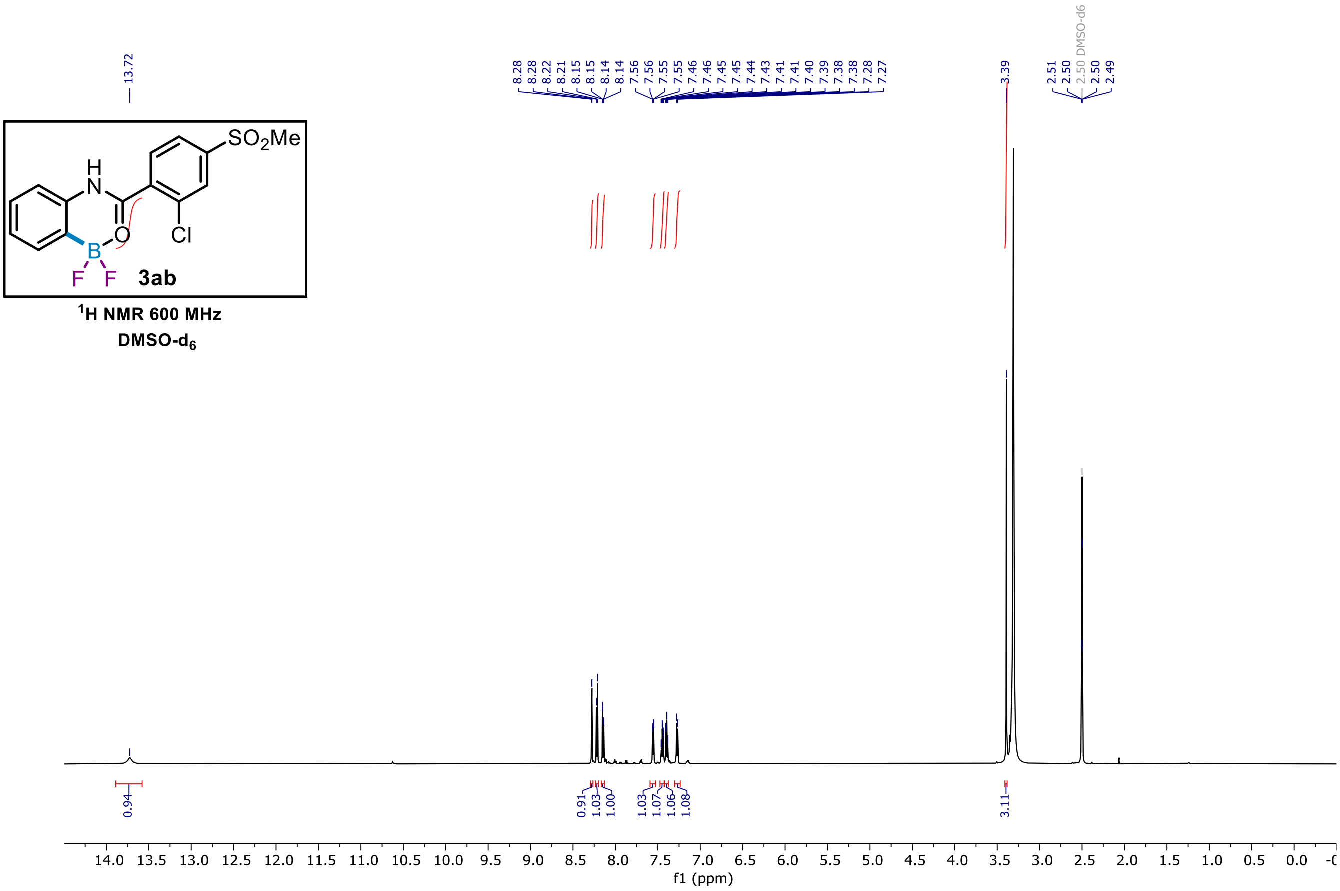

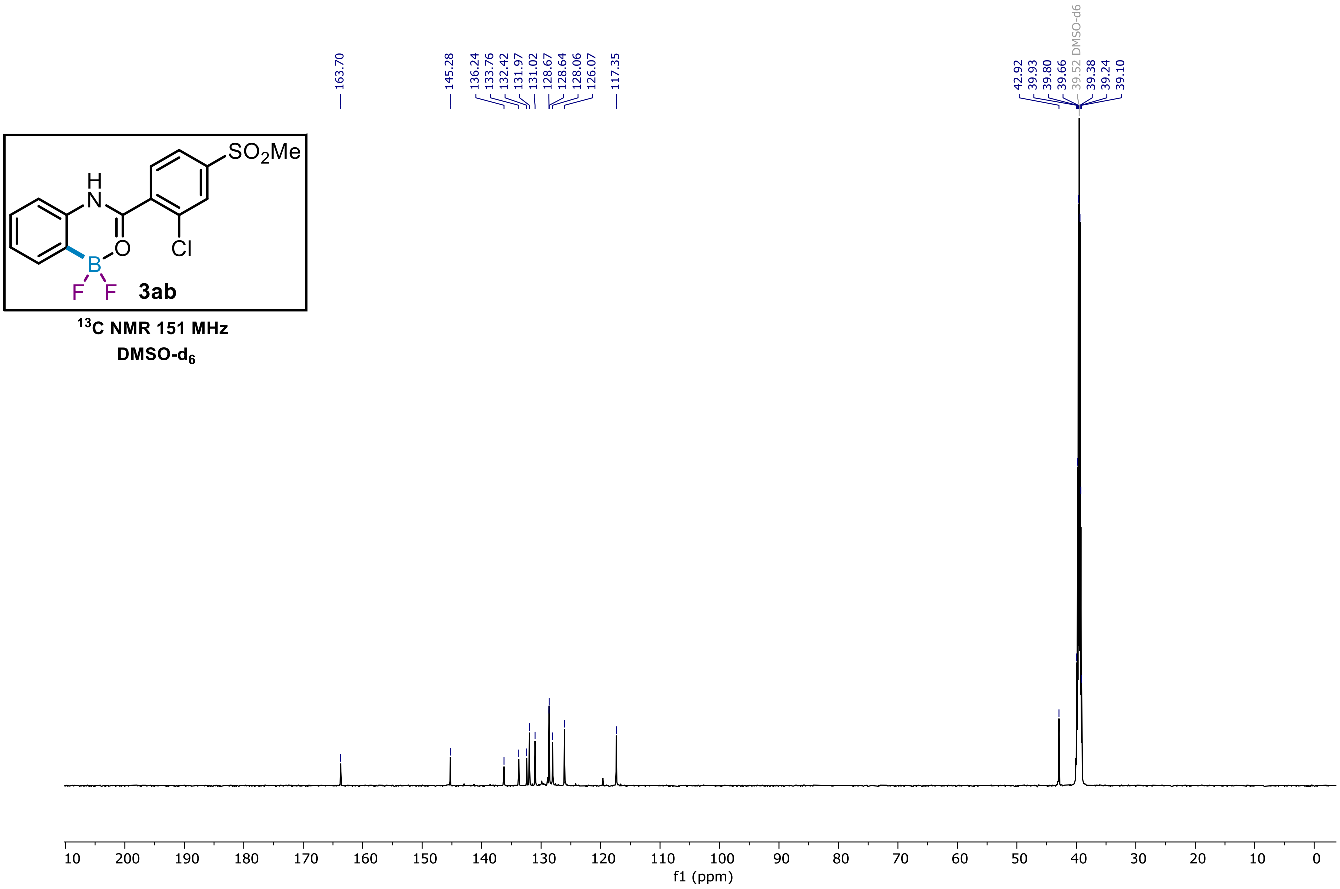

Figure S11-28: ^{13}C spectrum of compound **3ab** in DMSO-d_6. Note that the ^{13}C signal for the C-BF_2 bond does not appear.

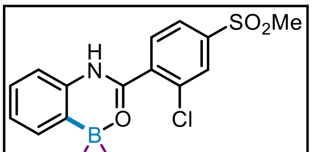

3ab

^{19}F NMR 659 MHz
DMSO-d_6

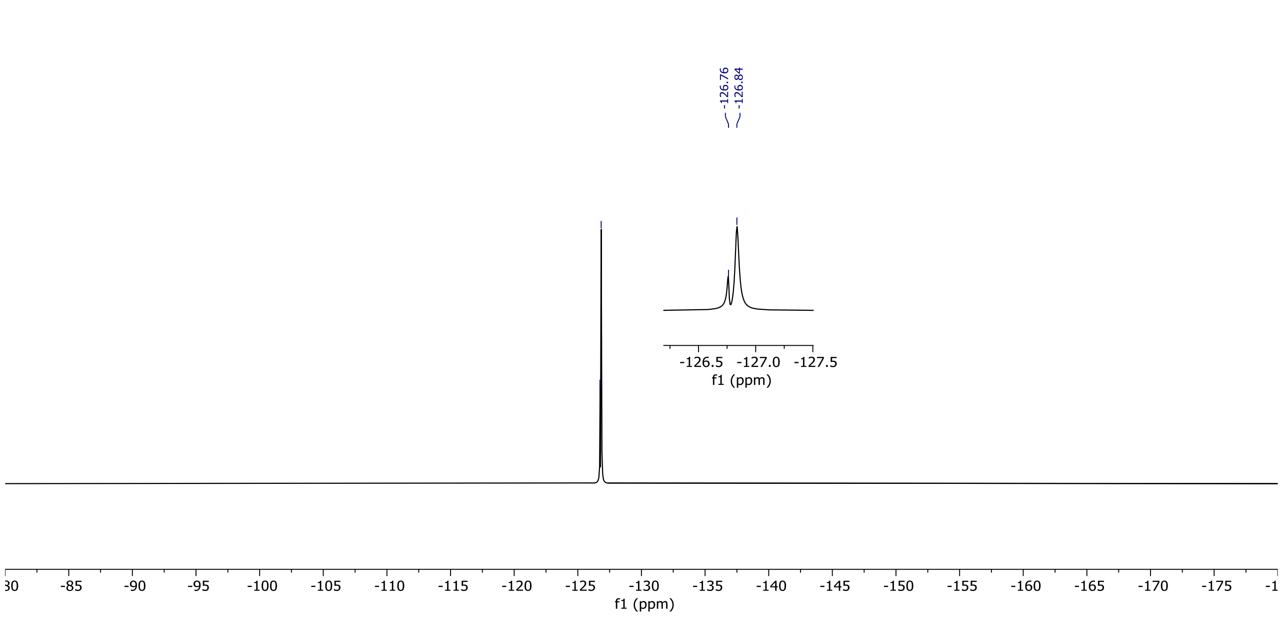

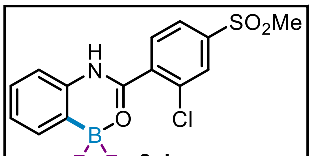

3ab

^{11}B NMR 193 MHz
DMSO-d_6

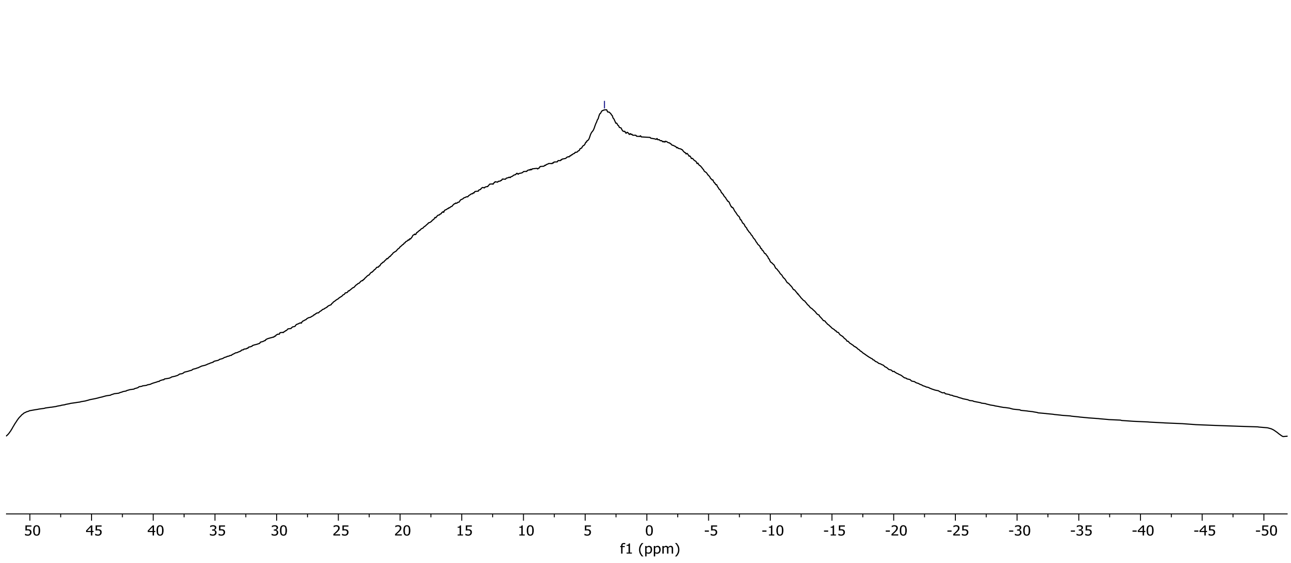

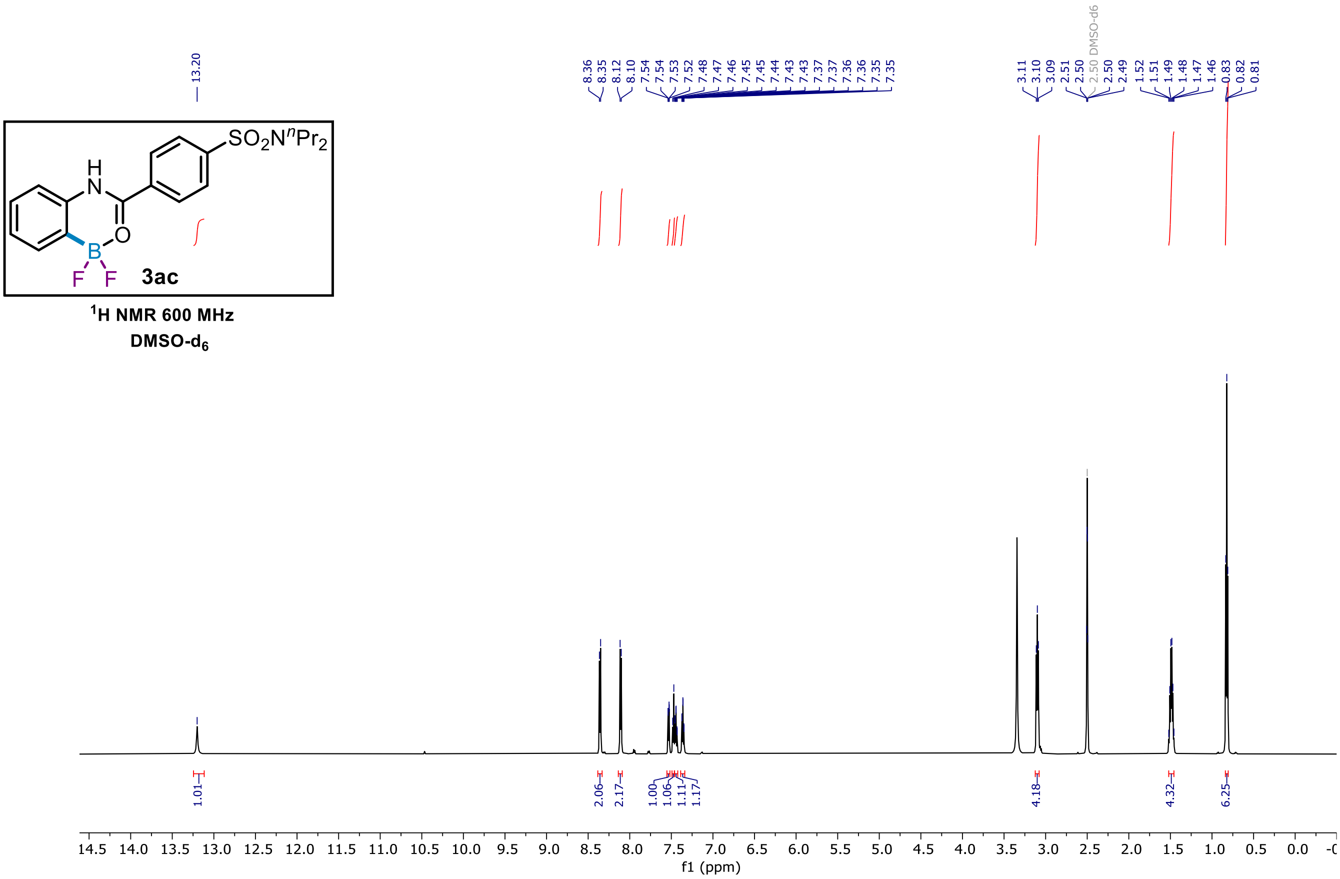

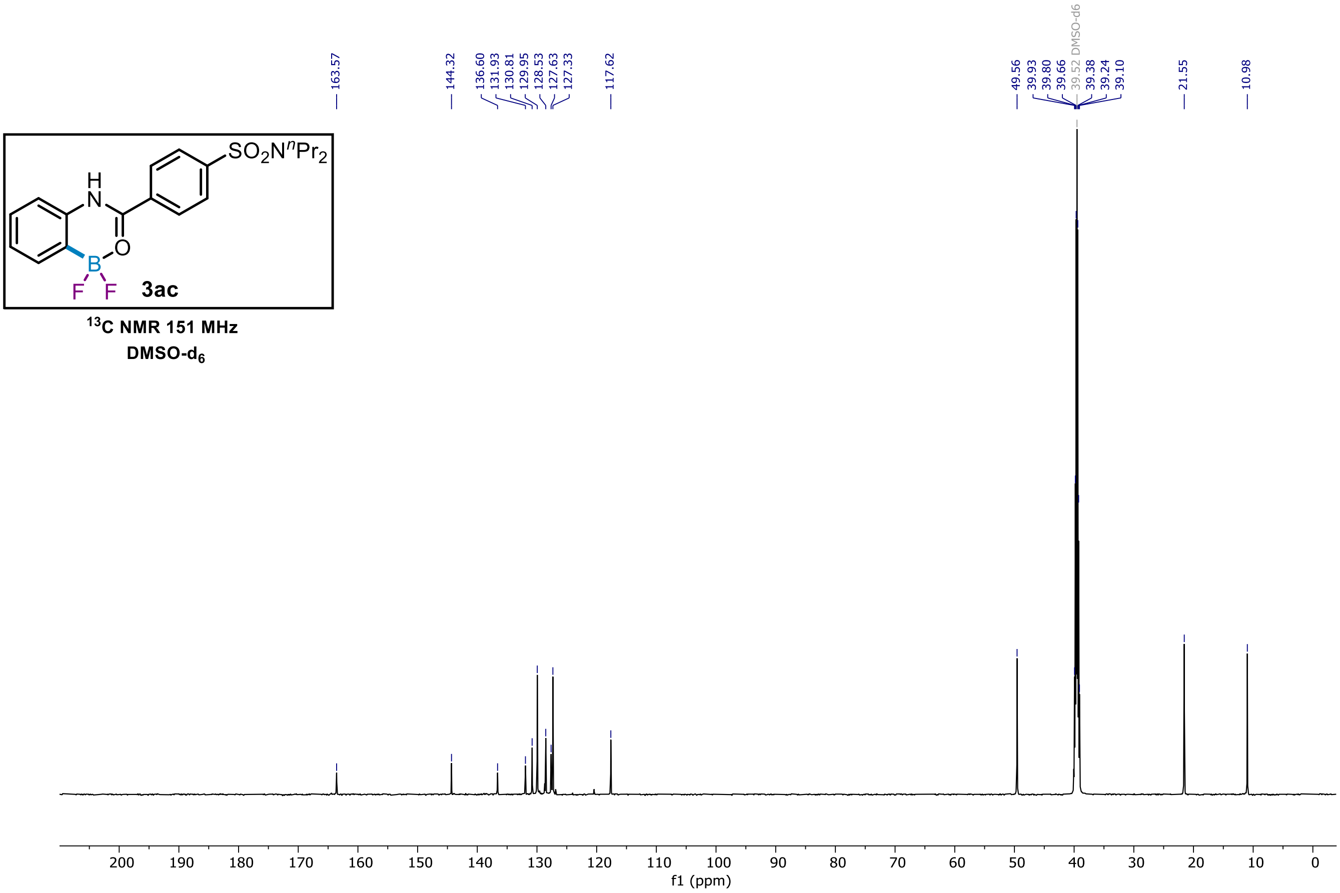

Figure S11-29: ^{13}C spectrum of compound **3ac** in DMSO-d_6. Note that the ^{13}C signal for the C-BF_2 bond does not appear.

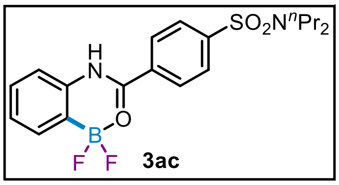

^{19}F NMR 659 MHz
DMSO-d$_6$

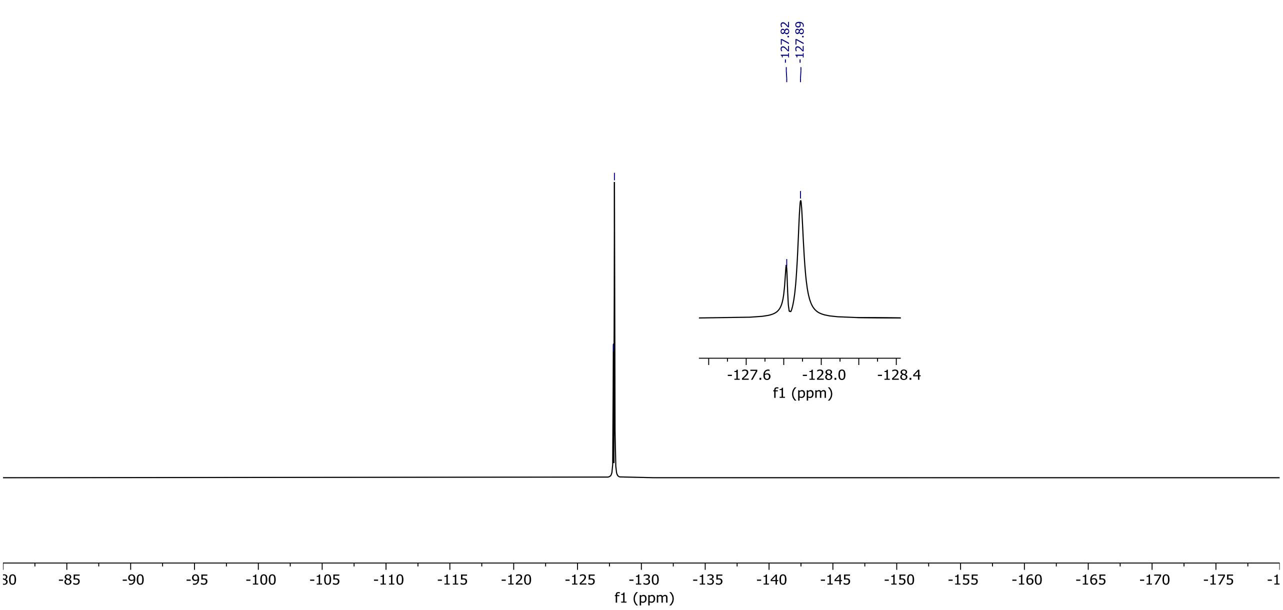

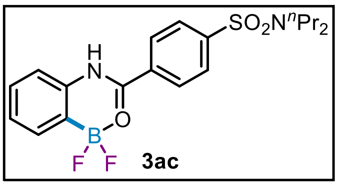

^{11}B NMR 193 MHz
DMSO-d$_6$

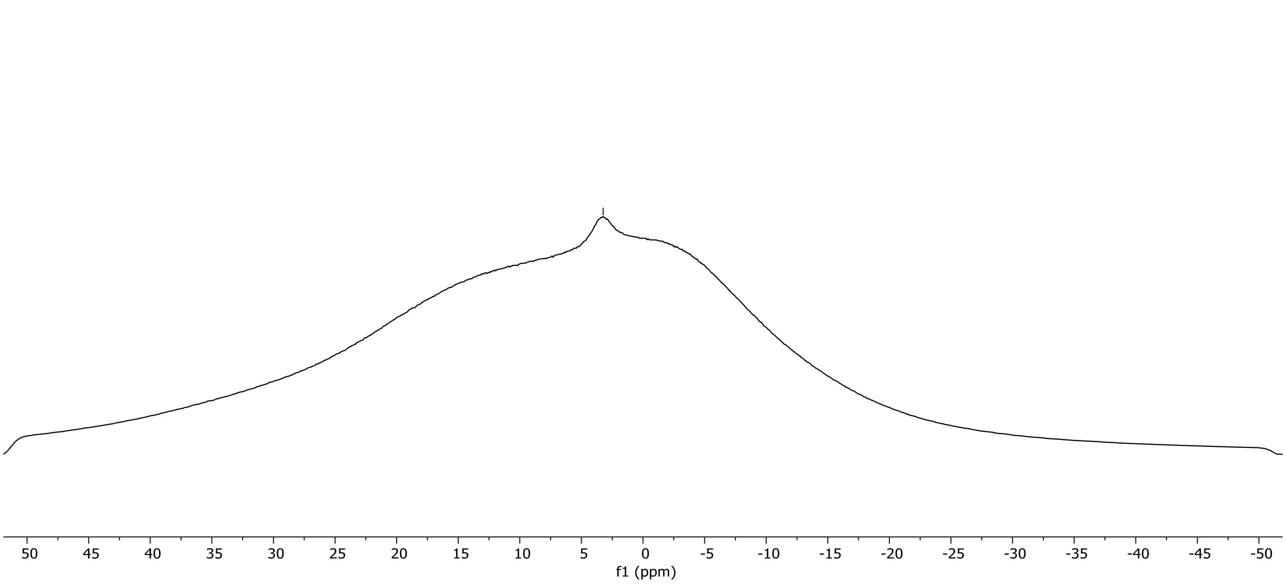

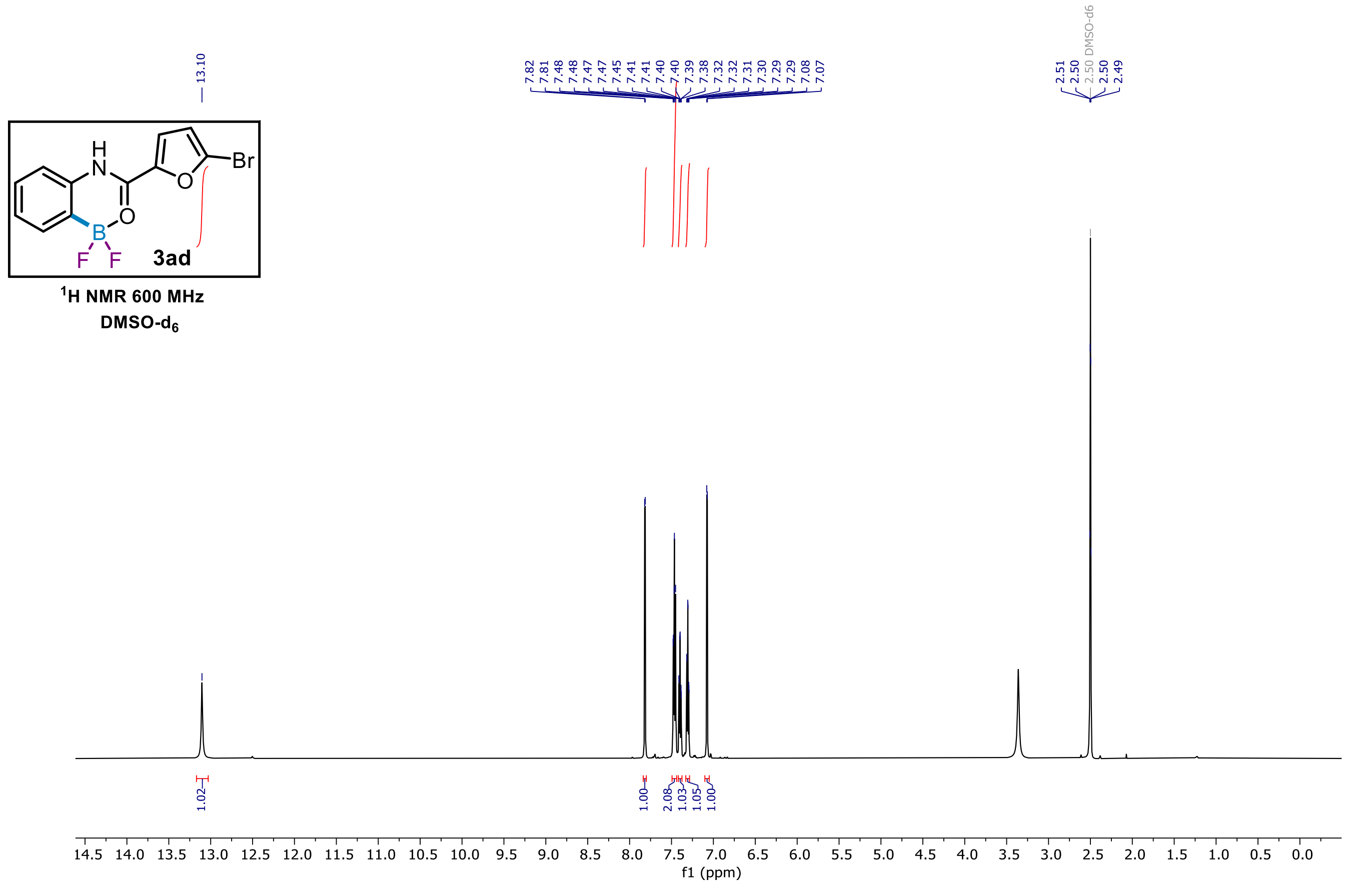

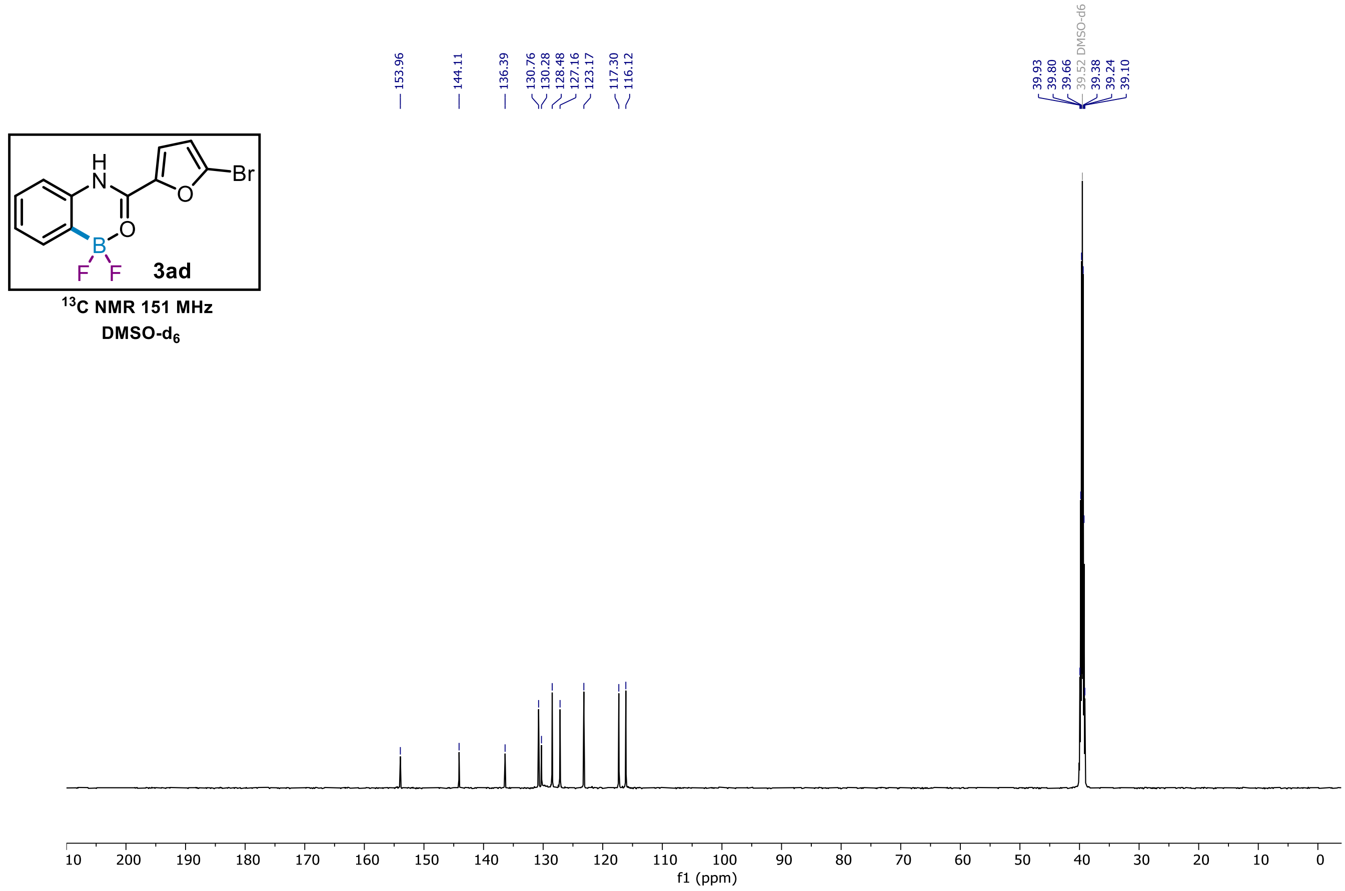

Figure S11-30: ^{13}C spectrum of compound **3ad** in DMSO-d$_6$. Note that the ^{13}C signal for the C-BF_2 bond does not appear.

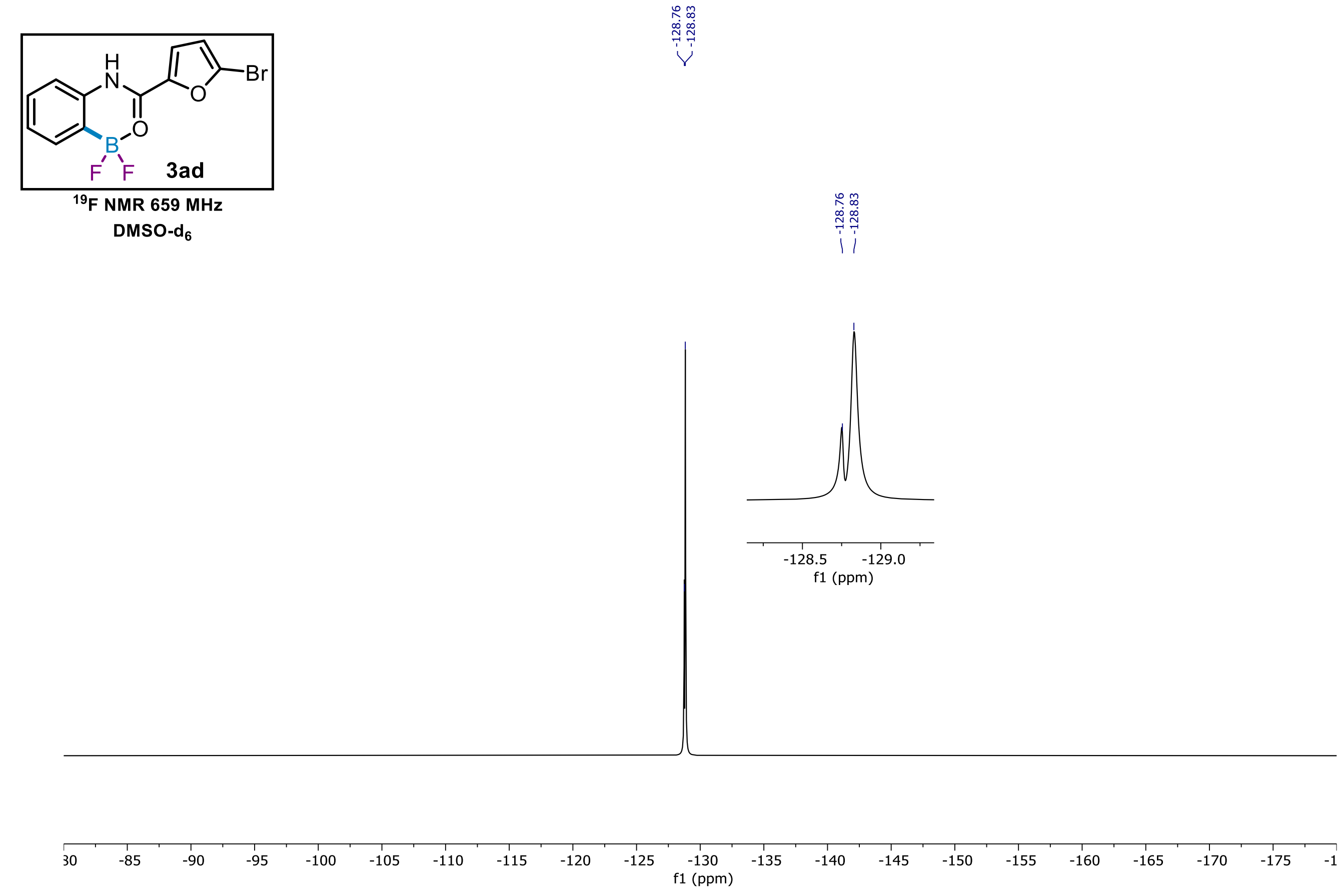

^{19}F NMR 659 MHz
DMSO-d_6

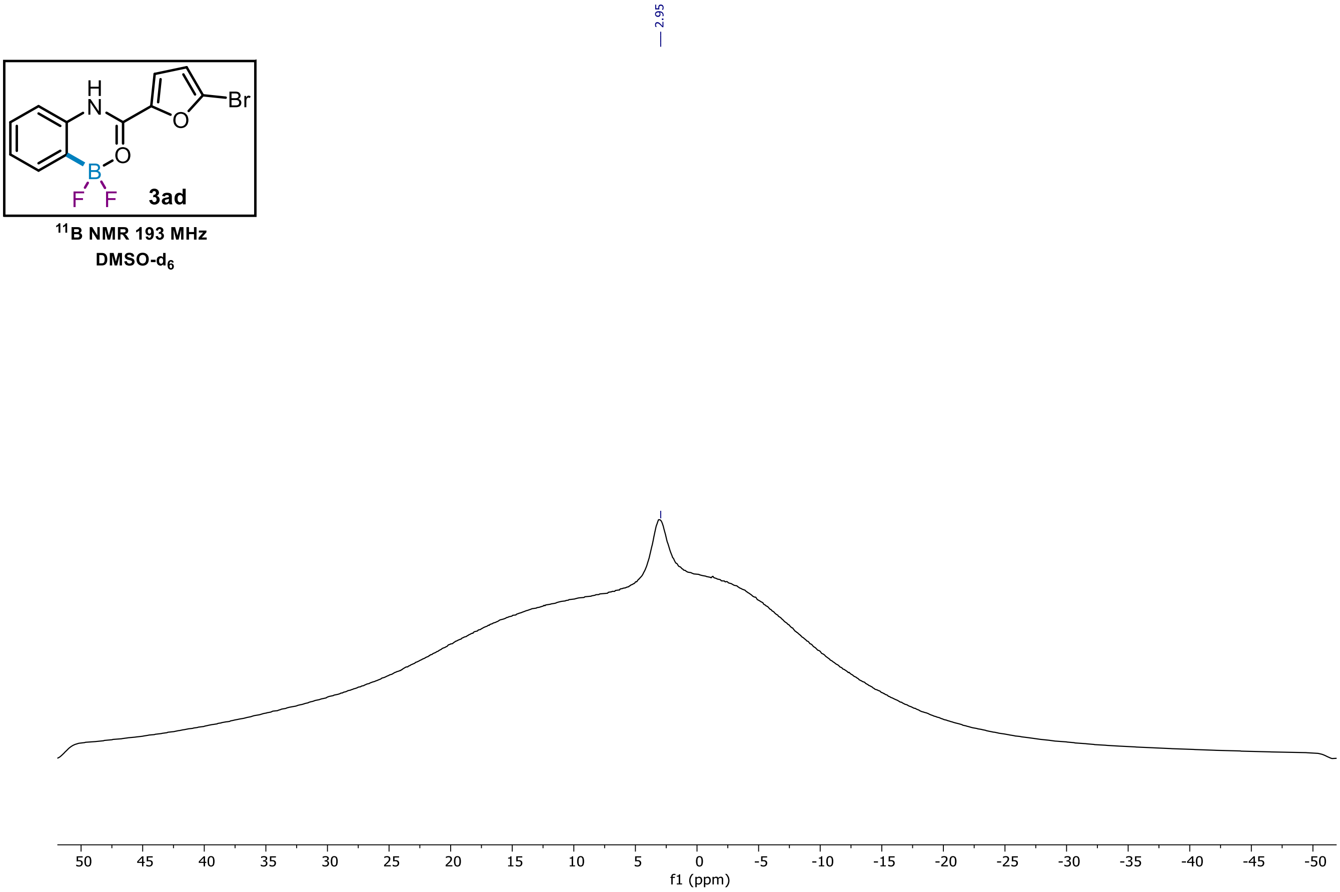

^{11}B NMR 193 MHz
DMSO-d_6

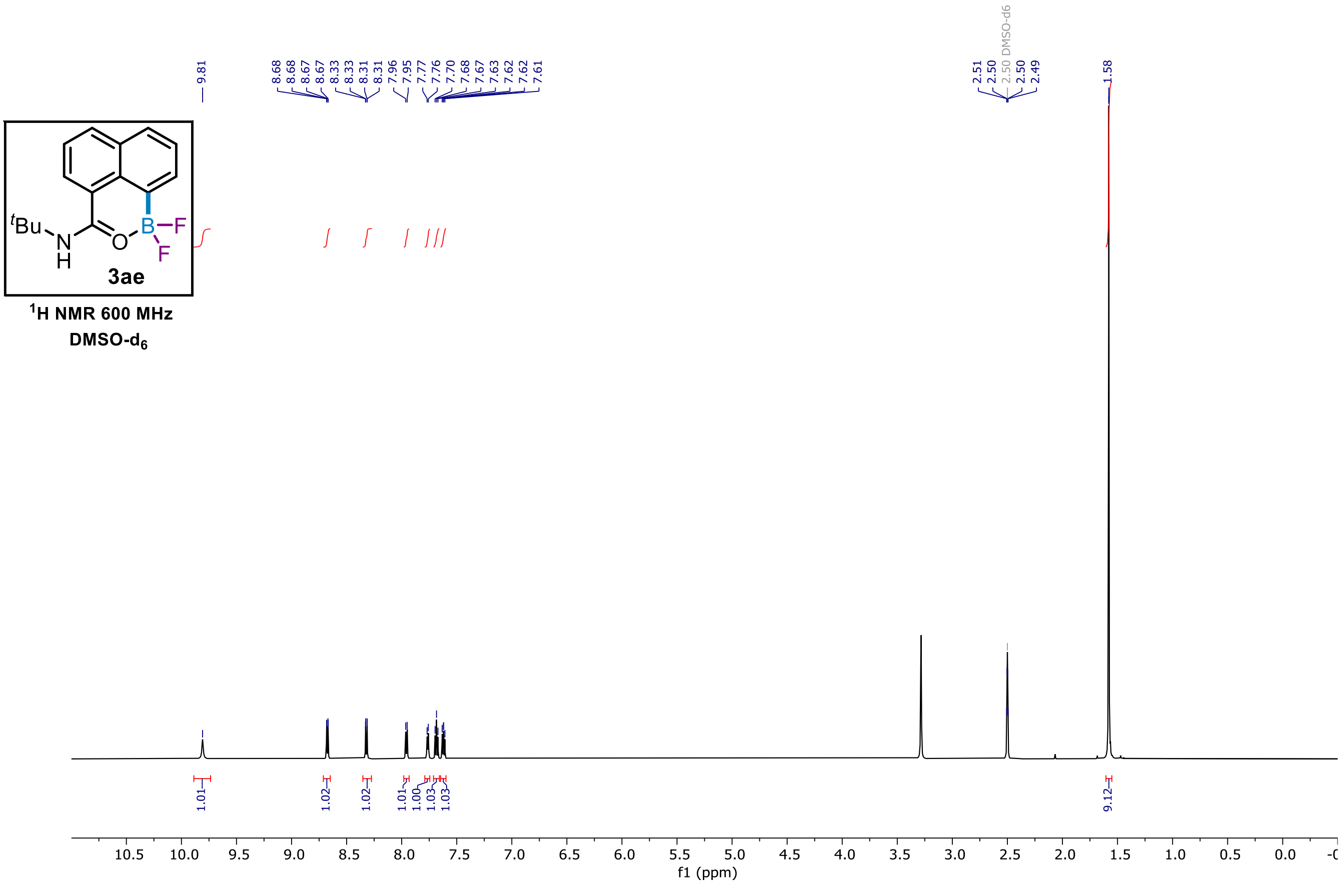

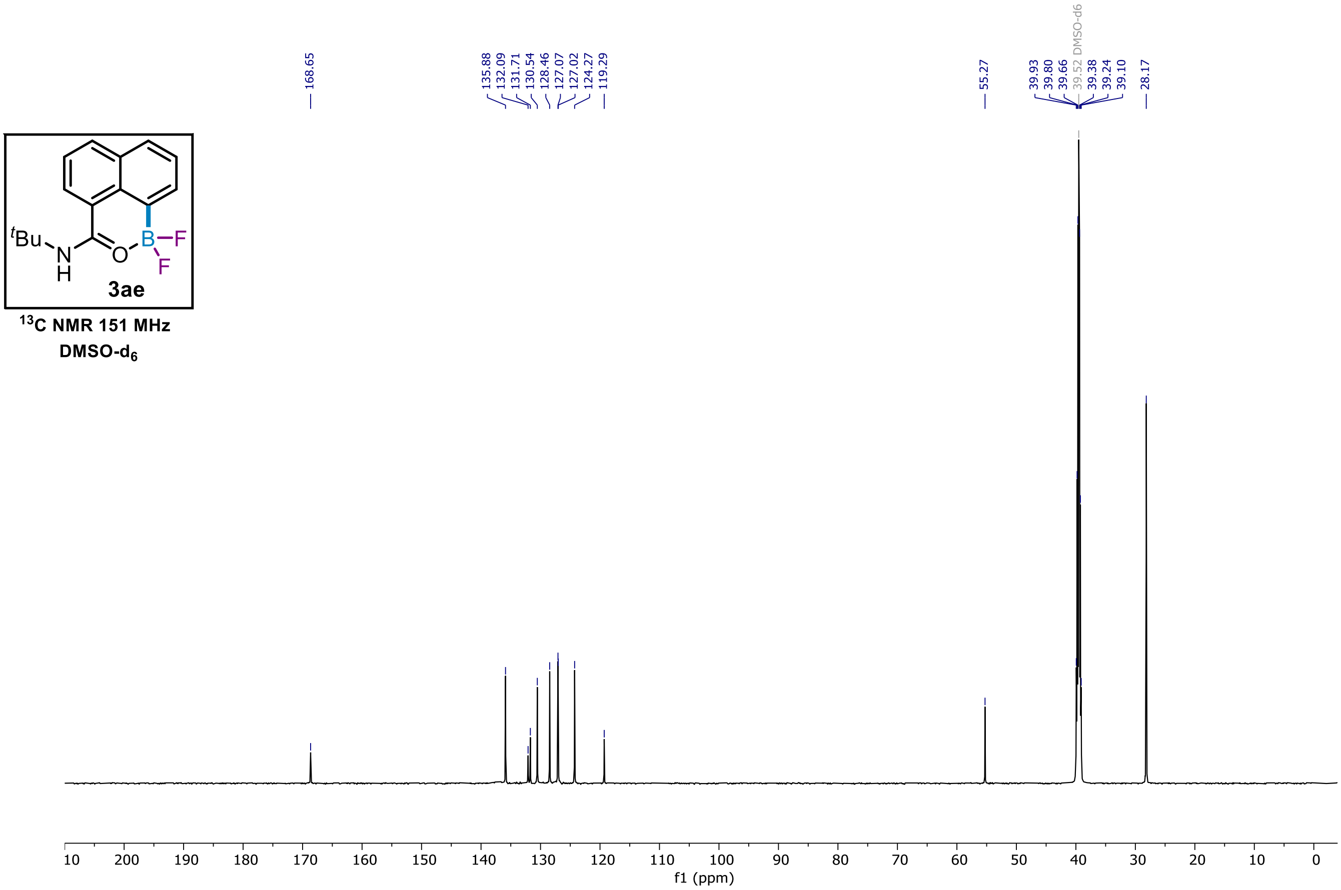

Figure S11-31: ^{13}C spectrum of compound **3ae** in DMSO-d_6. Note that the ^{13}C signal for the C-BF_2 bond does not appear.

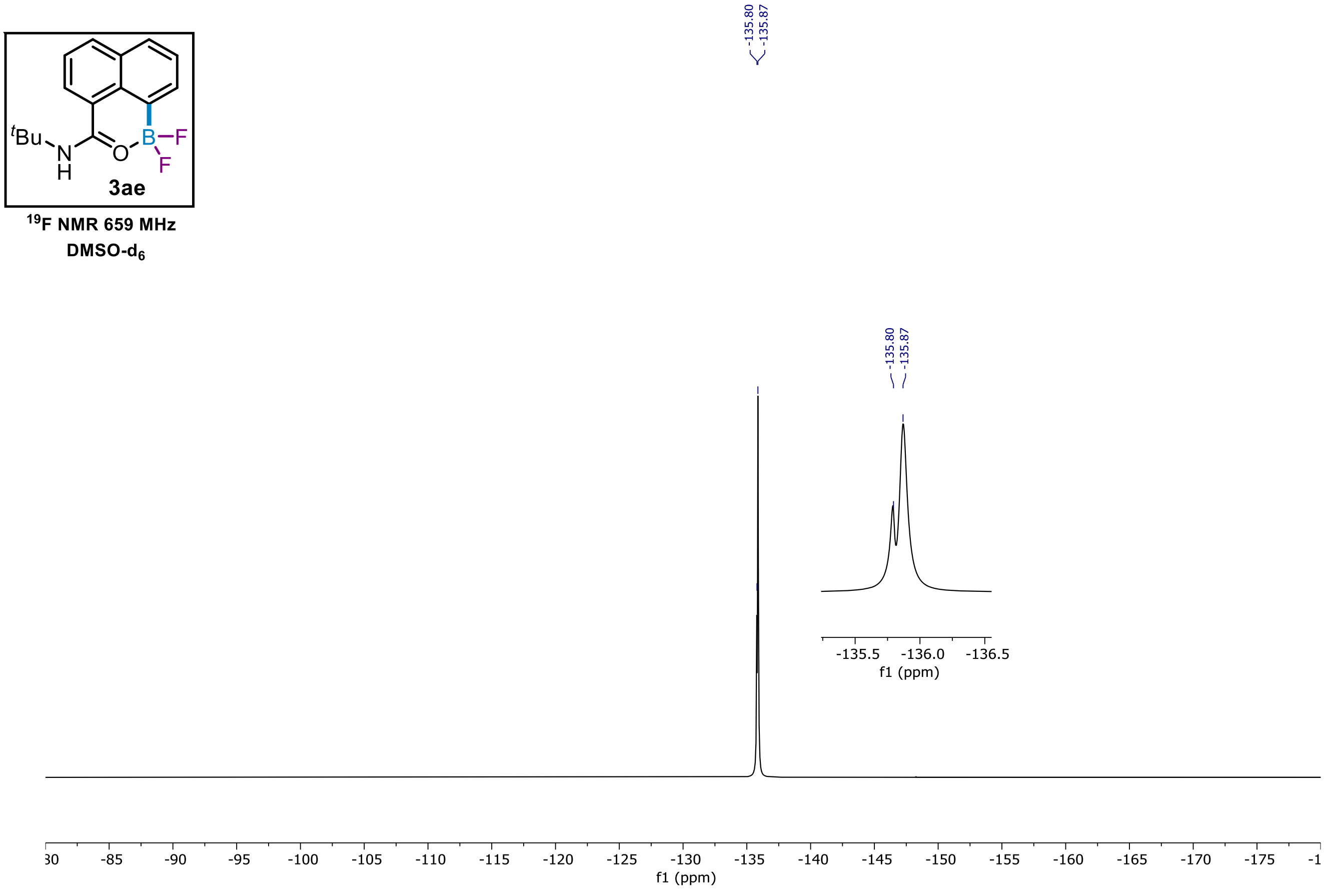

tBu
N
H
O
B
F
F
3ae
19F NMR 659 MHz
DMSO-d6
-135.80
-135.87
-135.5
-136.0
-136.5
f1 (ppm)
f1 (ppm)

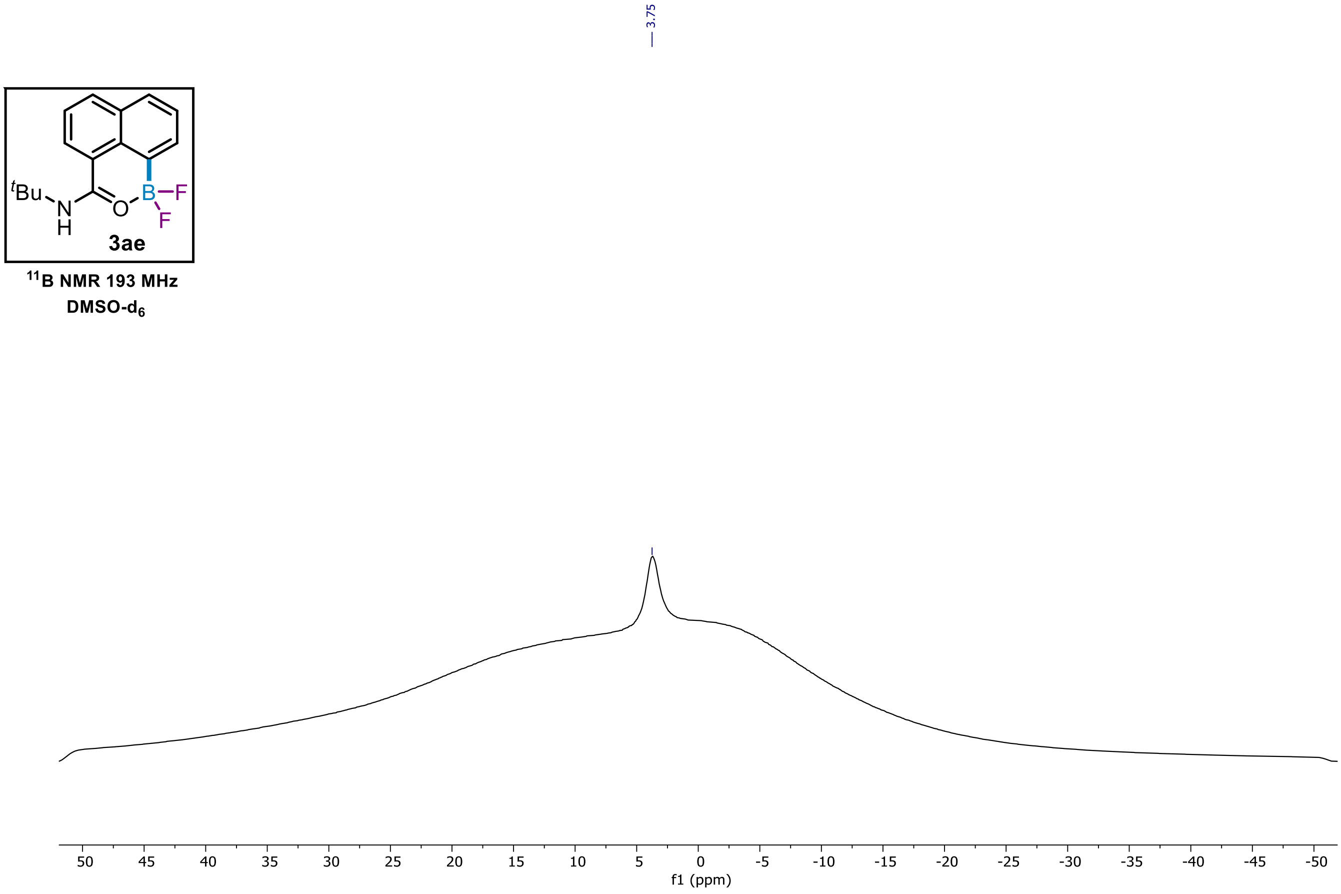

tBu
N
H
O
B
F
F
3ae
11B NMR 193 MHz
DMSO-d6
3.75
f1 (ppm)

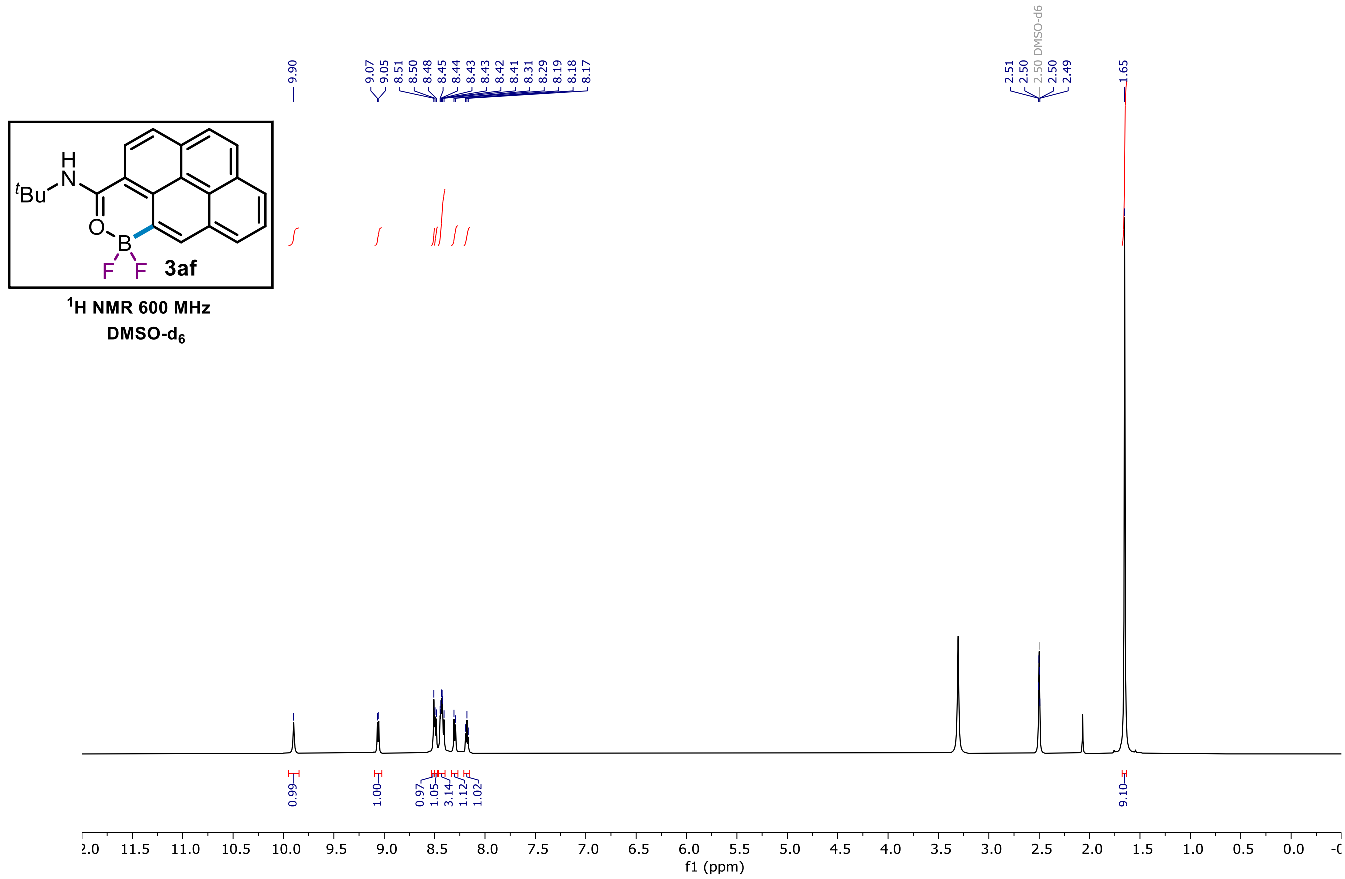

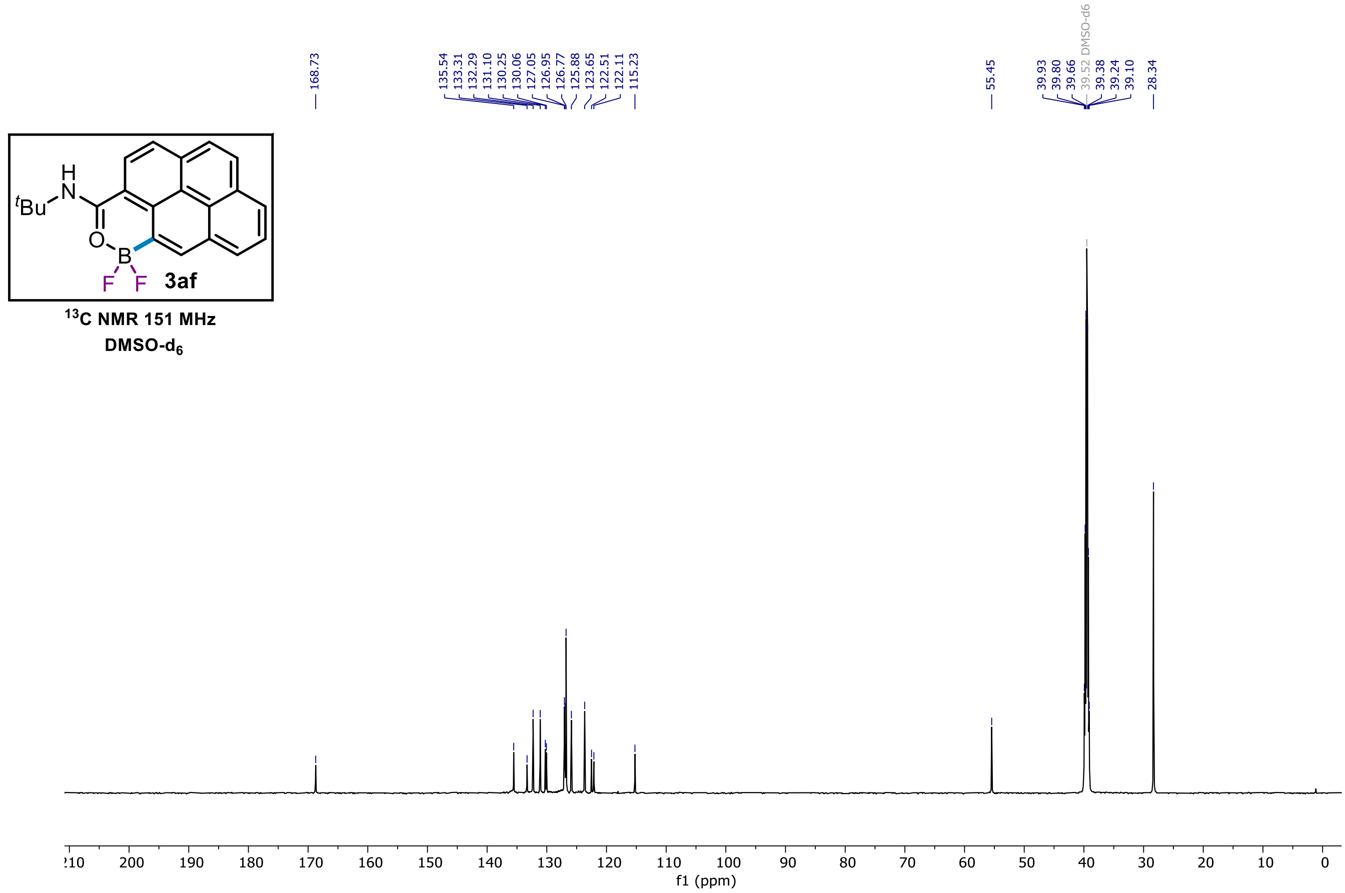

Figure S11-32: ^{13}C spectrum of compound **3af** in DMSO-d_6. Note that the ^{13}C signal for the C-BF_2 bond does not appear.

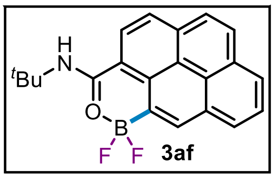

^{19}F NMR 659 MHz
DMSO-d_6

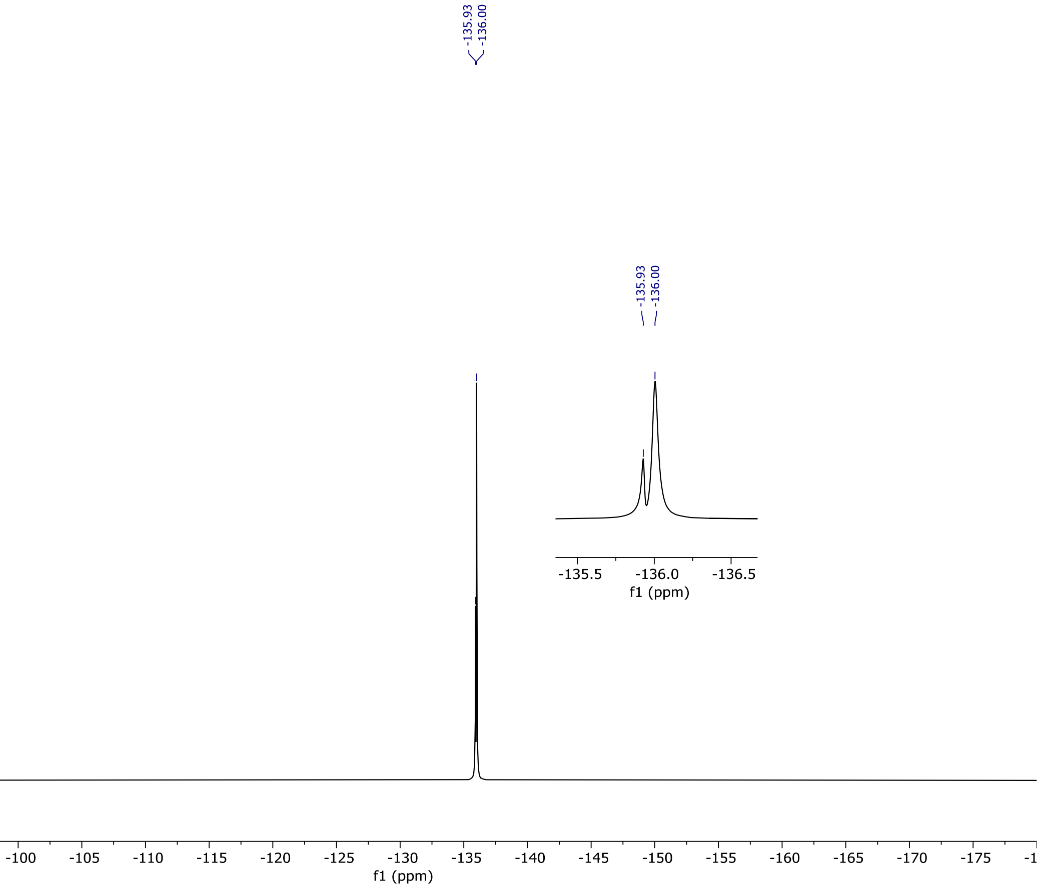

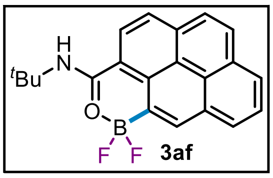

^{11}B NMR 193 MHz
DMSO-d_6

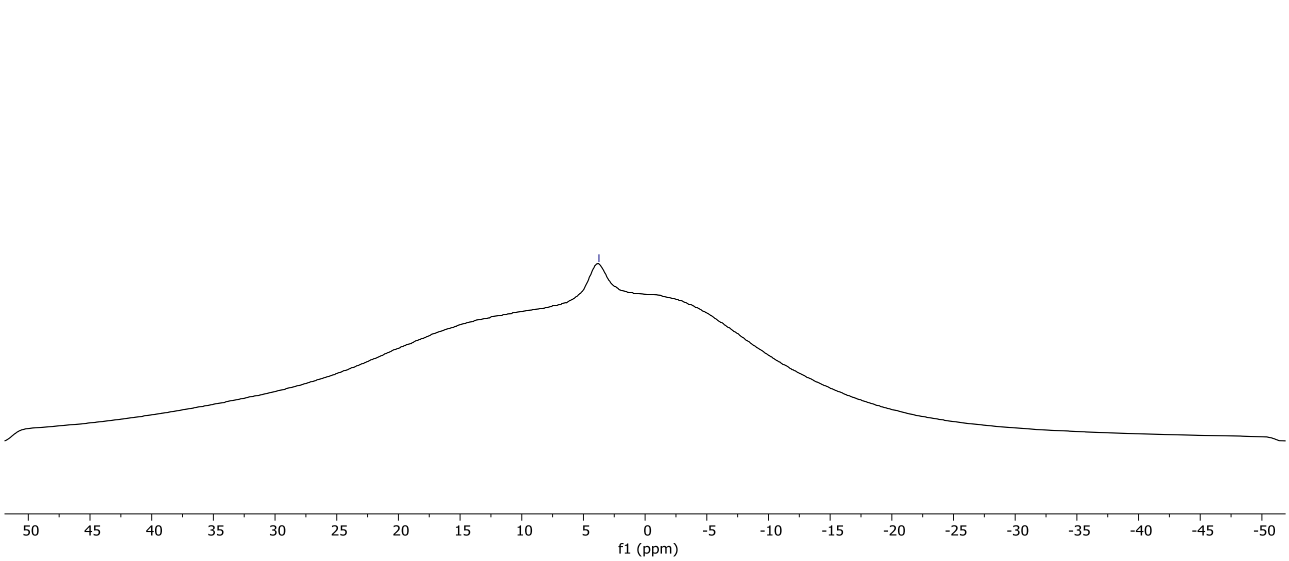

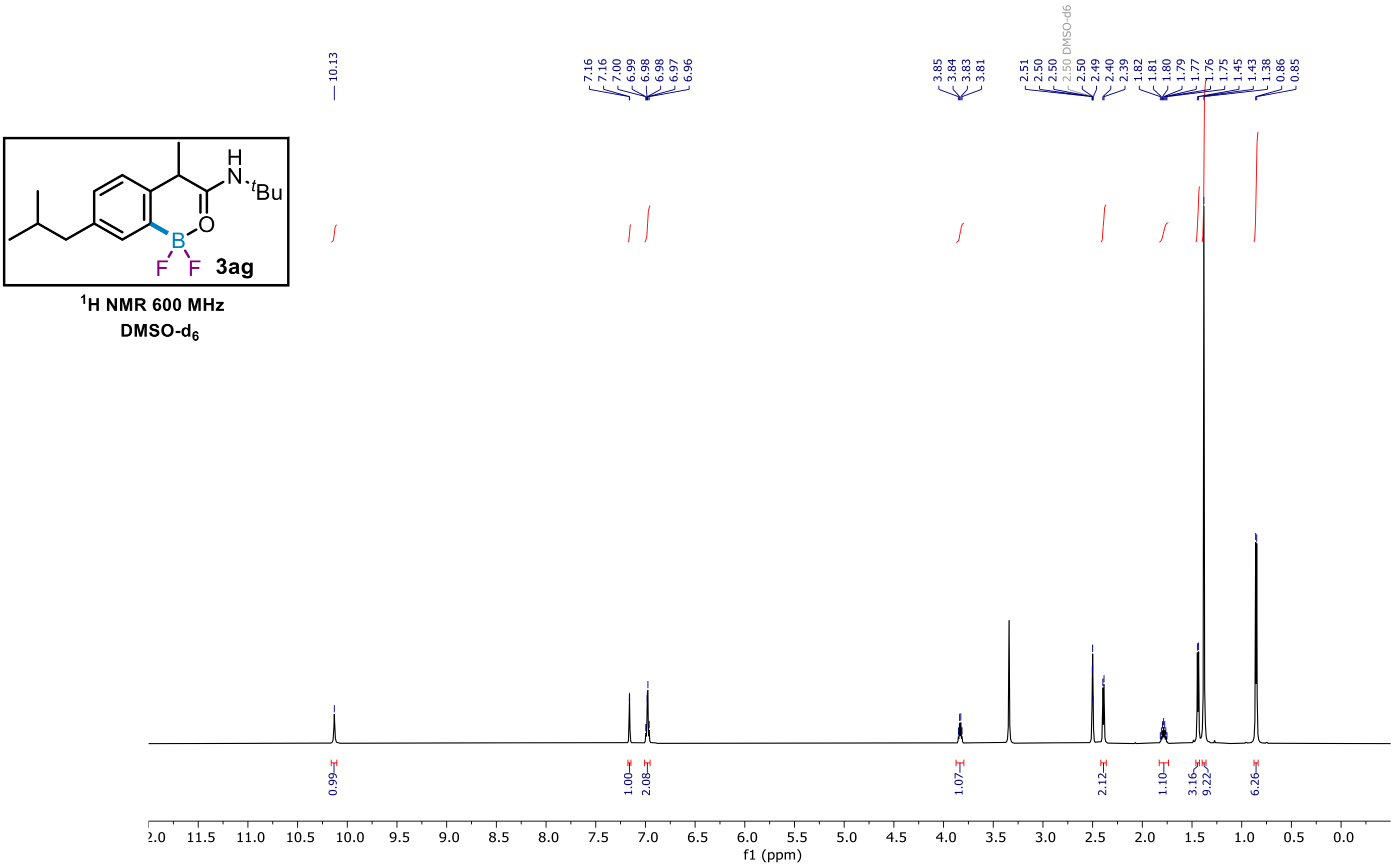

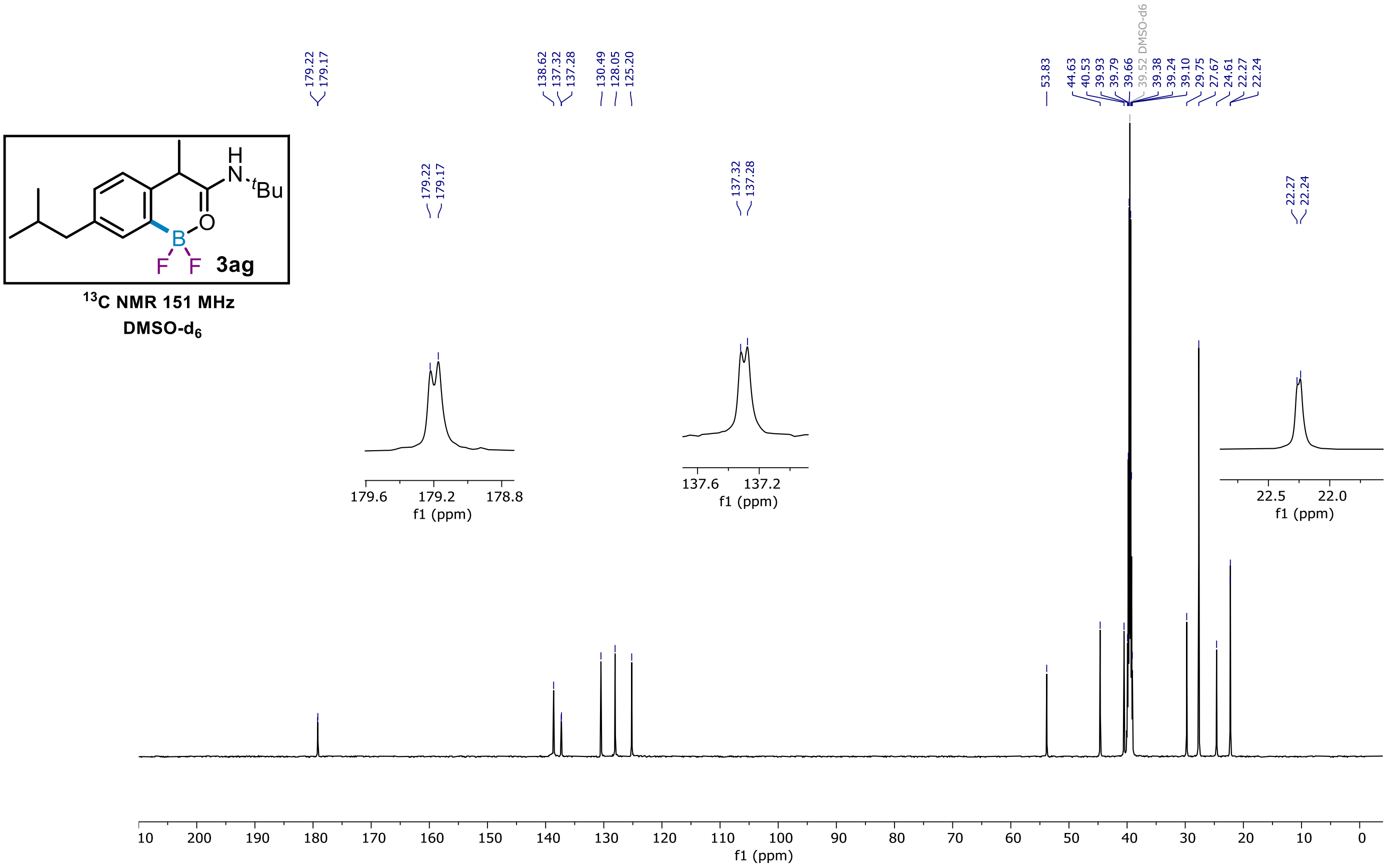

Figure S11-33-2: ^{13}C spectrum of compound **3ag** in DMSO-d6. Note that the ^{13}C signal for the C-BF_2 bond does not appear. Distereomers peaks were observed in the spectra.

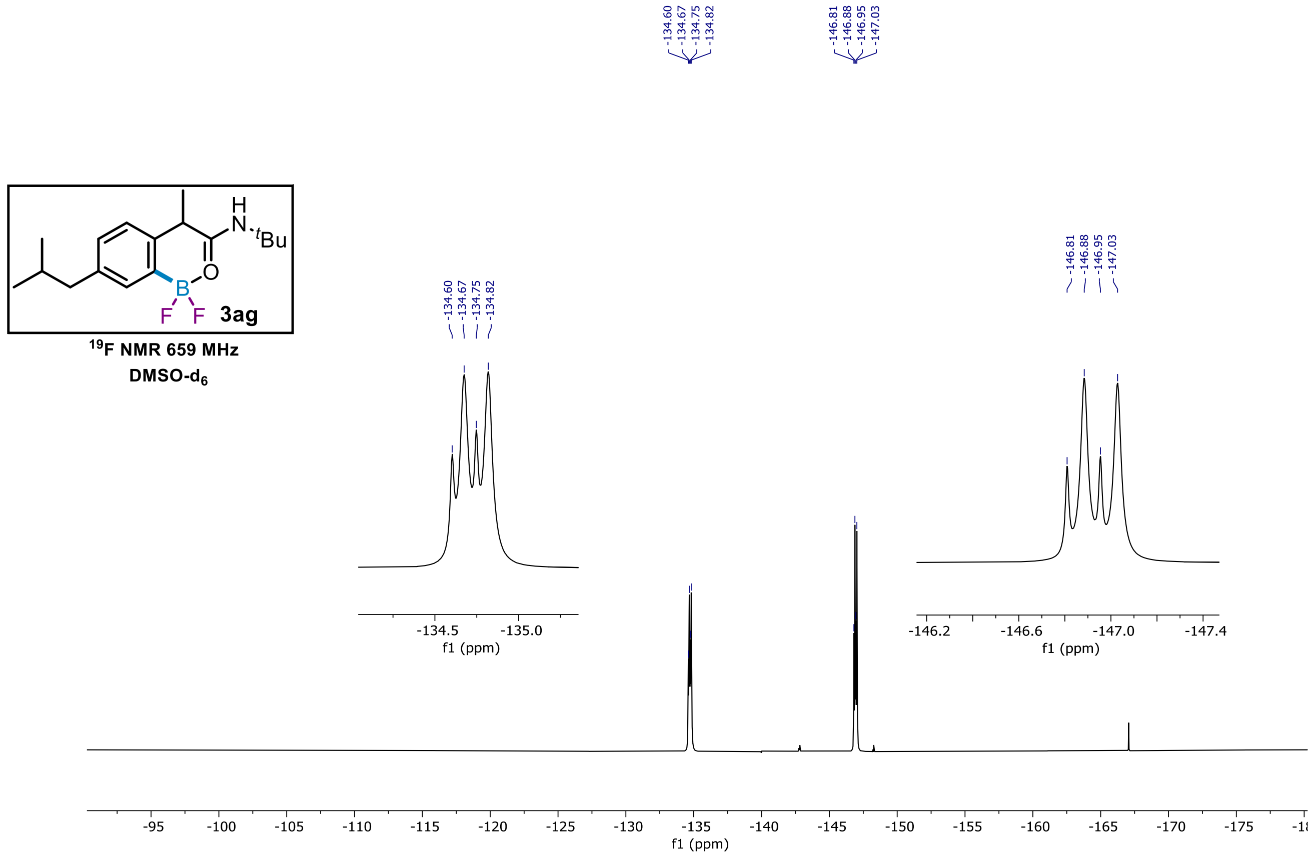

Figure S11-33-3: ^{19}F spectrum of compound **3ag** in DMSO-d_6. Two non equivalent fluorines spectrum in the two distereomers.

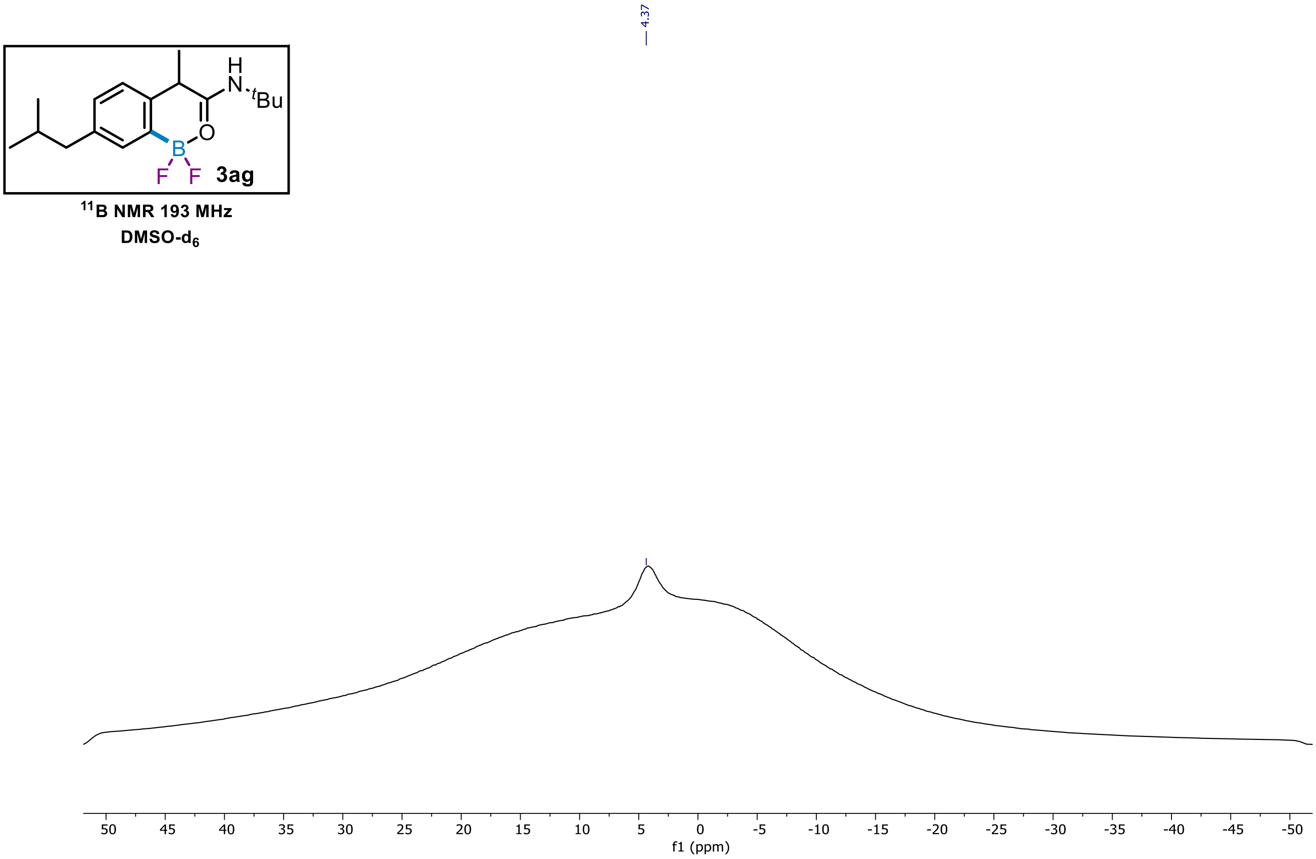

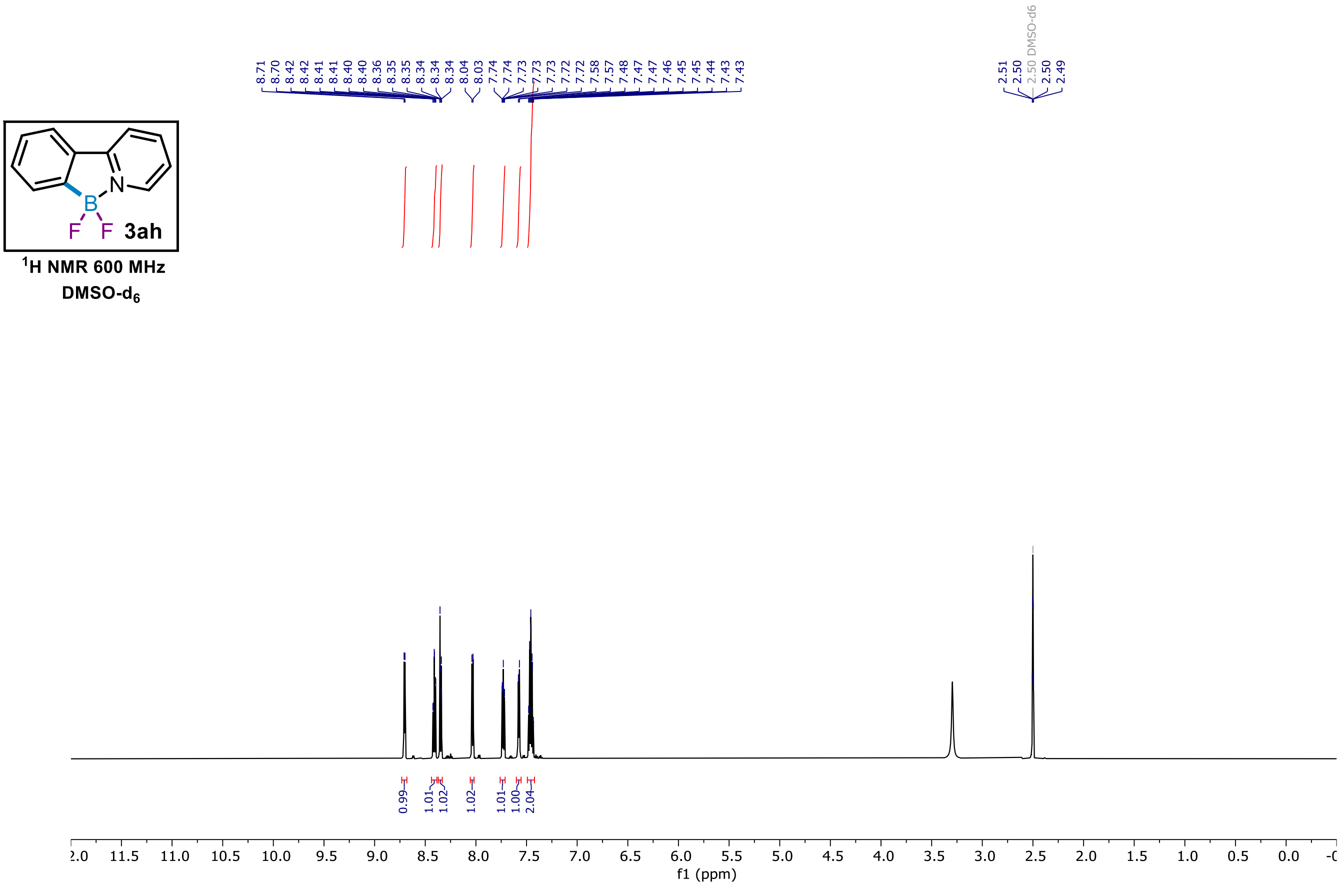

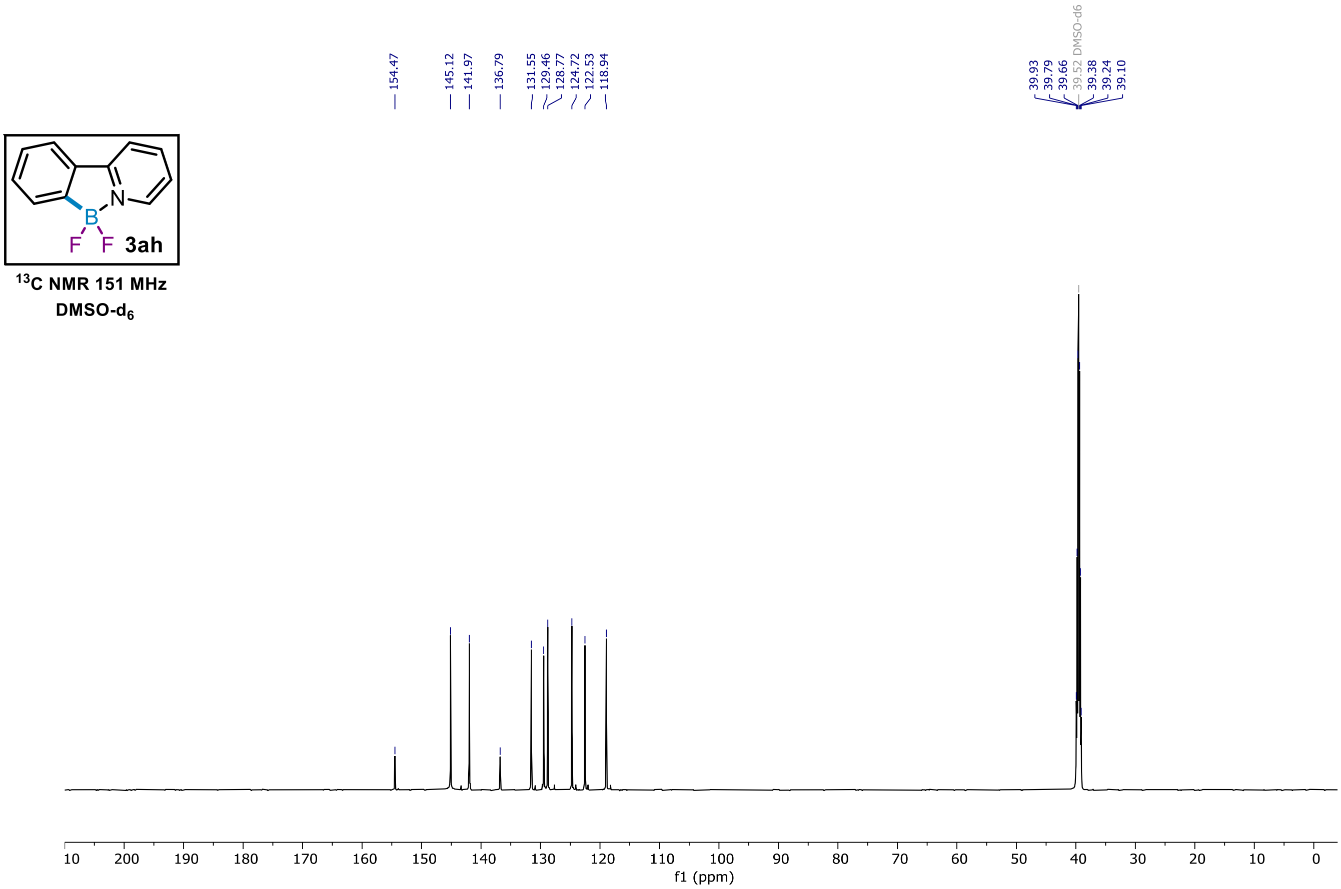

Figure S11-35: ^{13}C spectrum of compound **3ah** in DMSO-d_6. Note that the ^{13}C signal for the C-BF_2 bond does not appear.

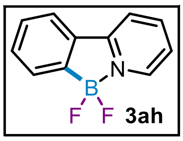

^{19}F NMR 659 MHz
DMSO-d_6

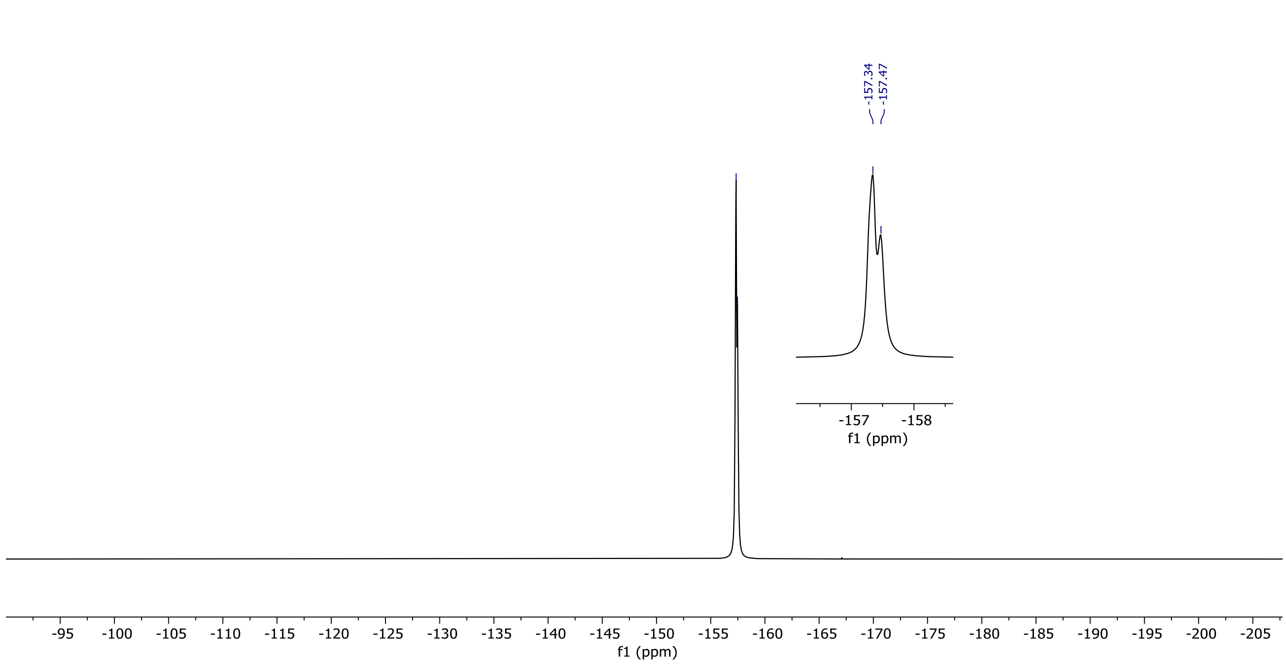

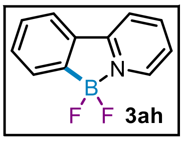

^{11}B NMR 193 MHz
DMSO-d_6

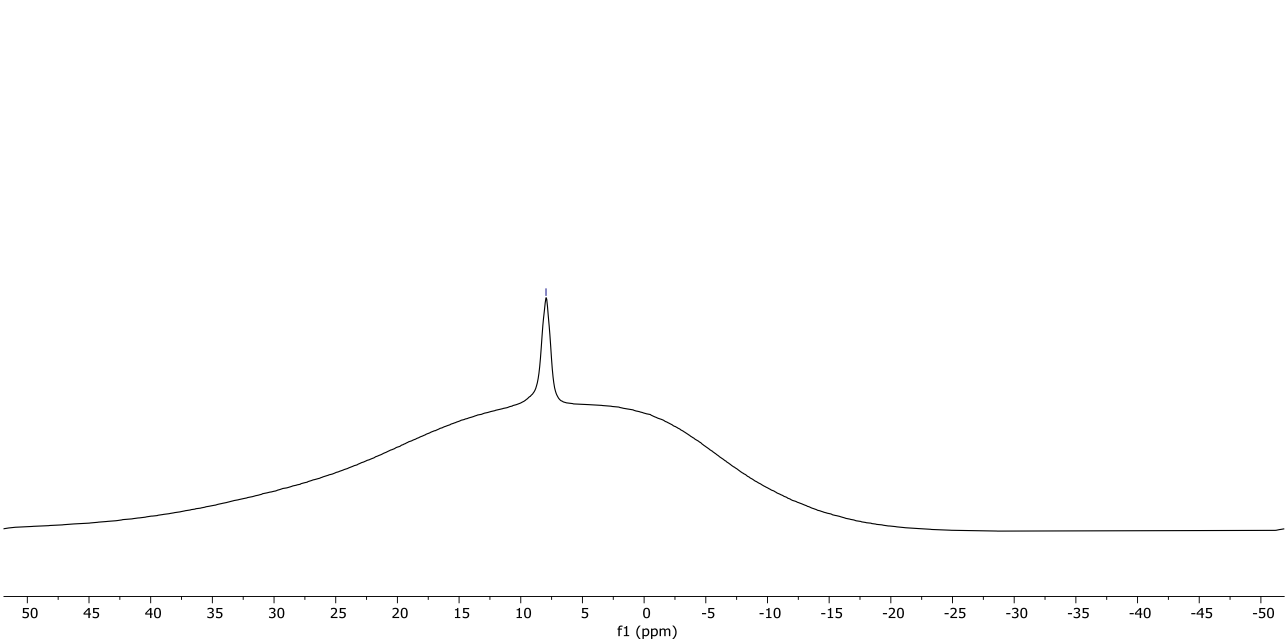

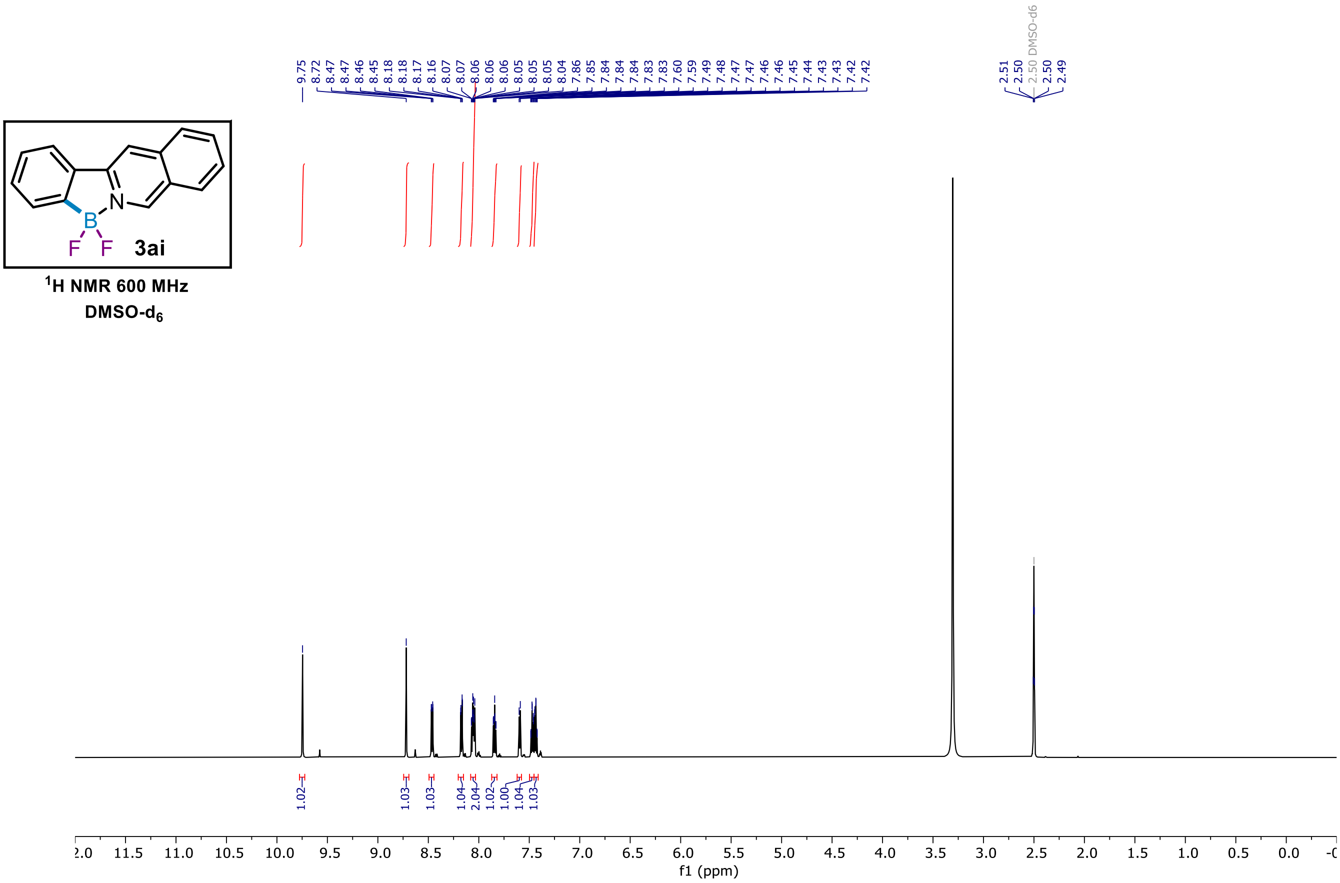

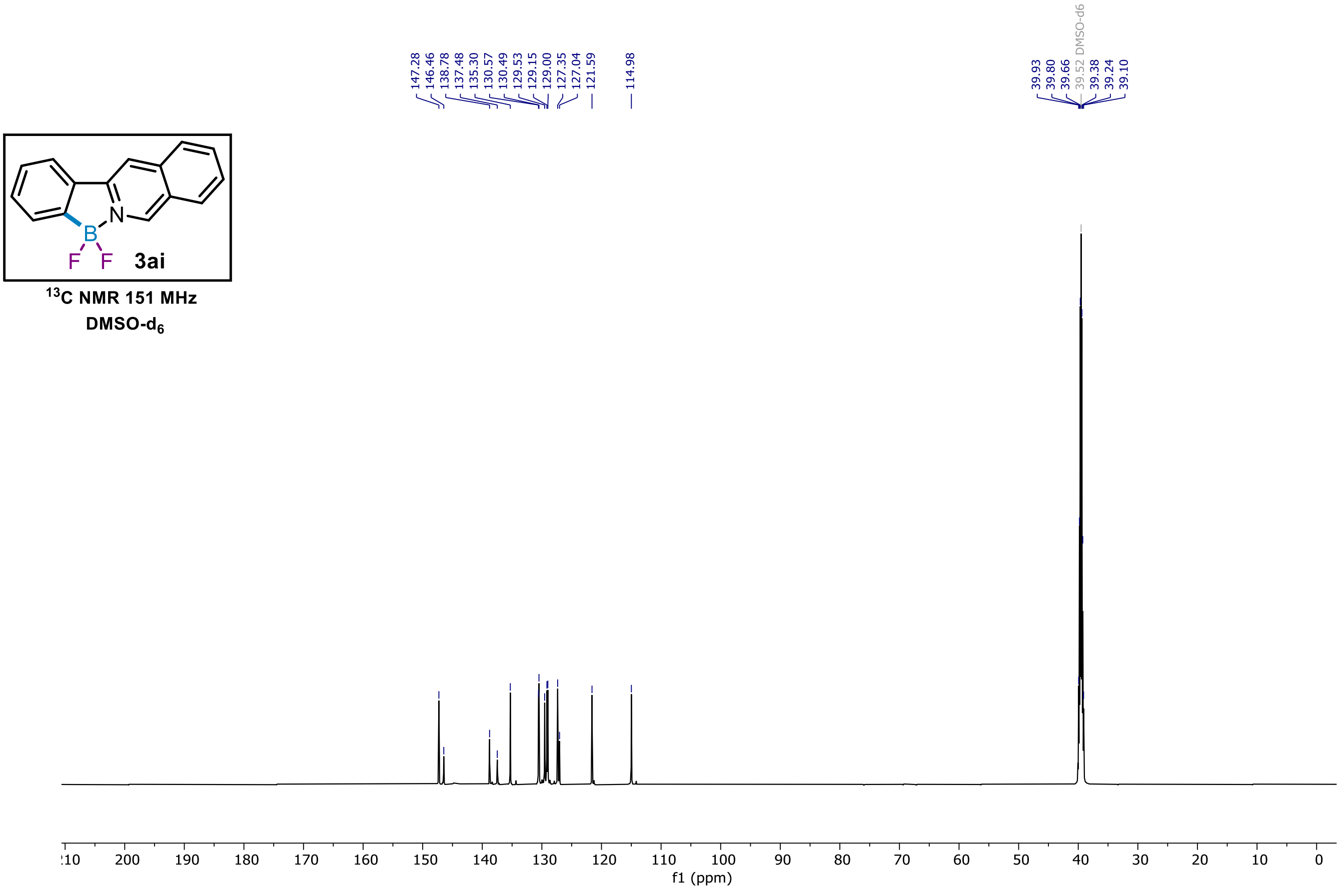

Figure S11-36: ^{13}C spectrum of compound **3ai** in DMSO-d_6. Note that the ^{13}C signal for the C-BF_2 bond does not appear.

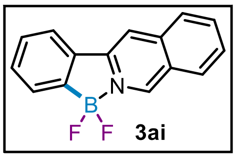

^{19}F NMR 659 MHz
DMSO-d$_6$

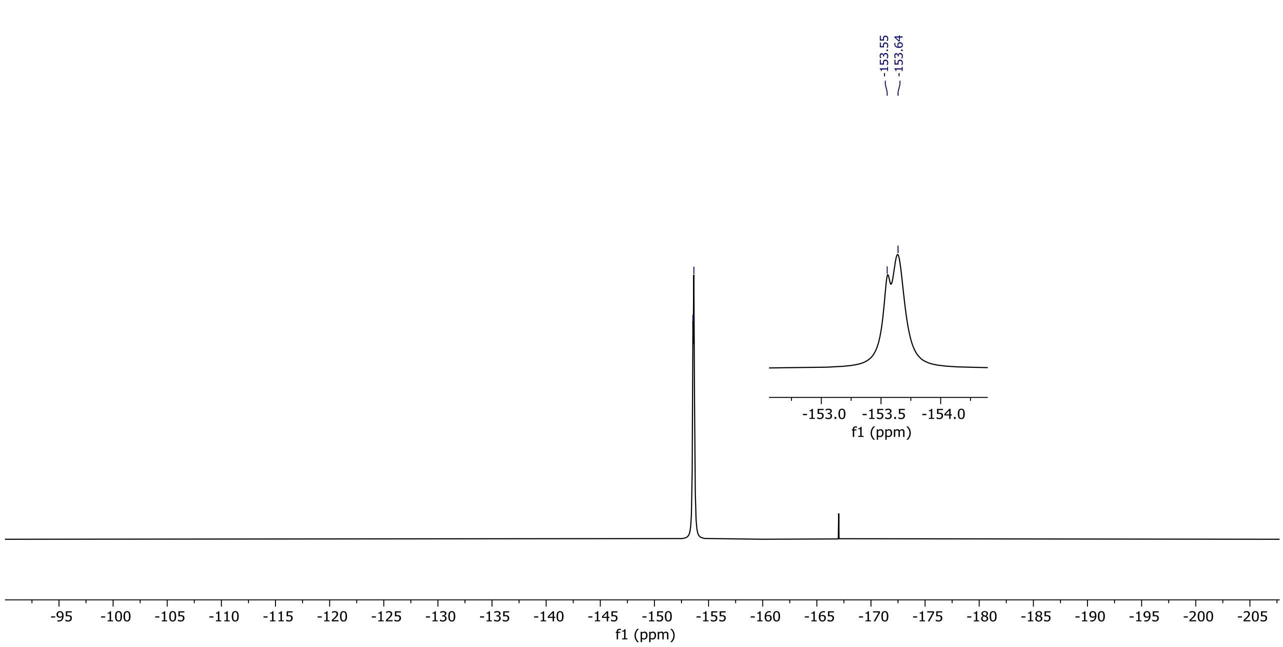

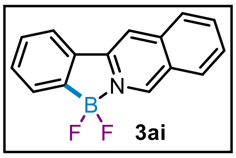

^{11}B NMR 193 MHz
DMSO-d$_6$

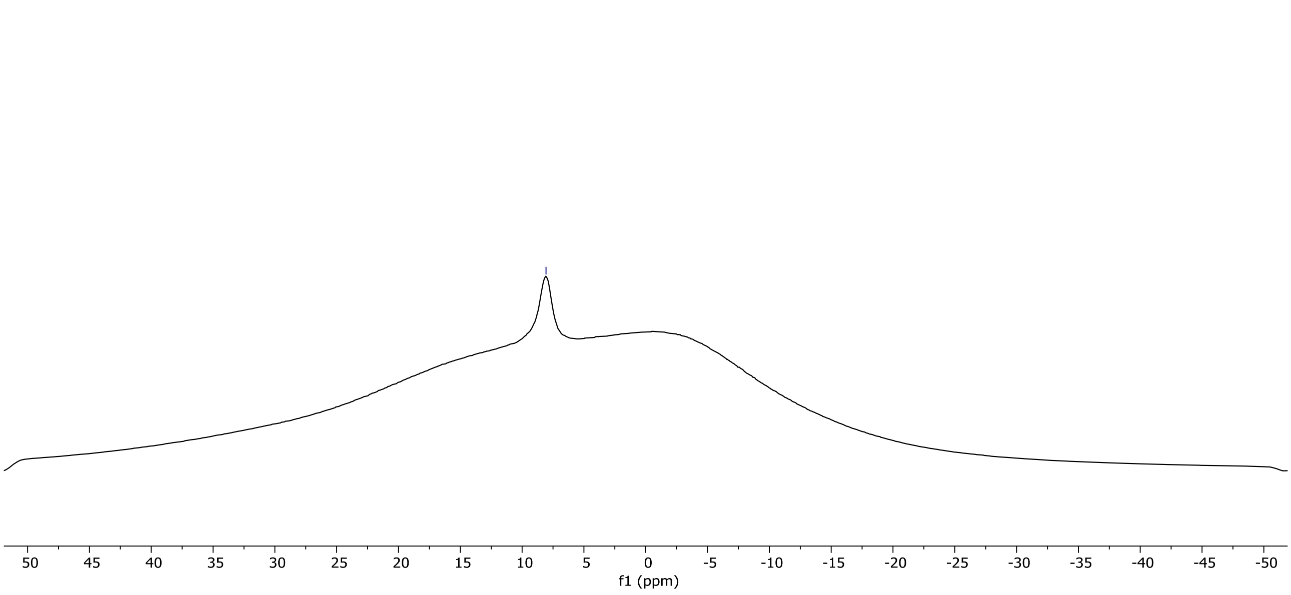

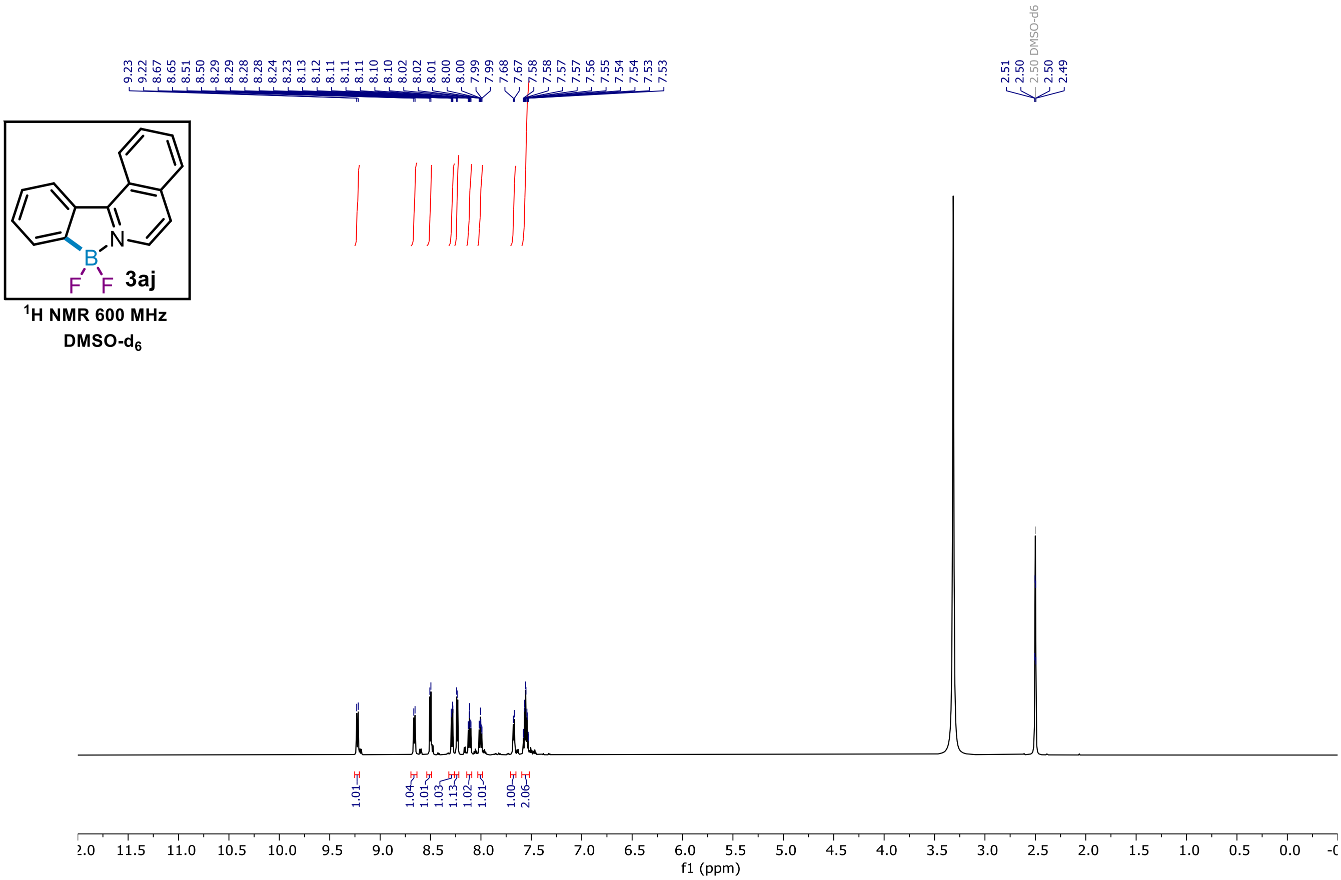

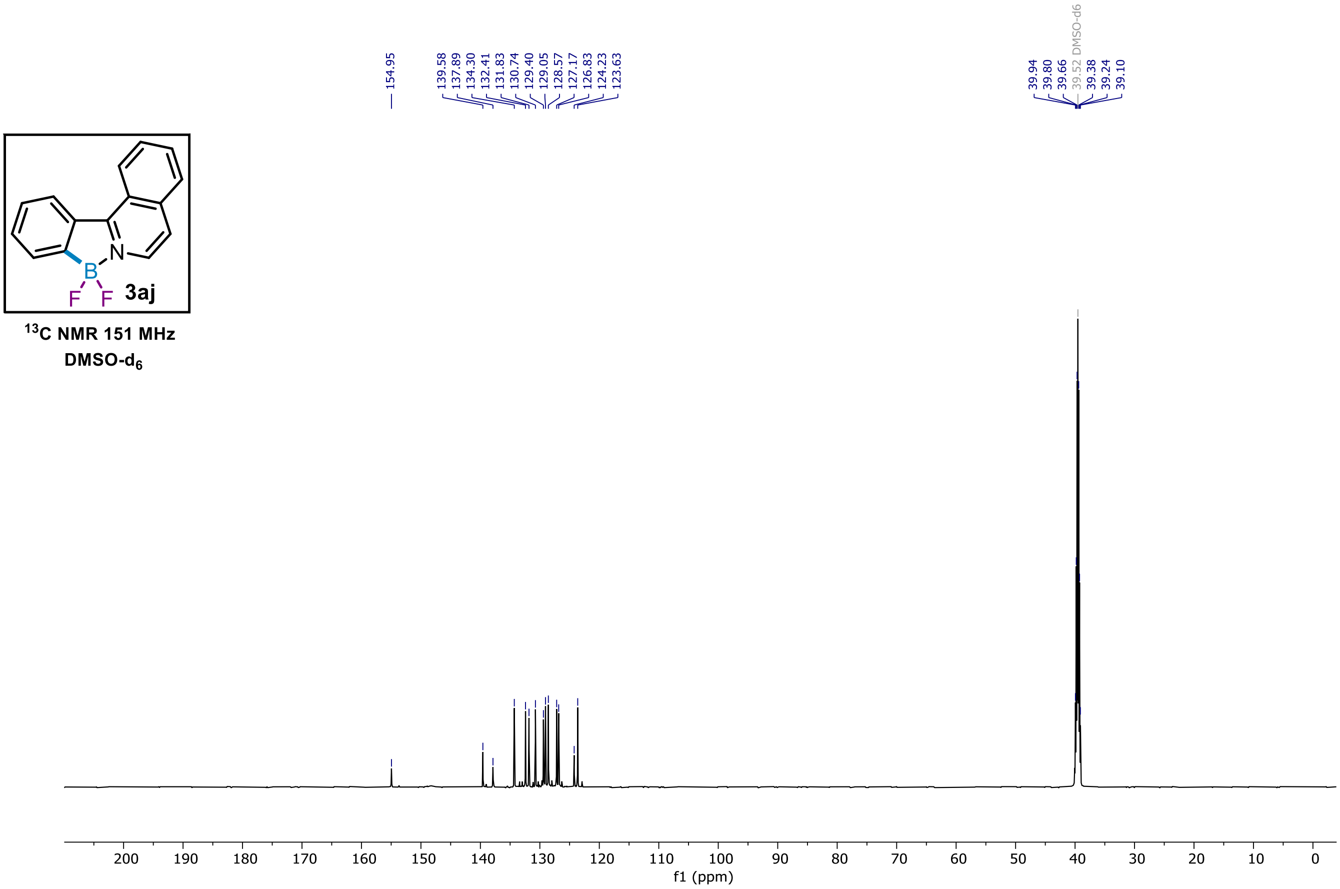

Figure S11-37: ^{13}C spectrum of compound **3aj** in DMSO-d$_6$. Note that the ^{13}C signal for the C-BF_2 bond does not appear.

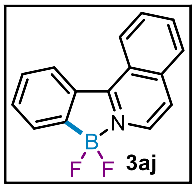

^{19}F NMR 659 MHz
DMSO-d_6

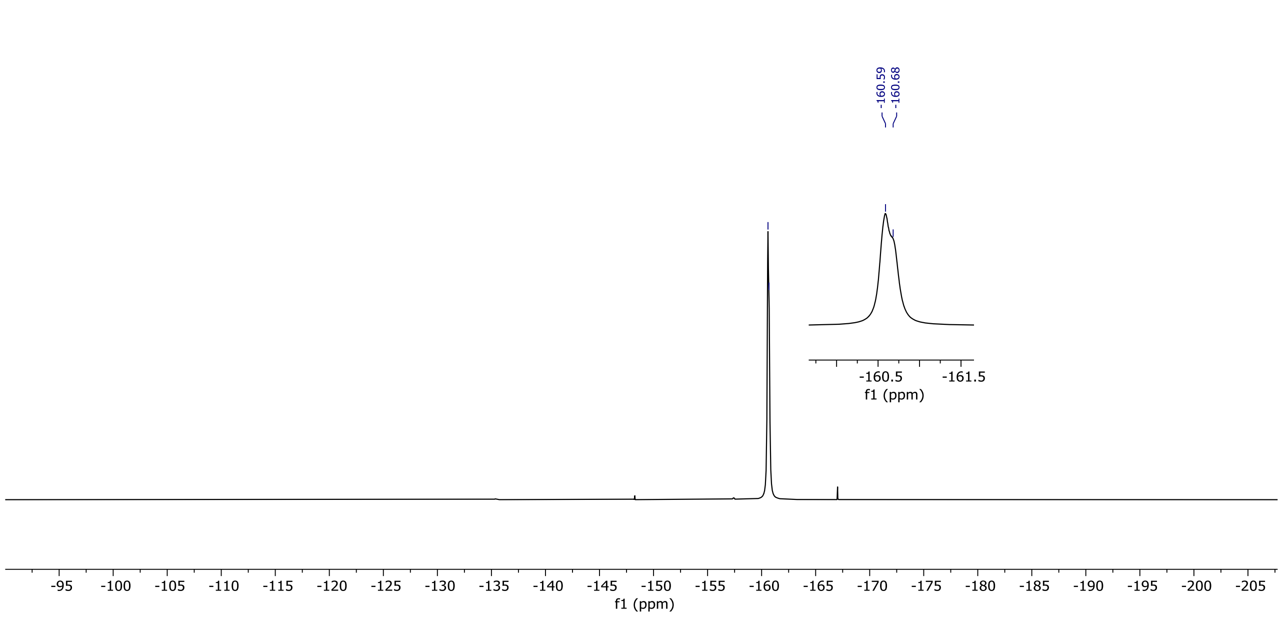

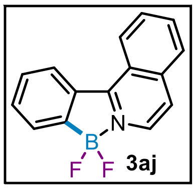

^{11}B NMR 193 MHz
DMSO-d_6

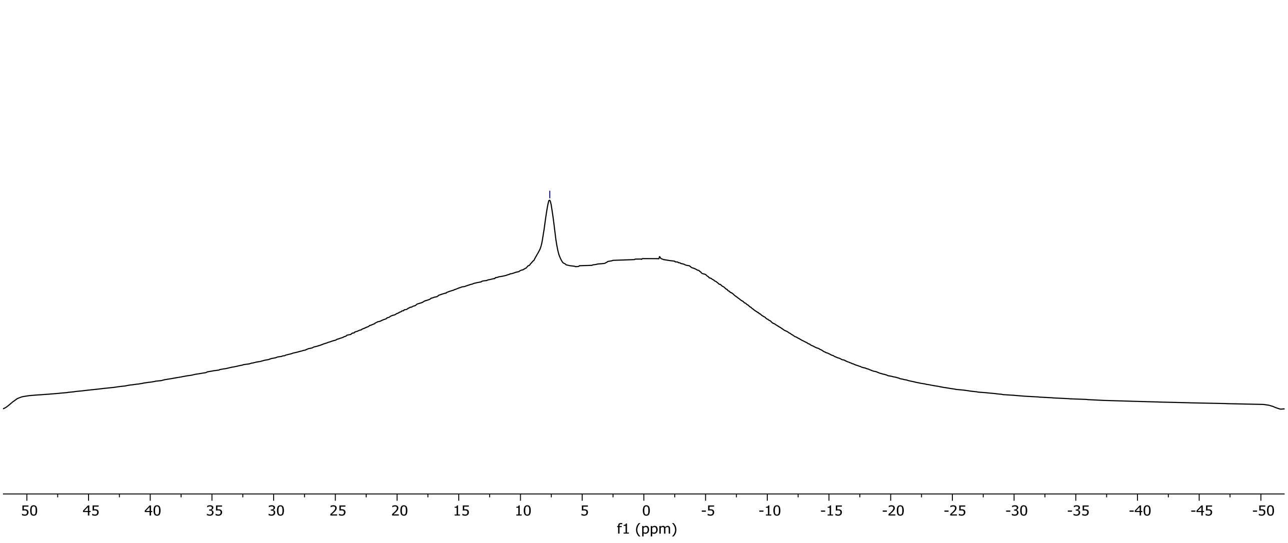

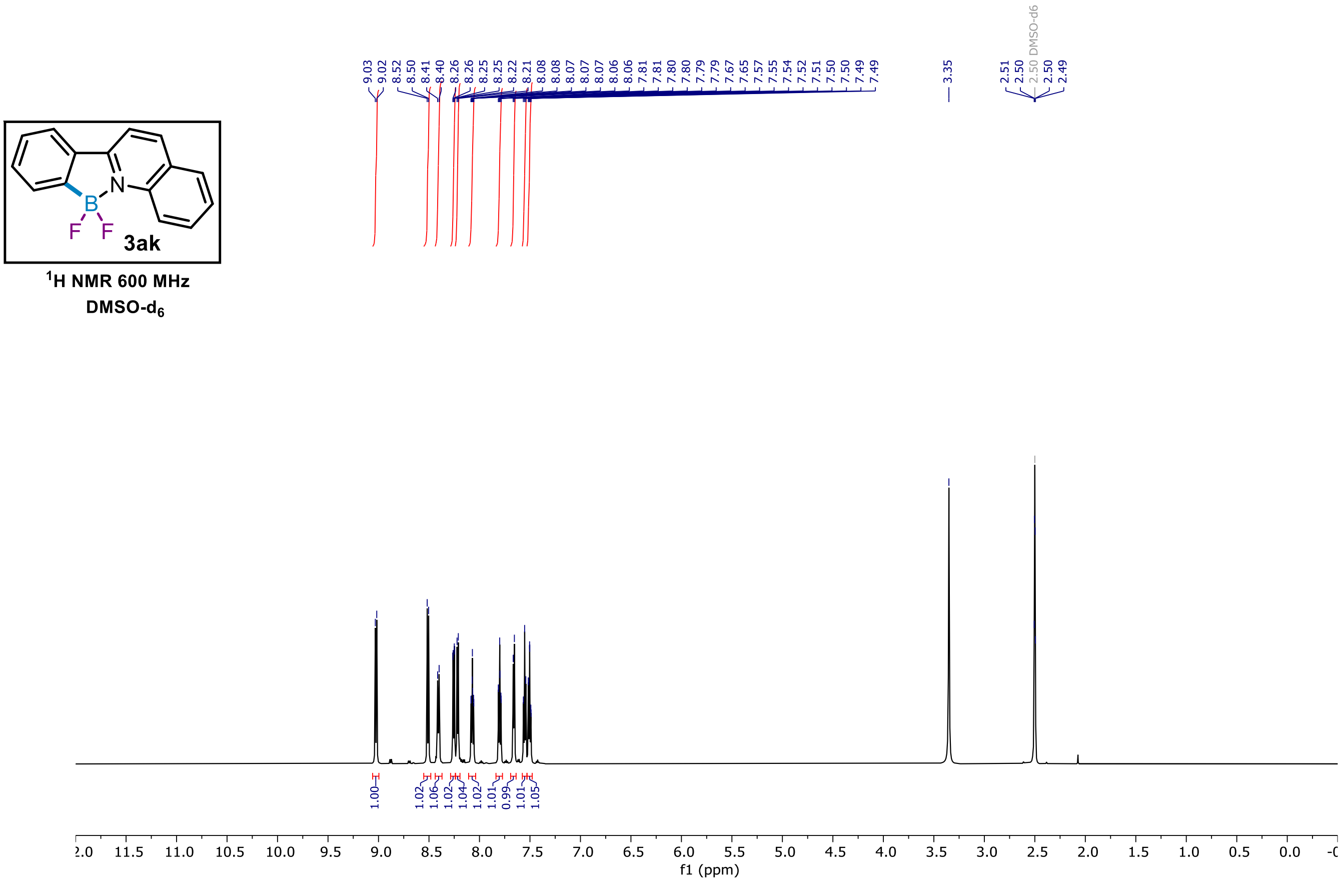

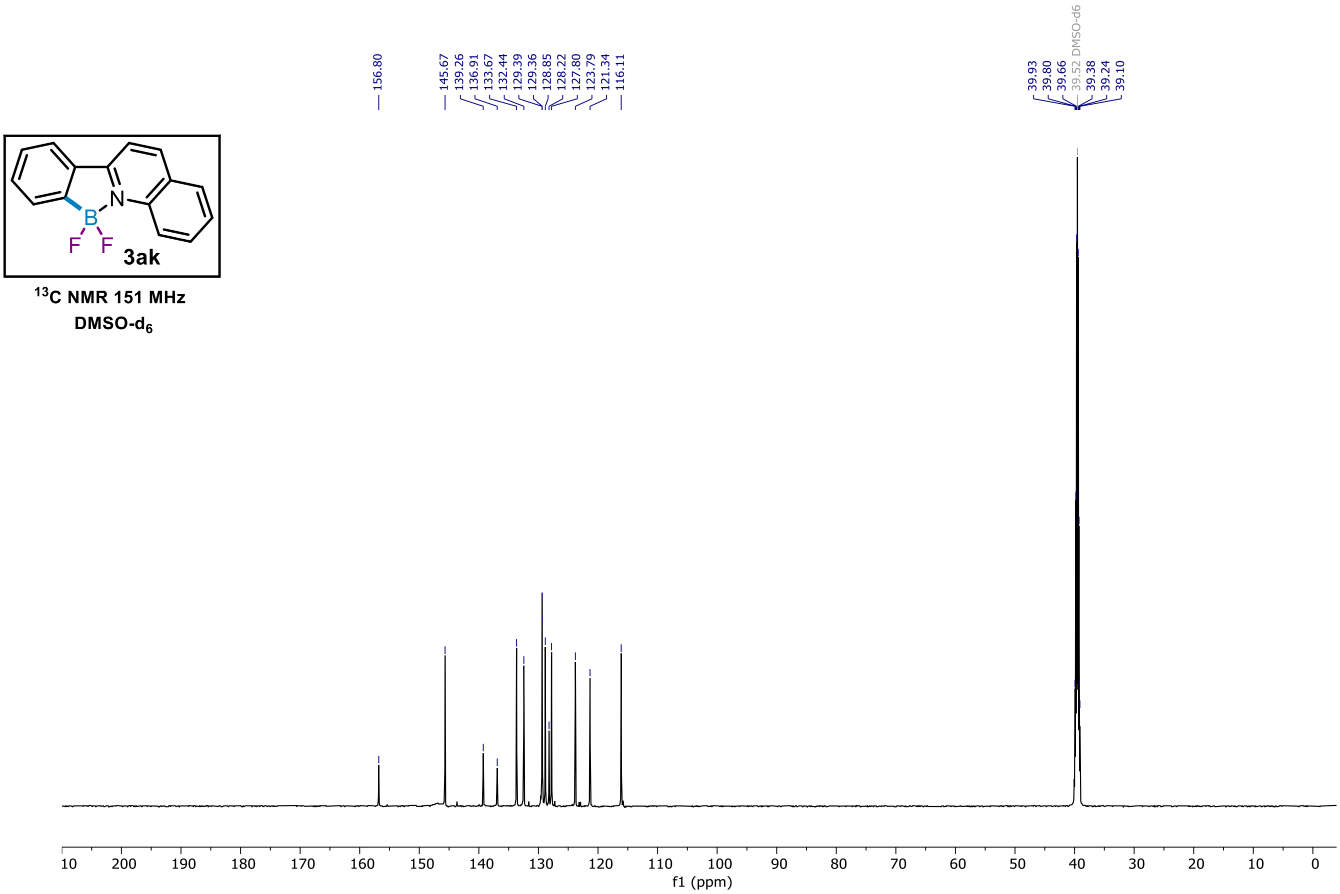

Figure S11-38: ^{13}C spectrum of compound **3ak** in DMSO-d$_6$. Note that the ^{13}C signal for the C-BF$_2$ bond does not appear.

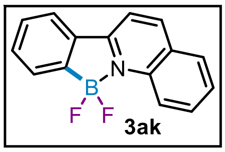

^{19}F NMR 659 MHz
DMSO-d_6

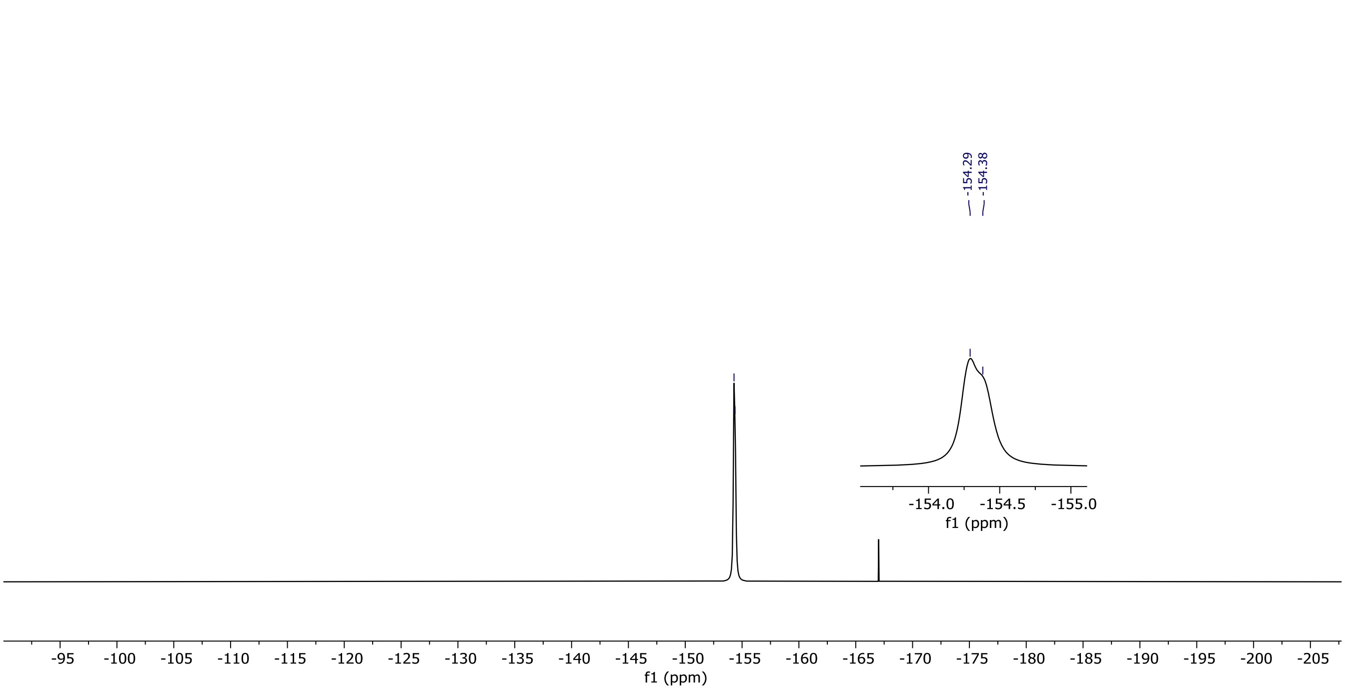

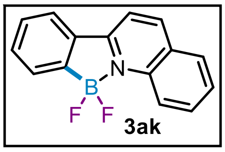

^{11}B NMR 193 MHz
DMSO-d_6

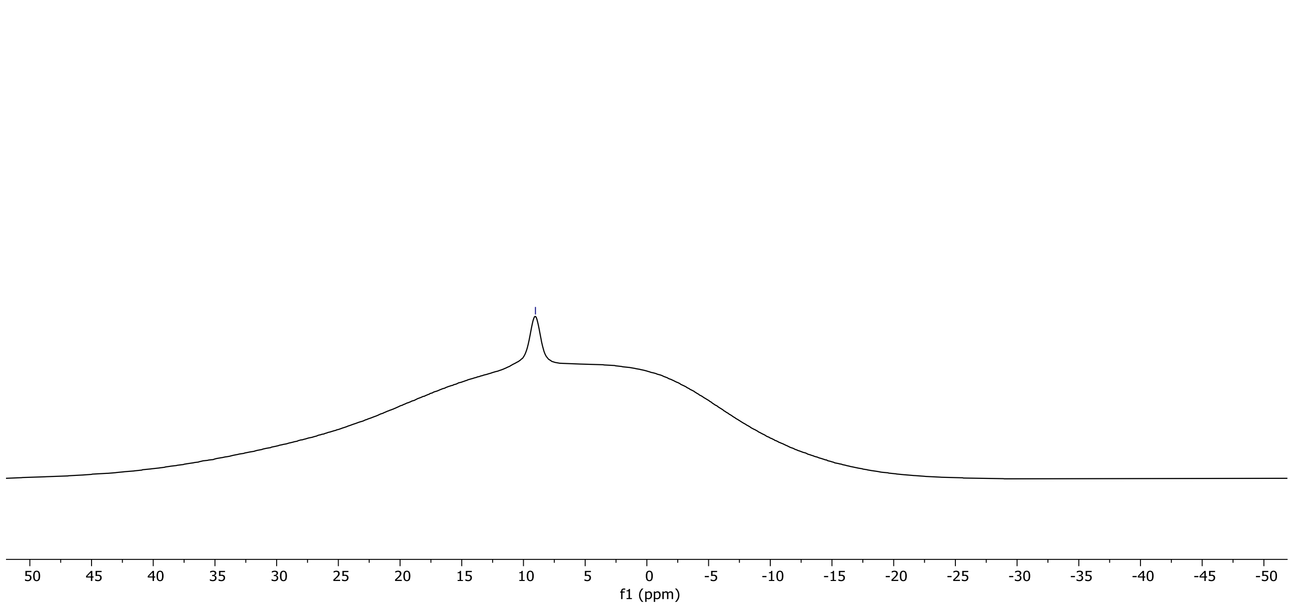

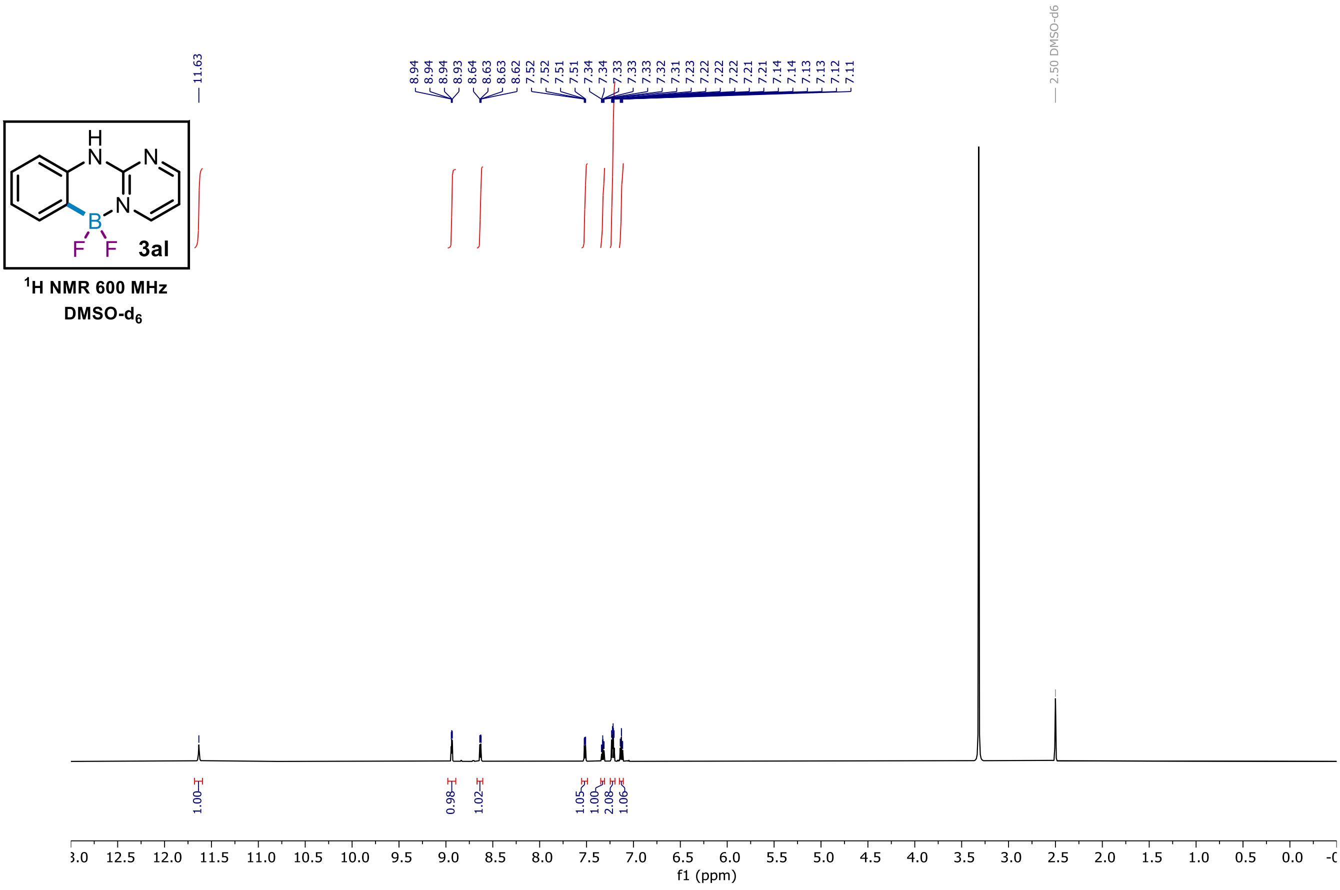

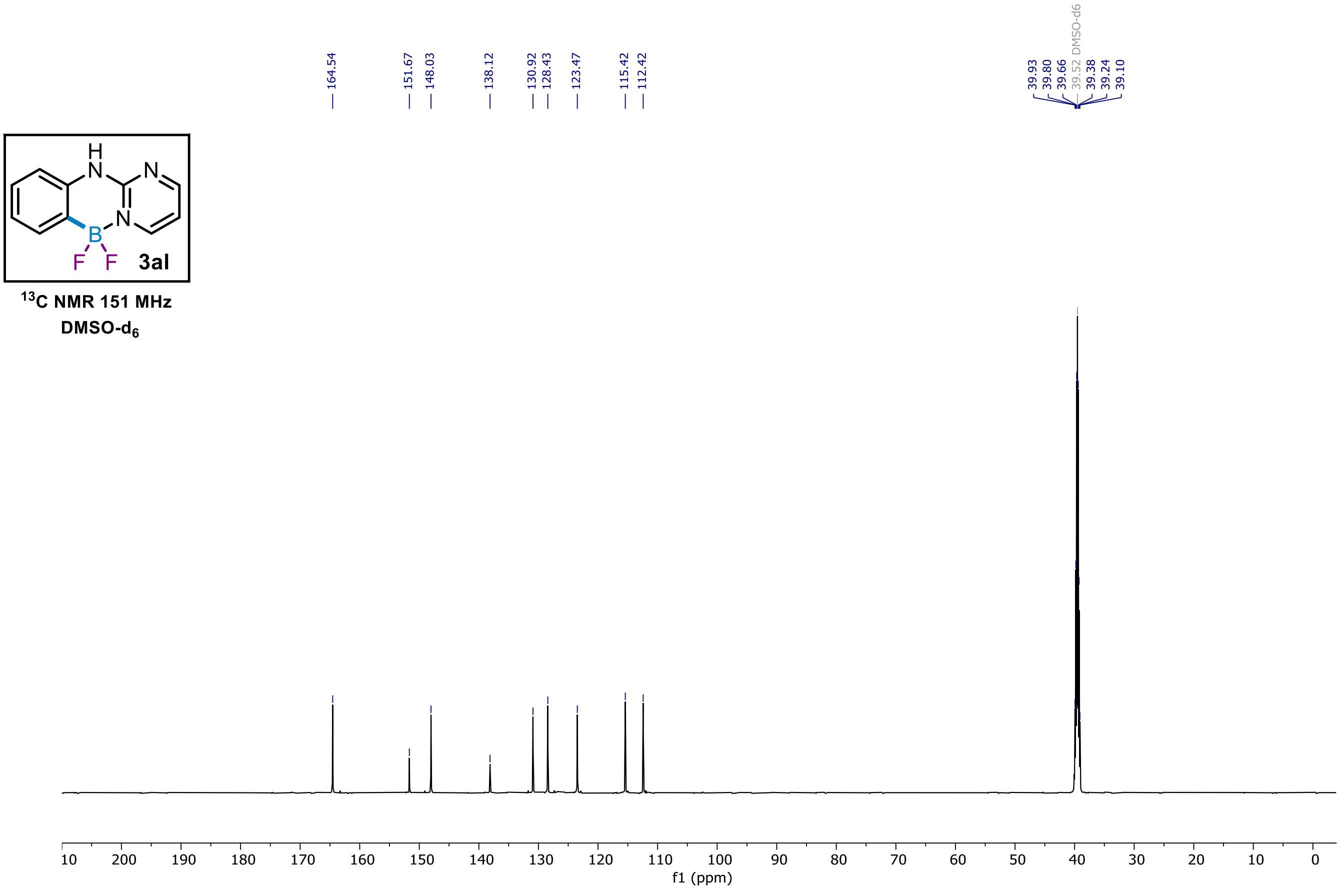

Figure S11-39: ^{13}C spectrum of compound **3al** in DMSO-d$_6$. Note that the ^{13}C signal for the C-BF_2 bond does not appear.

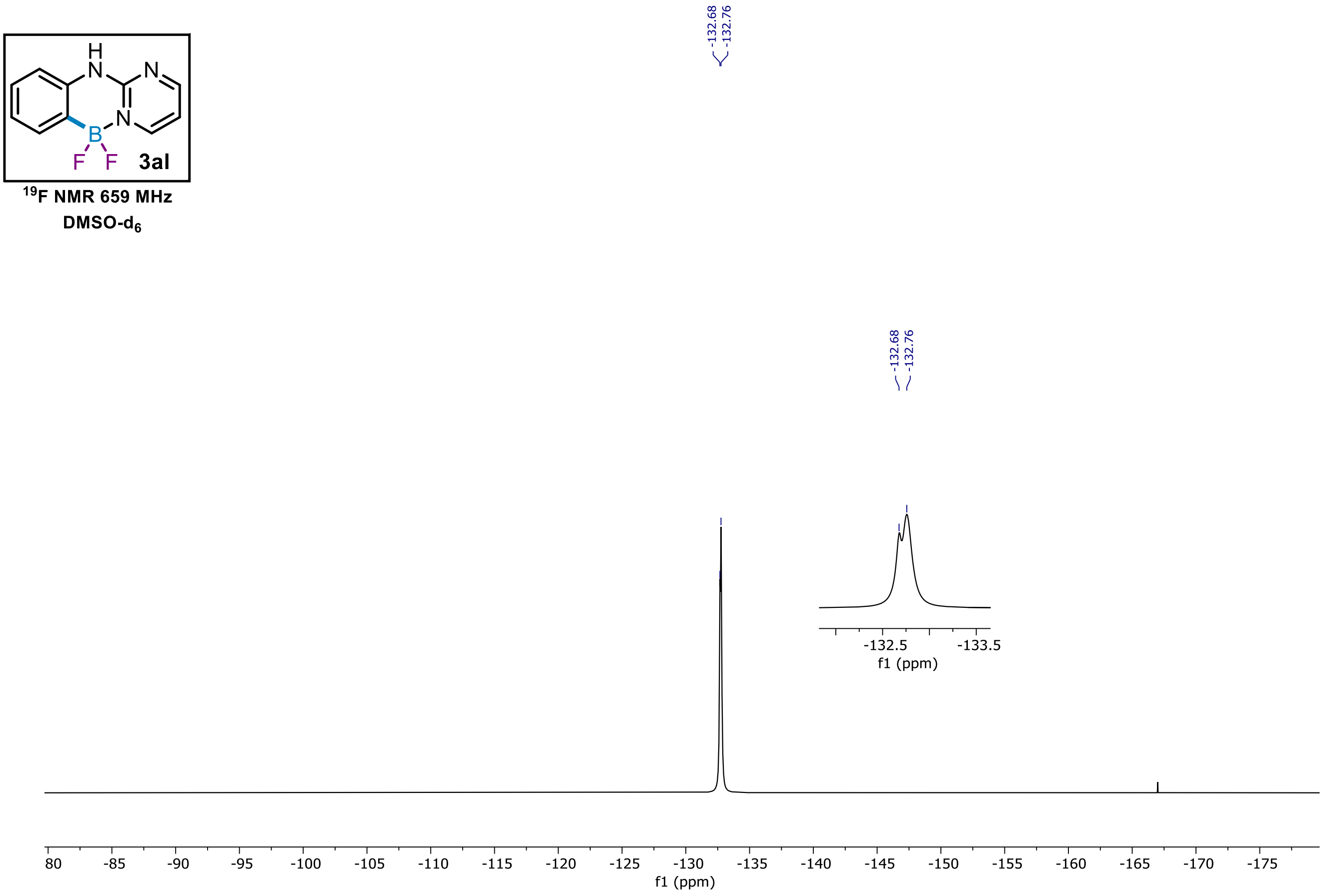
3al
^{19}F NMR 659 MHz
DMSO-d_6
-132.68
-132.76
-132.5
-133.5
f1 (ppm)
80 -85 -90 -95 -100 -105 -110 -115 -120 -125 -130 -135 -140 -145 -150 -155 -160 -165 -170 -175
f1 (ppm)

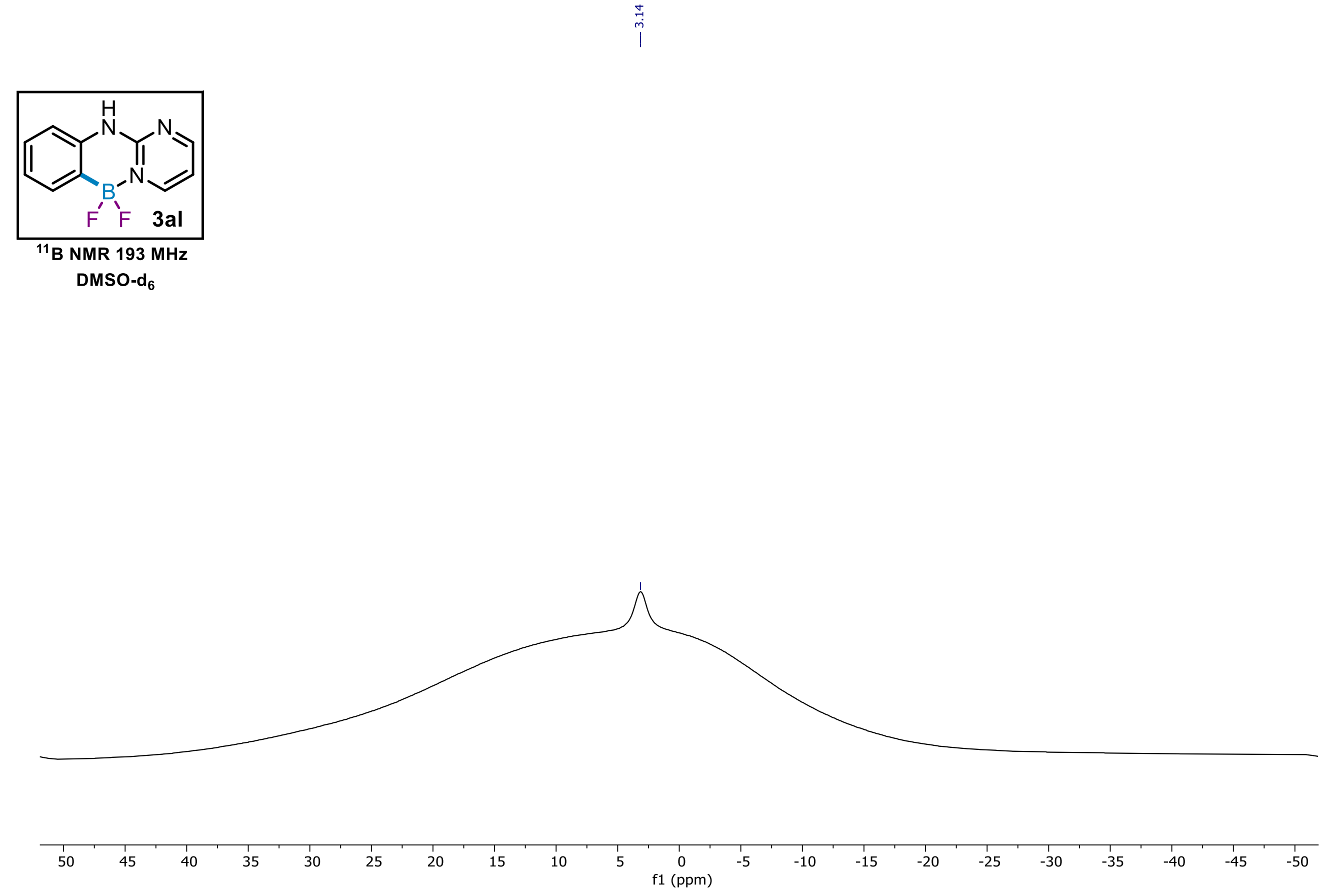
3.14
3al
^{11}B NMR 193 MHz
DMSO-d_6
50 45 40 35 30 25 20 15 10 5 0 -5 -10 -15 -20 -25 -30 -35 -40 -45 -50
f1 (ppm)

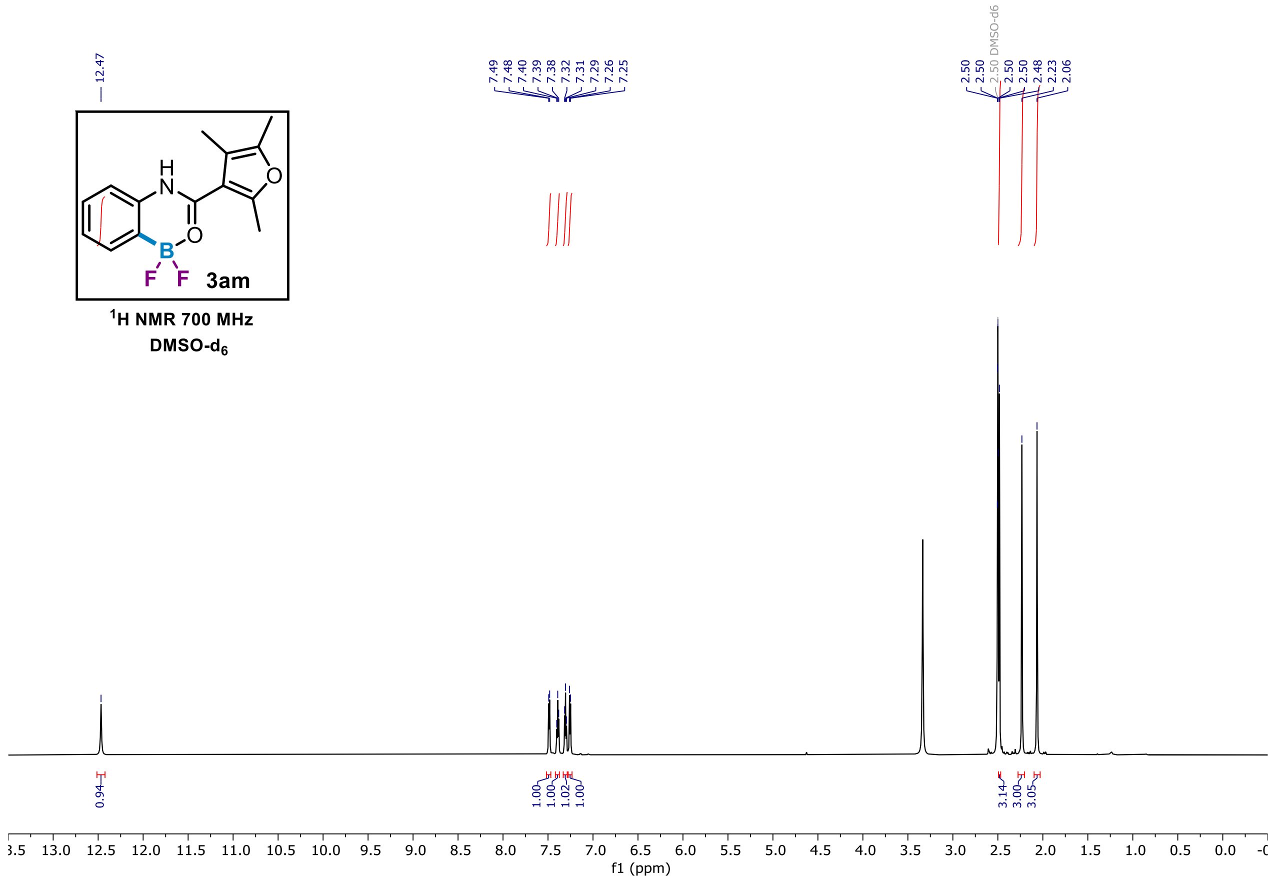

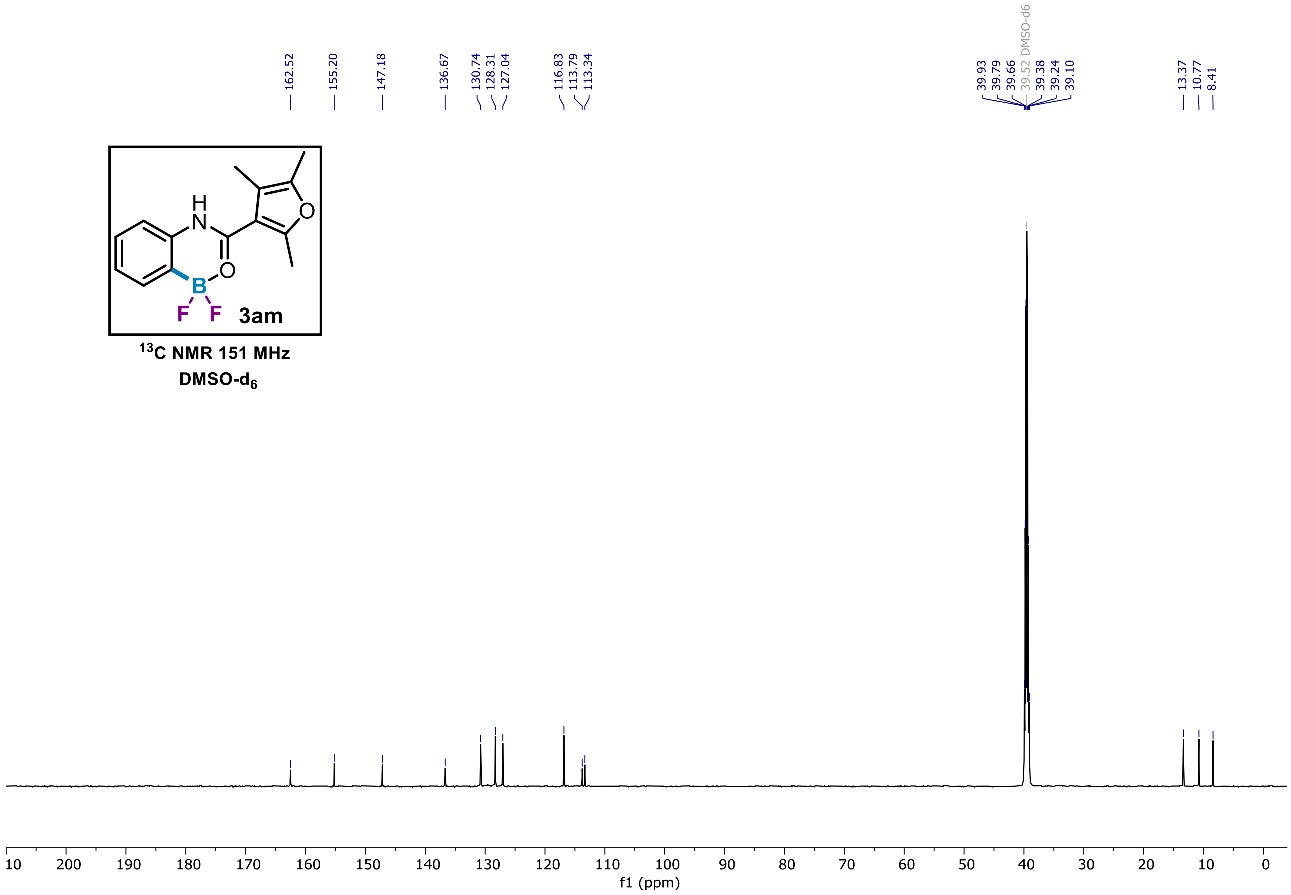

Figure S11-40: ^{13}C spectrum of compound **3am** in DMSO-d_6. Note that the ^{13}C signal for the C-BF_2 bond does not appear.

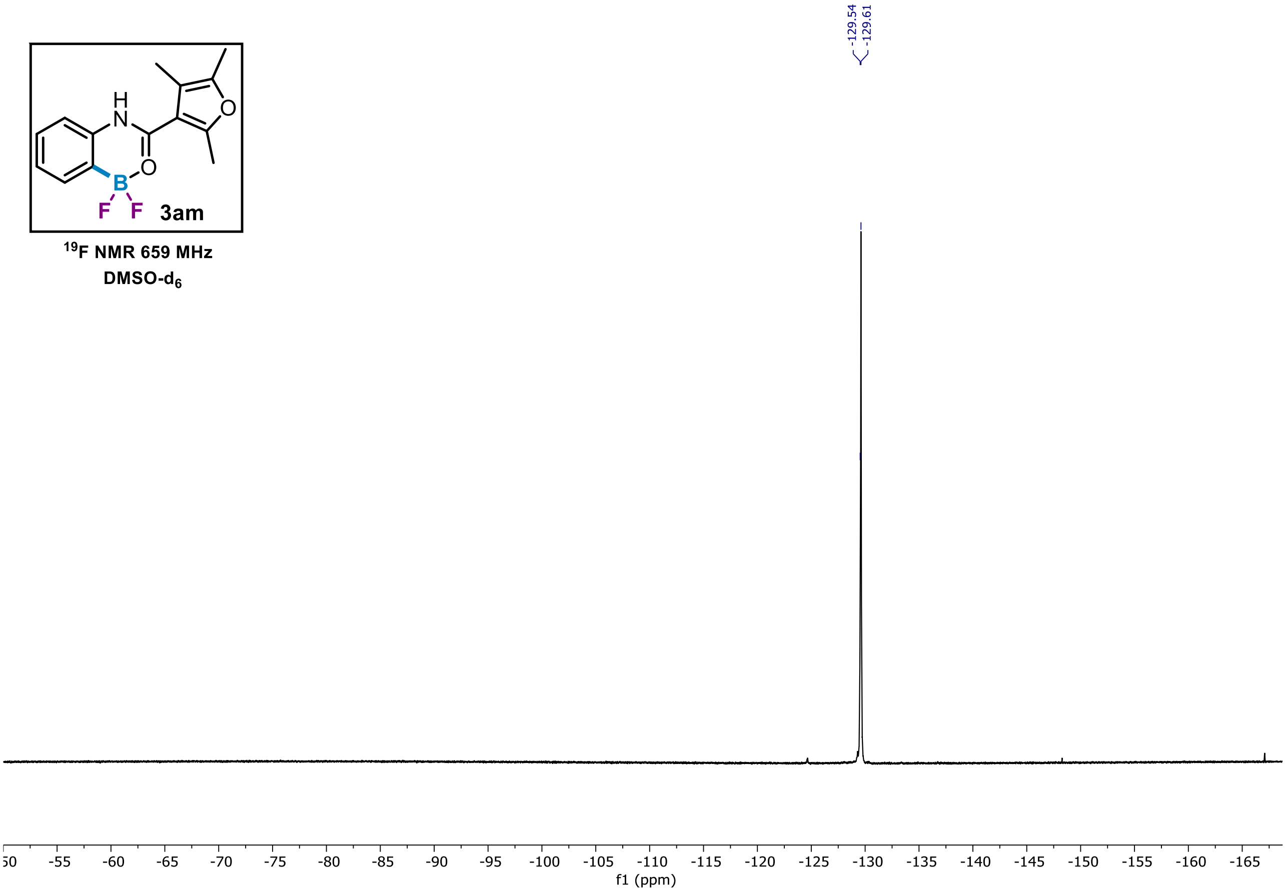

3am
^{19}F NMR 659 MHz
DMSO-d_6
-129.54
-129.61
f1 (ppm)

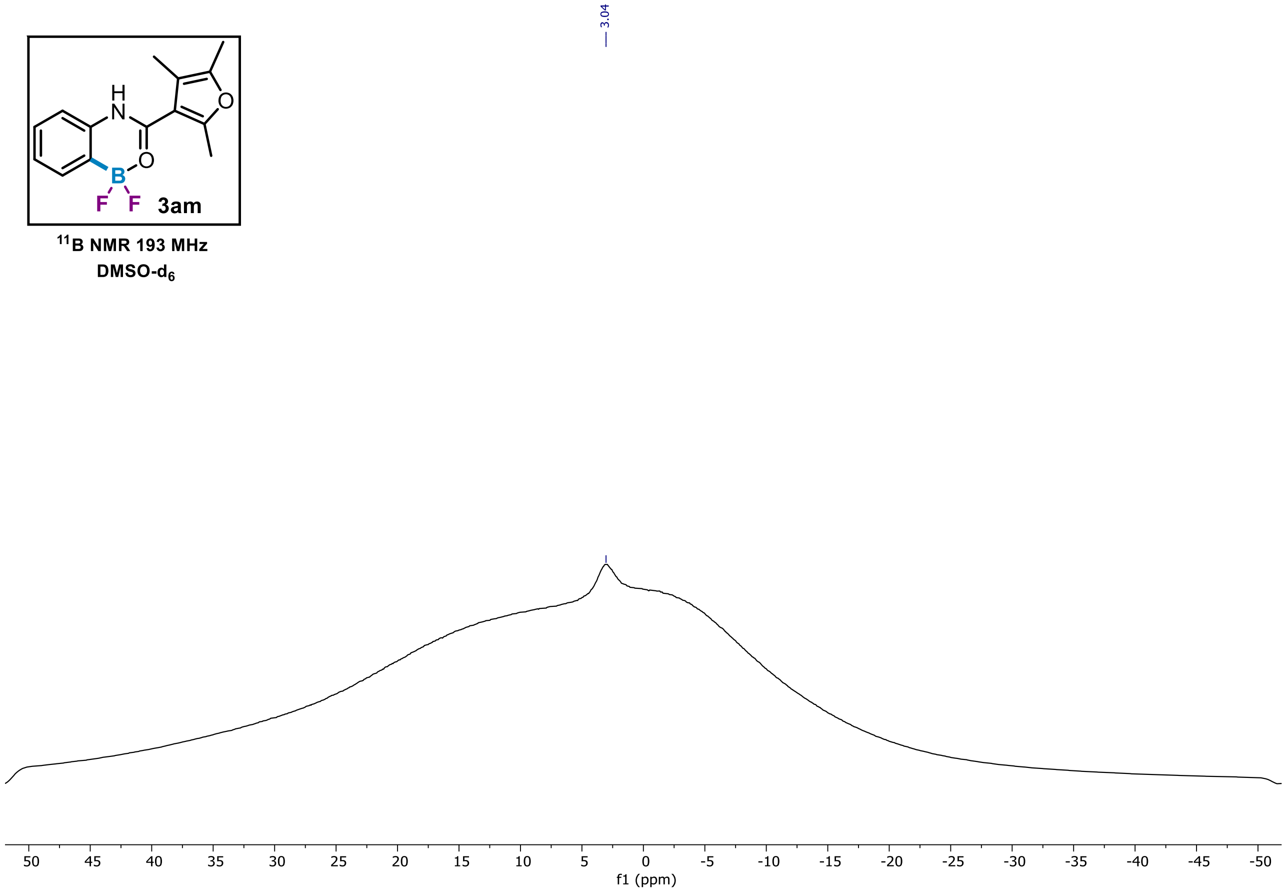

3am
^{11}B NMR 193 MHz
DMSO-d_6
3.04
f1 (ppm)

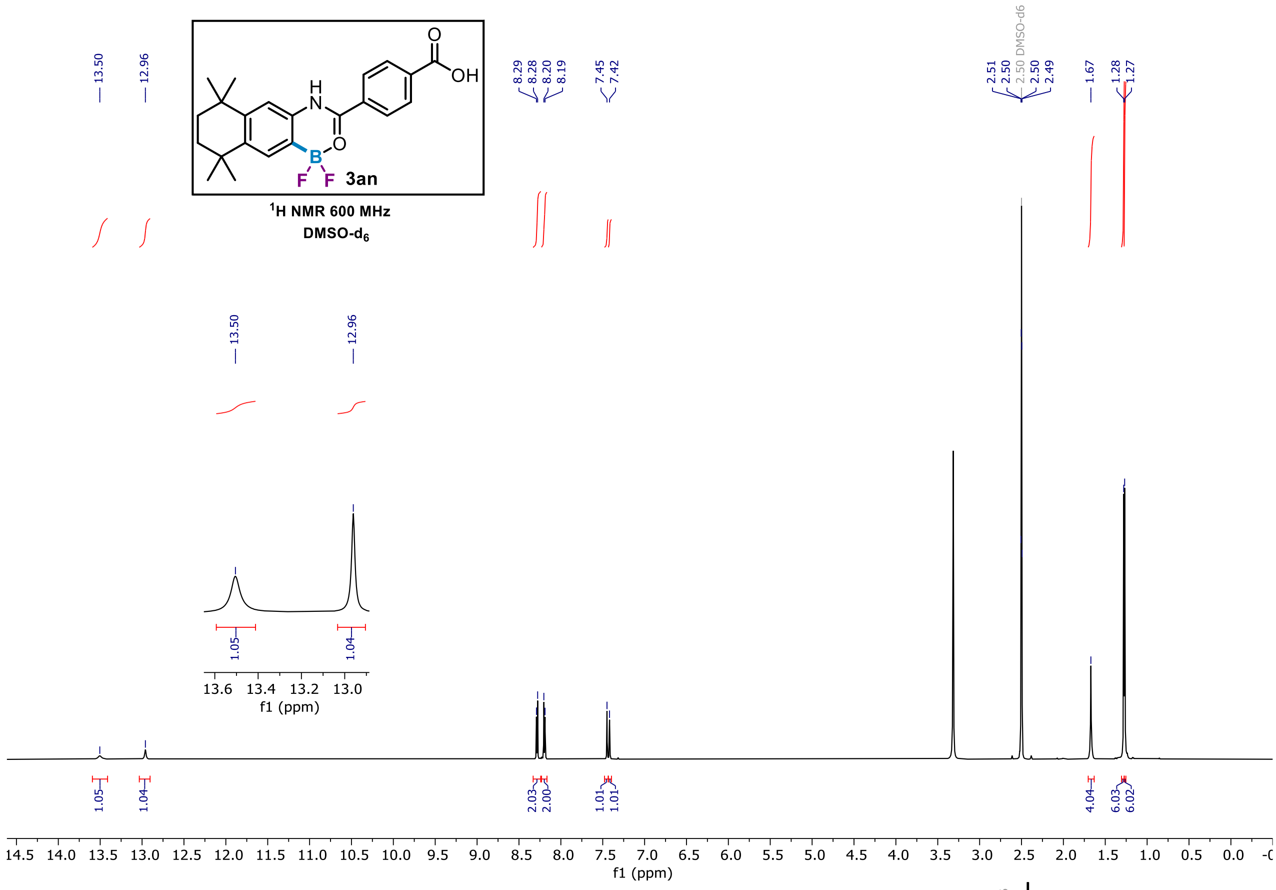

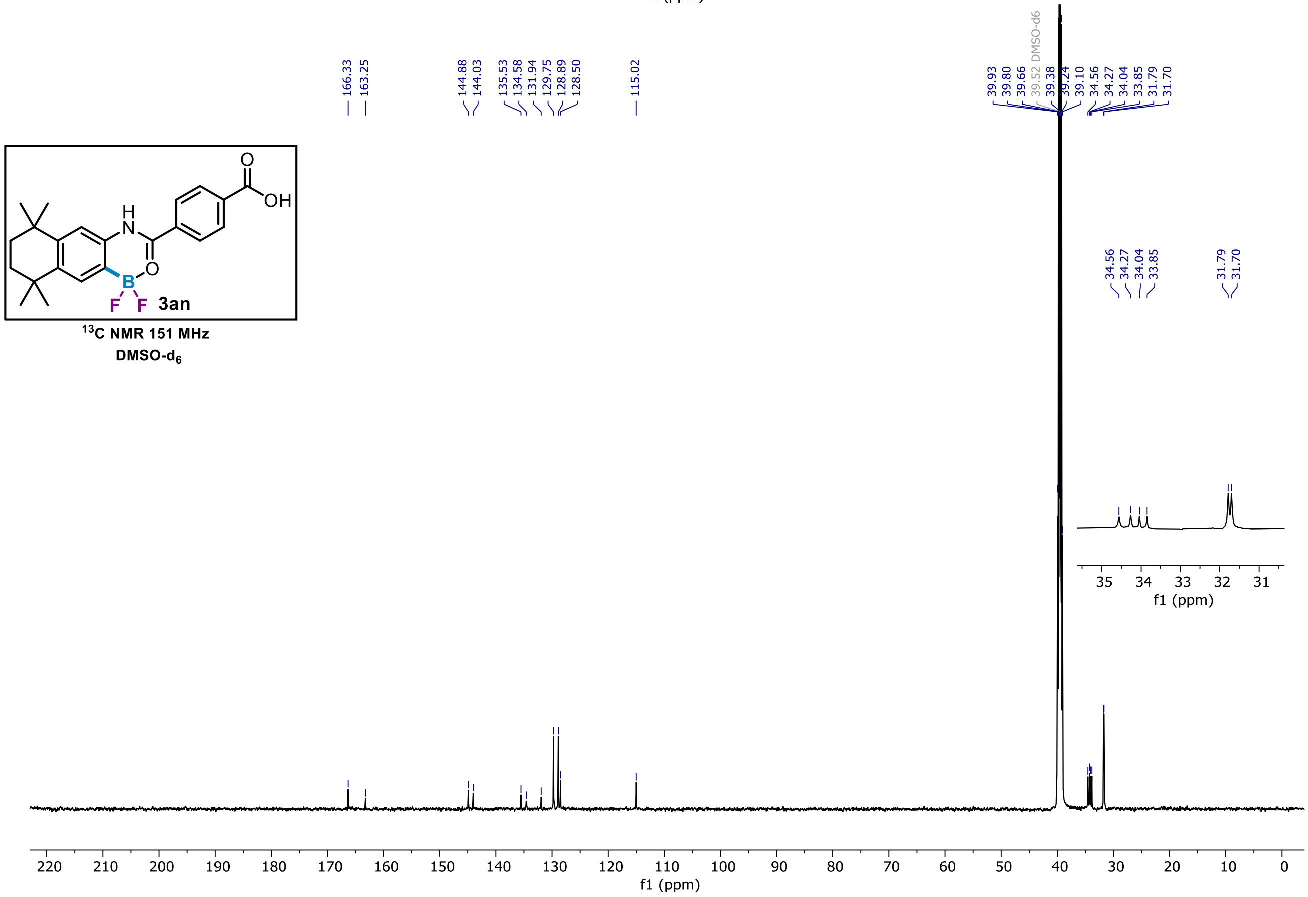

Figure S11-41: ^{13}C spectrum of compound **3an** in DMSO-d_6. Note that the ^{13}C signal for the C-BF_2 bond does not appear.

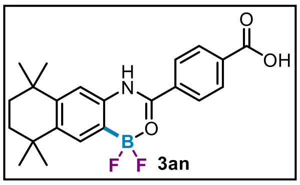

^{19}F NMR 659 MHz
DMSO-d_6

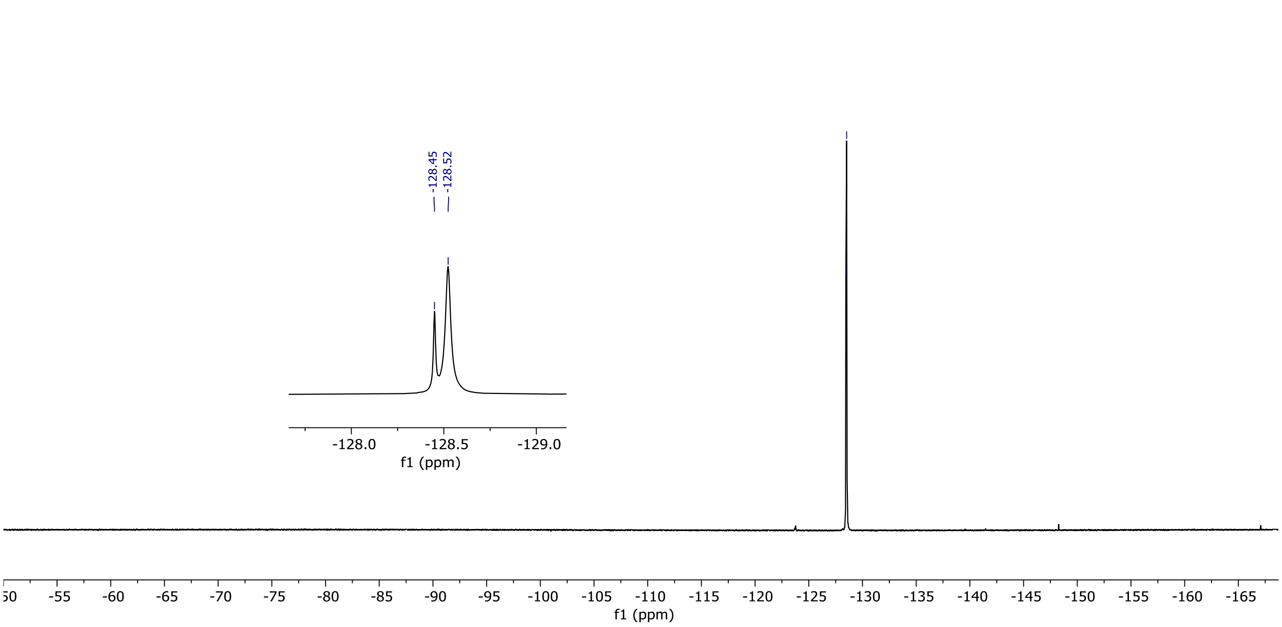

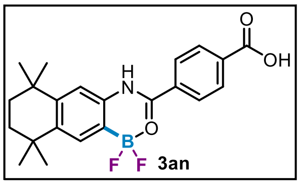

^{11}B NMR 193 MHz
DMSO-d_6

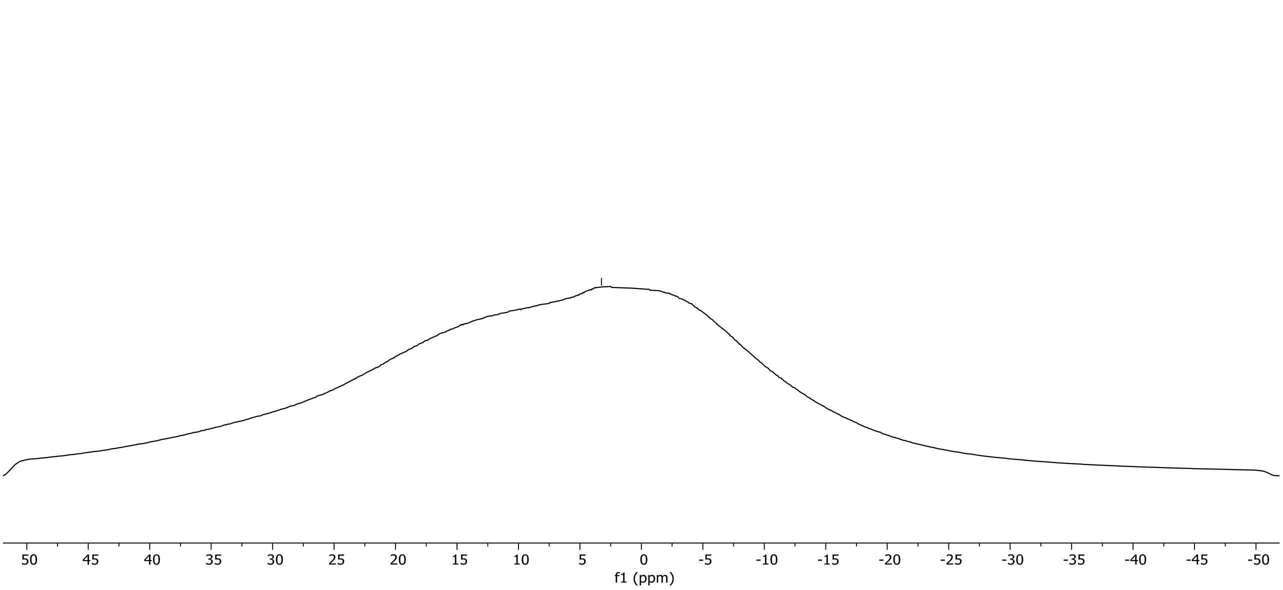

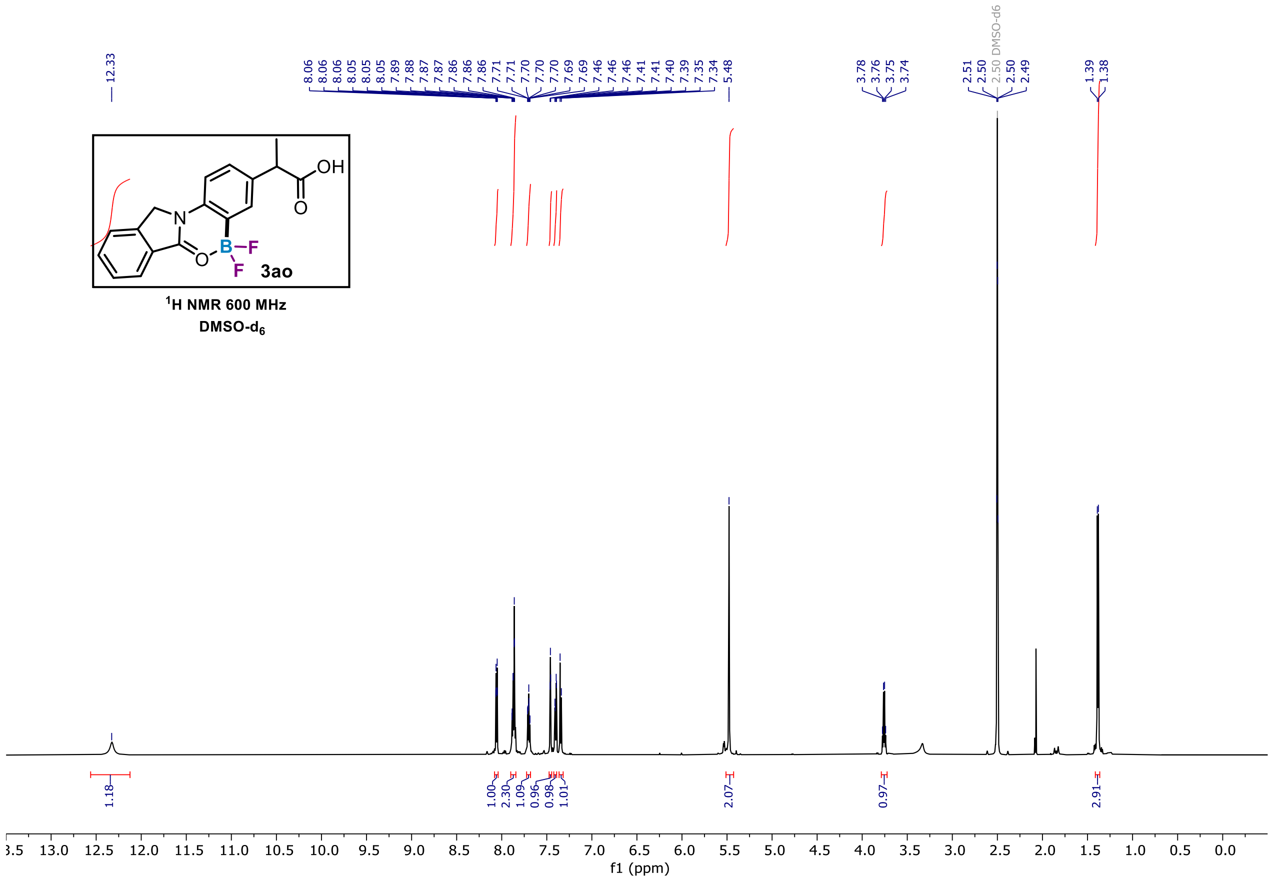

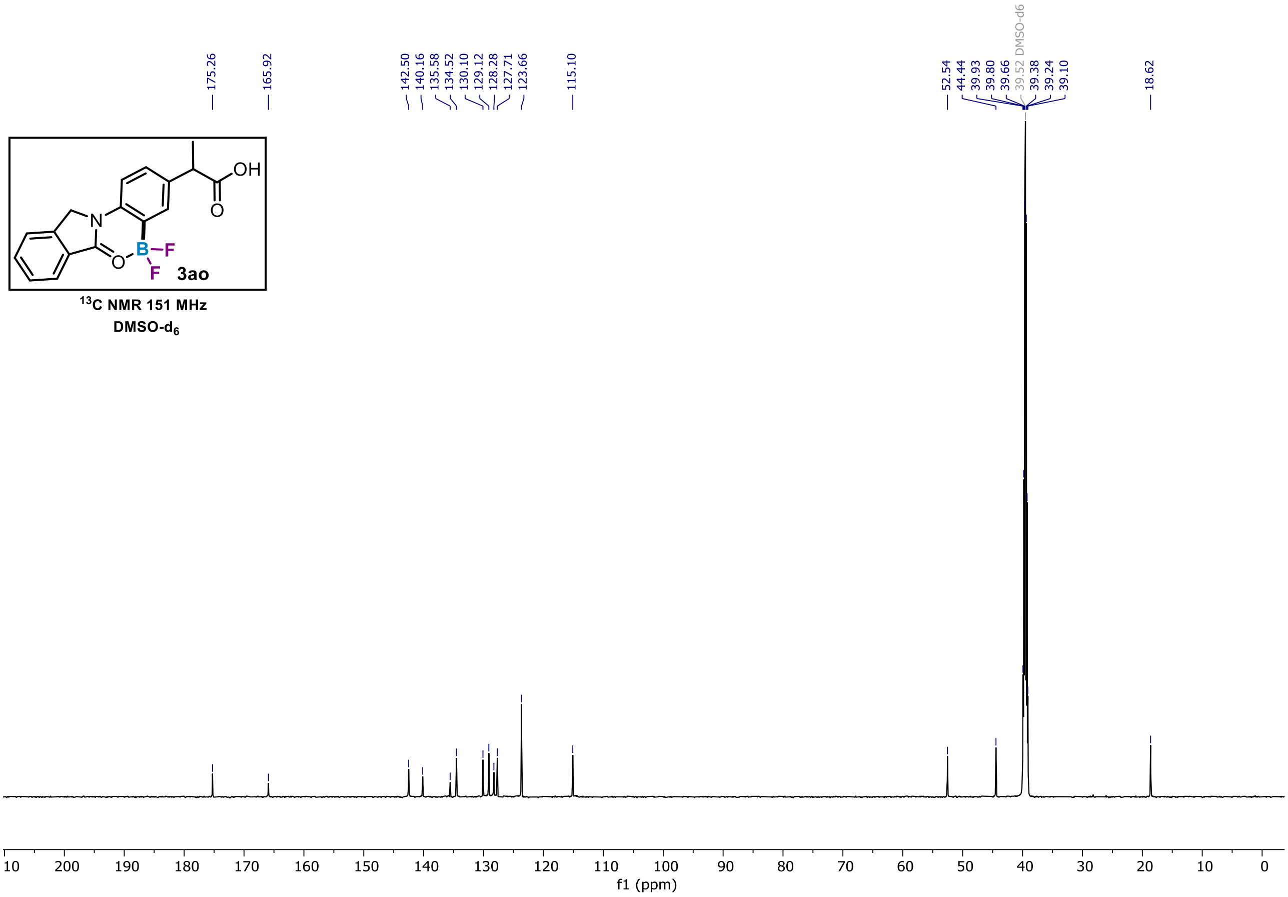

Figure S11-42: ^{13}C spectrum of compound **3ao** in DMSO-d$_6$. Note that the ^{13}C signal for the C-BF$_2$ bond does not appear.

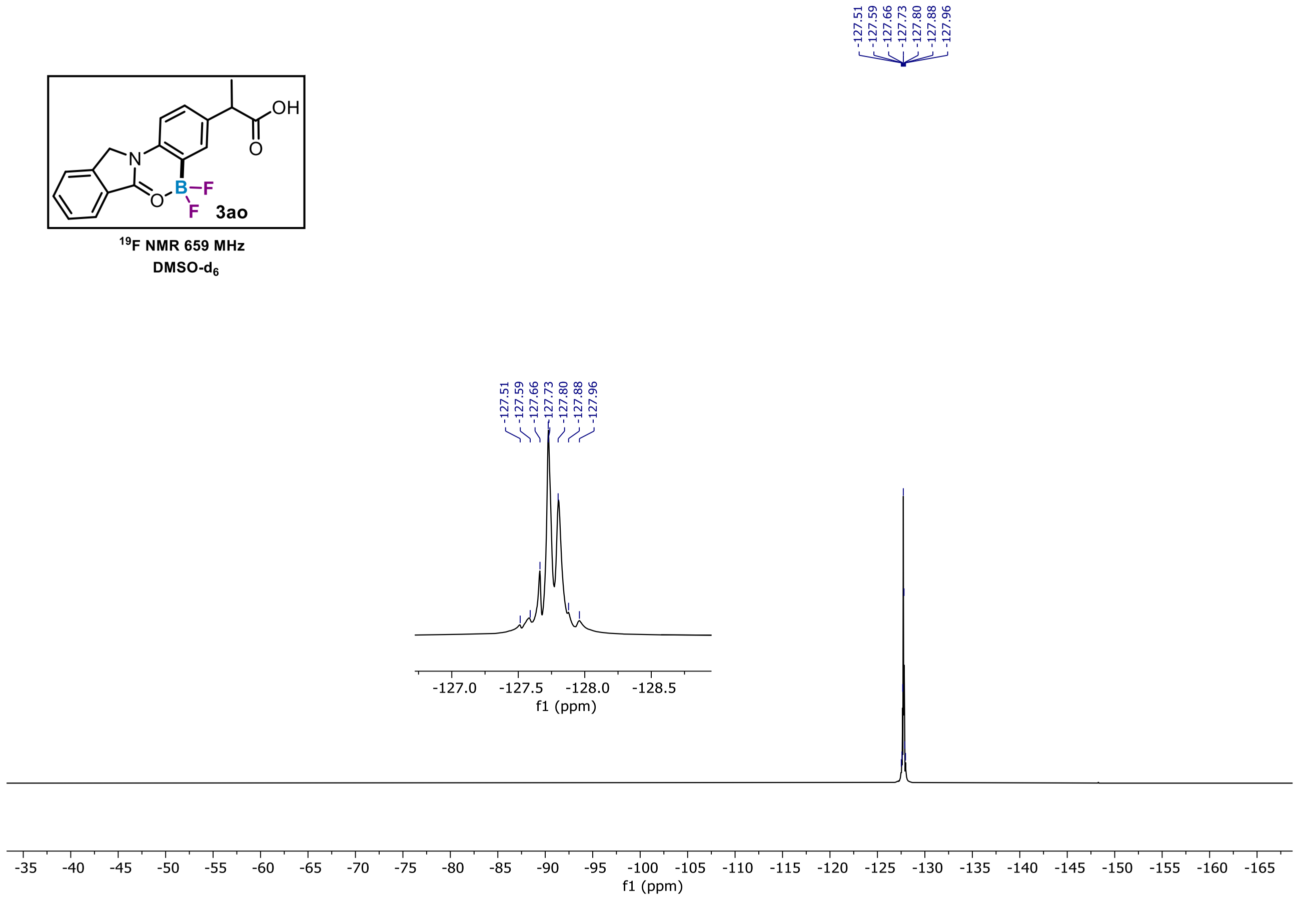
-127.51
-127.59
-127.66
-127.73
-127.80
-127.88
-127.96
OH
O
N
B
F
F
O
3ao
^{19}F NMR 659 MHz
DMSO-d6
-127.0
-127.5
-128.0
-128.5
f1 (ppm)
-35 -40 -45 -50 -55 -60 -65 -70 -75 -80 -85 -90 -95 -100 -105 -110 -115 -120 -125 -130 -135 -140 -145 -150 -155 -160 -165
f1 (ppm)

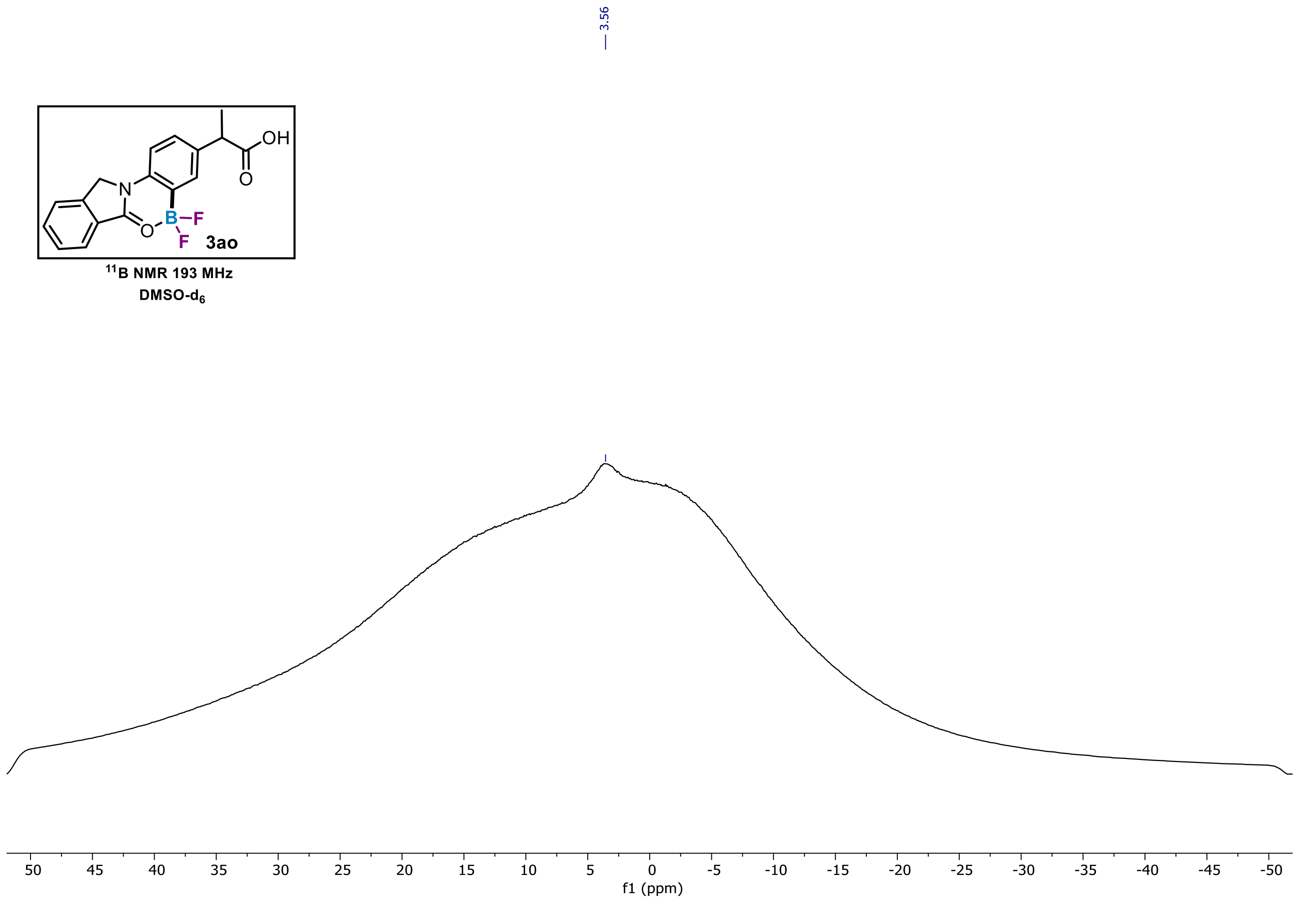
3.56
OH
O
N
B
F
F
O
3ao
^{11}B NMR 193 MHz
DMSO-d6
50 45 40 35 30 25 20 15 10 5 0 -5 -10 -15 -20 -25 -30 -35 -40 -45 -50
f1 (ppm)

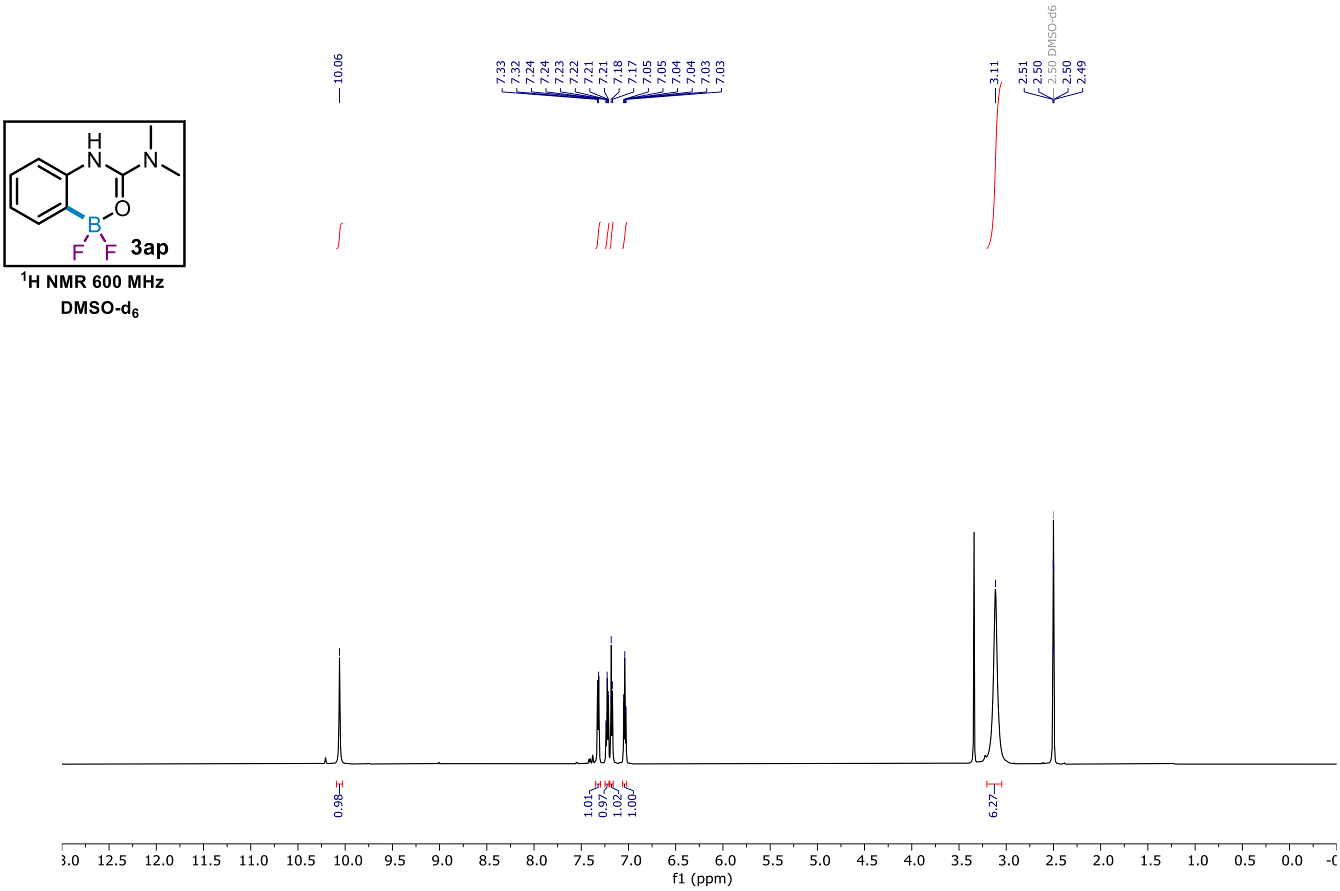

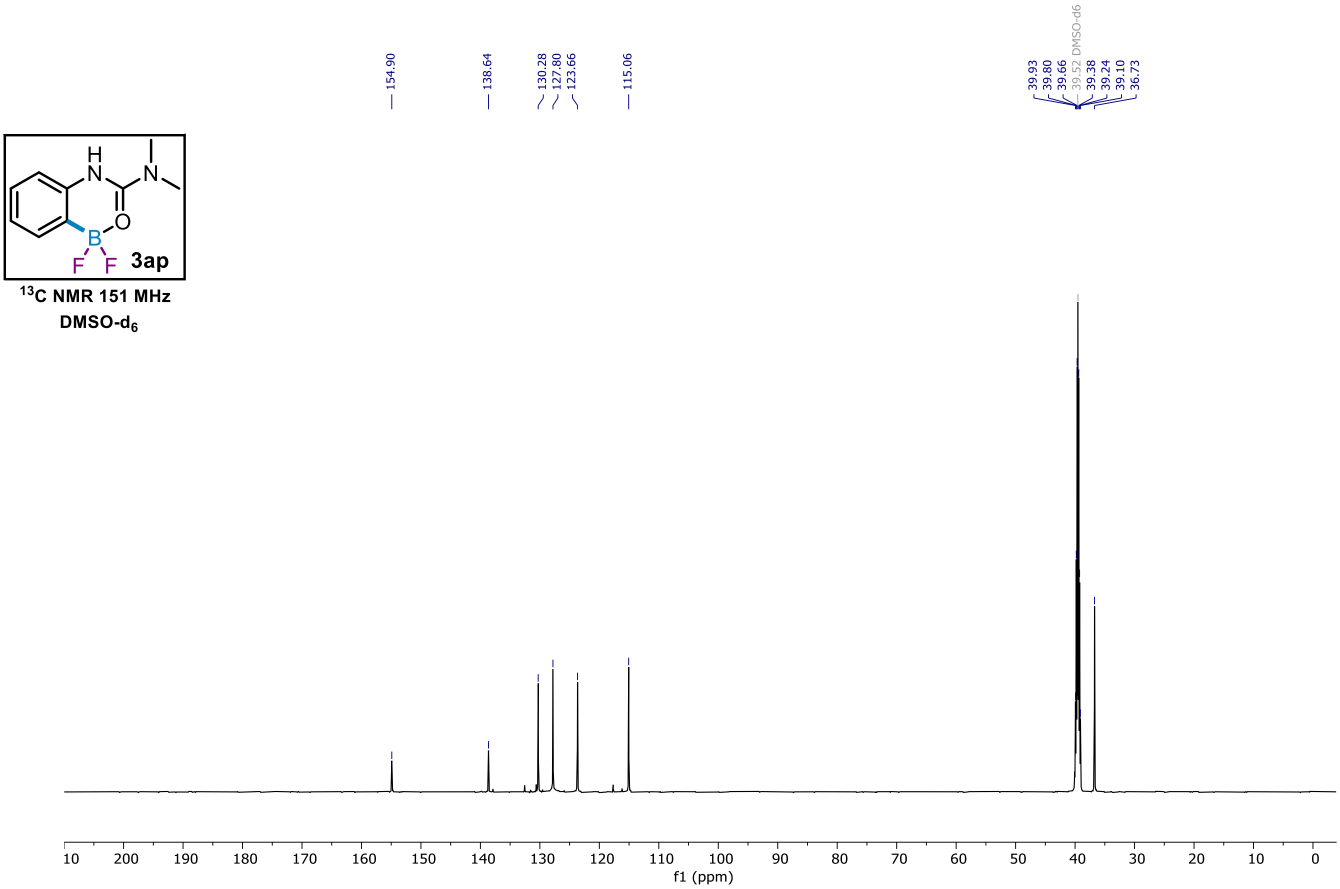

Figure S11-43: ^{13}C spectrum of compound **3ap** in DMSO-d_6. Note that the ^{13}C signal for the C-BF_2 bond does not appear.

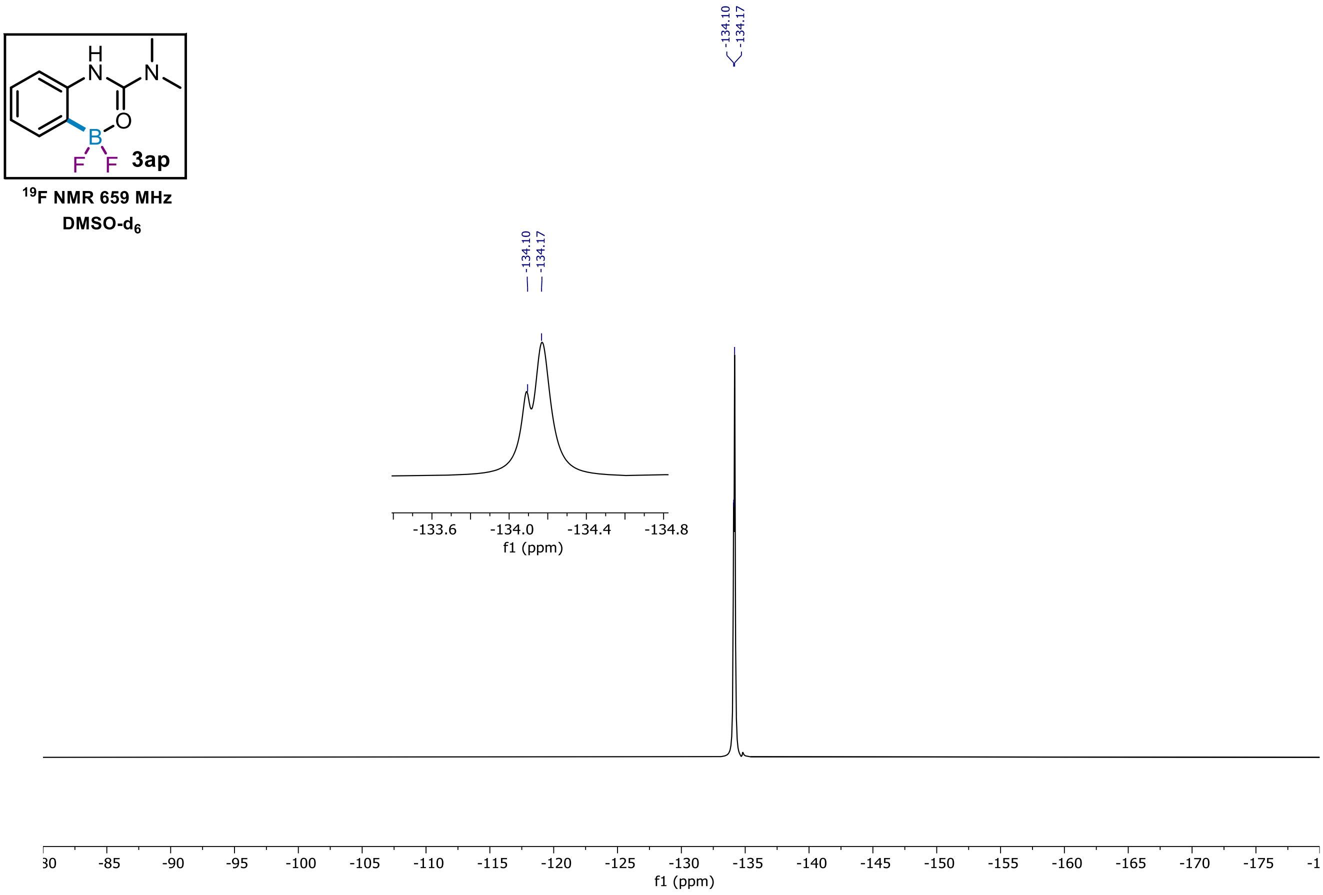
3ap
19F NMR 659 MHz
DMSO-d6
-134.10
-134.17
f1 (ppm)

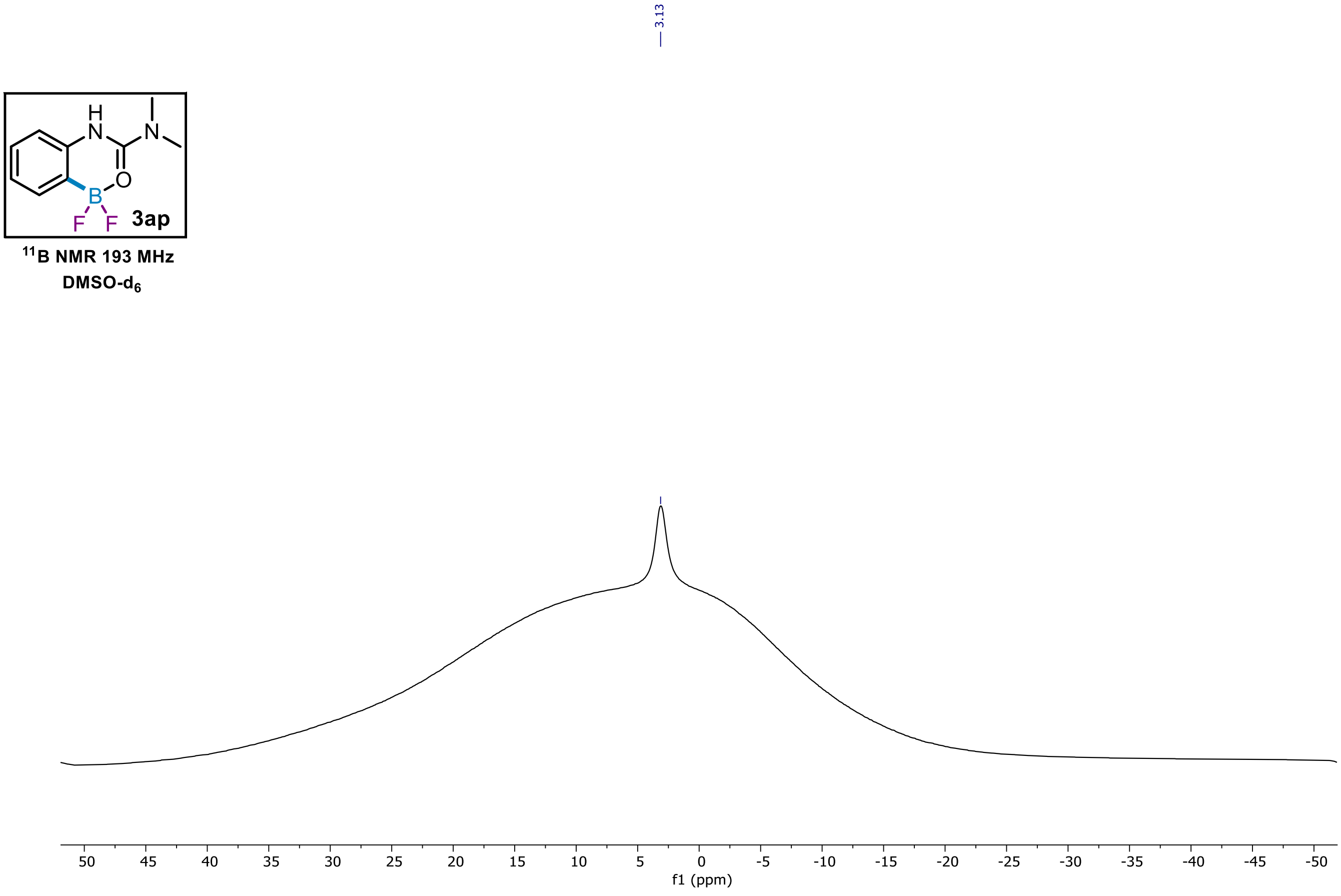
3ap
11B NMR 193 MHz
DMSO-d6
3.13
f1 (ppm)

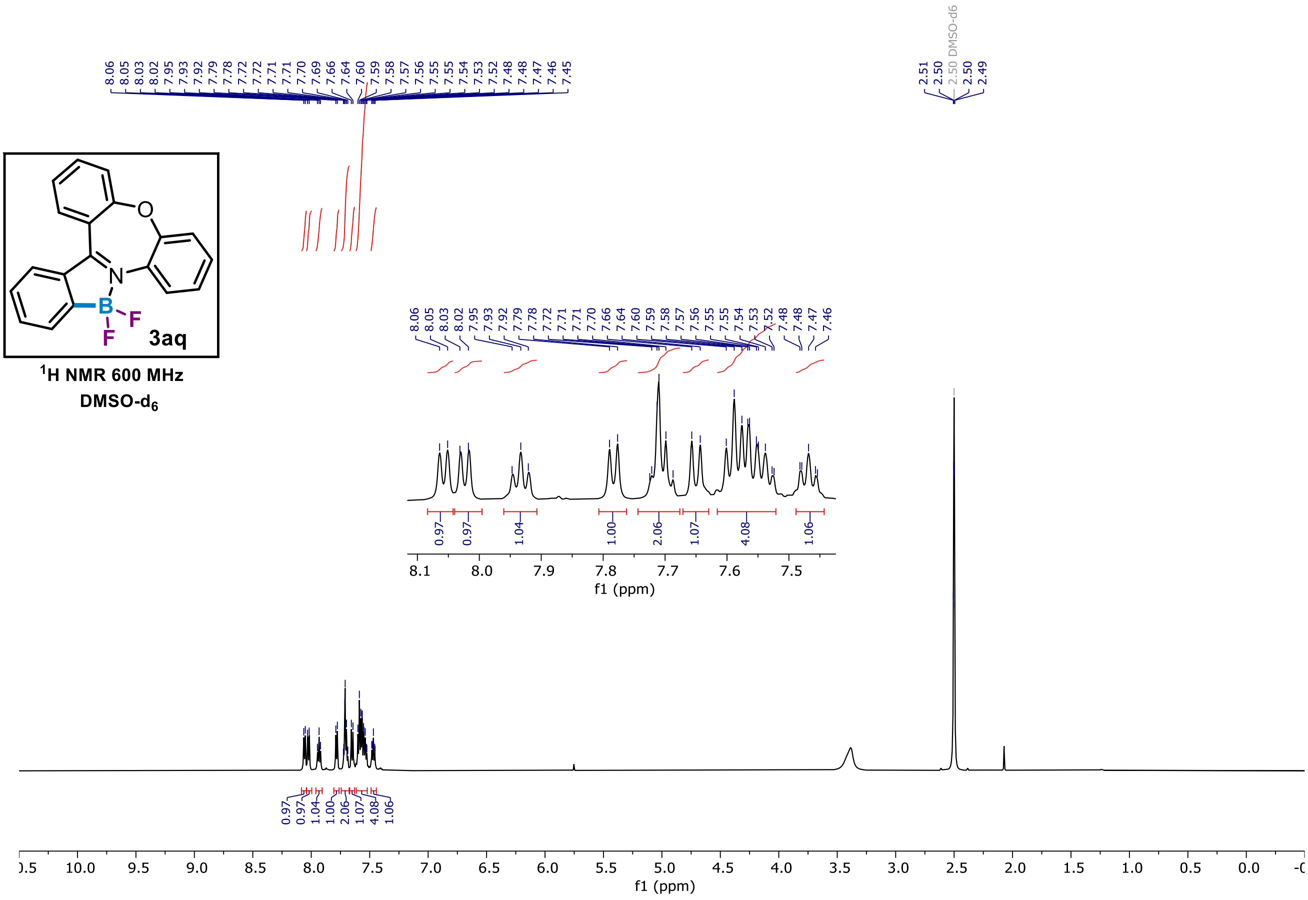

3aq

^{1}H NMR 600 MHz
DMSO-d_6

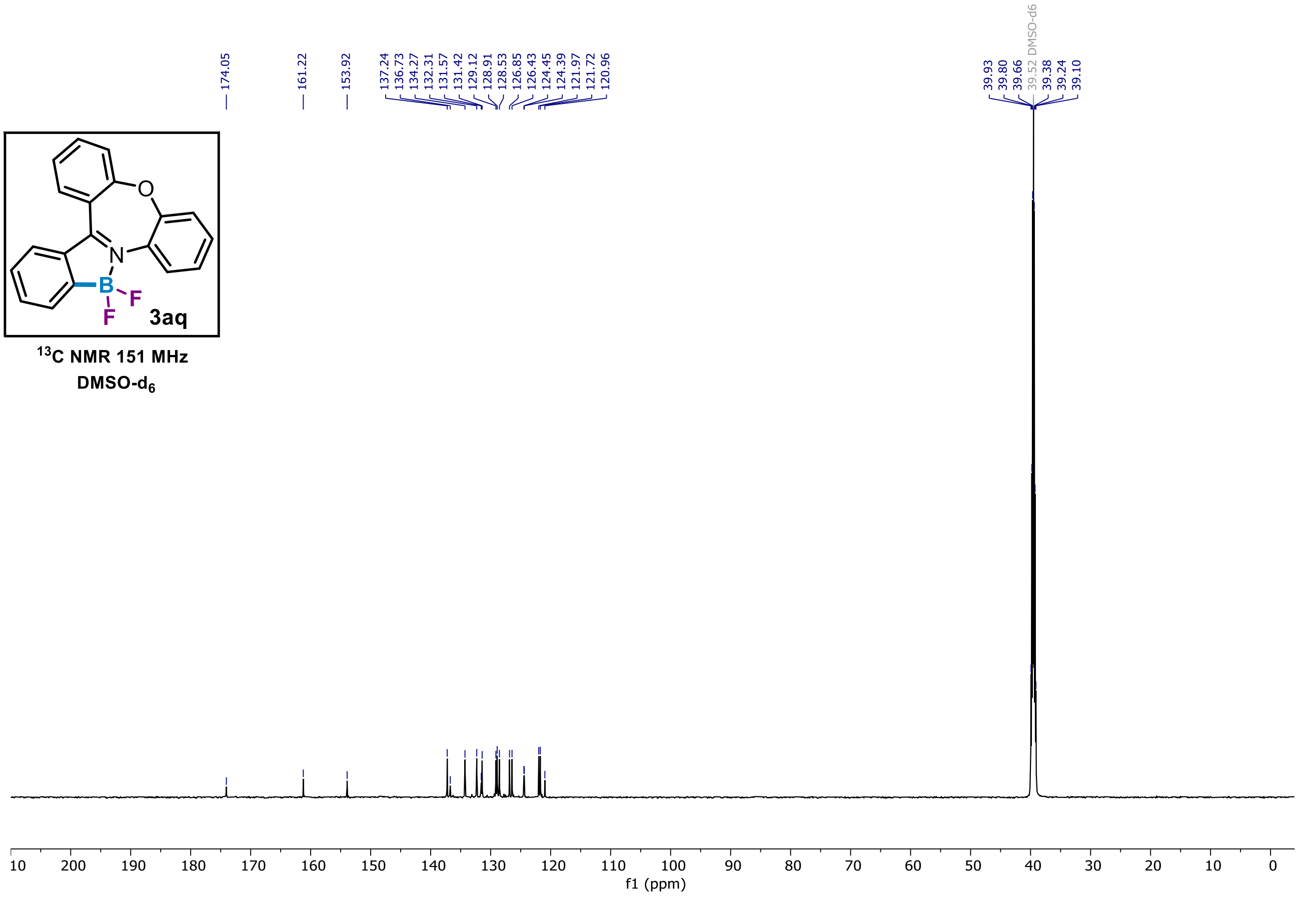

3aq

^{13}C NMR 151 MHz
DMSO-d_6

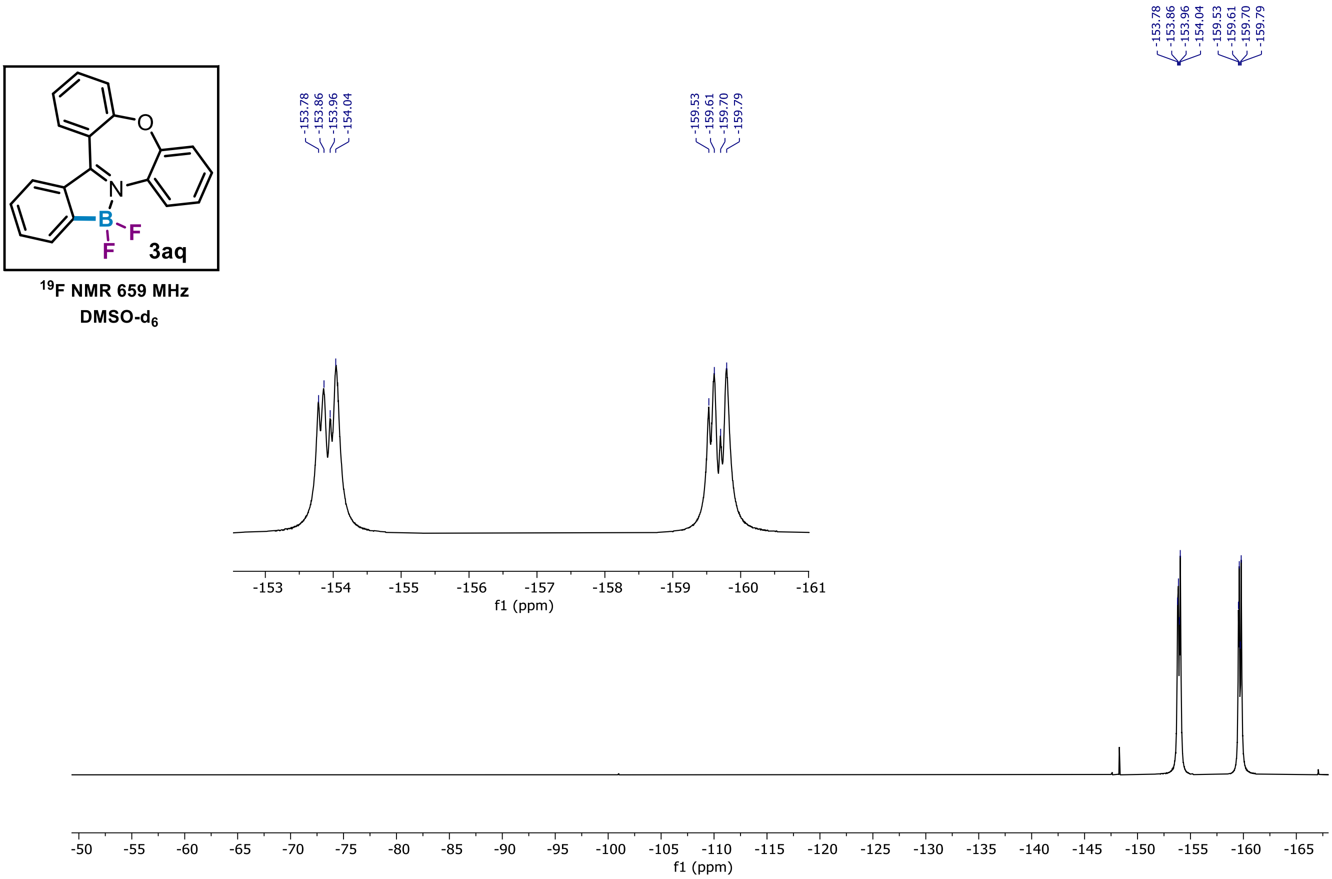

Figure S11-44-3: ^{19}F spectrum of compound **3aq** in DMSO-d$_6$. Two non equivalent fluorines spectrum in the two distereomers.

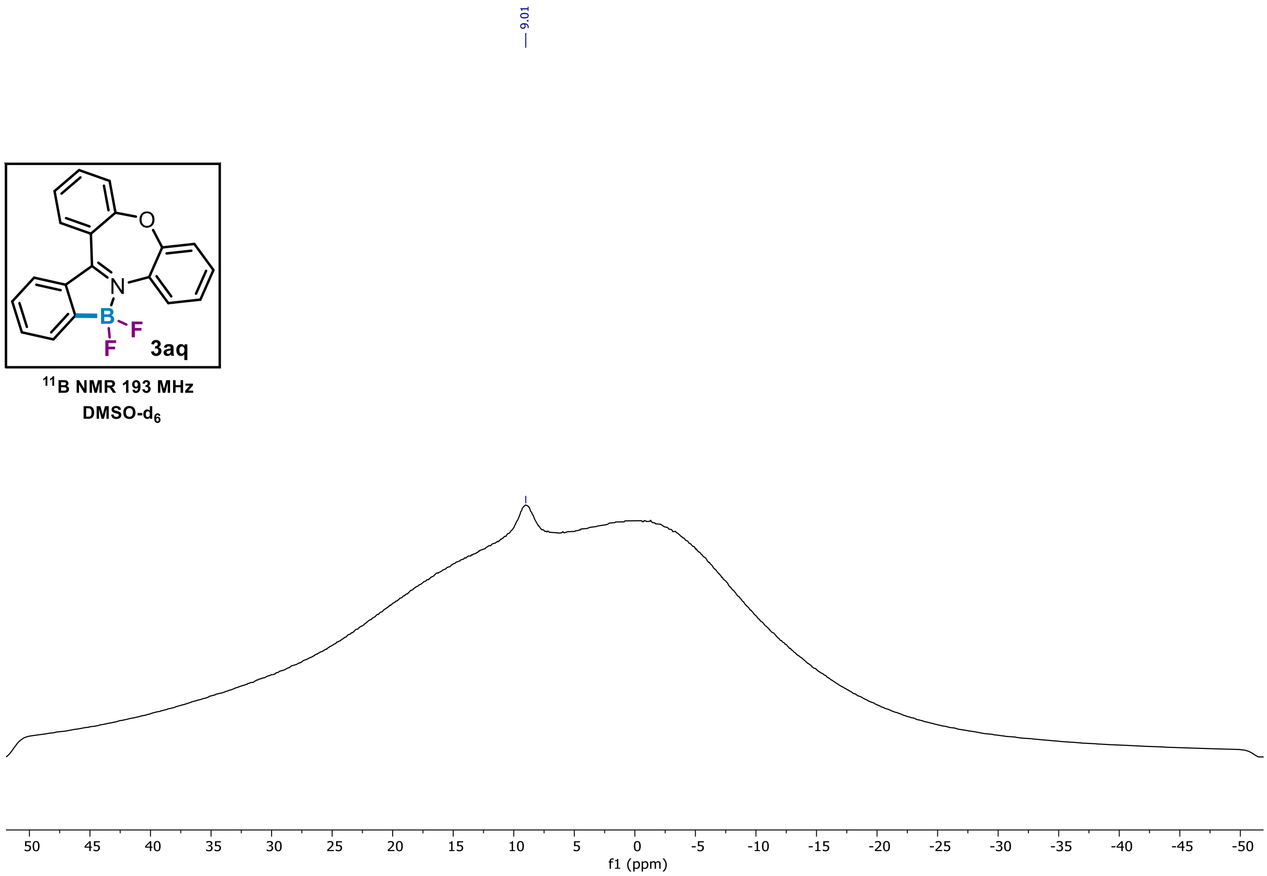

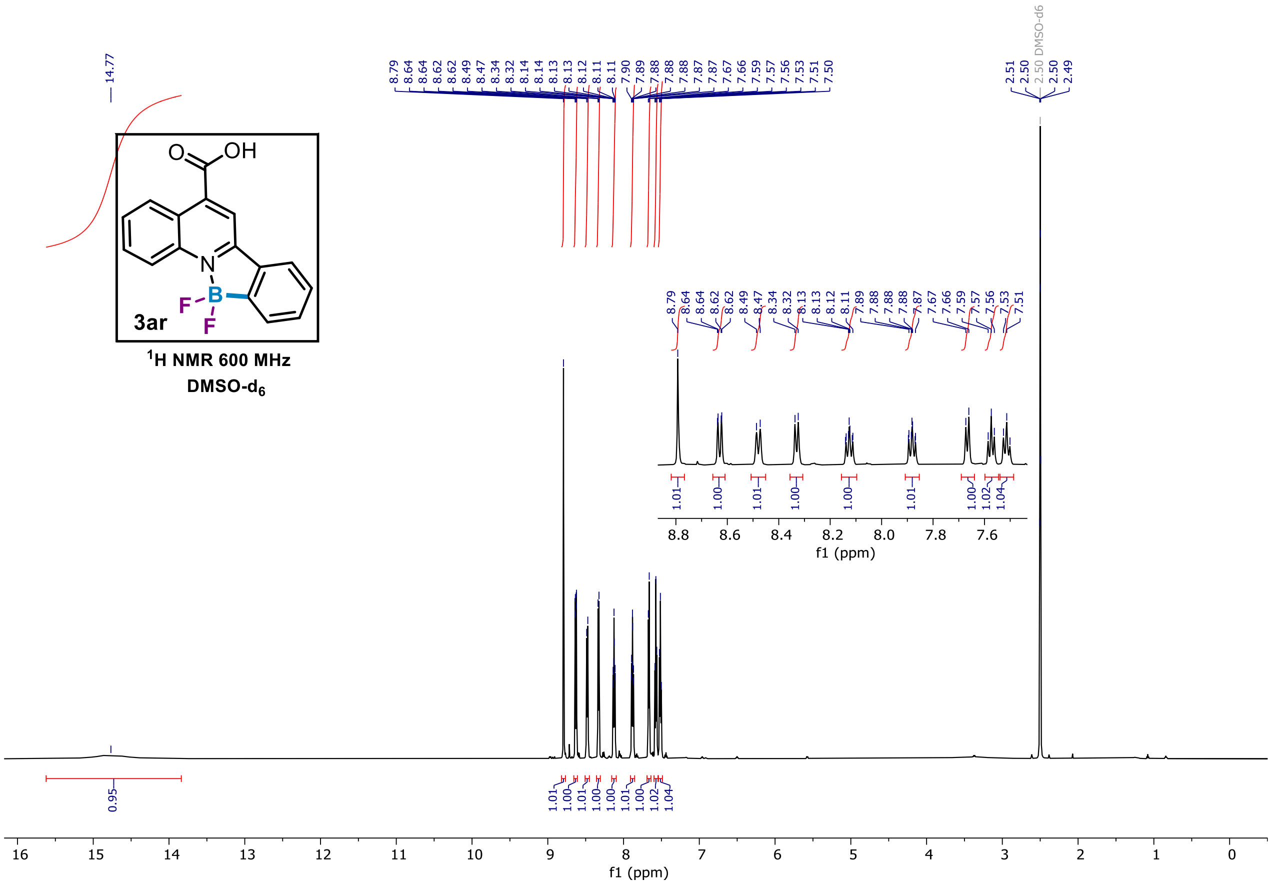

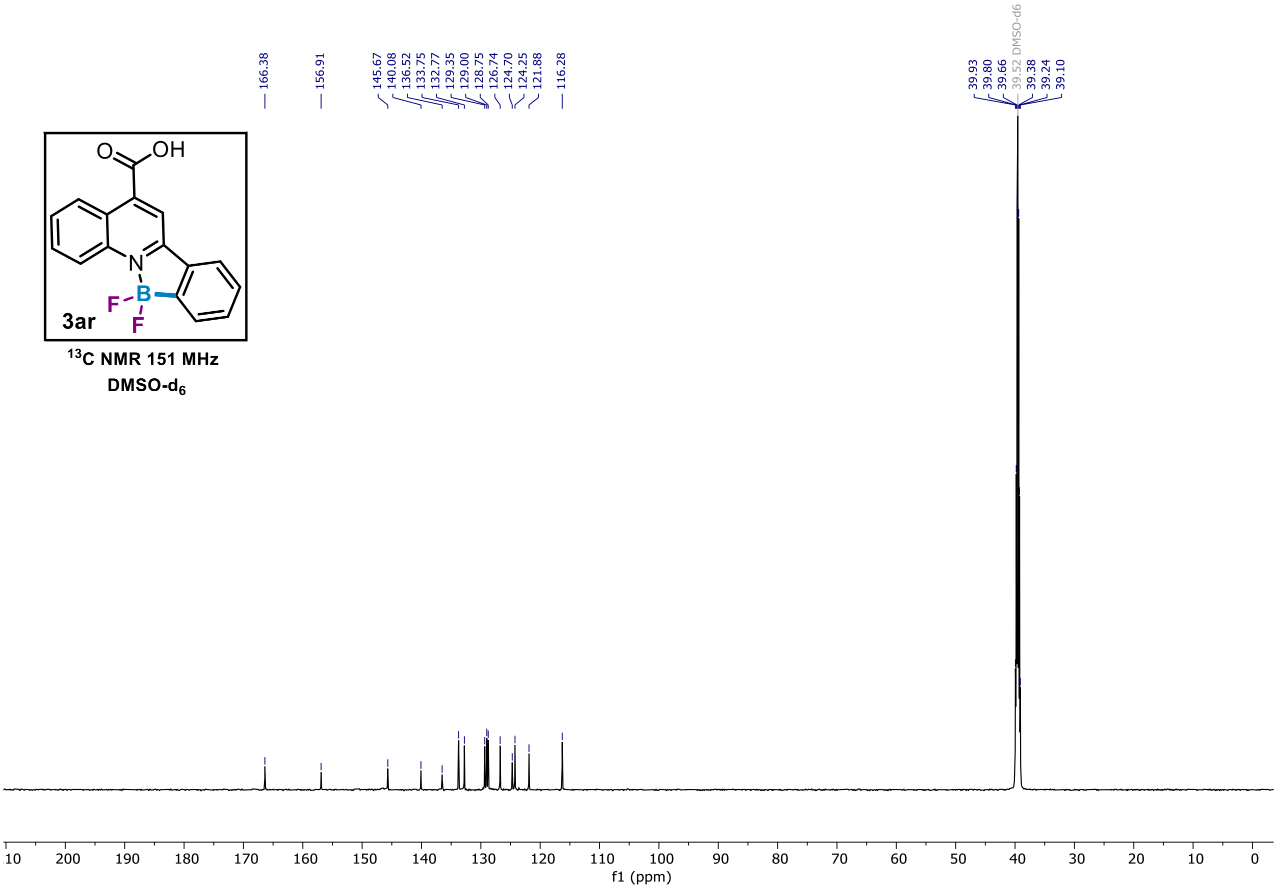

Figure S11-45: ^{13}C spectrum of compound **3ar** in DMSO-d_6. Note that the ^{13}C signal for the C-BF_2 bond does not appear.

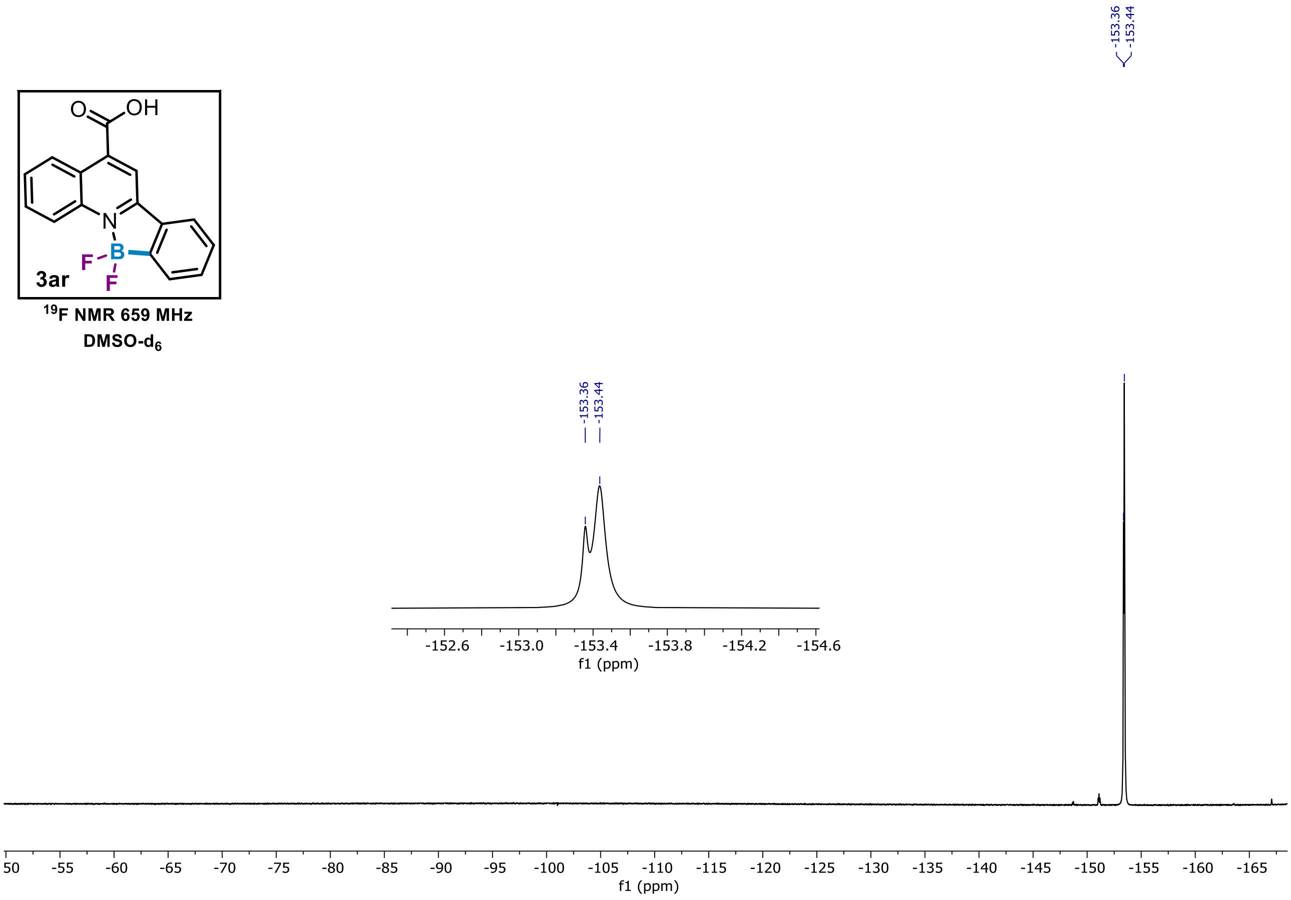
-153.36
-153.44
O
OH
N
B
F
F
3ar
19F NMR 659 MHz
DMSO-d6
-152.6
-153.0
-153.4
-153.8
-154.2
-154.6
f1 (ppm)
50 -55 -60 -65 -70 -75 -80 -85 -90 -95 -100 -105 -110 -115 -120 -125 -130 -135 -140 -145 -150 -155 -160 -165
f1 (ppm)

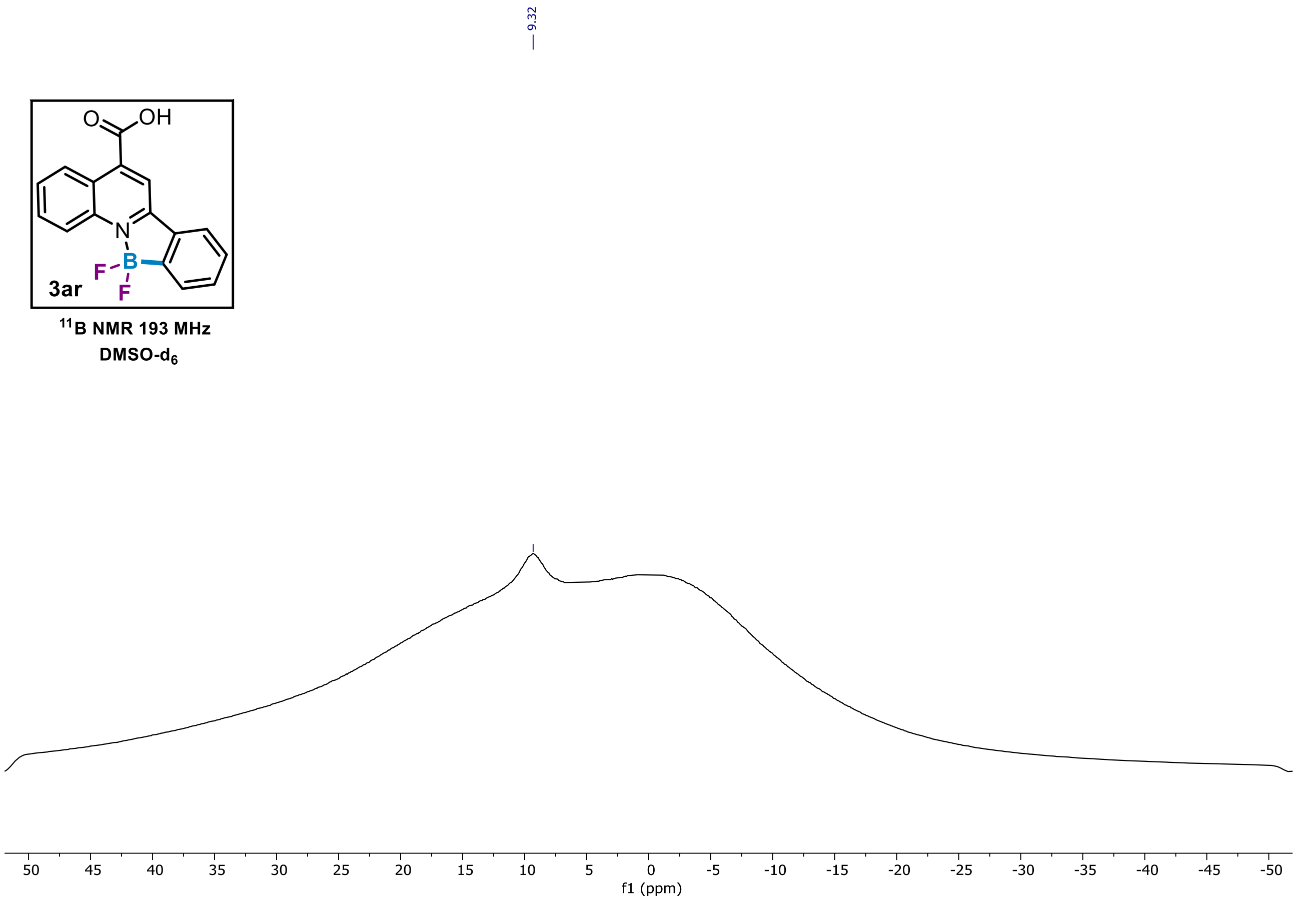
9.32
O
OH
N
B
F
F
3ar
11B NMR 193 MHz
DMSO-d6
50 45 40 35 30 25 20 15 10 5 0 -5 -10 -15 -20 -25 -30 -35 -40 -45 -50
f1 (ppm)

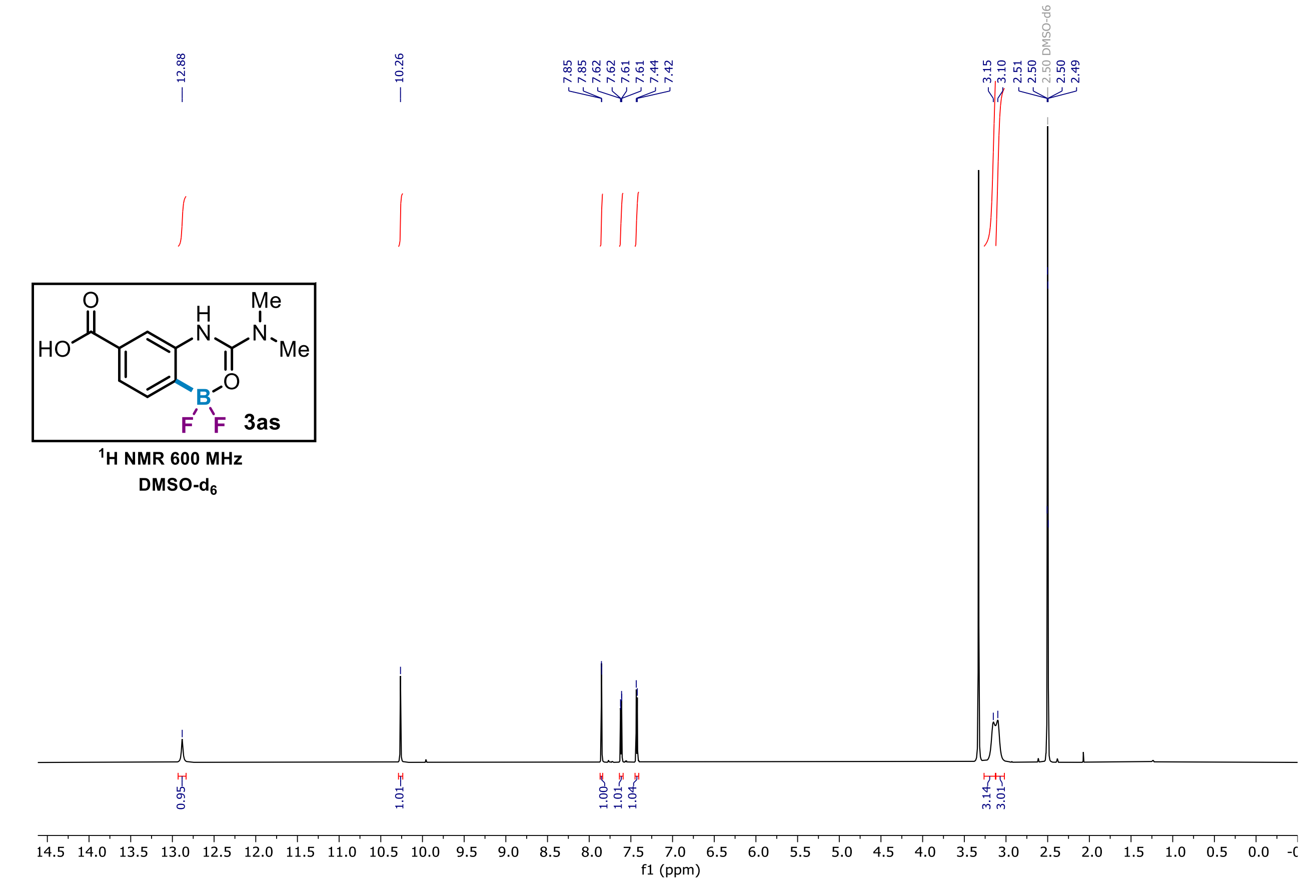

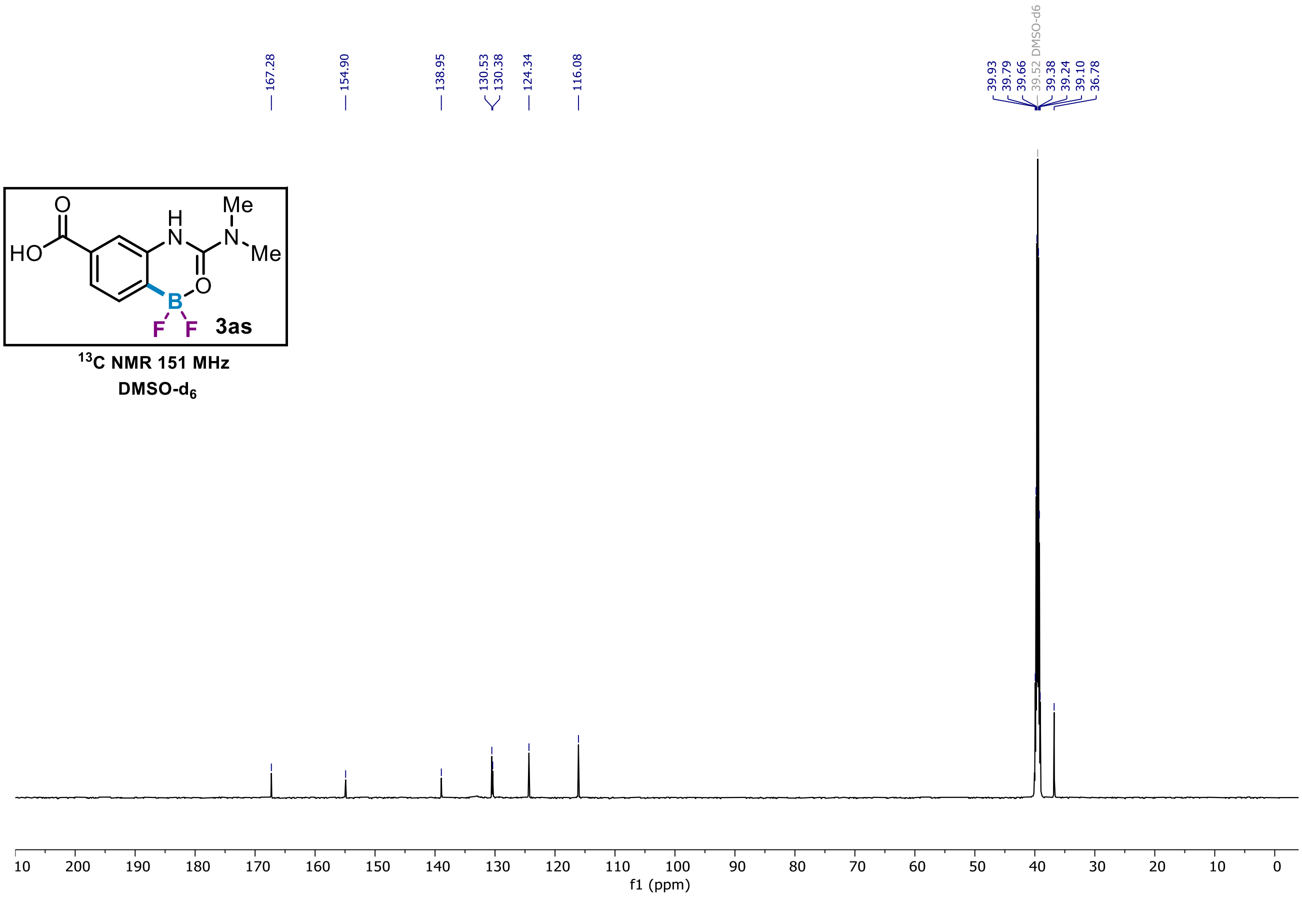

Figure S11-46: ^{13}C spectrum of compound **3as** in DMSO-d$_6$. Note that the ^{13}C signal for the C-BF_2 bond does not appear.

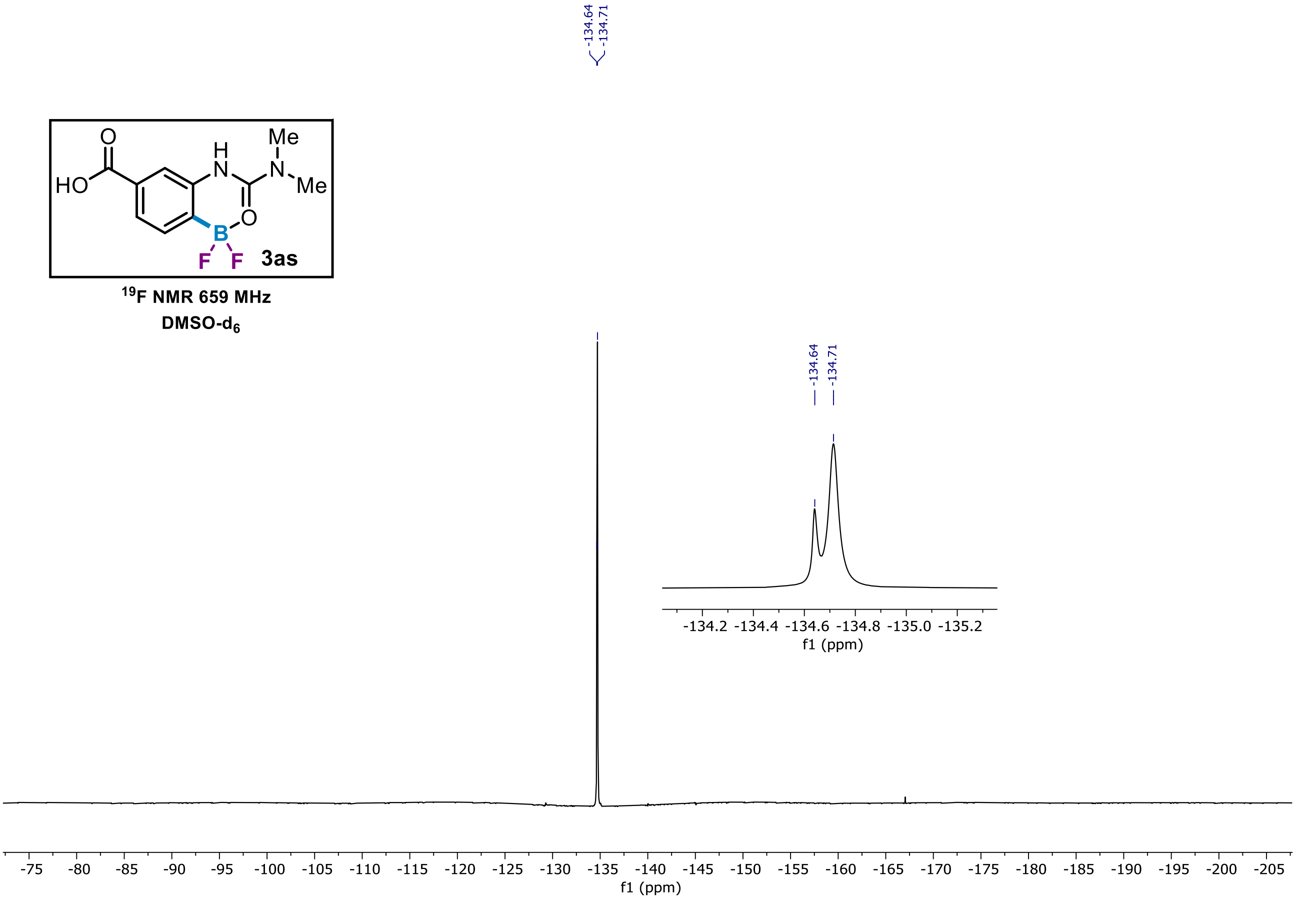
-134.64
-134.71
HO
O
H
N
Me
N
Me
O
B
F
F
3as
^{19}F NMR 659 MHz
DMSO-d$_6$
-134.2 -134.4 -134.6 -134.8 -135.0 -135.2
f1 (ppm)
-75 -80 -85 -90 -95 -100 -105 -110 -115 -120 -125 -130 -135 -140 -145 -150 -155 -160 -165 -170 -175 -180 -185 -190 -195 -200 -205
f1 (ppm)

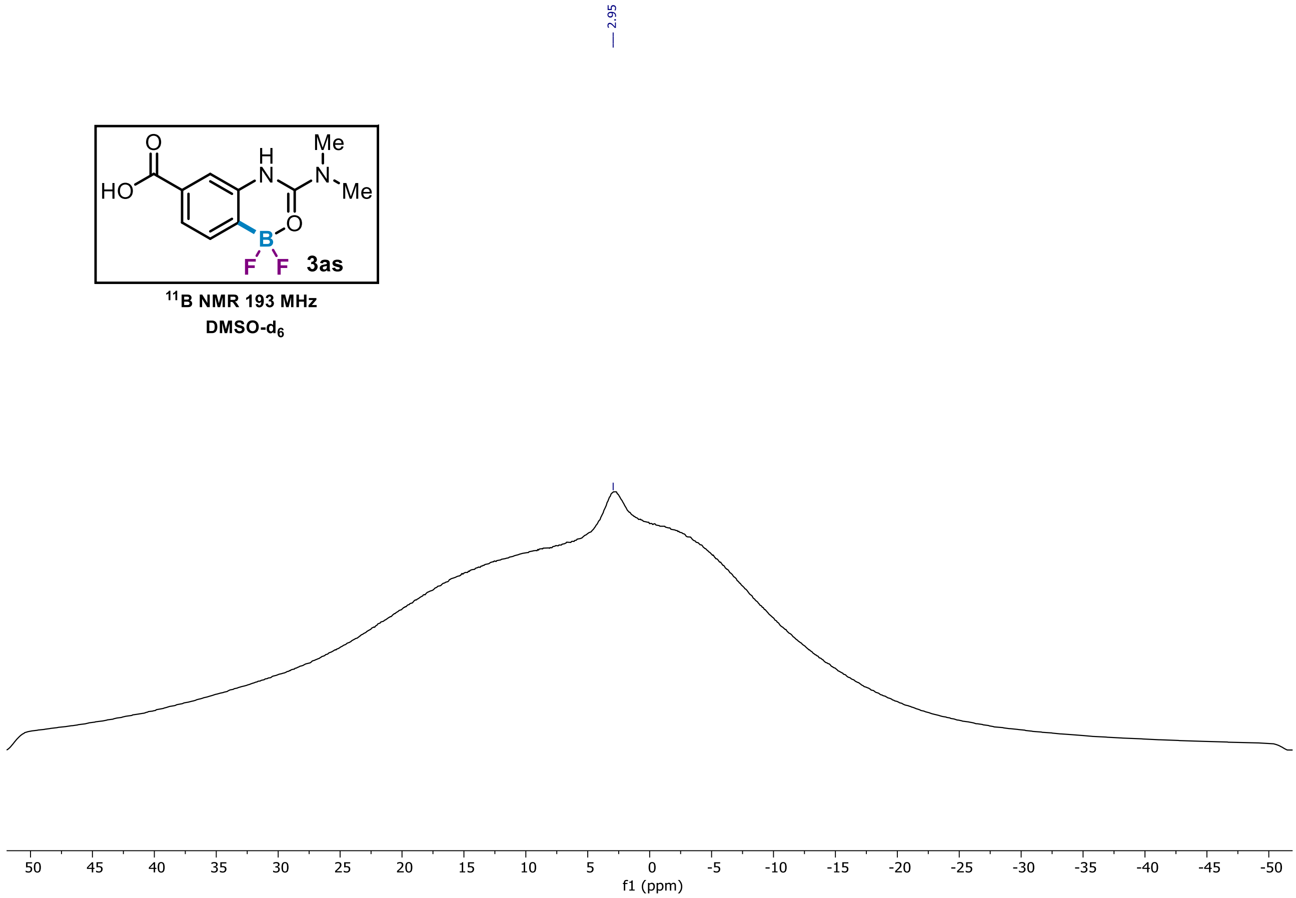
2.95
HO
O
H
N
Me
N
Me
O
B
F
F
3as
^{11}B NMR 193 MHz
DMSO-d$_6$
50 45 40 35 30 25 20 15 10 5 0 -5 -10 -15 -20 -25 -30 -35 -40 -45 -50
f1 (ppm)

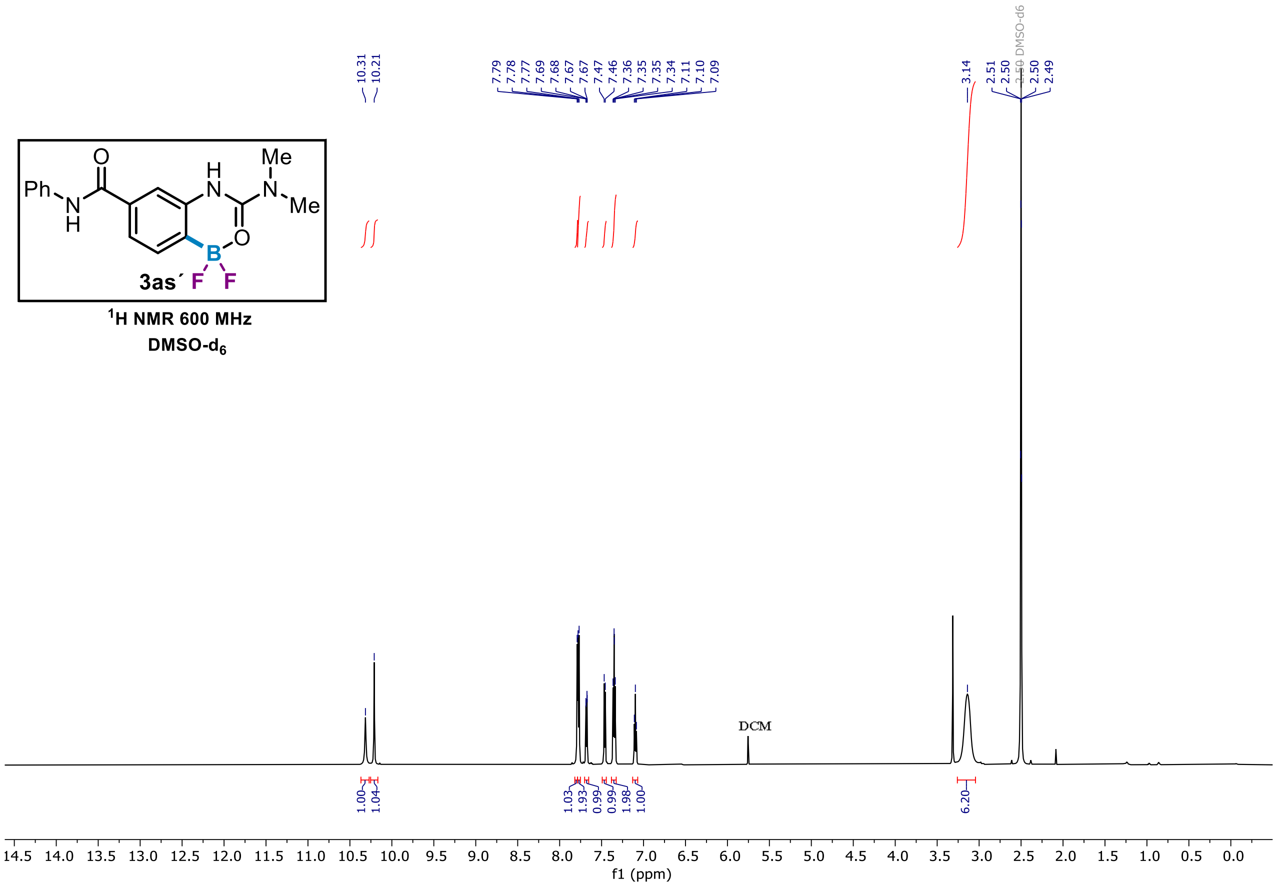

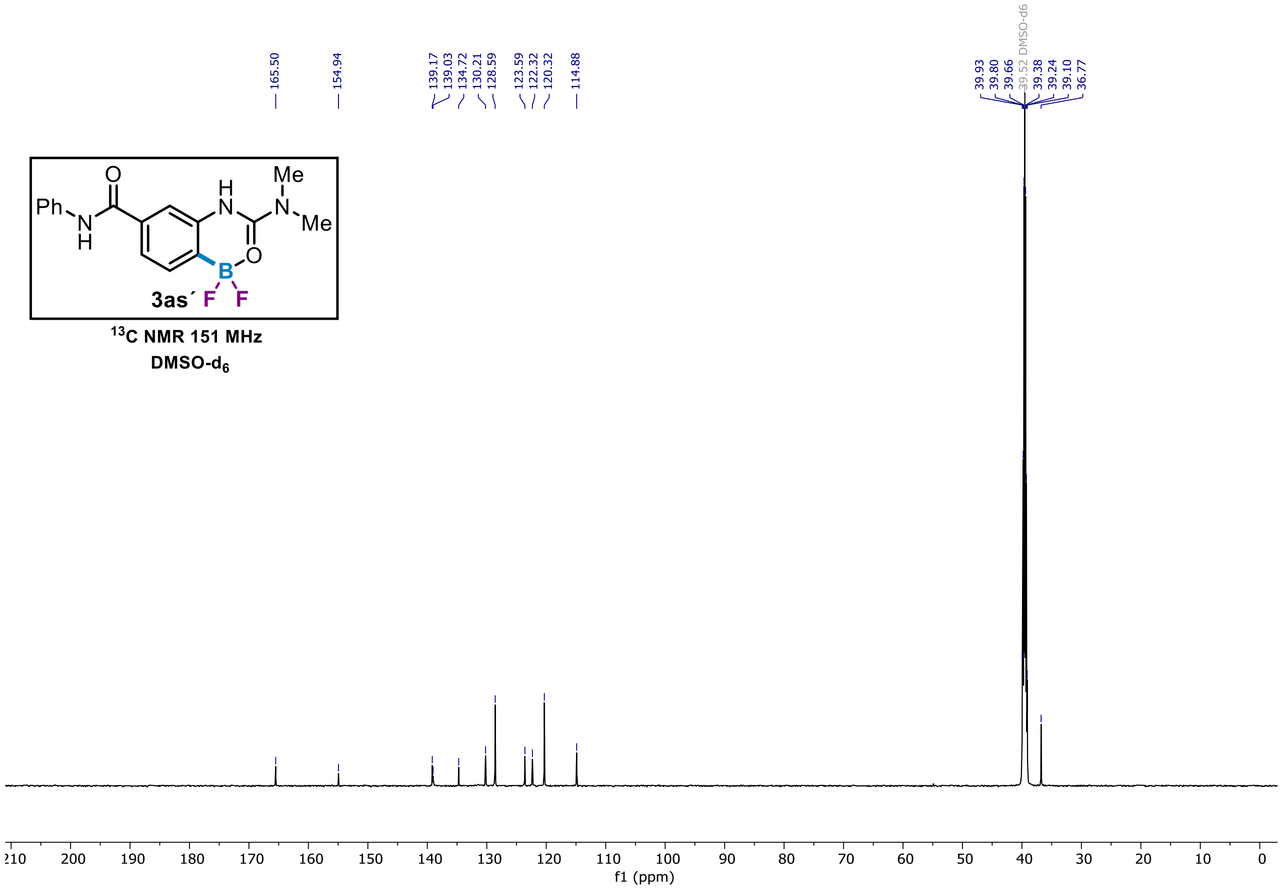

Figure S11-47: ^{13}C spectrum of compound **3as´** in DMSO-d$_6$. Note that the ^{13}C signal for the C-BF$_2$ bond does not appear.

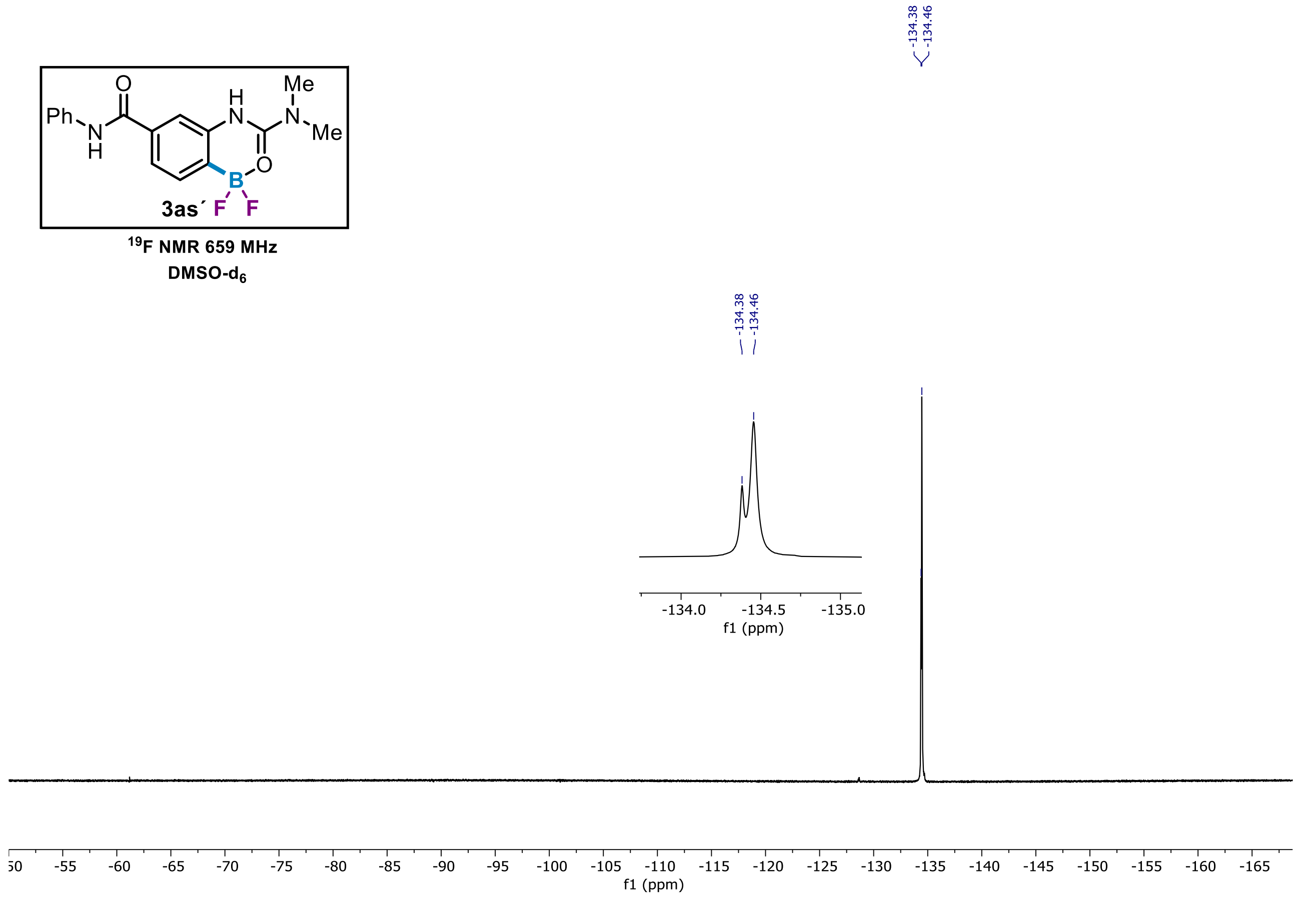
3as´
^{19}F NMR 659 MHz
DMSO-d$_6$
-134.38
-134.46
f1 (ppm)

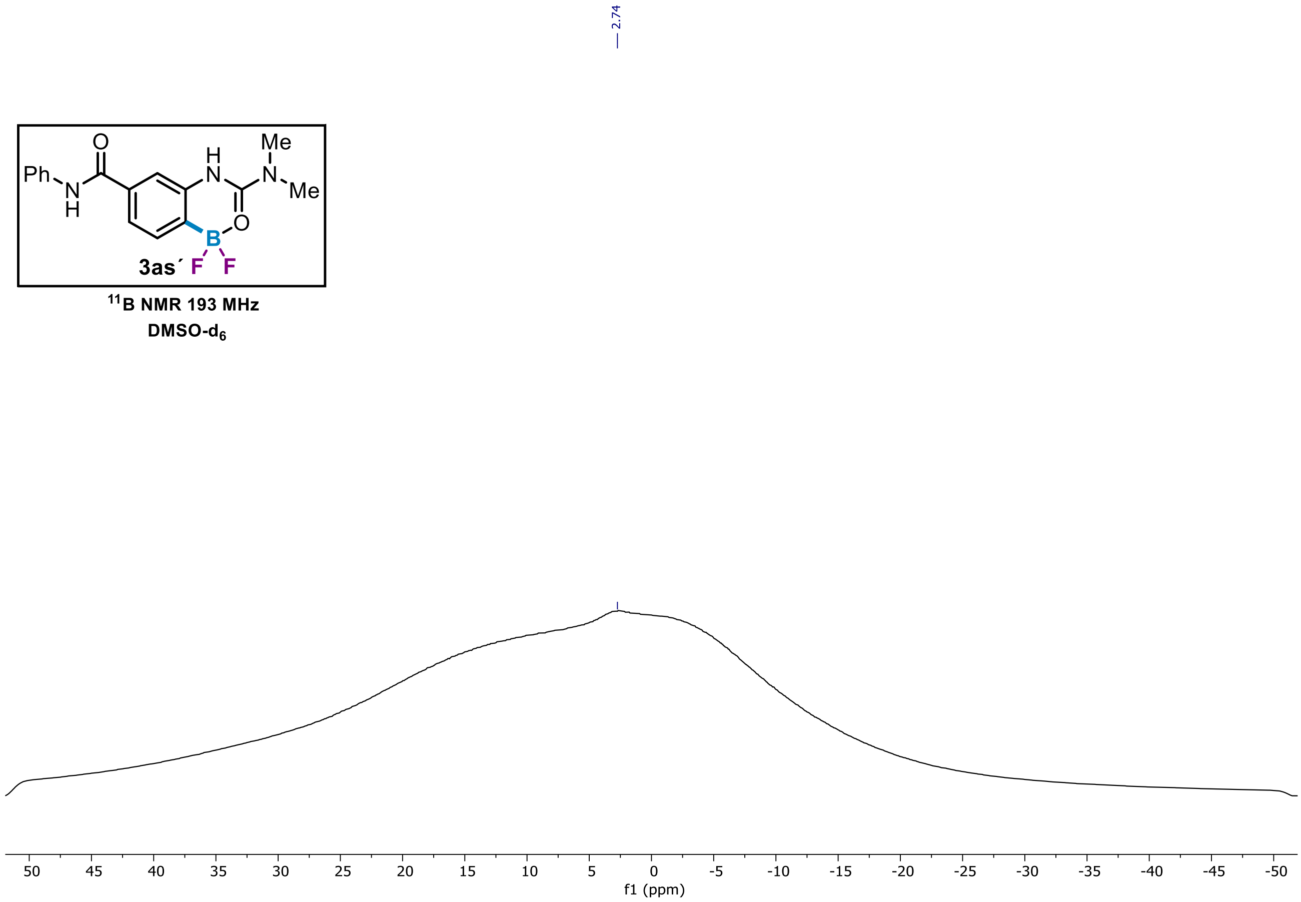
3as´
^{11}B NMR 193 MHz
DMSO-d$_6$
2.74
f1 (ppm)

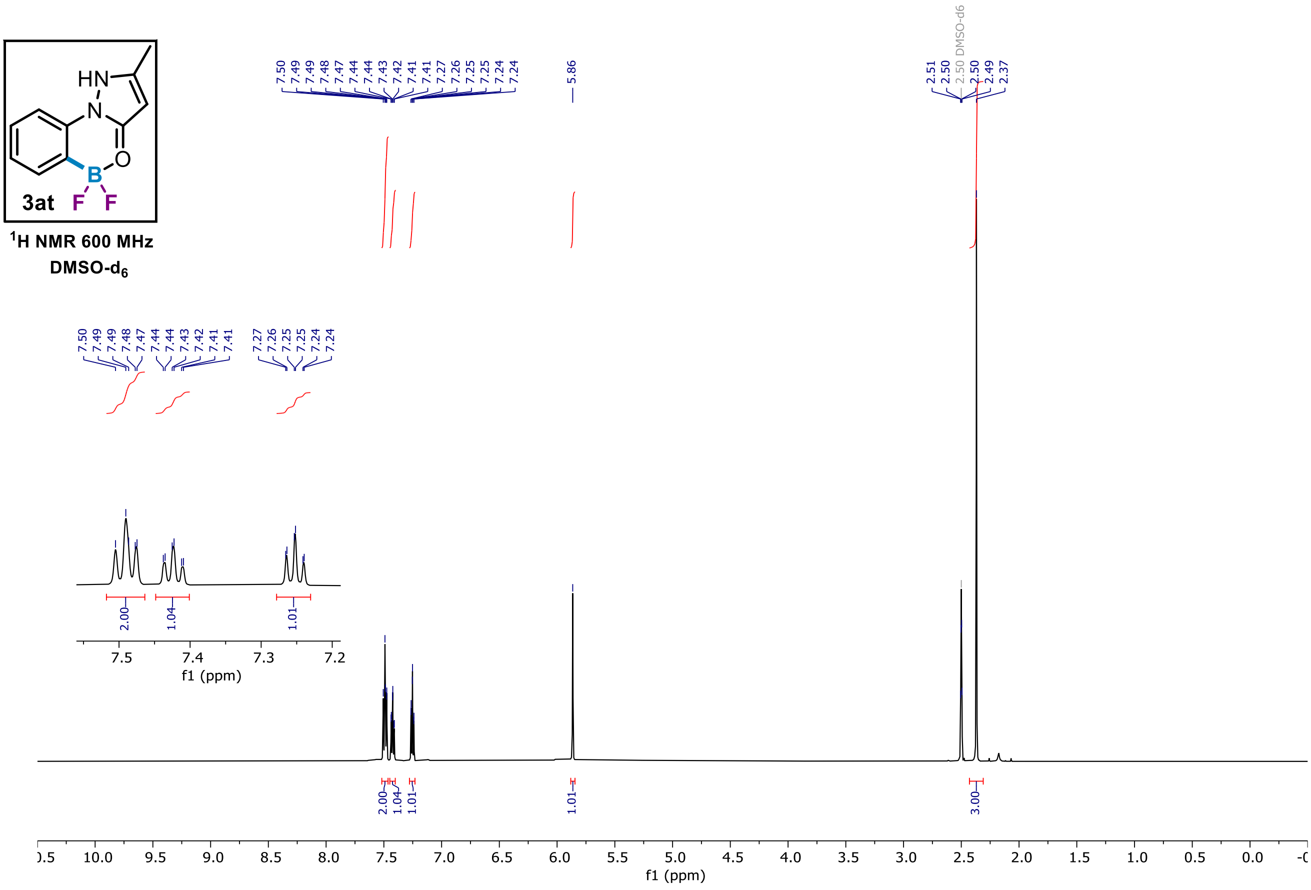

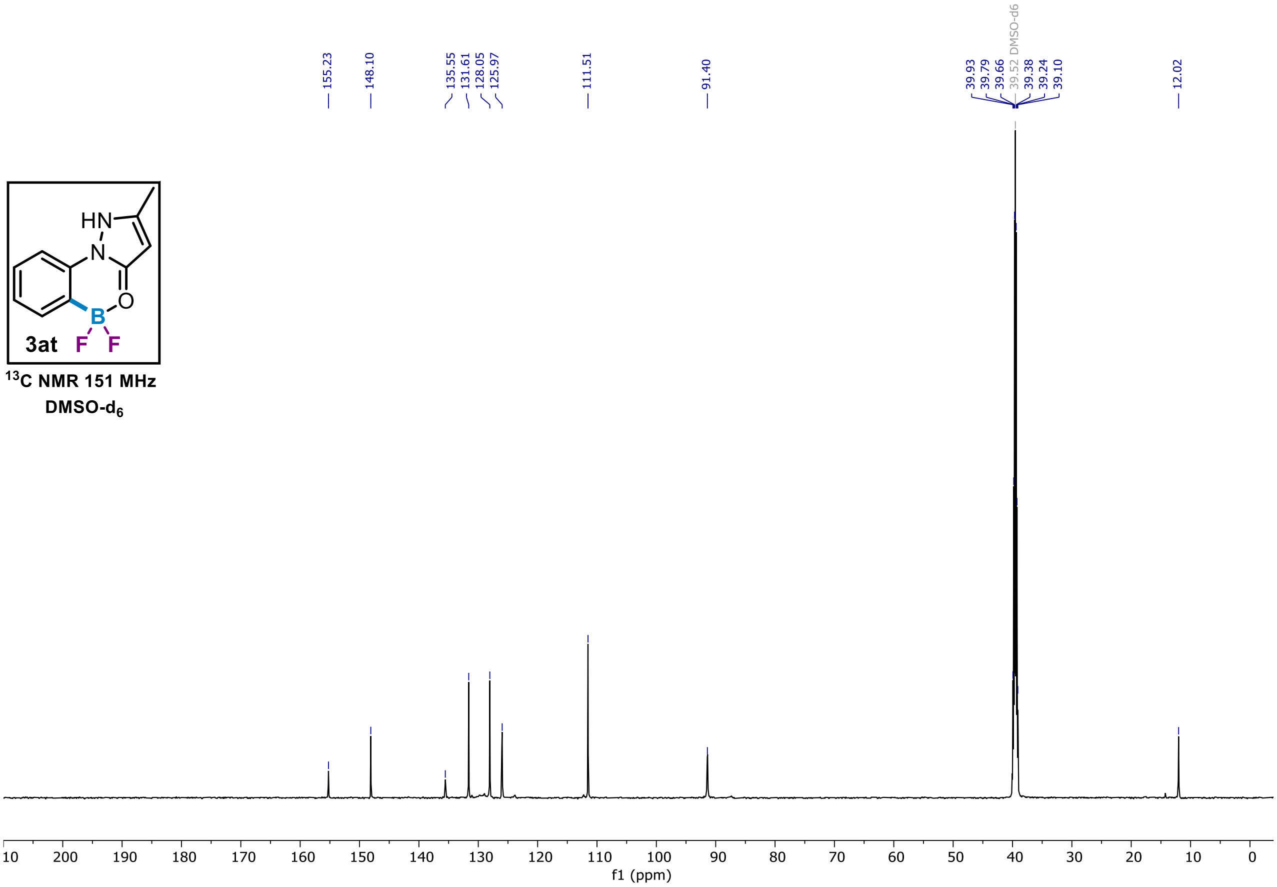

Figure S11-48: ^{13}C spectrum of compound **3at** in DMSO-d_6. Note that the ^{13}C signal for the C-BF_2 bond does not appear.

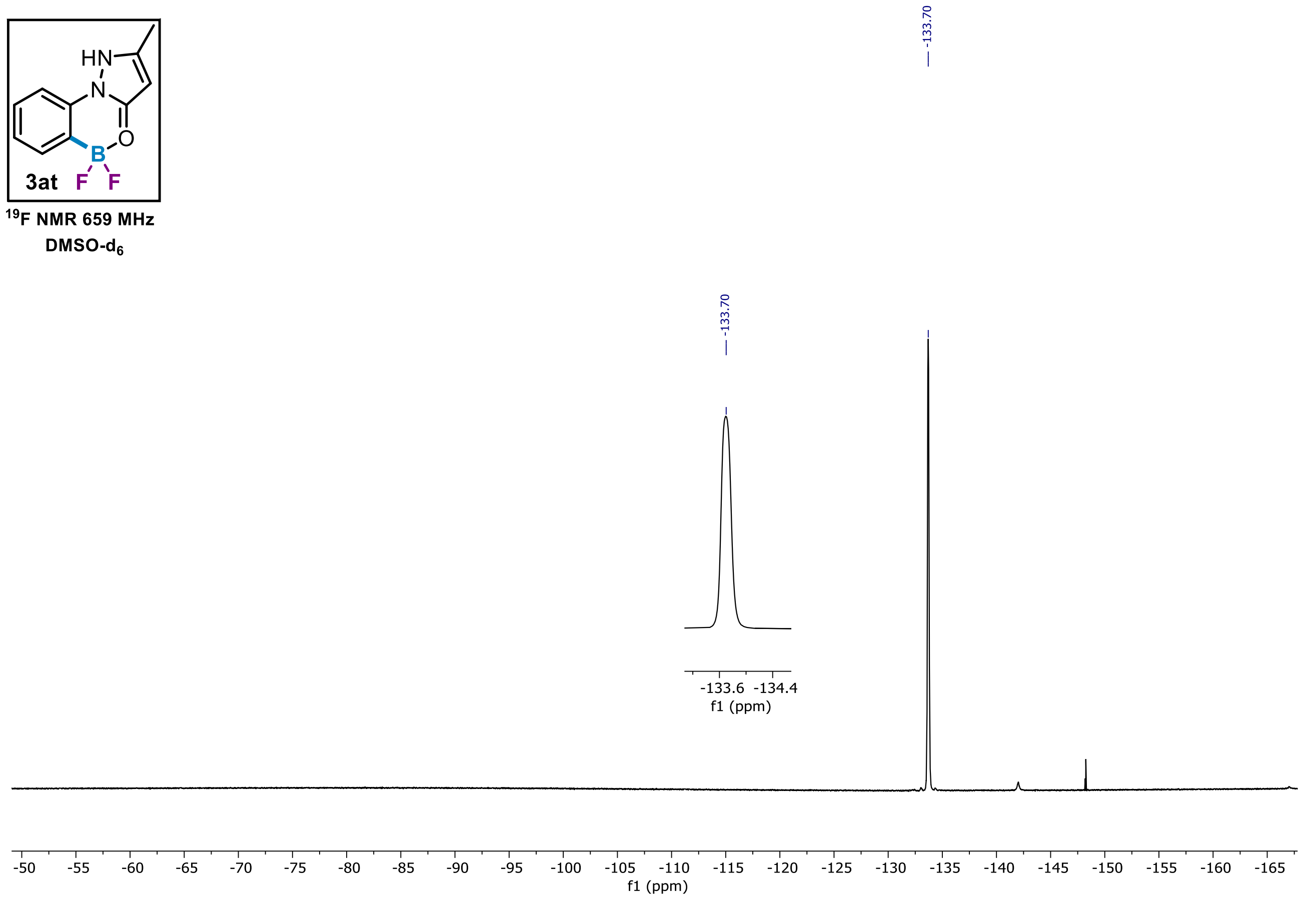
HN
N
O
B
3at
F
F
19F NMR 659 MHz
DMSO-d6
-133.70
-133.70
-133.6
-134.4
f1 (ppm)
-50 -55 -60 -65 -70 -75 -80 -85 -90 -95 -100 -105 -110 -115 -120 -125 -130 -135 -140 -145 -150 -155 -160 -165
f1 (ppm)

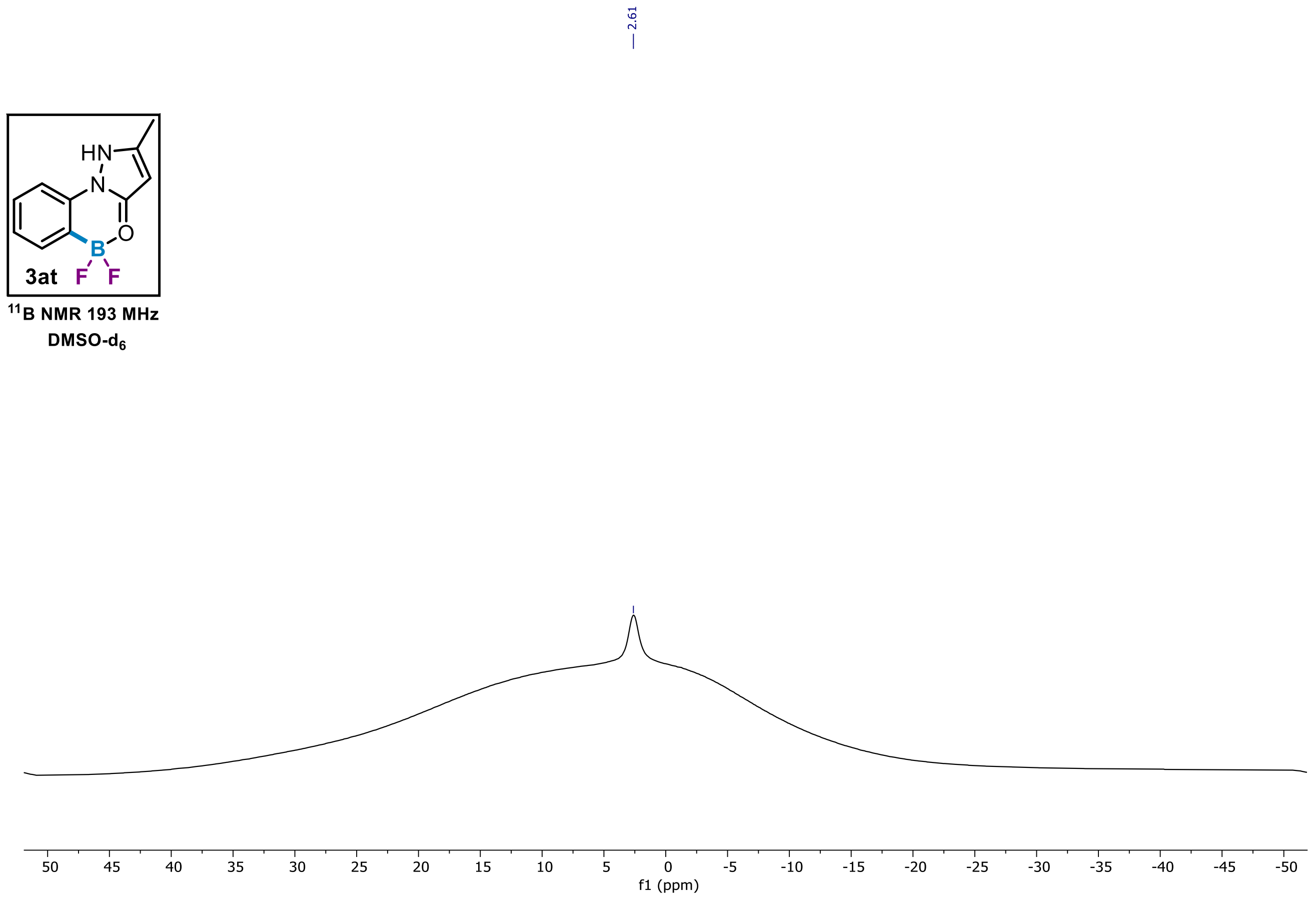
2.61
HN
N
O
B
3at
F
F
11B NMR 193 MHz
DMSO-d6
50 45 40 35 30 25 20 15 10 5 0 -5 -10 -15 -20 -25 -30 -35 -40 -45 -50
f1 (ppm)

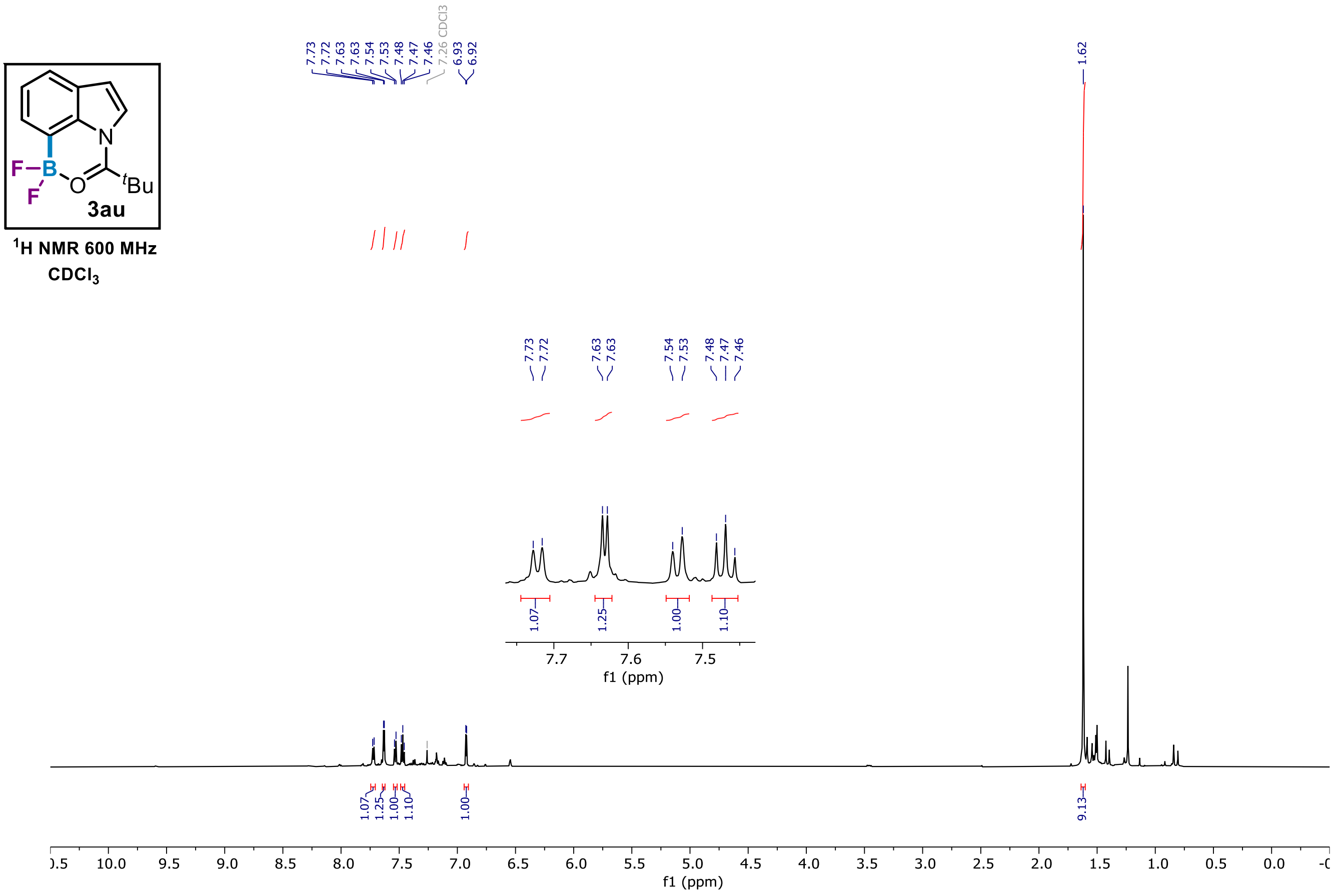

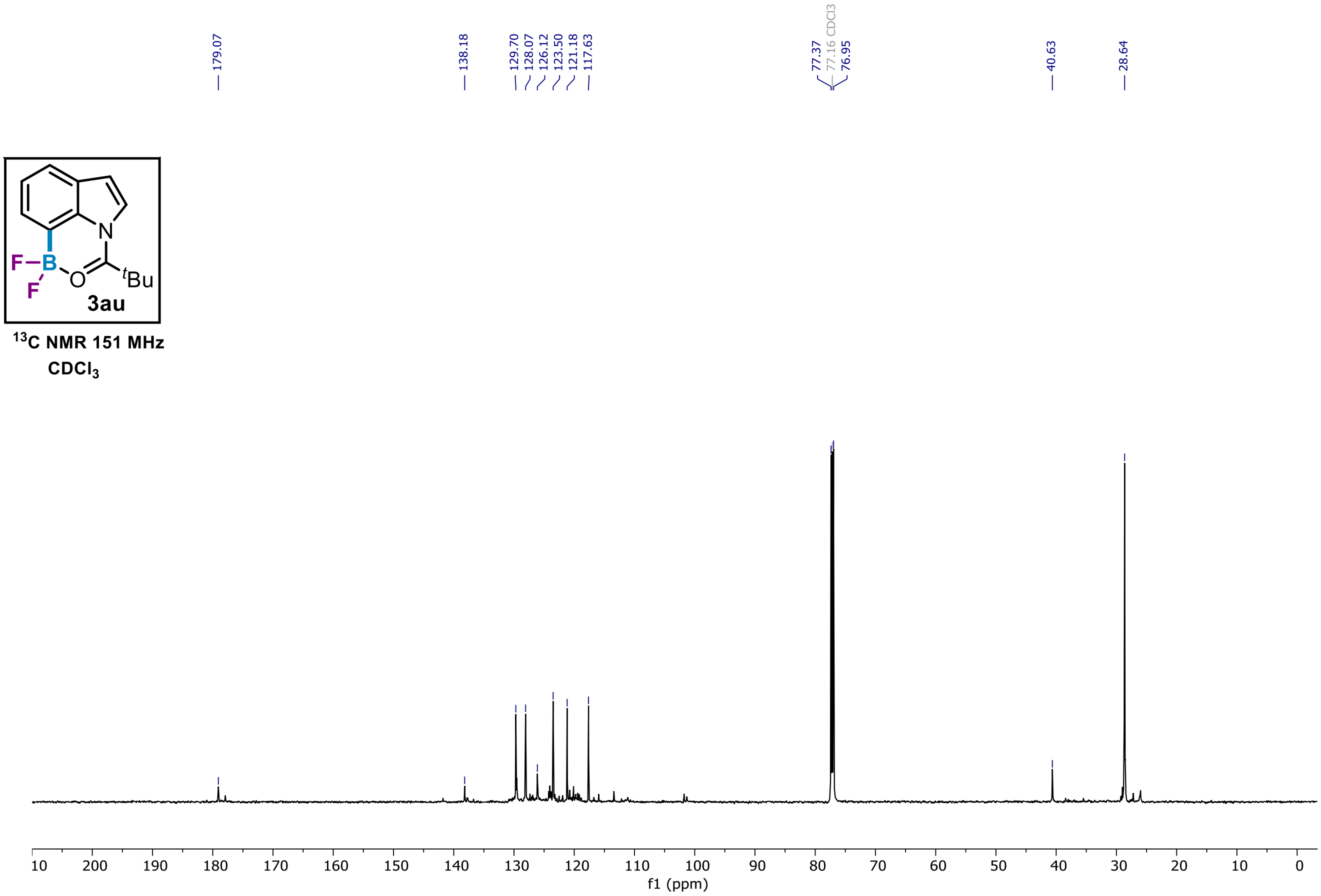

Figure S11-49: ^{13}C spectrum of compound **3au** in $CDCl_3$. Note that the ^{13}C signal for the C-BF_2 bond does not appear.

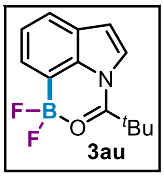

^{19}F NMR 564 MHz
$CDCl_3$

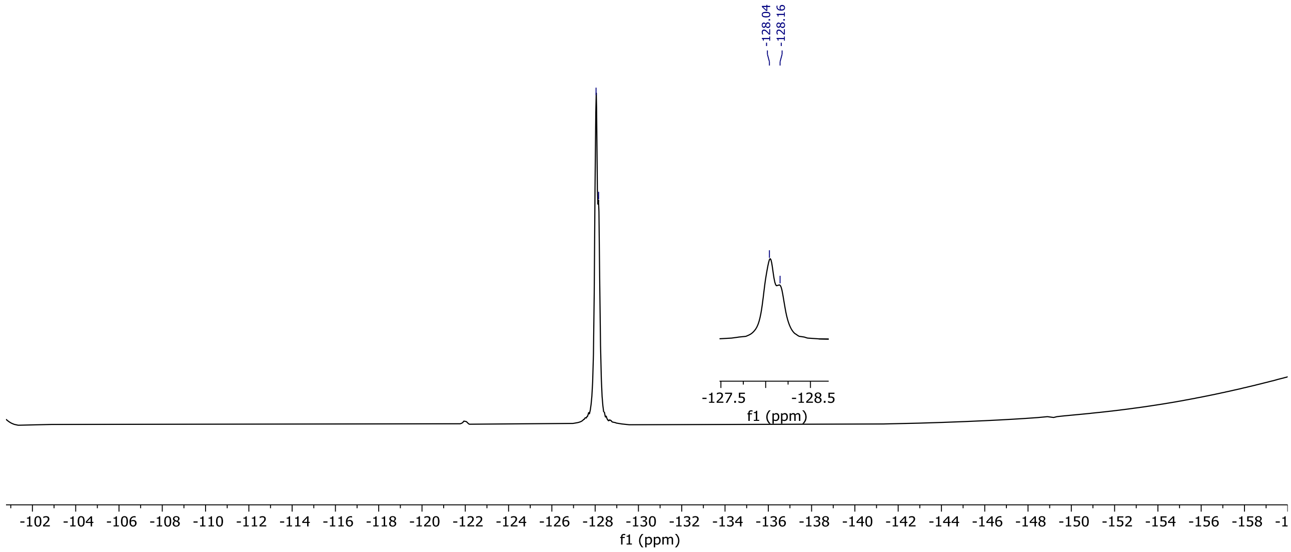

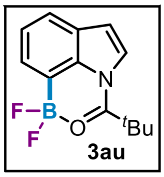

^{11}B NMR 193 MHz
$CDCl_3$

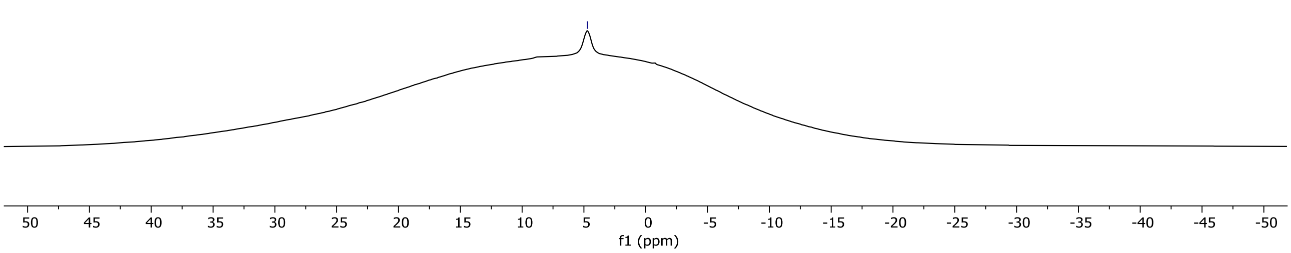

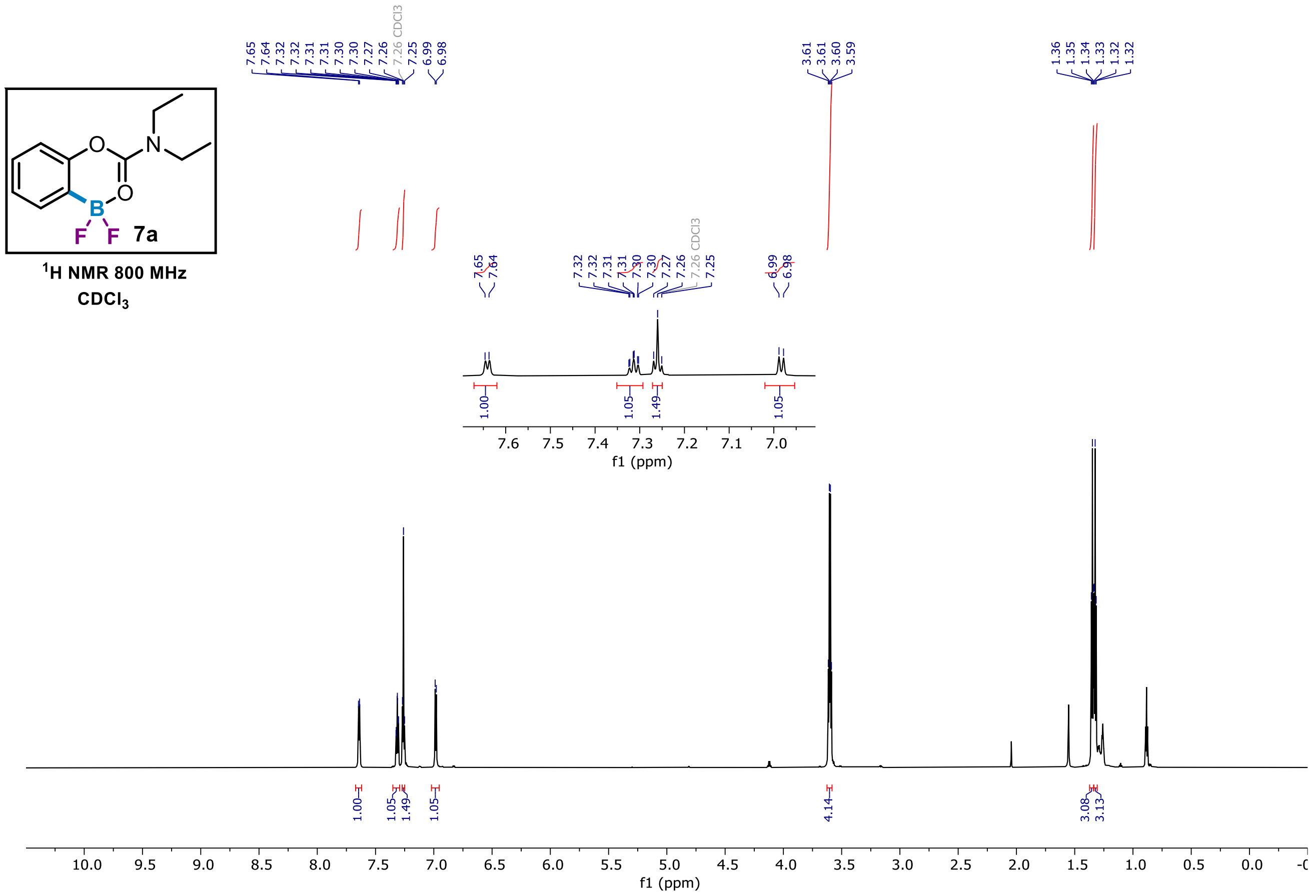

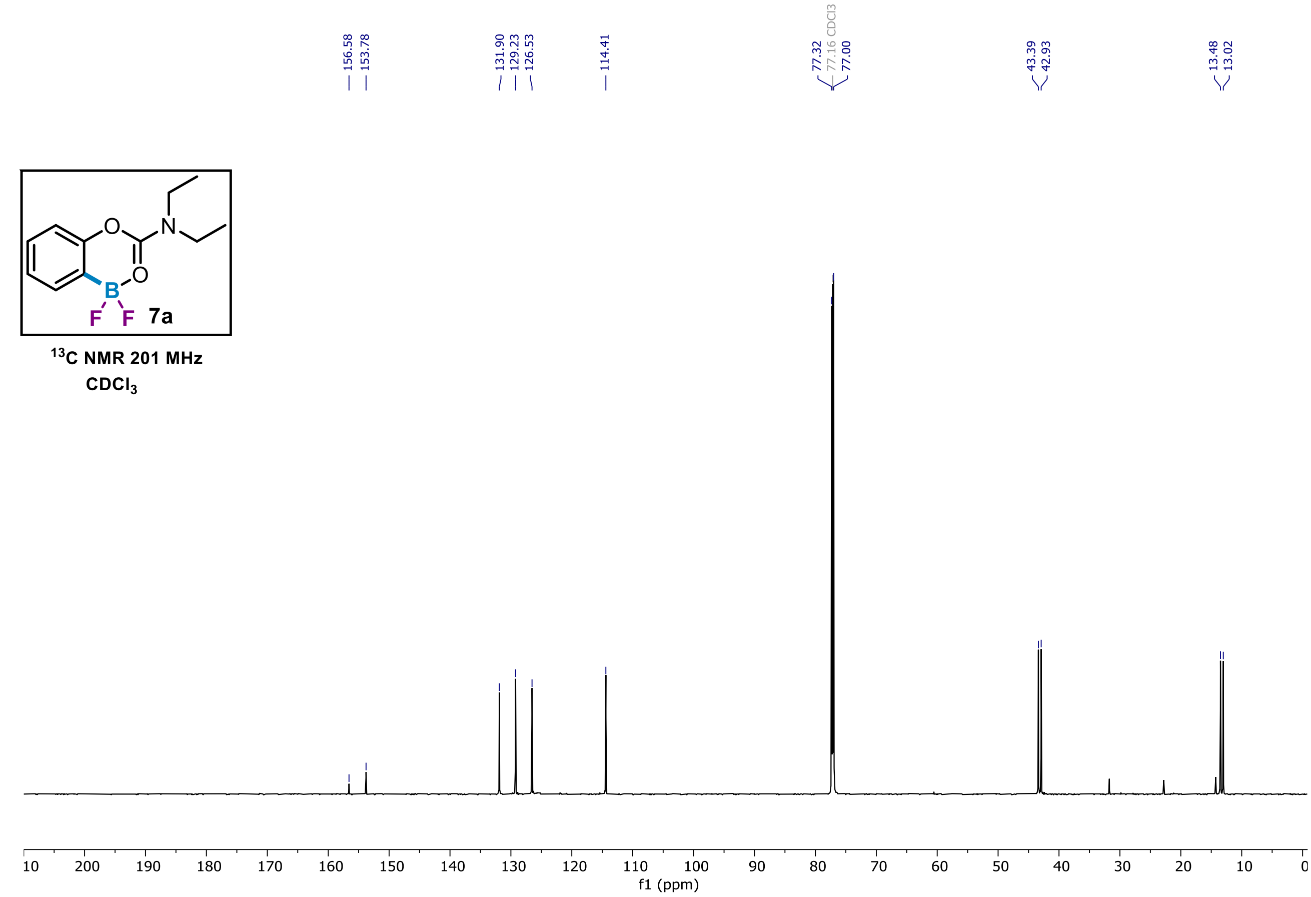

Figure S11-50: ^{13}C spectrum of compound **7a** in $CDCl_3$. Note that the ^{13}C signal for the C-BF_2 bond does not appear.

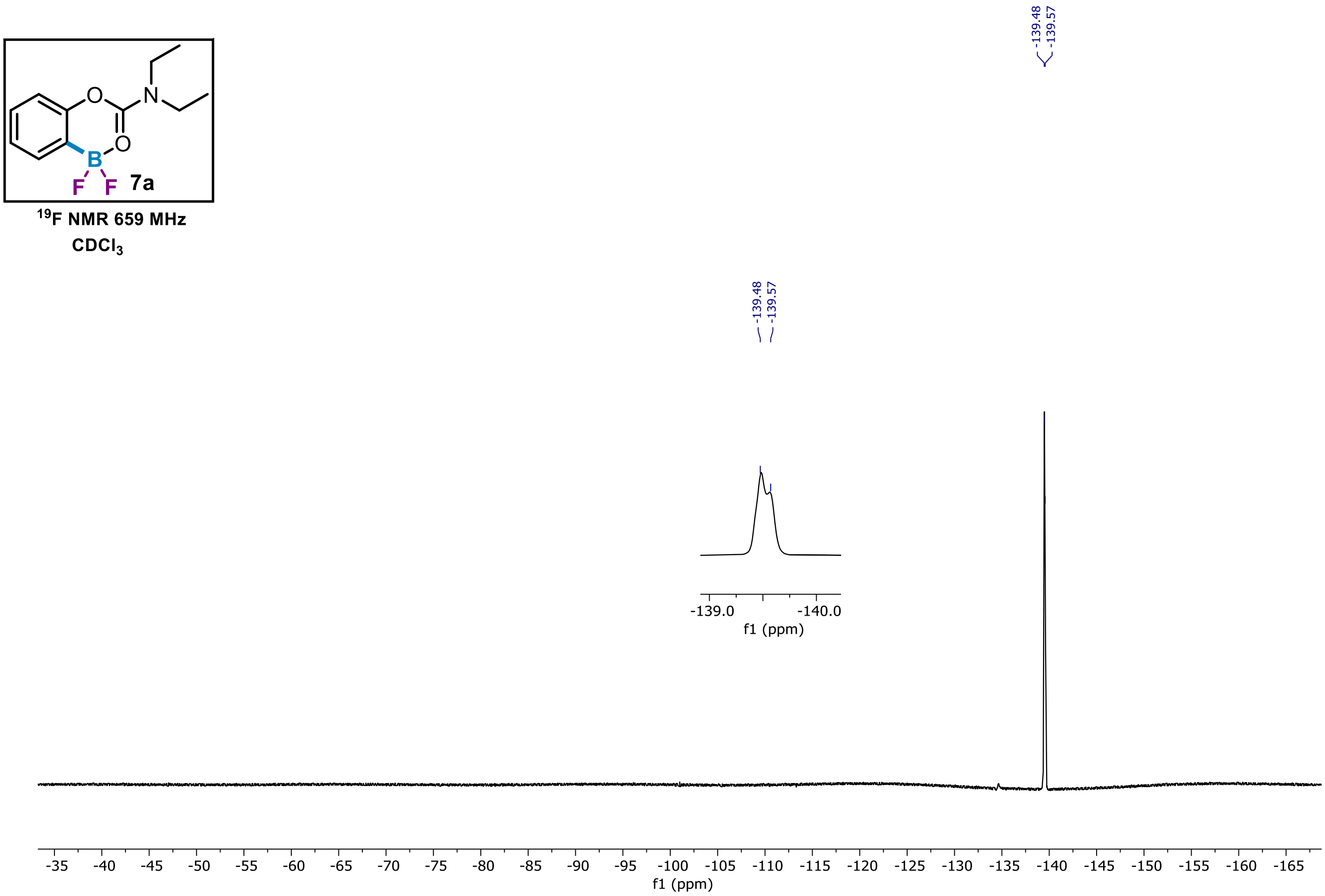
O
N
O
B
F
F
7a
^{19}F NMR 659 MHz
CDCl$_3$
-139.48
-139.57
-139.0
-140.0
f1 (ppm)
-35 -40 -45 -50 -55 -60 -65 -70 -75 -80 -85 -90 -95 -100 -105 -110 -115 -120 -125 -130 -135 -140 -145 -150 -155 -160 -165
f1 (ppm)

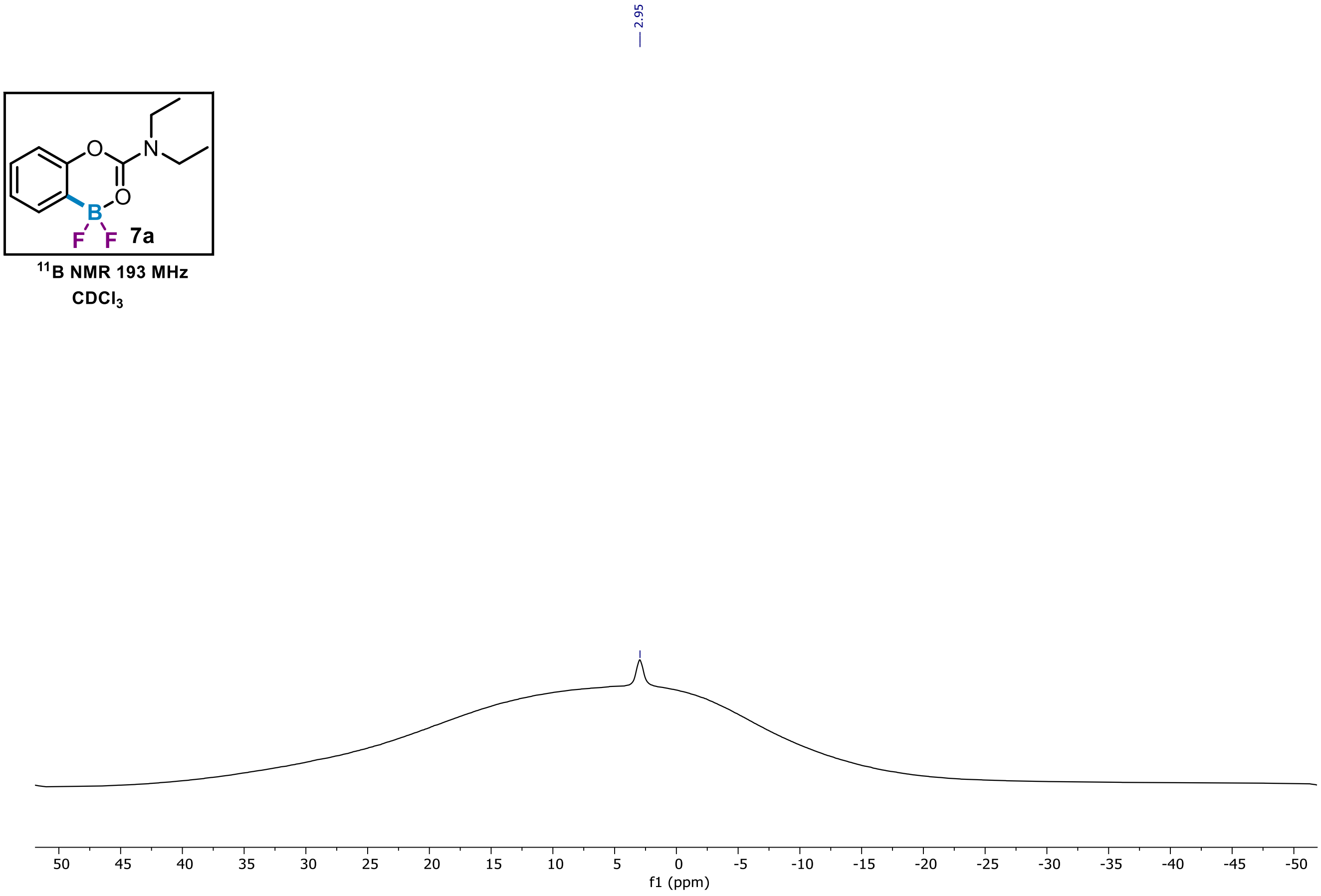
O
N
O
B
F
F
7a
^{11}B NMR 193 MHz
CDCl$_3$
2.95
50 45 40 35 30 25 20 15 10 5 0 -5 -10 -15 -20 -25 -30 -35 -40 -45 -50
f1 (ppm)

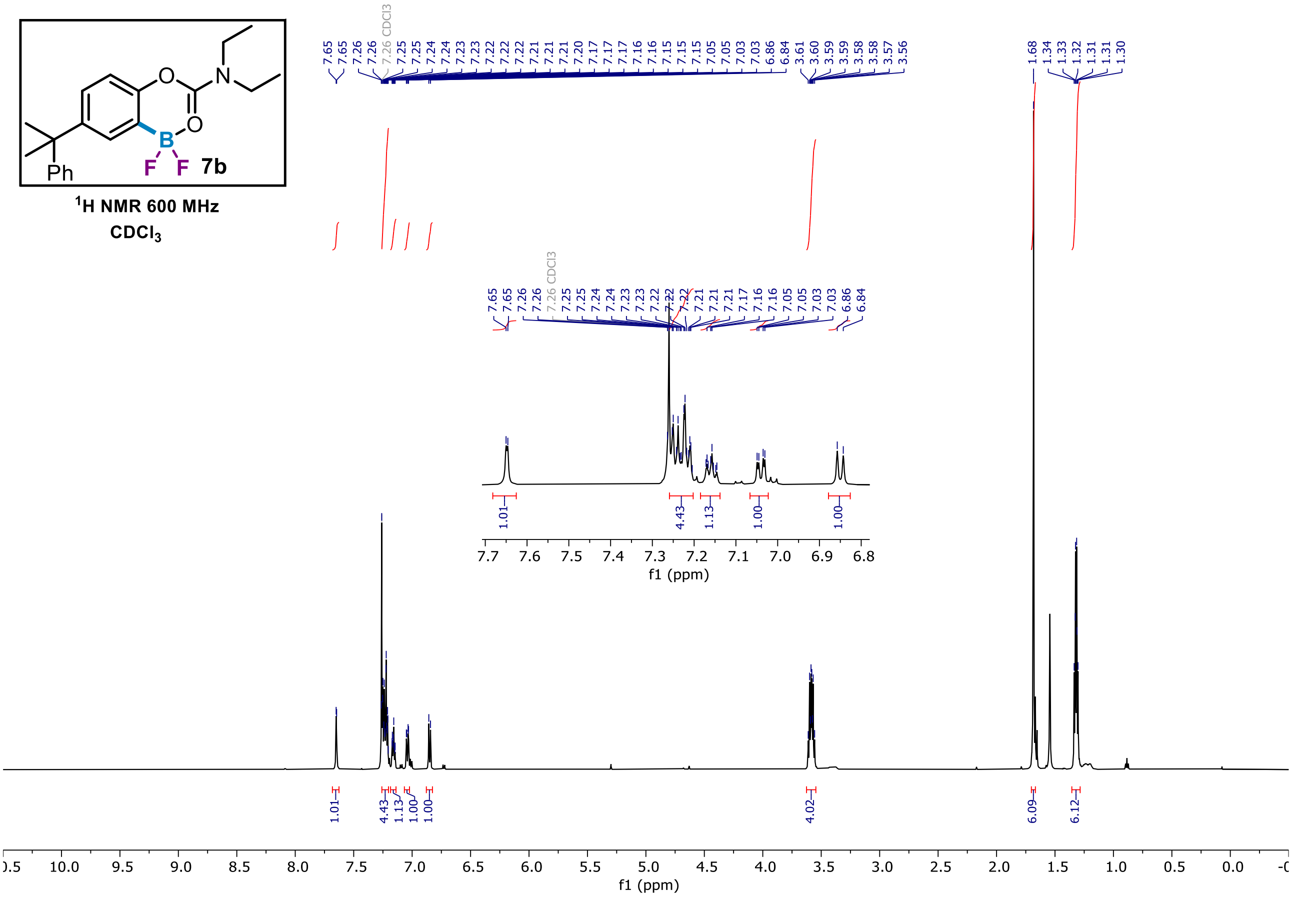

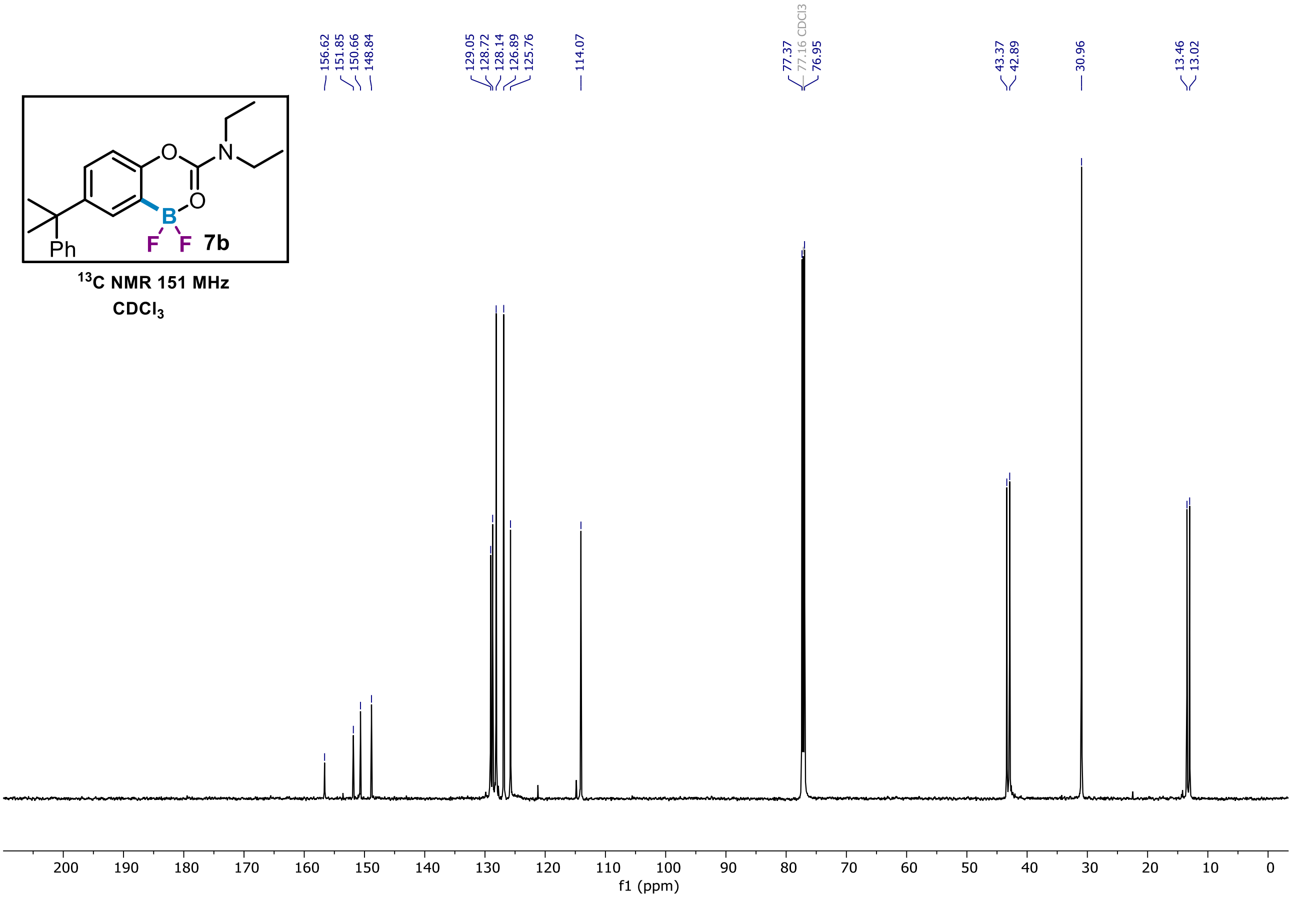

Figure S11-51: ^{13}C spectrum of compound **7b** in $CDCl_3$. Note that the ^{13}C signal for the C-BF_2 bond does not appear.

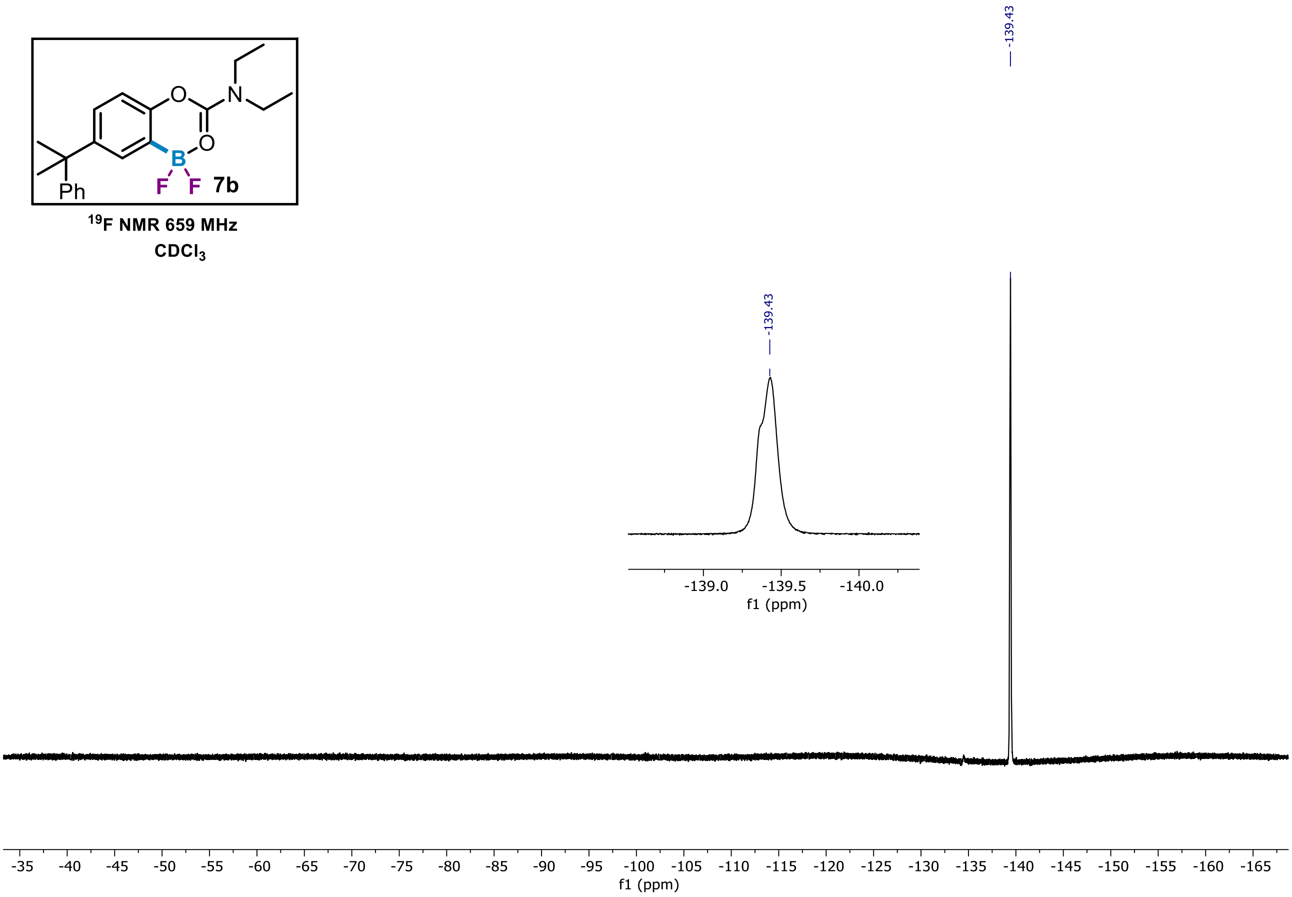
7b
^{19}F NMR 659 MHz
CDCl$_3$
-139.43
-139.0
-139.5
-140.0
f1 (ppm)
-35 -40 -45 -50 -55 -60 -65 -70 -75 -80 -85 -90 -95 -100 -105 -110 -115 -120 -125 -130 -135 -140 -145 -150 -155 -160 -165
f1 (ppm)

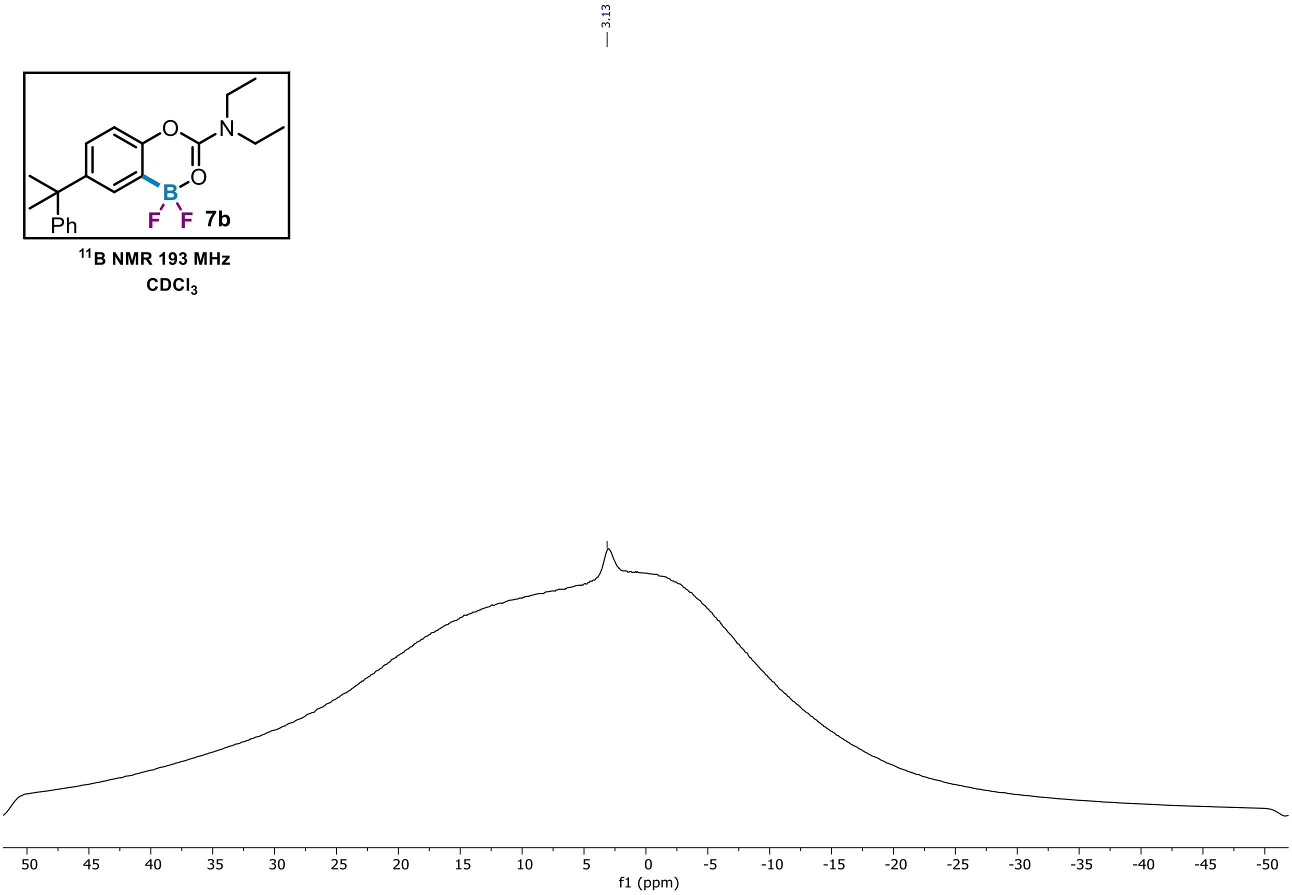
7b
^{11}B NMR 193 MHz
CDCl$_3$
3.13
50 45 40 35 30 25 20 15 10 5 0 -5 -10 -15 -20 -25 -30 -35 -40 -45 -50
f1 (ppm)

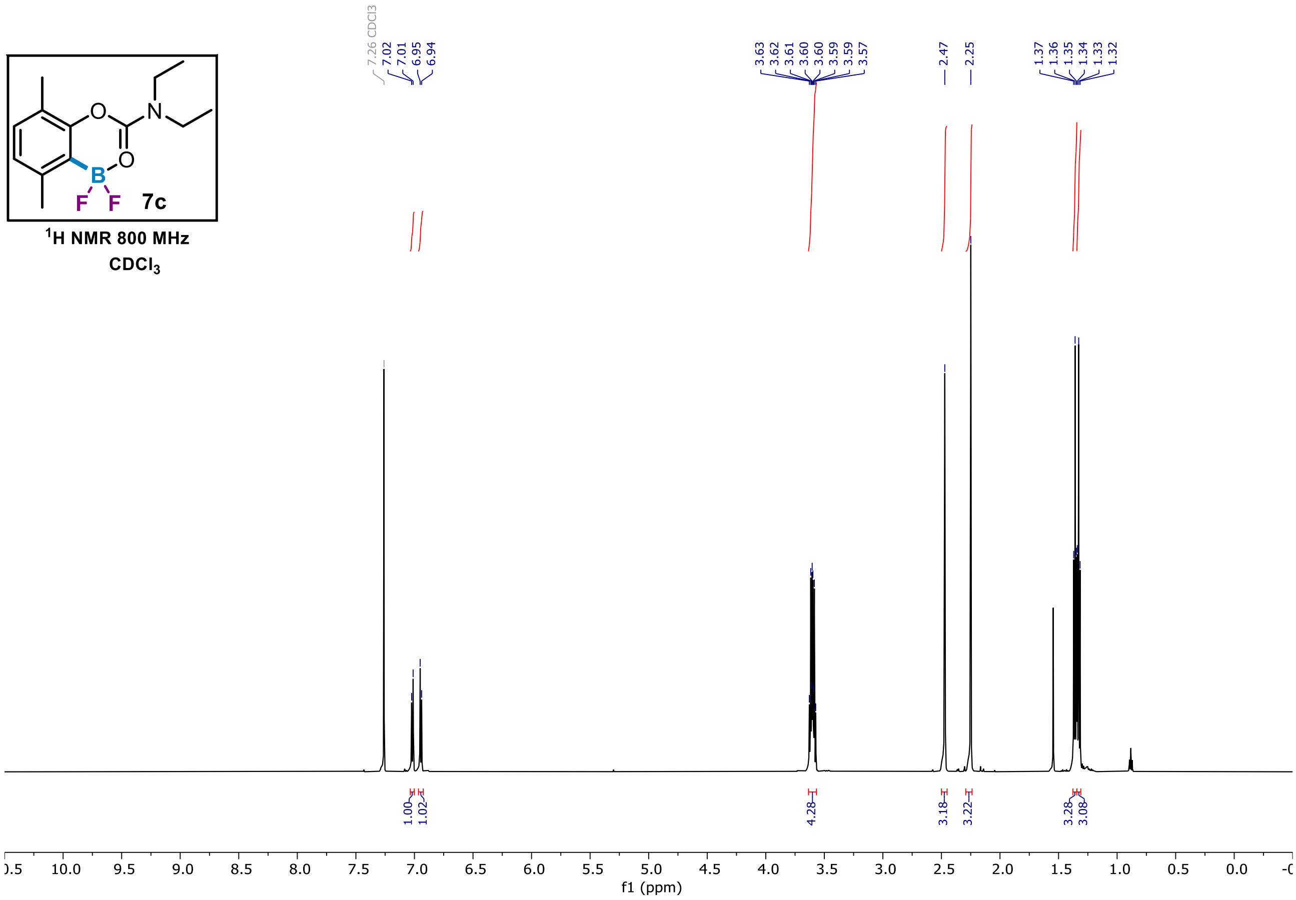

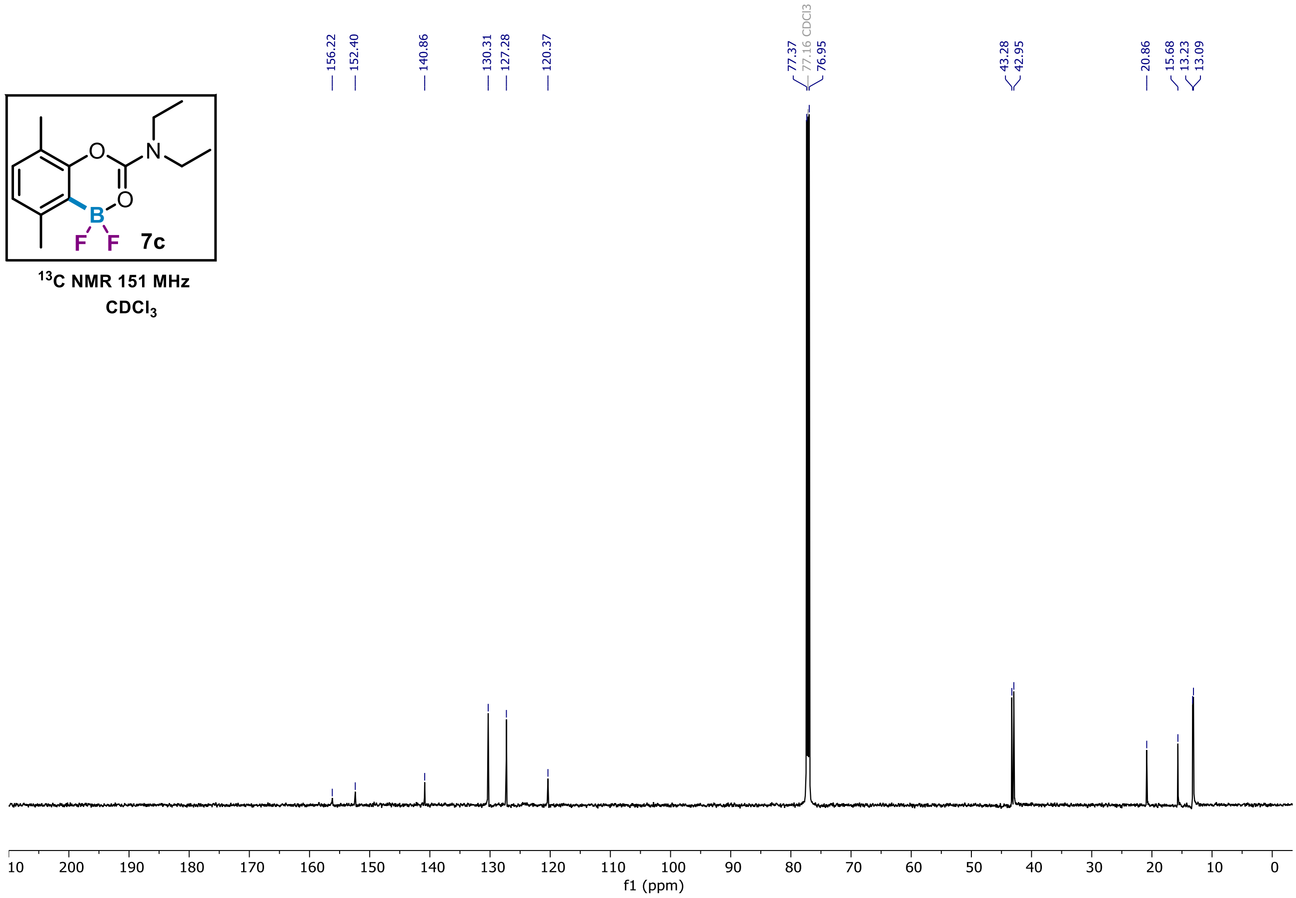

Figure S11-52: ^{13}C spectrum of compound **7c** in $CDCl_3$. Note that the ^{13}C signal for the C-BF_2 bond does not appear.

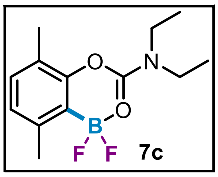

^{19}F NMR 659 MHz
$CDCl_3$

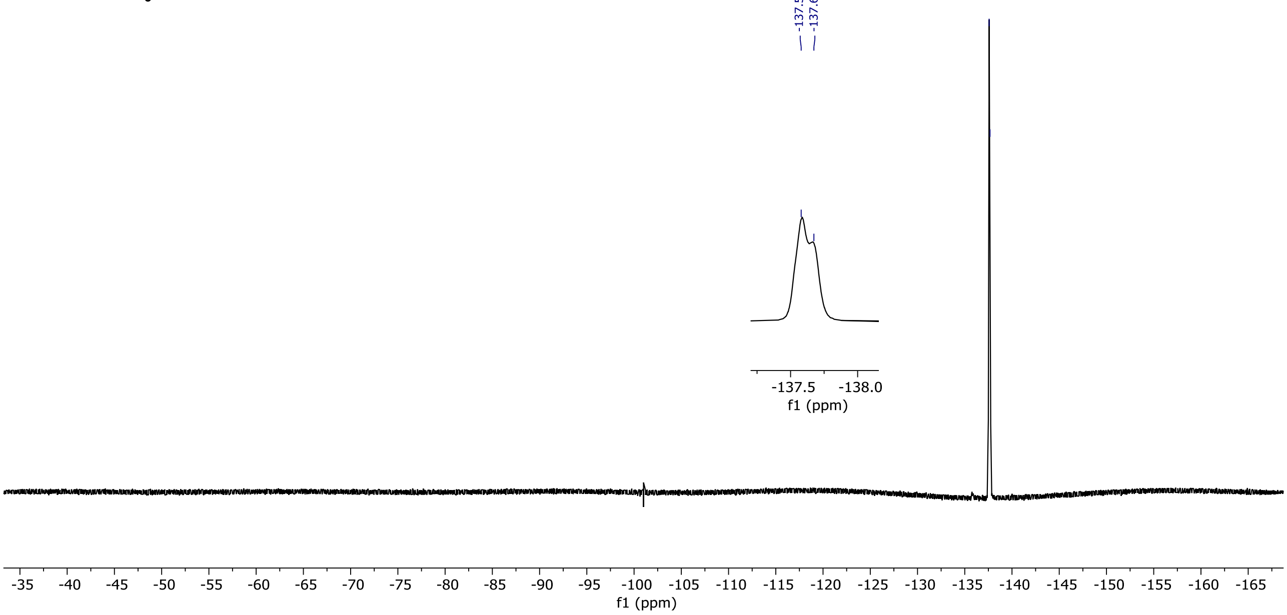

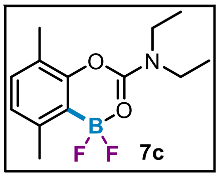

^{11}B NMR 193 MHz
$CDCl_3$

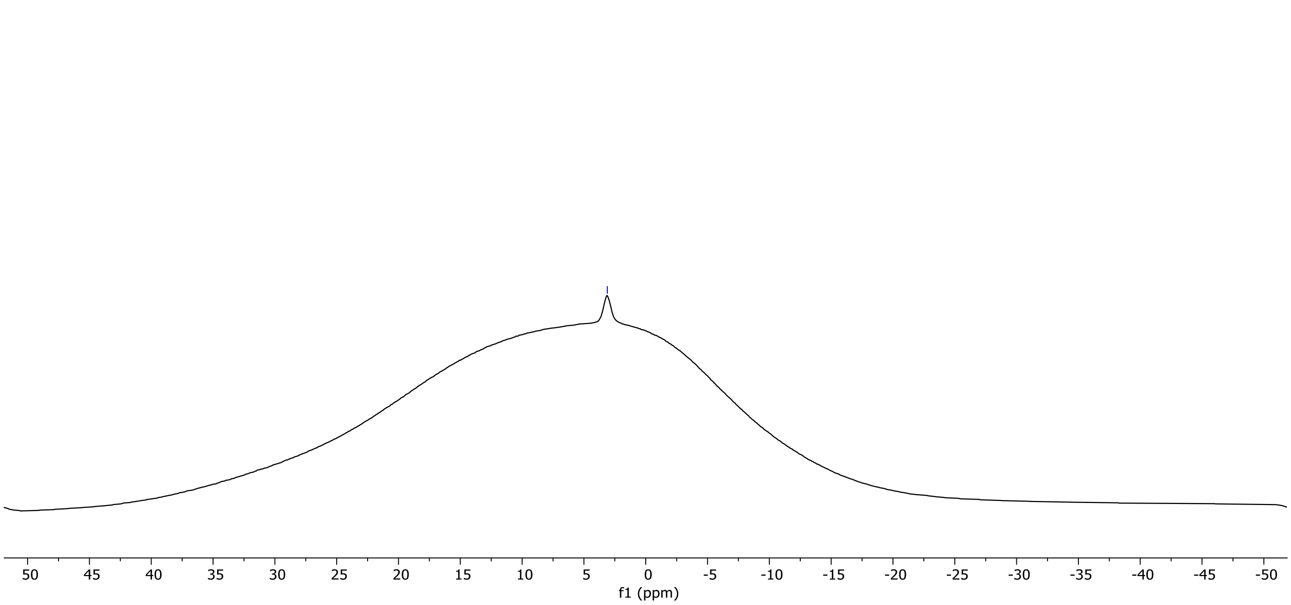

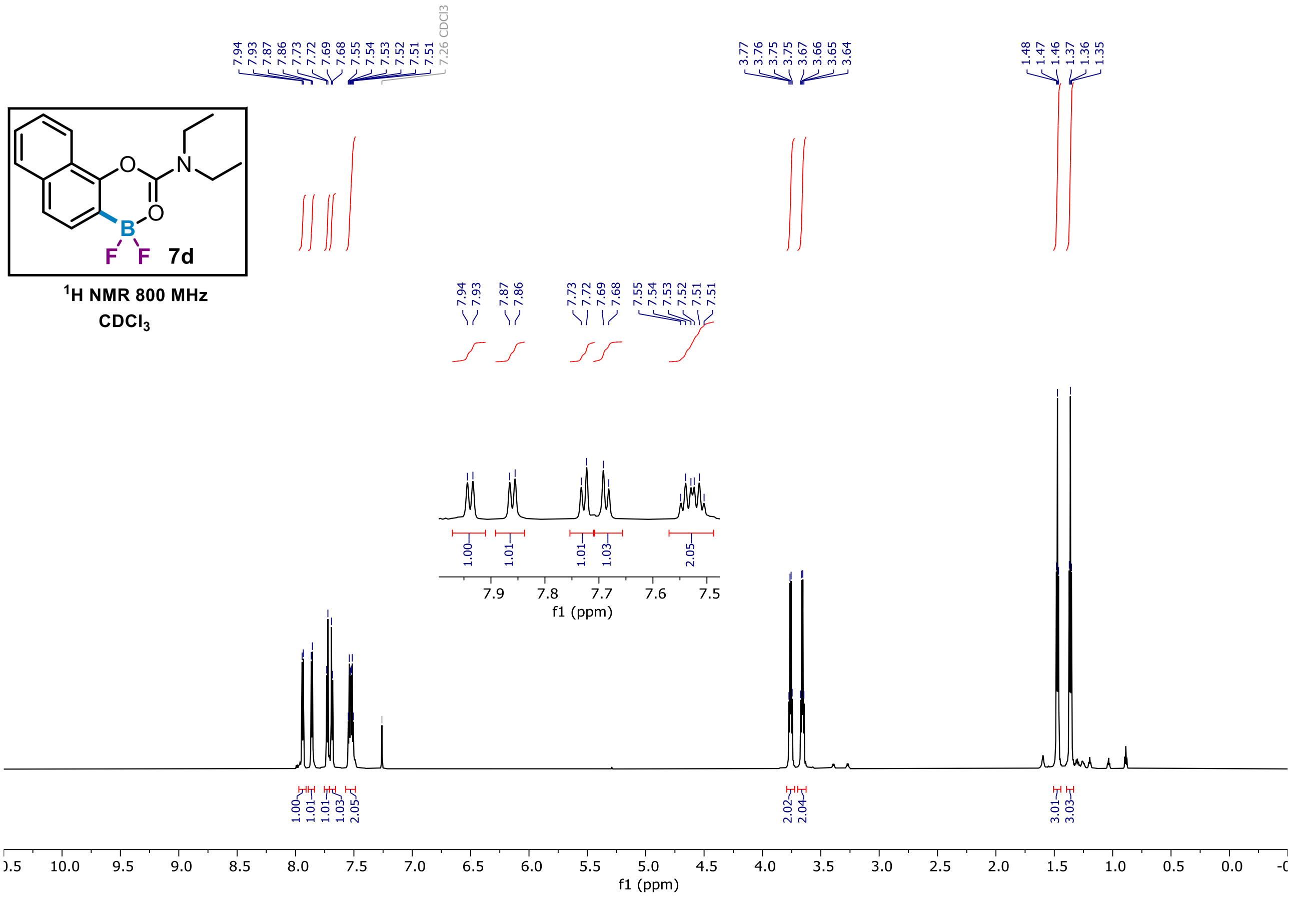

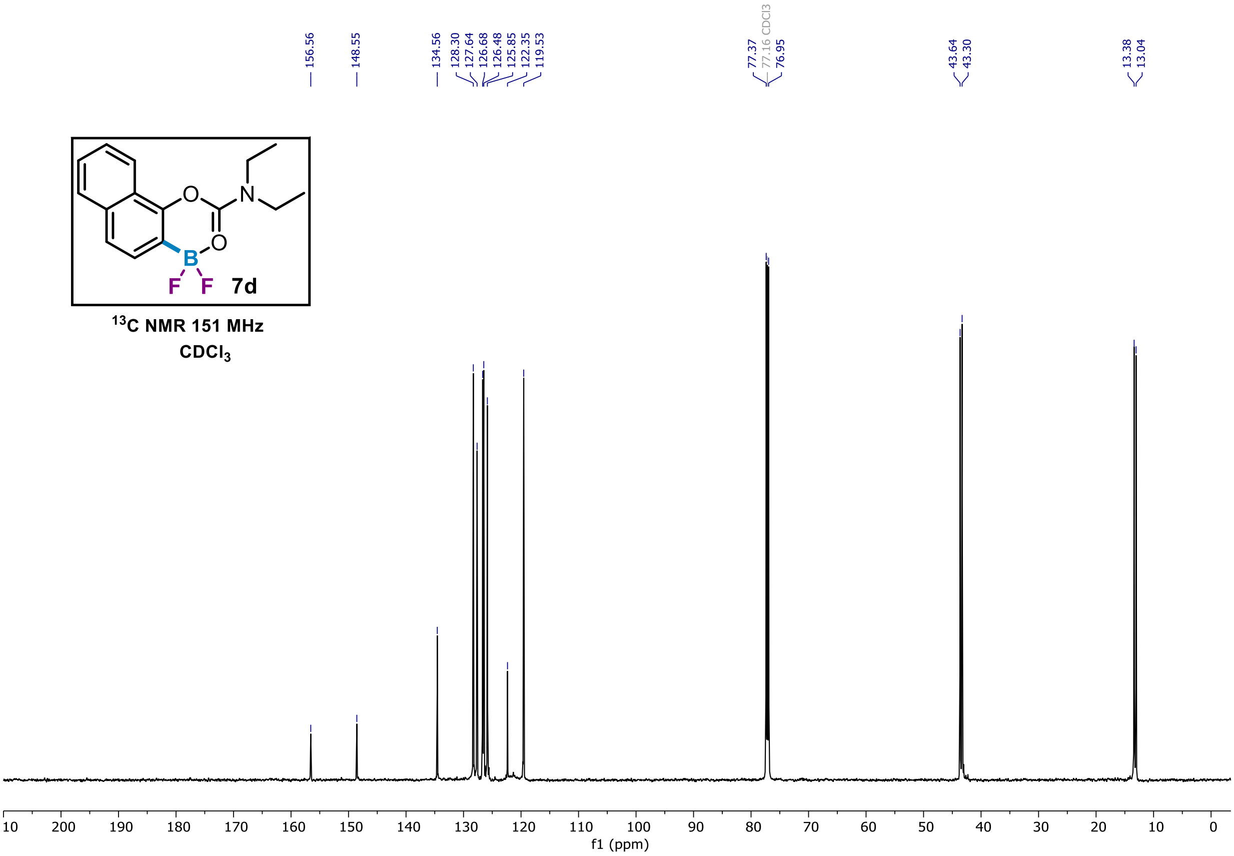

Figure S11-53: ^{13}C spectrum of compound **7d** in $CDCl_3$. Note that the ^{13}C signal for the C-BF_2 bond does not appear.

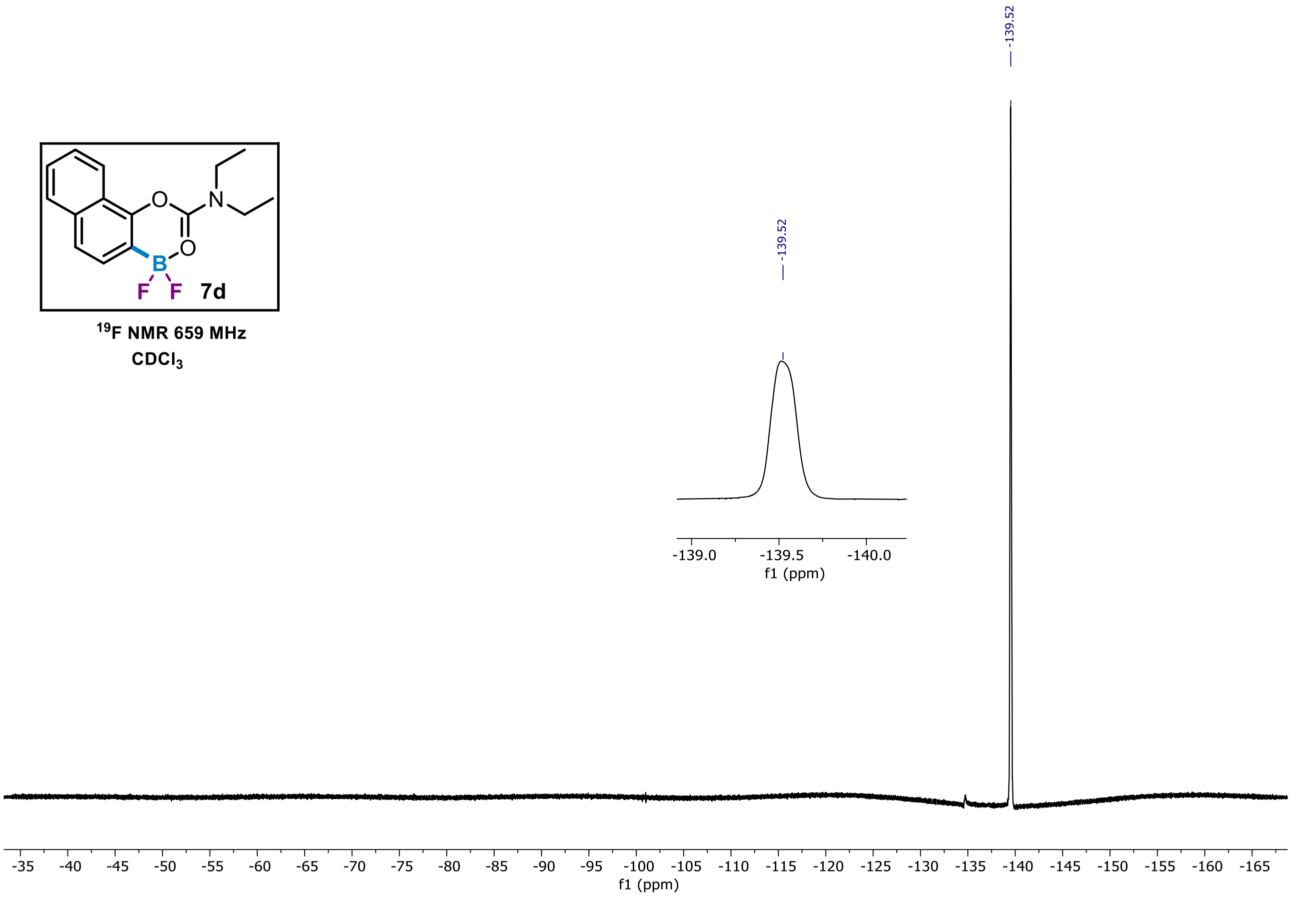

^{19}F NMR 659 MHz
$CDCl_3$

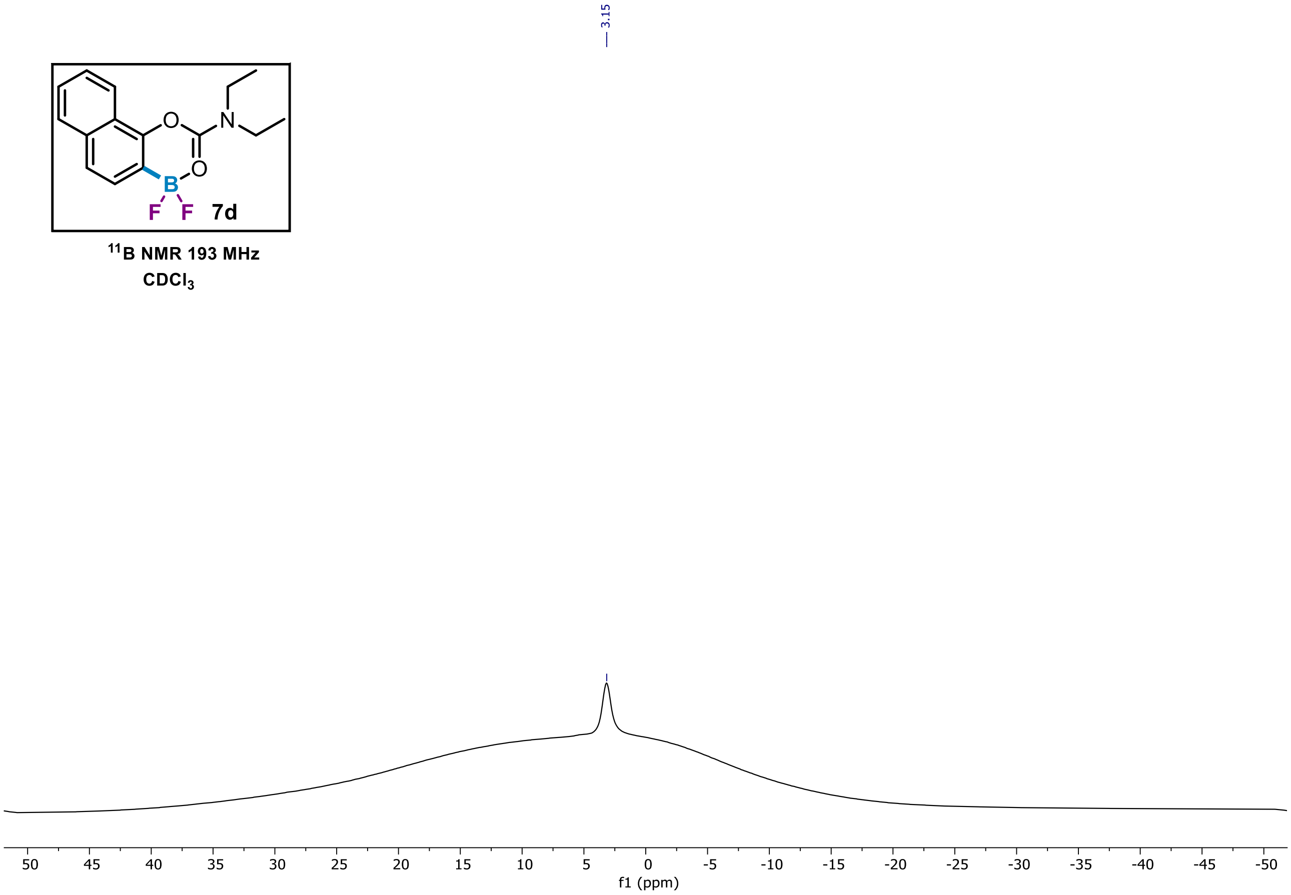

^{11}B NMR 193 MHz
$CDCl_3$

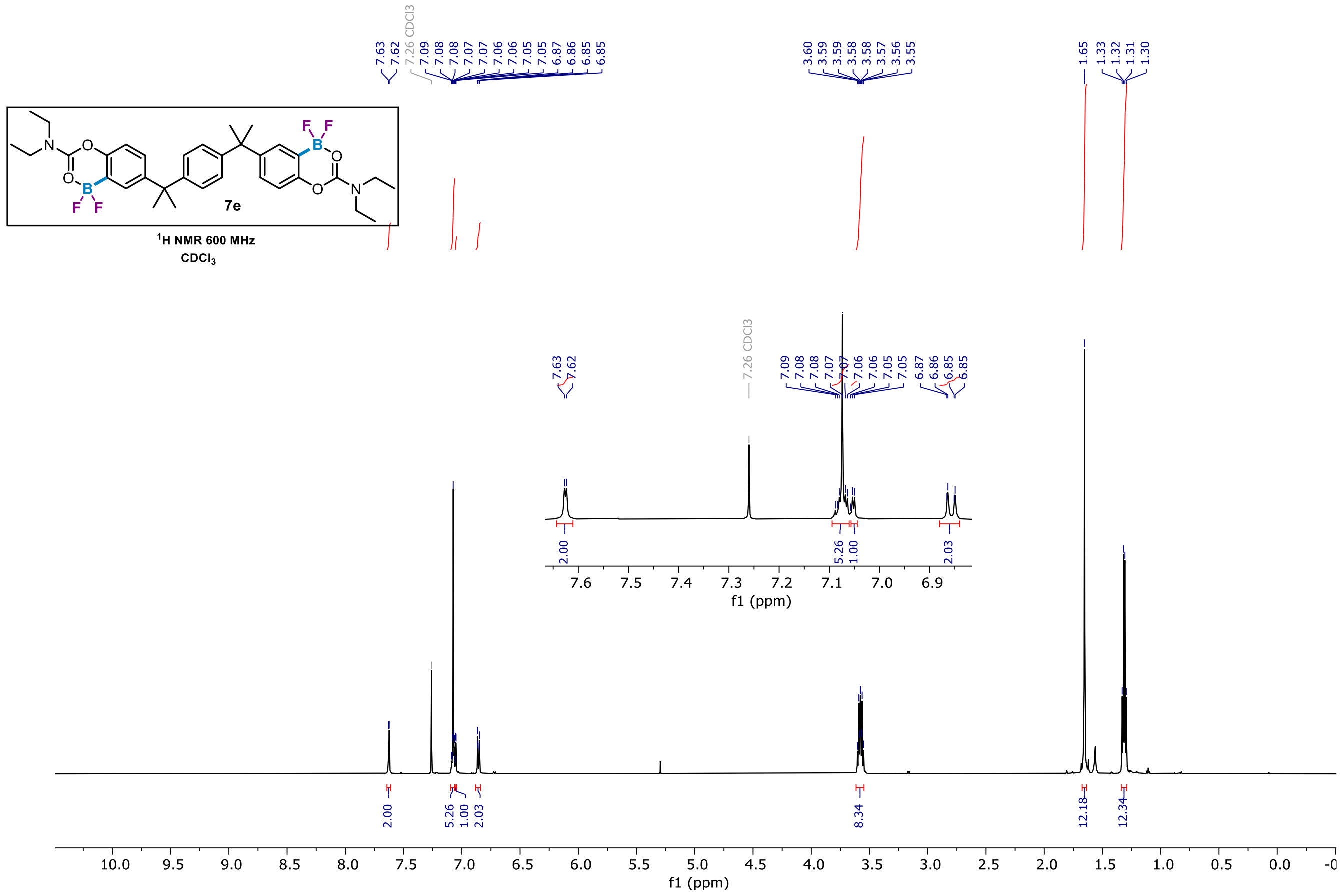

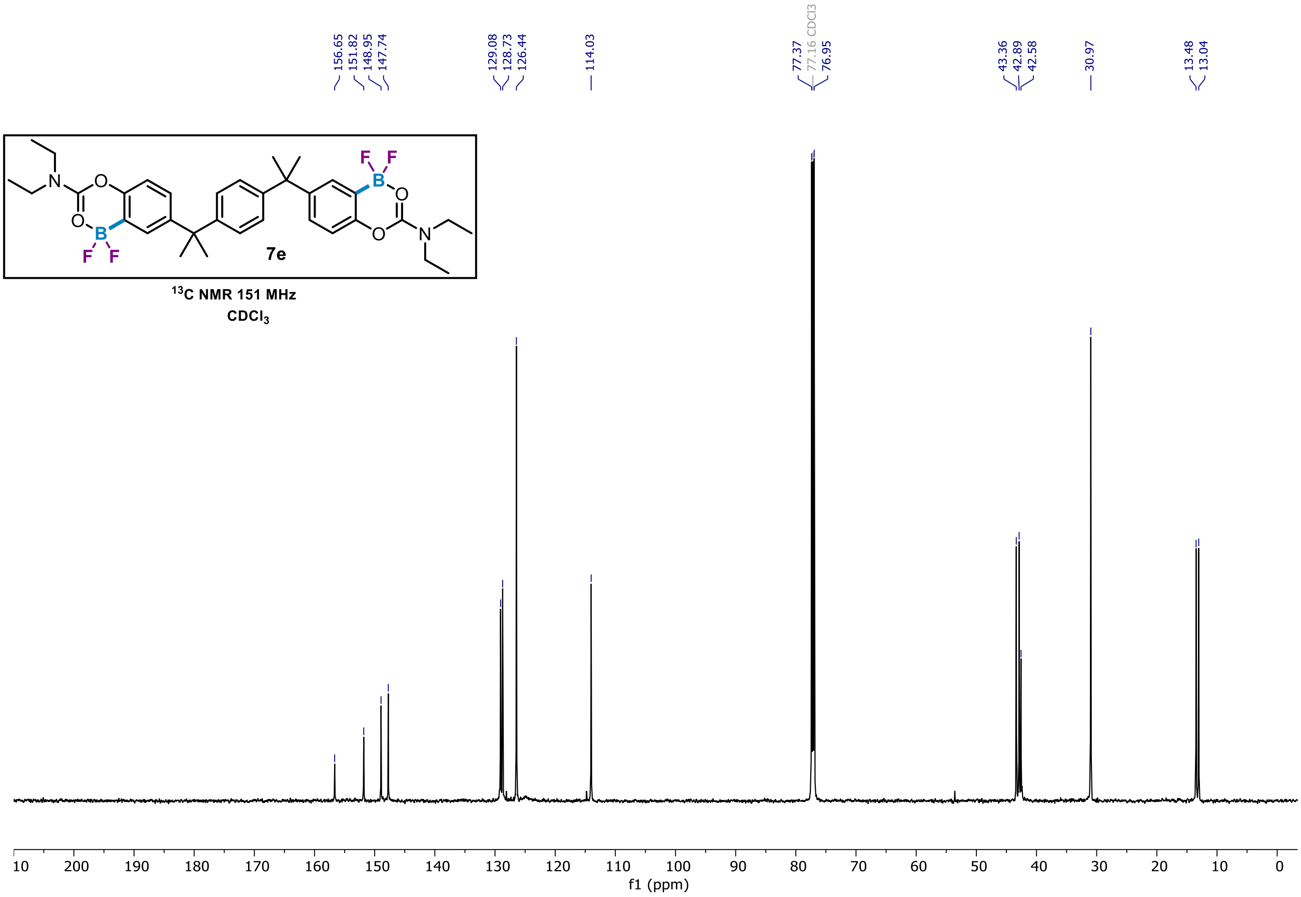

Figure S11-54: ^{13}C spectrum of compound **7e** in $CDCl_3$. Note that the ^{13}C signal for the C-BF_2 bond does not appear.

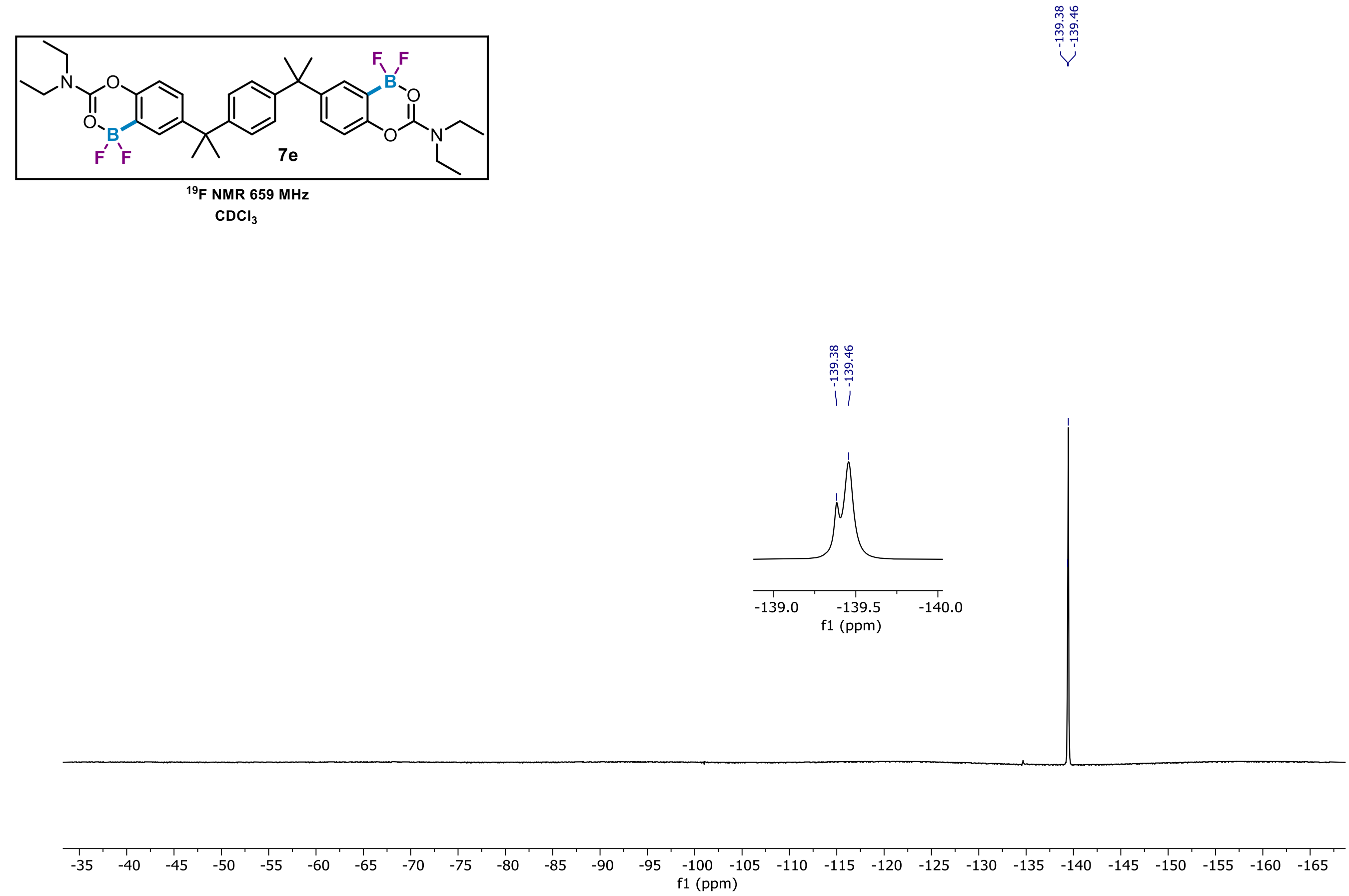
7e
19F NMR 659 MHz
CDCl3
-139.38
-139.46
-139.0
-139.5
-140.0
f1 (ppm)
-35 -40 -45 -50 -55 -60 -65 -70 -75 -80 -85 -90 -95 -100 -105 -110 -115 -120 -125 -130 -135 -140 -145 -150 -155 -160 -165
f1 (ppm)

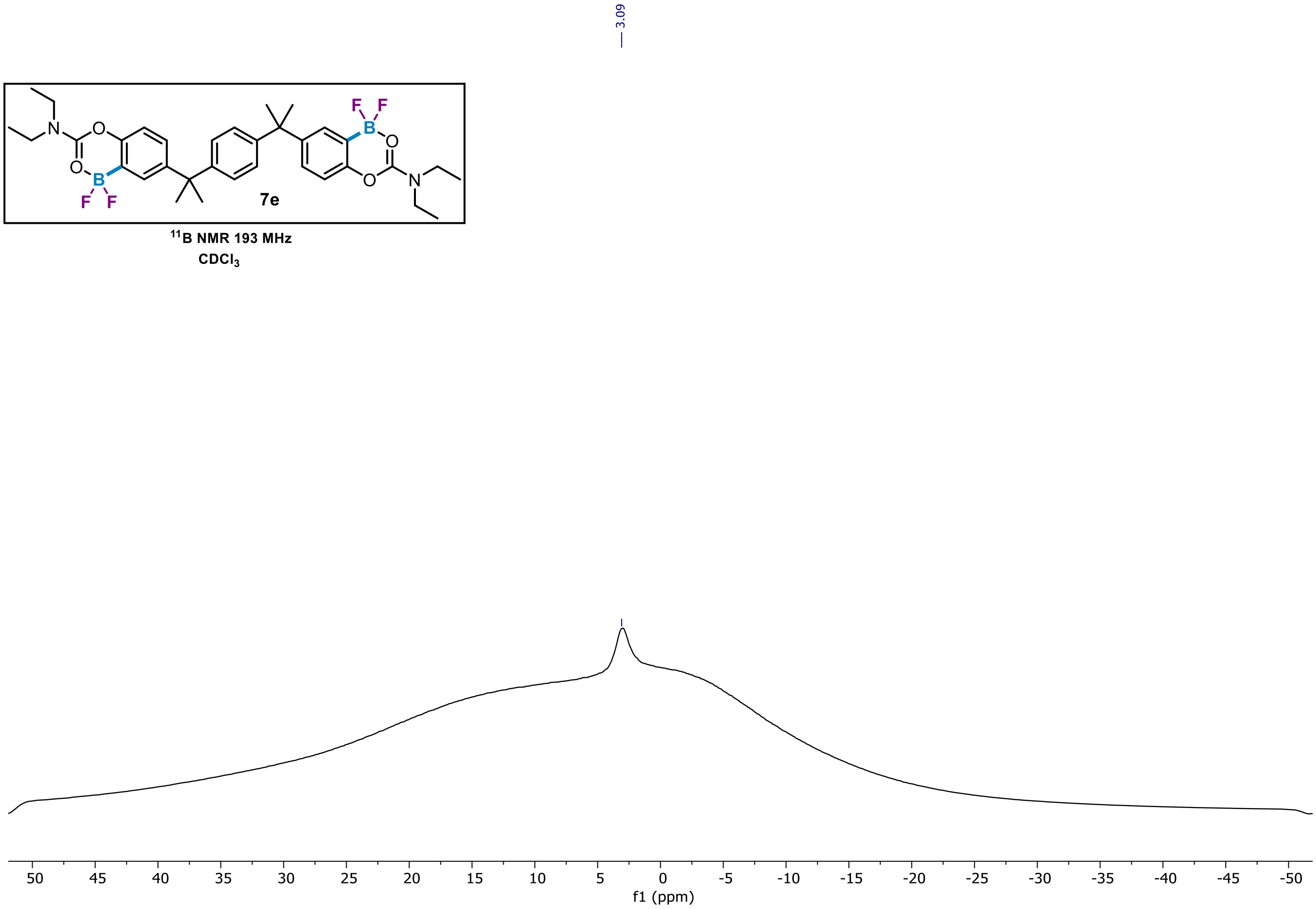
3.09
7e
11B NMR 193 MHz
CDCl3
50 45 40 35 30 25 20 15 10 5 0 -5 -10 -15 -20 -25 -30 -35 -40 -45 -50
f1 (ppm)

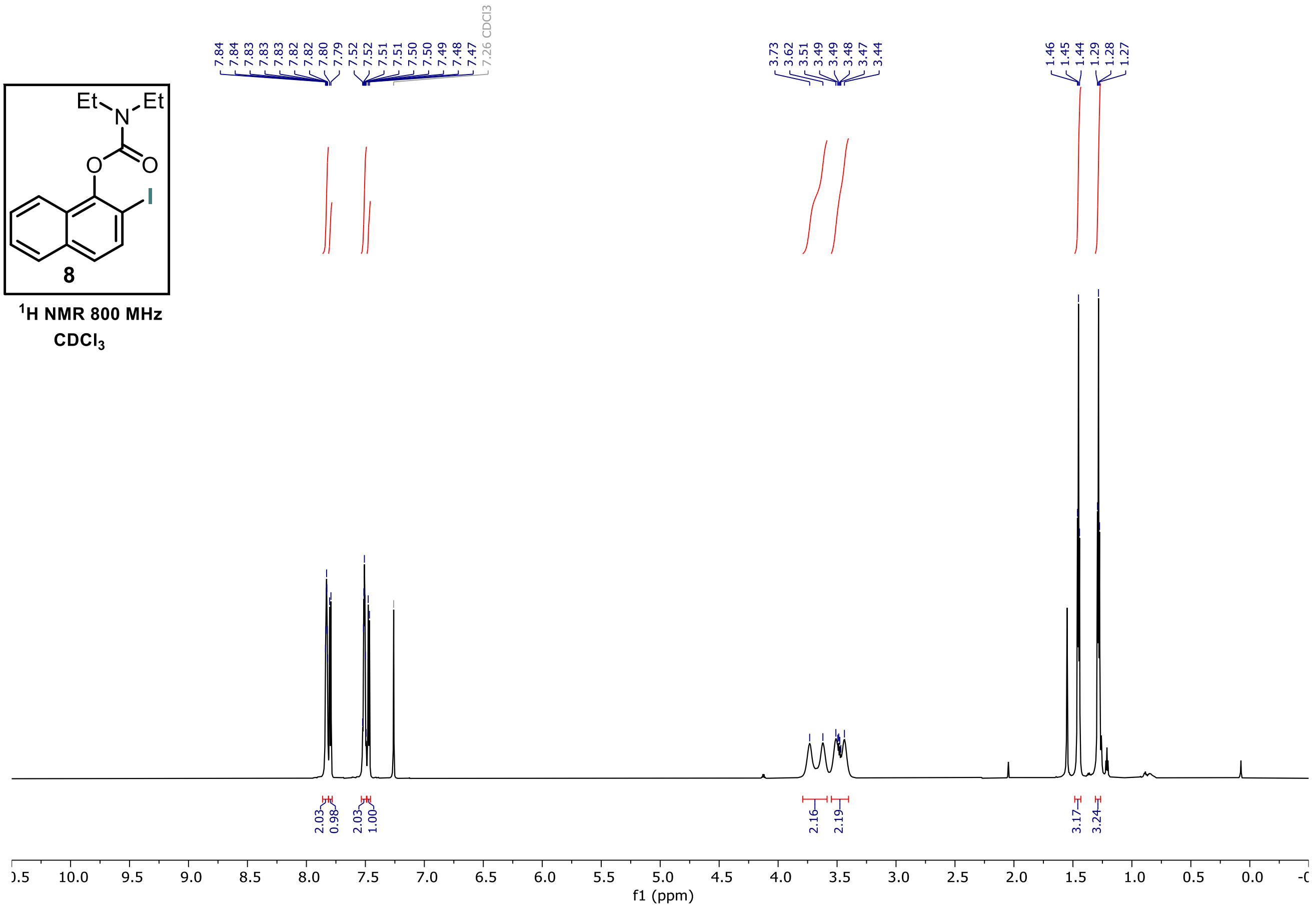
7.84 7.84 7.83 7.83 7.83 7.82 7.82 7.80 7.79 7.52 7.52 7.51 7.51 7.50 7.49 7.48 7.47 7.26 CDCl3
3.73 3.62 3.51 3.49 3.49 3.48 3.47 3.44
1.46 1.45 1.44 1.29 1.28 1.27
Et N Et
O O
I
8
^{1}H NMR 800 MHz
$CDCl_3$
2.03 0.98 2.03 1.00
2.16 2.19
3.17 3.24
f1 (ppm)

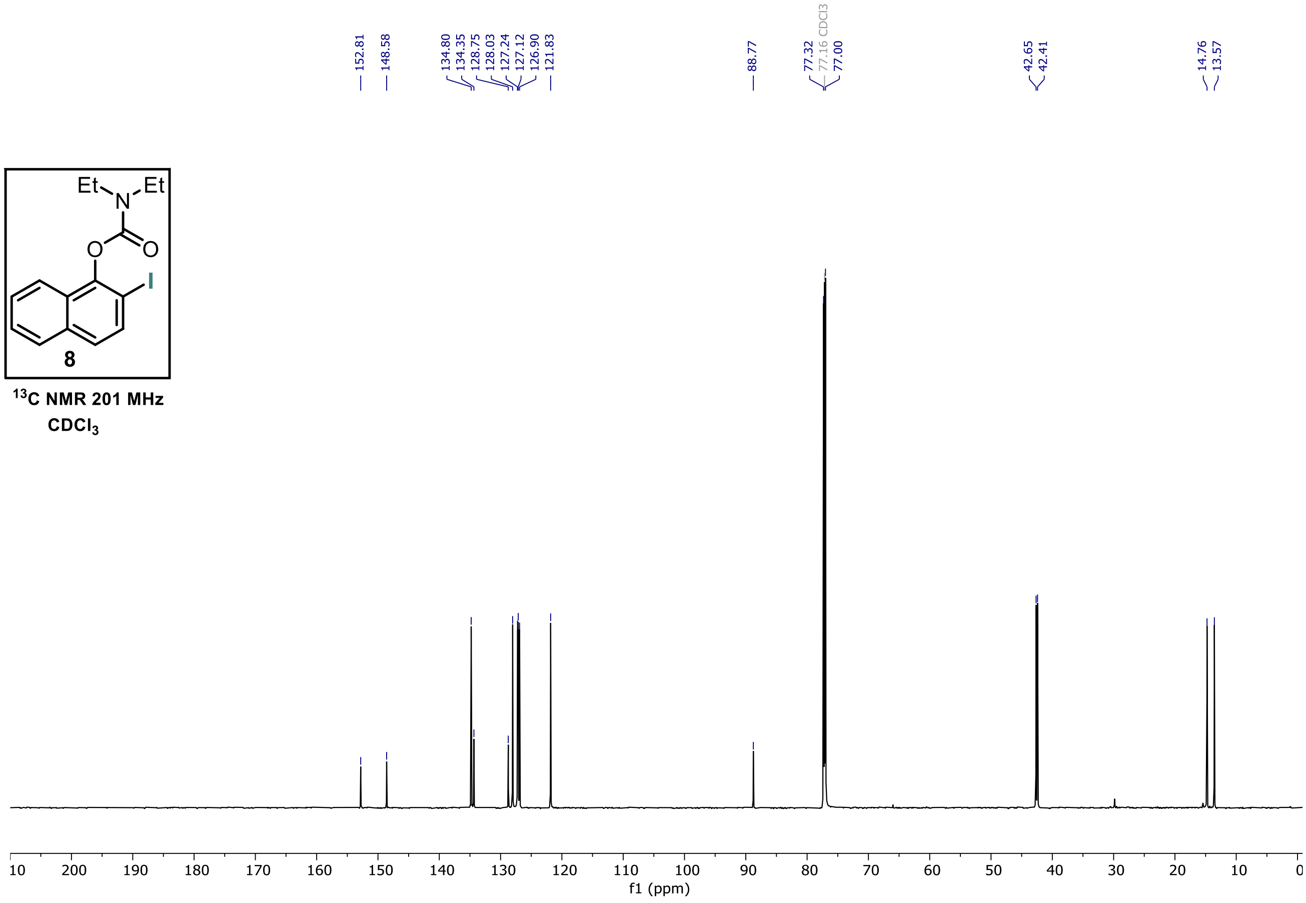
152.81 148.58
134.80 134.35 128.75 128.03 127.24 127.12 126.90 121.83
88.77
77.32 77.16 CDCl3 77.00
42.65 42.41
14.76 13.57
Et N Et
O O
I
8
^{13}C NMR 201 MHz
$CDCl_3$
f1 (ppm)

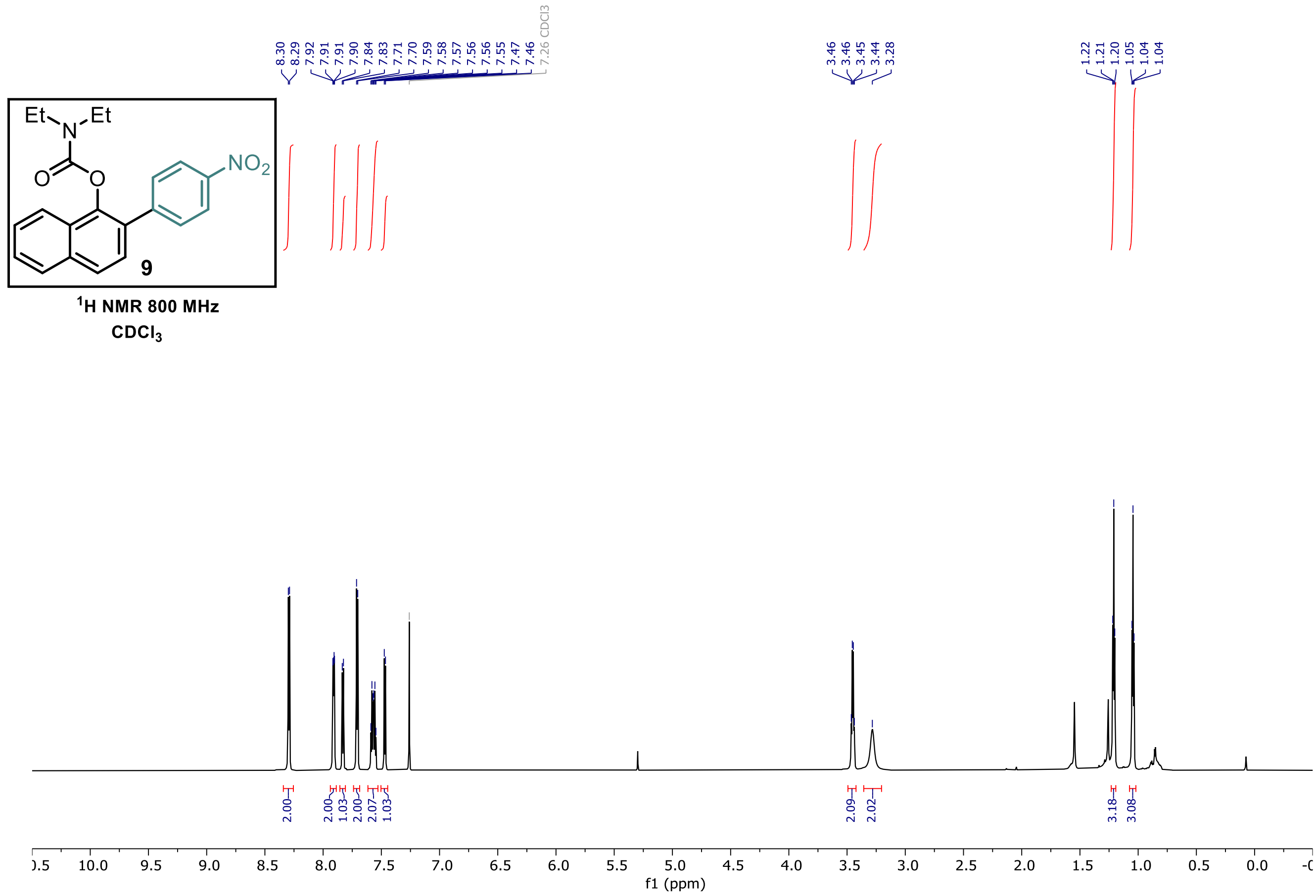
8.30
8.29
7.92
7.91
7.91
7.90
7.84
7.83
7.71
7.70
7.59
7.58
7.57
7.56
7.56
7.55
7.47
7.46
7.26 CDCl3
3.46
3.46
3.45
3.44
3.28
1.22
1.21
1.20
1.05
1.04
1.04
Et
N
Et
O
O
NO2
9
1H NMR 800 MHz
CDCl3
2.00
2.00
1.03
2.00
2.07
1.03
2.09
2.02
3.18
3.08
f1 (ppm)

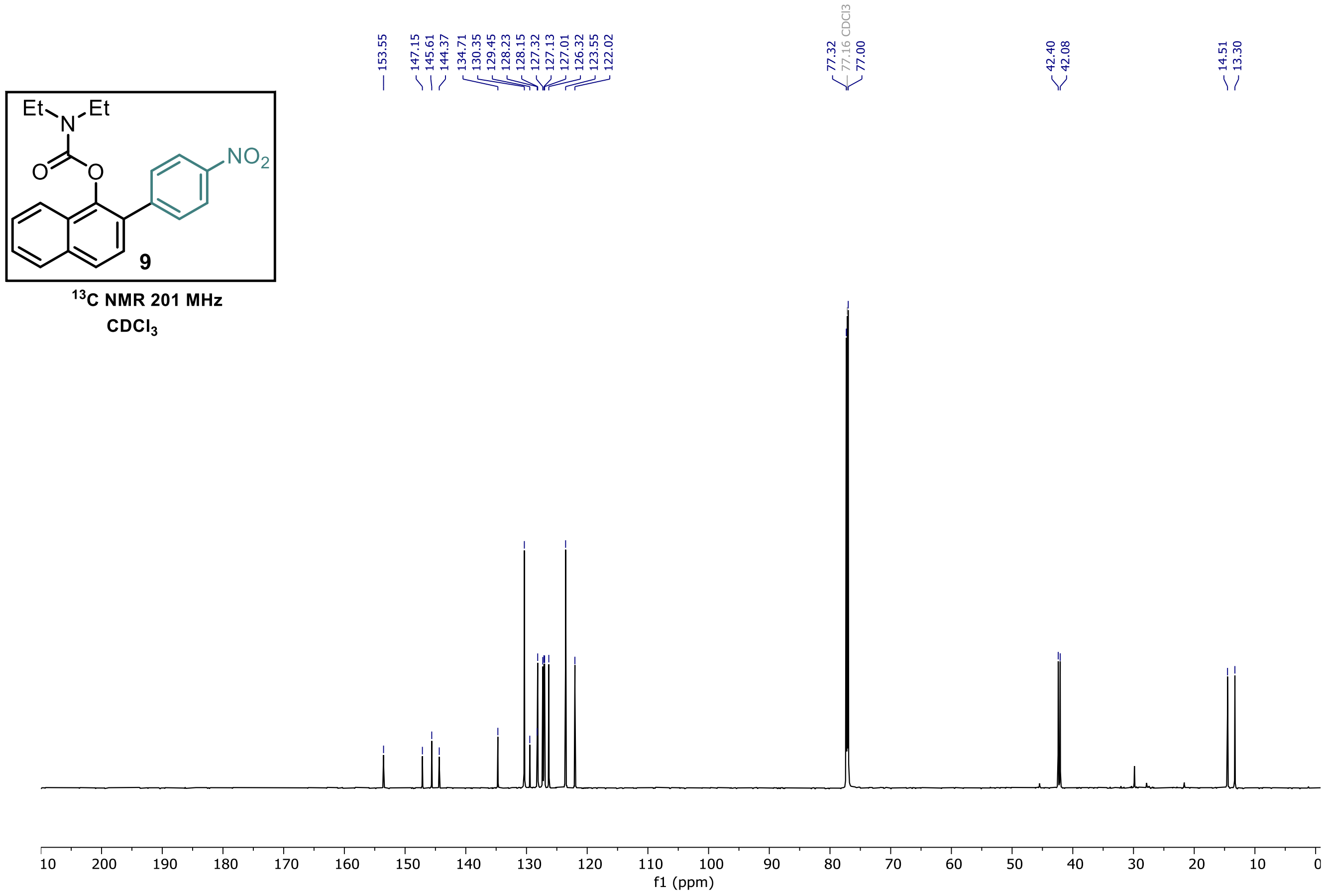
153.55
147.15
145.61
144.37
134.71
130.35
129.45
128.23
128.15
127.32
127.13
127.01
126.32
123.55
122.02
77.32
77.16 CDCl3
77.00
42.40
42.08
14.51
13.30
Et
N
Et
O
O
NO2
9
13C NMR 201 MHz
CDCl3
f1 (ppm)

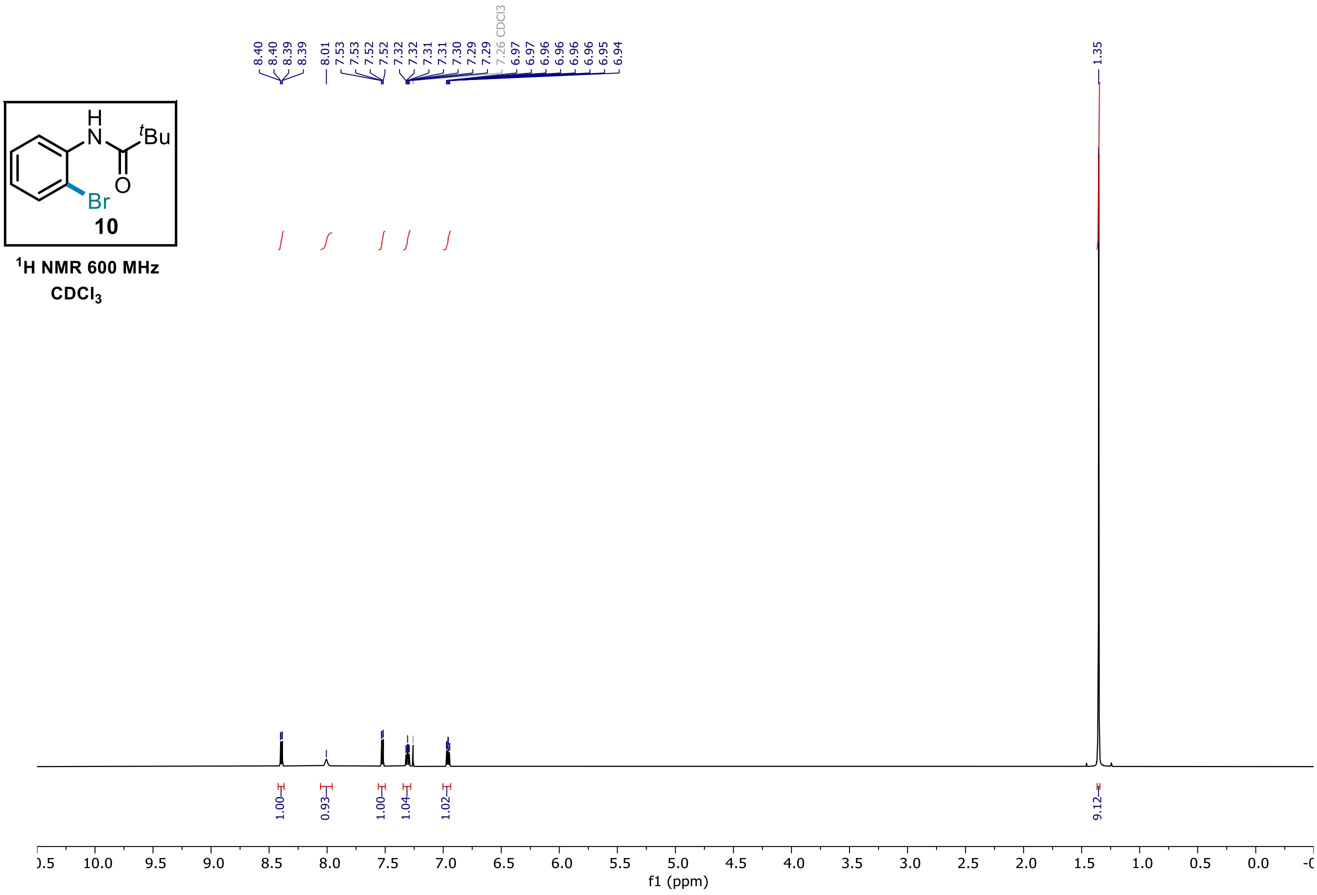
H
N
tBu
O
Br
10
1H NMR 600 MHz
CDCl3
8.40
8.40
8.39
8.39
8.01
7.53
7.53
7.52
7.52
7.32
7.32
7.31
7.31
7.30
7.29
7.29
7.26 CDCl3
6.97
6.97
6.96
6.96
6.96
6.96
6.95
6.94
1.35
1.00
0.93
1.00
1.04
1.02
9.12
f1 (ppm)

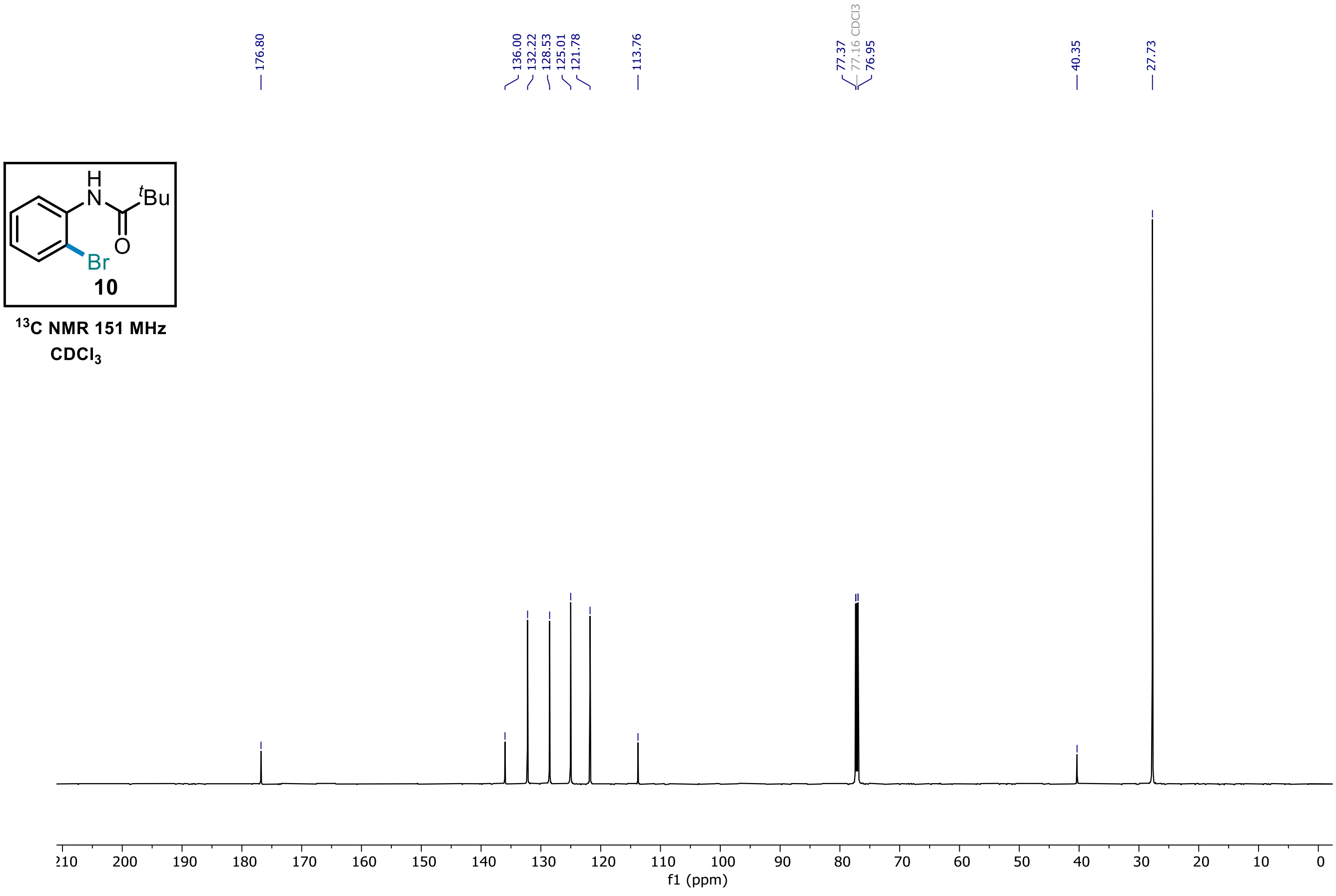
H
N
tBu
O
Br
10
13C NMR 151 MHz
CDCl3
176.80
136.00
132.22
128.53
125.01
121.78
113.76
77.37
77.16 CDCl3
76.95
40.35
27.73
f1 (ppm)

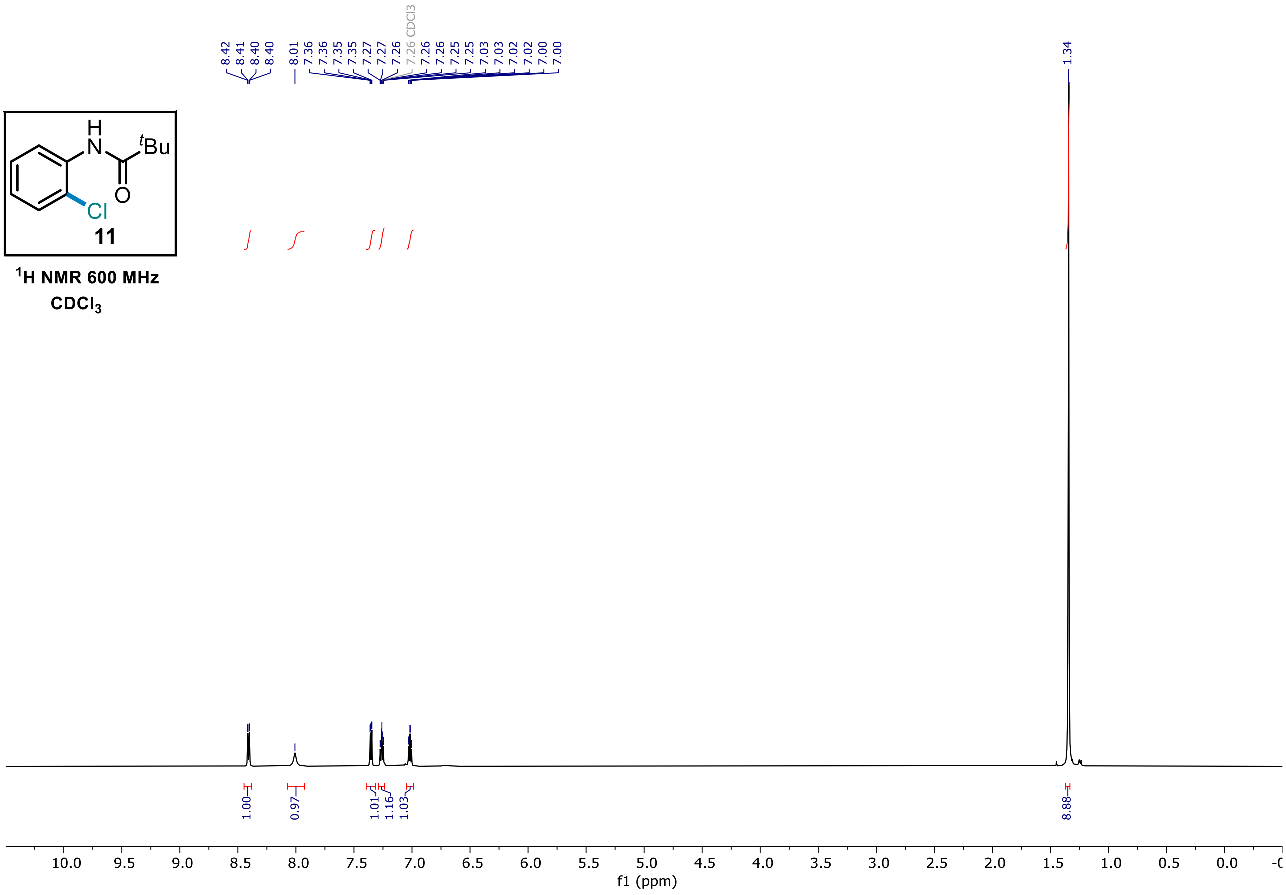
11
^{1}H NMR 600 MHz
$CDCl_3$
8.42
8.41
8.40
8.40
8.01
7.36
7.36
7.35
7.35
7.27
7.27
7.26
7.26 CDCl3
7.26
7.26
7.25
7.25
7.03
7.03
7.02
7.02
7.00
7.00
1.34
1.00
0.97
1.01
1.16
1.03
8.88
f1 (ppm)

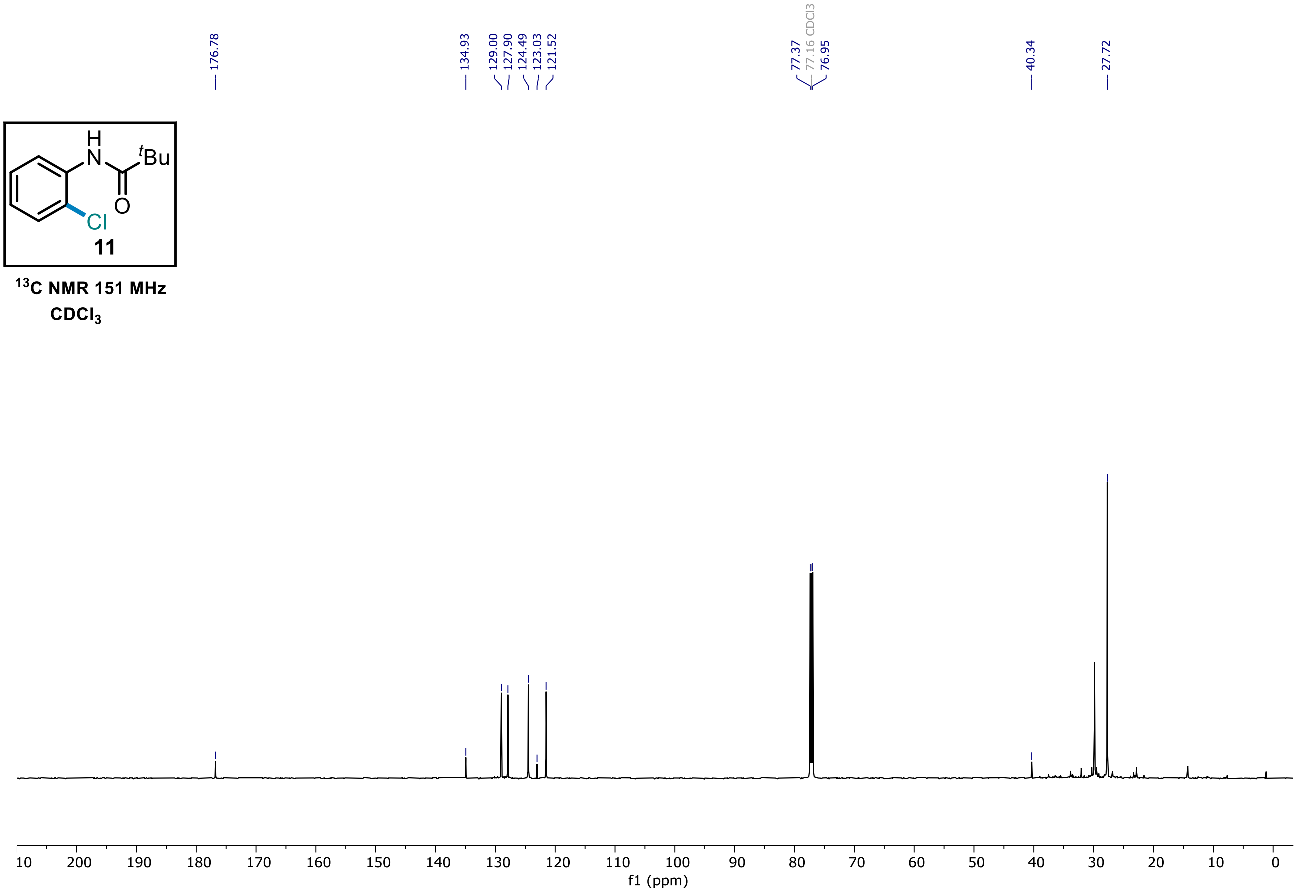
11
^{13}C NMR 151 MHz
$CDCl_3$
176.78
134.93
129.00
127.90
124.49
123.03
121.52
77.37
77.16 CDCl3
76.95
40.34
27.72
f1 (ppm)

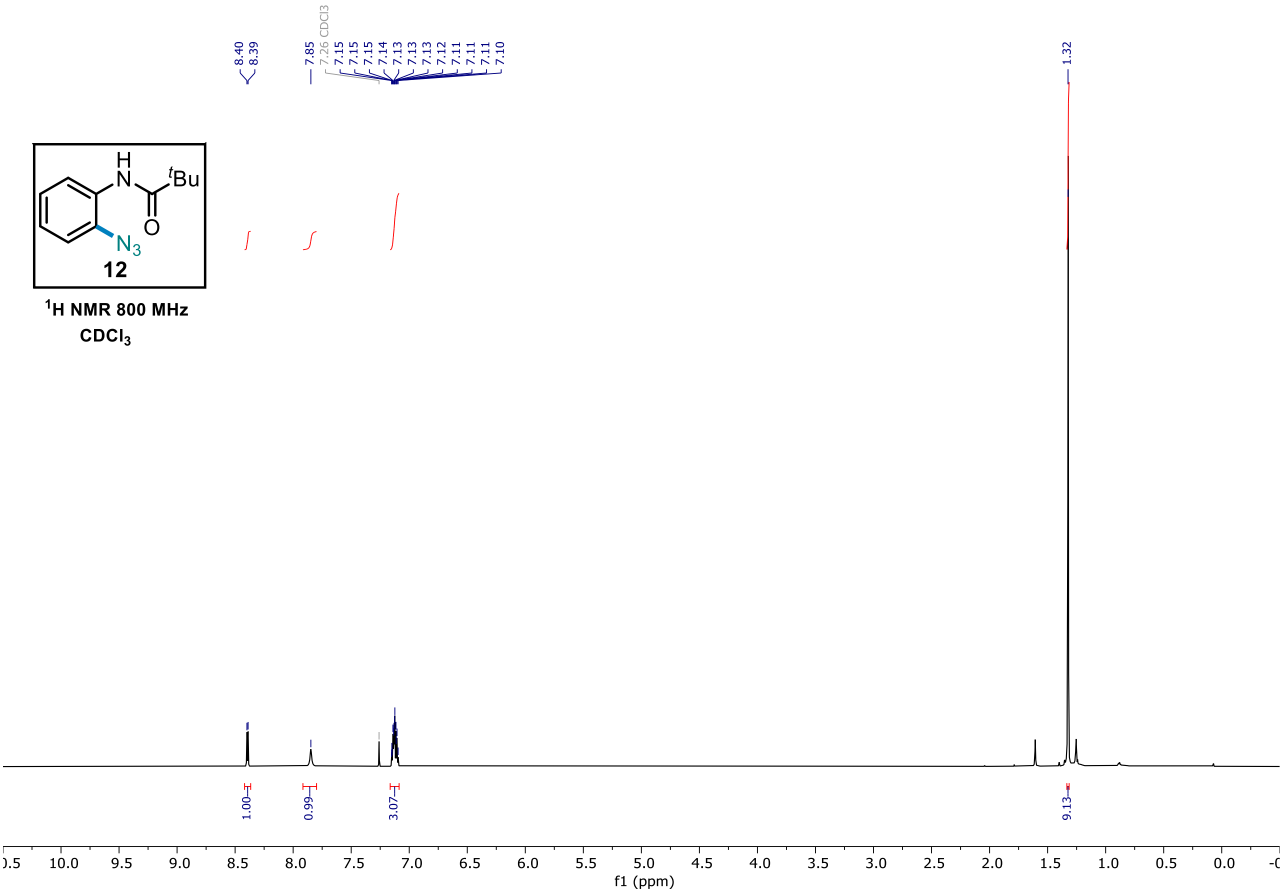
8.40
8.39
7.85
7.26 CDCl3
7.15
7.15
7.15
7.14
7.13
7.13
7.13
7.12
7.11
7.11
7.11
7.10
1.32
H
N
tBu
O
N3
12
1H NMR 800 MHz
CDCl3
1.00
0.99
3.07
9.13
f1 (ppm)

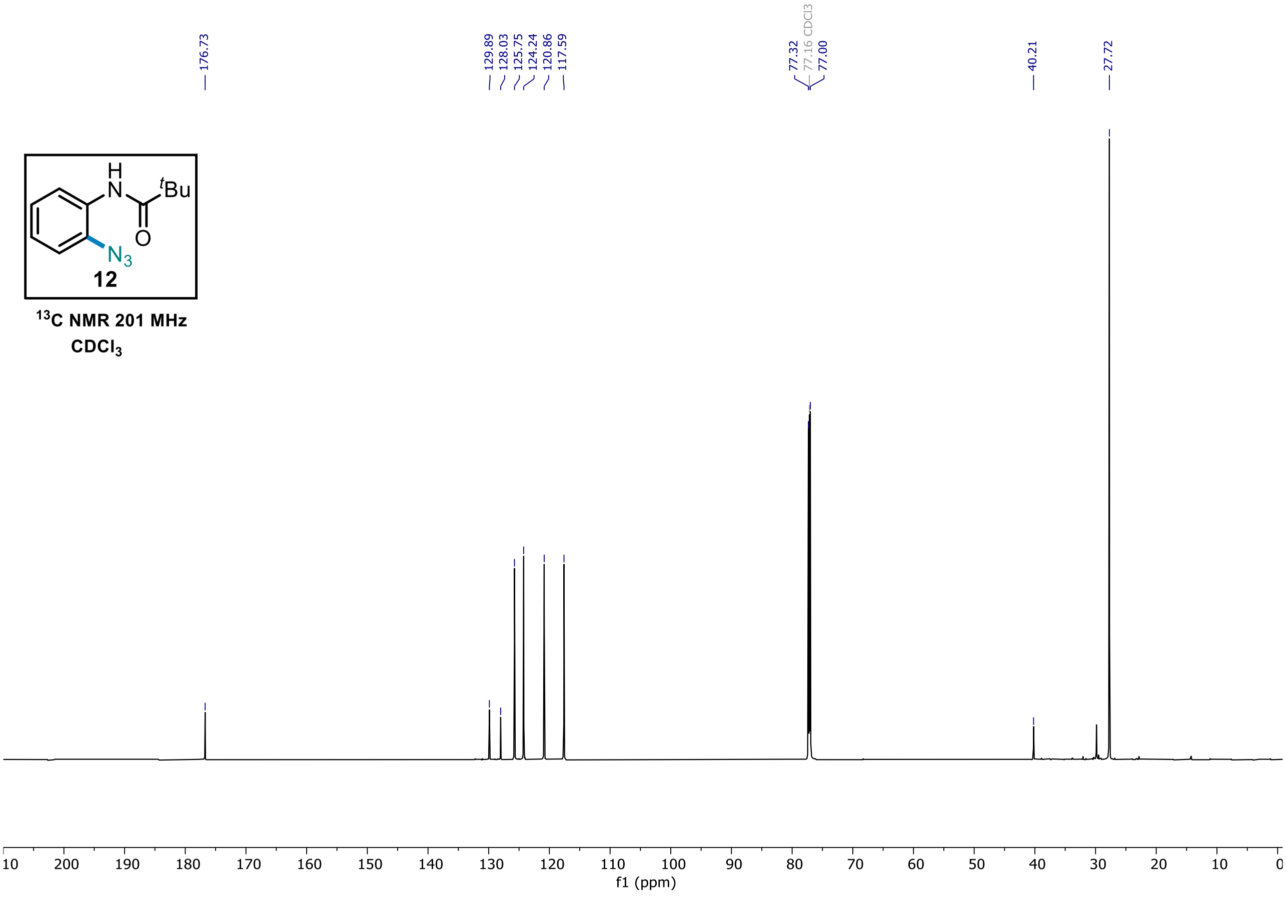
176.73
129.89
128.03
125.75
124.24
120.86
117.59
77.32
77.16 CDCl3
77.00
40.21
27.72
H
N
tBu
O
N3
12
13C NMR 201 MHz
CDCl3
f1 (ppm)

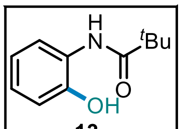

13

^{1}H NMR 800 MHz
$CDCl_3$

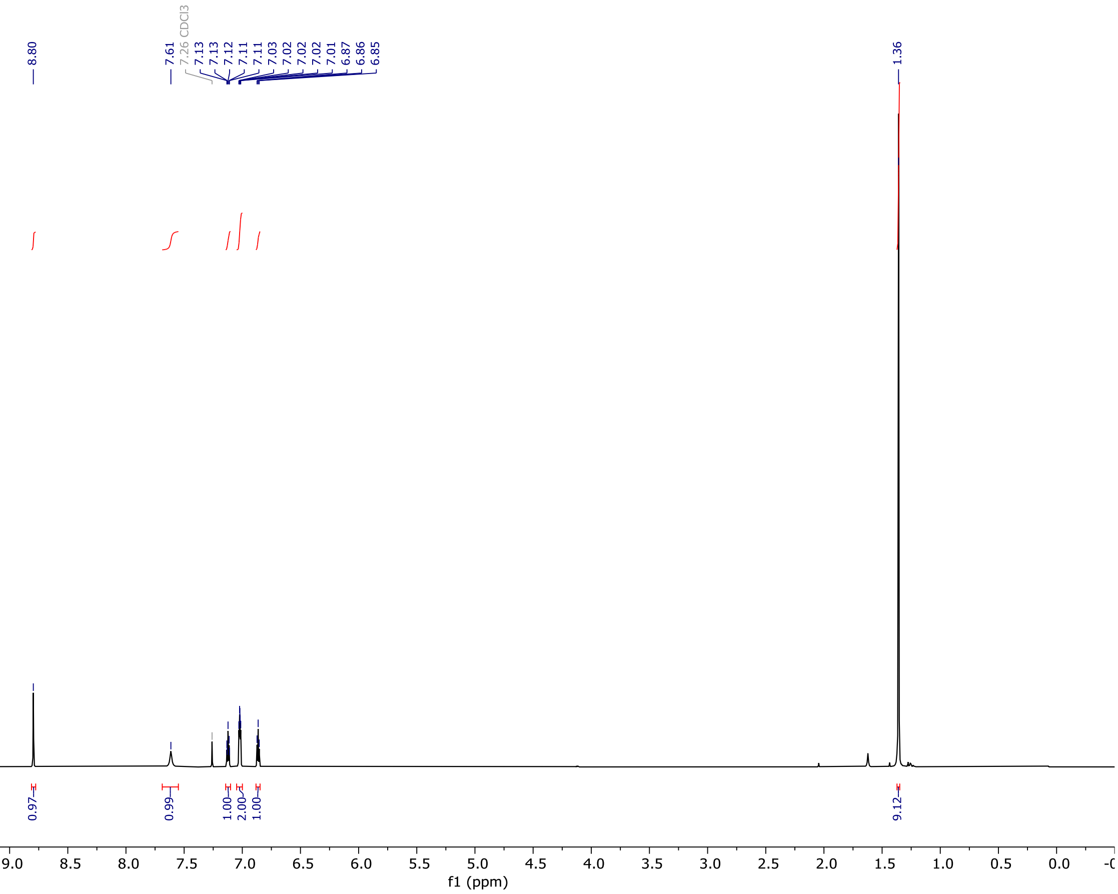

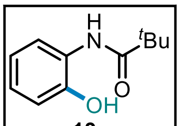

13

^{13}C NMR 201 MHz
$CDCl_3$

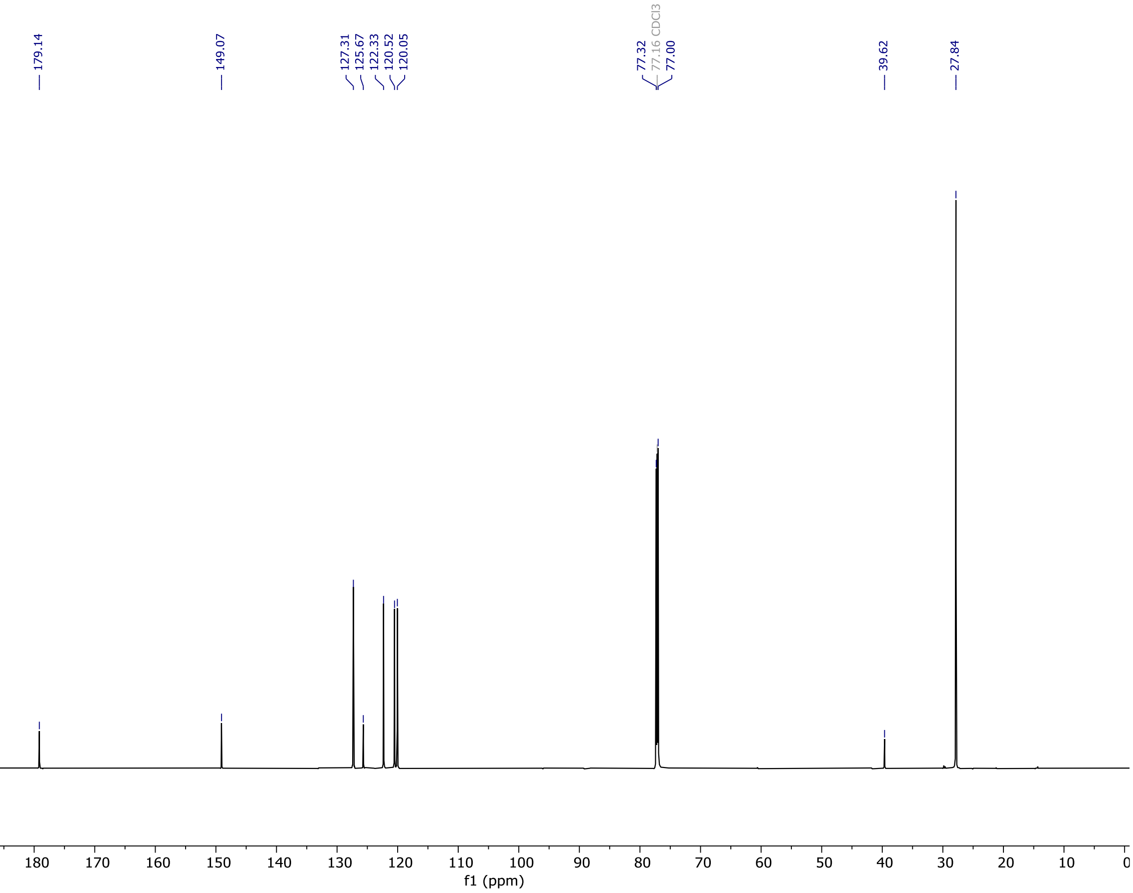

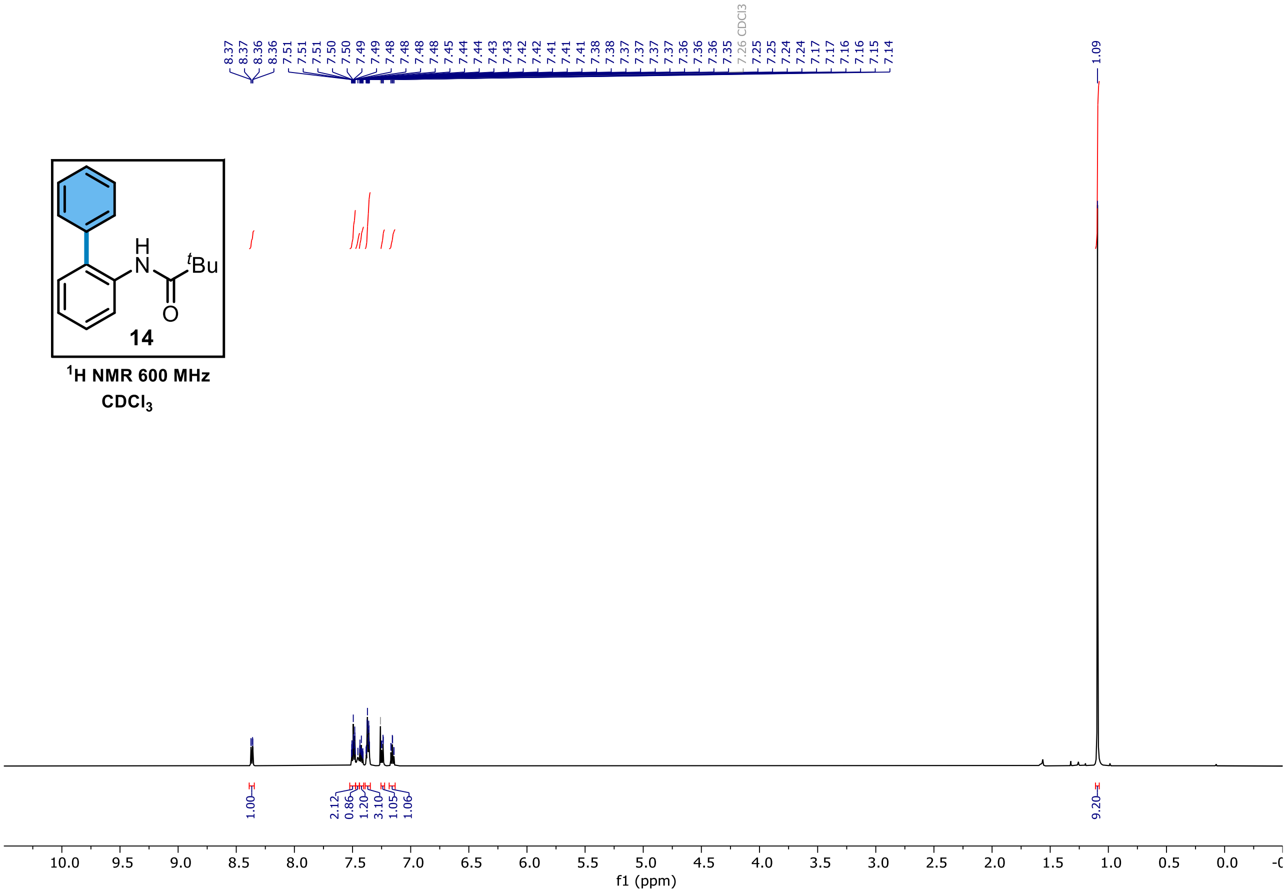
8.37 8.37 8.36 8.36 7.51 7.51 7.51 7.50 7.50 7.49 7.49 7.48 7.48 7.48 7.48 7.45 7.44 7.44 7.44 7.43 7.43 7.43 7.42 7.42 7.41 7.41 7.41 7.38 7.38 7.37 7.37 7.37 7.37 7.36 7.36 7.36 7.35 7.26 CDCl3 7.25 7.25 7.24 7.24 7.17 7.17 7.16 7.15 7.14
1.09
H
N
tBu
O
14
1H NMR 600 MHz
CDCl3
1.00
2.12 0.86 1.20 3.10 1.05 1.06
9.20
f1 (ppm)

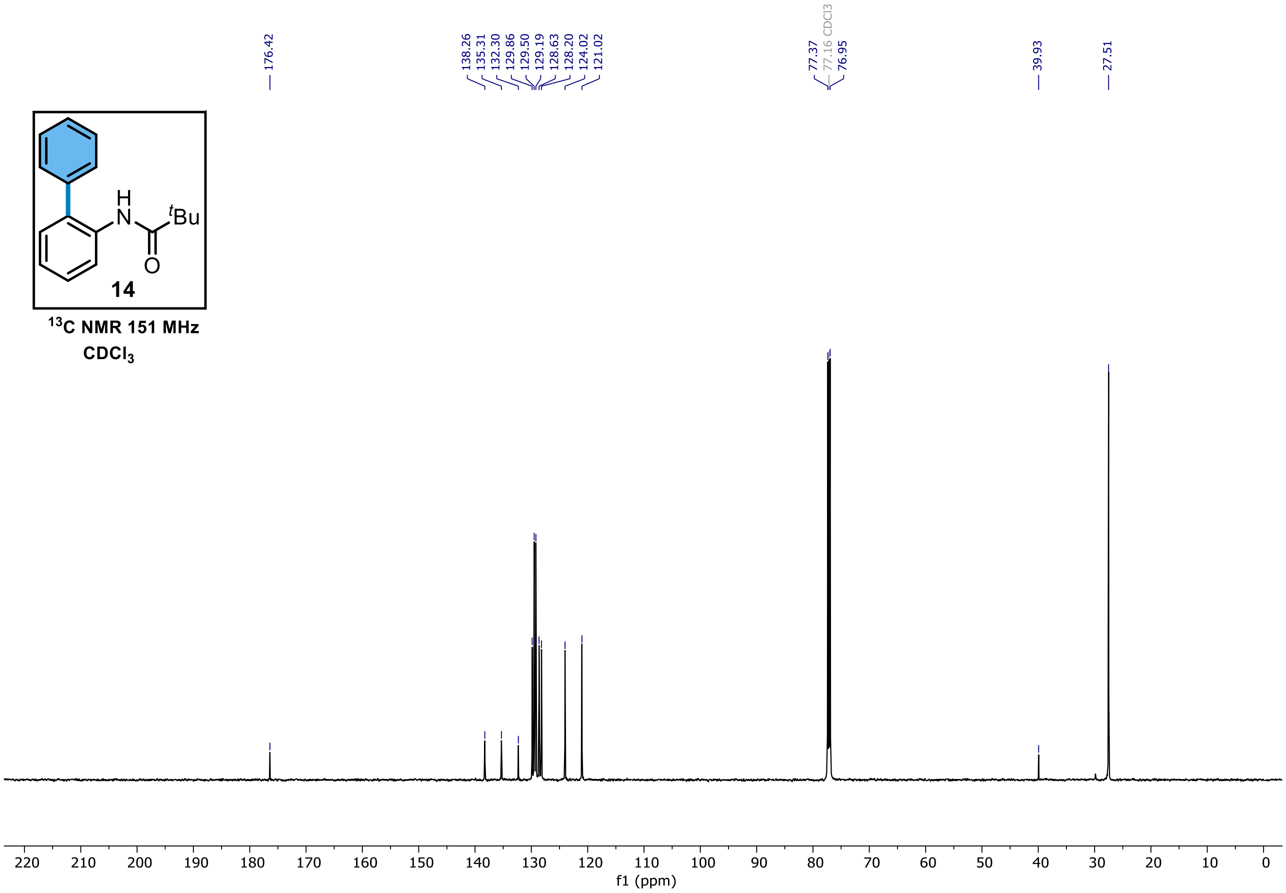
176.42
138.26 135.31 132.30 129.86 129.50 129.19 128.63 128.20 124.02 121.02
77.37 77.16 CDCl3 76.95
39.93
27.51
H
N
tBu
O
14
13C NMR 151 MHz
CDCl3
f1 (ppm)

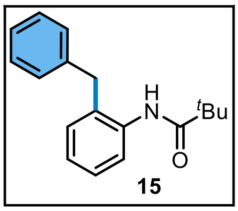

^{1}H NMR 600 MHz
$CDCl_3$

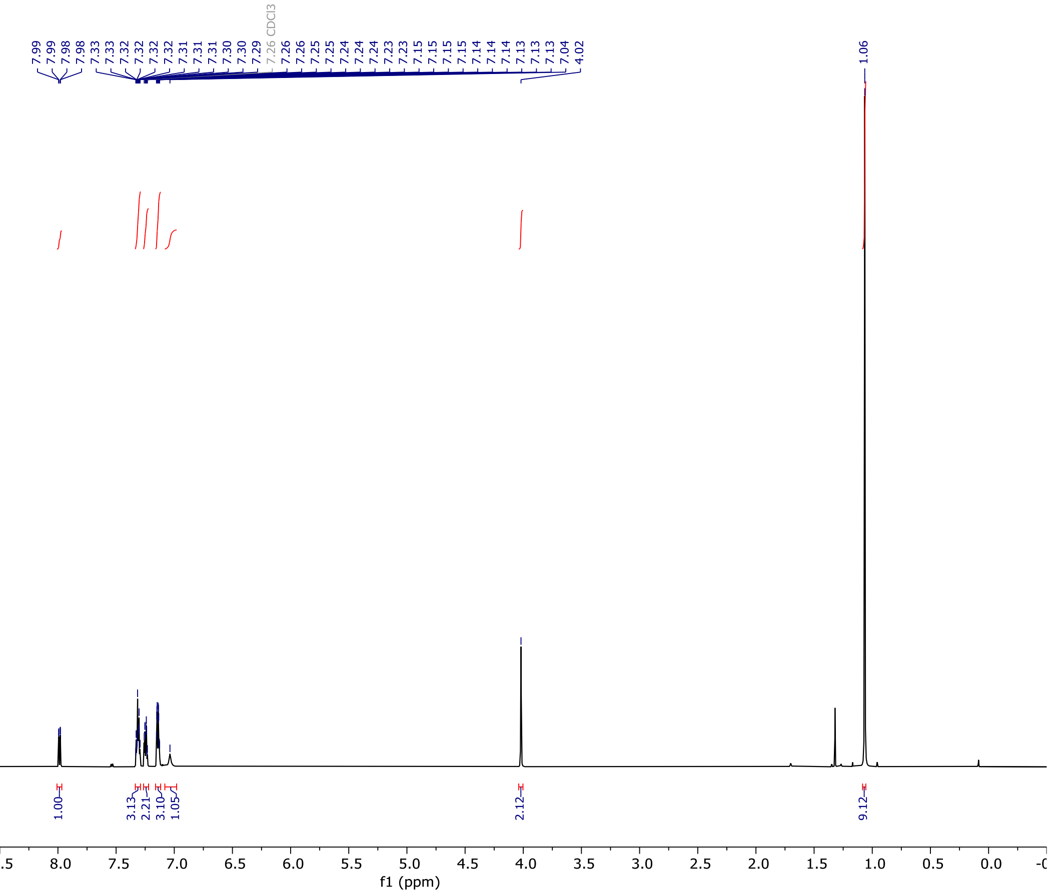

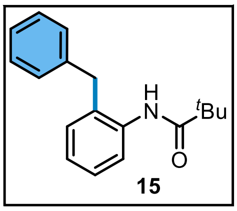

^{13}C NMR 151 MHz
$CDCl_3$

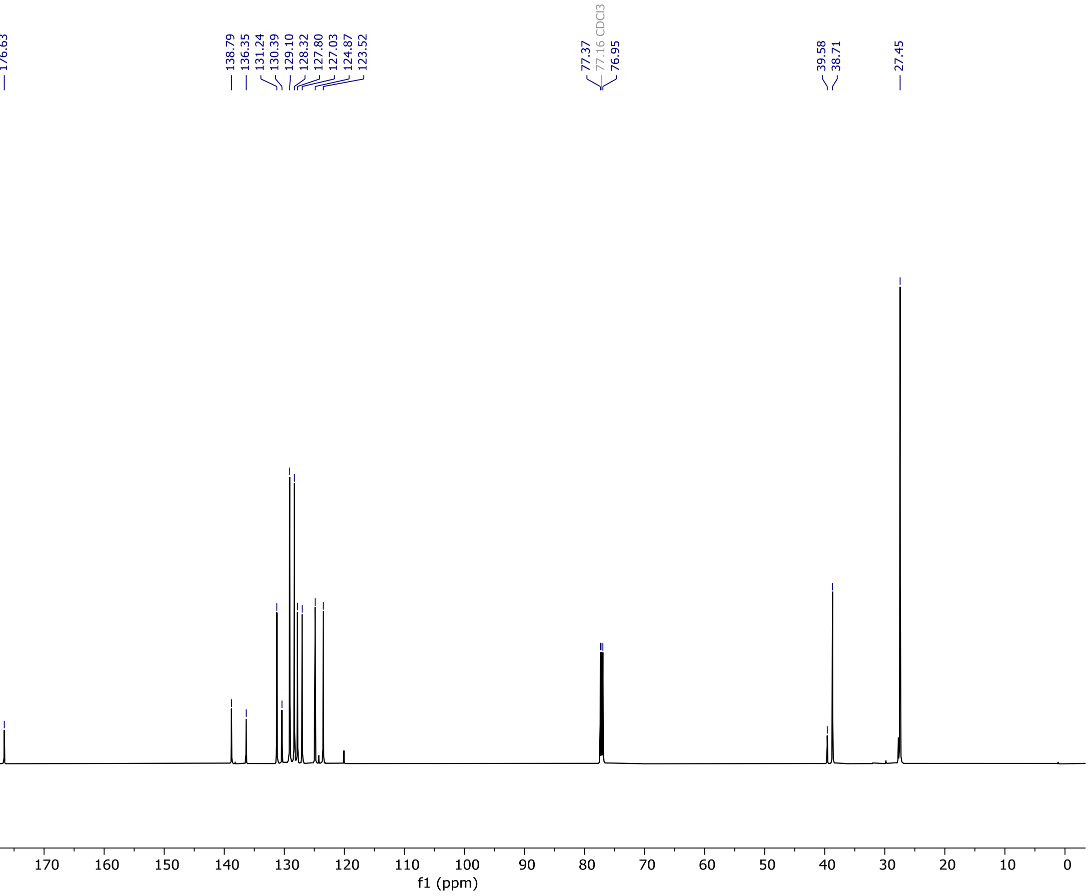

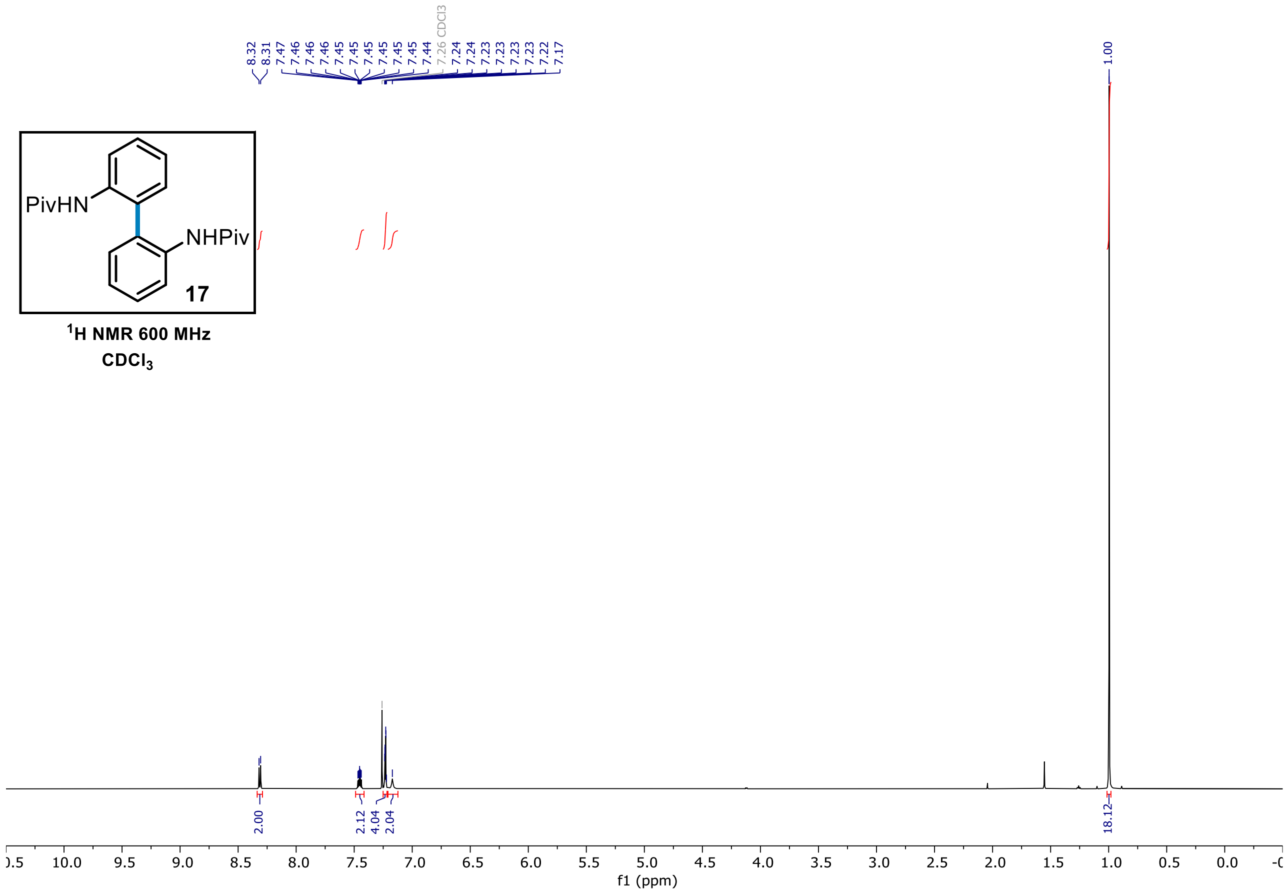
8.32
8.31
7.47
7.46
7.46
7.46
7.45
7.45
7.45
7.45
7.45
7.45
7.44
7.26 CDCl3
7.24
7.24
7.24
7.23
7.23
7.23
7.23
7.23
7.22
7.17
1.00
PivHN
NHPiv
17
^{1}H NMR 600 MHz
$CDCl_3$
2.00
2.12
4.04
2.04
18.12
f1 (ppm)

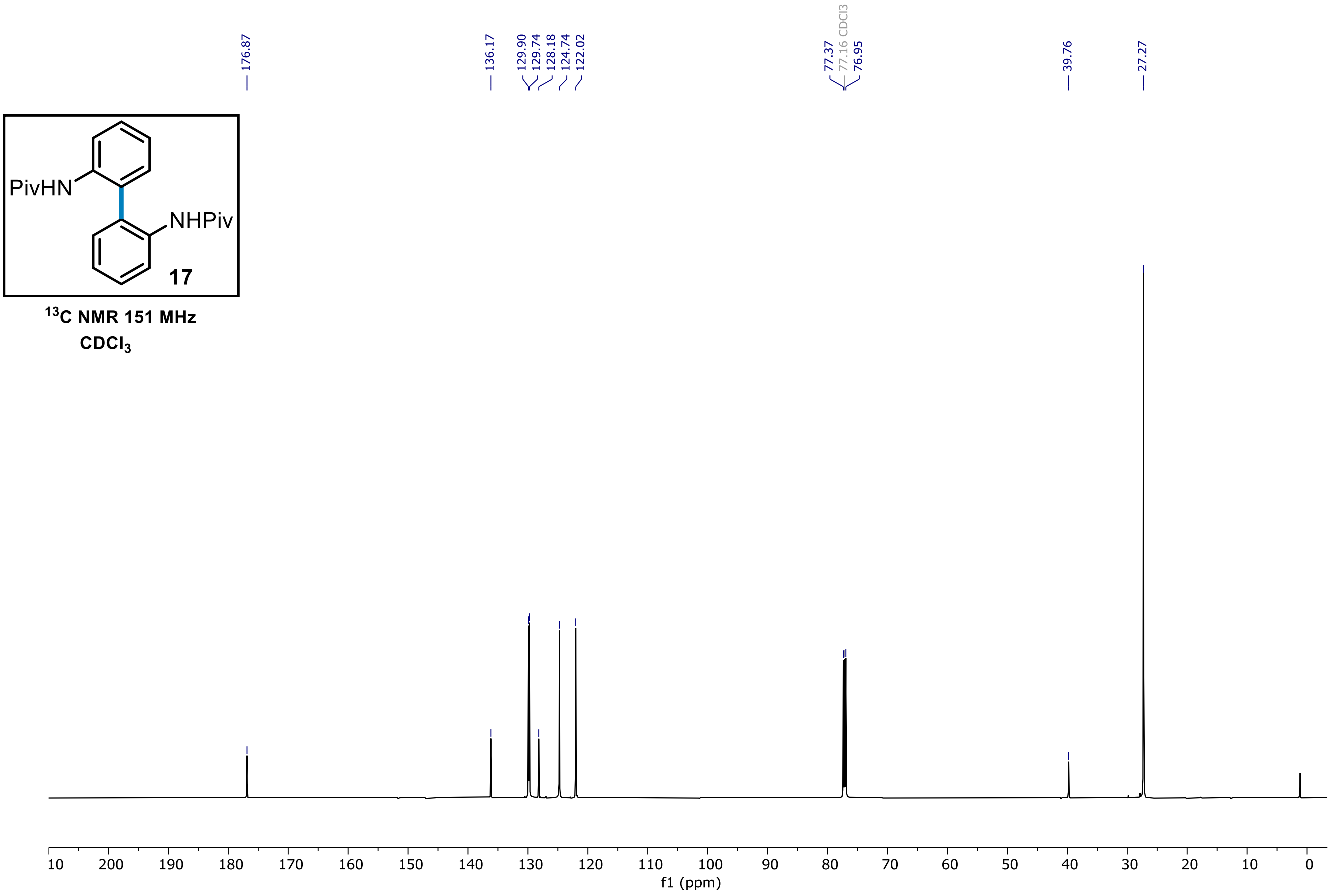
176.87
136.17
129.90
129.74
128.18
124.74
122.02
77.37
77.16 CDCl3
76.95
39.76
27.27
PivHN
NHPiv
17
^{13}C NMR 151 MHz
$CDCl_3$
f1 (ppm)

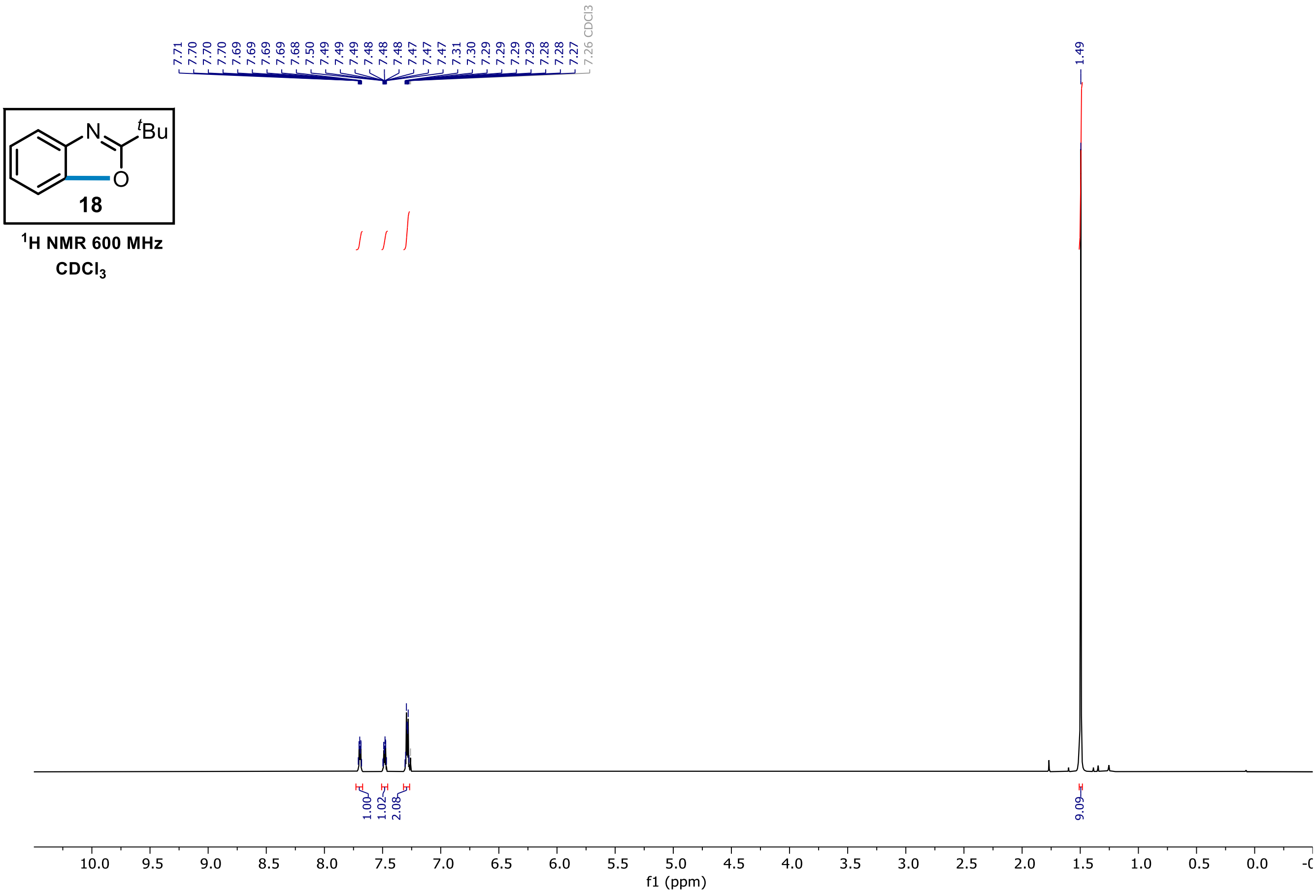
tBu
N
O
18
1H NMR 600 MHz
CDCl3
7.71
7.70
7.70
7.70
7.69
7.69
7.69
7.69
7.68
7.50
7.49
7.49
7.49
7.48
7.48
7.48
7.47
7.47
7.47
7.31
7.30
7.29
7.29
7.29
7.29
7.28
7.28
7.27
7.26 CDCl3
1.49
1.00
1.02
2.08
9.09
f1 (ppm)

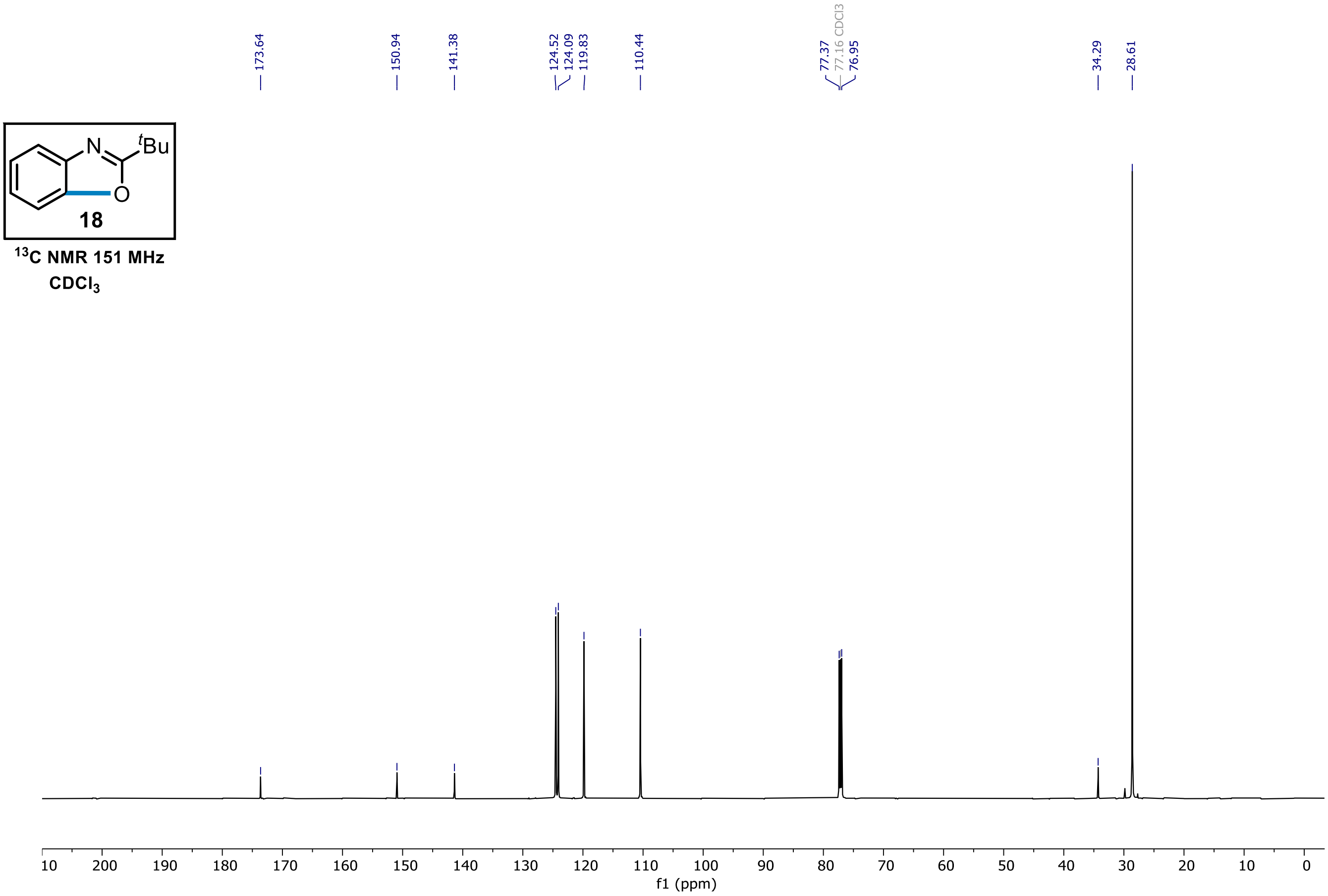
tBu
N
O
18
13C NMR 151 MHz
CDCl3
173.64
150.94
141.38
124.52
124.09
119.83
110.44
77.37
77.16 CDCl3
76.95
34.29
28.61
f1 (ppm)

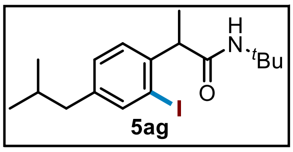

^{1}H NMR 600 MHz
$CDCl_3$

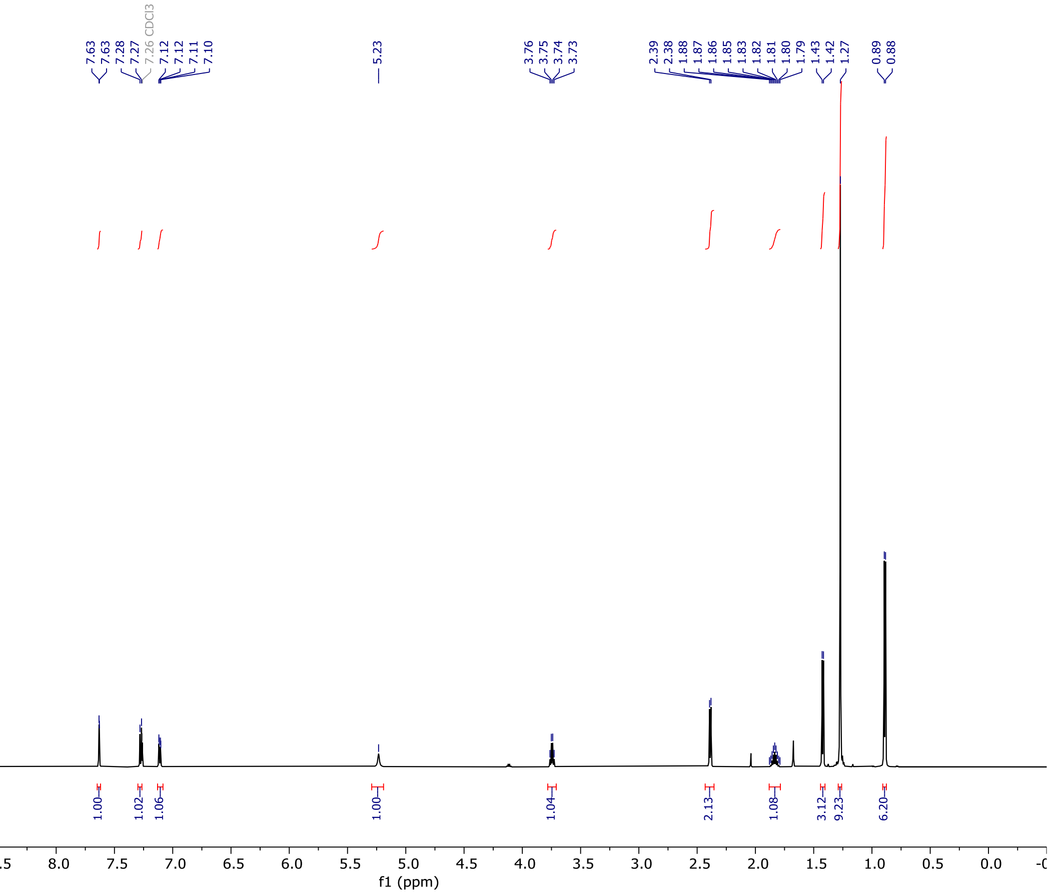

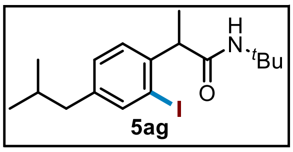

^{13}C NMR 151 MHz
$CDCl_3$

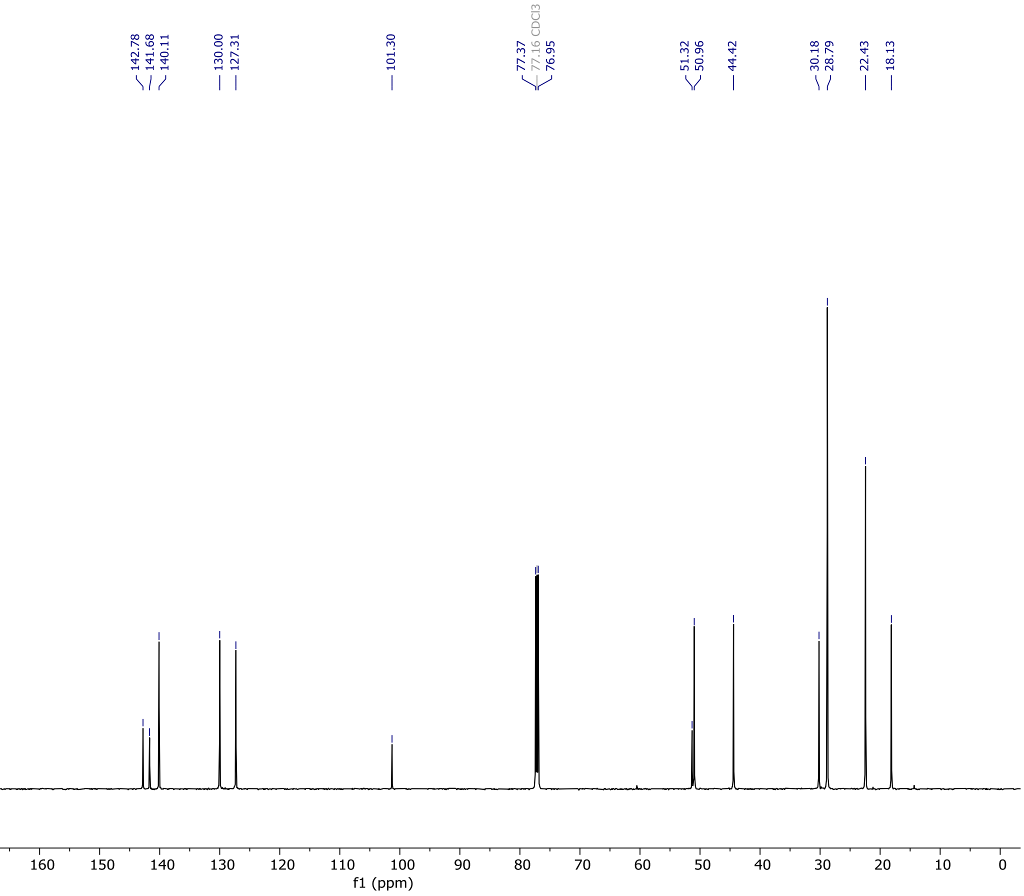

Supplement: Supplementary file 1 — Supporting Information [file ANIE-65-e18421-s001.pdf]
